# MONet: cancer driver gene identification algorithm based on integrated analysis of multi-omics data and network models

**DOI:** 10.3389/ebm.2025.10399

**Published:** 2025-02-04

**Authors:** Yingzan Ren, Tiantian Zhang, Jian Liu, Fubin Ma, Jiaxin Chen, Ponian Li, Guodong Xiao, Chuanqi Sun, Yusen Zhang

**Affiliations:** School of Mathematics and Statistics, Shandong University, Weihai, Shandong, China

**Keywords:** pan-cancer, driver genes, multi-omics data, graph convolutional network, graph attention network

## Abstract

Cancer progression is orchestrated by the accrual of mutations in driver genes, which endow malignant cells with a selective proliferative advantage. Identifying cancer driver genes is crucial for elucidating the molecular mechanisms of cancer, advancing targeted therapies, and uncovering novel biomarkers. Based on integrated analysis of Multi-Omics data and Network models, we present MONet, a novel cancer driver gene identification algorithm. Our method utilizes two graph neural network algorithms on protein-protein interaction (PPI) networks to extract feature vector representations for each gene. These feature vectors are subsequently concatenated and fed into a multi-layer perceptron model (MLP) to perform semi-supervised identification of cancer driver genes. For each mutated gene, MONet assigns the probability of being potential driver, with genes identified in at least two PPI networks selected as candidate driver genes. When applied to pan-cancer datasets, MONet demonstrated robustness across various PPI networks, outperforming baseline models in terms of both the area under the receiver operating characteristic curve and the area under the precision-recall curve. Notably, MONet identified 37 novel driver genes that were missed by other methods, including 29 genes such as APOBEC2, GDNF, and PRELP, which are corroborated by existing literature, underscoring their critical roles in cancer development and progression. Through the MONet framework, we successfully identified known and novel candidate cancer driver genes, providing biologically meaningful insights into cancer mechanisms.

## Impact statement

The mechanisms underlying cancer development are complex, and identifying cancer driver genes is crucial for cancer diagnosis and personalized treatment. Therefore, we have developed a novel cancer driver gene identification algorithm called MONet, based on the comprehensive analysis of multi-omics data and network models. Our results demonstrate that MONet identifies a substantial number of confirmed and potential cancer driver genes with superior performance and reveals new driver genes that other methods have missed. Conducting biomedical experimental research on the new driver genes discovered by MONet can aid precision medicine and provide better treatment options for cancer patients.

## Introduction

The progression of cancer is driven by mutations in specific genes, known as cancer driver genes, that confer growth advantages to malignant cells [1–3]. Identifying these driver genes is crucial for disease diagnosis and personalized treatment, making it a primary objective of cancer genomics research [4–6]. Large-scale collaborative efforts such as The Cancer Genome Atlas (TCGA) [7] and the International Cancer Genome Consortium (ICGC) [8] have amassed unprecedented datasets, furnishing comprehensive resources for cancer driver gene discovery. Over the past decade, researchers have developed a multitude of computational methods to identify potential cancer driver genes, often grounded in experimental hypotheses. For instance, frequency-based methods typically assume that driver genes exhibit recurrent mutations at a higher frequency than non-driver genes [9–11]. In contrast, network-based methods hypothesize that cancer results from alterations in multiple genes that interact closely and play key roles in cancer-related biological pathways, rather than single-gene alterations [12]. These complementary approaches have collectively enriched our understanding of the complex and multifactorial nature of cancer. Computational methods based on gene mutation frequency have been widely applied to identify cancer driver genes. For example, Dees ND et al. developed MuSiC [9], an integrated mutation analysis tool that combines standardized sequence-based data with clinical data to infer relationships between mutations, affected genes, and pathways. This allows researchers to prioritize driver genes and distinguish significant driver mutations from passenger mutations. Tamborero D et al. proposed OncodriveCLUST [11], which uses silent mutations in coding regions as a background mutation model to identify genes with mutation frequencies significantly exceeding the background rate in specific protein regions. Lawrence MS et al. proposed the MutSigCV [10] algorithm, which is based on the mutation frequency and lineage of specific patients. This algorithm uses a background mutation model that incorporates gene expression and replication timing information to adjust for variations, thereby calculating the background mutation rate of specific genes to improve the accuracy of identifying cancer-related genes.

In recent years, through network analysis, researchers can identify cancer driver genes, a process that is vital for understanding the mechanisms and progression of cancer [13]. Representative algorithms for driver gene identification based on pathway and network analysis include the following. Leiserson MD et al. proposed the HotNet2 [14] algorithm, which is designed to identify mutated subnetworks within gene interaction networks. HotNet2 considers the weights of mutations within single protein networks, enhancing its ability to identify and understand key roles within mutated subnetworks. Cho A et al. introduced the MUFFINN [15] algorithm, which integrates mutation information of individual genes with that of neighboring genes in functional networks to identify driver genes. Colaprico A et al. developed the Moonlight [16] algorithm, designed to identify cancer driver genes that act as dual-role players within the transcriptome network.

In current cancer research, methods that integrate multi-omics data and biological network analysis are widely used for cancer driver gene identification. These methods not only enhance our understanding of cancer development mechanisms but also provide new strategies and approaches for personalized treatment. Therefore, combining multi-omics data integration with biological network analysis is becoming an inevitable trend in exploring cancer complexity. EMOGI [17] is an interpretable machine learning method based on graph convolutional networks (GCN) that integrates genomics, epigenomics, and transcriptomics data as gene features and combines them with PPI networks to learn more abstract gene features. MTGCN [18] is a multi-task learning framework based on GCN that optimizes both node classification and edge link prediction tasks by learning node embedding features. These methods have shown promising results, confirming the effectiveness of combining multi-omics data with network models for cancer driver gene identification.

Nevertheless, despite the efficacy of graph neural network-based methods, their predictive performance in cancer driver gene identification can be limited by the inherent complexity of biological networks. To address this, we propose MONet, which integrates both graph convolutional networks and graph attention networks to enhance the representational power of gene features through the concatenation of feature vectors. Additionally, we selected six independent PPI networks for model training, ensuring that the predicted candidate driver genes are comprehensive and accurate. Through MONet, we identified 376 candidate driver genes, 184 of which are known driver genes recorded by multiple benchmarks. Among remaining 192 predicted genes, most of them are supported by other datasets or corroborative studies, highlighting the potential of MONet in cancer driver gene identification.

## Materials and methods

### Multi-omics data and PPI networks

We utilized the same multi-omics data and PPI networks as EMOGI to predict cancer driver genes. For the sake of completeness, we briefly introduce these data.

Our method employed four types of multi-omics data: somatic mutation (SM), copy number variation (CNV), gene expression (GE), and DNA methylation (DNAm). We integrated these four types of multi-omics data from 16 cancer types: BLCA, BRCA, CESC, COAD, ESCA, HNSC, KIRC, KIRP, LIHC, LUAD, LUSC, PRAD, READ, STAD, THCA, and UCEC. After normalizing these datasets, we concatenated them to form a feature matrix, where rows represent genes and columns represent features.

We collected protein-protein interactions from CPDB [19], STRING-db [20], MultinetI [21], IRefIndex [22], and PCNet [23]. Depending on the network, we only considered high-confidence interactions. For CPDB, interactions with a score higher than 0.5 are retained. For STRING-db, interactions with a score higher than 0.85 are kept. Multinet and the older version of IRefIndex (v.9.0) were collected from the HotNet2 GitHub repository. For the newer version of IRefIndex (v.15.0), only interactions between human proteins were considered. PCNet was used without further processing and serves as a consensus network.

### Benchmark datasets

In the absence of a recognized “gold standard” dataset containing both positive and negative driver gene annotations, it is challenging to accurately assess the performance of previous prediction tools [24, 25]. To comprehensively evaluate our method, we utilized three commonly used datasets as benchmark datasets, and their union was used as the source of positive samples. For ease of comparison, the datasets were used in the same versions as EMOGI. These datasets include CGC [6], NCG [26], and DigSEE [27]. The CGC database manually curates a list of 723 common genes causally implicated in cancer. The NCG database contains a curated list of expert-selected and candidate cancer genes, with the included genes being proven or predicted to be drivers of cancer. In the selection of positive samples, only confirmed cancer driver genes were used. DigSEE was employed to search for genes related to cancer in the PubMed database, restricted to the 16 cancer types, and a set of 85 highly confident cancer genes was identified using DNA methylation and gene expression as evidence.

Negative samples represent genes least likely to be associated with cancer. To generate a list of negative samples, potential cancer-related genes were recursively removed from all genes, including those present in the NCG database, genes related to cancer pathways in the KEGG [28]database, genes in the OMIM [29] disease database, genes predicted to be cancer-related in MutSigdb [30], and genes whose expression is correlated with cancer genes [31].

Since the proposed algorithm is trained using different PPI networks, only positive and negative samples contained within the underlying PPI networks were used for training. Table 1 presents the total number of genes included in the PPI networks used, along with the counts of positive samples, negative samples, and unlabeled genes.

**TABLE 1:** The total number of genes included in the PPI networks, along with the counts of positive samples, negative samples, and unlabeled genes.

PPI	Number	Positive	Negative	Unlabeled
CPDB	13,627	796	2,187	10,644
STRING	13,179	783	2,415	9,981
Multinet	14,398	790	3,709	9,899
IRefIndex	17,013	836	4,056	12,121
IRefIndex (2015)	12,129	785	1,971	9,373
PCNet	19,781	859	3,921	15,001

### Overview of MONet

**FIGURE 1:**
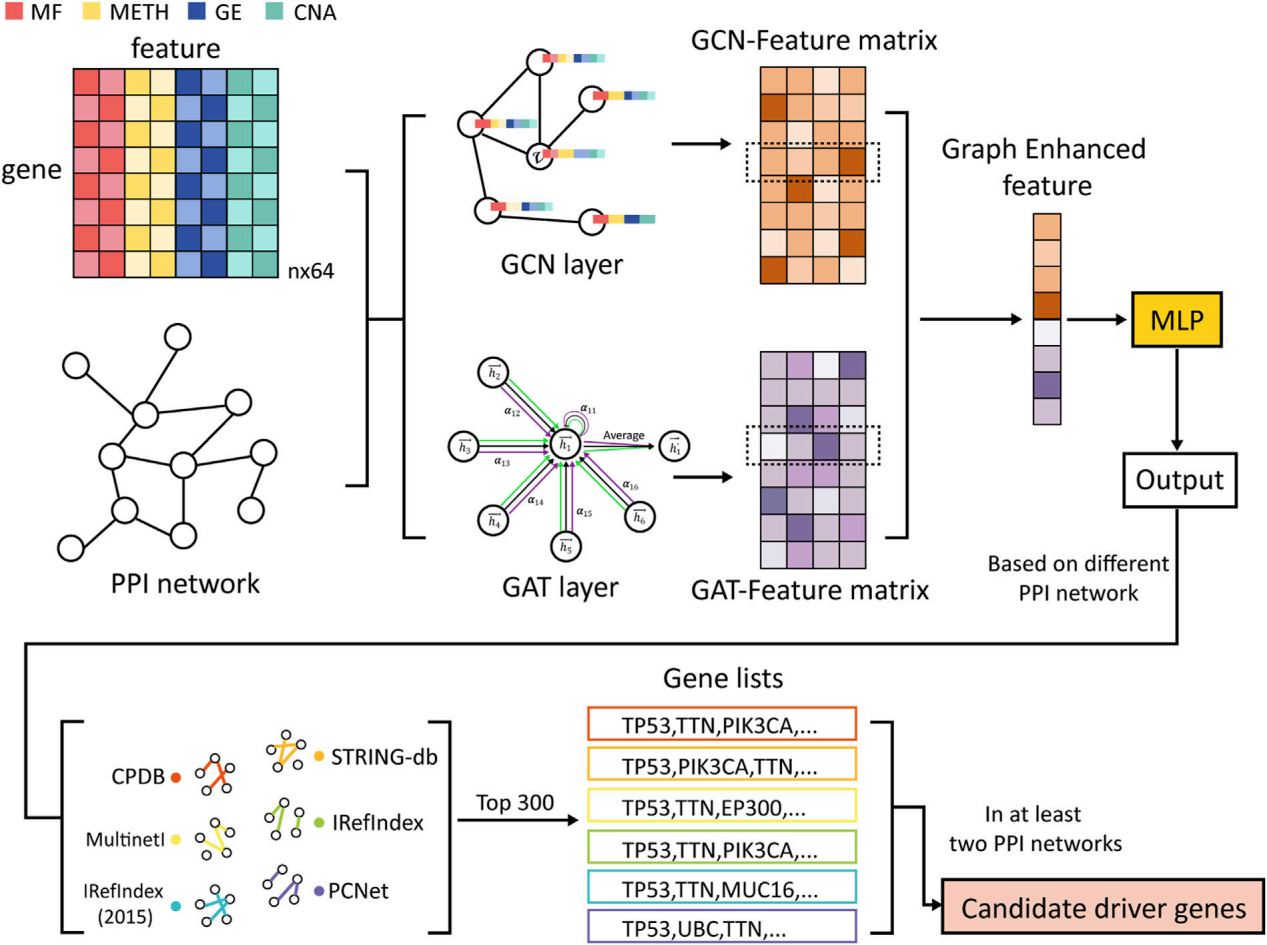
Overview of MONet. First, the graph structures of the PPI networks are learned using GCN and GAT to obtain feature vector representations for each gene. Then, these feature vectors from the two matrices are concatenated together to form a new feature vector. Subsequently, the new feature vectors are input into an MLP for the driver gene identification task. Finally, the top 300 candidate genes are selected from each of the 6 PPI networks, and genes appearing in at least two networks are designated as candidate driver genes. The multi-omics data include Mutation Features (MF), DNA Methylation Features (METH), Gene Expression Features (GE), and Copy Number Alteration Features (CNA).

Figure 1 illustrates the main workflow of MONet. Firstly, the graph structure constructed from the PPI network is fed into two graph neural network algorithms, GCN and GAT, to learn the feature vector representations of each gene, resulting in two feature matrices: the GCN-Feature matrix and the GAT-Feature matrix. Next, the feature vectors obtained from the two matrices are concatenated together gene-wise, forming a new feature vector for each gene, termed as the Graph Enhanced feature. Subsequently, the new feature vectors are inputted into a multilayer perceptron (MLP) model to perform semi-supervised cancer driver gene identification tasks, comprehensively learning the node features. This process yields the probability of each gene being predicted as a cancer driver gene, and genes are ranked based on these probabilities. Finally, the top 300 candidate genes from each of the six PPI networks are selected, and genes that appear in at least two networks are considered as candidate driver genes.

### GCN layer and GAT layer

GCN algorithm can preserve the structural information of the graph. Therefore, we first use the GCN algorithm on the graph structure constructed from the PPI network to learn the feature vector representation of each gene.

GCN learns node features through the following steps. First, the initial graph structure data $G=\left(V,E\right)$ is mapped to a new space $f^{G}\to f^{*}$ . Taking a single-layer forward propagation graph convolutional neural network as an example, the feature of the i -th layer neural network is represented by $w_{i}$ . When computing the nodes $v_{i}$ in the graph, the output $H^{l+1}$ of each layer of the network can be represented by a nonlinear function $f\left(∙,∙\right)$ as $H^{l+1}=f\left(H^{l},A\right)$ , where A is the adjacency matrix. The graph convolutional neural network structure is realized through a nonlinear activation function $\sigma \left(∙\right)$ , and its layer-wise propagation rule is given in Equation 1.

$$f\left(H^{l+1},A\right)=\sigma \left({\tilde{D}}^{-\frac{1}{2}}\tilde{A}{\tilde{D}}^{-\frac{1}{2}}H^{l}W^{l}\right)$$ (1)

where $\tilde{A}=A+I$ represents the adjacency matrix of graph G , I represents the identity matrix, $\tilde{D}=\sum {\tilde{A}}_{ij}$ represents the degree matrix of the adjacency matrix $\tilde{A}$ , and $W^{l}$ represents the weight matrix of the convolutional neural network at layer l .

The GCN algorithm relies on two input matrices: the adjacency matrix of the network and the feature matrix composed of the features of each node. This allows GCN to preserve the structural information of the graph, fully exploiting the latent information in the PPI network, thereby enhancing the classification performance for genes. However, during the learning process, GCN assigns equal importance to all nodes within the same neighborhood. In practical applications, we need an algorithm that can more intelligently discern the importance of neighboring nodes, which is where GAT excels.

In MONet, we used the GAT method with integrated multi-head attention mechanism. In GAT, for a single graph attention layer, the input consists of a set of node features: $h=\left\{\vec{h_{1}},\vec{h_{2}},...,\vec{h_{N}}\right\},\vec{h_{i}}\in R^{F}$ , where N is the number of nodes and F represents the dimensionality of each node’s feature vector. This layer generates a new set of node features (with feature dimension F’ ): $h^{’}=\left\{\vec{h_{1}^{\prime }},\vec{h_{2}^{\prime }},...,\vec{h_{N}^{\prime }}\right\},\vec{h_{i}^{\prime }}\in R^{F^{\prime }}$ . The attention layer propagation mechanism employed in this study is based on Equations 2–5.

$$e_{ij}=a\left(W\vec{h_{i}},W\vec{h_{j}}\right)$$ (2)

$$e_{ij}=Leaky\text{Re}LU\left({\vec{a}}^{T}\left[W\vec{h_{i}}∥W\vec{h_{j}}\right]\right)$$ (3)

$$\alpha_{ij}={softmax}_{j}\left(e_{ij}\right)=\frac{\exp\left(e_{ij}\right)}{\sum_{k\in N_{i}}\exp\left(e_{ik}\right)}$$ (4)

$$\vec{h_{i}^{\prime }}=\sigma \left(\frac{1}{K}\sum_{K=1}^{K}\sum_{j\in N_{i}}\alpha_{ij}^{k}W^{k}\vec{h_{j}}\right)$$ (5)

where $e_{ij}$ represents the importance of node j to node i , W and $\vec{a}$ are trainable parameters, K represents the number of attention heads, and ∥ denotes the concatenation operation.

Enhancing the GAT method through the integration of the multi-head attention mechanism brings several significant advantages. Firstly, introducing multiple attention heads allows for parallel processing of different aspects of the graph, thus improving computational efficiency. Each head focuses on learning different features, achieving a comprehensive representation of the graph’s complex structure. Additionally, the multi-head mechanism enhances the model’s robustness and generalization ability by integrating diverse attention distributions. Even in the presence of noise or errors in individual heads, collective insights from multiple heads ensure the model’s resilience. Lastly, and most importantly, introducing multi-head attention mechanism can enhance the expressive power of the attention layers.

### Enhanced feature

By applying both the GCN layer and the GAT layer, the original gene feature vectors are transformed, resulting in two new feature matrices: the GCN-Feature matrix and the GAT-Feature matrix, where the rows represent genes and the columns represent new features. To fully leverage the gene feature vector representations learned by GCN and GAT, MONet innovatively concatenates these learned feature vectors into a single new feature vector, thereby enhancing the gene features. This new feature vector is termed the Graph Enhanced feature.

GCN and GAT are two distinct types of graph neural network algorithms, each employing different methods for information aggregation. GCN aggregates information from neighboring nodes based on the graph’s Laplacian spectrum (or adjacency matrix), making it well-suited for capturing global structural features of the graph, particularly excelling in learning global relationships based on the graph topology. In contrast, GAT utilizes a self-attention mechanism to dynamically assign weights to each neighboring node, enabling it to flexibly capture the importance of local neighbors and highlight critical interactions between nodes. Therefore, GCN focuses more on learning global structures, while GAT emphasizes the importance of local interactions between nodes. By concatenating the feature vectors learned by both algorithms, the Graph Enhanced feature integrates the strengths of both methods, providing a more comprehensive description of gene characteristics. Our subsequent comparisons reveal that the feature vectors learned by GCN and GAT may possess complementary properties. By combining these features, we can address the limitations of features learned by each algorithm individually, thereby enhancing the overall feature representation. MONet achieved better results compared to using GCN and GAT alone.

### Identification and screening of driver genes

MLP is an artificial neural network composed of multiple layers of perceptrons or neurons. Each layer is fully connected to the next. A basic perceptron model includes three components: input values, weights and biases, and an activation function. Each perceptron receives a set of inputs, multiplies these inputs by the corresponding weights, and then adds a bias. This result is passed through an activation function to produce an output value. The training of an MLP typically involves the backpropagation algorithm and gradient descent optimization. In MONet, the integrated gene feature vectors, referred to as Graph Enhanced features, are input into an MLP to perform a semi-supervised cancer driver gene identification task. This process fully learns the node features, ultimately producing a probability for each gene being a cancer driver gene. The genes are then ranked based on these probability values.

Selecting candidate driver genes that appear in multiple PPI networks helps to reduce the influence of randomness and noise from individual networks. Additionally, by screening across different networks and choosing genes with high occurrence frequency as candidate driver genes, the consistency and reproducibility of the results are increased, thereby enhancing the credibility of the study. Consequently, we applied the MONet to the graph structures constructed from the six PPI networks used in this study, resulting in six sets of gene rankings. We selected the top 300 genes from each ranking as candidate genes and included those that appeared in at least two sets as candidate driver genes for further analysis.

## Results

### Evaluation metrics

We evaluated the performance of MONet using common evaluation metrics, including accuracy, the area under the receiver operating characteristic curve (AUROC), the area under the precision-recall curve (AUPR), the F1 Score, and the Matthews Correlation Coefficient (MCC). AUROC represents the area under the receiver operating characteristic (ROC) curve, which is an important indicator for measuring classification performance. By computing the true positive rate (TPR) and false positive rate (FPR) and generating the ROC curve, we calculated the area under it. The precision-recall (PR) curve illustrates the relationship between precision and recall at different thresholds, and the area under it represents the AUPR value. The F1 Score and MCC are particularly well-suited for imbalanced datasets. The F1 Score evaluates a model’s ability to predict positive samples by combining precision and recall into a single metric. MCC provides a comprehensive assessment of a model’s predictive performance by incorporating all elements of the confusion matrix, including true positives (TP), true negatives (TN), false positives (FP), and false negatives (FN). These evaluation metrics comprehensively assess the classification performance and predictive capability of the model. Several indicators are introduced below.

$$Accuracy=\frac{TP+TN}{TP+FP+TN+FN}$$ (6)

ROC curve according to the following equation:

$$\left\{\begin{array}{l} \;TPR=\frac{TP}{TP+FN}\\ \;FPR=\frac{FP}{TP+FP} \end{array}\right.$$ (7)

PR curve according to the following equation:

$$\left\{\begin{array}{l} \;Precision=\frac{TP}{TP+FP}\\ Recall=\frac{TP}{TP+FN}\; \end{array}\right.$$ (8)

$$F1\;Score=2\;\cdot \;\frac{Precision\;\cdot \;Recall}{Precision+Recall}$$ (9)

$$MCC=\frac{TP\;\cdot \;TN-FP\;\cdot \;FN}{\sqrt{\left(TP+FP\right)\left(TP+FN\right)\left(TN+FP\right)\left(TN+FN\right)}}$$ (10)

where True Negative (TN), True Positive (TP), False Negative (FN), and False Positive (FP), respectively, are in Equations 6–10.

### Model training

In this study, we used a binary cross-entropy loss function $L=-\left(cylog\sigma \left(x\right)+\left(1-y\right)\log\left(1-\sigma \left(x\right)\right)\right)$ . Due to the imbalance between positive and negative samples in the training data, we applied a weight c to the positive samples in the loss function. For instance, in the CPDB network, there are 796 positive samples and 2,187 negative samples. Since the negative samples are approximately three times the number of positive samples, we assigned a weight of 3 to the positive samples in the loss function (c = 3). When training MONet, we first divided the labeled samples, i.e., the total of positive and negative samples, into 75% for the training set and 25% for the test set. We employed ten-fold cross-validation to train the model and calculated the average results of the test sets from the ten folds to evaluate the model’s performance.

For the graph structure constructed from the CPDB network, after tuning the parameters, we found that in GCN, the number of hidden layers is 2, with dimensions of 300 and 100, respectively. The dimension of the GCN output layer is set to 16. In GAT, there is one hidden layer with a dimension of 100, incorporating a multi-head attention mechanism, with 5 heads in the hidden layer and 1 head in the output layer. The output layer of the GAT has a dimension of 16. We concatenated the output vectors from GCN and GAT to form a 32-dimensional vector, which was then fed into an MLP. The MLP has one hidden layer with a dimension of 16. Finally, the MLP outputs the probability that each node is predicted to be positive, which corresponds to the probability of each gene being a cancer driver gene. We ranked the genes based on these probability values to identify candidate cancer driver genes. During training, we used the Adam optimizer with a learning rate of 0.001, a weight decay rate of 0.0005, and a dropout rate of 0.5. We set the epochs to 2000 and used the validation set loss as the criterion for early stopping.

### Performance on PPI network

**FIGURE 2:**
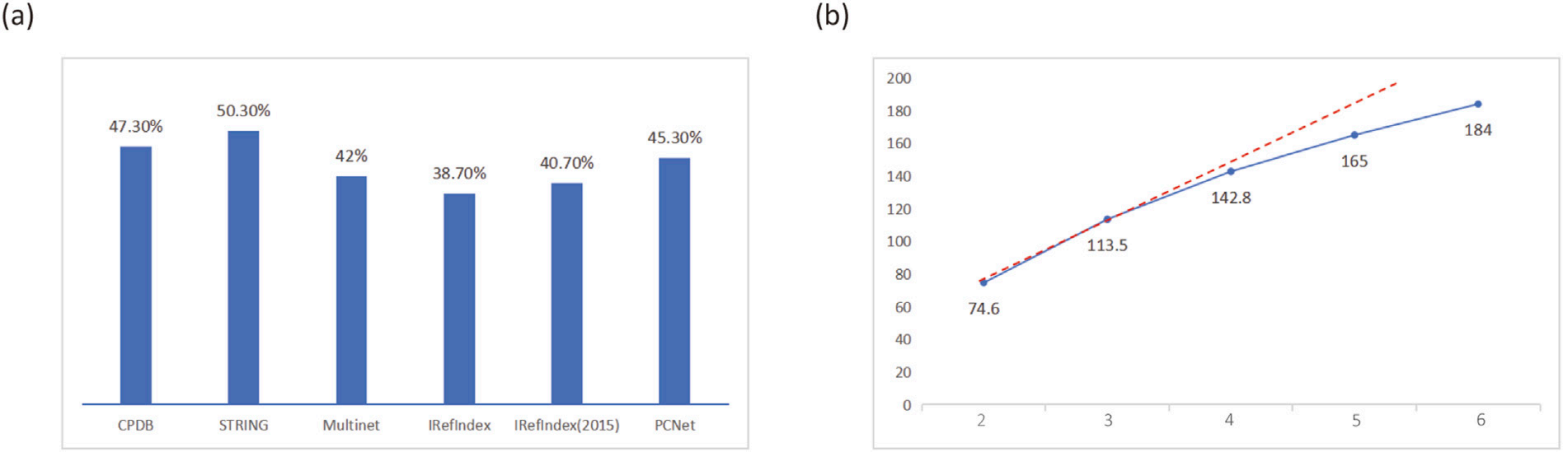
Impact of different PPI networks on MONet performance. **(A)** Proportion of known driver genes among the predicted driver genes identified using a single PPI network. **(B)** Trend in the number of known driver genes identified when using 2–6 PPI networks, with the results for 2–5 networks represented as the average.

To demonstrate the necessity of using multiple PPI networks, we conducted tests with varying numbers of PPI networks. The results showed that when using a single PPI network, only a small proportion of the top 300 genes predicted by MONet were confirmed as known driver genes in the reference dataset. Moreover, the performance varied significantly across different networks, with the proportion of known driver genes reaching 38.7% for IRefIndex but 50.3% for STRING (Figure 2A), indicating suboptimal overall performance. When multiple PPI networks were used, and genes appearing in at least two networks were selected as candidate driver genes, the proportion of known driver genes identified was higher than that achieved with a single network (Supplementary File S1). This demonstrates that integrating multiple networks is more advantageous for identifying driver genes.

Figure 2B shows the trend in the number of known driver genes identified by MONet when using 2–6 PPI networks, with results for 2–5 networks represented as averages. It can be observed that as the number of PPI networks increased, the number of identified known driver genes also grew. This may be attributed to the inclusion of additional protein-protein interaction pathways from newly added networks, which are potentially associated with driver gene functionality. However, the growth rate gradually diminished, suggesting that increasing the number of PPI networks does not always lead to continuous performance improvement. Since MONet’s performance is highly dependent on the quality of PPI networks, it is significantly influenced by the accuracy and reliability of the network data. With the ongoing advancements in medical experiments, the diversity and quantity of PPI networks are continuously expanding. Given MONet’s strong performance, we believe it can be applied to a broader range of PPI network structures, providing robust support for driver gene research.

To evaluate the capability of the proposed MONet method in predicting cancer driver genes, we employ five performance metrics: accuracy (ACC), AUROC, AUPR, F1 Score and MCC. In our study, we train MONet method on six different PPI networks and evaluate its performance on each network. By training and evaluating on multiple networks, we can comprehensively understand MONet’s generalization ability and robustness. The performance metrics for MONet on each PPI network are summarized in Table 2. These metrics will help us thoroughly assess MONet’s effectiveness in predicting cancer driver genes and provide a critical reference for further experimental results.

**TABLE 2:** Performance of MONet on each PPI network. ACC, AUROC, AUPR, F1 score, and MCC values across six PPI networks.

PPI	ACC	AUROC	AUPR	F1 score	MCC
CPDB	0.7909	0.8864	0.7781	0.6750	0.5445
STRING	0.8125	0.9119	0.8069	0.6862	0.5774
Multinet	0.8622	0.9360	0.7825	0.6830	0.6172
IRefIndex	0.8618	0.9053	0.7076	0.6258	0.5432
IRefIndex (2015)	0.8058	0.8805	0.7525	0.7035	0.5740
PCNet	0.9067	0.9379	0.7700	0.6942	0.6456

Observing the table, it can be seen that on CPDB, MONet performs well in terms of AUROC (0.8864) and AUPR (0.7781), though its ACC (0.7909) is slightly lower compared to other networks. On STRING, MONet demonstrates robust performance, particularly excelling in AUPR (0.8069) and achieving high AUROC (0.9119) and ACC (0.8125) values. Its F1 Score (0.6862) and MCC (0.5774) reflect good alignment between predictions and true labels. On Multinet, MONet achieves exceptional performance, with one of the highest AUROC (0.9360) values and strong AUPR (0.7825). The F1 Score (0.6830) and MCC (0.6172) highlight its ability to make balanced predictions. IRefIndex and IRefIndex (2015) show relatively better performance in AUPR compared to other metrics. PCNet emerges as the top-performing network, with the highest ACC (0.9067), AUROC (0.9379), and MCC (0.6456), along with a strong F1 Score (0.6942), reflecting its robust and balanced predictions. Overall, our MONet algorithm performs well on all six PPI networks.

### Ablation experiment

MONet employed multi-omics data for predicting driver genes. To examine whether the inclusion of multi-omics features improves model performance, we conducted ablation experiments. Specifically, we individually inputted single omics type features (namely, Mutation Features (MF), DNA Methylation Features (METH), Gene Expression Features (GE), and Copy Number Alteration Features (CNA)) into the model, as well as combined features of two omics types (namely, MF + METH, MF + GE, MF + CNA, METH + GE, METH + CNA, GE + CNA), combined features of three omics types (namely, MF + METH + GE, MF + METH + CNA, MF + GE + CNA, METH + GE + CNA), and all omics type features. The experimental results are presented in Table 3.

**TABLE 3:** The performance comparison of MONet and its variants in driver gene prediction. The multi-omics features include Mutation Features (MF), DNA Methylation Features (METH), Gene Expression Features (GE), and Copy Number Alteration Features (CNA).

Features	ACC	AUROC	AUPR
MF	0.7708	0.8601	0.7497
METH	0.7547	0.8563	0.7242
GE	0.7480	0.8711	0.7351
CNA	0.7145	0.8055	0.6438
MF + METH	0.7855	0.8790	0.7689
MF + GE	0.7373	0.8300	0.6767
MF + CNA	0.7601	0.8639	0.7546
METH + GE	0.7761	0.8774	0.7430
METH + CNA	0.7399	0.8476	0.7098
GE + CNA	0.7668	0.8697	0.7399
MF + METH + GE	0.7601	0.8666	0.7352
MF + METH + CNA	0.7480	0.8717	0.7610
MF + GE + CNA	0.7413	0.8683	0.7537
METH + GE + CNA	0.7688	0.8714	0.7453
Multi-omics	0.7909	0.8864	0.7781

Table 3 displays the performance comparison of MONet and its variants in pan-cancer driver gene prediction. Firstly, we observed that when various omics features were individually applied to the model, multi-omics exhibited the best model performance. Specifically, the combination of multi-omics features achieved the highest scores in terms of ACC, AUROC, and AUPR, with values of 0.7909, 0.8864, and 0.7781, respectively. This indicates that integrating multiple omics data can better predict genes and improve model performance. Next, we further compared the performance of single omics features. Among single omics features, GE performed the best in terms of AUROC, reaching 0.8711, while CNA performed the worst in terms of ACC and AUPR, with values of 0.7145 and 0.8055, respectively. This may reflect the importance of gene expression data in gene prediction, and the relatively weaker predictive ability of copy number alteration features compared to other omics data. Subsequently, we analyzed the combination effects of various omics type features. We observed that the MF + METH combination exhibited the best comprehensive performance in terms of ACC, AUROC, and AUPR, with values of 0.7855, 0.8790, and 0.7689, respectively. However, the MF + METH + CNA combination achieved the highest score in terms of AUPR, reaching 0.7610, indicating that adding copy number alteration features can improve the performance of gene prediction models in certain scenarios.

Overall, integrating multiple omics data can significantly improve the performance of gene prediction models, and the combination of different omics features may have varying degrees of impact on model performance.

### Comparison with other methods

**FIGURE 3:**
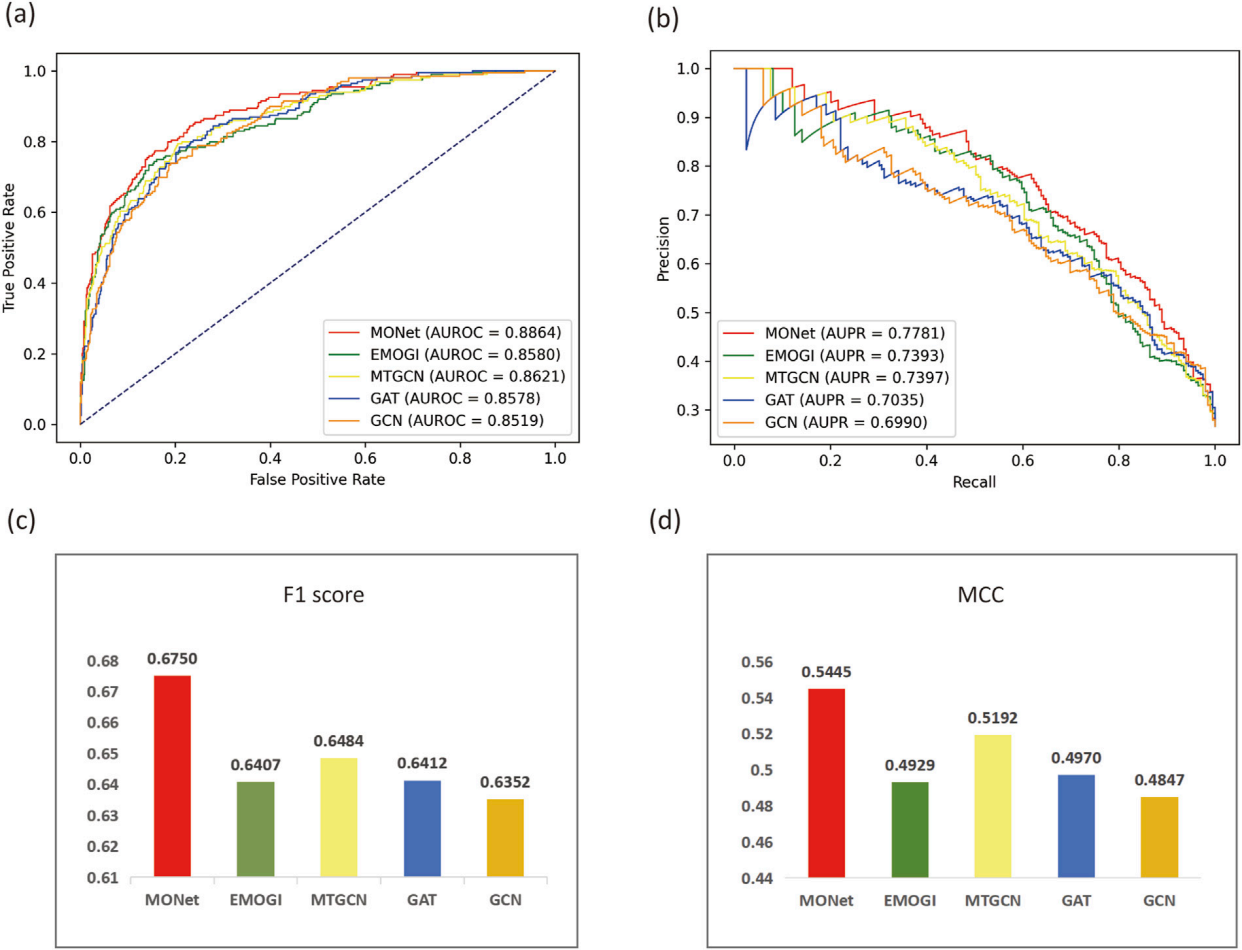
Comparison of MONet with other methods. **(A)** ROC curves and AUROC values of MONet, EMOGI, MTGCN, GAT, and GCN. **(B)** PR curves and AUPR values of MONet, EMOGI, MTGCN, GAT, and GCN. **(C)** F1 Scores of MONet, EMOGI, MTGCN, GAT, and GCN. **(D)** MCC values of MONet, EMOGI, MTGCN, GAT, and GCN.

To further evaluate the performance of MONet, we selected four evaluation metrics, AUROC, AUPR, F1 Score and MCC. For comparison, we chose four other algorithms to compare their performance with MONet, including the EMOGI, MTGCN, GCN, and GAT. The EMOGI and MTGCN method are both graph neural network algorithms based on the integration of multi-omics data, where EMOGI is based on GCN and predicts cancer driver genes using multi-omics data, while MTGCN, also based on GCN, is a multitask learning framework that simultaneously optimizes node prediction and link prediction tasks. The GCN and GAT algorithms apply the integrated multi-omics data to GCN and GAT models, respectively, as in our study. Using the experimental data from our study, we applied the five algorithms to the CPDB network, and for the other four algorithms, we followed the default parameter settings of their original algorithms. For the results obtained with different algorithms, we plotted ROC curves (Figure 3A) and PR curves (Figure 3B) to compare their AUROC and AUPR values. Additionally, bar charts were created to visualize the F1 Score (Figure 3C) and MCC (Figure 3D) of the different methods.

Observing Figure 3, we find that MONet outperforms the other four baseline methods on the CPDB network. The AUROC of MONet reaches 0.8864, which is 0.0243 higher than the relatively effective MTGCN algorithm and 0.0315 higher than the least effective GCN. Comparing the area under the PR curves, the advantage of MONet becomes more prominent, reaching 0.7781, surpassing the other four algorithms. MONet outperforms other algorithms in terms of F1 Score (0.675) and MCC (0.5445), demonstrating its superior ability to balance precision and recall as well as to handle data imbalance effectively. Compared to other methods, MONet exhibits more balanced overall performance, making it a reliable and effective tool for cancer driver gene identification. Furthermore, comparing MONet, GCN, and GAT, it is evident that MONet significantly outperforms GCN and GAT. This improvement stems from the complementary nature of the new features derived from MONet’s concatenation, which effectively integrates the global graph information captured by GCN with the local neighborhood relationships emphasized by GAT. This finding also validates the effectiveness of the ensemble approach in the task of pan-cancer driver gene prediction.

### Identifying novel cancer driver genes

#### Database comparison

**FIGURE 4:**
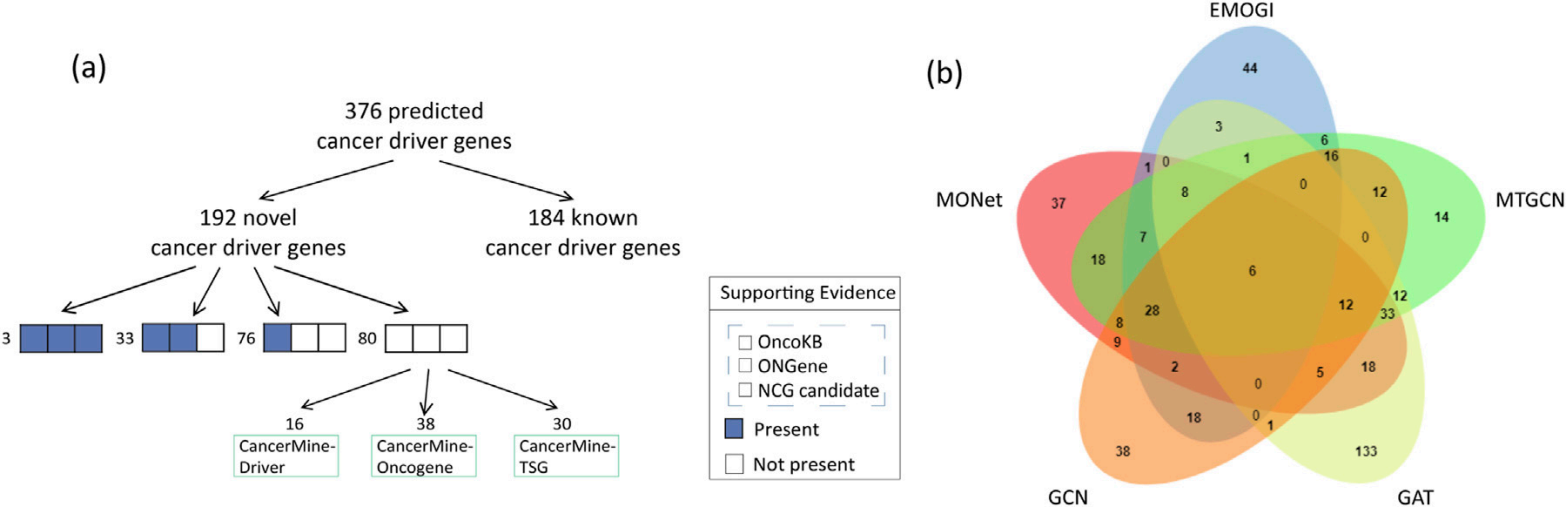
The analysis of cancer driver genes predicted by MONet. **(A)** Among the cancer driver genes predicted by MONet, 49% are already known cancer driver genes. For the newly predicted cancer driver genes, most have multiple sources of evidence supporting their potential as driver genes, including candidate cancer genes from NCG, manually curated cancer genes from OncoKB, and literature-curated cancer genes from ONGene. Specifically, 3 genes are supported by all three datasets, 33 genes are supported by two datasets, 76 genes are supported by one dataset, and 80 genes are newly identified by MONet. Based on the analysis from the CancerMine database, among these 80 newly identified genes, 61 genes are implicated in one or more aspects related to driver factors, oncogenes, or tumor suppressors. **(B)** Venn diagram showing the overlap between MONet and other methods. Thirty-seven genes were uniquely predicted by MONet.

The identification of cancer driver genes is crucial for elucidating the mechanisms of tumorigenesis and cancer progression. Here, we present the capability of MONet in identifying novel cancer driver genes. Among the 376 candidate cancer driver genes identified by integrating six PPI networks, 184 genes were validated against benchmark datasets, accounting for approximately 49%. This indicates that MONet has a high predictive accuracy and that the selection of candidate driver genes is reasonable. In other words, these 184 genes are known cancer driver genes, while the remaining 192 genes are predicted cancer driver genes identified by MONet. Next, we compared the remaining 192 newly predicted cancer driver genes with three independent cancer gene sets, specifically from NCG, OncoKB [32], and ONGene [33], ensuring no overlap with the known cancer gene sets used for training MONet. Additionally, we analyzed the newly predicted cancer driver genes using CancerMine [34], a database that employs text mining and regular updates to collect information on driver factors, oncogenes, and tumor suppressors. The comparison results are illustrated in Figure 4A.

The study results indicate that among the remaining 192 newly predicted cancer driver genes, over 58% have at least one piece of evidence suggesting their potential as cancer driver genes. Specifically, 3 genes are supported by all three datasets (NCG, OncoKB, and ONGene), 33 genes are supported by two datasets, 76 genes are supported by one dataset, and the remaining 80 genes are considered new potential cancer driver genes. Analysis based on the CancerMine database shows that among these 80 new genes, 61 are implicated in one or more aspects related to driver factors, oncogenes, or tumor suppressors.

Ultimately, only 29 genes are not included in the four selected reference sets of candidate cancer driver genes. Overall, approximately 85% (163/192) of the newly predicted cancer driver genes have at least one piece of evidence supporting their potential as cancer driver genes.

#### Comparative analysis

Similar to MONet, we applied EMOGI, MTGCN, GCN, and GAT to six PPI networks, selecting genes that ranked in the top 300 across at least two of the PPI networks for discussion. Our results revealed that MONet predicted 37 novel driver genes that did not overlap with those identified by other methods, demonstrating MONet’s unique ability to uncover new driver genes missed by other approaches (Figure 4B). Among these novel driver genes, 29 have been supported by existing literature, indicating their association with cancer progression (Supplementary File S2). Among them, APOBEC2 may be associated with nucleotide alterations in cancer-related gene transcripts, potentially promoting carcinogenesis [35]. GDNF is considered a growth factor that plays a crucial role in the nervous system, affecting cell survival and differentiation. Evidence suggests that GDNF can promote the survival and spread of already occurring cancer cells in specific environments, such as the leptomeninges [36]. Furthermore, PRELP is linked to the onset, progression, and metastasis of colorectal cancer, suggesting it may act as a promoter in cancer progression and could be a potential therapeutic target or prognostic marker [37].

#### KEGG and GO enrichment analysis

**FIGURE 5:**
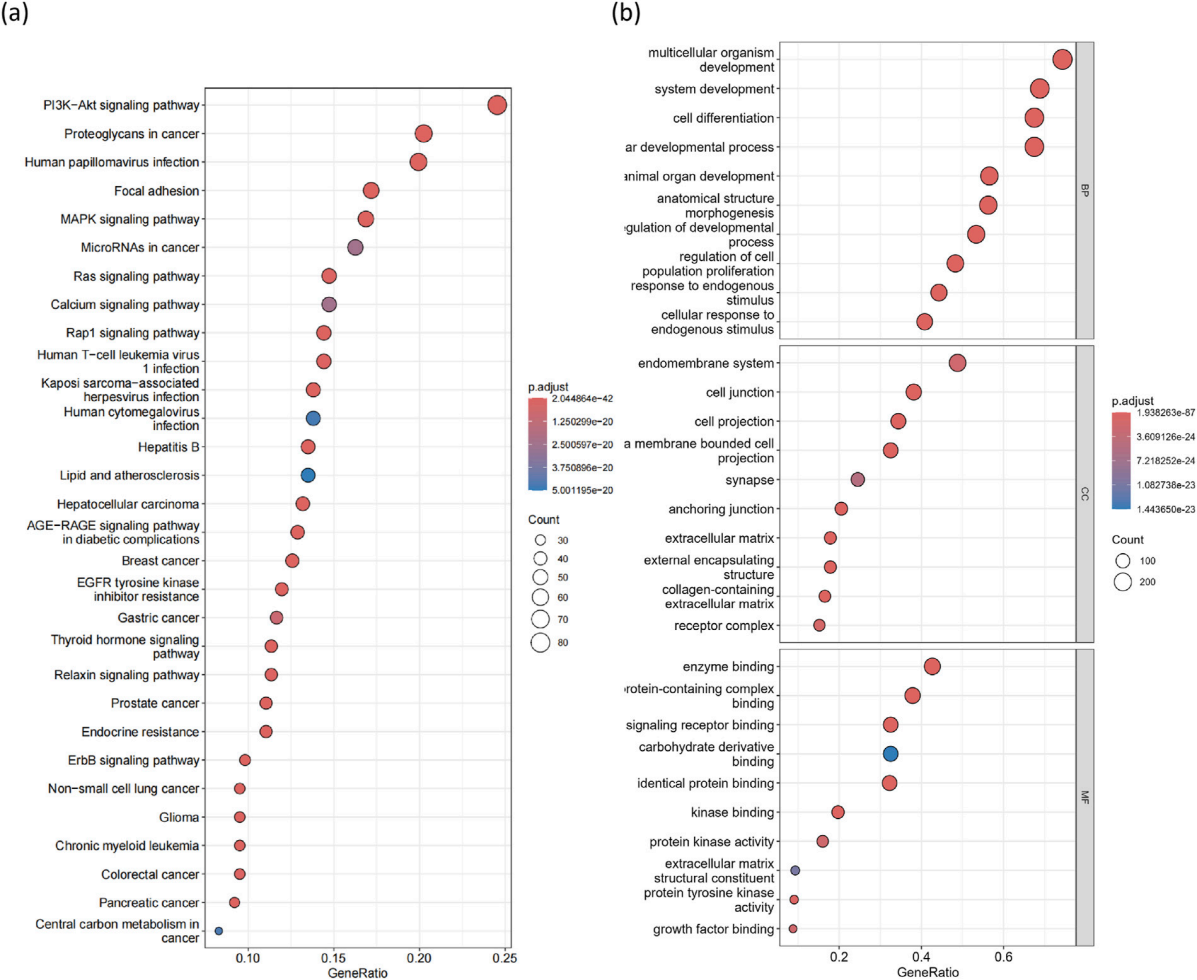
GO and KEGG enrichment analysis of 376 candidate driver genes. **(A)** Top 30 significantly enriched KEGG pathways. The horizontal axis represents the ratio of genes in each enriched KEGG pathway, and the vertical axis represents significantly enriched pathways. **(B)** Top 10 significantly enriched GO terms in biological processes (BP), cellular components (CC), and molecular functions (MF).

Using the R package clusterProfiler (v4.10.0) [38], we found that 181 KEGG pathways were significantly enriched (p < 0.05, q < 0.05) among the cancer driver genes identified by MONet (Supplementary File S3). The top 30 most significant pathways are considered known or potentially related to cancer (Figure 5A). For instance, pathways such as proteoglycans in cancer (p.adjust = 2.04 × 10^−42^), human papillomavirus infection (p.adjust = 3.66 × 10^−28^), prostate cancer (p.adjust = 1.86 × 10^−25^), breast cancer (p.adjust = 9.30 × 10^−24^), and microRNAs in cancer (p.adjust = 2.49 × 10^−20^) are well-known cancer pathways. Additionally, the PI3K-Akt signaling pathway (p.adjust = 3.89 × 10^−39^) plays a crucial role in regulating cell growth, survival, and metastasis, making it an attractive therapeutic target in cancer due to the frequent deregulation of PI3K pathway signaling [39]. The MAPK signaling pathway (p.adjust = 1.88 × 10^−22^) is significant in regulating cancer resistance and suggests that targeting this pathway could be a potential therapeutic strategy for cancer treatment [40].

Next, we mapped the 376 candidate driver genes identified by MONet to GO terms (Supplementary File S4), including biological processes (BP), cellular components (CC), and molecular functions (MF). Our charts display the top 30 GO terms (Figure 5B). Overall, these terms are associated with processes such as cell death, cell differentiation, cell proliferation, cell activation, and immune system functions, all of which play critical roles in cancer development.

#### Analysis of 29 newly predicted candidate driver genes

For the 29 candidate driver genes newly predicted by MONet, we conducted a search on the PubMed website^1^ and found that 22 of these genes are closely related to the processes of cancer occurrence, development, and treatment. For instance, For example, LNX1 has been identified as a negative regulator of cancer stem-like cells (CSCs), playing a significant role in regulating the stemness of colorectal cancer cells [41]. Overexpression of SNW1 has been confirmed to be associated with poor prognosis in various types of cancers, with upregulation observed in a subset of prostate cancer samples [42]. COL4A4 is downregulated in lung adenocarcinoma and is associated with various tumor microenvironment (TME) parameters, immune therapy response, and drug resistance [43]. Previous reports have also indicated differential expression of COL4A4 in other tumors, correlating with prognosis, tumor stemness, immune checkpoint gene expression, and TME parameters. NID2, when demethylated or overexpressed in lung cancer cells, leads to decreased cell viability, proliferation, migration, and invasion, suggesting its role in promoting cancer development [44]. Silencing PTGER3 by siRNA in ovarian cancer cells is associated with decreased cell growth, reduced invasiveness, cell cycle arrest, and increased apoptosis, indicating PTGER3 as a potential therapeutic target for chemotherapy-resistant ovarian cancer with high levels of expression of certain oncogenic proteins [45]. Additionally, genes like ACTA1 [46], COL4A3 [47], A2M [48], ADRB2 [49], MYOC [50], among others, are closely associated with cancer biomarkers, candidate prognostic factors, and therapeutic targets.

**FIGURE 6:**
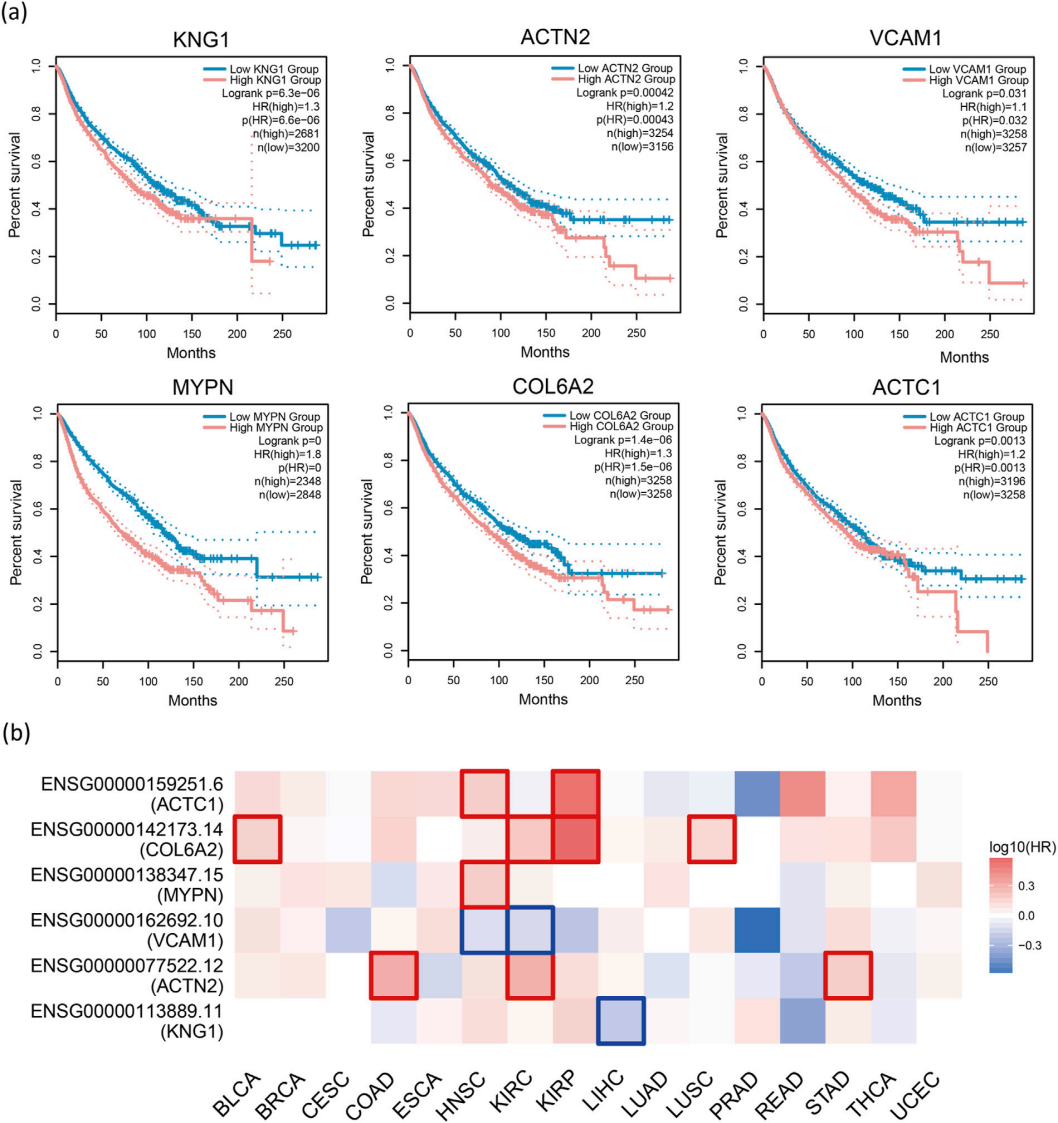
Survival analysis. **(A)** Survival analysis of 29 genes using GEPIA2 reveals that low expression of KNG1, ACTN2, VCAM1, MYDN, COL6A2, and ACTC1 is significantly associated with poor overall survival (OS), indicating that they are cancer risk factors (P < 0.05, HR > 1, group cutoff = median). **(B)** The heatmap displays the logarithmic scale (log10) of hazard ratios for different genes. Red and blue blocks represent higher and lower risks, respectively. Rectangles with borders indicate significant adverse and favorable outcomes in the prognostic analysis.

Gene Expression Profiling Interactive Analysis 2 (GEPIA2) [51] is an updated version of GEPIA used for analyzing RNA sequencing data. It includes expression data from 9,736 tumor samples and 8,575 normal samples obtained from The Cancer Genome Atlas (TCGA) and the Genotype-Tissue Expression (GTEx) project. In this study, GEPIA2 was employed to conduct survival analysis on 29 candidate cancer driver genes, with cancer types limited to 16 pan-cancer datasets. The survival analysis using GEPIA2 revealed that low expression of six genes, namely KNG1, ACTN2, VCAM1, MYDN, COL6A2, and ACTC1, was significantly associated with poor overall survival (OS) and represented cancer risk factors (P < 0.05, HR > 1, group cutoff = median, (Figure 6A).

GEPIA2 conducts survival analysis based on gene or isoform expression levels. For a given list of cancer types, it provides a heatmap displaying survival analysis results for multiple cancer types. We restrict the six genes obtained just now to individual cancers and plot a heatmap to observe the relationship between these genes and the corresponding cancers (Figure 6B). Specifically, red squares indicate higher risk. We can observe that most gene blocks in the figure are red. Additionally, in certain cancers, high gene expression is associated with shorter survival time (P < 0.05, HR > 1), as indicated by the red-bordered blocks in the heatmap. We can observe many such squares in the figure. These findings suggest that these genes are likely associated with cancer. To explore the relevance of these genes to cancer, we conducted a literature search. Previous studies have indicated that the loss of ACTN1 inhibits cancer cell proliferation, invasion, and migration, while ACTN1 itself can promote tumor growth and metastasis [52]. COL6A2 has been identified as a central gene in risk prediction models for BLCA, with qRT-PCR results showing downregulation [53]. In KIRC, high expression of COL6A2 in patients correlates with poorer survival and may be associated with adverse outcomes and distant metastases [54]. COL6A2 has also been identified as one of the genes in classifiers distinguishing LUSC from other cancer types [55]. Based on these findings, we speculate that the aforementioned genes could serve as potential biomarkers for their corresponding cancers, aiding in auxiliary diagnosis and prognosis assessment, or could become candidate targets for targeted therapy, thus contributing to the development of new personalized treatment strategies.

### Analysis of gene-drug target associations

The improvement in cancer survival rates is primarily driven by advancements in early diagnosis and novel drug treatments [56]. Therefore, identifying the molecular targets of each drug and discovering new drug targets in cancer are crucial for enhancing cancer treatment efficacy.

The Drug-Gene Interaction Database (DGIdb v5.0.6, ^2^) [57] integrates reported literature on drug-gene interactions and includes data from four sources: Gene Sources, Drug Sources, Interaction Sources, and Potentially Druggable Sources, comprising 47 databases. It provides information on the associations between genes and their known or potential drug interactions. DGIdb contains over 10,000 genes and 15,000 drugs involved in over 50,000 drug-gene interactions or belonging to one of 43 potentially druggable gene categories. Drugs targeting specific genes may be closely associated with the development and progression of cancer, and may even represent potential anticancer drugs.

**FIGURE 7:**
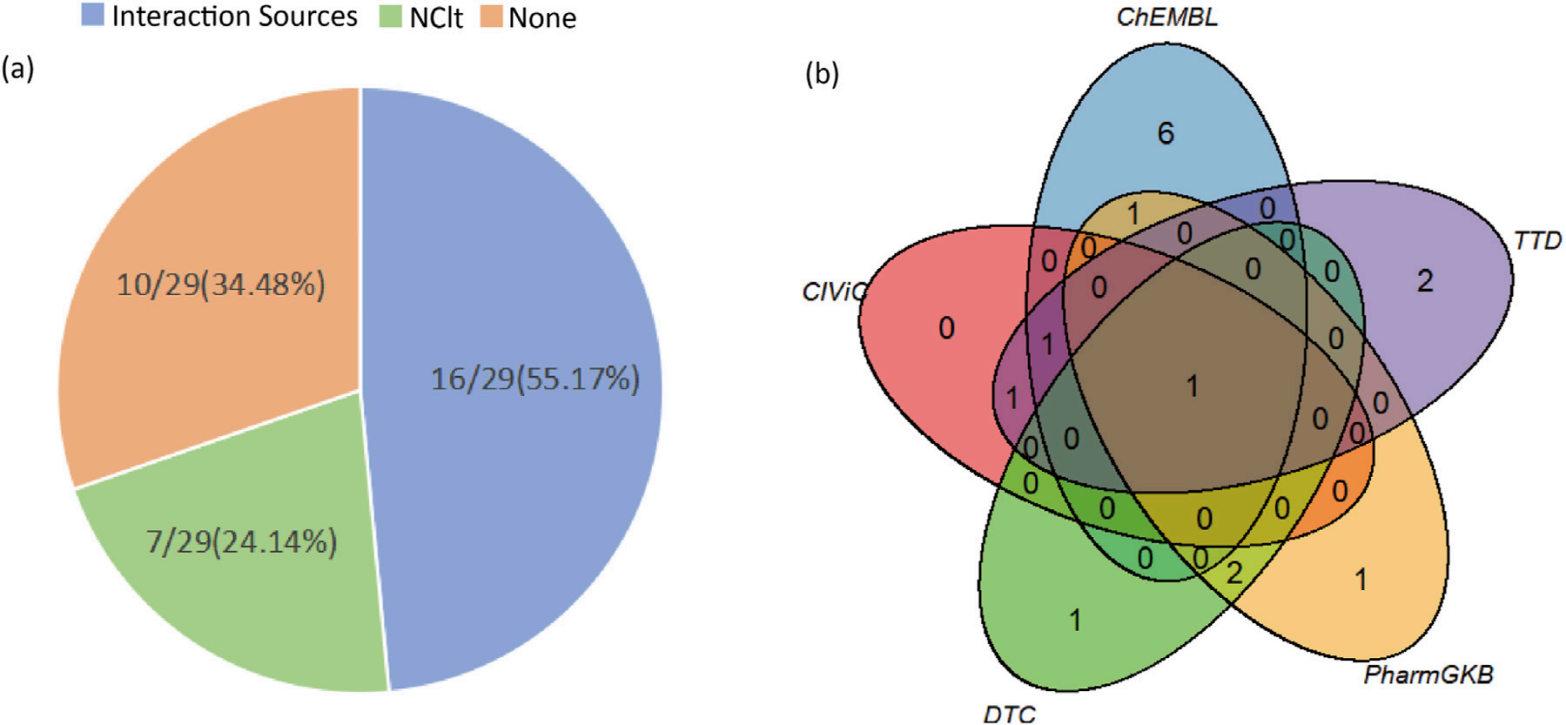
The results of the detection of candidate driver genes in the DGIdb database. **(A)** Left figure displays the number of candidate driver genes retrieved from the five databases within Interaction Sources, the NCIt database, and those not found in the DGIdb database, along with their proportions relative to the total number of candidate driver genes. **(B)** Right figure illustrates the overlap of candidate driver genes from the five databases within Interaction Sources (ChEMBL, CIViC, DTC, PharmGKB, TTD).

In this study, we examined the newly predicted candidate driver genes using the DGIdb database. The data were limited to the NCIt database in Drug Sources and five databases in Interaction Sources (ChEMBL, CIViC, DTC, PharmGKB, TTD). Among the 29 newly predicted candidate driver genes, 19 were found to be associated with drugs in the DGIdb database, with 7 found in the NCIt database and 16 in the remaining five databases (Figure 7).

According to the literature supported by the DGIdb database, there is empirical evidence indicating the relevance of newly discovered genes to the occurrence, development, and treatment of cancer. For instance, van Huis-Tanja et al. conducted a clinical correlation study and found that specific genetic markers may influence the efficacy of oral 5-fluoropyrimidine prodrug capecitabine in treating metastatic colorectal cancer. In patients receiving single-agent capecitabine therapy, the rs4702484 variant located near the ADCY2 gene and the MTRR gene may be slightly associated with a decreased progression-free survival (PFS) in homozygous wild-type patients [58]. Additionally, the transferrin receptor TfR, which is upregulated in certain cancer cells, has emerged as a potential therapeutic target. A targeted drug against TfR is Transferrin Receptor-Targeted Anti-RRM2 siRNA CALAA-01 (NCI Thesaurus Code: C78450). It is a proprietary nanoparticle formulation targeted at the transferrin receptor, containing non-chemically modified small interfering RNA (siRNA) against the M2 subunit of ribonucleotide reductase (RRM2), with potential anti-tumor activity. This drug binds to and releases anti-RRM2 siRNA via the transferrin receptor (TfR), silencing RRM2 expression, thereby inhibiting the assembly of ribonucleotide reductase (RR) and resulting in cell proliferation suppression. Furthermore, a targeted drug for ACTC1, DEXAMETHASONE, is commonly used in cancer treatment to alleviate side effects induced by cancer therapy, control cancer-related inflammation and immune responses as part of cancer treatment. However, several clinical studies have found an association between the use of dexamethasone and a decrease in overall survival rate in patients. Preclinical studies in mouse glioma models have shown a reduction in tumor-infiltrating lymphocytes after dexamethasone treatment [59].

### Multi-omics feature analysis

To further validate the reliability of utilizing multi-omics features for identifying cancer driver genes, we systematically compared driver genes selected from the CGC database with neutral genes (NGs) obtained from DORGE [60] (3,417 genes in total). We evaluated each feature across different cancer types to assess whether there are significant differences between CGC and NGs genes.

**FIGURE 8:**
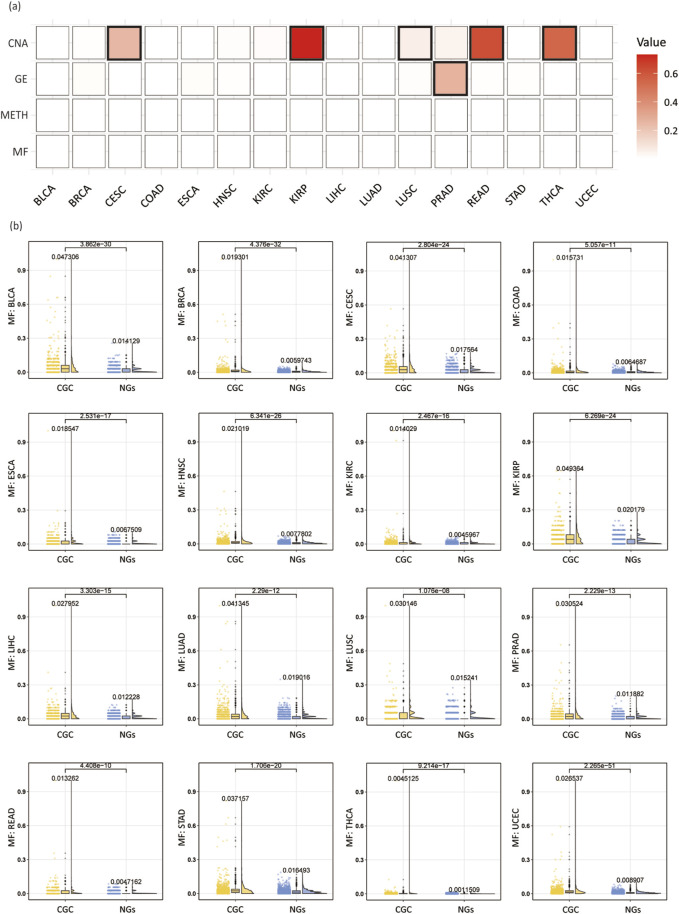
Analysis of multi-omics features in individual cancer types. **(A)** Wilcoxon rank-sum test p-values across 16 cancer types, with bold borders highlighting squares where the p-value is greater than 0.05. **(B)** Box plots, scatter plots, and half-violin plots for the MF feature across 16 cancer types. Each plot compares CGC and NGs, displaying the mean values for both groups and the p-values from the Wilcoxon rank-sum test.

Specifically, we extracted four types of features from multi-omics data and conducted a comparative analysis between CGC and NGs based on these features. To evaluate the distributional differences between the two groups of genes, we performed Wilcoxon rank-sum tests and calculated p-values for each feature to assess statistical significance. Typically, a p-value less than 0.05 is considered significant. Using the CPDB network as an example, we visualized the feature distributions between CGC and NGs through a heatmap (Figure 8A), box plots, scatter plots, and half-violin plots (Figure 8B). The analysis results for other networks are provided in the Supplementary Files S5-1, S5-2.

As shown in Figure 8, among the 64 features across 16 cancer types, the majority of features exhibit p-values below 0.05, indicating significant differences, with only six features failing to reach significance. This demonstrates that the selected four multi-omics features are effective in distinguishing CGC from NGs. Among them, the MF and METH features show significant differences across all 16 cancer types, whereas the GE feature fails to show significance in the PRAD cancer type, and the CNA feature does not achieve significance in CESC, KIRP, LUSC, READ, and THCA cancer types. These findings suggest that most features exhibit significant differences between CGC and NGs, implying that these features may hold potential biological relevance in the identification of cancer driver genes.

## Discussion

In this study, we introduced MONet, an integrated algorithm based on GCN and GAT, for the identification of cancer driver genes. MONet combines four pan-cancer omics data types (gene mutations, DNA methylation, gene expression, and copy number variations) with PPI networks to predict cancer driver genes. By integrating six PPI networks, MONet identified 376 candidate cancer driver genes. Among them, 184 were already known cancer driver genes, while most of the remaining 192 newly predicted cancer driver genes were supported by other datasets or research methods. Among the 192 newly predicted genes, we compared these genes with the driver genes identified by EMOGI, MTGCN, GAT, and GCN across the six PPI networks. Our analysis revealed that 37 genes were uniquely predicted by MONet. Notably, 29 genes, including APOBEC2, GDNF, and PRELP, have been confirmed by existing literature to be associated with cancer development and progression.

We observed that approximately 85% (163/192) of the newly predicted cancer driver genes were supported by evidence suggesting their potential as cancer driver genes.

The innovation of this study lies in the effective integration of GCN and GAT algorithms into a unified framework. The MONet model combines the complementary strengths of these two algorithms: GCN excels at capturing global graph structures, while GAT emphasizes the importance of local neighborhoods through its attention mechanism. By integrating multi-omics data with PPI networks to fully explore the potential information of multi-omics features and gene interactions, thereby improving the effectiveness of identifying cancer driver genes.

Results showed that the MONet model outperformed baseline models in terms of the area under the receiver operating characteristic (ROC) curve and the area under the precision-recall (PR) curve, demonstrating excellent performance and stability across different PPI networks. By conducting ablation experiments on the multi-omics data used by MONet, we verified that using multi-omics data can improve the prediction performance of driver genes. Additionally, we provided evidence support for newly predicted driver genes by comparing with existing driver gene databases, performing KEGG enrichment analysis and GO enrichment analysis, and consulting existing literature. For genes that could not be validated, we conducted survival analysis and drug target analysis to support their potential as cancer driver genes. Definitive evidence indicates the involvement of newly discovered genes to the occurrence, development, and treatment of cancer. We ultimately confirmed the reliability of the selected multi-omics data and can be used to explore and identify novel cancer driver genes, which provides a foundational assurance for our study.

Although this study has achieved significant results in identifying cancer driver genes, there is still room for improvement. For example, when constructing the graph structure based on PPI networks, we did not consider the issue of edge weights. Future research could incorporate edge weight information to develop more accurate cancer driver gene identification algorithms to further improve identification effectiveness. Our study demonstrates that increasing the number of PPI networks can enhance the performance of driver gene identification; however, the marginal benefits gradually diminish. Future research could focus on exploring optimal strategies for PPI network combinations to achieve a better balance between performance and resource utilization. Furthermore, as biotechnology advances, higher-quality PPI networks will further improve the reliability of driver gene identification, providing greater possibilities and opportunities for optimization in future studies.

## Data Availability

The original contributions presented in the study are included in the article/Supplementary Material, further inquiries can be directed to the corresponding author.

## Footnotes

1
https://pubmed.ncbi.nlm.nih.gov/

2
https://www.dgidb.org/
