# Supplementary figures and images for: MONet: cancer driver gene identification algorithm based on integrated analysis of multi-omics data and network models (part 2 of 4)
Source: Exp Biol Med (Maywood). 2025 Feb 4;250:10399. doi: 10.3389/ebm.2025.10399 (PMC11834253; doi:10.3389/ebm.2025.10399)

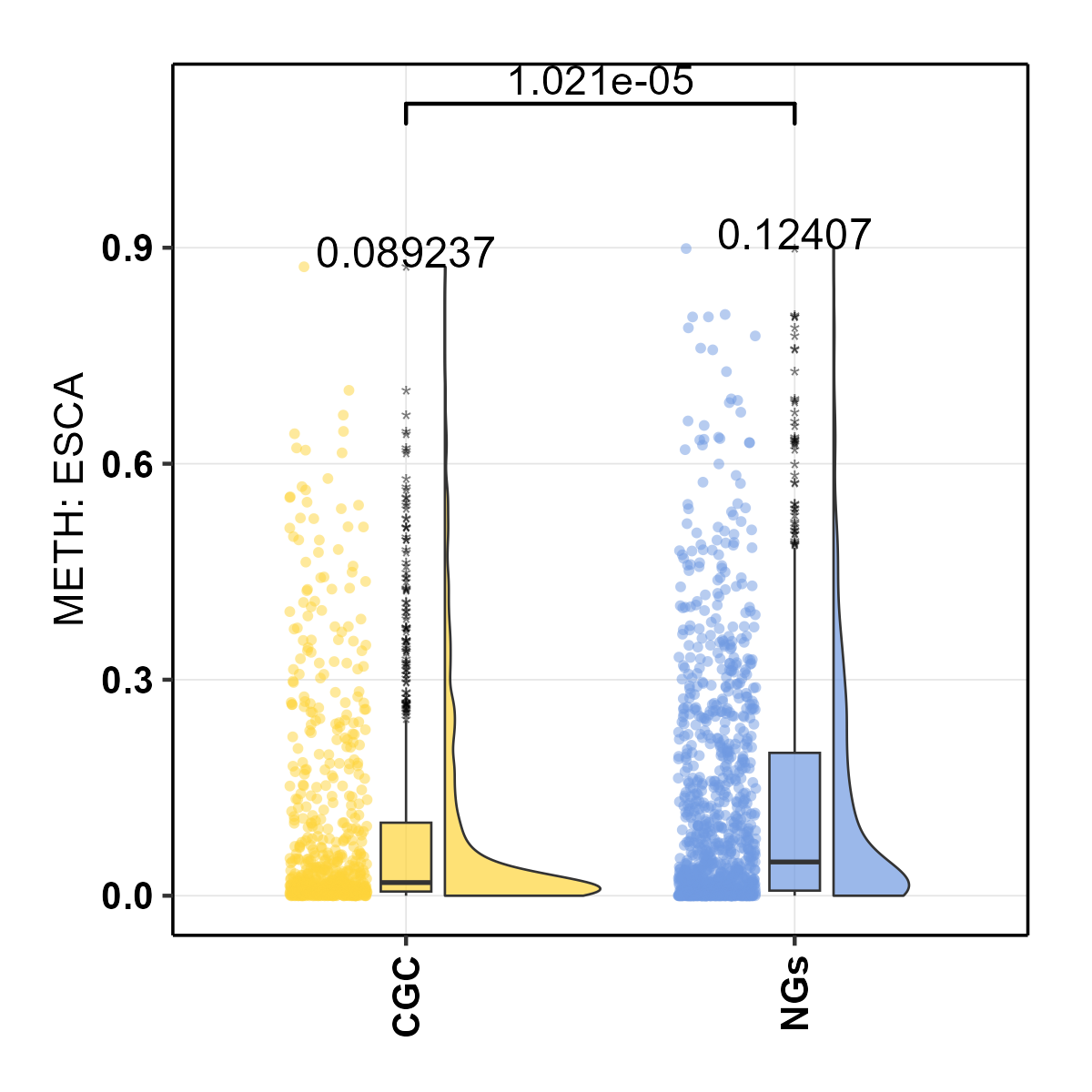

Supplement: Supplementary file 3 [file DataSheet1.ZIP › Supplementary file 5-1/IReflndex_2015/METH_ESCA.png]

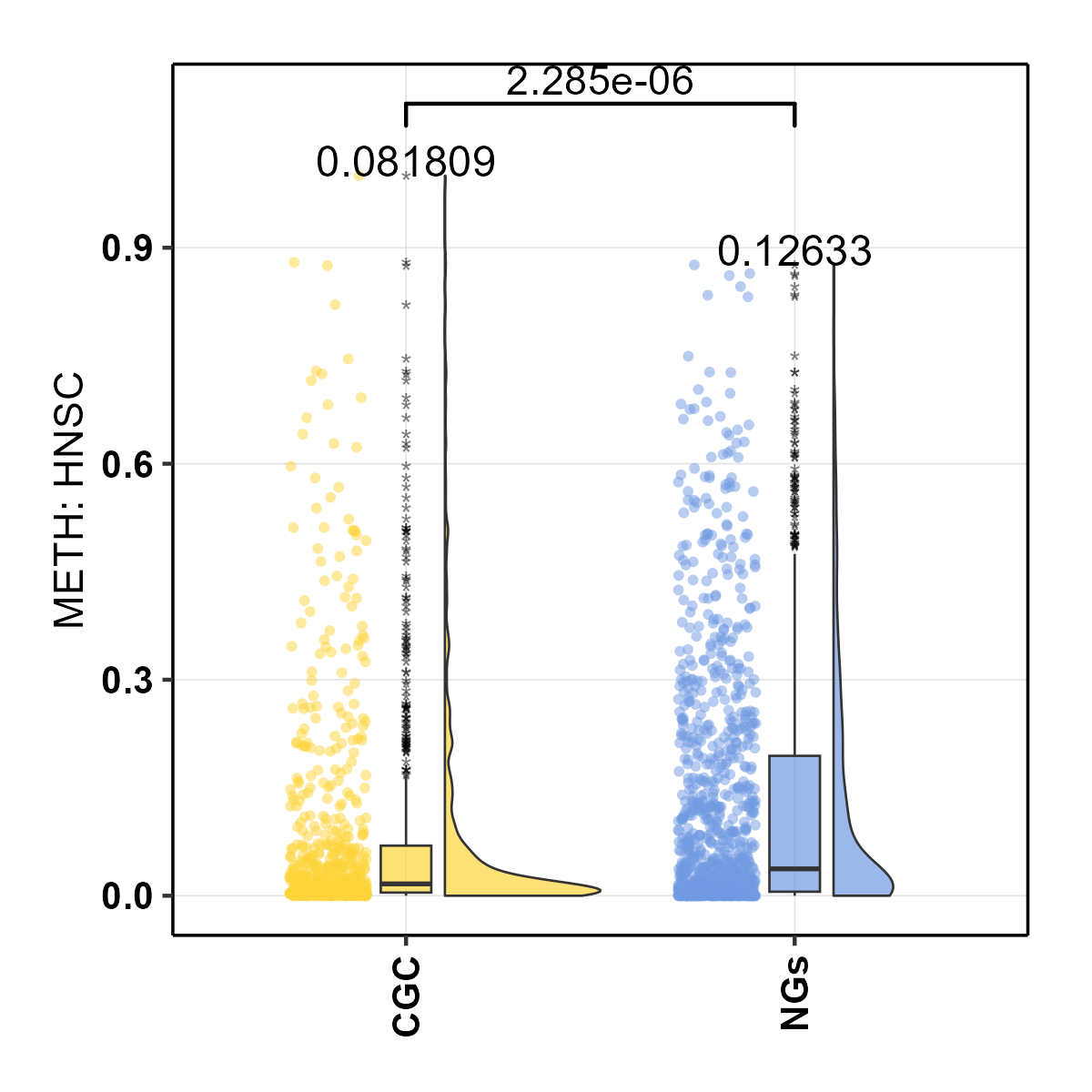

Supplement: Supplementary file 3 [file DataSheet1.ZIP › Supplementary file 5-1/IReflndex_2015/METH_HNSC.png]

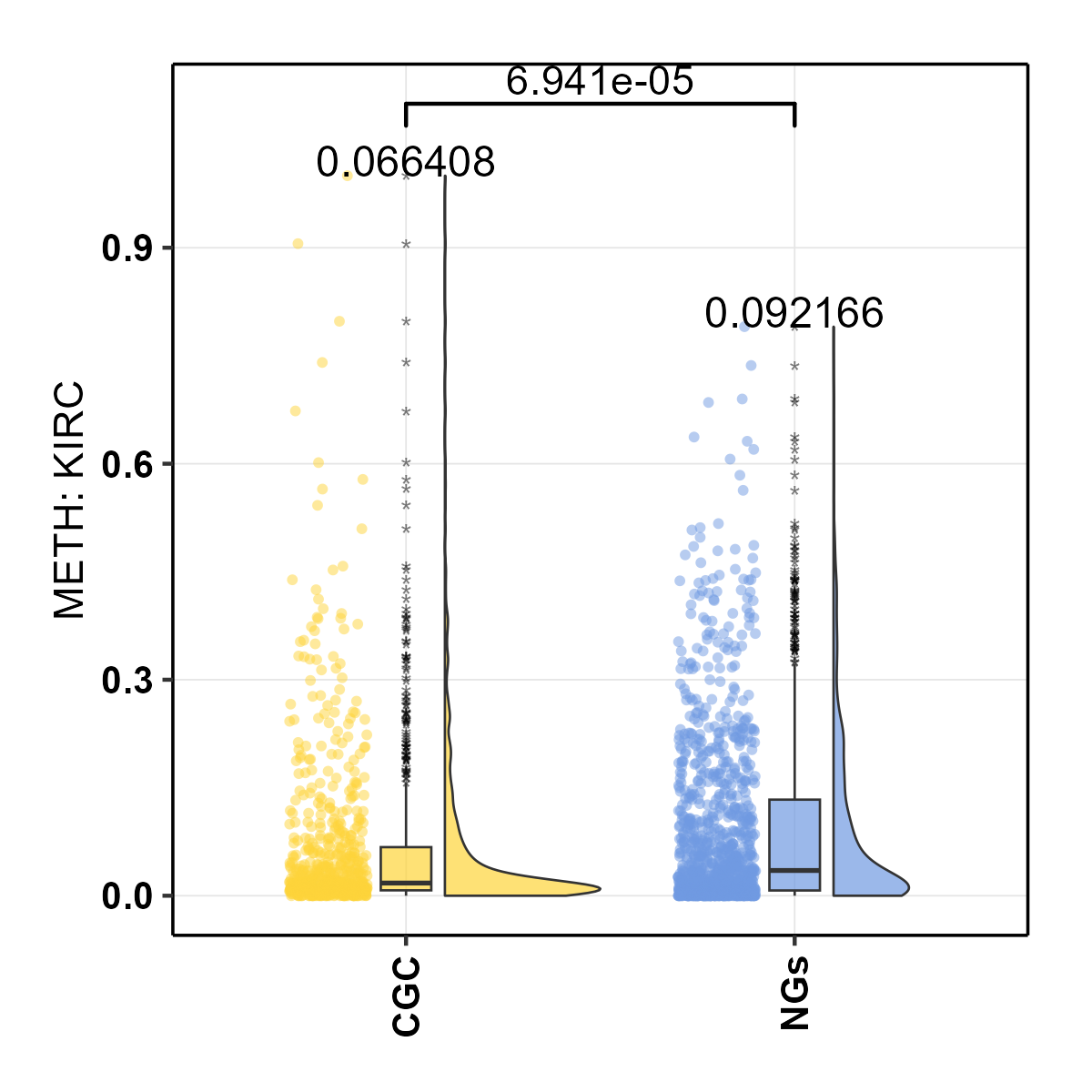

Supplement: Supplementary file 3 [file DataSheet1.ZIP › Supplementary file 5-1/IReflndex_2015/METH_KIRC.png]

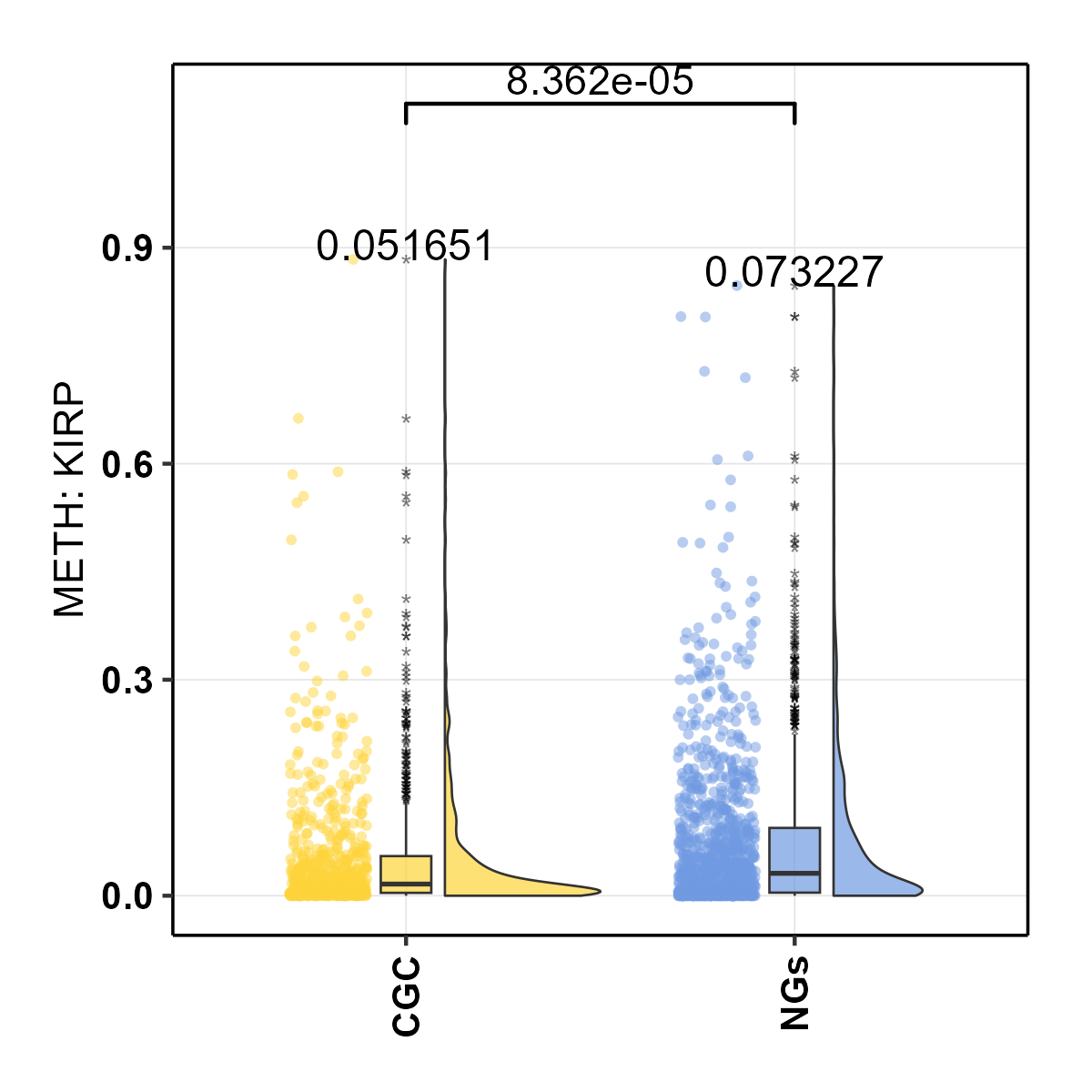

Supplement: Supplementary file 3 [file DataSheet1.ZIP › Supplementary file 5-1/IReflndex_2015/METH_KIRP.png]

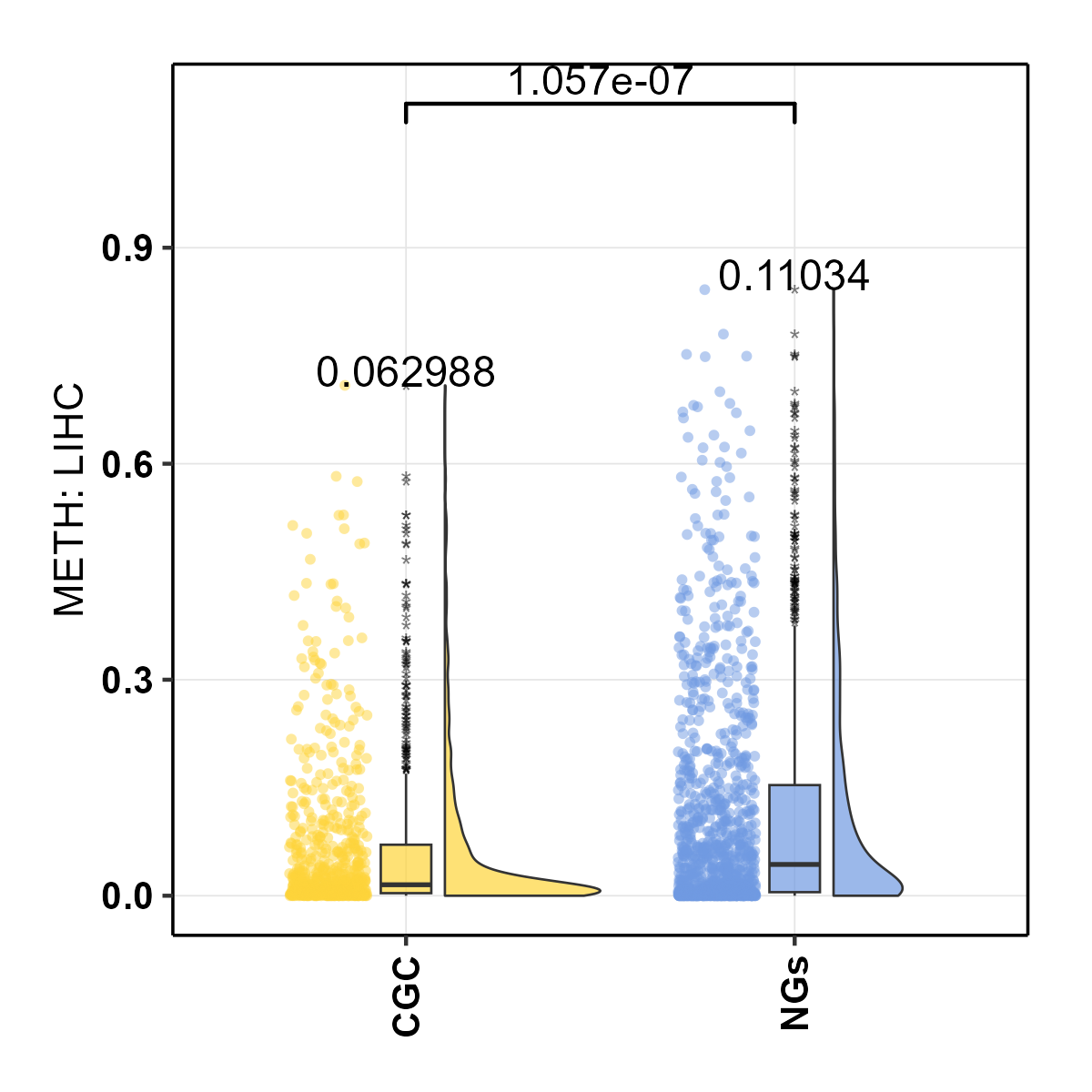

Supplement: Supplementary file 3 [file DataSheet1.ZIP › Supplementary file 5-1/IReflndex_2015/METH_LIHC.png]

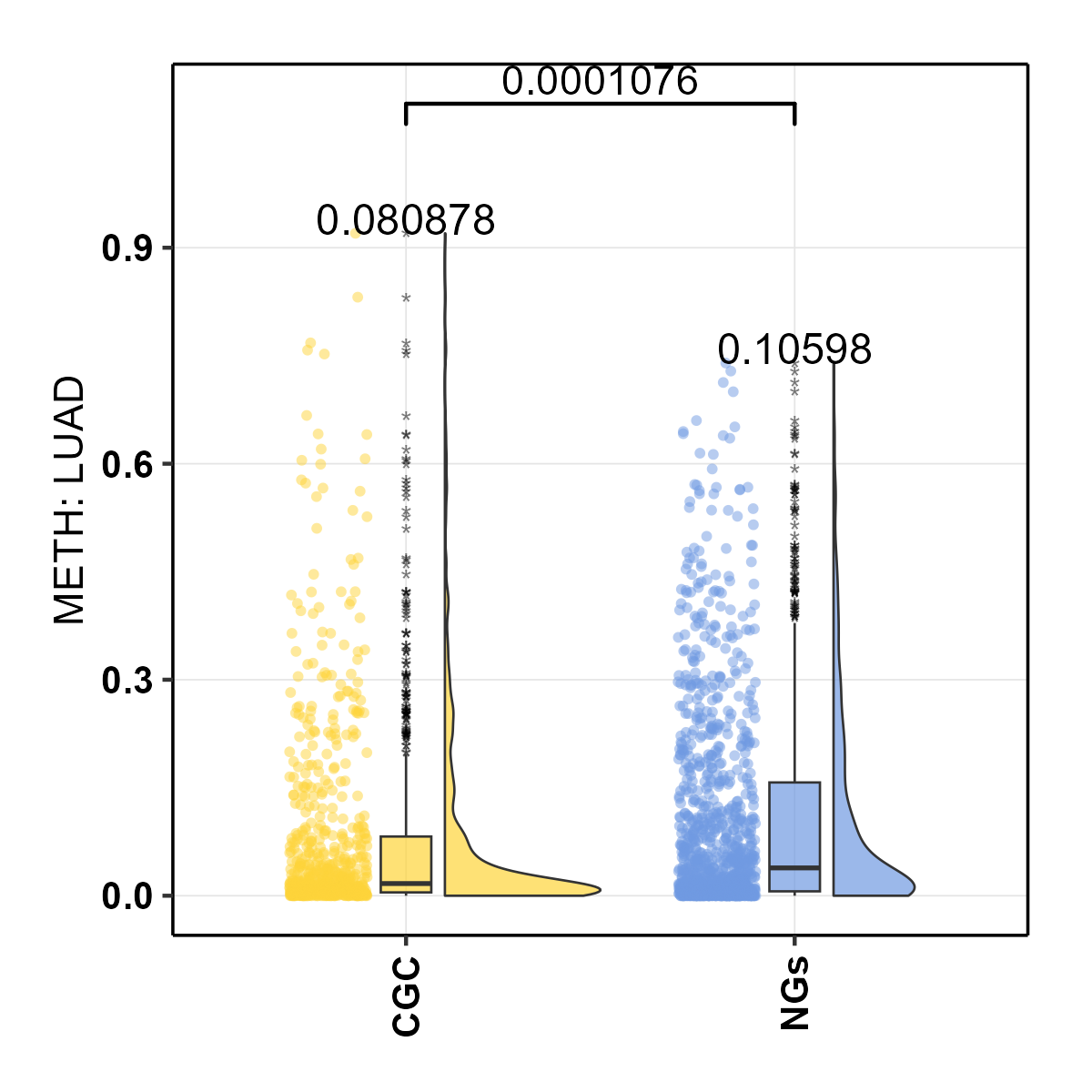

Supplement: Supplementary file 3 [file DataSheet1.ZIP › Supplementary file 5-1/IReflndex_2015/METH_LUAD.png]

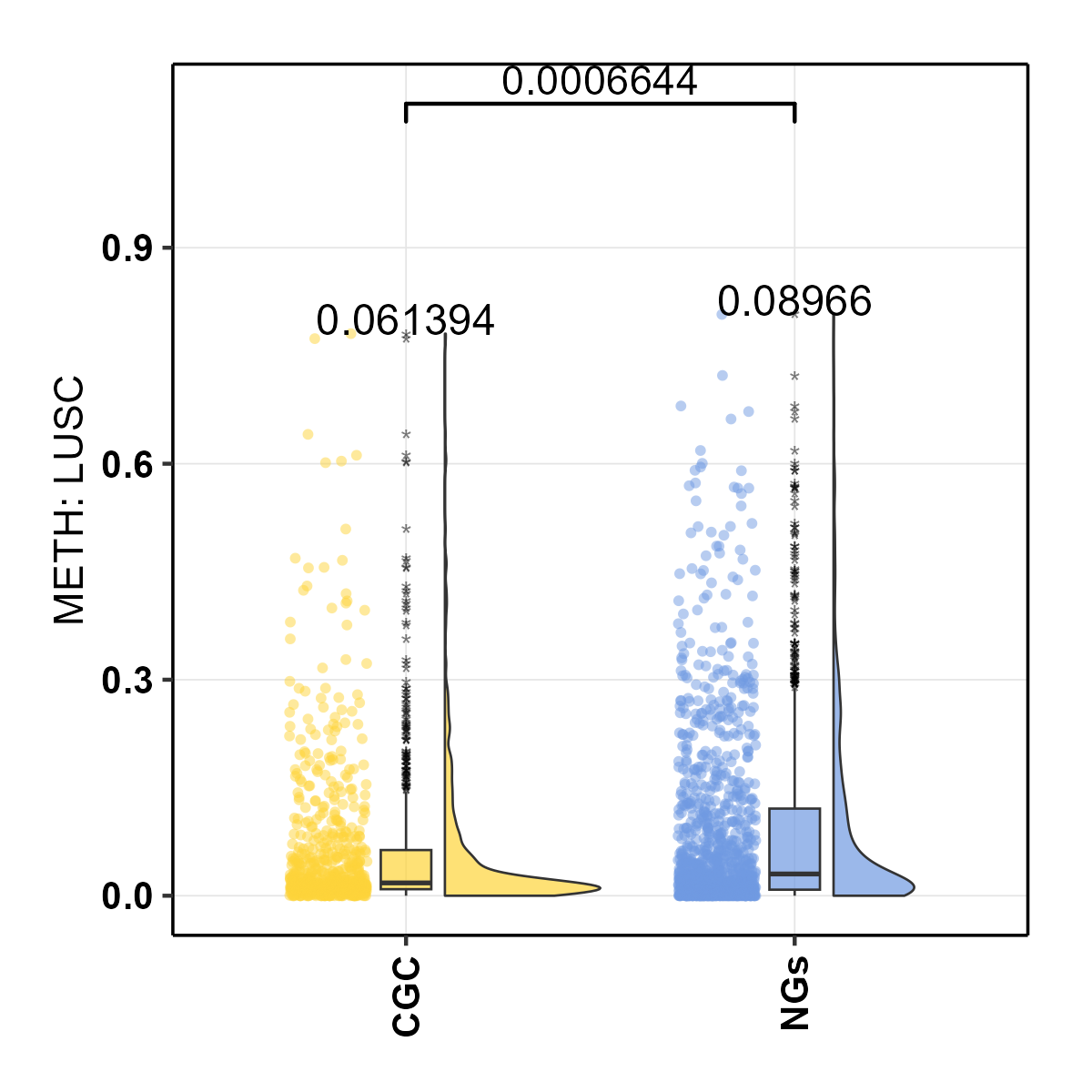

Supplement: Supplementary file 3 [file DataSheet1.ZIP › Supplementary file 5-1/IReflndex_2015/METH_LUSC.png]

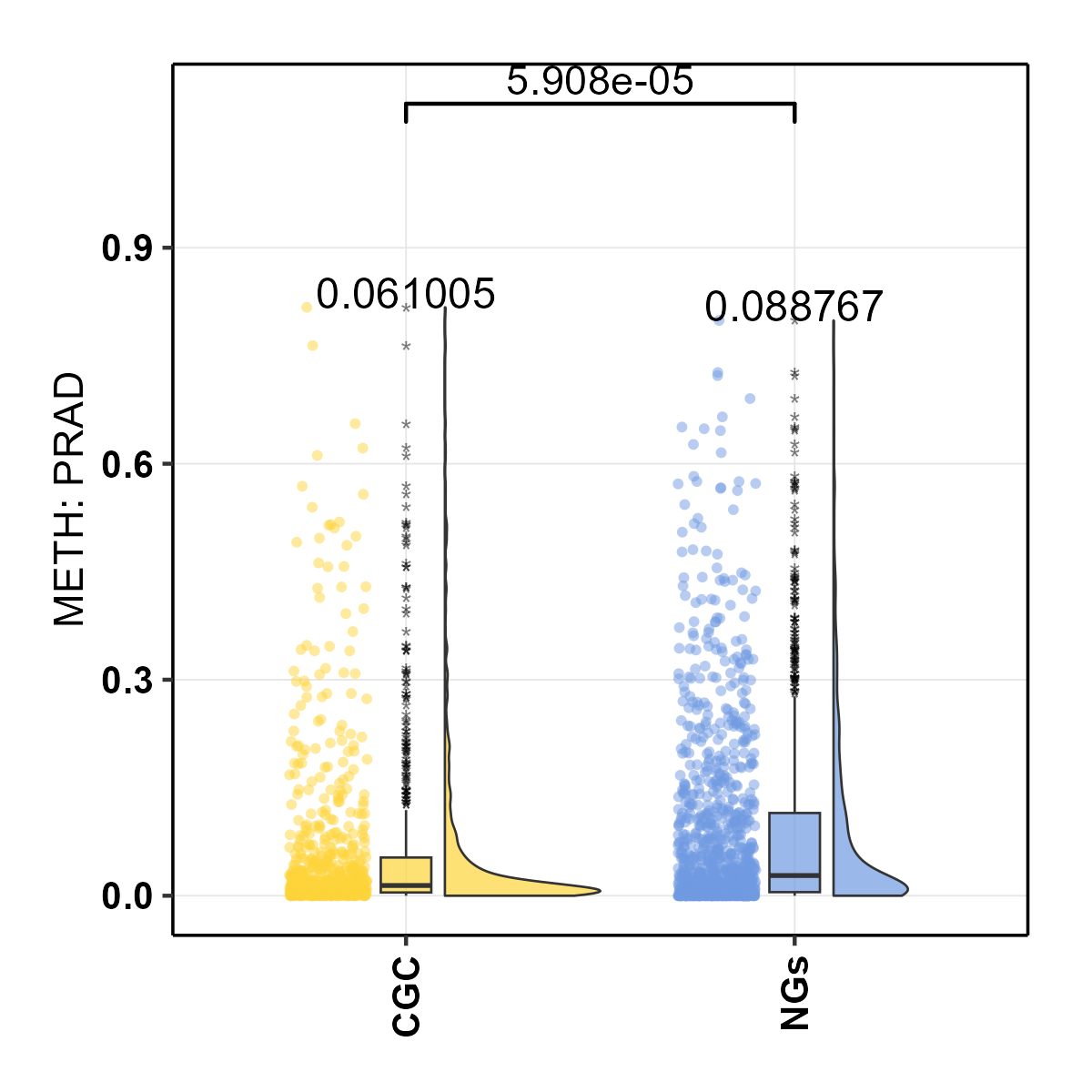

Supplement: Supplementary file 3 [file DataSheet1.ZIP › Supplementary file 5-1/IReflndex_2015/METH_PRAD.png]

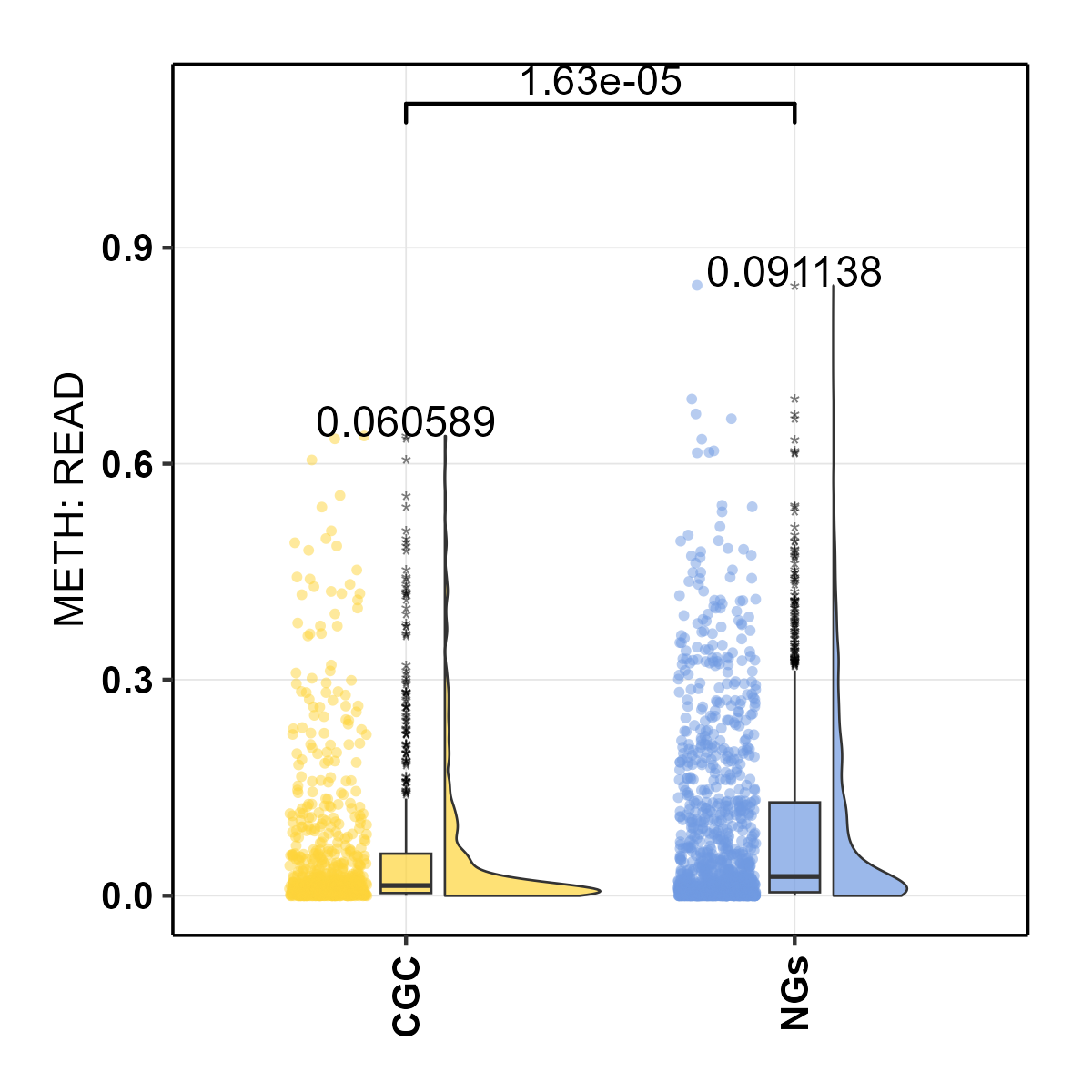

Supplement: Supplementary file 3 [file DataSheet1.ZIP › Supplementary file 5-1/IReflndex_2015/METH_READ.png]

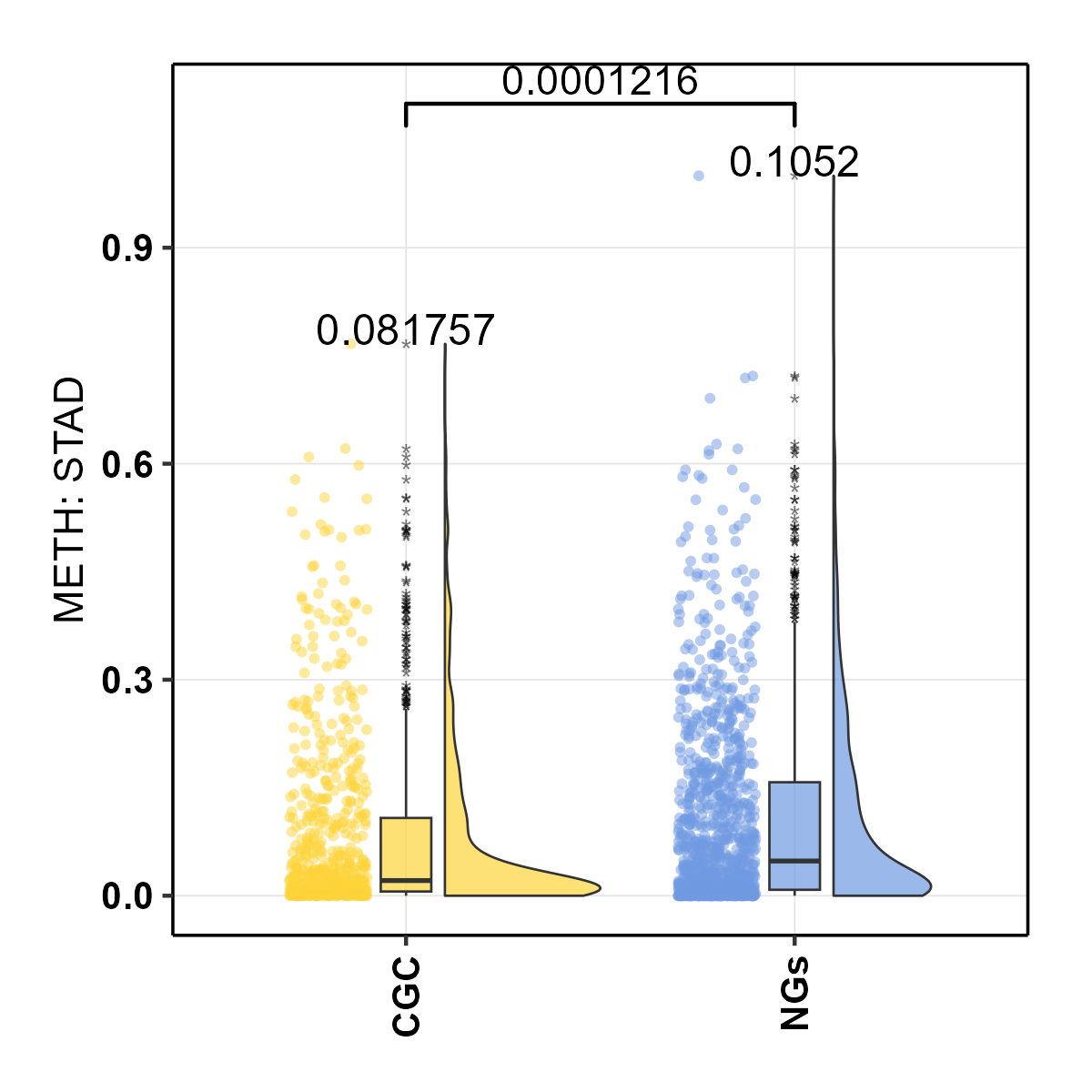

Supplement: Supplementary file 3 [file DataSheet1.ZIP › Supplementary file 5-1/IReflndex_2015/METH_STAD.png]

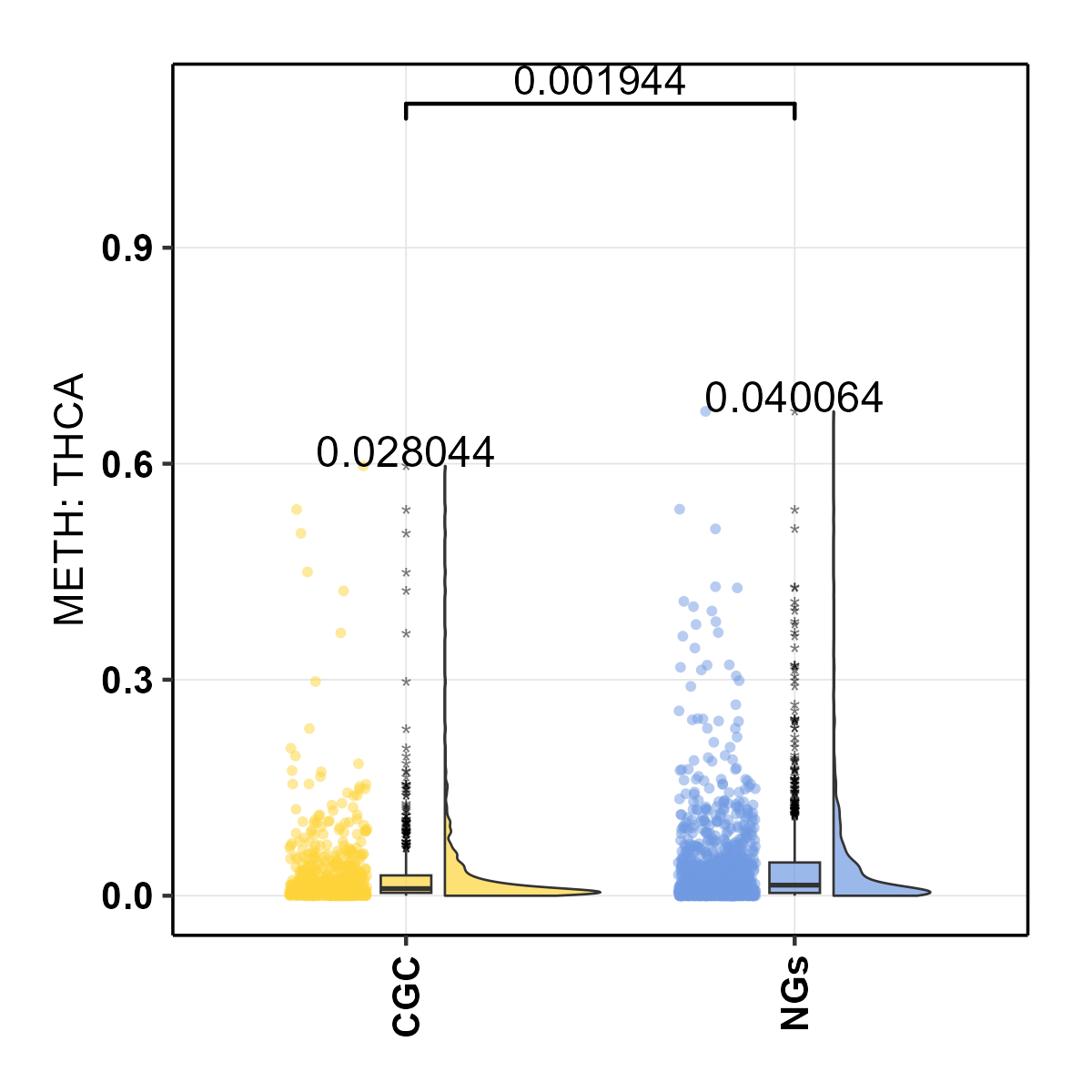

Supplement: Supplementary file 3 [file DataSheet1.ZIP › Supplementary file 5-1/IReflndex_2015/METH_THCA.png]

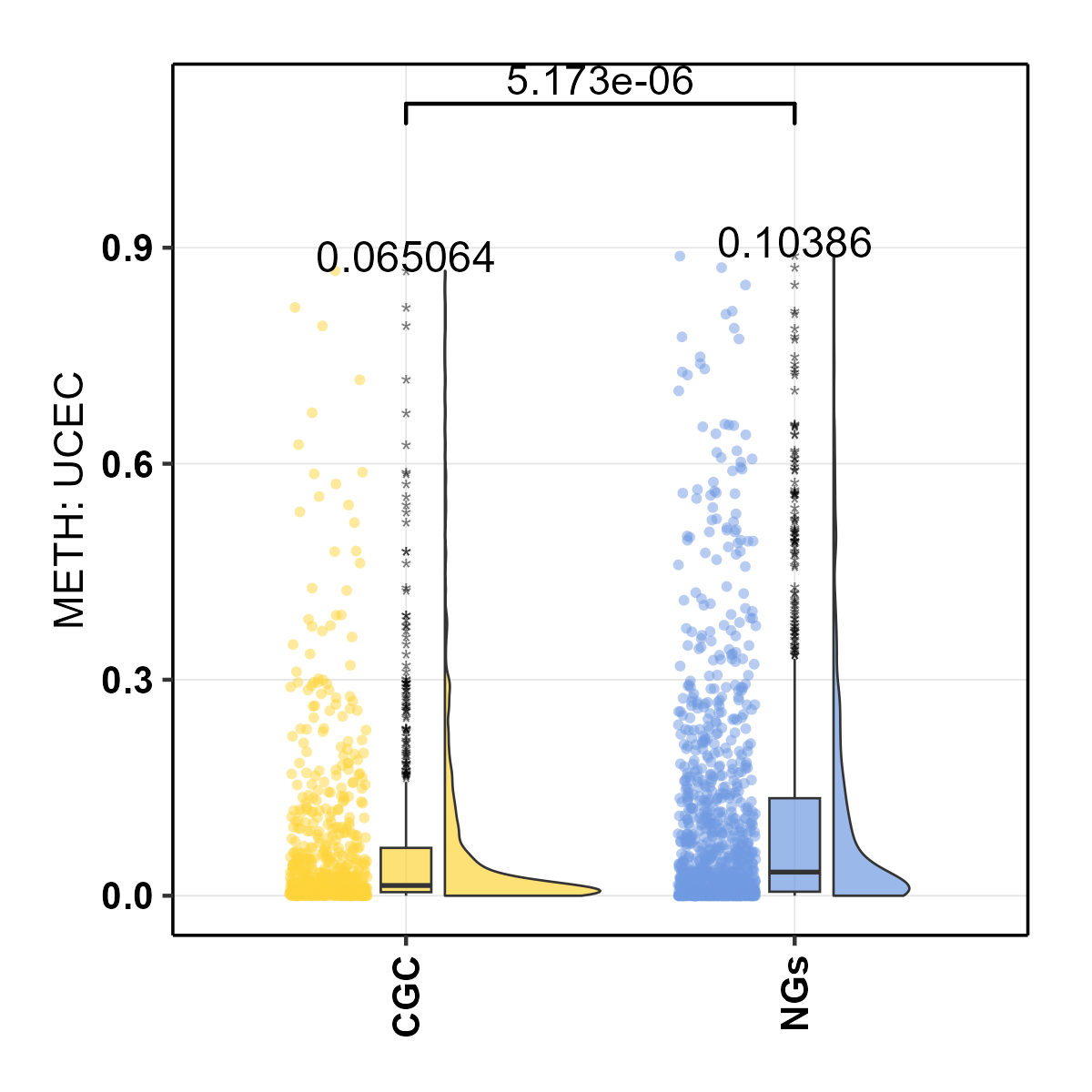

Supplement: Supplementary file 3 [file DataSheet1.ZIP › Supplementary file 5-1/IReflndex_2015/METH_UCEC.png]

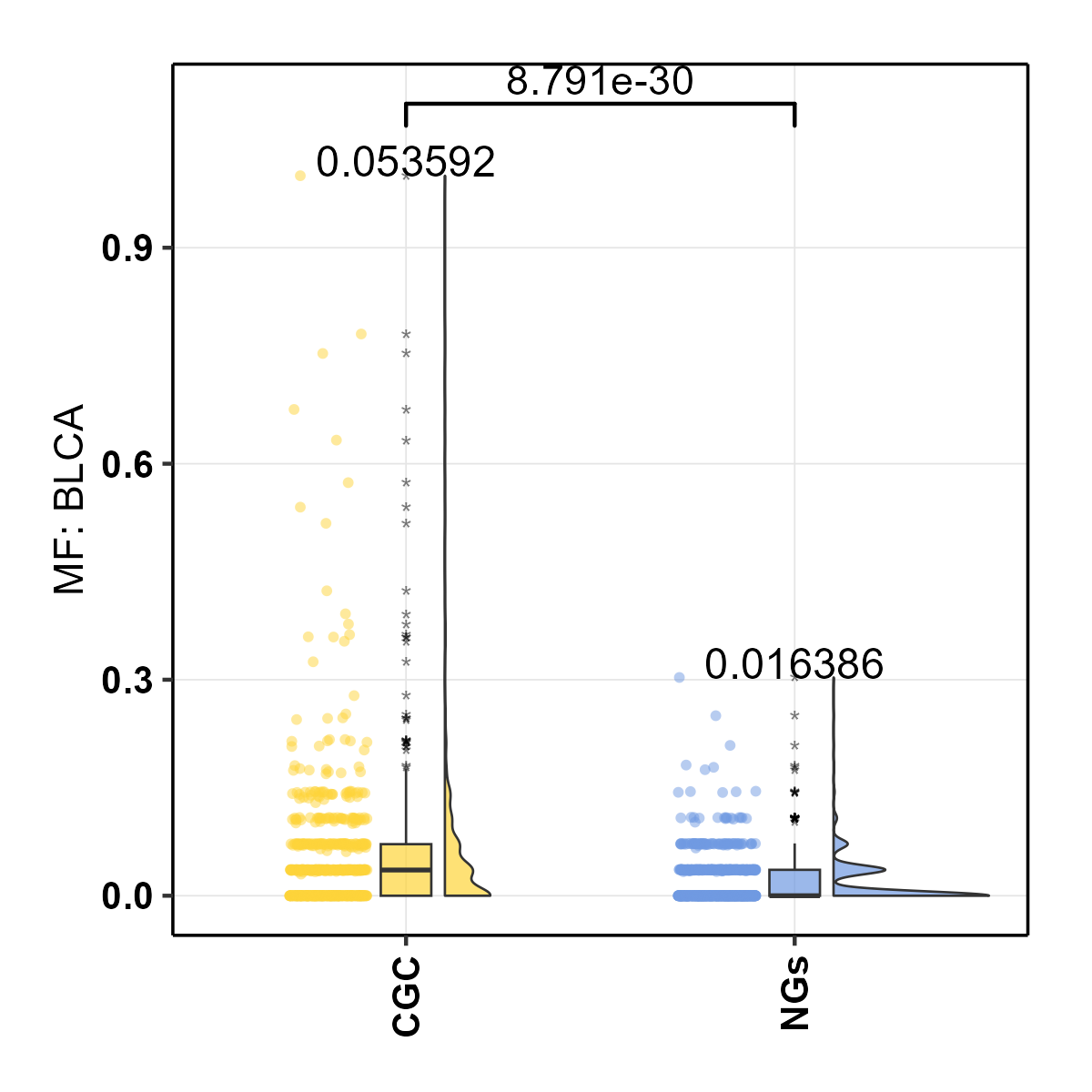

Supplement: Supplementary file 3 [file DataSheet1.ZIP › Supplementary file 5-1/IReflndex_2015/MF_BLCA.png]

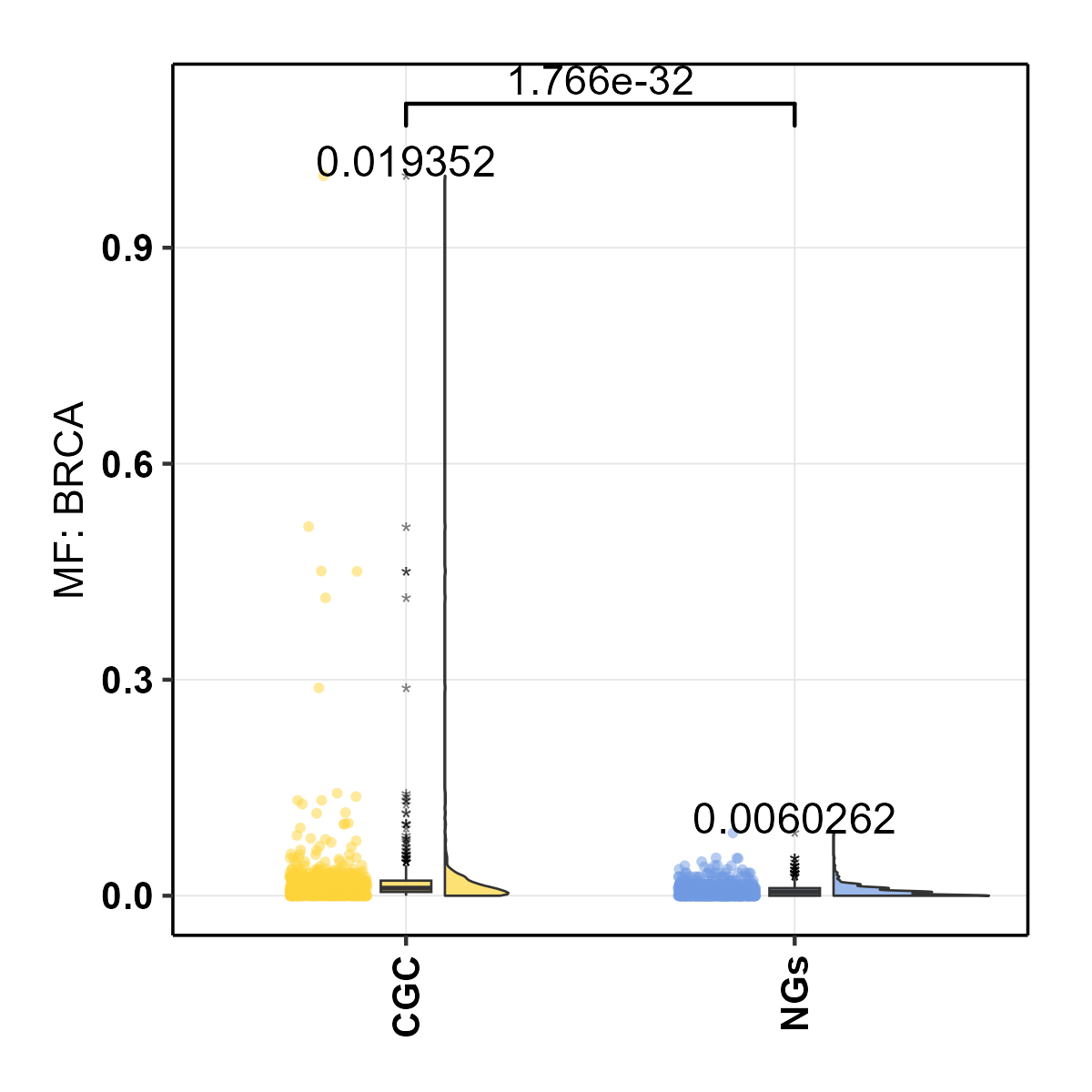

Supplement: Supplementary file 3 [file DataSheet1.ZIP › Supplementary file 5-1/IReflndex_2015/MF_BRCA.png]

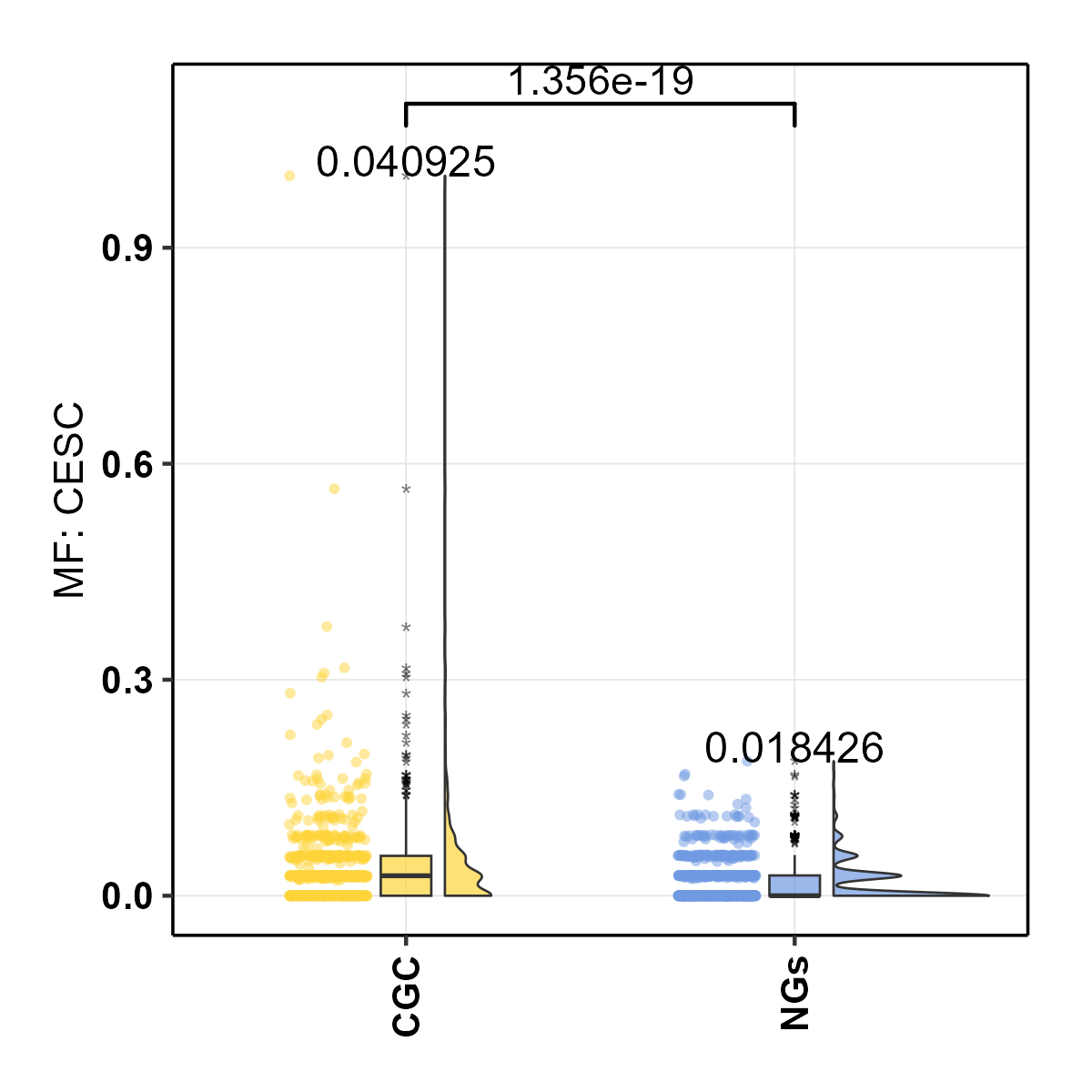

Supplement: Supplementary file 3 [file DataSheet1.ZIP › Supplementary file 5-1/IReflndex_2015/MF_CESC.png]

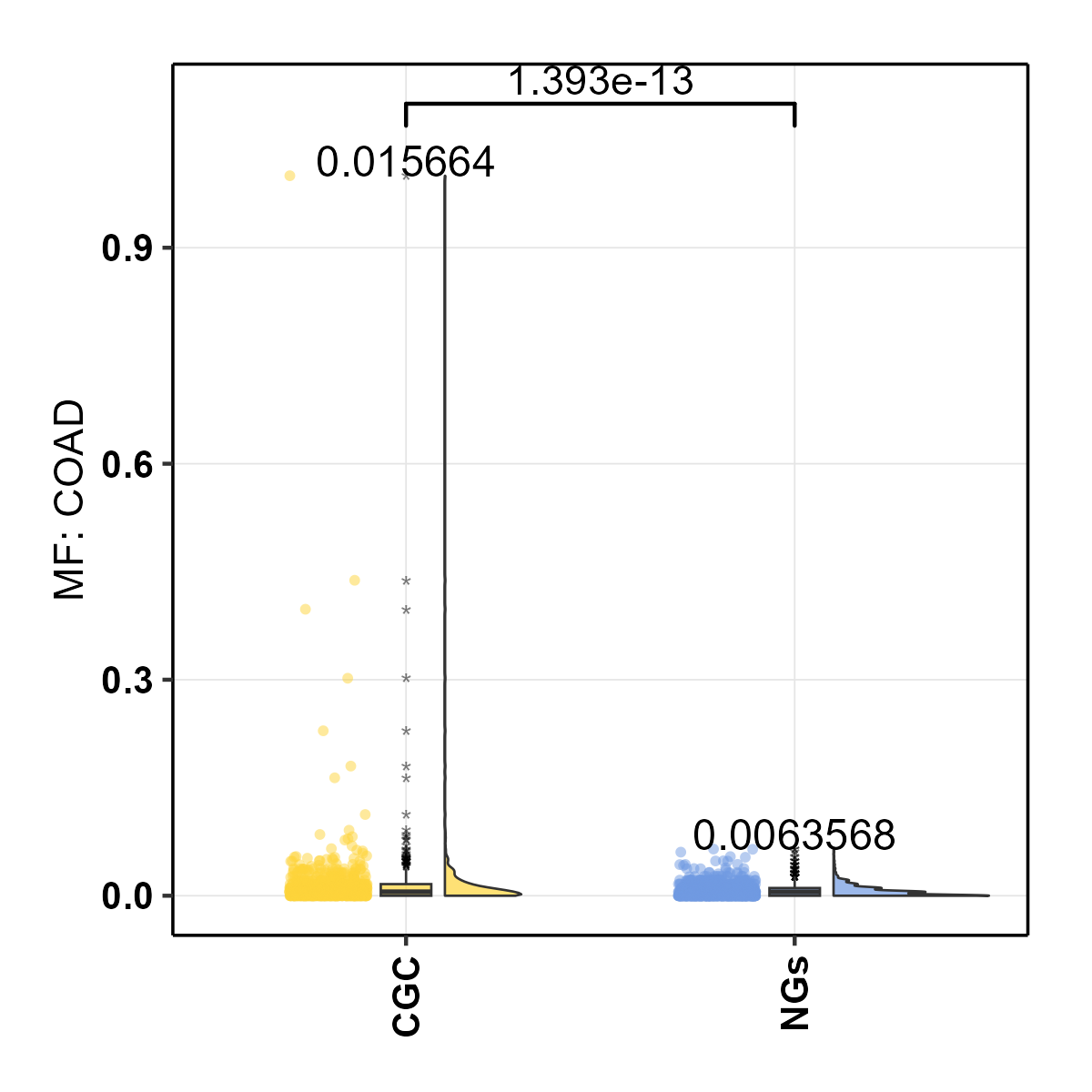

Supplement: Supplementary file 3 [file DataSheet1.ZIP › Supplementary file 5-1/IReflndex_2015/MF_COAD.png]

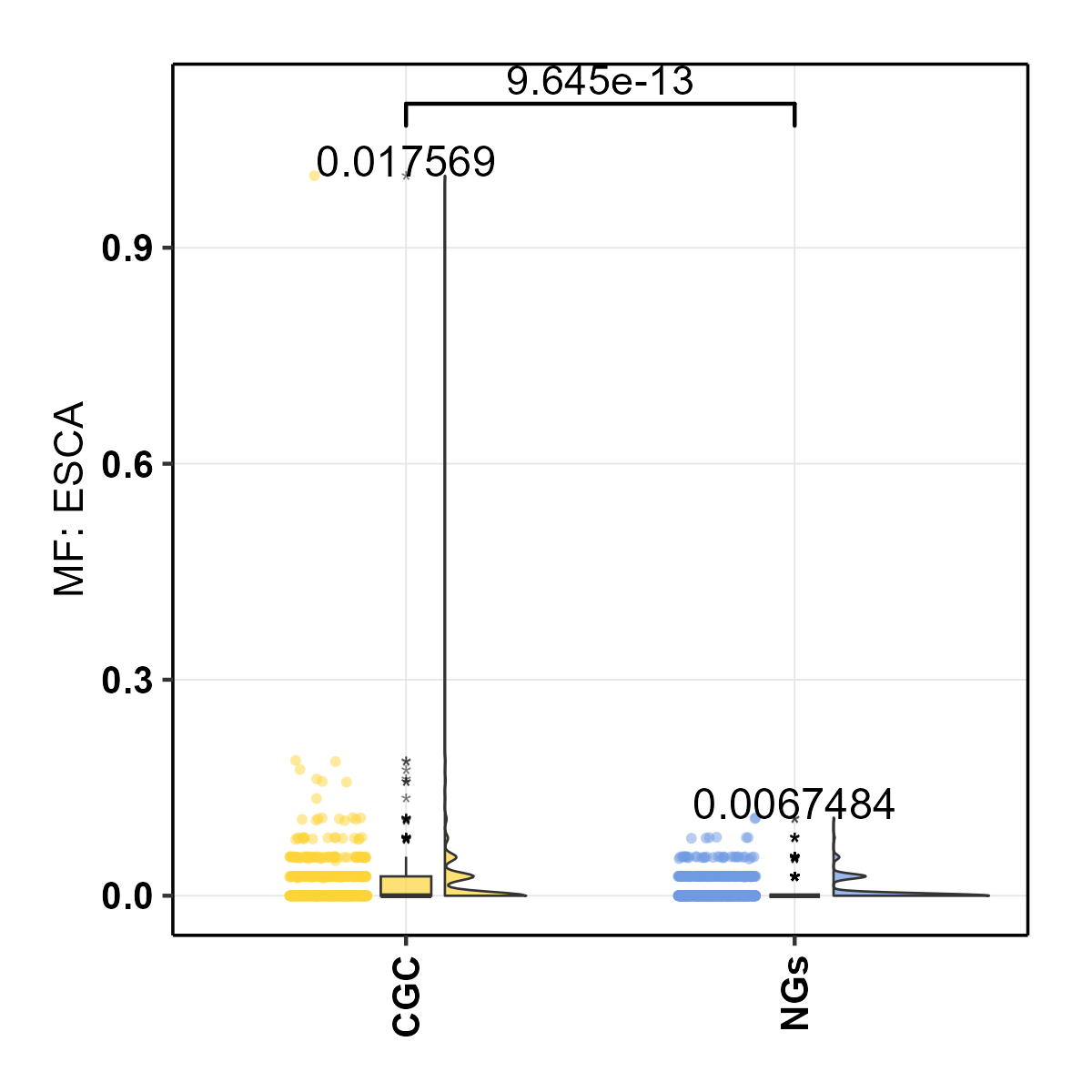

Supplement: Supplementary file 3 [file DataSheet1.ZIP › Supplementary file 5-1/IReflndex_2015/MF_ESCA.png]

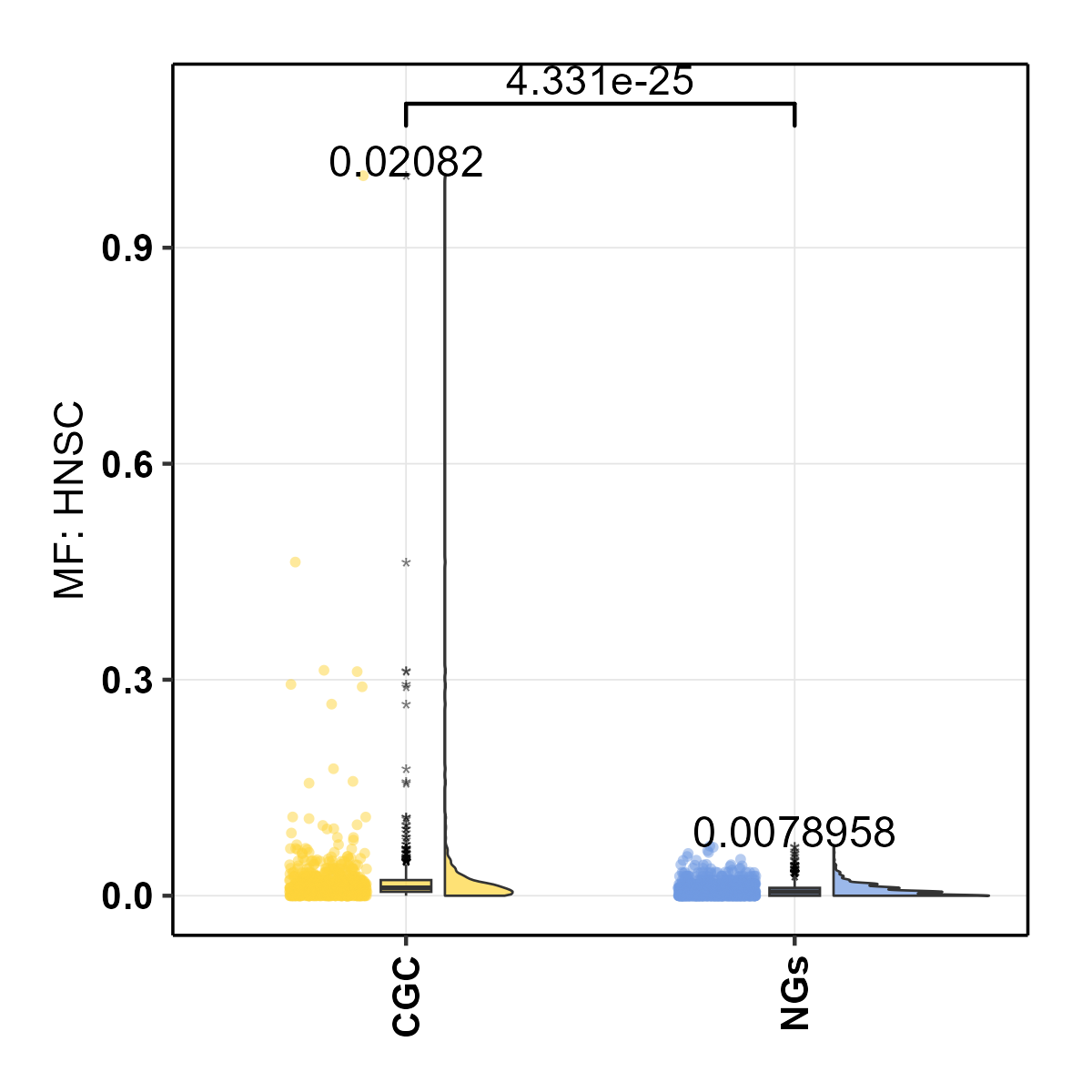

Supplement: Supplementary file 3 [file DataSheet1.ZIP › Supplementary file 5-1/IReflndex_2015/MF_HNSC.png]

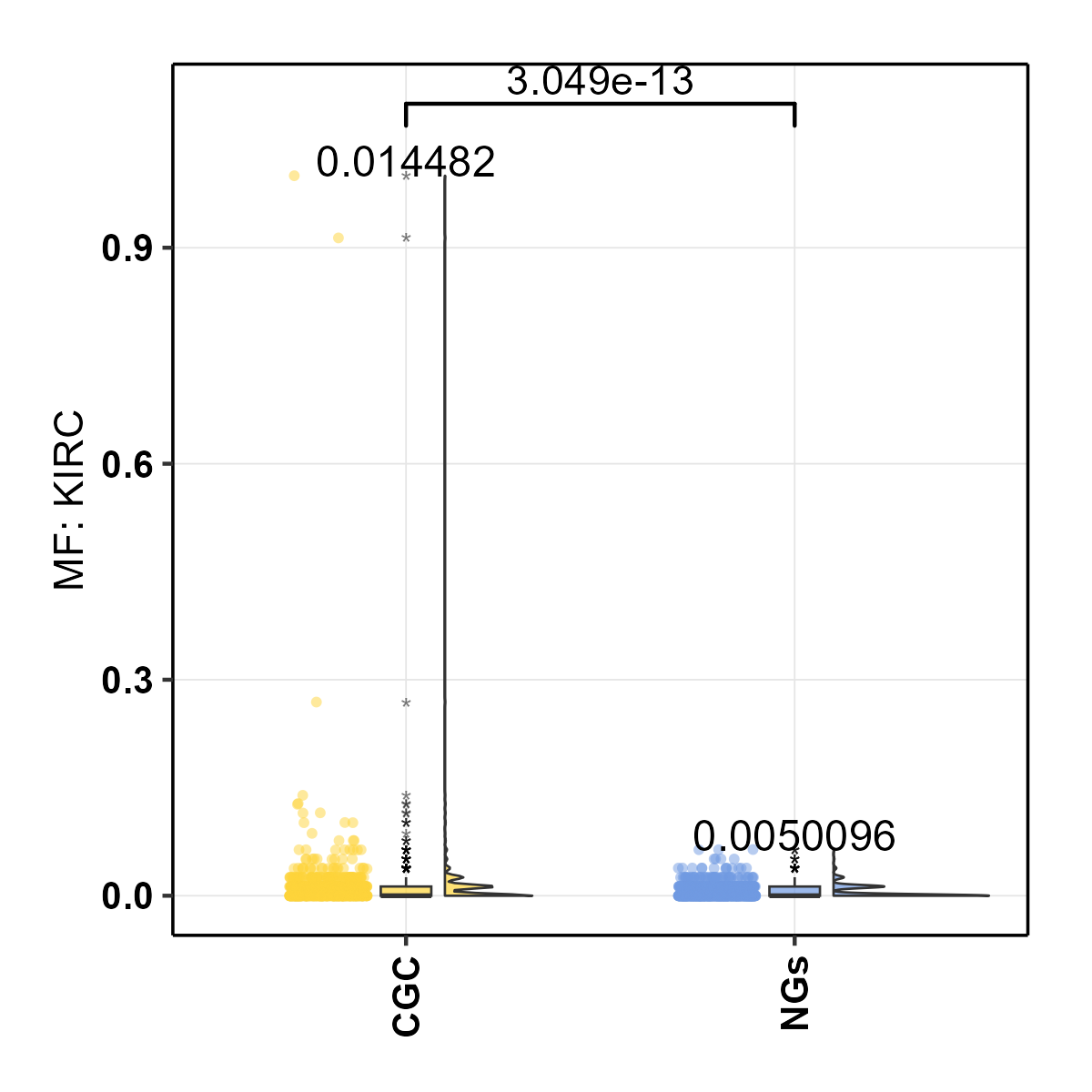

Supplement: Supplementary file 3 [file DataSheet1.ZIP › Supplementary file 5-1/IReflndex_2015/MF_KIRC.png]

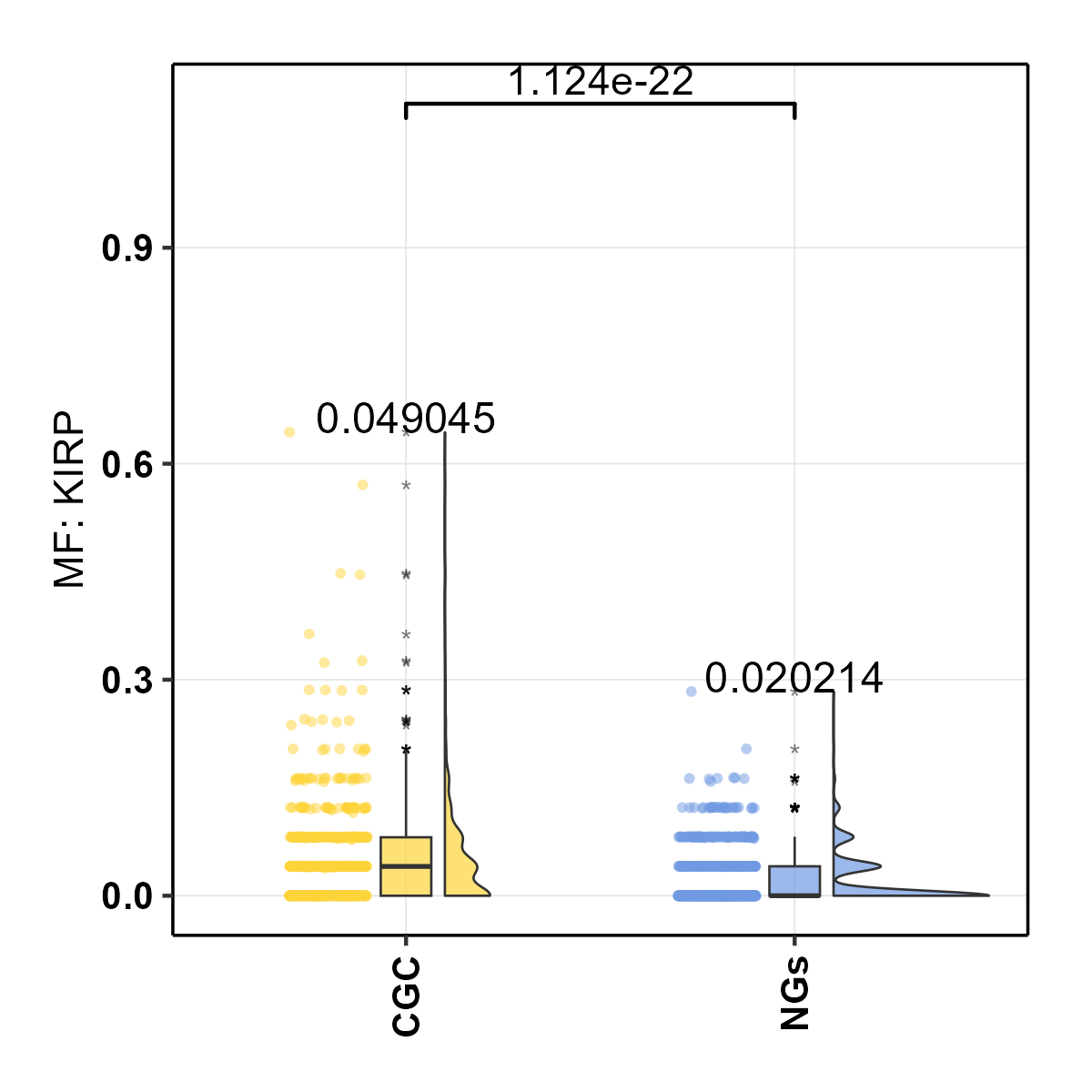

Supplement: Supplementary file 3 [file DataSheet1.ZIP › Supplementary file 5-1/IReflndex_2015/MF_KIRP.png]

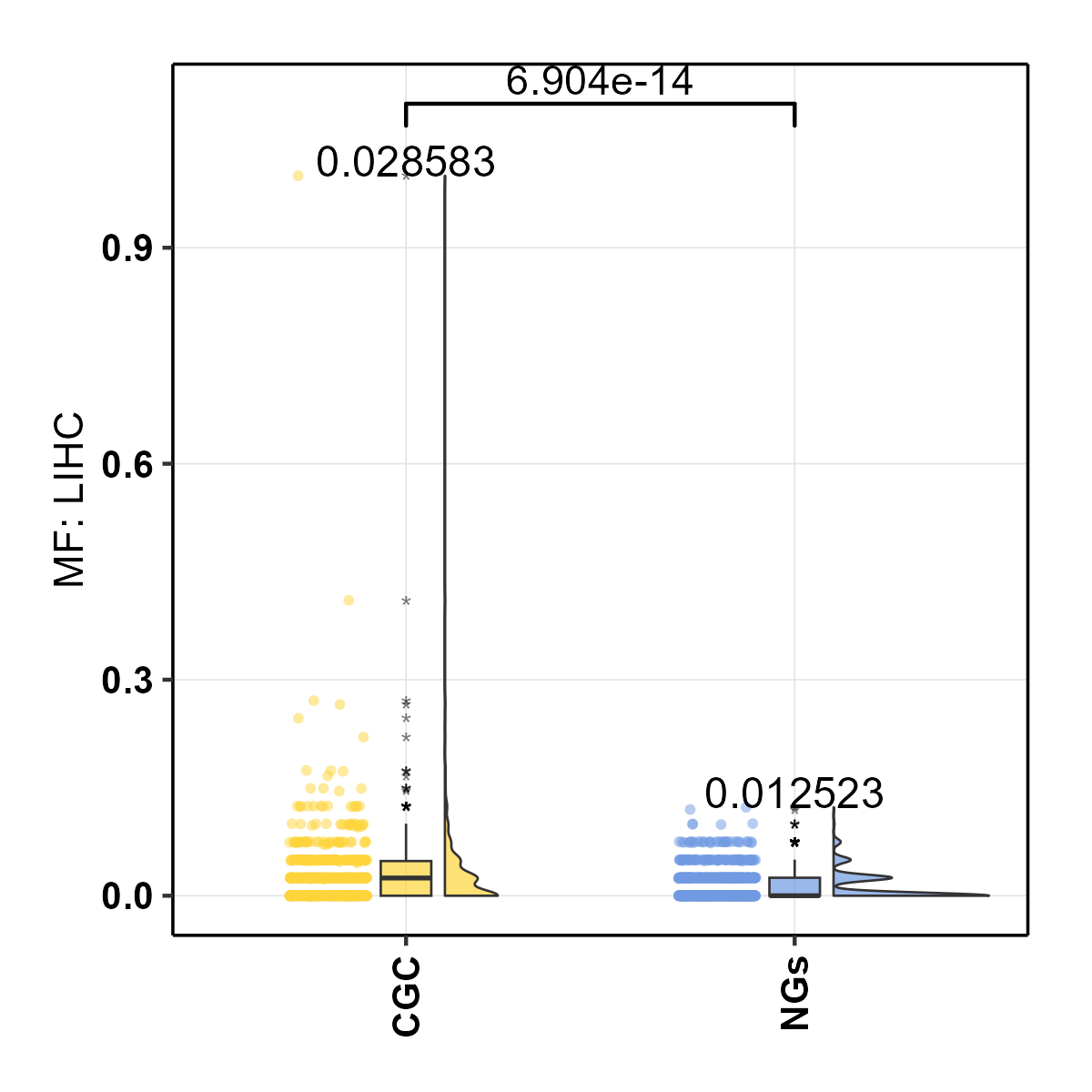

Supplement: Supplementary file 3 [file DataSheet1.ZIP › Supplementary file 5-1/IReflndex_2015/MF_LIHC.png]

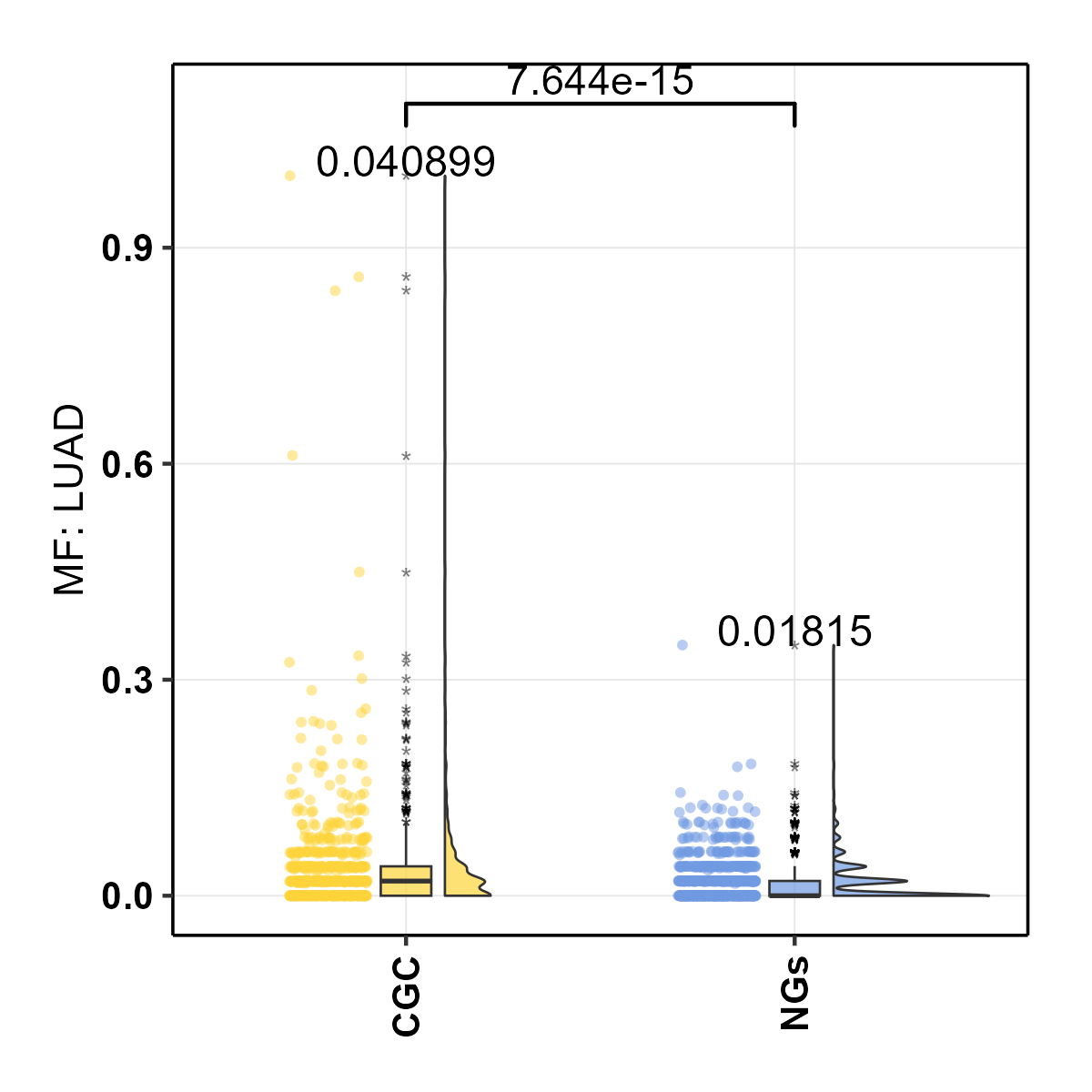

Supplement: Supplementary file 3 [file DataSheet1.ZIP › Supplementary file 5-1/IReflndex_2015/MF_LUAD.png]

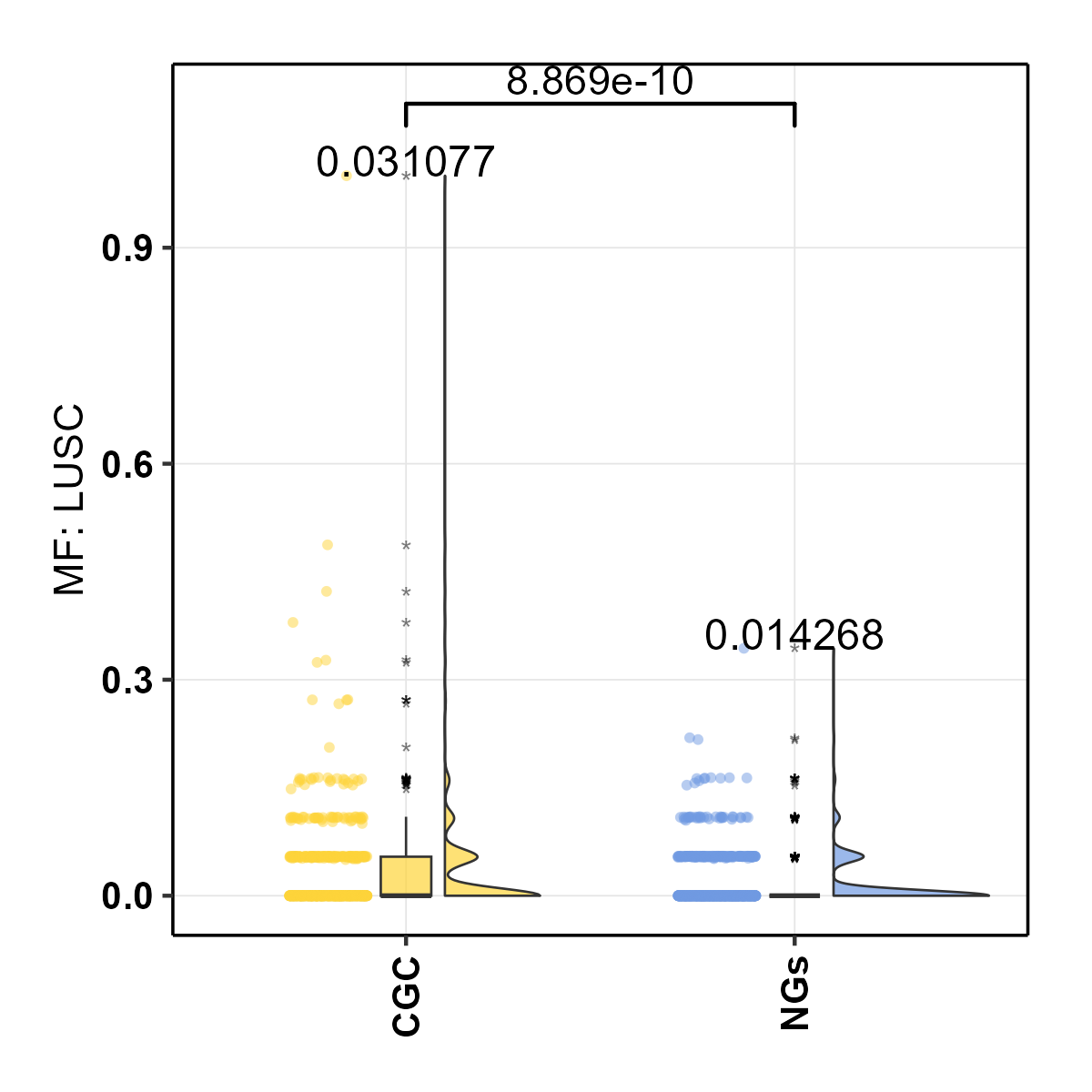

Supplement: Supplementary file 3 [file DataSheet1.ZIP › Supplementary file 5-1/IReflndex_2015/MF_LUSC.png]

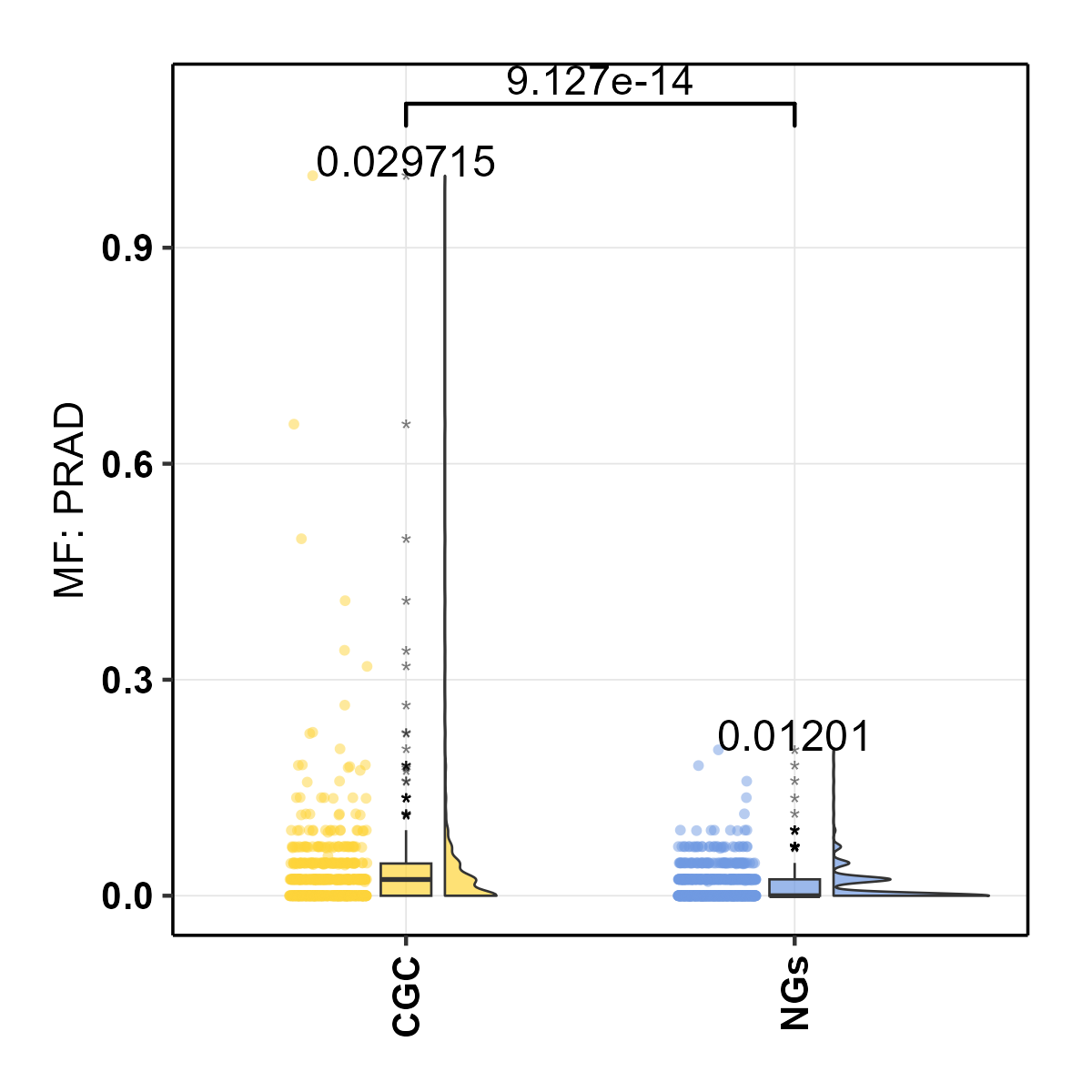

Supplement: Supplementary file 3 [file DataSheet1.ZIP › Supplementary file 5-1/IReflndex_2015/MF_PRAD.png]

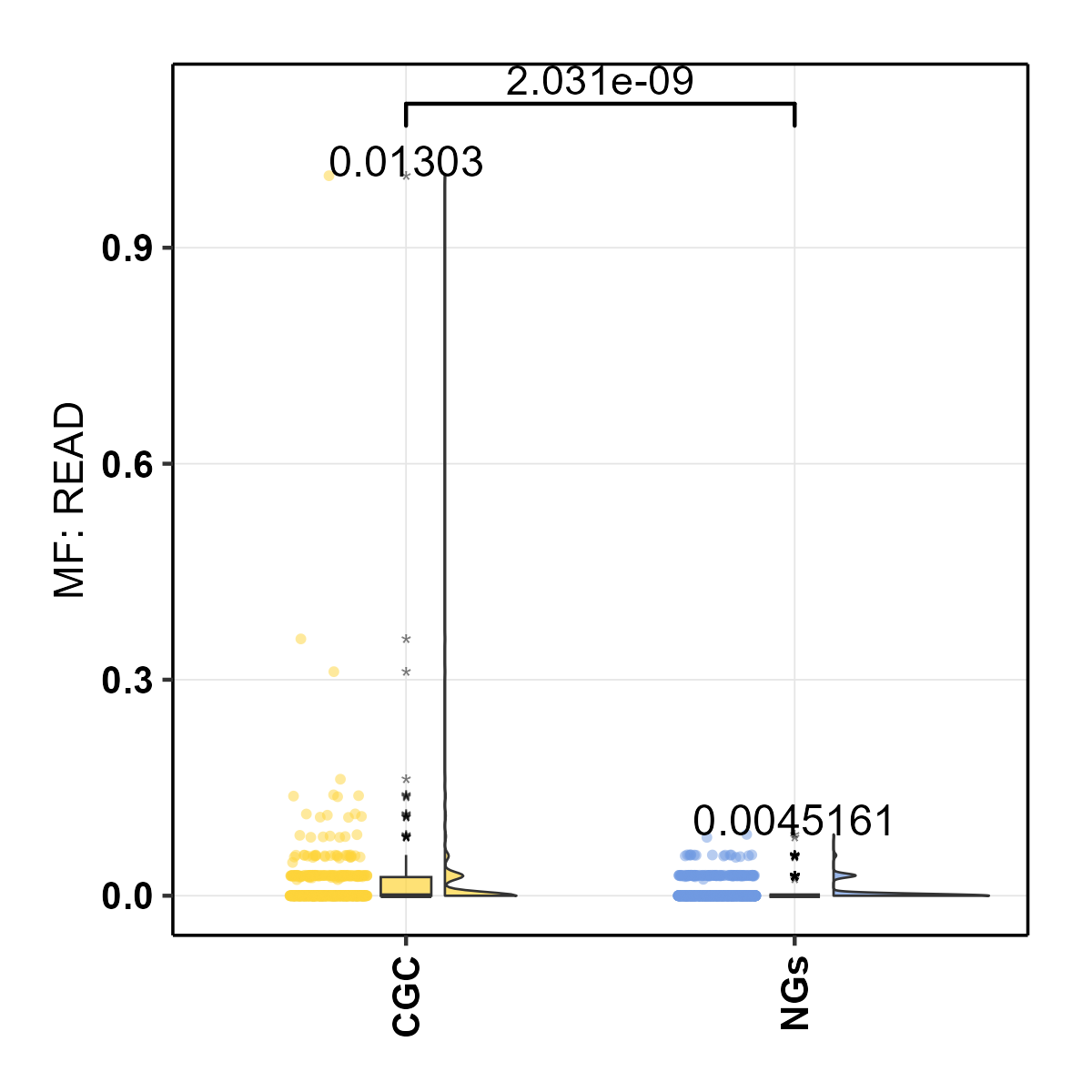

Supplement: Supplementary file 3 [file DataSheet1.ZIP › Supplementary file 5-1/IReflndex_2015/MF_READ.png]

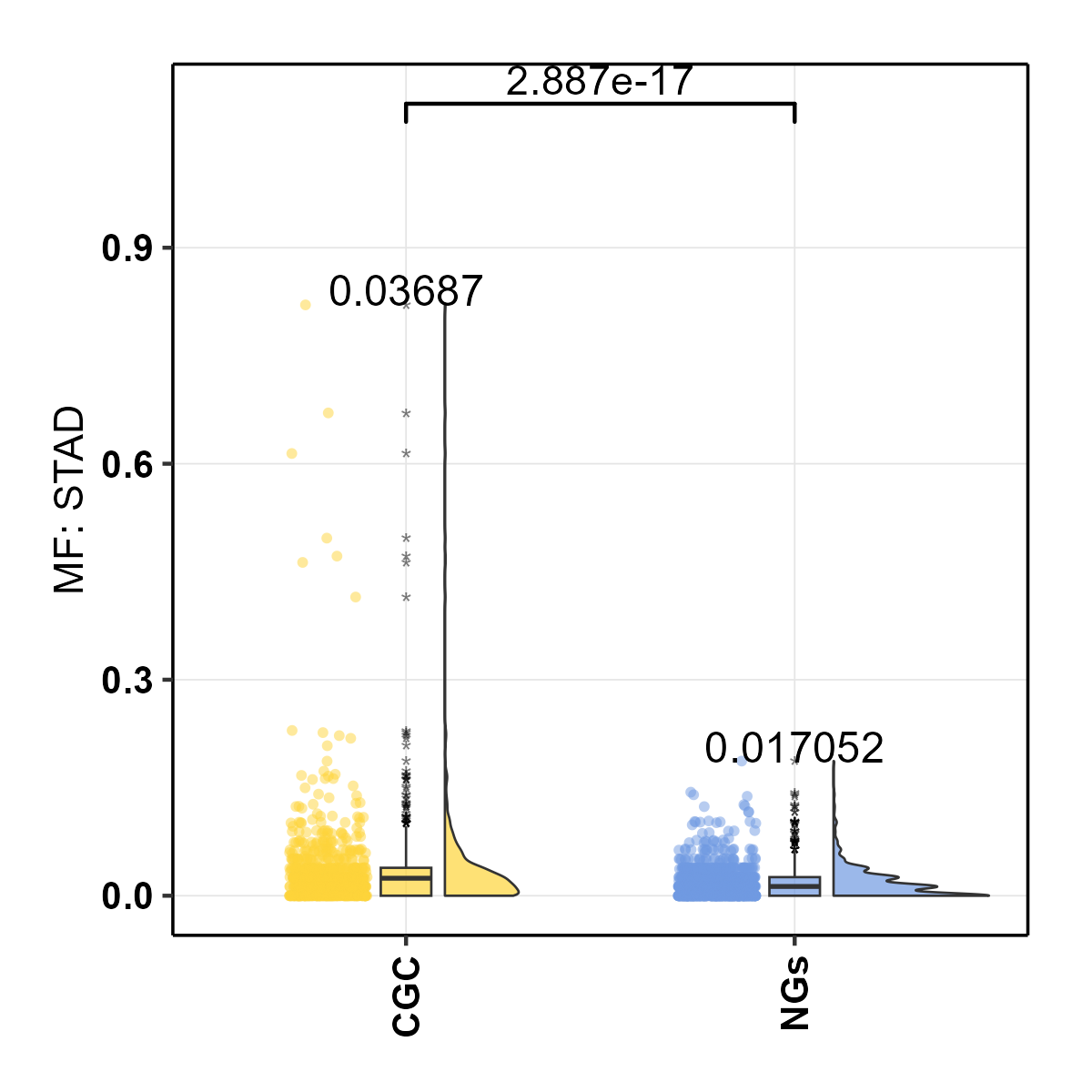

Supplement: Supplementary file 3 [file DataSheet1.ZIP › Supplementary file 5-1/IReflndex_2015/MF_STAD.png]

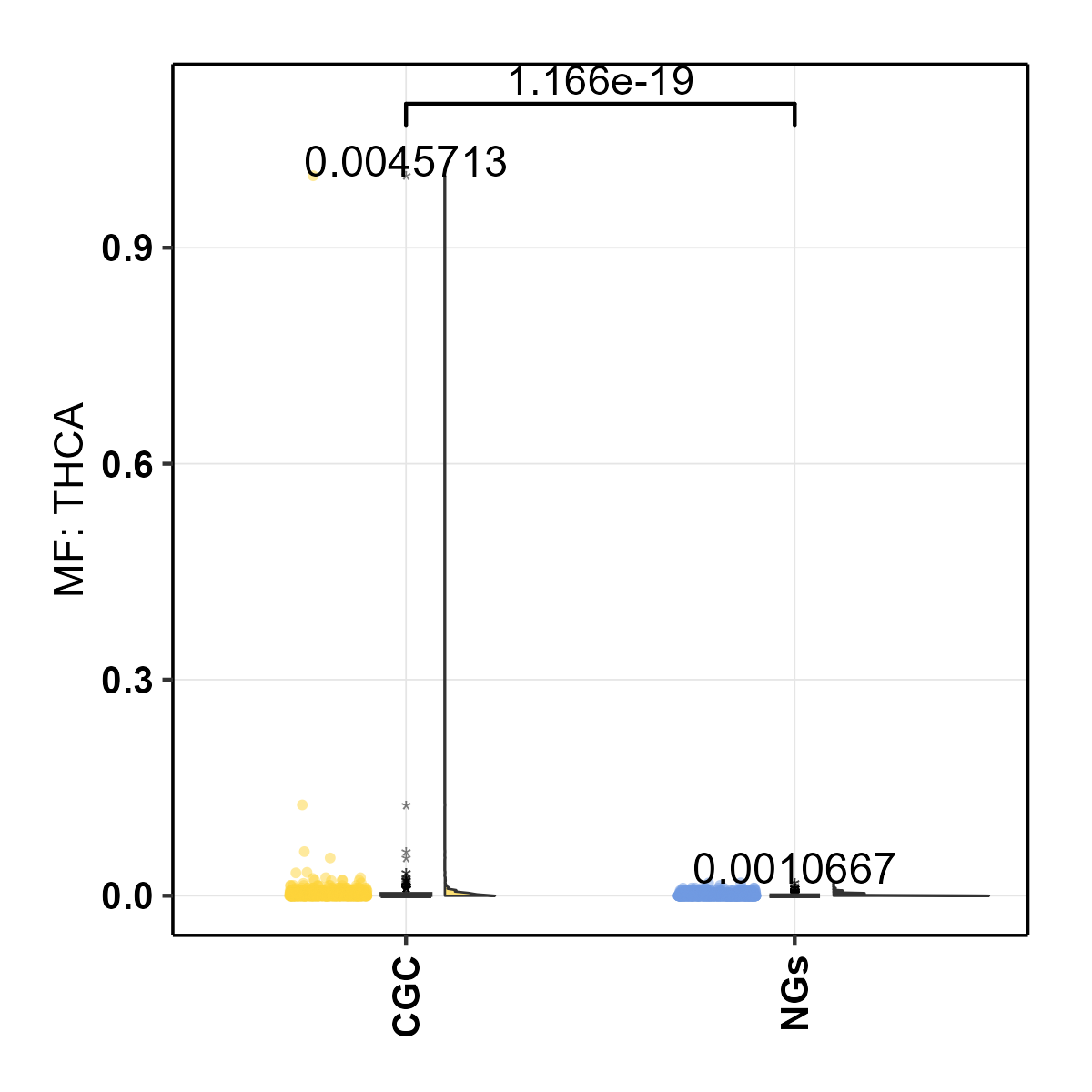

Supplement: Supplementary file 3 [file DataSheet1.ZIP › Supplementary file 5-1/IReflndex_2015/MF_THCA.png]

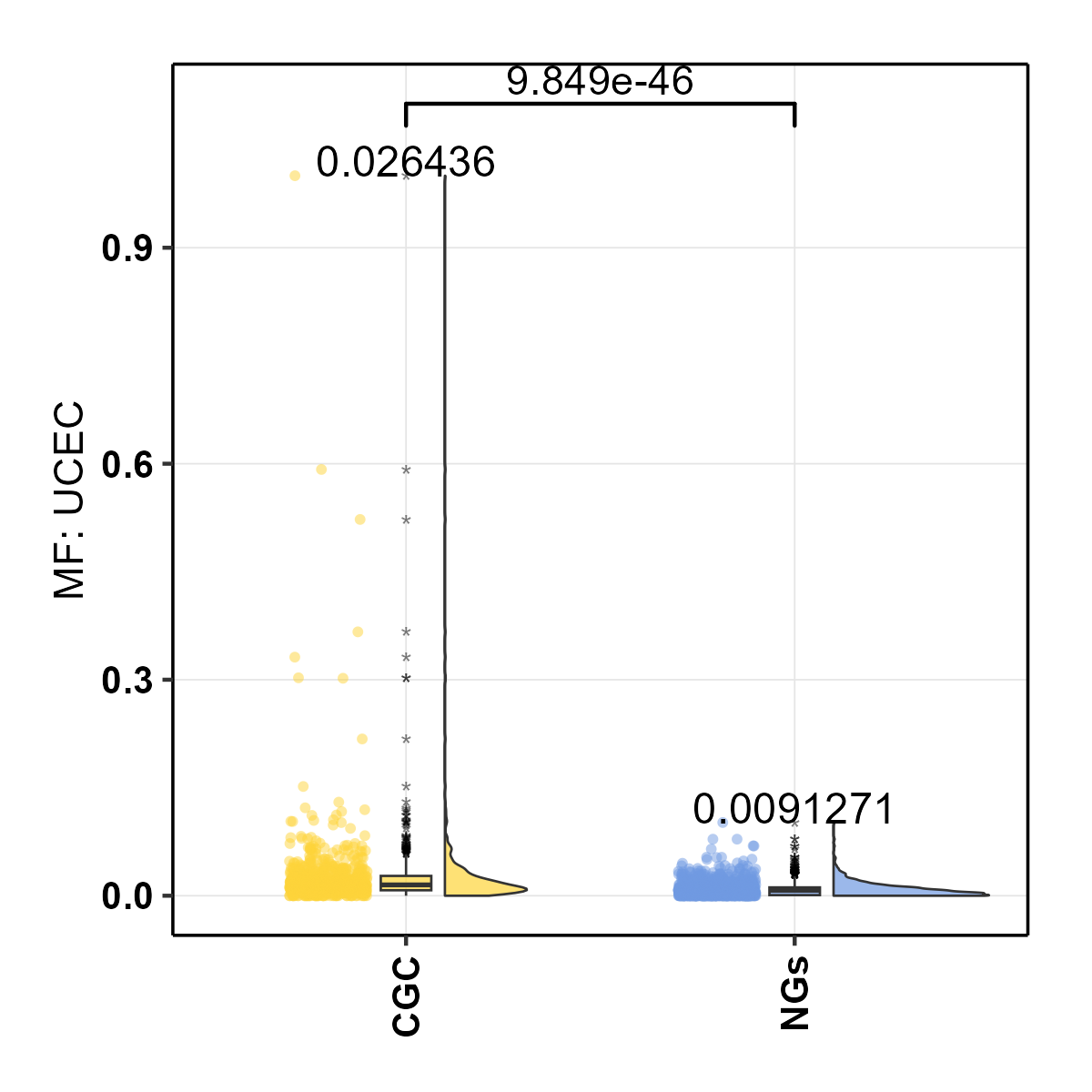

Supplement: Supplementary file 3 [file DataSheet1.ZIP › Supplementary file 5-1/IReflndex_2015/MF_UCEC.png]

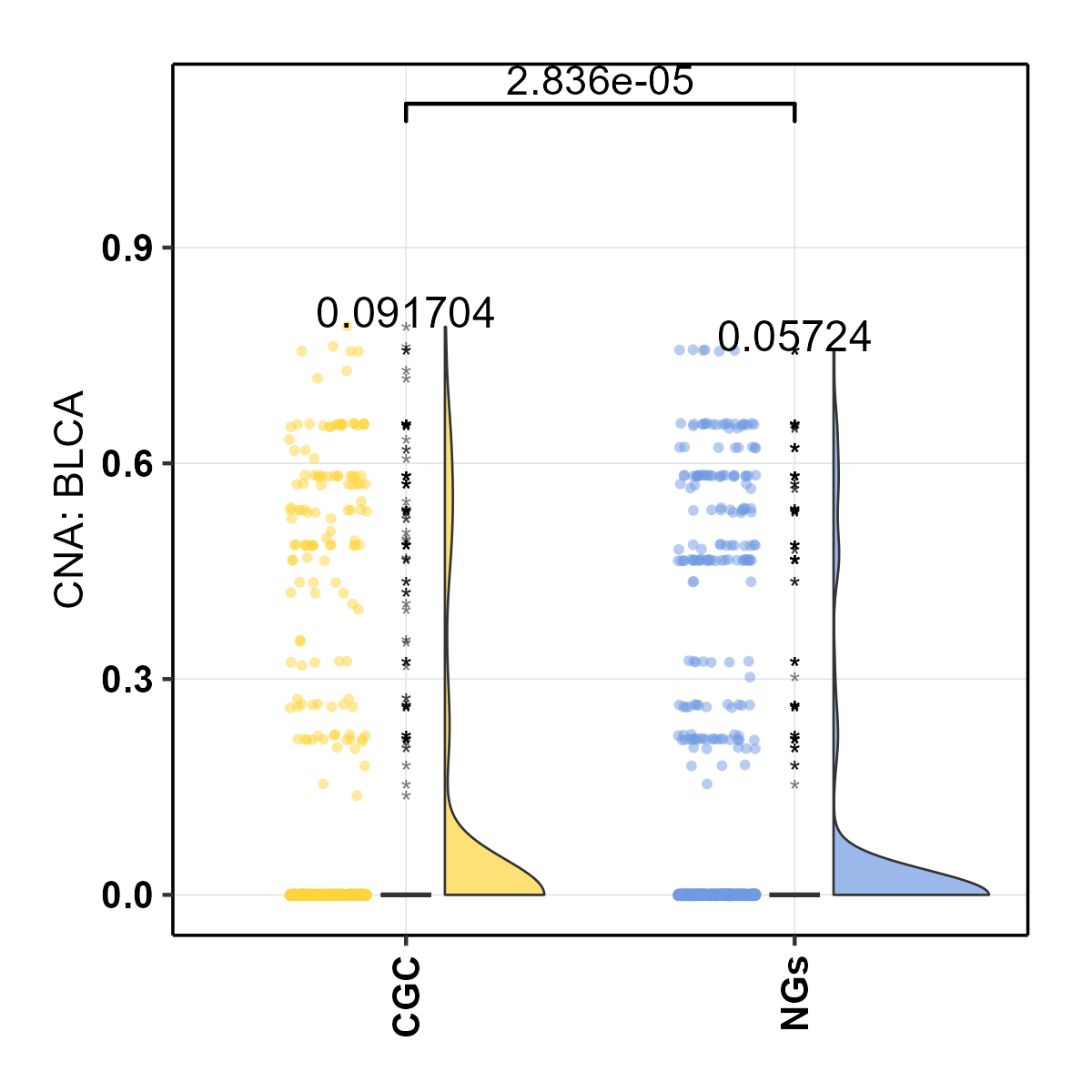

Supplement: Supplementary file 3 [file DataSheet1.ZIP › Supplementary file 5-1/Multinet/CNA_BLCA.png]

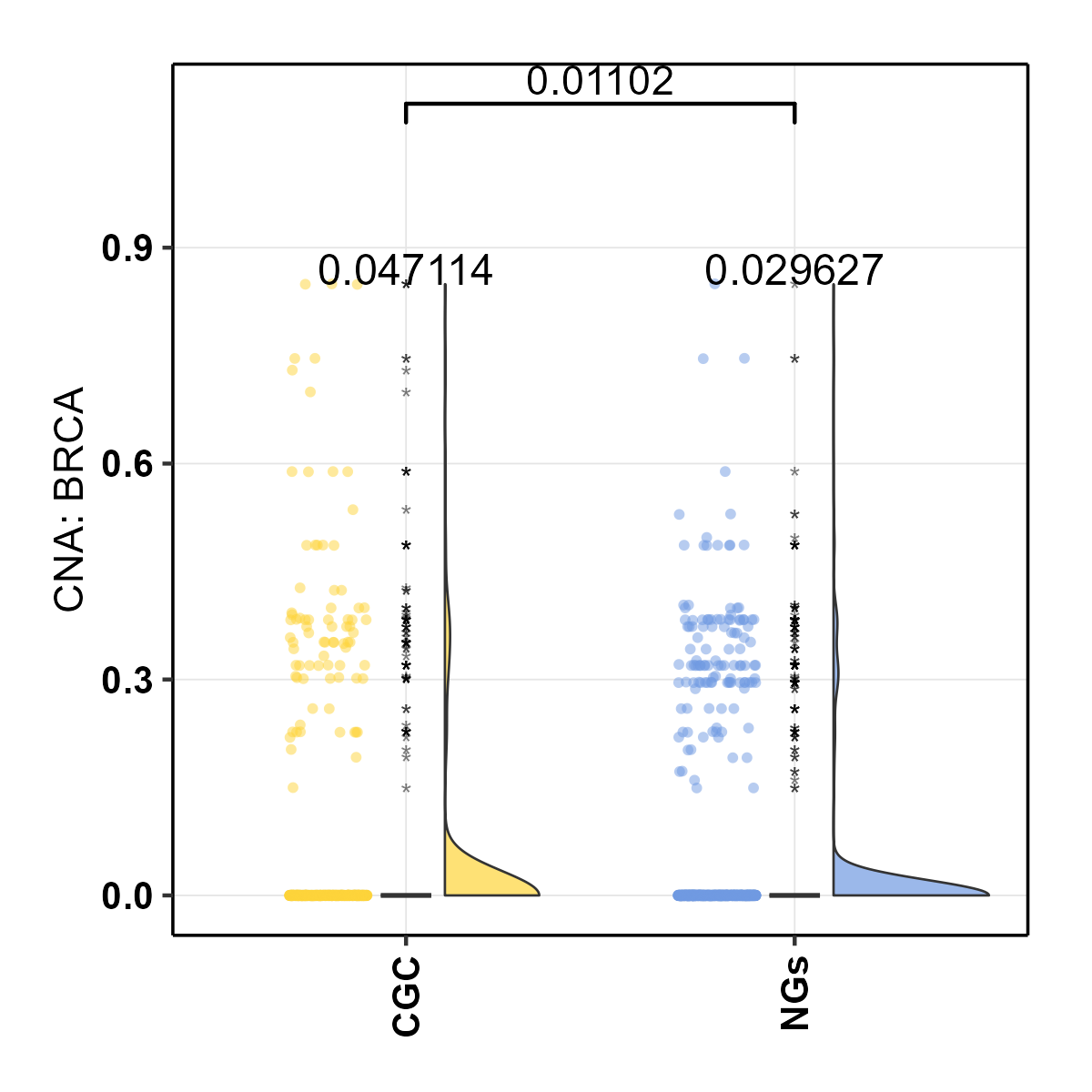

Supplement: Supplementary file 3 [file DataSheet1.ZIP › Supplementary file 5-1/Multinet/CNA_BRCA.png]

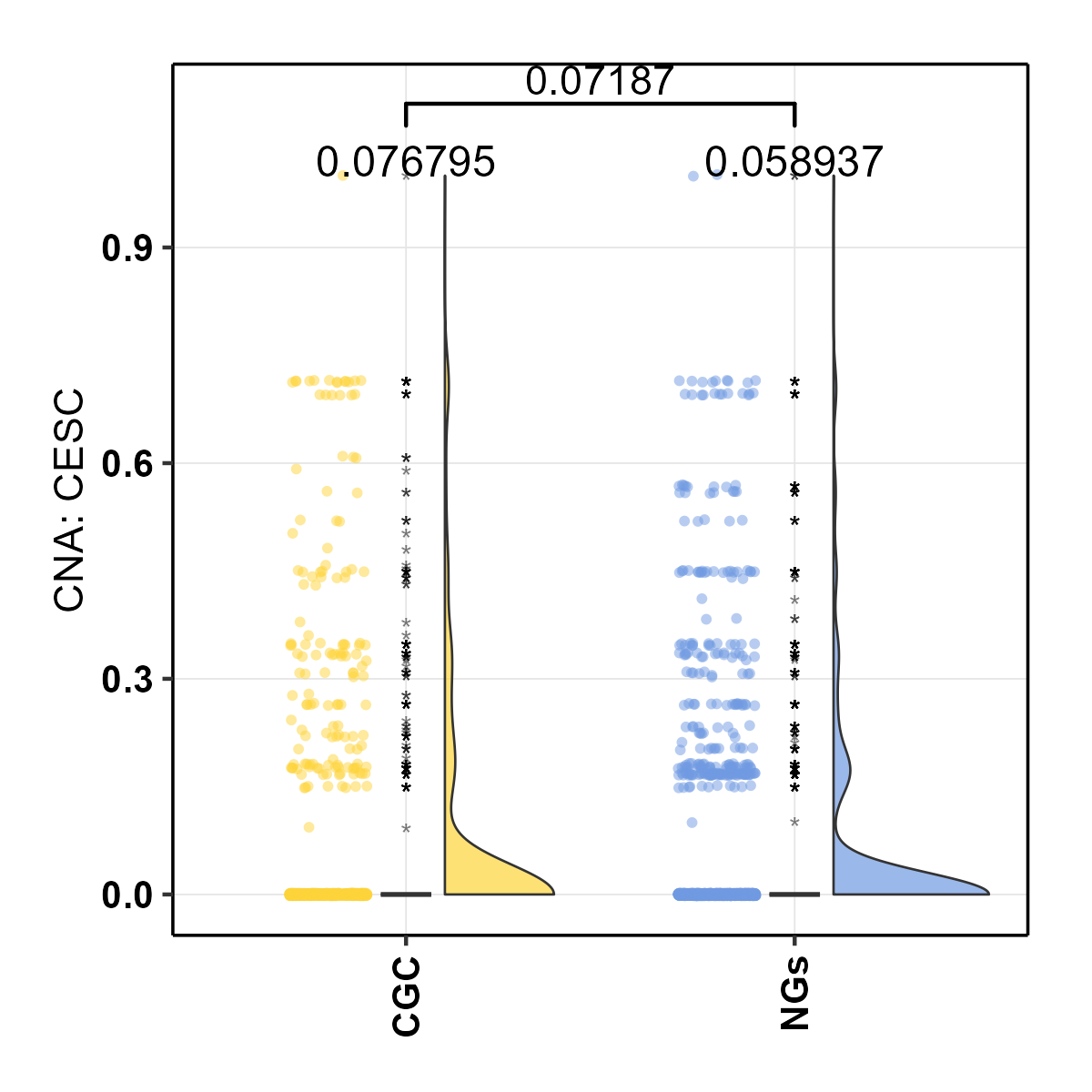

Supplement: Supplementary file 3 [file DataSheet1.ZIP › Supplementary file 5-1/Multinet/CNA_CESC.png]

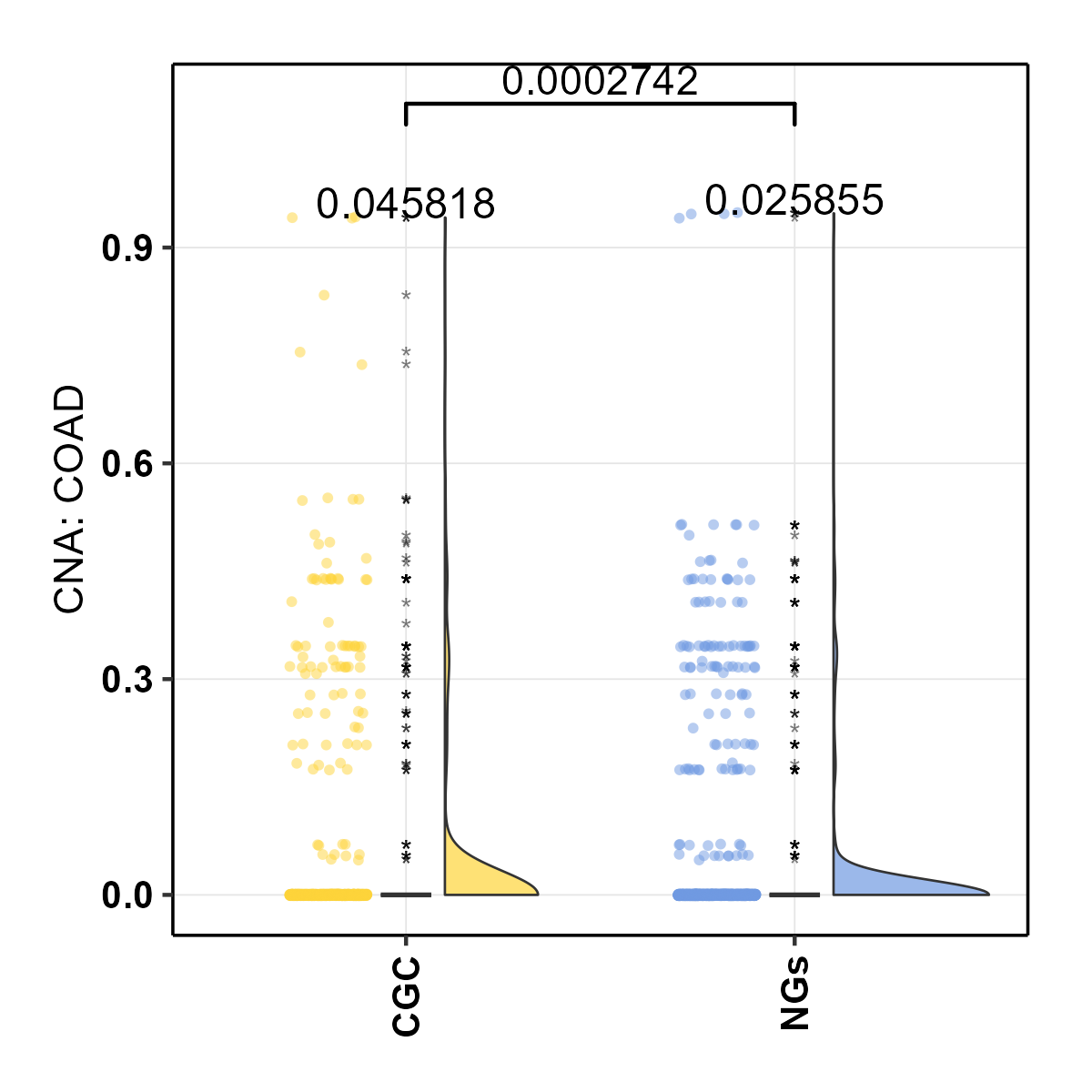

Supplement: Supplementary file 3 [file DataSheet1.ZIP › Supplementary file 5-1/Multinet/CNA_COAD.png]

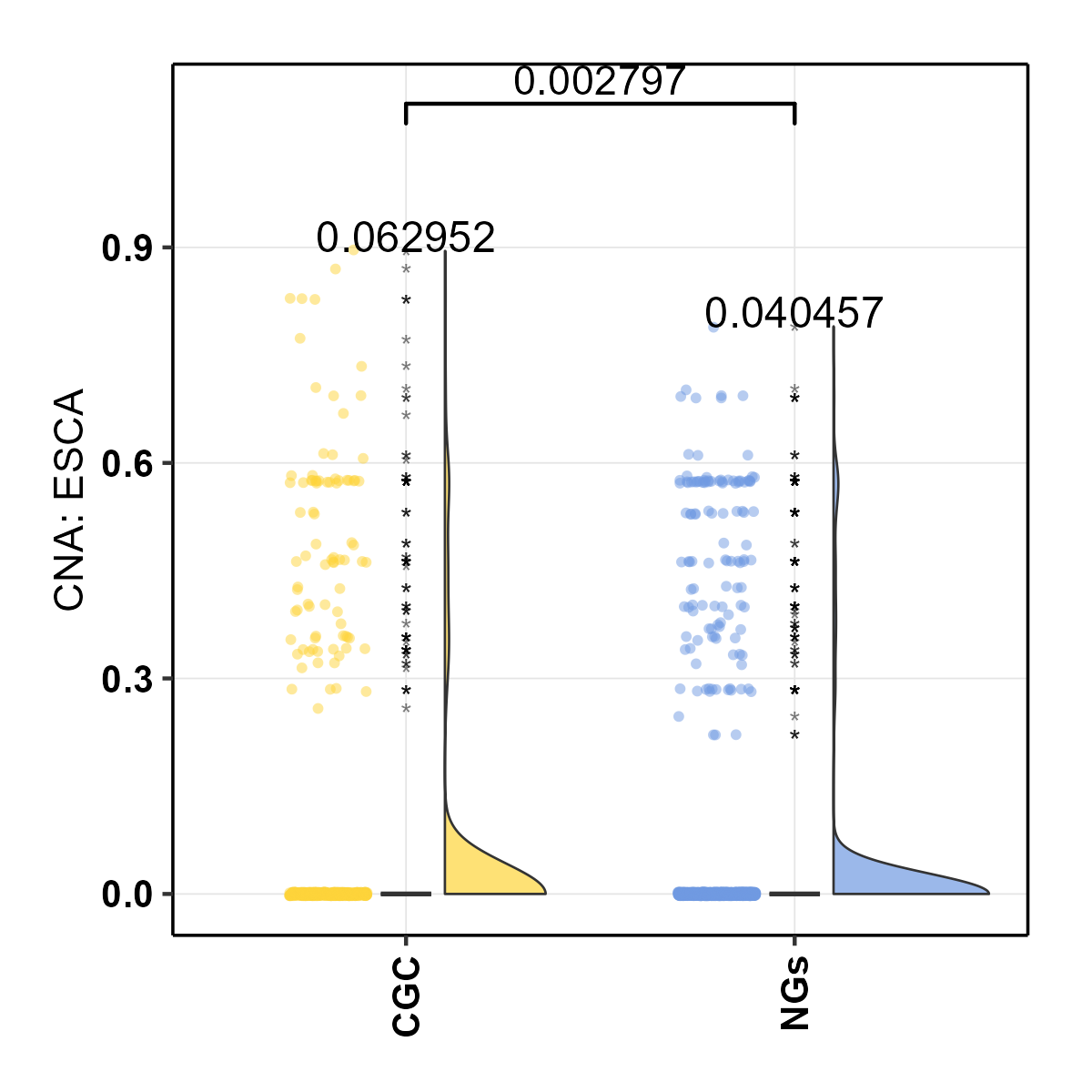

Supplement: Supplementary file 3 [file DataSheet1.ZIP › Supplementary file 5-1/Multinet/CNA_ESCA.png]

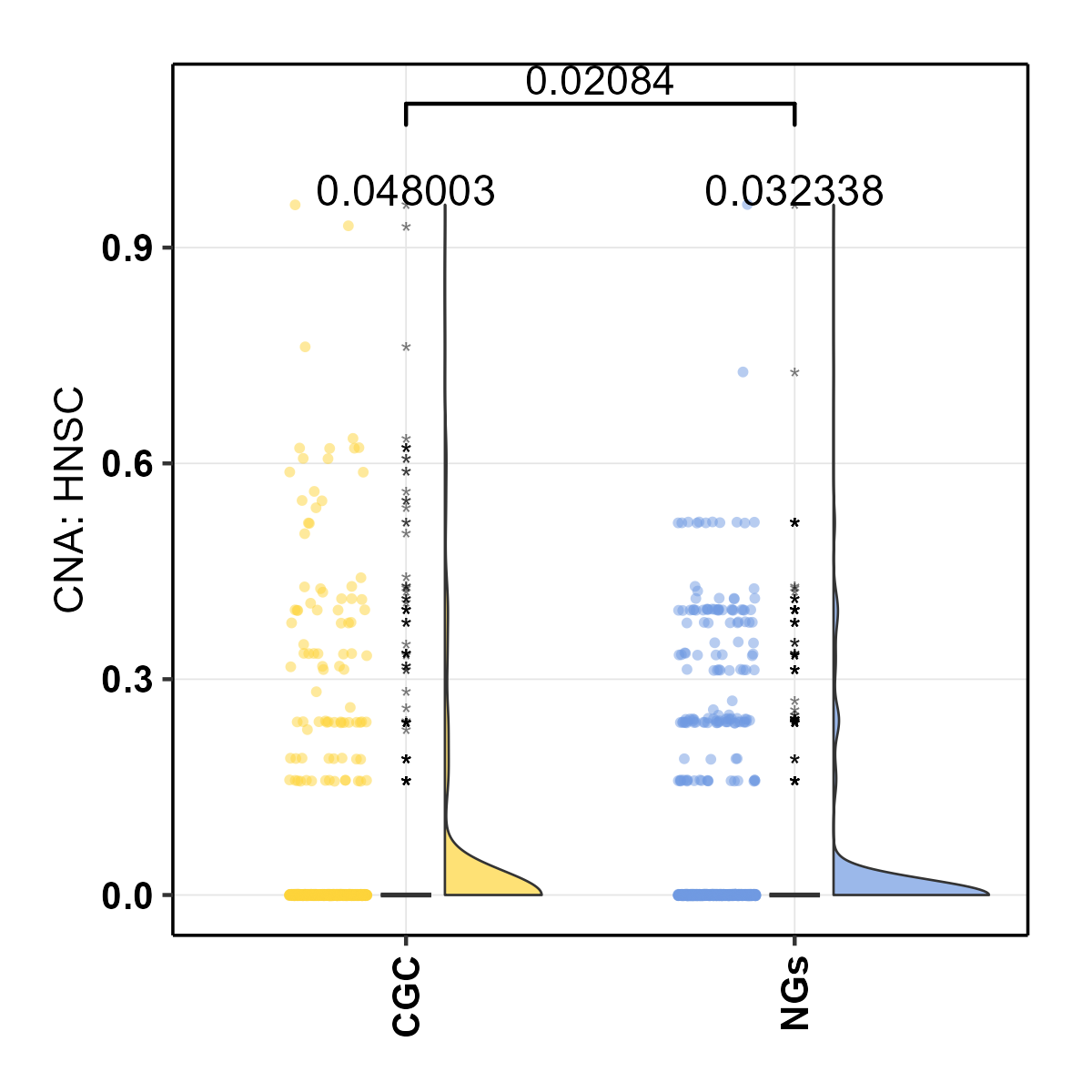

Supplement: Supplementary file 3 [file DataSheet1.ZIP › Supplementary file 5-1/Multinet/CNA_HNSC.png]

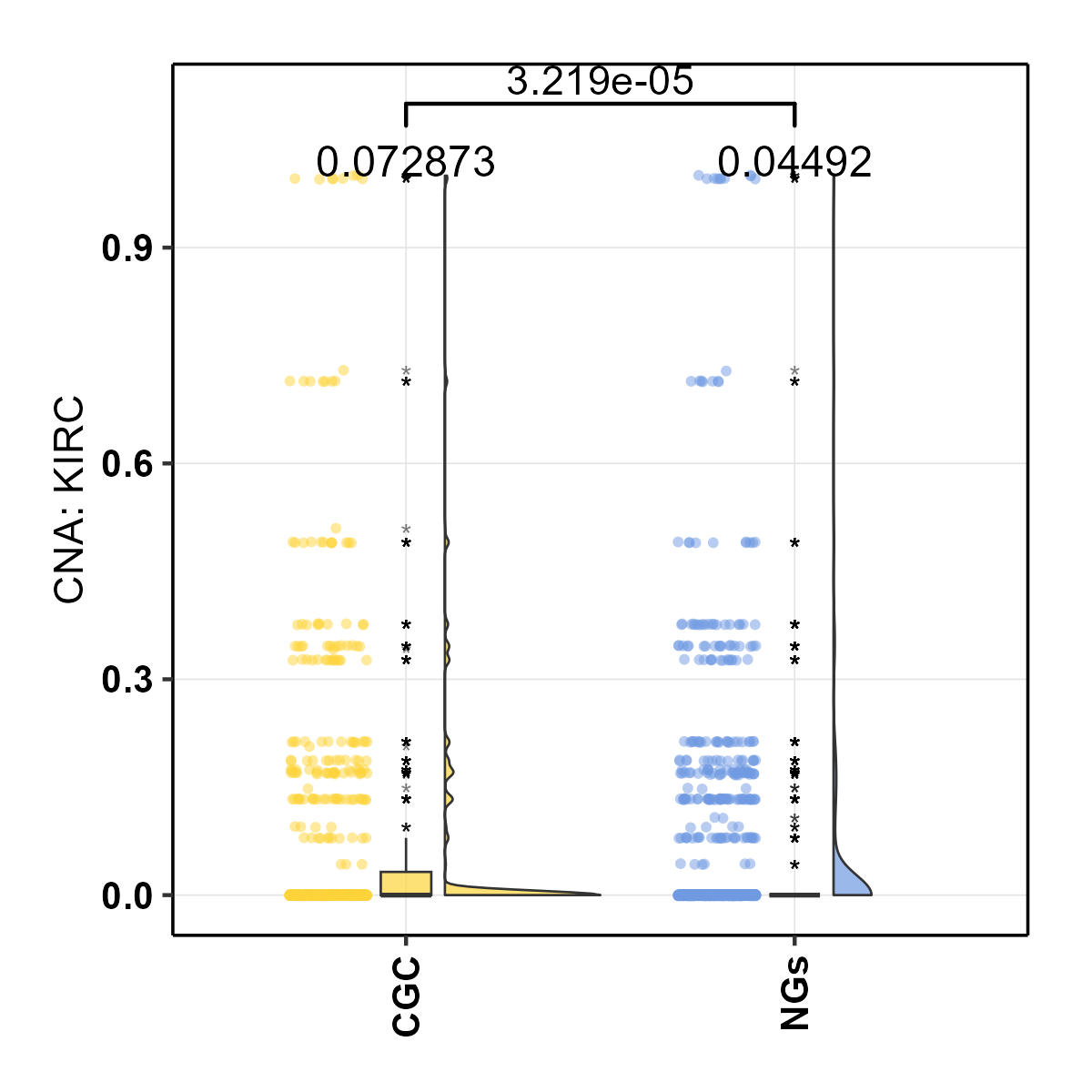

Supplement: Supplementary file 3 [file DataSheet1.ZIP › Supplementary file 5-1/Multinet/CNA_KIRC.png]

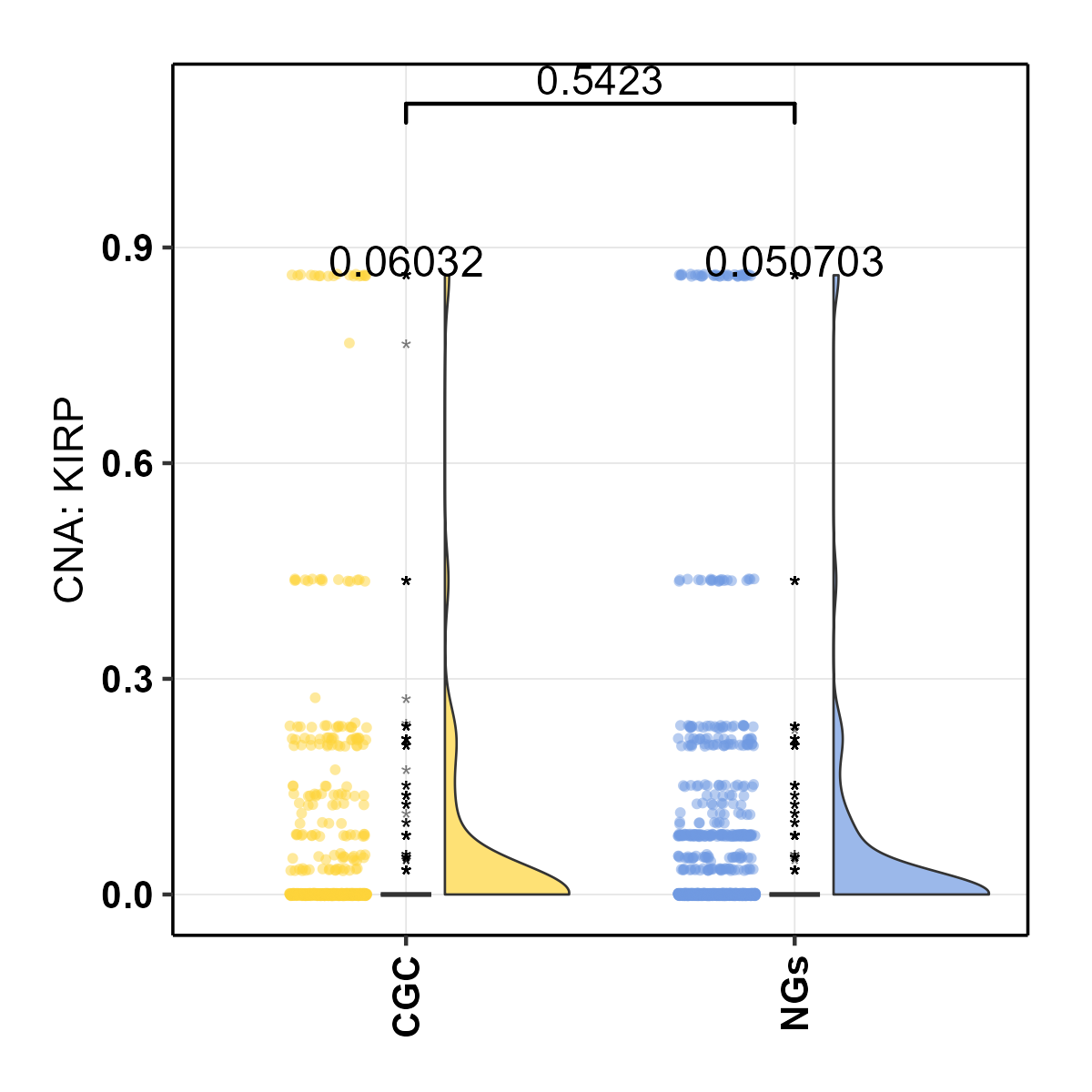

Supplement: Supplementary file 3 [file DataSheet1.ZIP › Supplementary file 5-1/Multinet/CNA_KIRP.png]

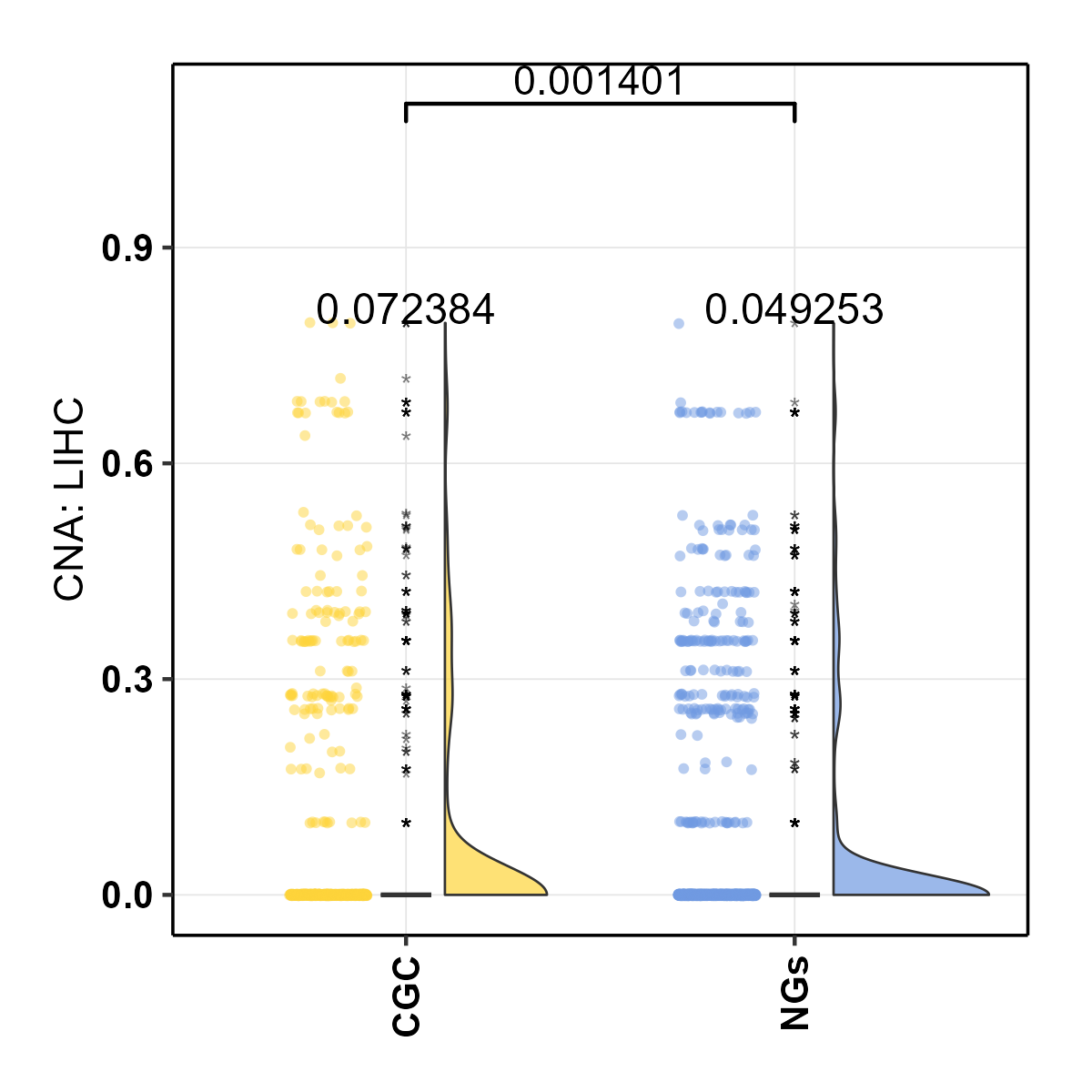

Supplement: Supplementary file 3 [file DataSheet1.ZIP › Supplementary file 5-1/Multinet/CNA_LIHC.png]

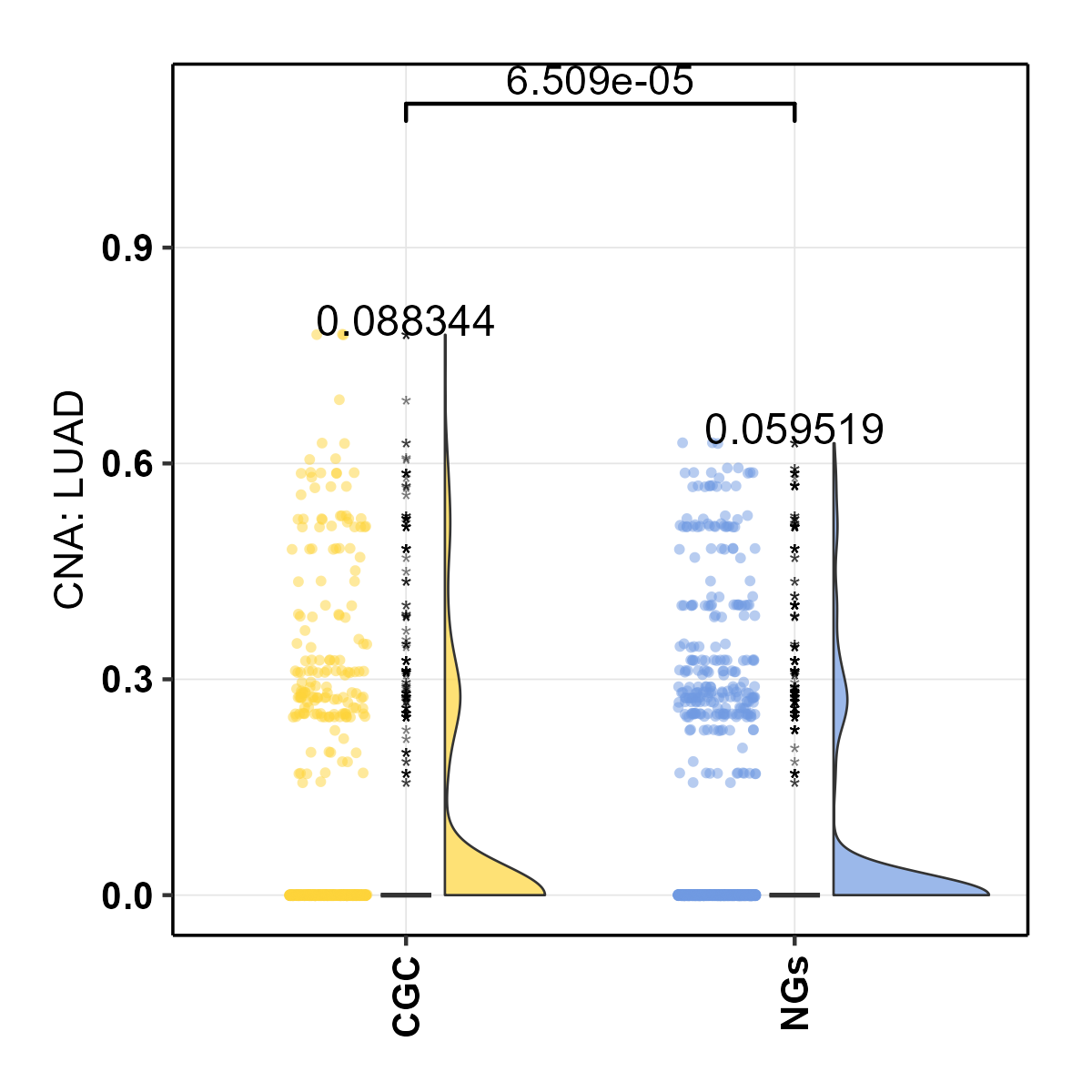

Supplement: Supplementary file 3 [file DataSheet1.ZIP › Supplementary file 5-1/Multinet/CNA_LUAD.png]

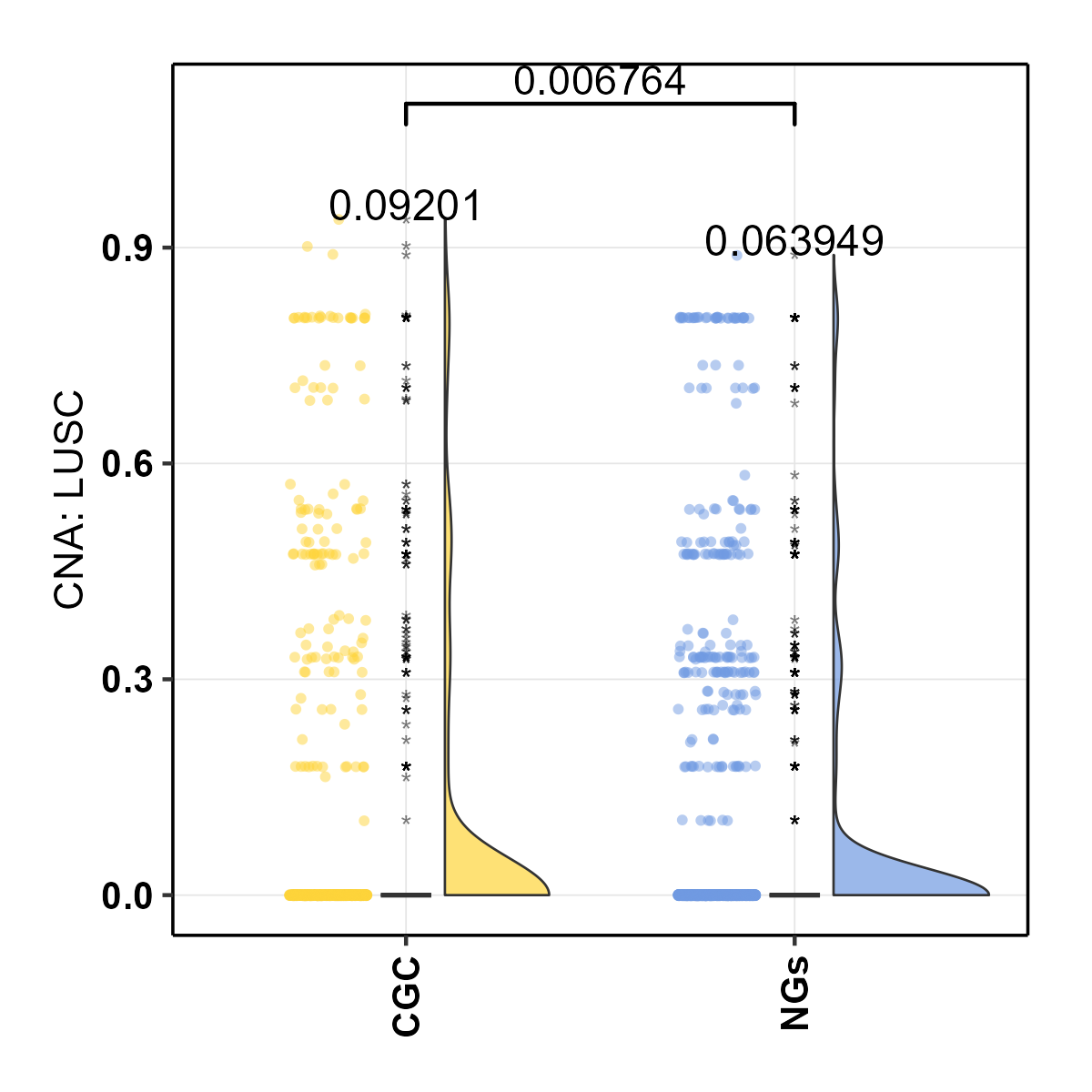

Supplement: Supplementary file 3 [file DataSheet1.ZIP › Supplementary file 5-1/Multinet/CNA_LUSC.png]

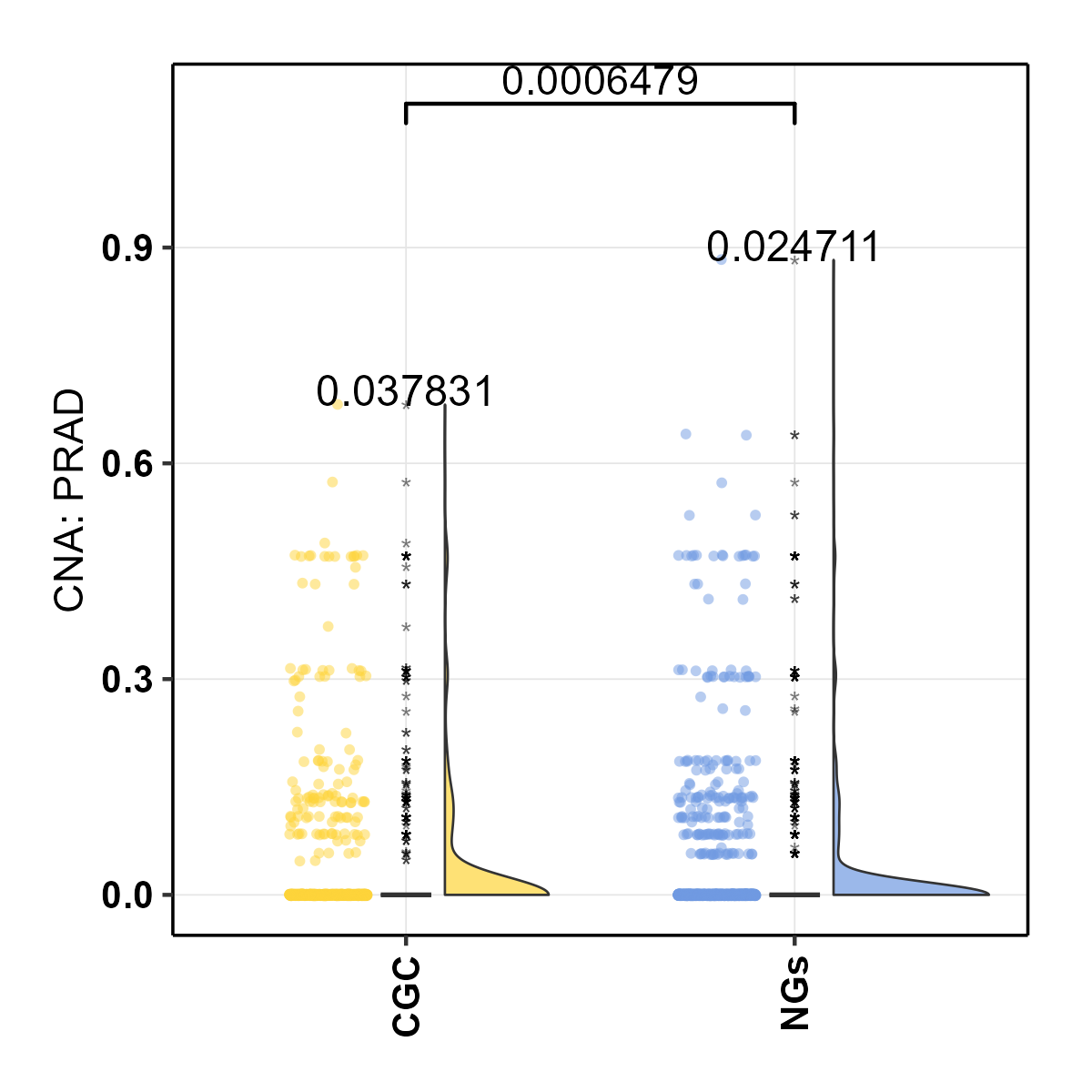

Supplement: Supplementary file 3 [file DataSheet1.ZIP › Supplementary file 5-1/Multinet/CNA_PRAD.png]

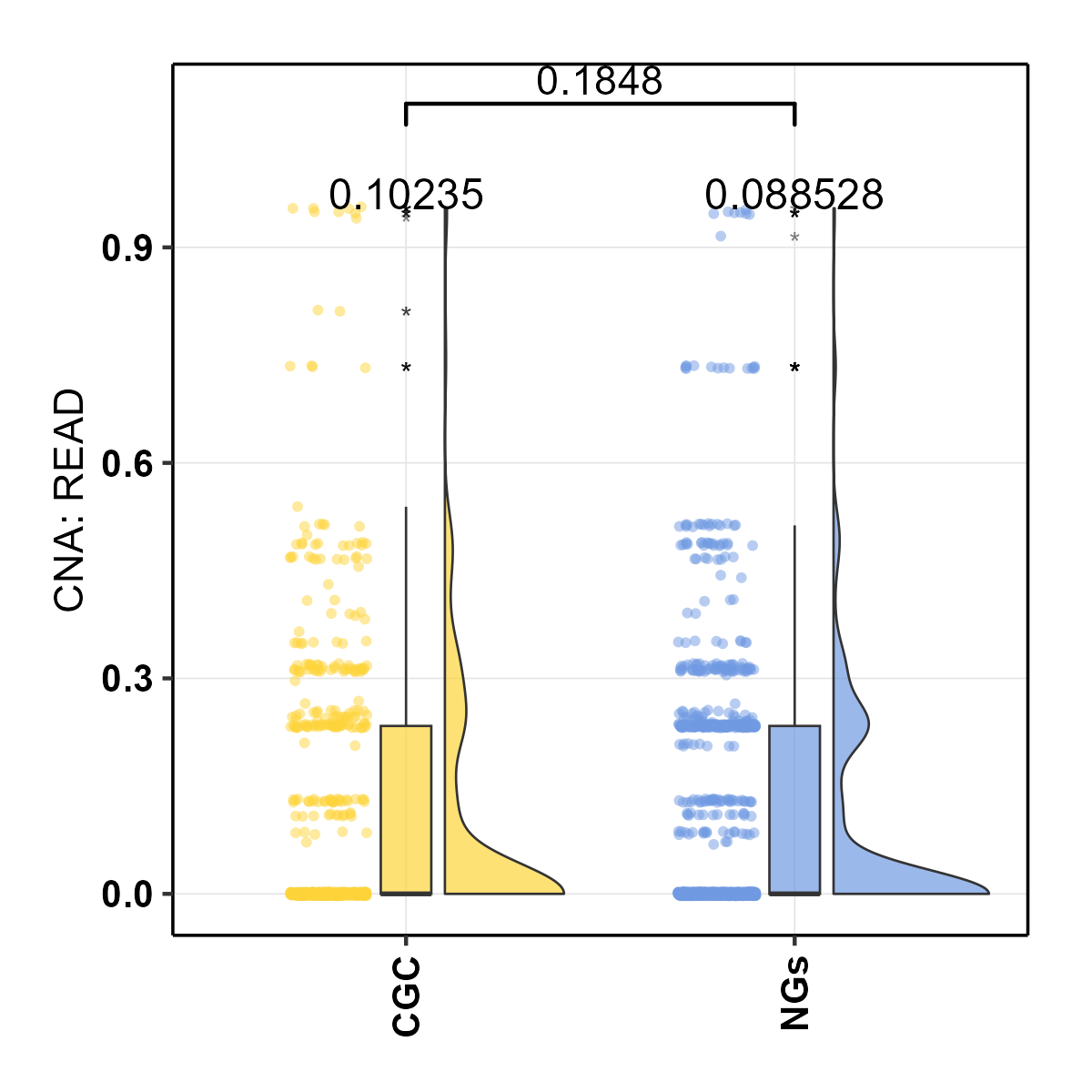

Supplement: Supplementary file 3 [file DataSheet1.ZIP › Supplementary file 5-1/Multinet/CNA_READ.png]

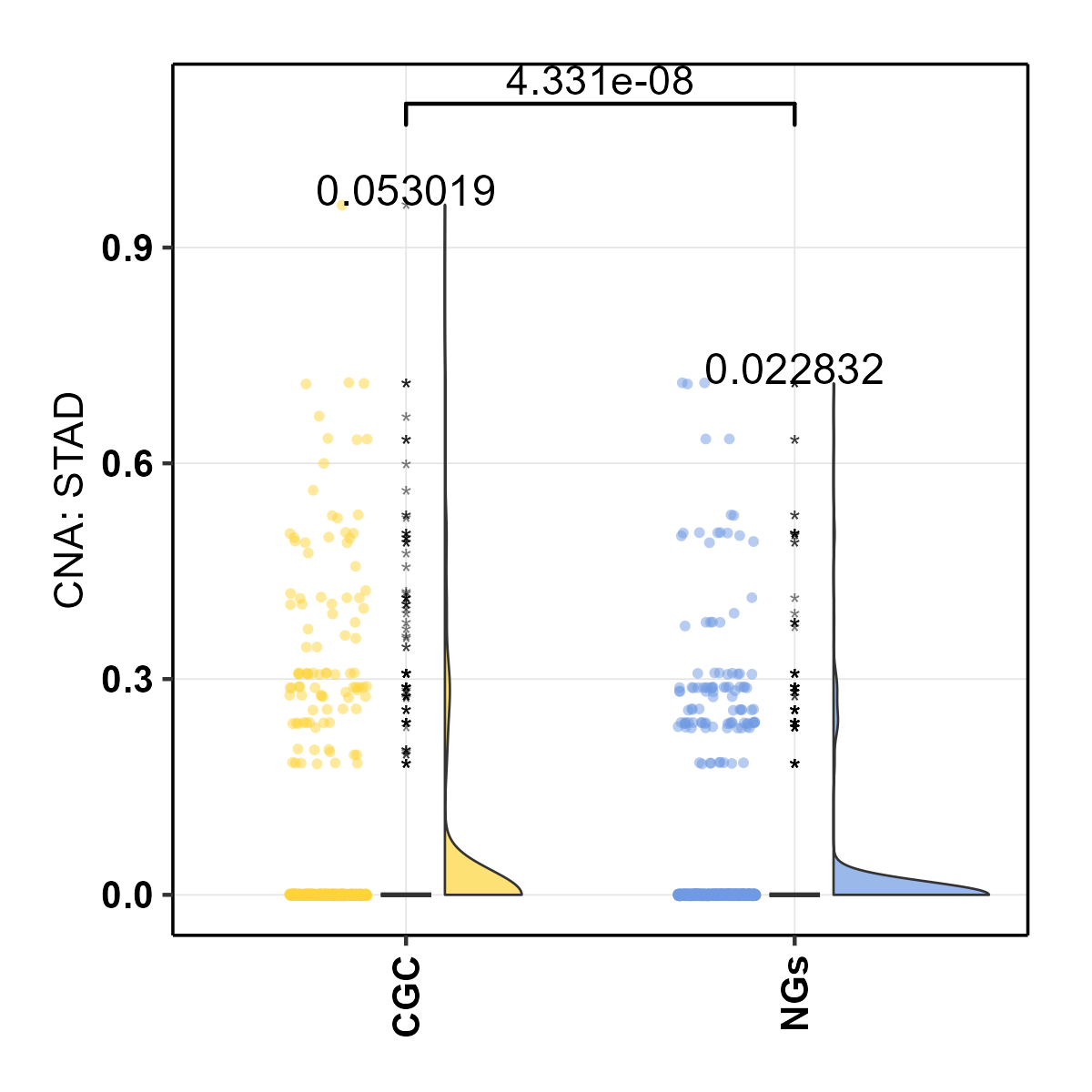

Supplement: Supplementary file 3 [file DataSheet1.ZIP › Supplementary file 5-1/Multinet/CNA_STAD.png]

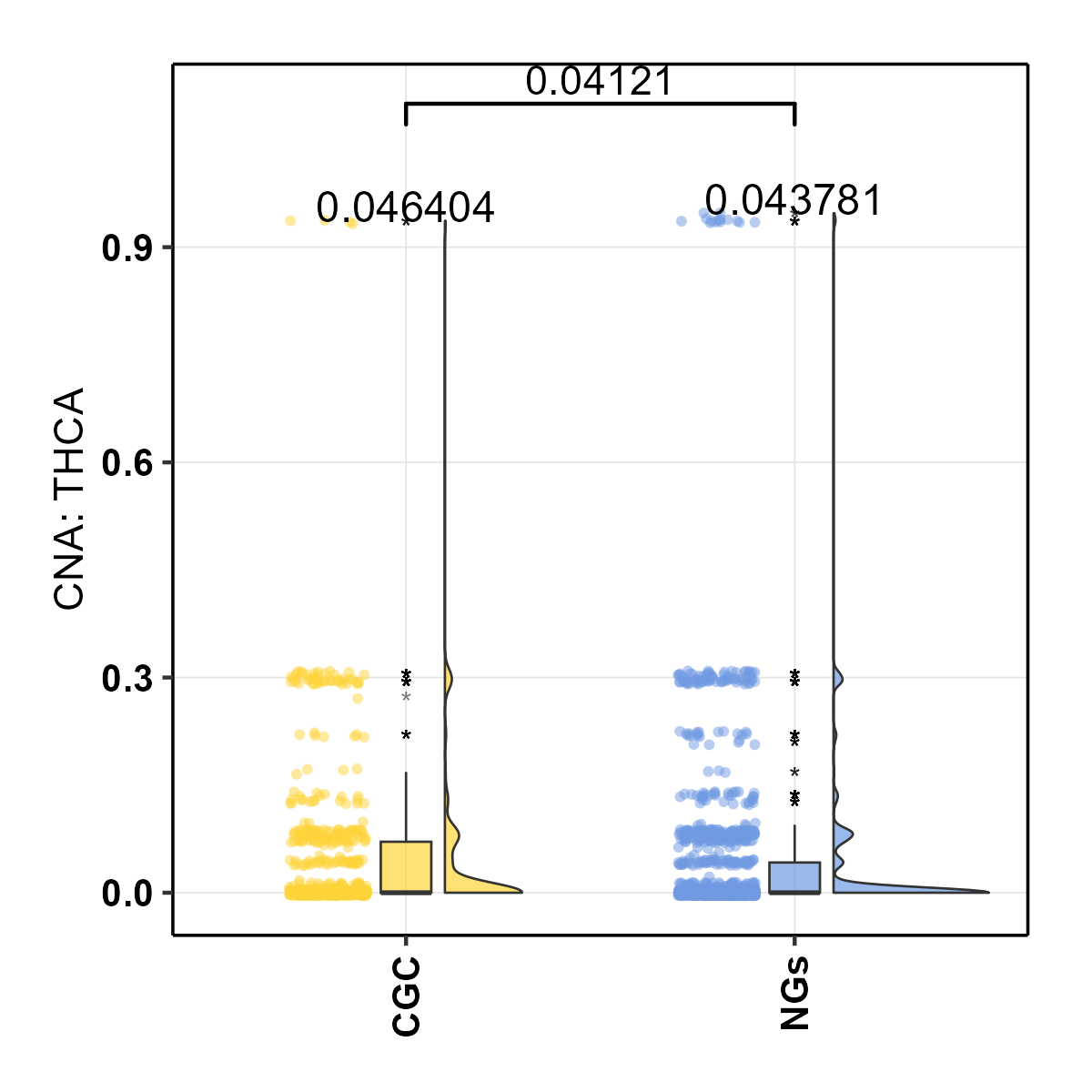

Supplement: Supplementary file 3 [file DataSheet1.ZIP › Supplementary file 5-1/Multinet/CNA_THCA.png]

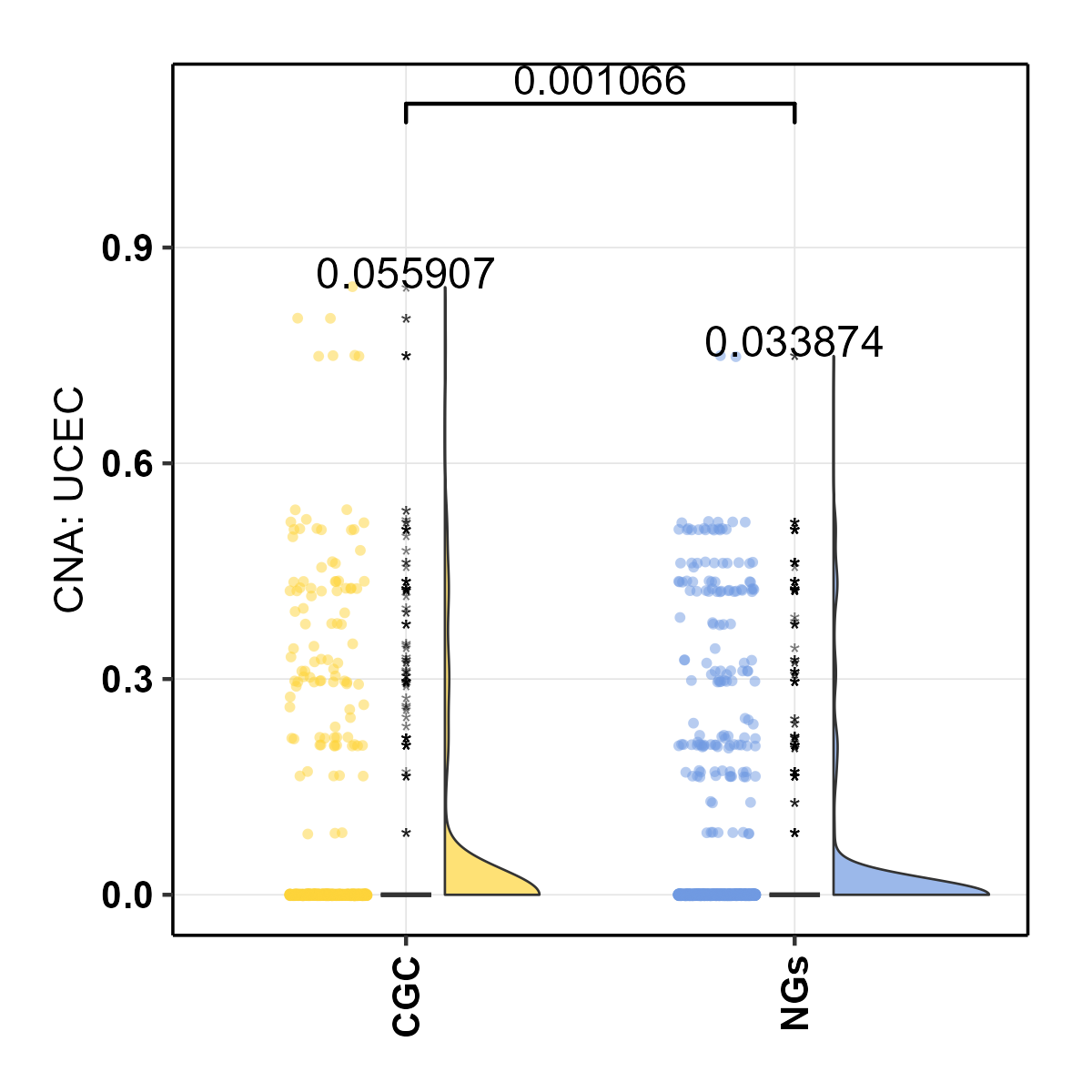

Supplement: Supplementary file 3 [file DataSheet1.ZIP › Supplementary file 5-1/Multinet/CNA_UCEC.png]

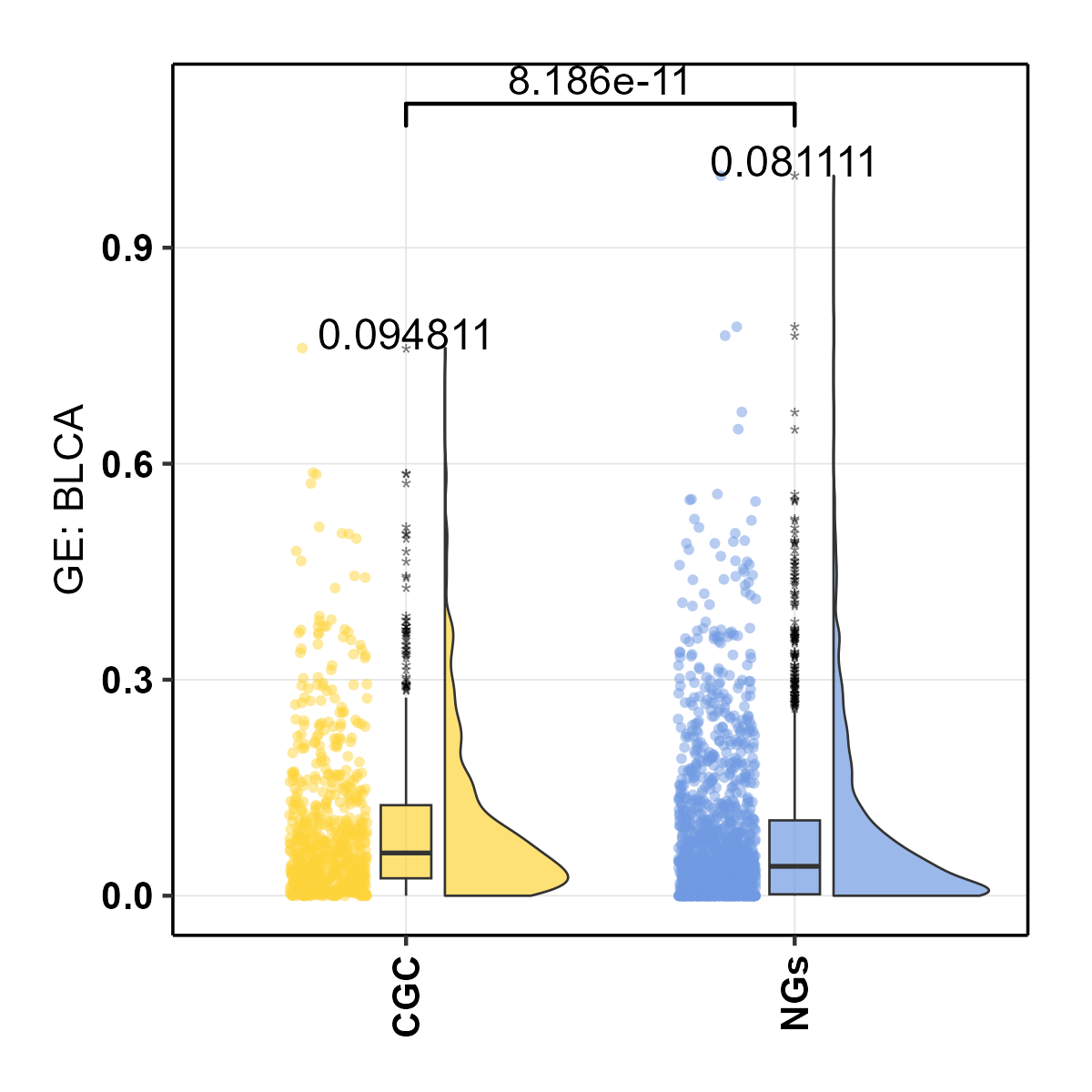

Supplement: Supplementary file 3 [file DataSheet1.ZIP › Supplementary file 5-1/Multinet/GE_BLCA.png]

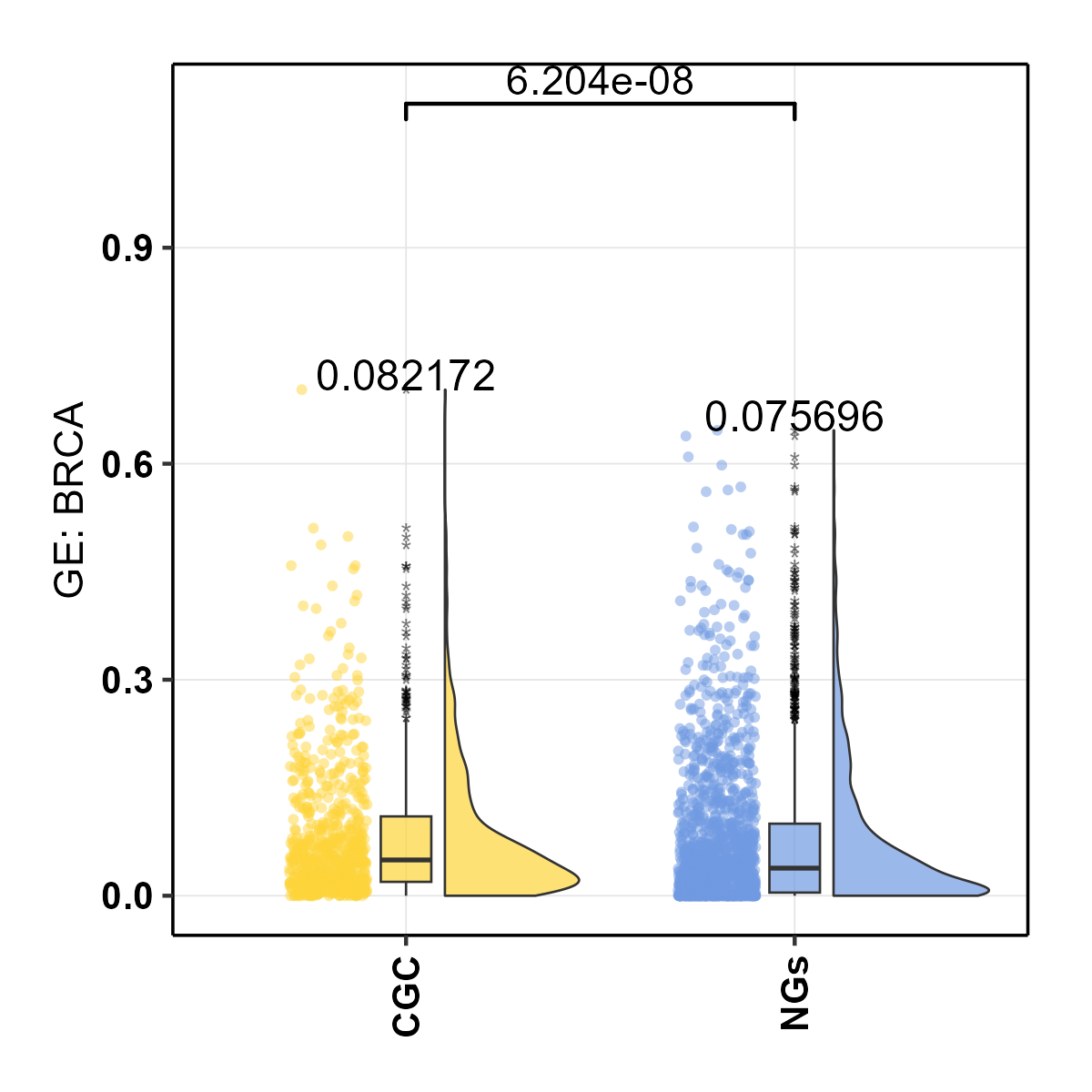

Supplement: Supplementary file 3 [file DataSheet1.ZIP › Supplementary file 5-1/Multinet/GE_BRCA.png]

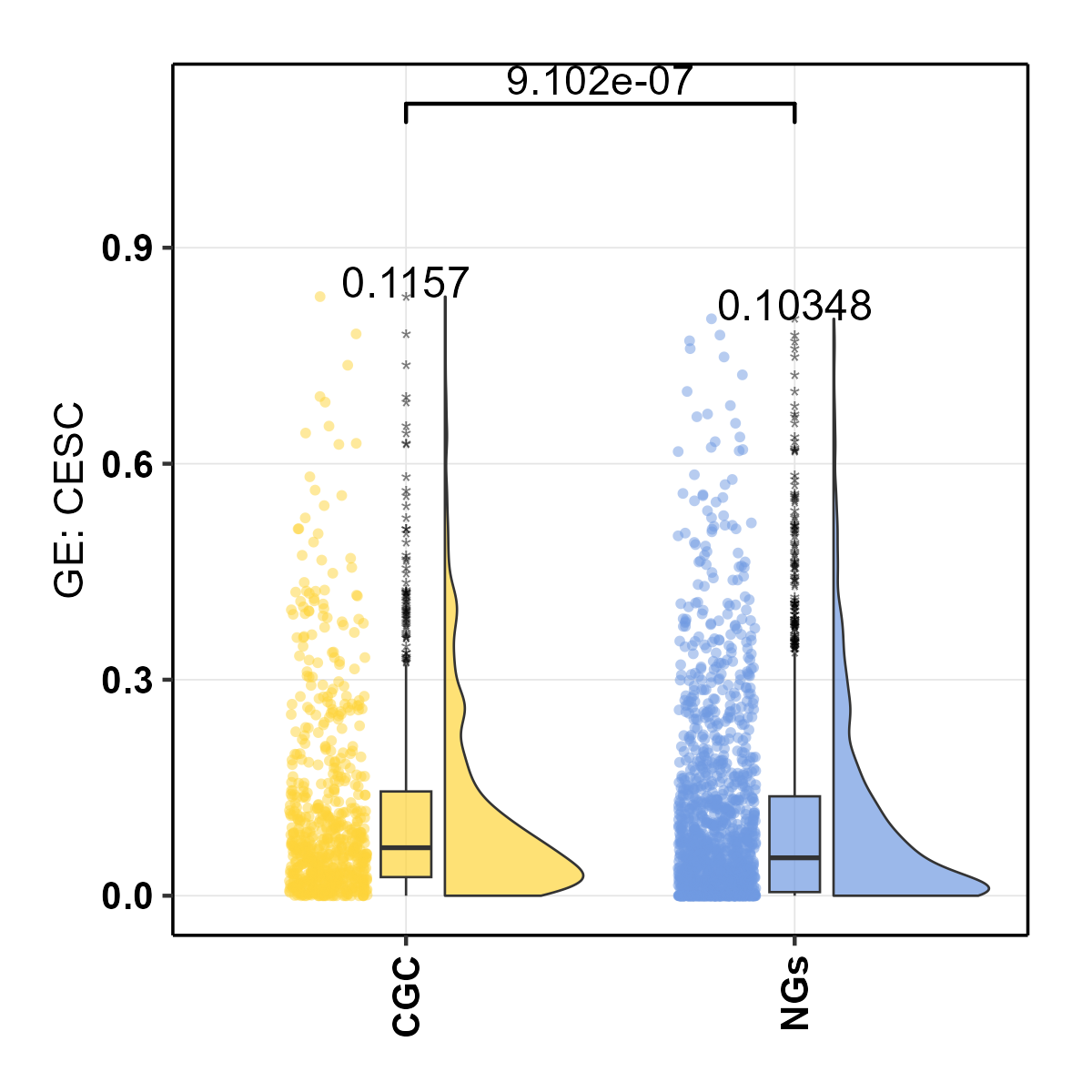

Supplement: Supplementary file 3 [file DataSheet1.ZIP › Supplementary file 5-1/Multinet/GE_CESC.png]

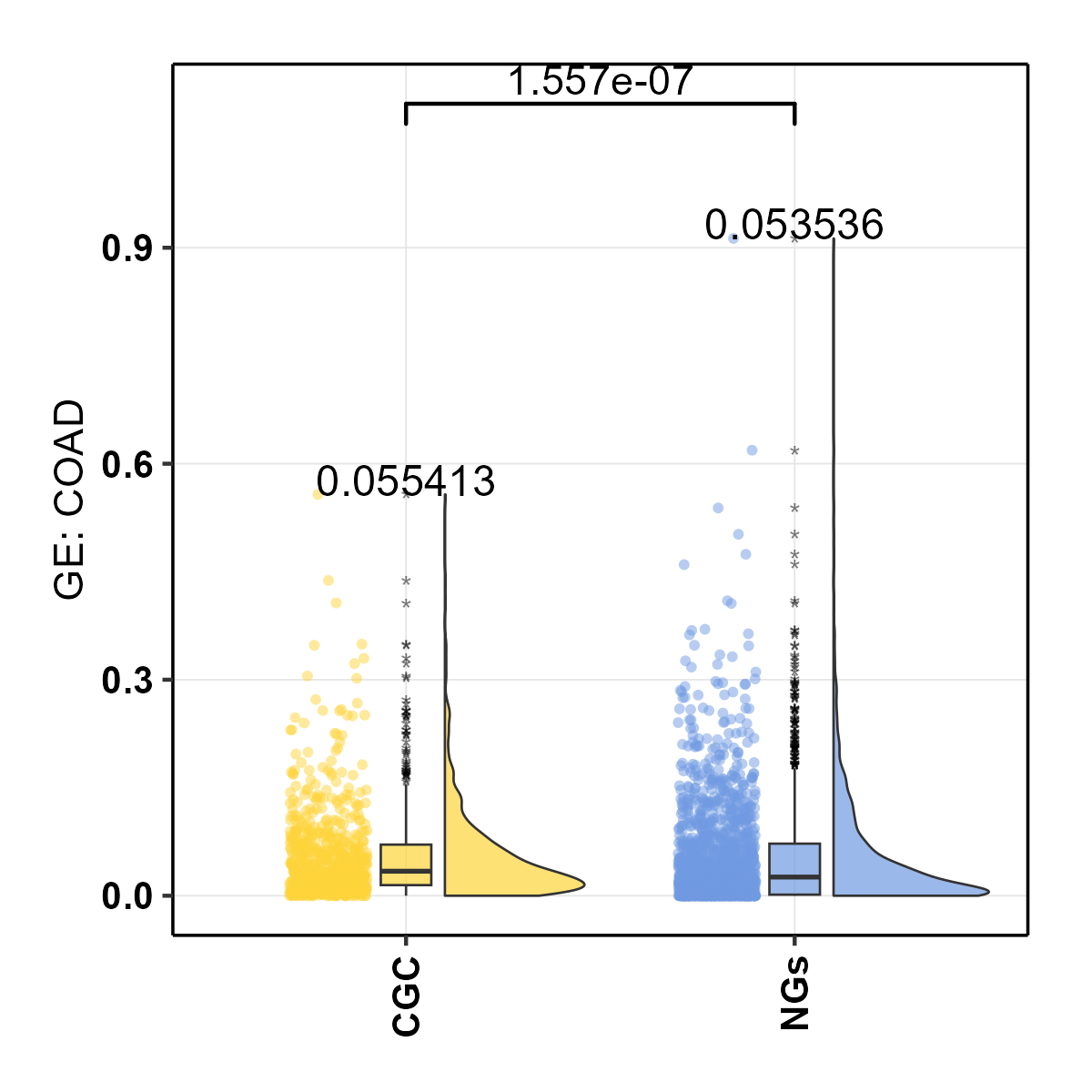

Supplement: Supplementary file 3 [file DataSheet1.ZIP › Supplementary file 5-1/Multinet/GE_COAD.png]

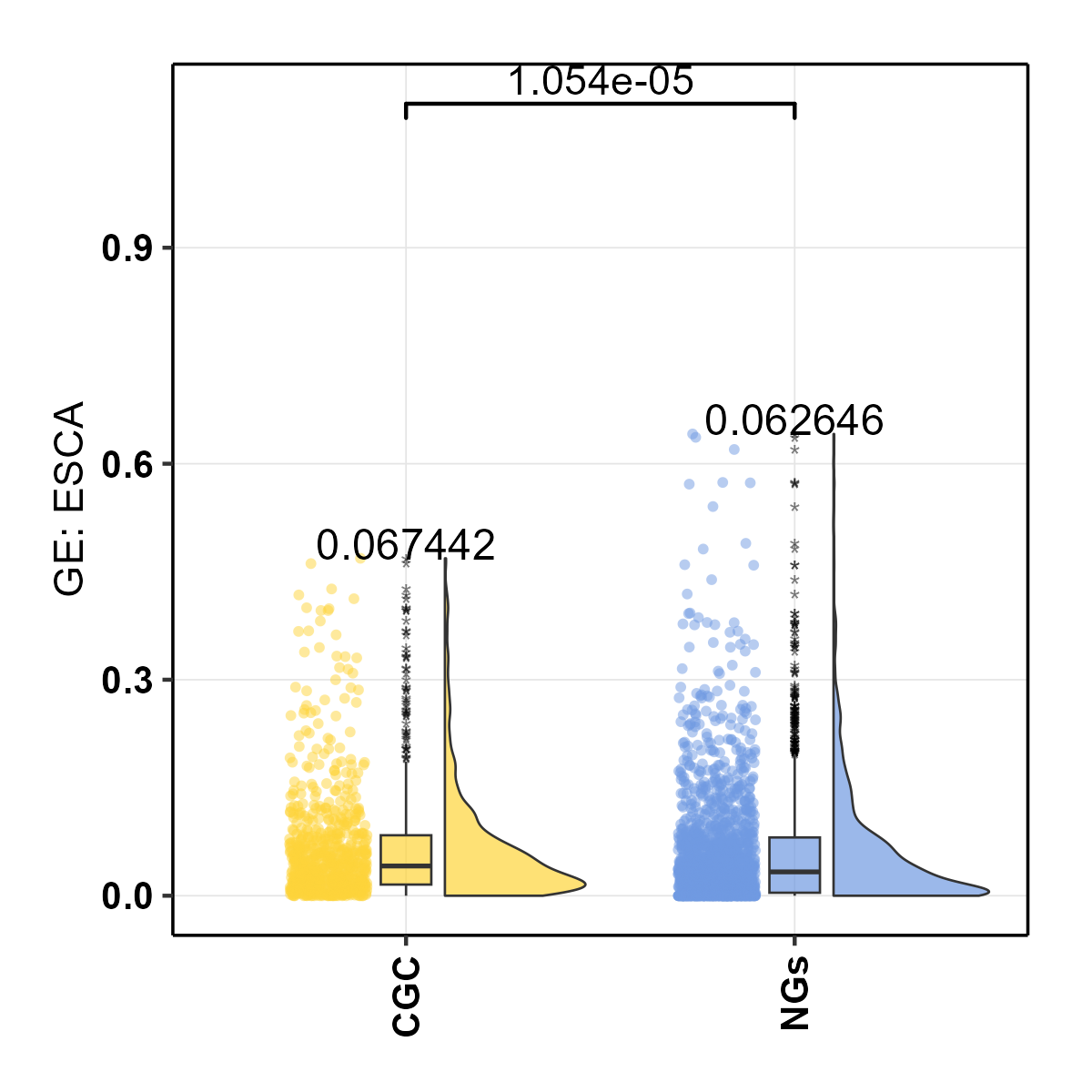

Supplement: Supplementary file 3 [file DataSheet1.ZIP › Supplementary file 5-1/Multinet/GE_ESCA.png]

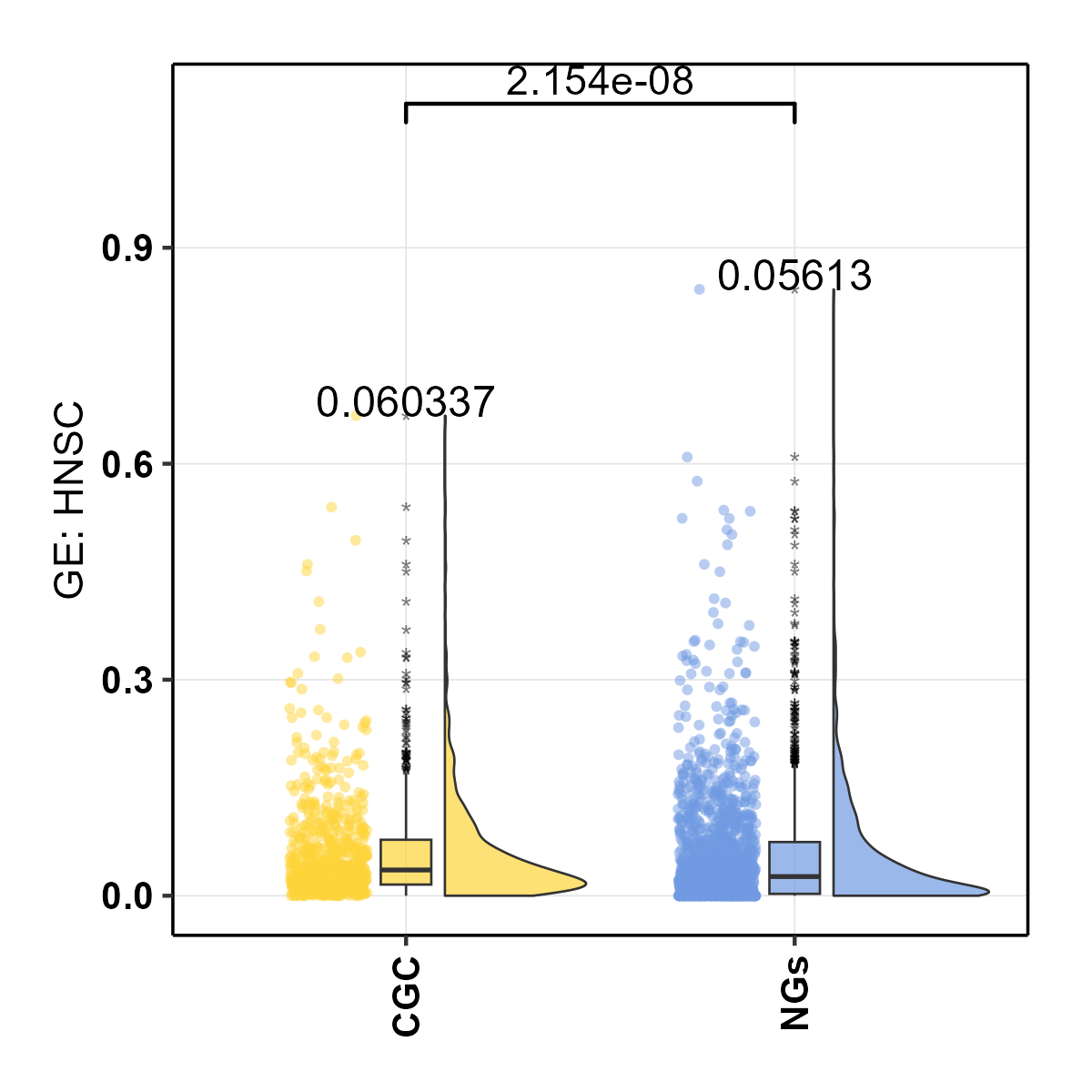

Supplement: Supplementary file 3 [file DataSheet1.ZIP › Supplementary file 5-1/Multinet/GE_HNSC.png]

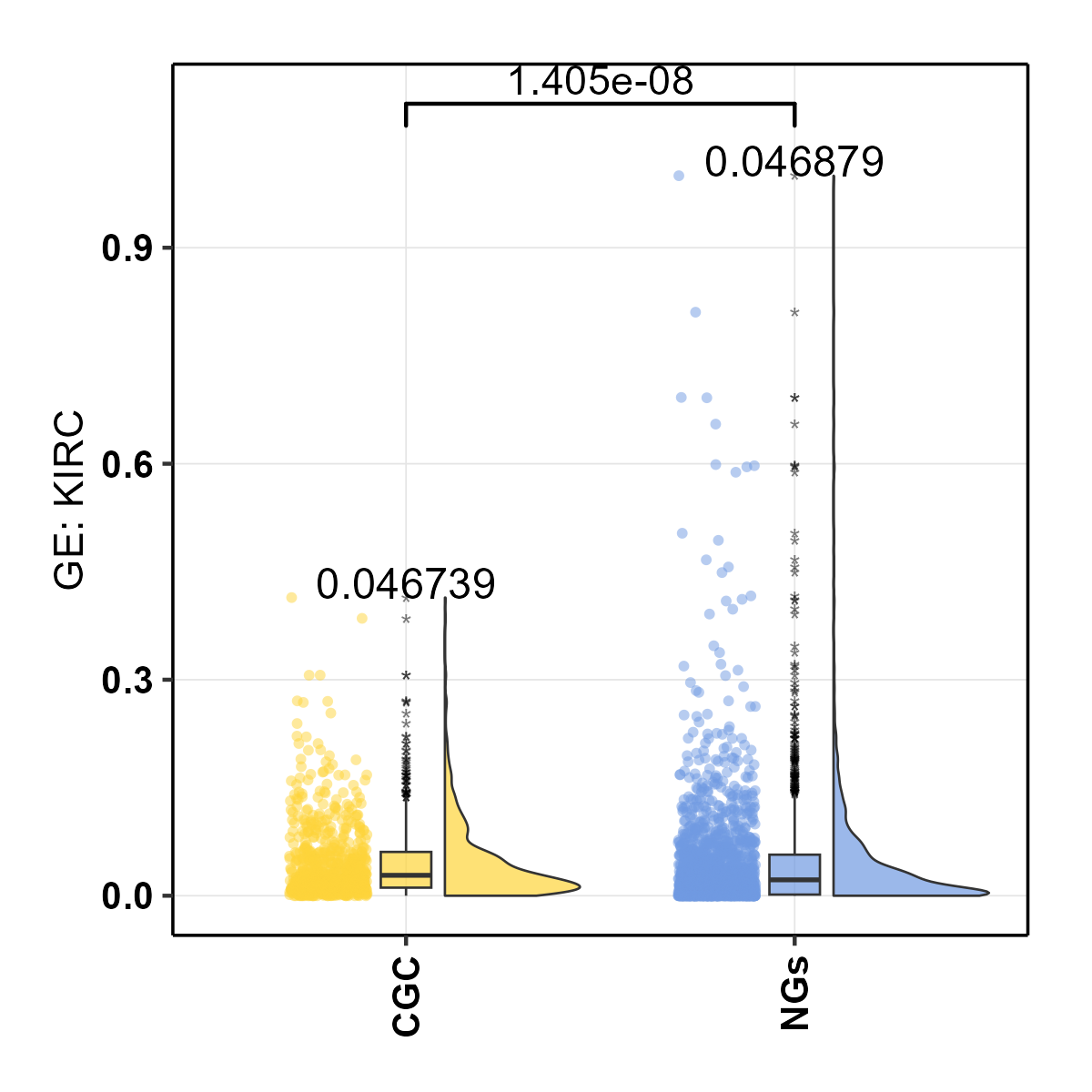

Supplement: Supplementary file 3 [file DataSheet1.ZIP › Supplementary file 5-1/Multinet/GE_KIRC.png]

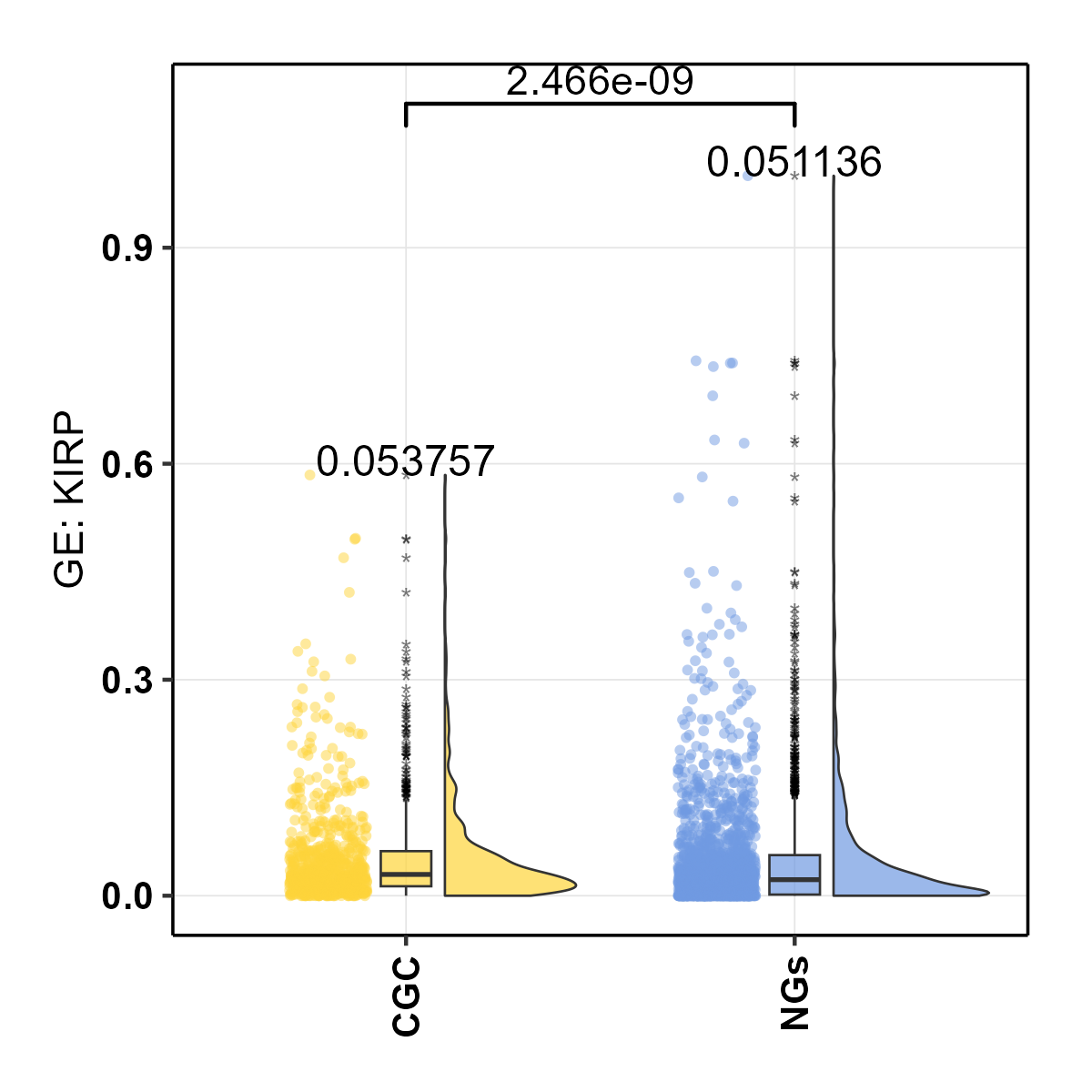

Supplement: Supplementary file 3 [file DataSheet1.ZIP › Supplementary file 5-1/Multinet/GE_KIRP.png]

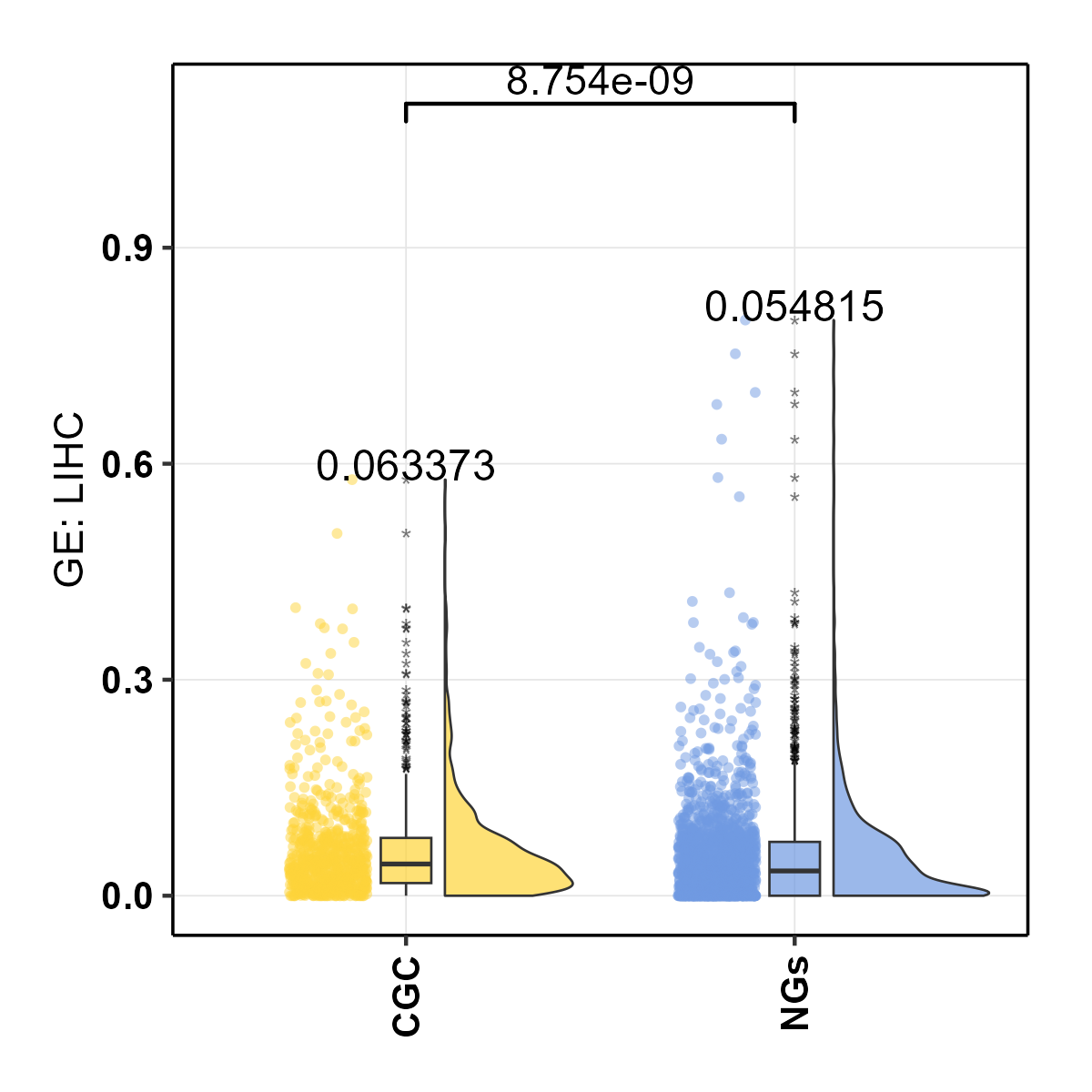

Supplement: Supplementary file 3 [file DataSheet1.ZIP › Supplementary file 5-1/Multinet/GE_LIHC.png]

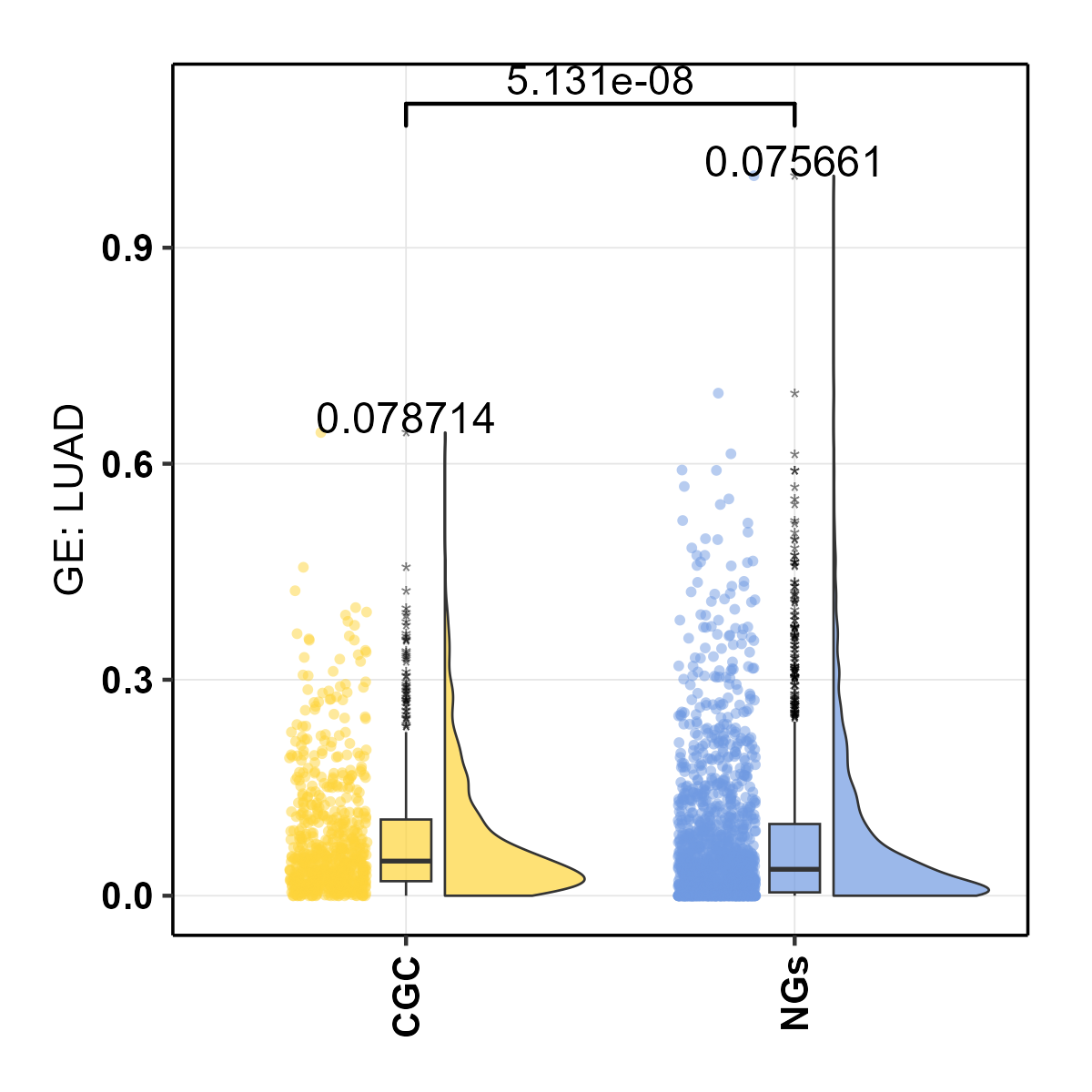

Supplement: Supplementary file 3 [file DataSheet1.ZIP › Supplementary file 5-1/Multinet/GE_LUAD.png]

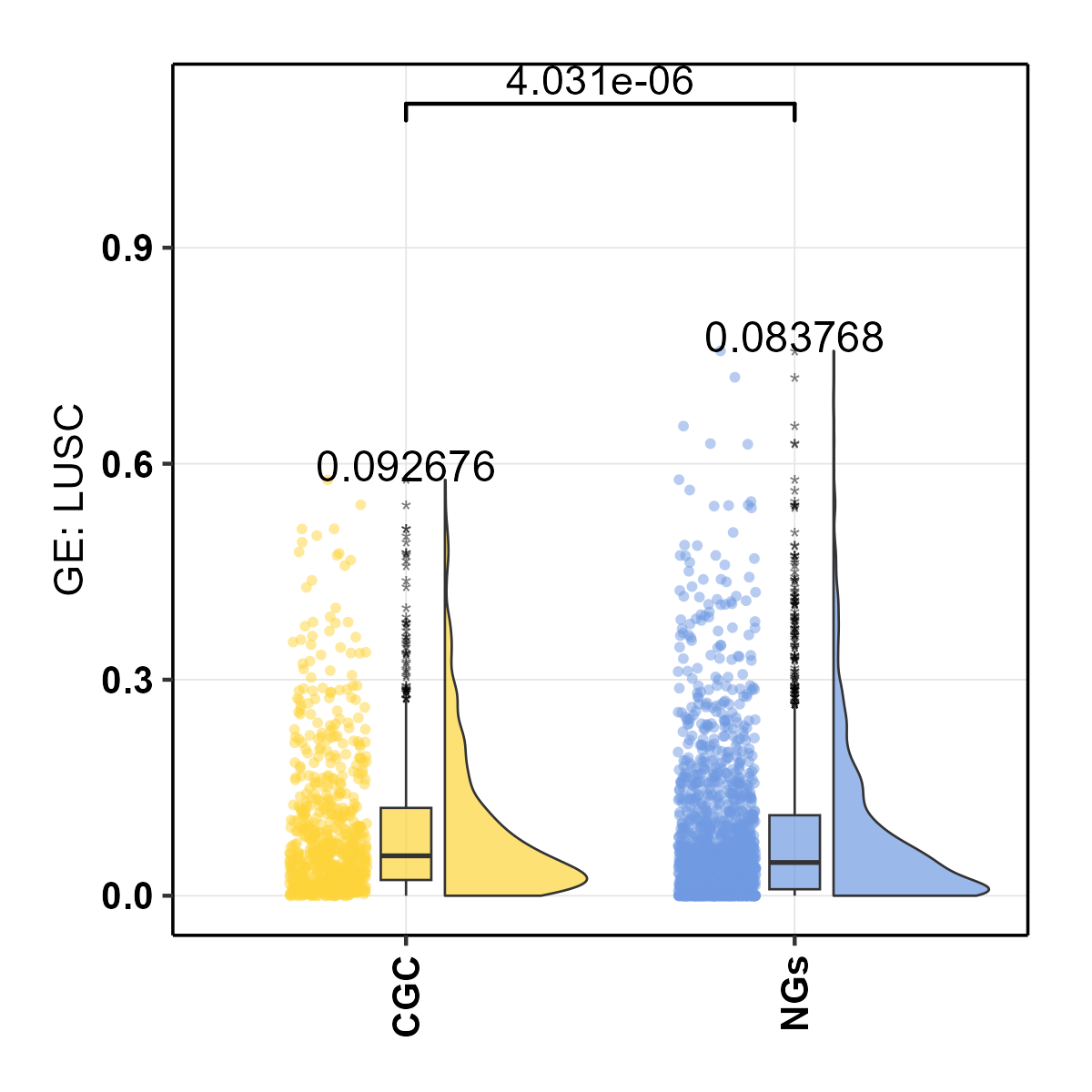

Supplement: Supplementary file 3 [file DataSheet1.ZIP › Supplementary file 5-1/Multinet/GE_LUSC.png]

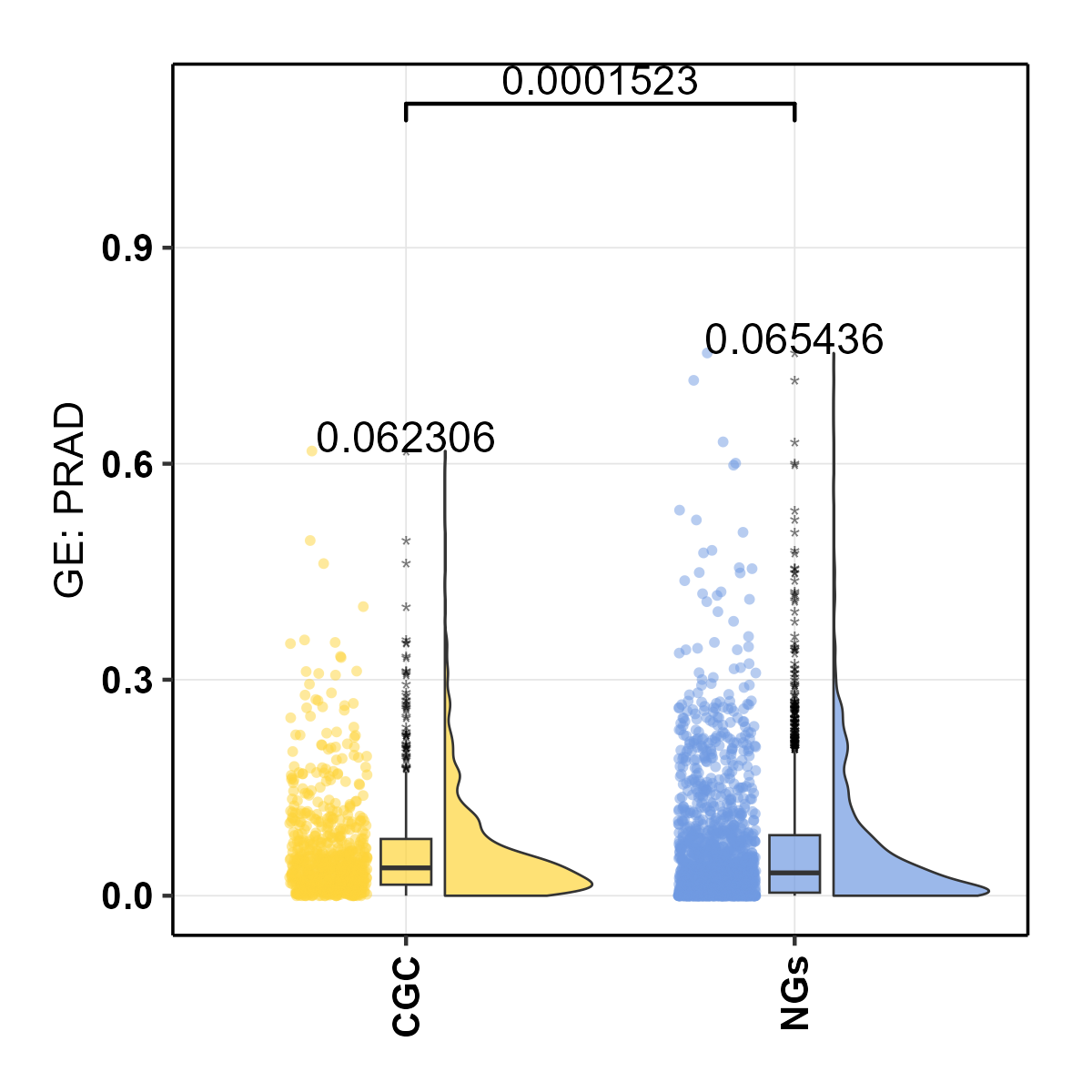

Supplement: Supplementary file 3 [file DataSheet1.ZIP › Supplementary file 5-1/Multinet/GE_PRAD.png]

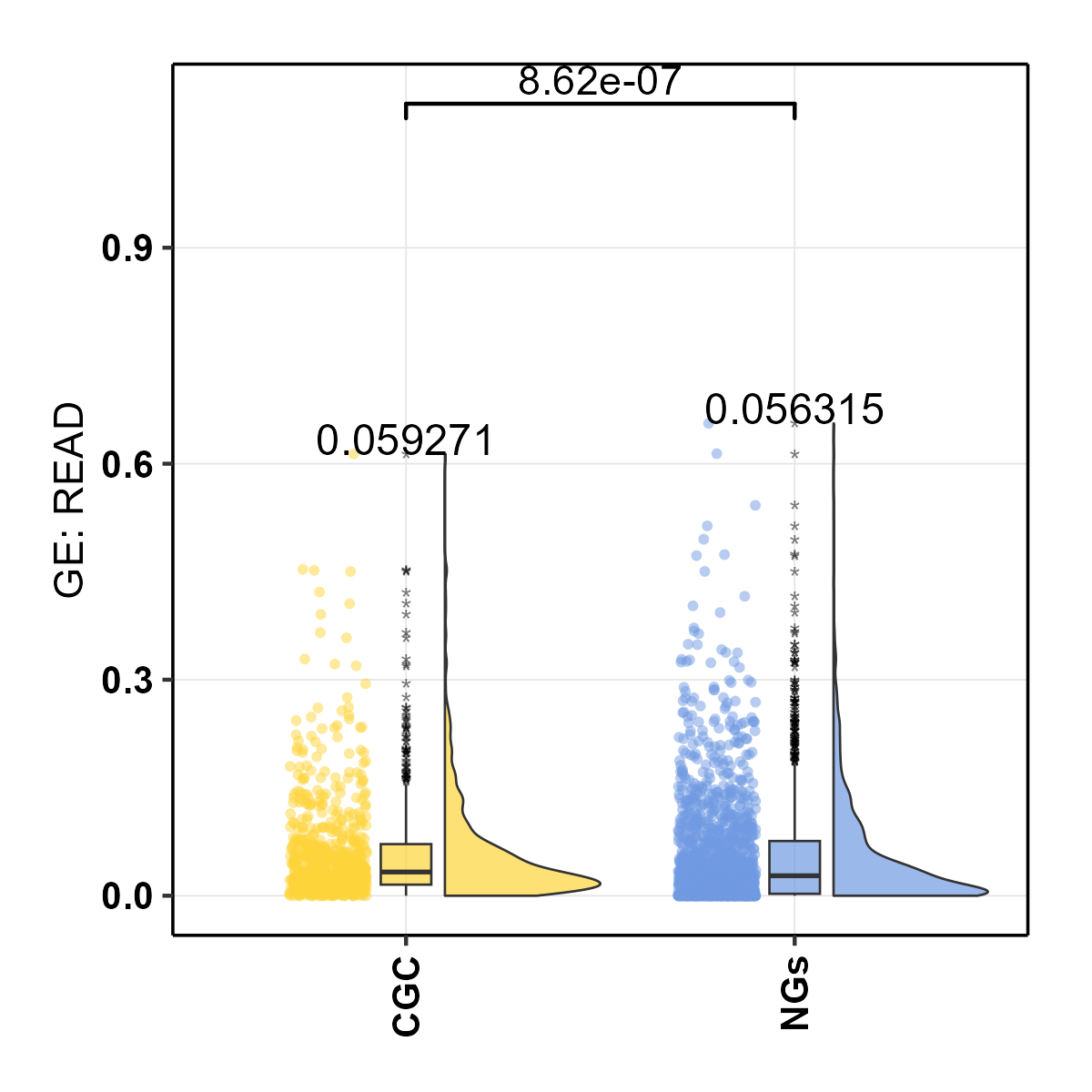

Supplement: Supplementary file 3 [file DataSheet1.ZIP › Supplementary file 5-1/Multinet/GE_READ.png]

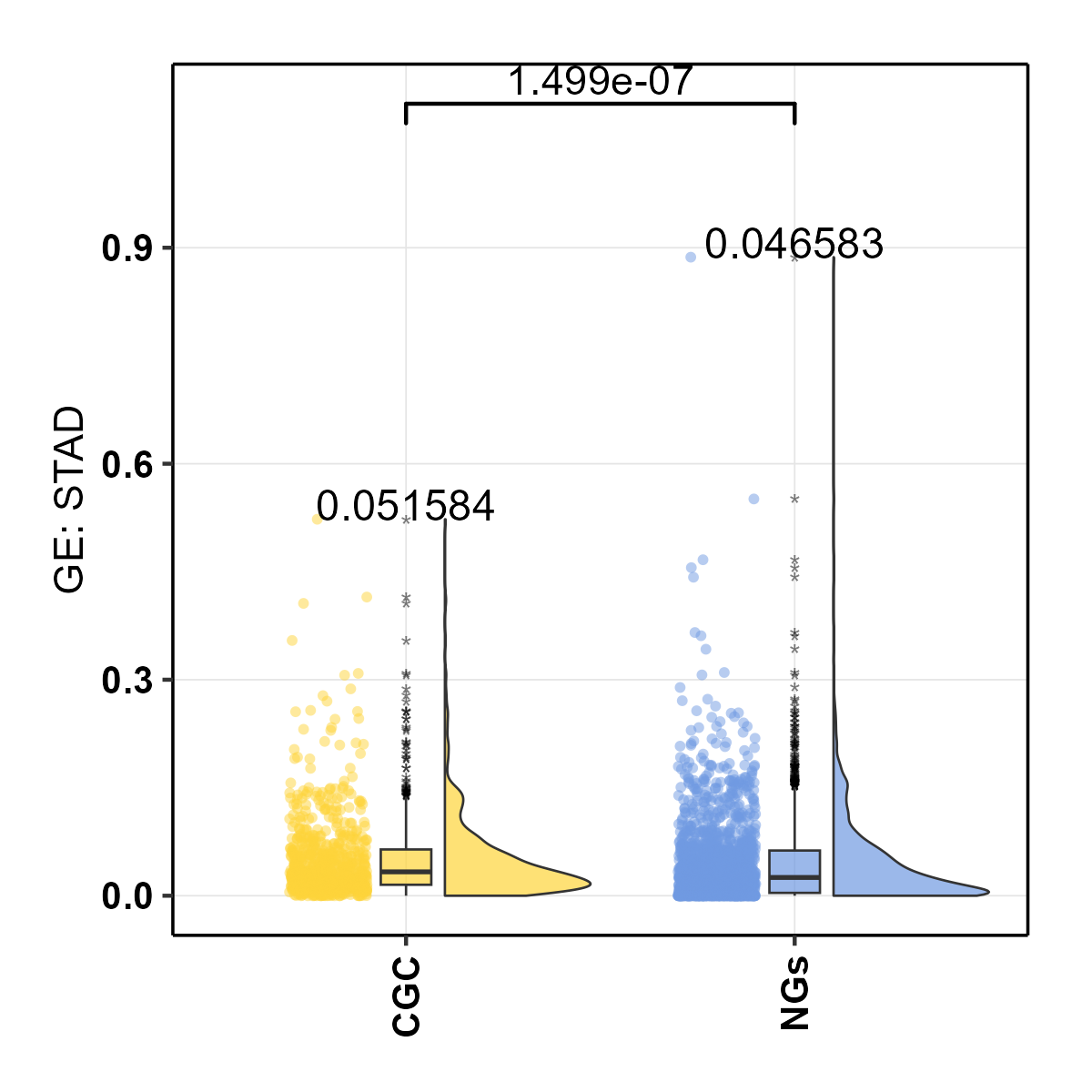

Supplement: Supplementary file 3 [file DataSheet1.ZIP › Supplementary file 5-1/Multinet/GE_STAD.png]

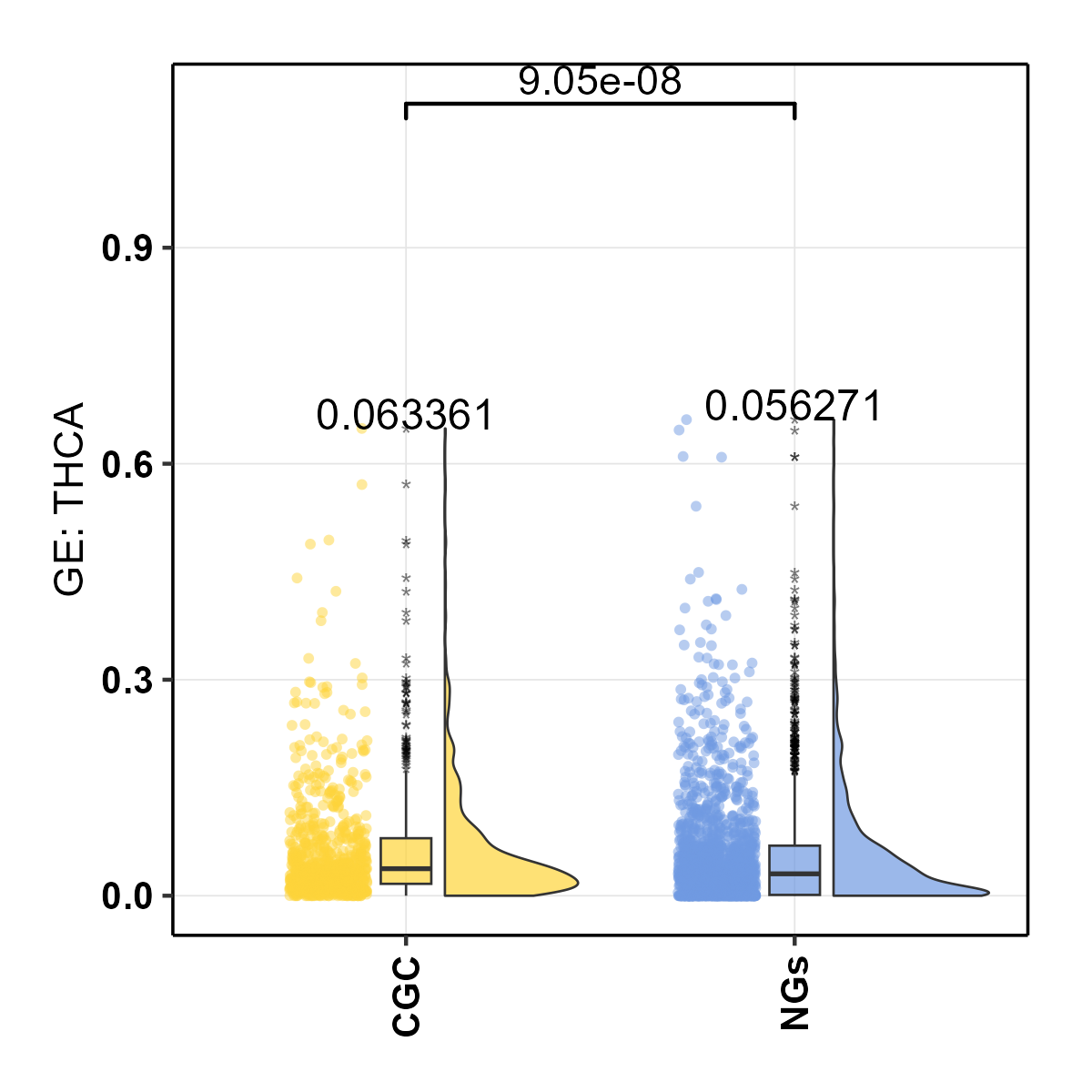

Supplement: Supplementary file 3 [file DataSheet1.ZIP › Supplementary file 5-1/Multinet/GE_THCA.png]

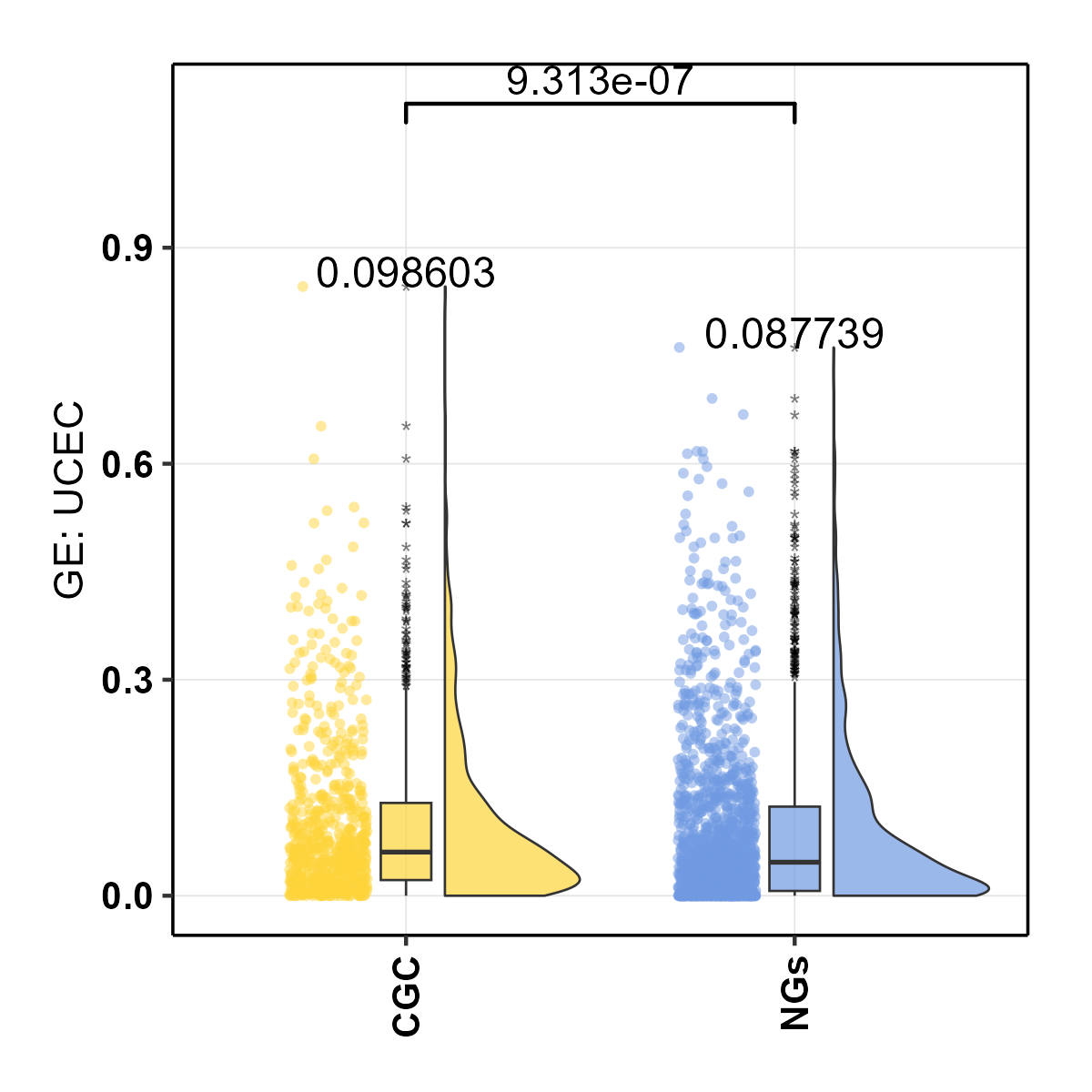

Supplement: Supplementary file 3 [file DataSheet1.ZIP › Supplementary file 5-1/Multinet/GE_UCEC.png]

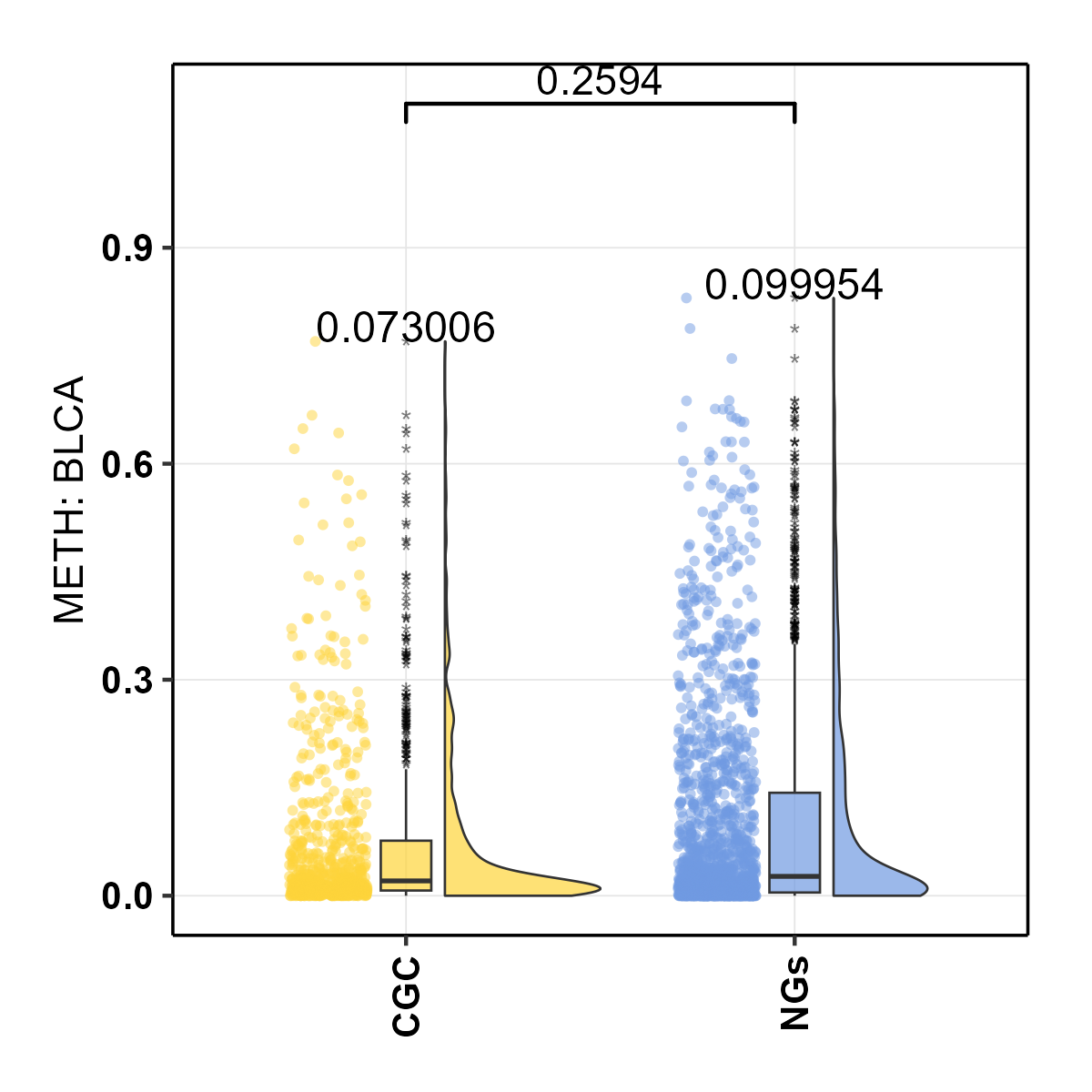

Supplement: Supplementary file 3 [file DataSheet1.ZIP › Supplementary file 5-1/Multinet/METH_BLCA.png]

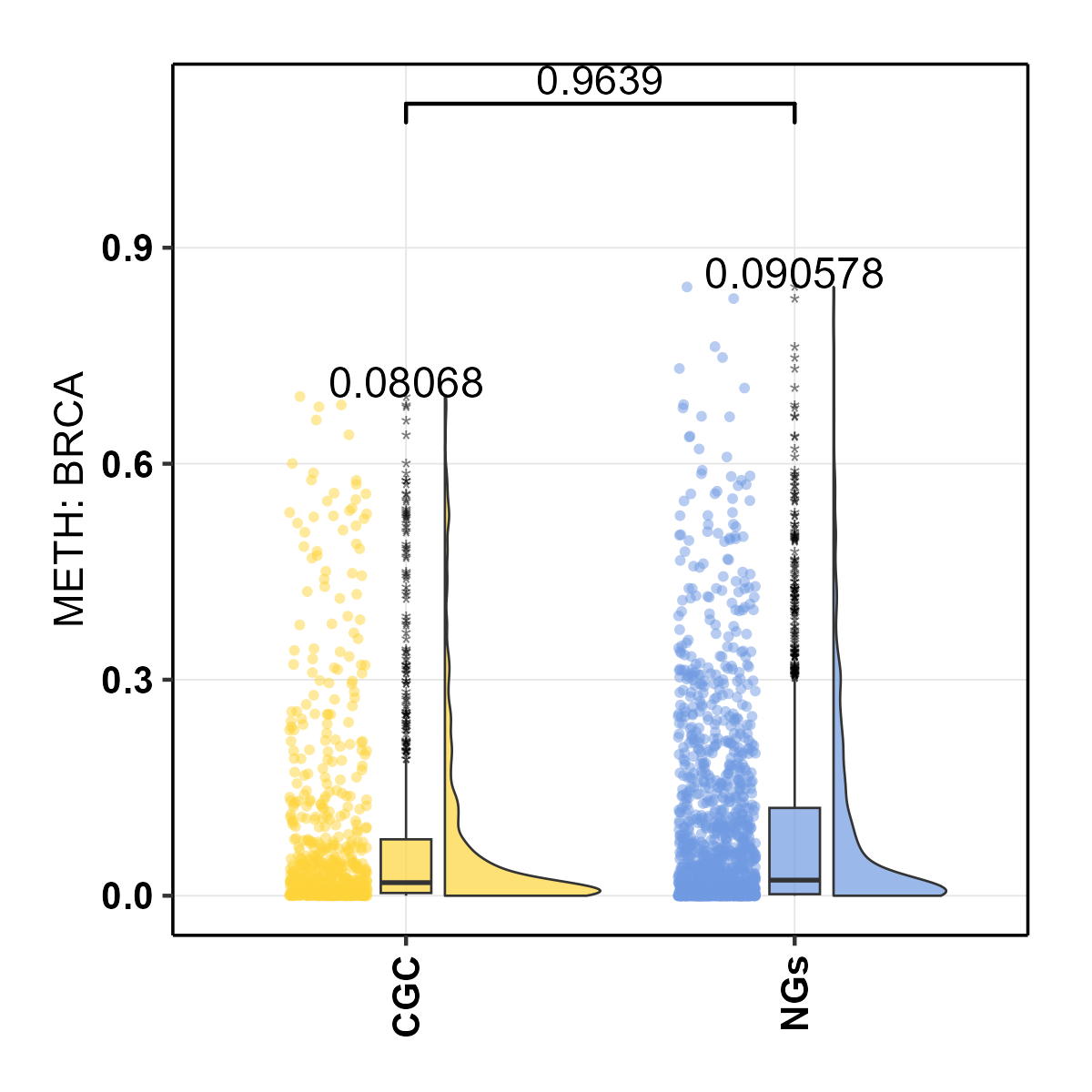

Supplement: Supplementary file 3 [file DataSheet1.ZIP › Supplementary file 5-1/Multinet/METH_BRCA.png]

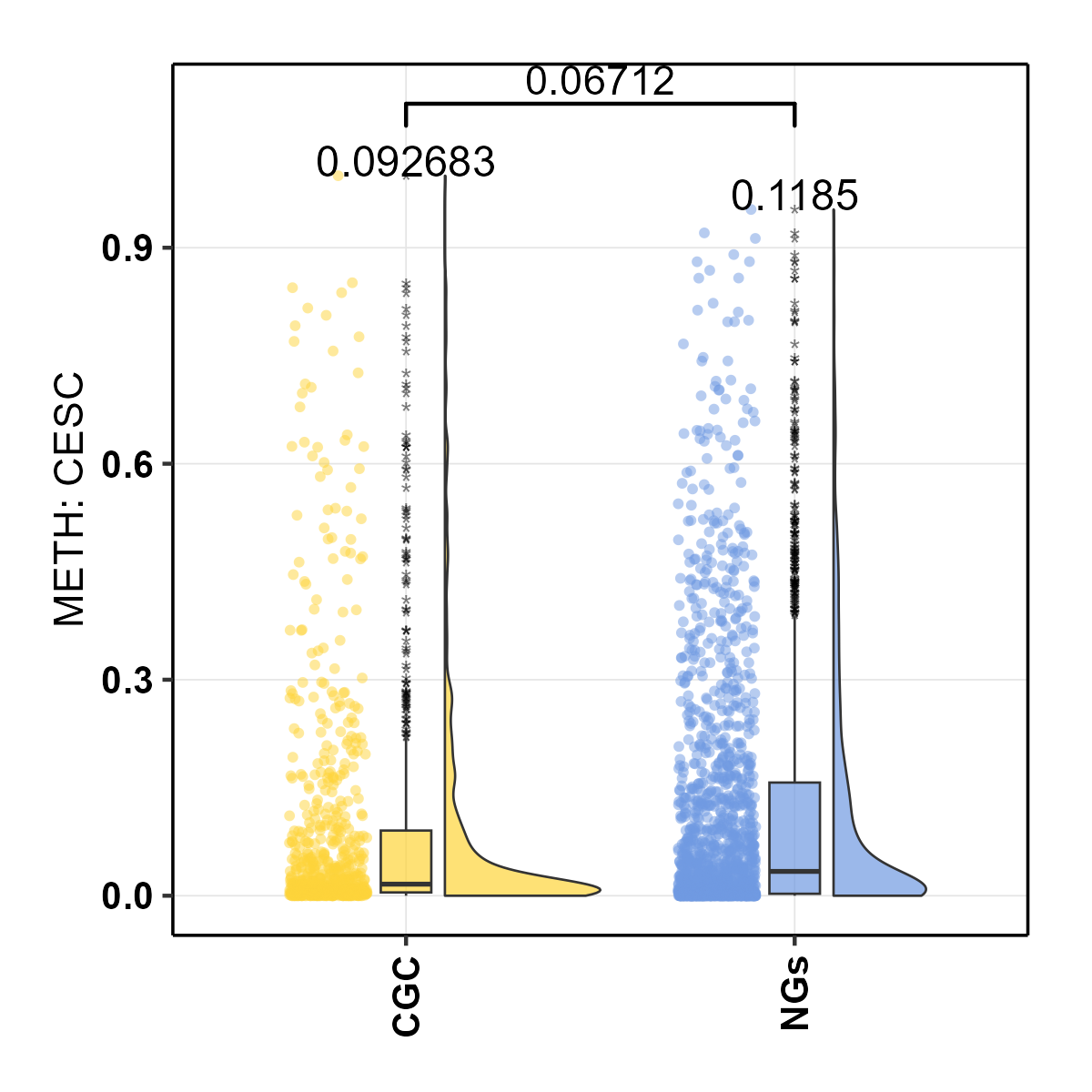

Supplement: Supplementary file 3 [file DataSheet1.ZIP › Supplementary file 5-1/Multinet/METH_CESC.png]

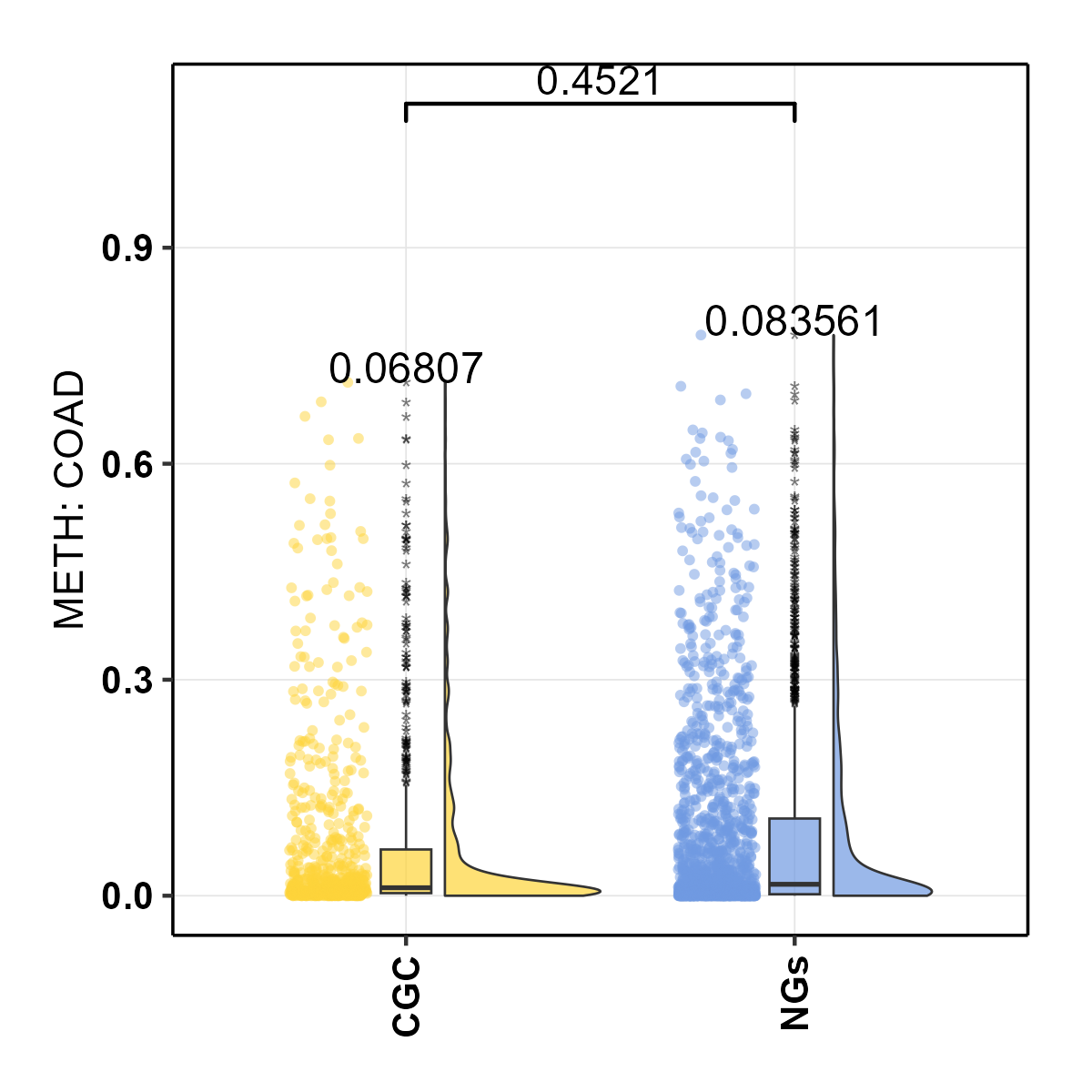

Supplement: Supplementary file 3 [file DataSheet1.ZIP › Supplementary file 5-1/Multinet/METH_COAD.png]

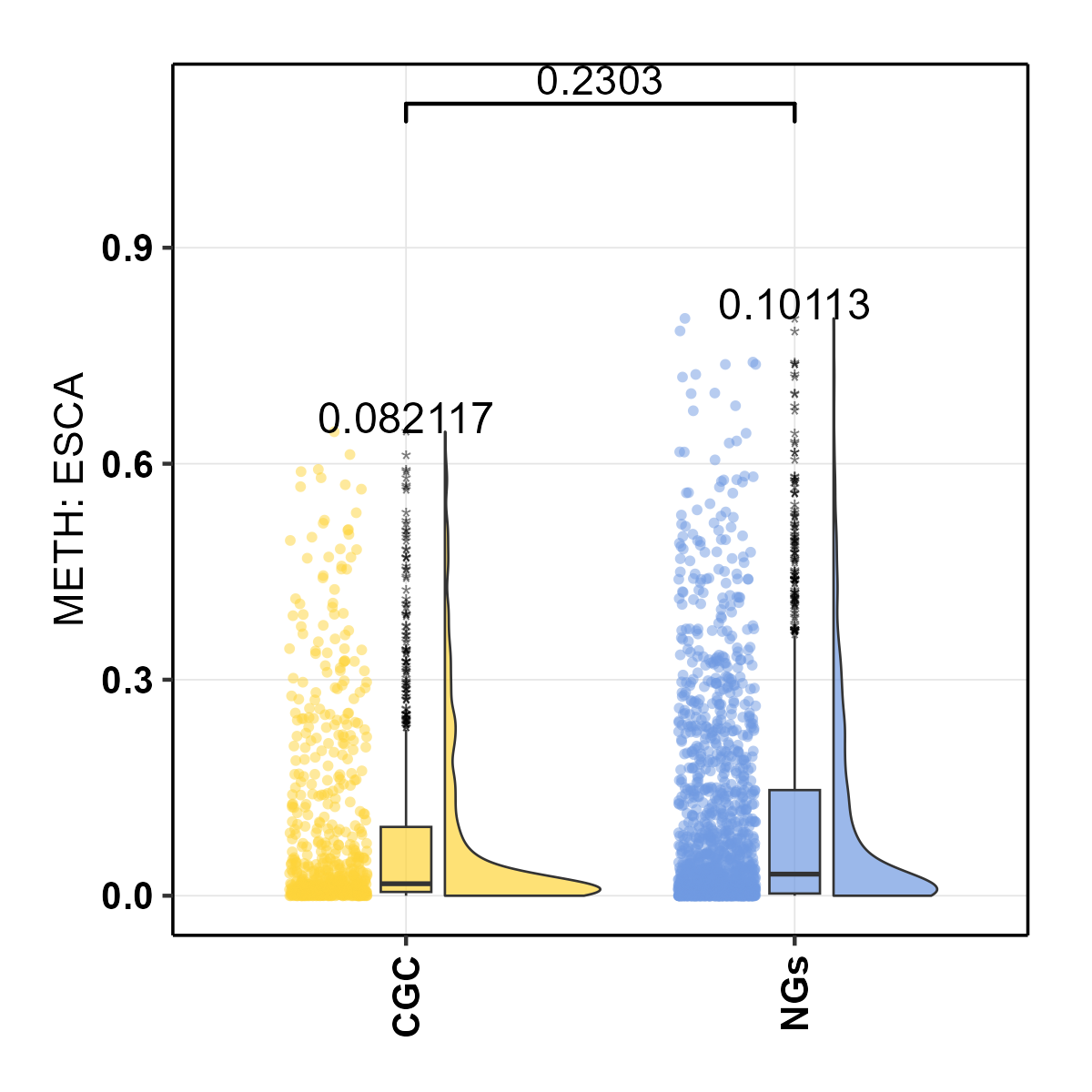

Supplement: Supplementary file 3 [file DataSheet1.ZIP › Supplementary file 5-1/Multinet/METH_ESCA.png]

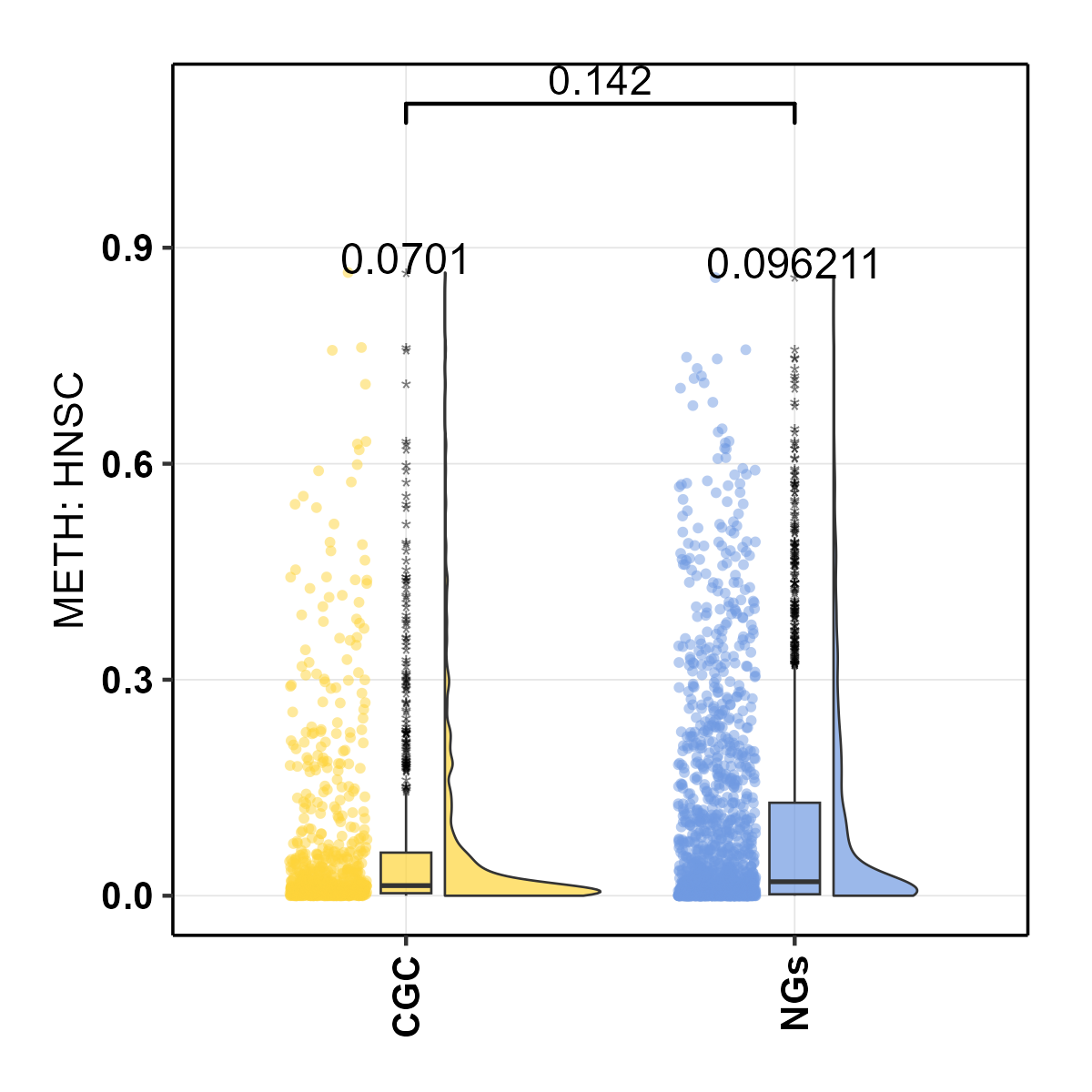

Supplement: Supplementary file 3 [file DataSheet1.ZIP › Supplementary file 5-1/Multinet/METH_HNSC.png]

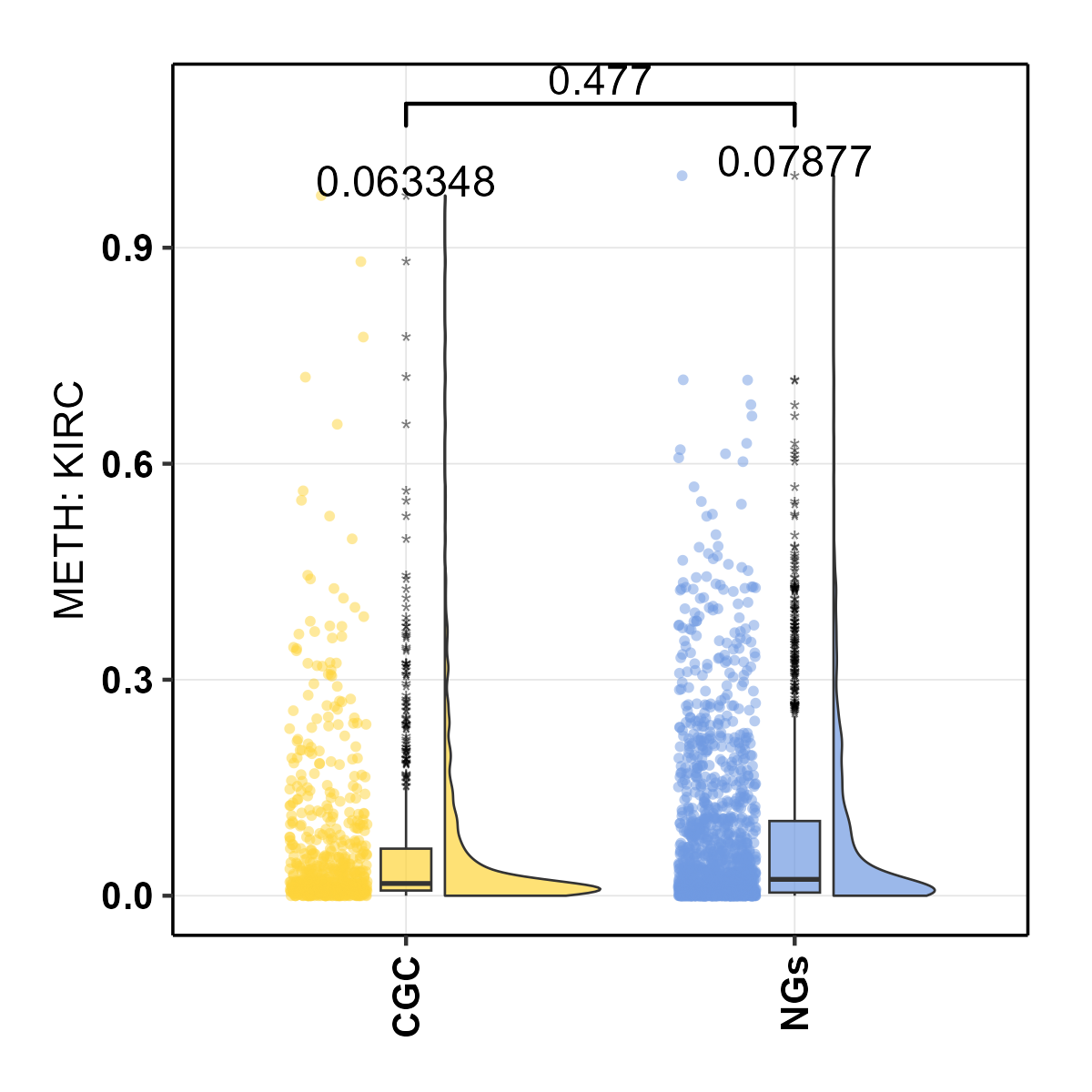

Supplement: Supplementary file 3 [file DataSheet1.ZIP › Supplementary file 5-1/Multinet/METH_KIRC.png]

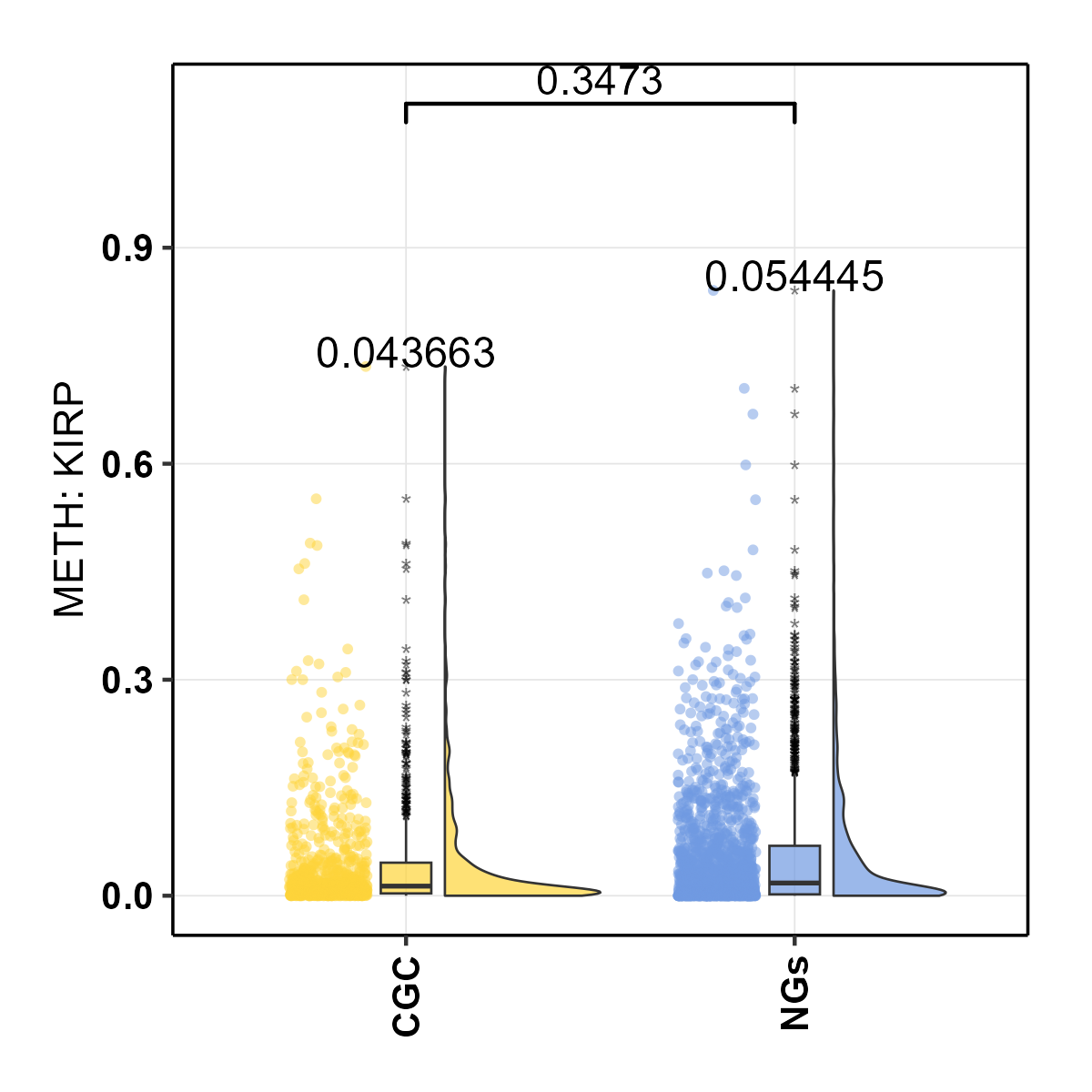

Supplement: Supplementary file 3 [file DataSheet1.ZIP › Supplementary file 5-1/Multinet/METH_KIRP.png]

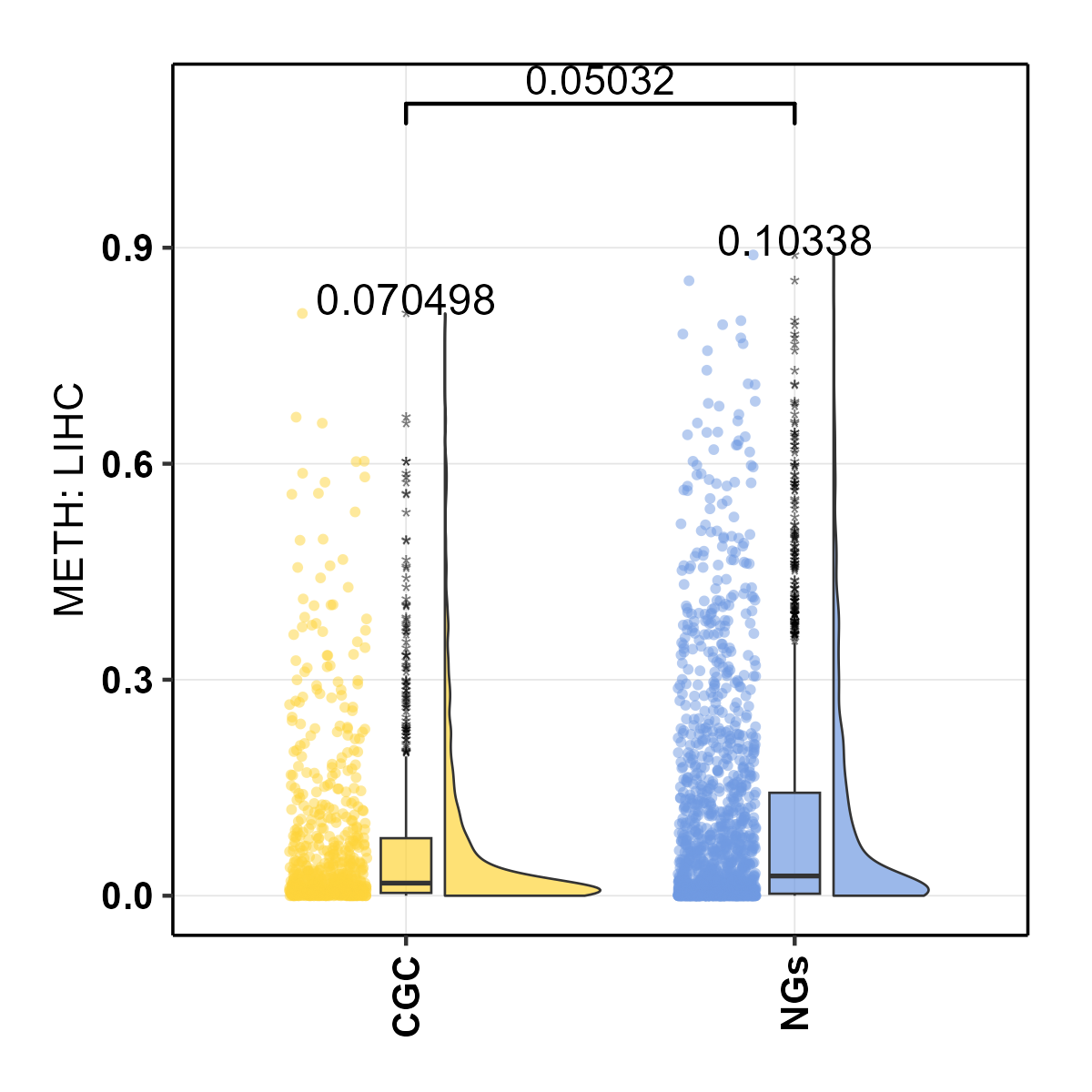

Supplement: Supplementary file 3 [file DataSheet1.ZIP › Supplementary file 5-1/Multinet/METH_LIHC.png]

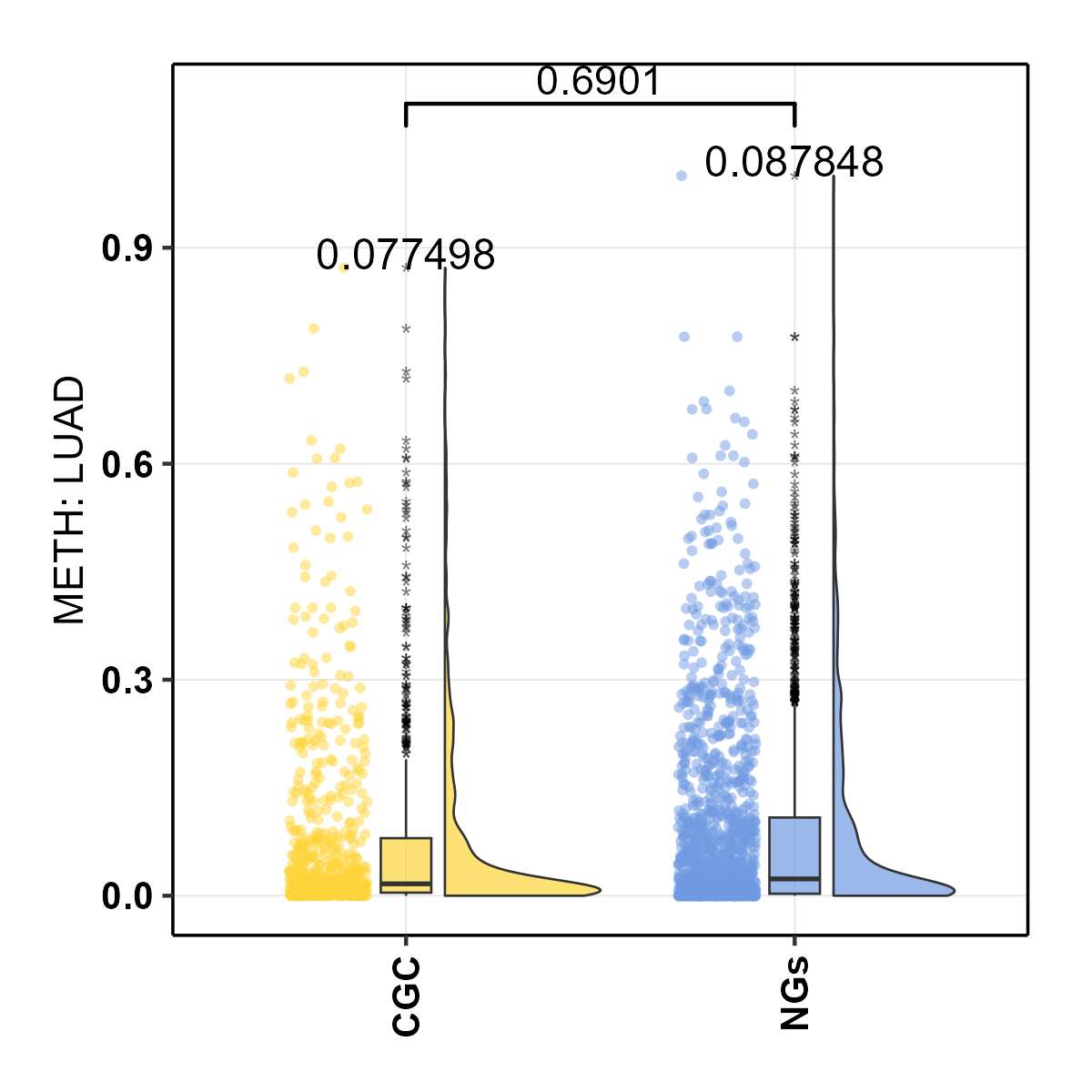

Supplement: Supplementary file 3 [file DataSheet1.ZIP › Supplementary file 5-1/Multinet/METH_LUAD.png]

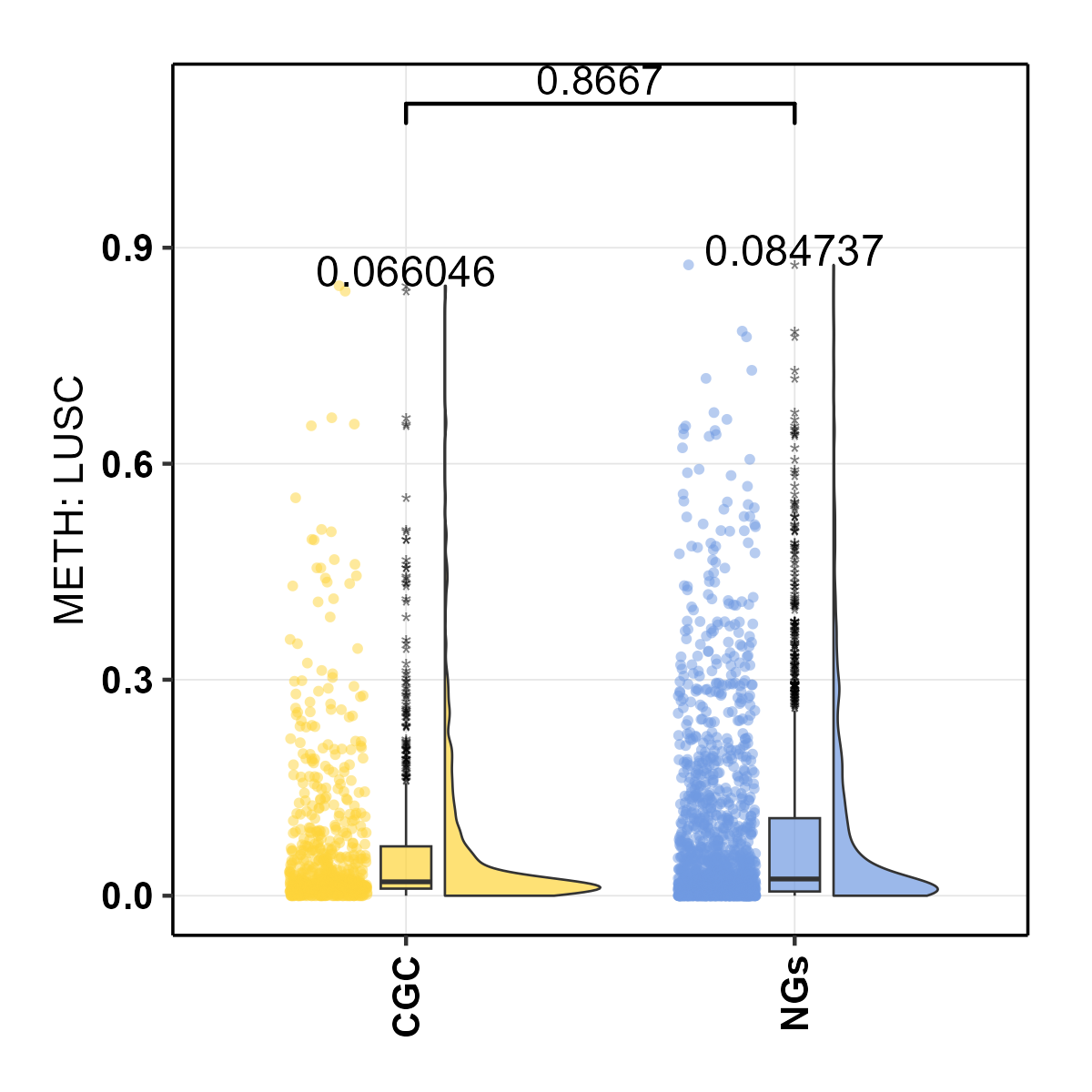

Supplement: Supplementary file 3 [file DataSheet1.ZIP › Supplementary file 5-1/Multinet/METH_LUSC.png]

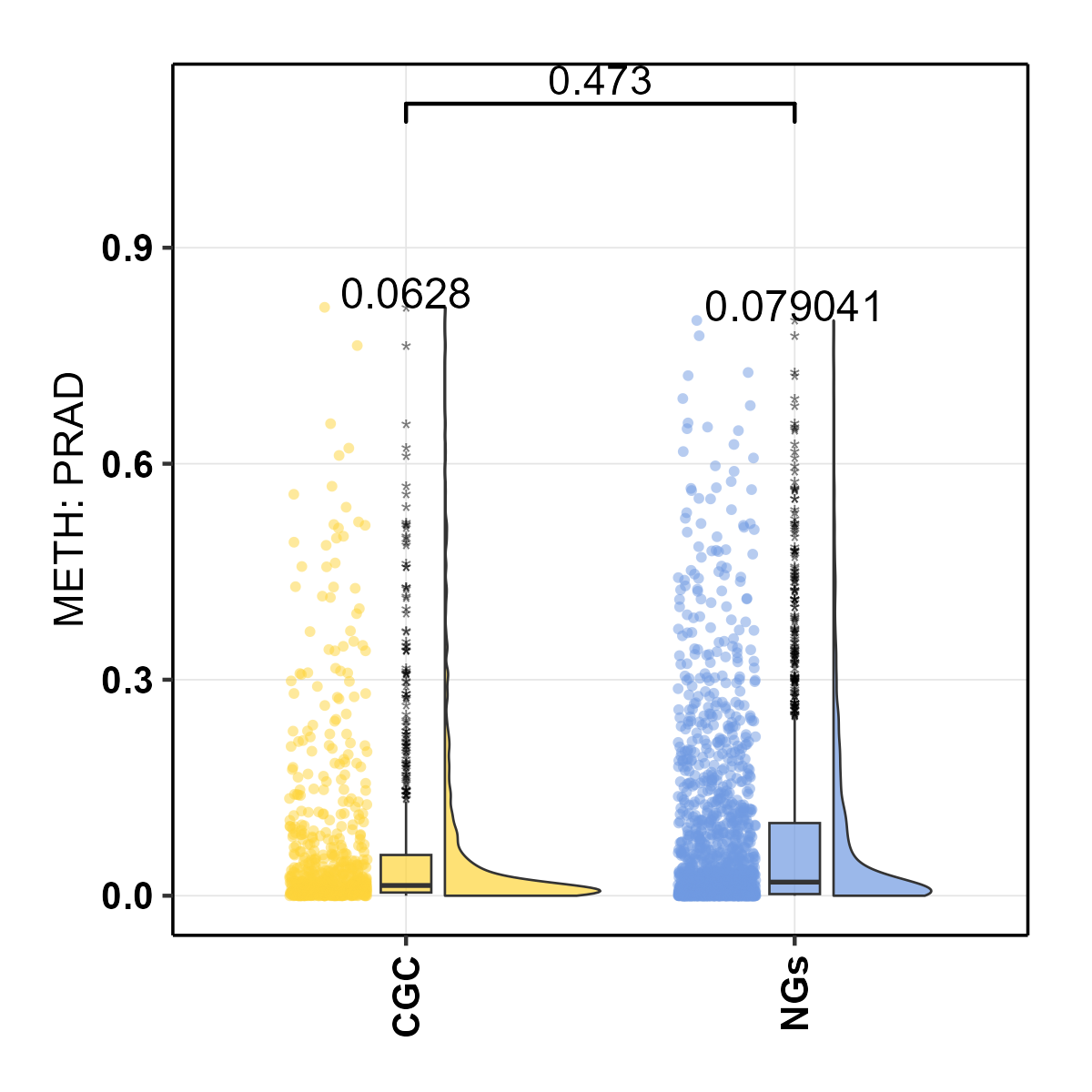

Supplement: Supplementary file 3 [file DataSheet1.ZIP › Supplementary file 5-1/Multinet/METH_PRAD.png]

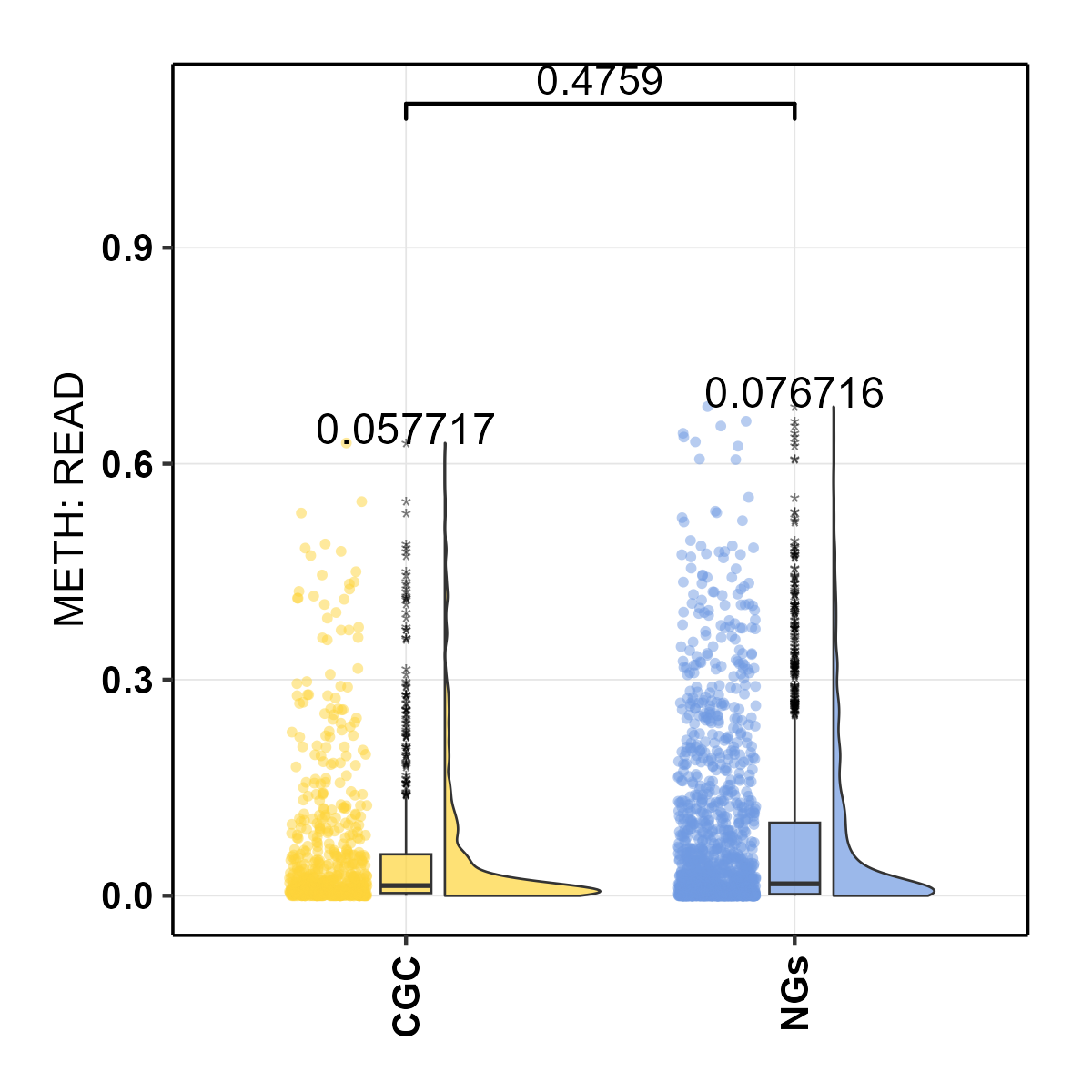

Supplement: Supplementary file 3 [file DataSheet1.ZIP › Supplementary file 5-1/Multinet/METH_READ.png]

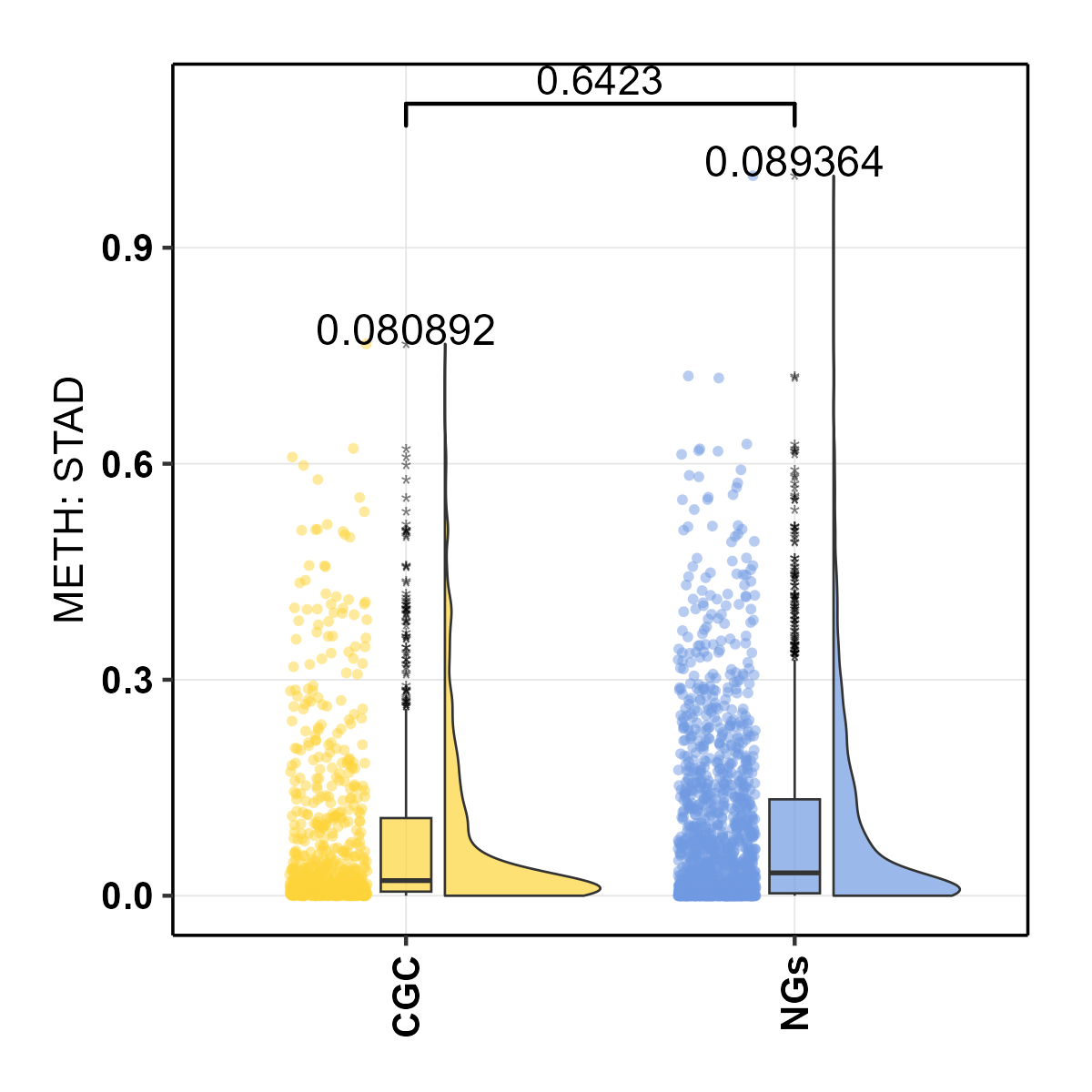

Supplement: Supplementary file 3 [file DataSheet1.ZIP › Supplementary file 5-1/Multinet/METH_STAD.png]

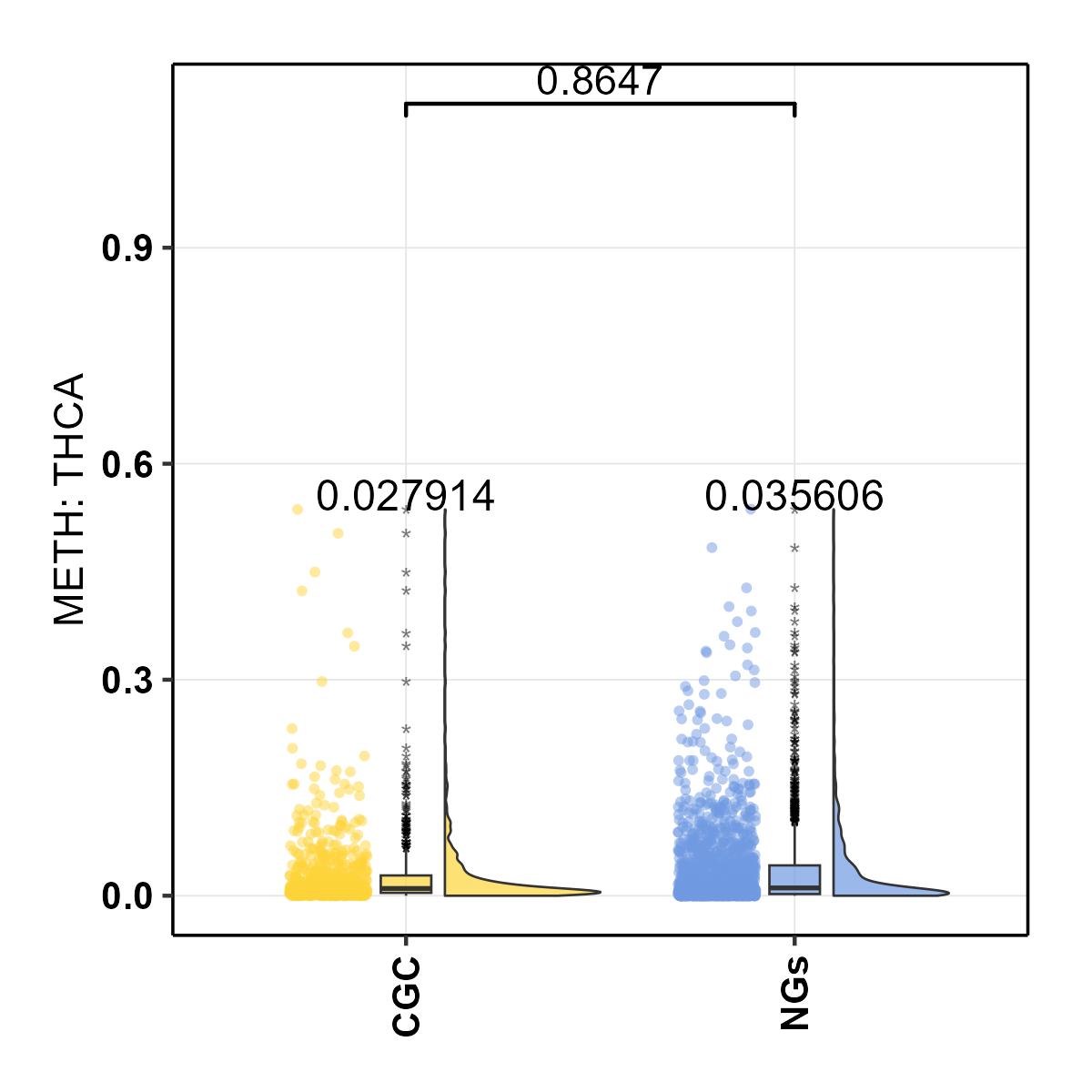

Supplement: Supplementary file 3 [file DataSheet1.ZIP › Supplementary file 5-1/Multinet/METH_THCA.png]

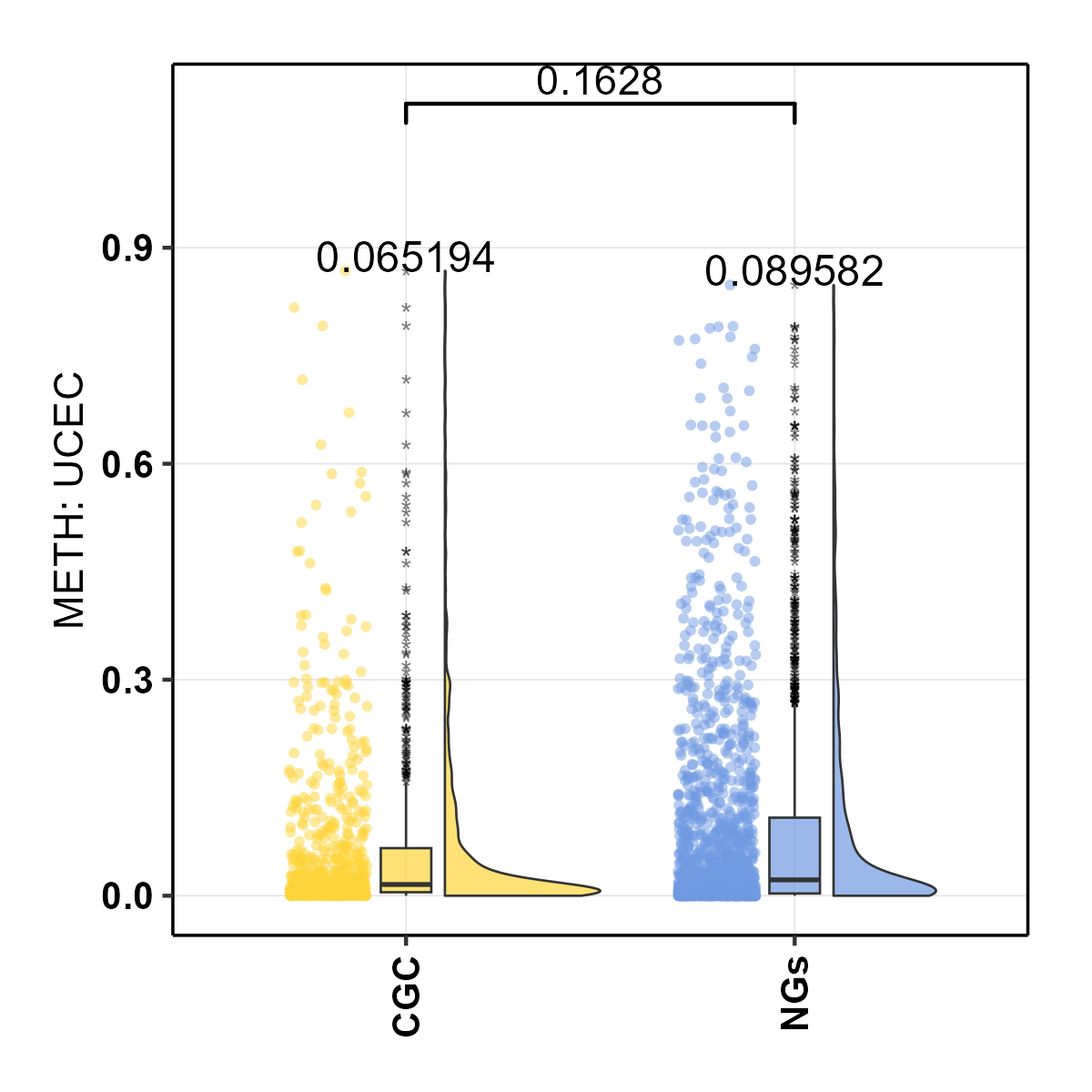

Supplement: Supplementary file 3 [file DataSheet1.ZIP › Supplementary file 5-1/Multinet/METH_UCEC.png]

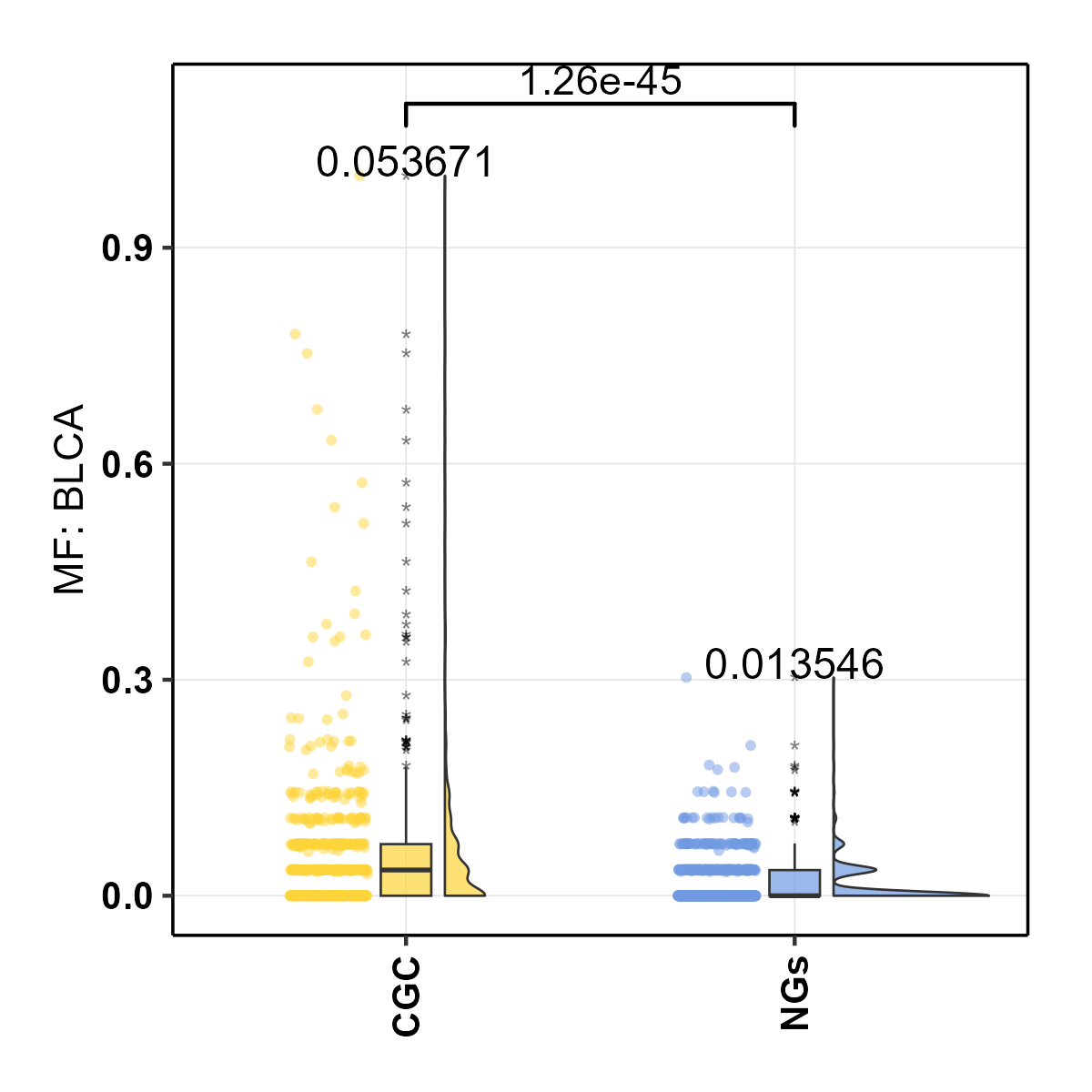

Supplement: Supplementary file 3 [file DataSheet1.ZIP › Supplementary file 5-1/Multinet/MF_BLCA.png]

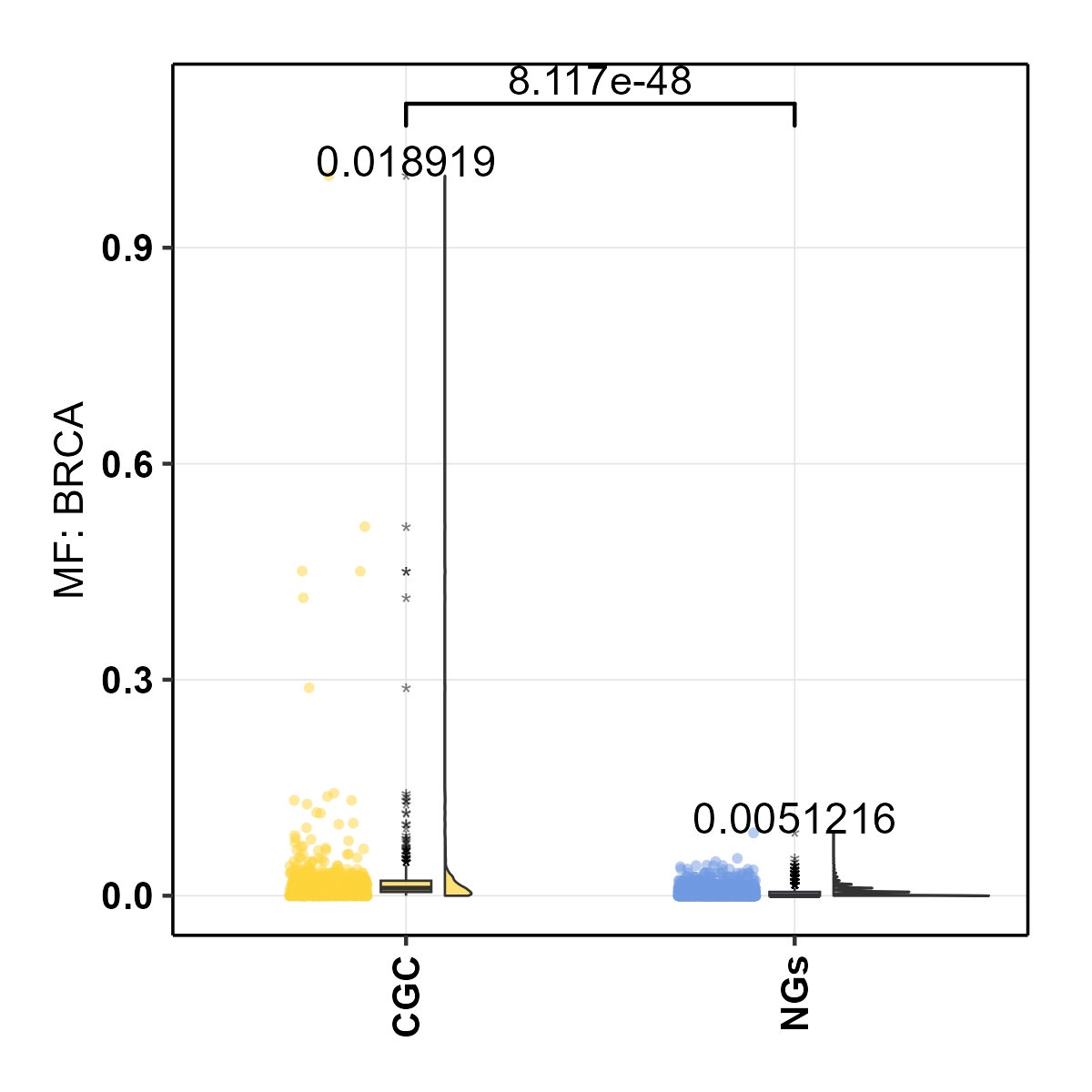

Supplement: Supplementary file 3 [file DataSheet1.ZIP › Supplementary file 5-1/Multinet/MF_BRCA.png]

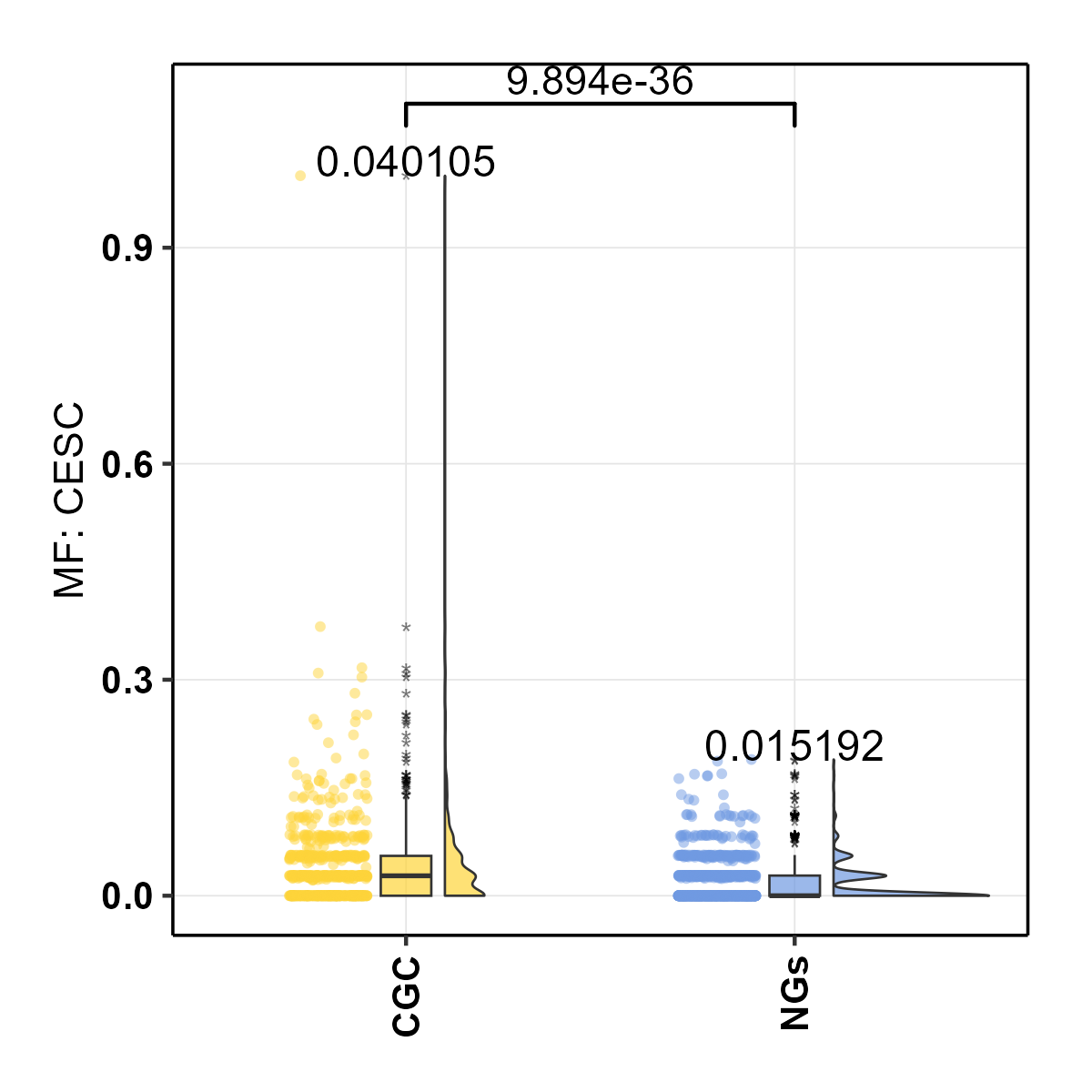

Supplement: Supplementary file 3 [file DataSheet1.ZIP › Supplementary file 5-1/Multinet/MF_CESC.png]

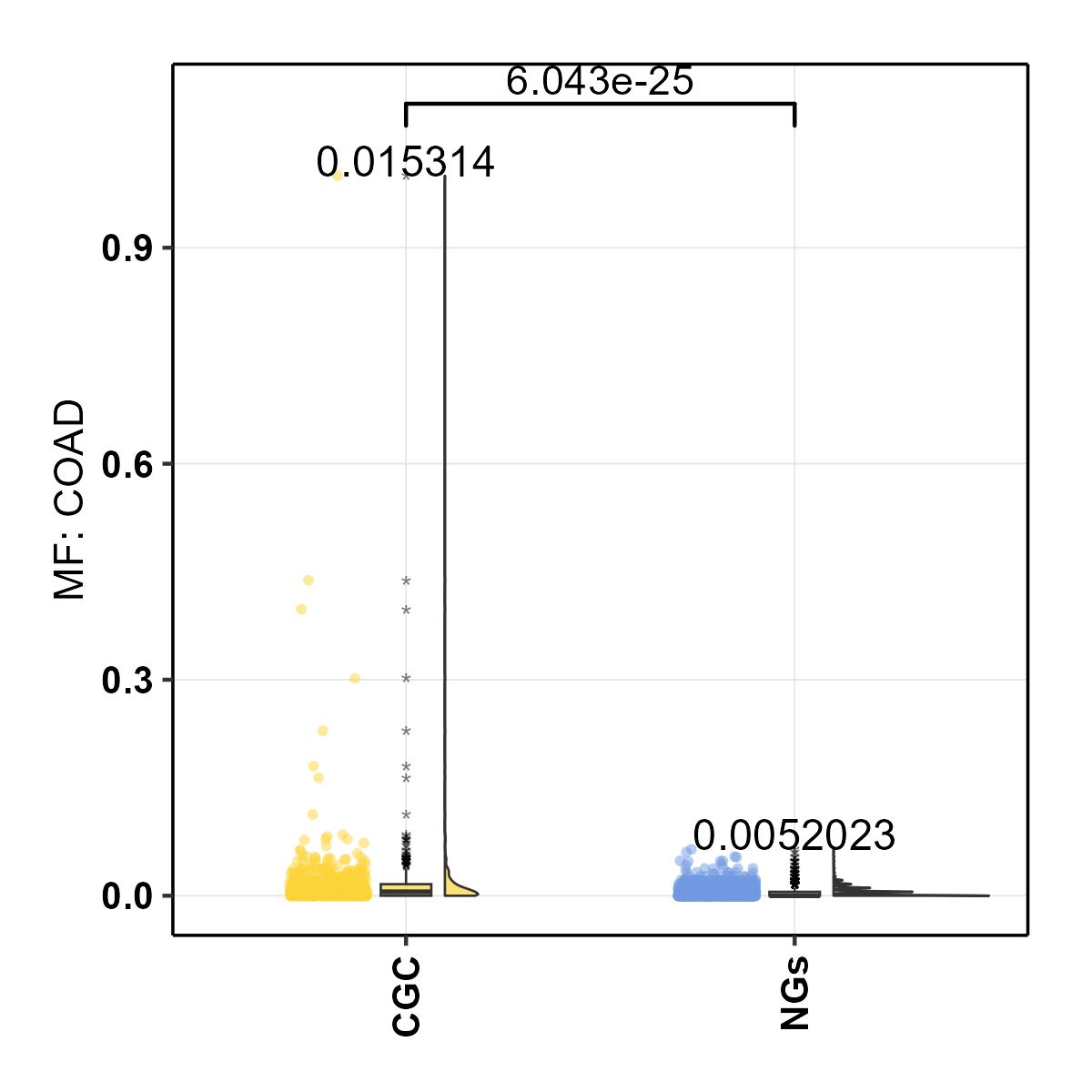

Supplement: Supplementary file 3 [file DataSheet1.ZIP › Supplementary file 5-1/Multinet/MF_COAD.png]

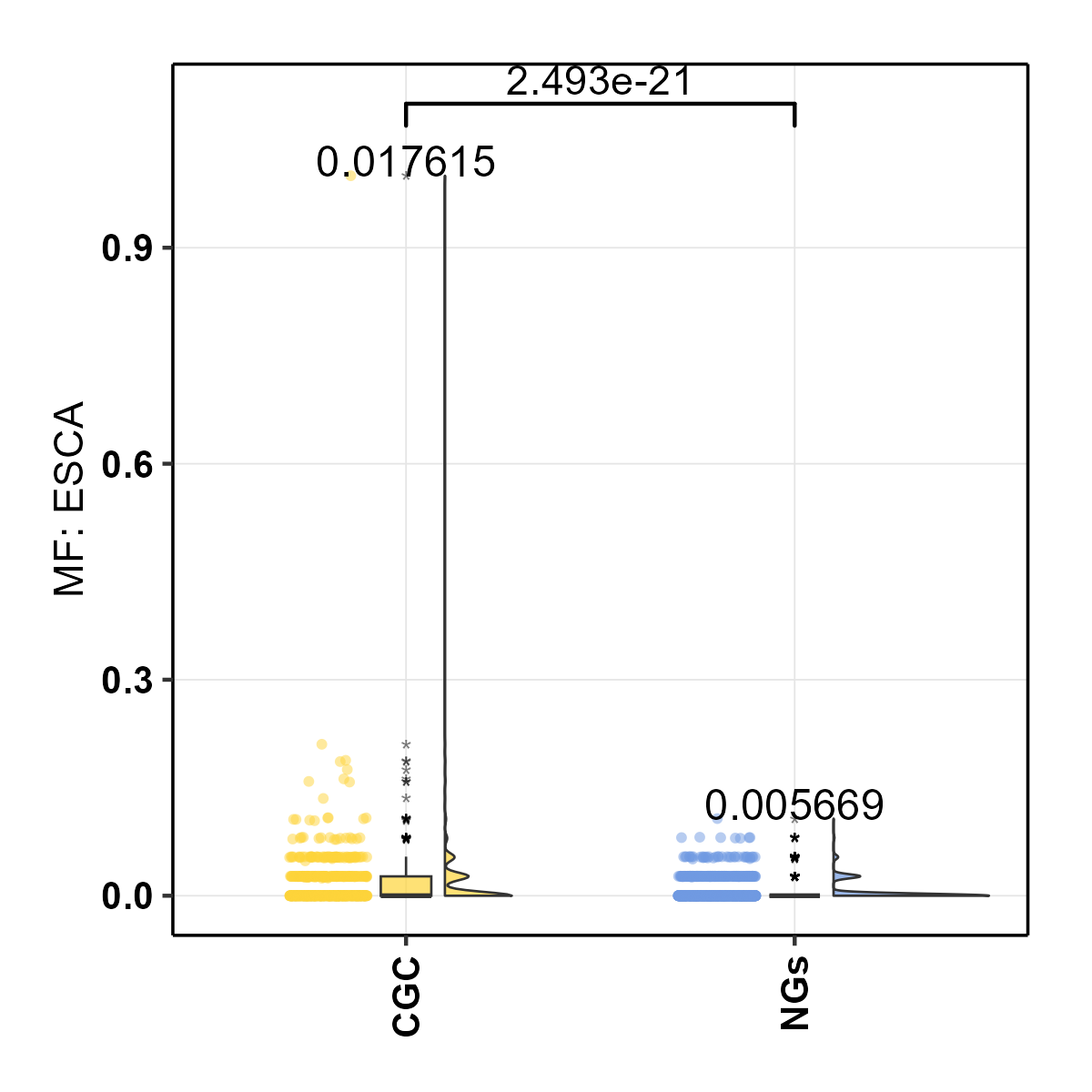

Supplement: Supplementary file 3 [file DataSheet1.ZIP › Supplementary file 5-1/Multinet/MF_ESCA.png]

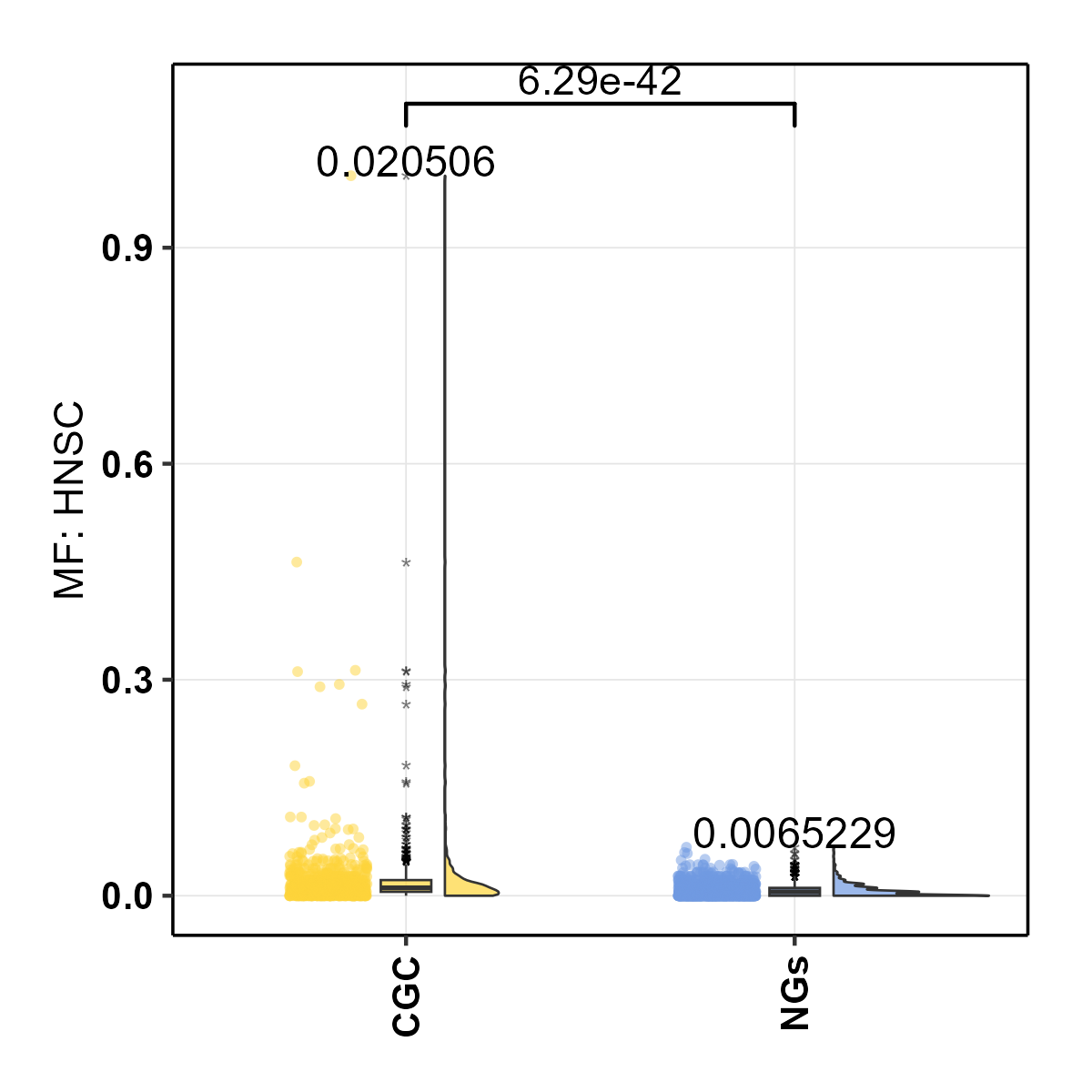

Supplement: Supplementary file 3 [file DataSheet1.ZIP › Supplementary file 5-1/Multinet/MF_HNSC.png]

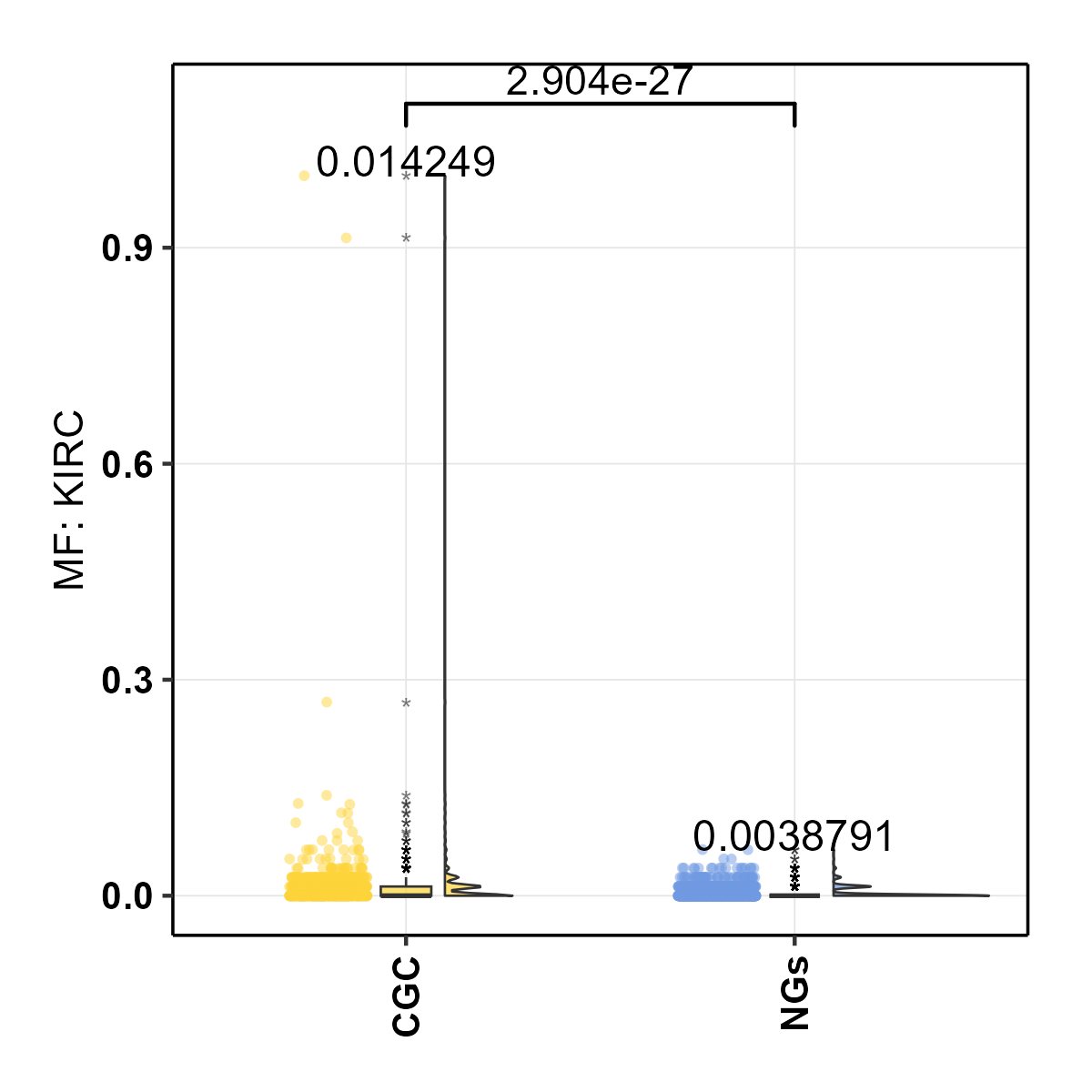

Supplement: Supplementary file 3 [file DataSheet1.ZIP › Supplementary file 5-1/Multinet/MF_KIRC.png]

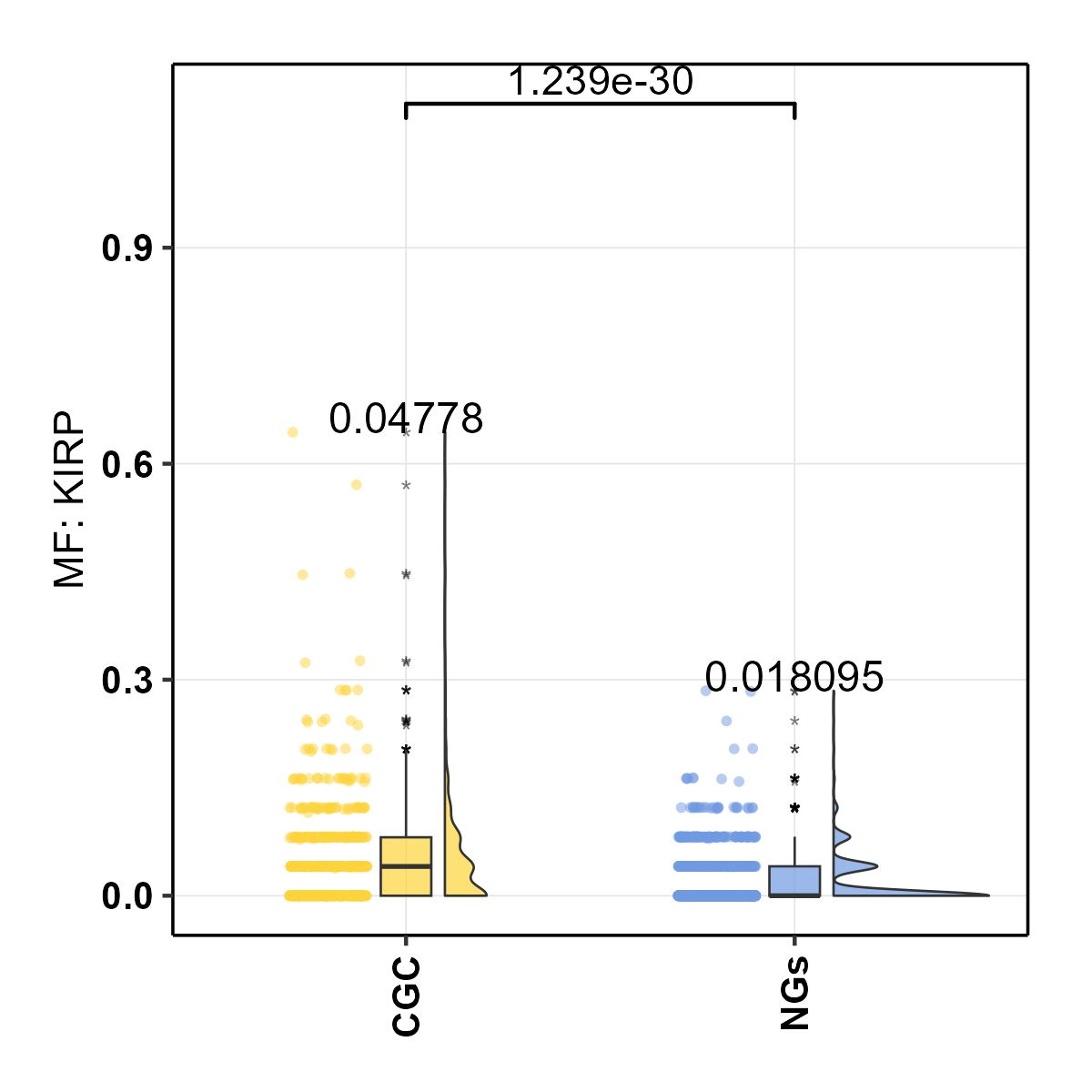

Supplement: Supplementary file 3 [file DataSheet1.ZIP › Supplementary file 5-1/Multinet/MF_KIRP.png]

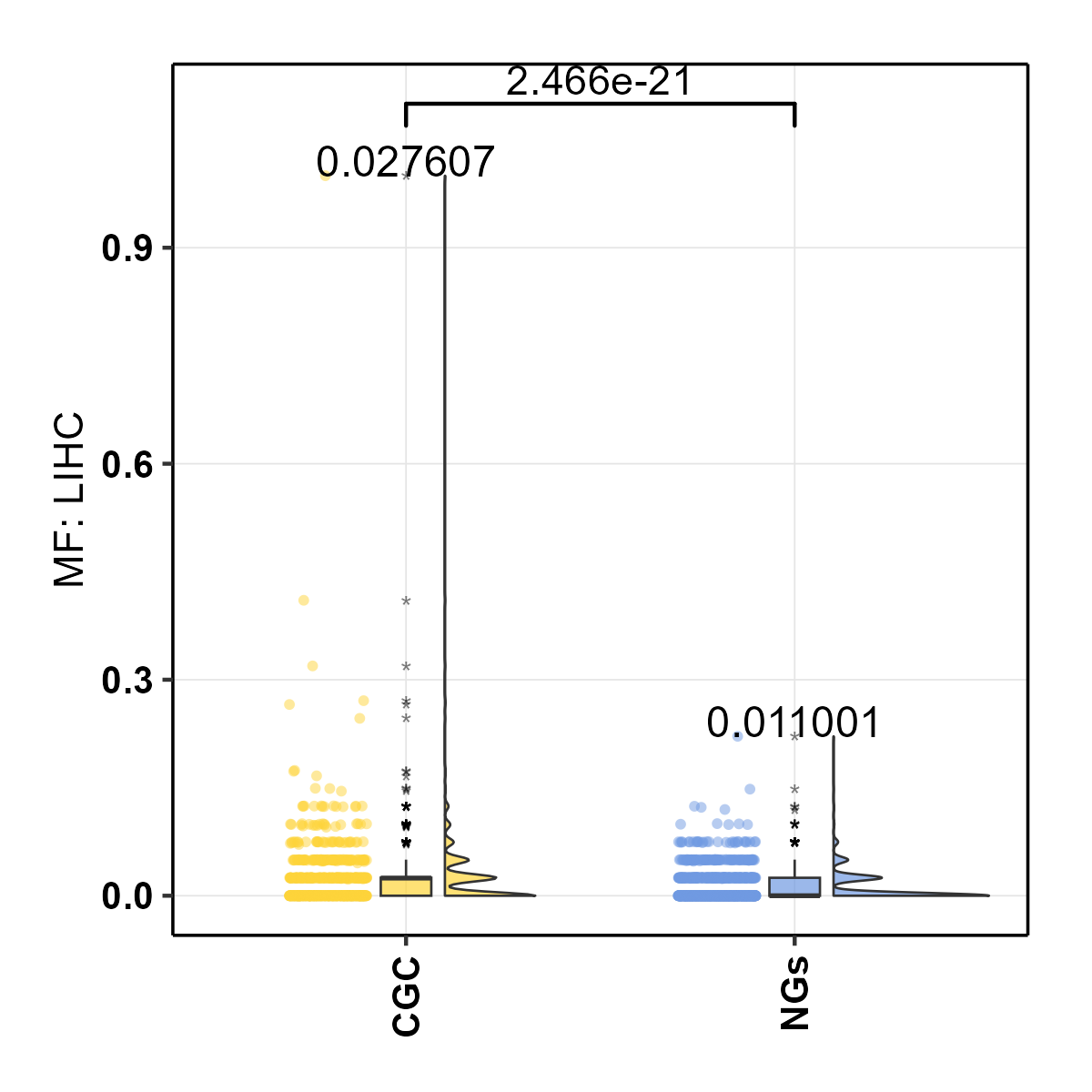

Supplement: Supplementary file 3 [file DataSheet1.ZIP › Supplementary file 5-1/Multinet/MF_LIHC.png]

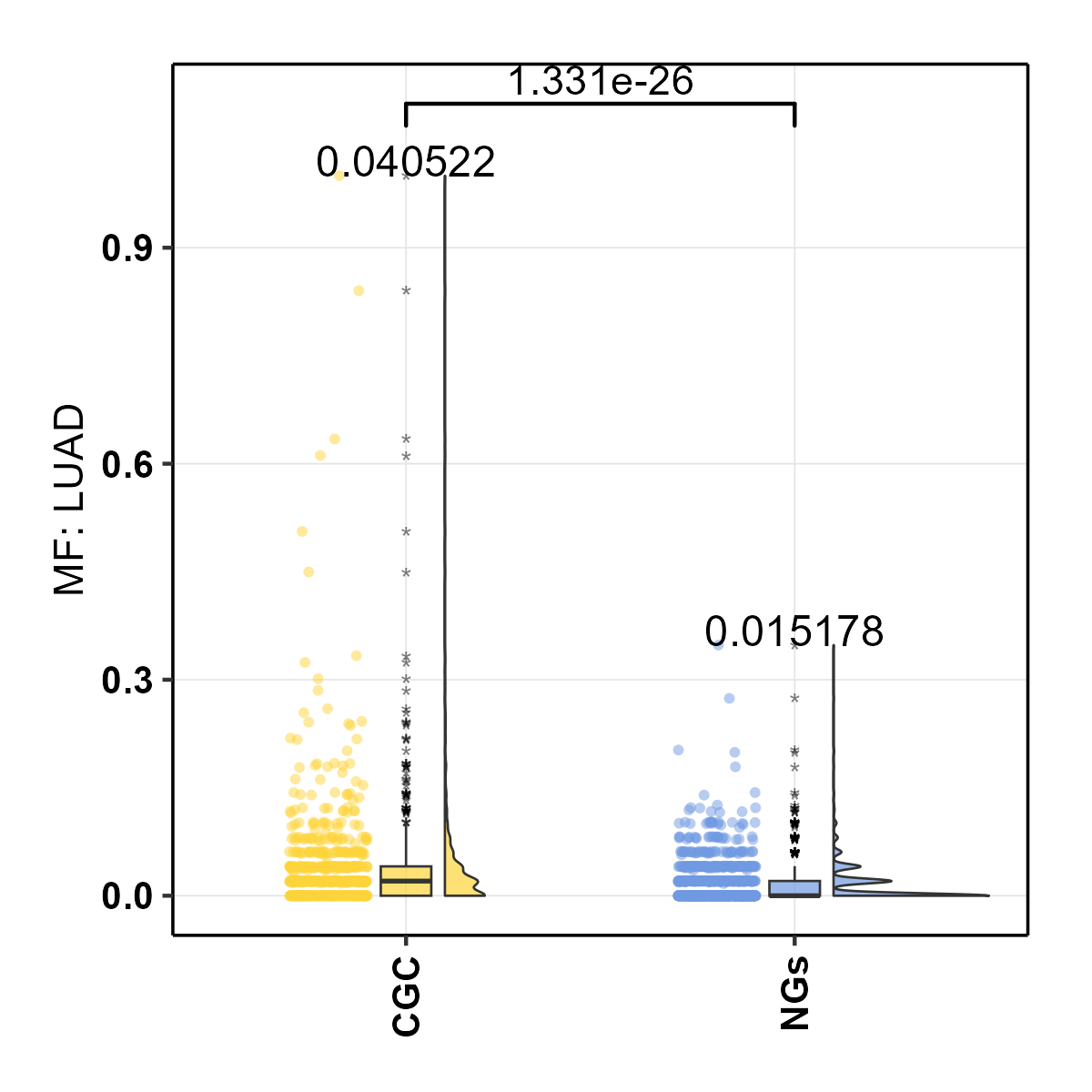

Supplement: Supplementary file 3 [file DataSheet1.ZIP › Supplementary file 5-1/Multinet/MF_LUAD.png]

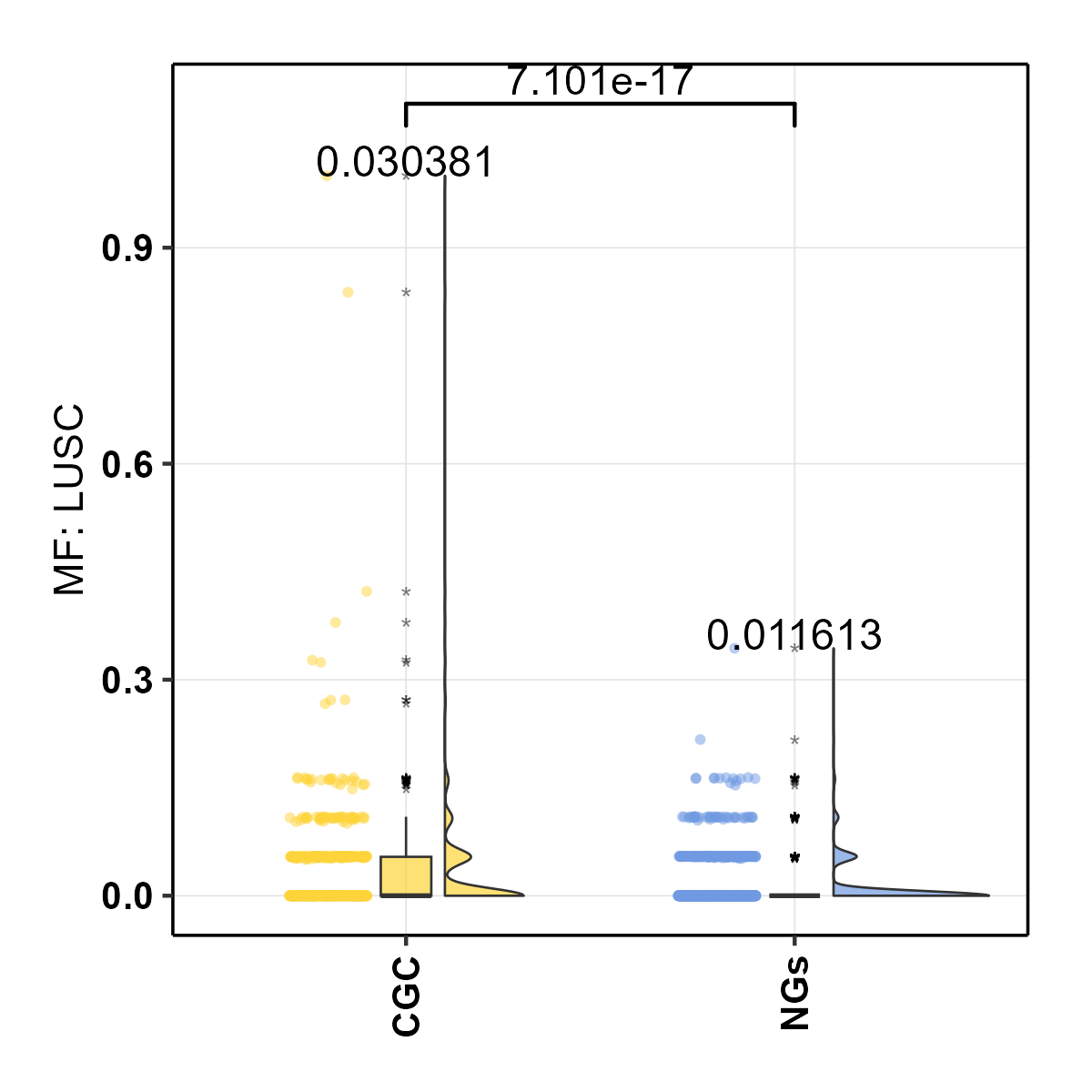

Supplement: Supplementary file 3 [file DataSheet1.ZIP › Supplementary file 5-1/Multinet/MF_LUSC.png]

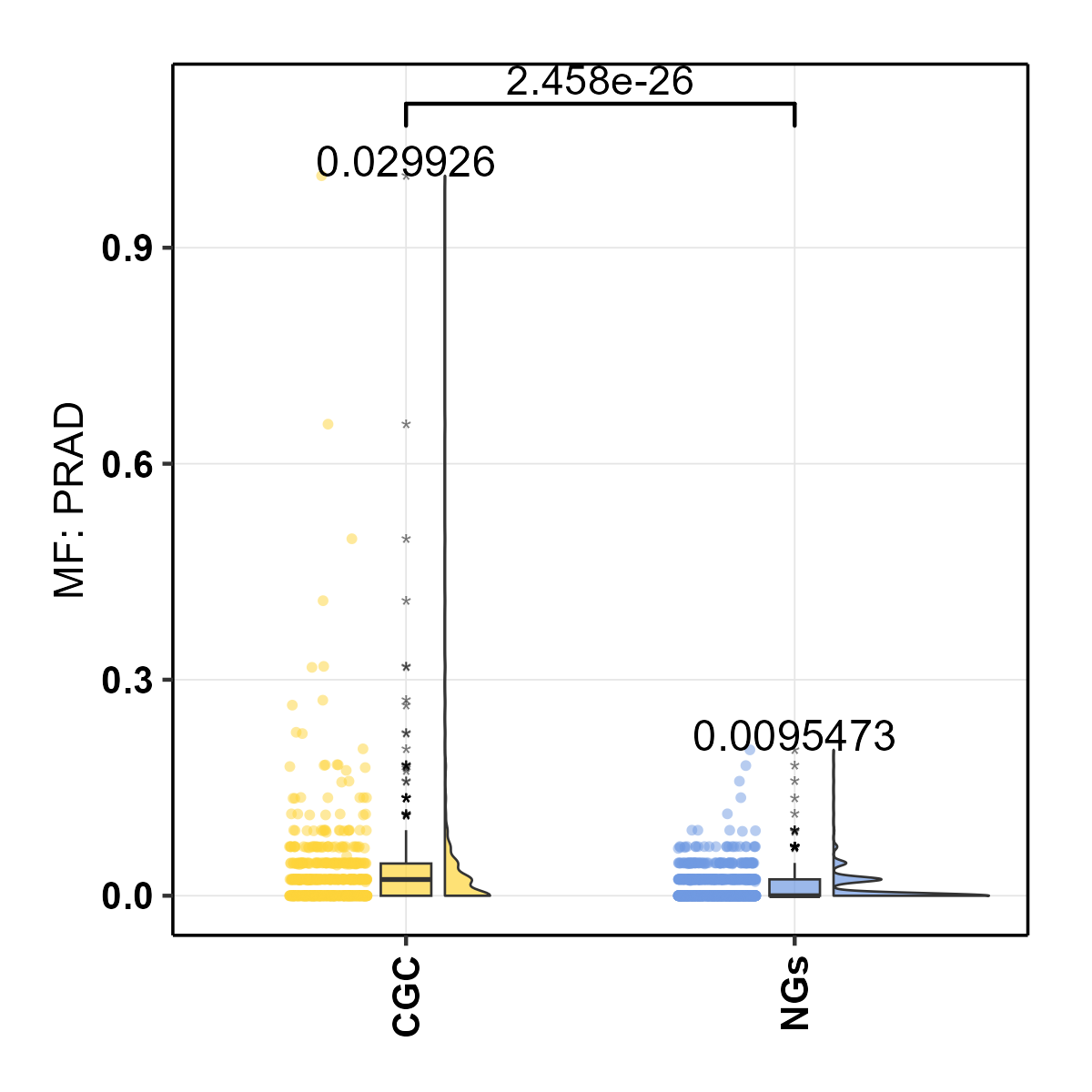

Supplement: Supplementary file 3 [file DataSheet1.ZIP › Supplementary file 5-1/Multinet/MF_PRAD.png]

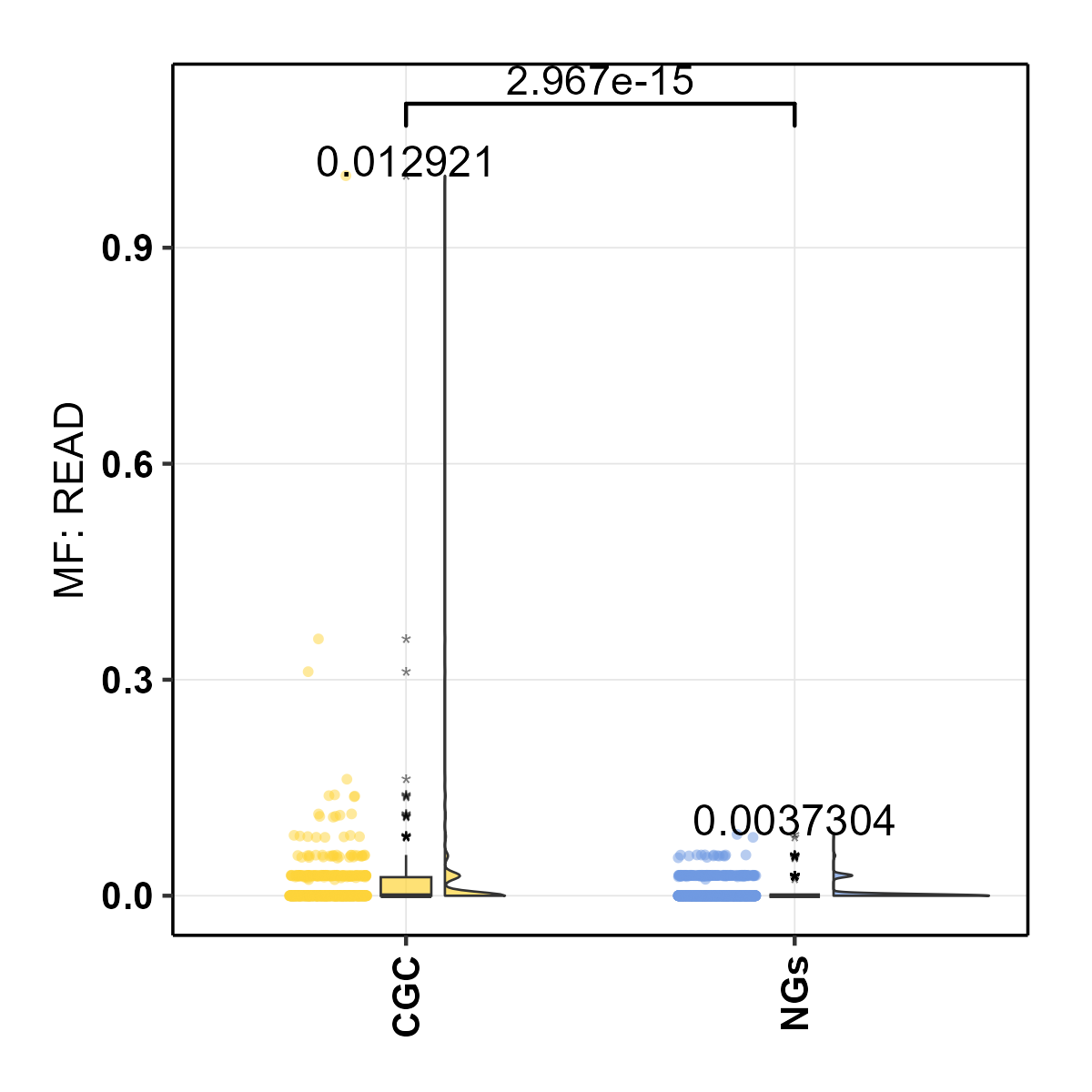

Supplement: Supplementary file 3 [file DataSheet1.ZIP › Supplementary file 5-1/Multinet/MF_READ.png]

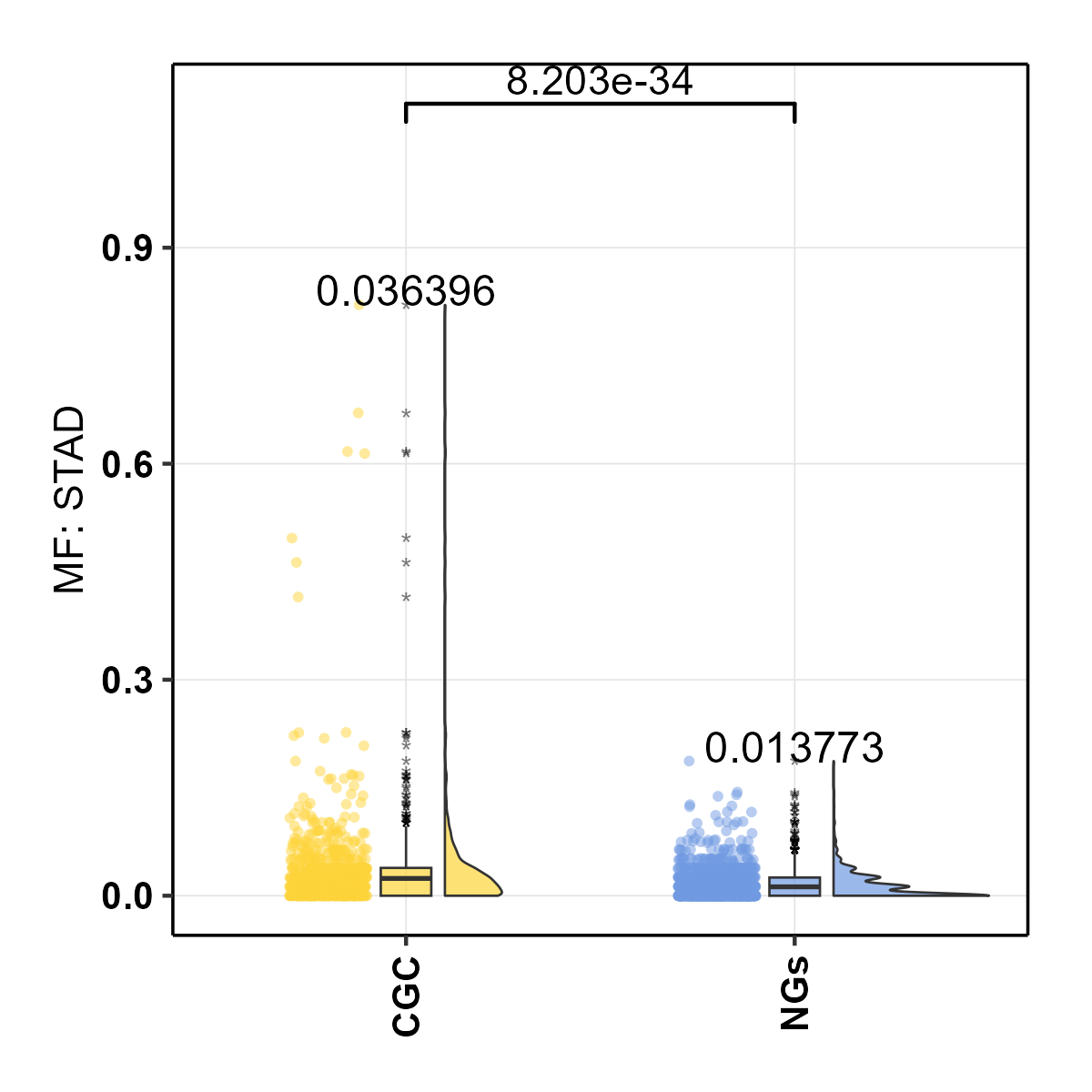

Supplement: Supplementary file 3 [file DataSheet1.ZIP › Supplementary file 5-1/Multinet/MF_STAD.png]

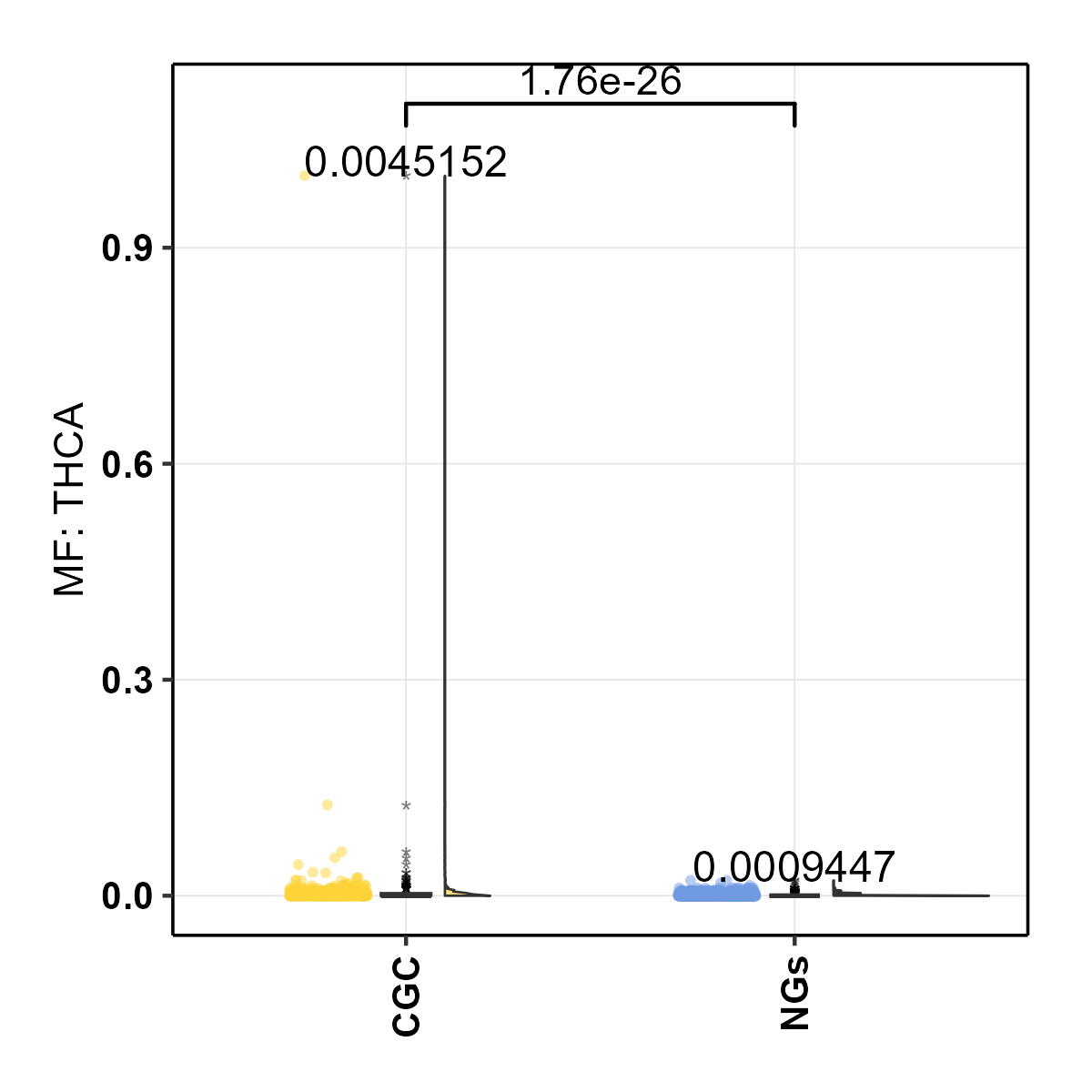

Supplement: Supplementary file 3 [file DataSheet1.ZIP › Supplementary file 5-1/Multinet/MF_THCA.png]

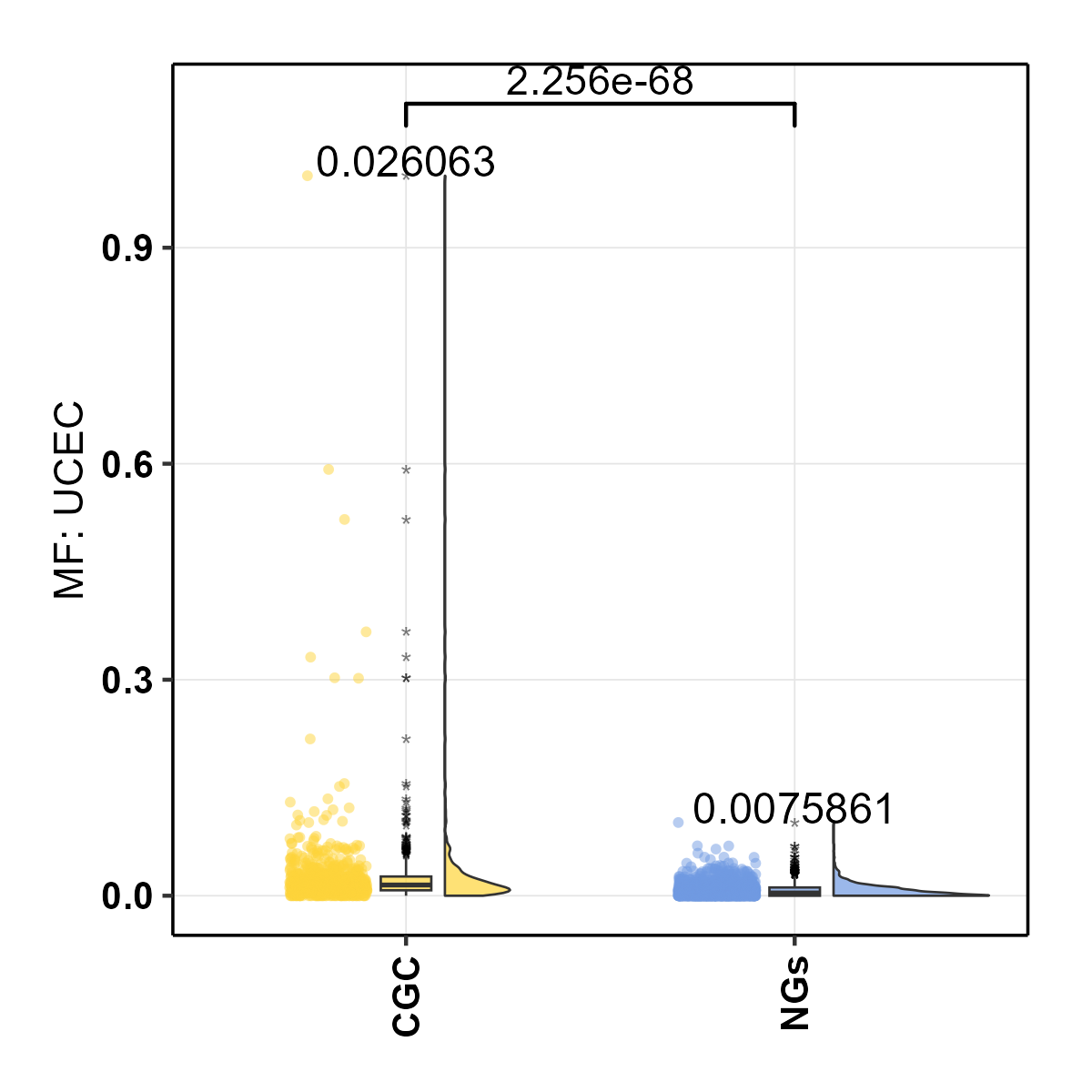

Supplement: Supplementary file 3 [file DataSheet1.ZIP › Supplementary file 5-1/Multinet/MF_UCEC.png]

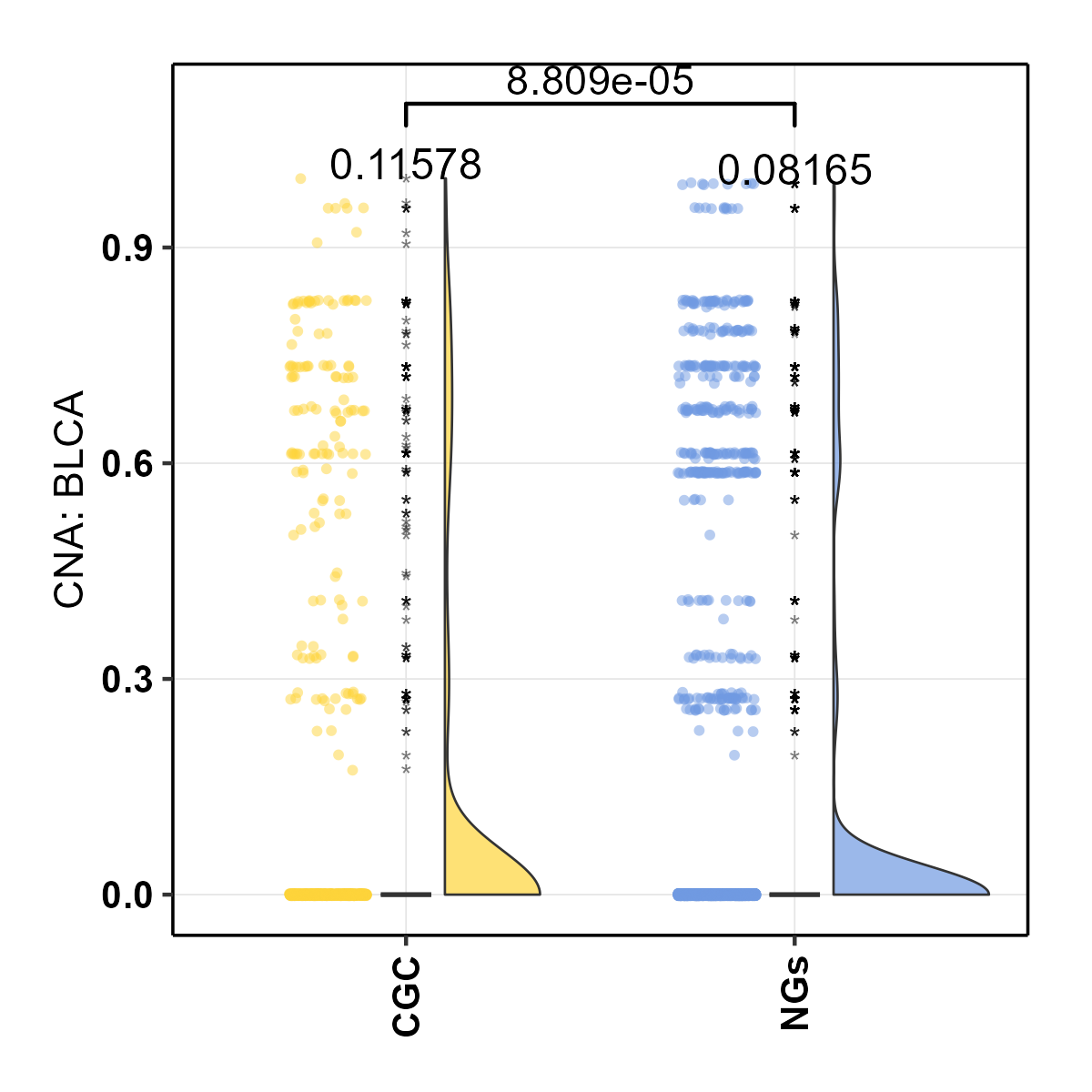

Supplement: Supplementary file 5 [file DataSheet2.ZIP › Supplementary file 5-2/PCNet/CNA_BLCA.png]

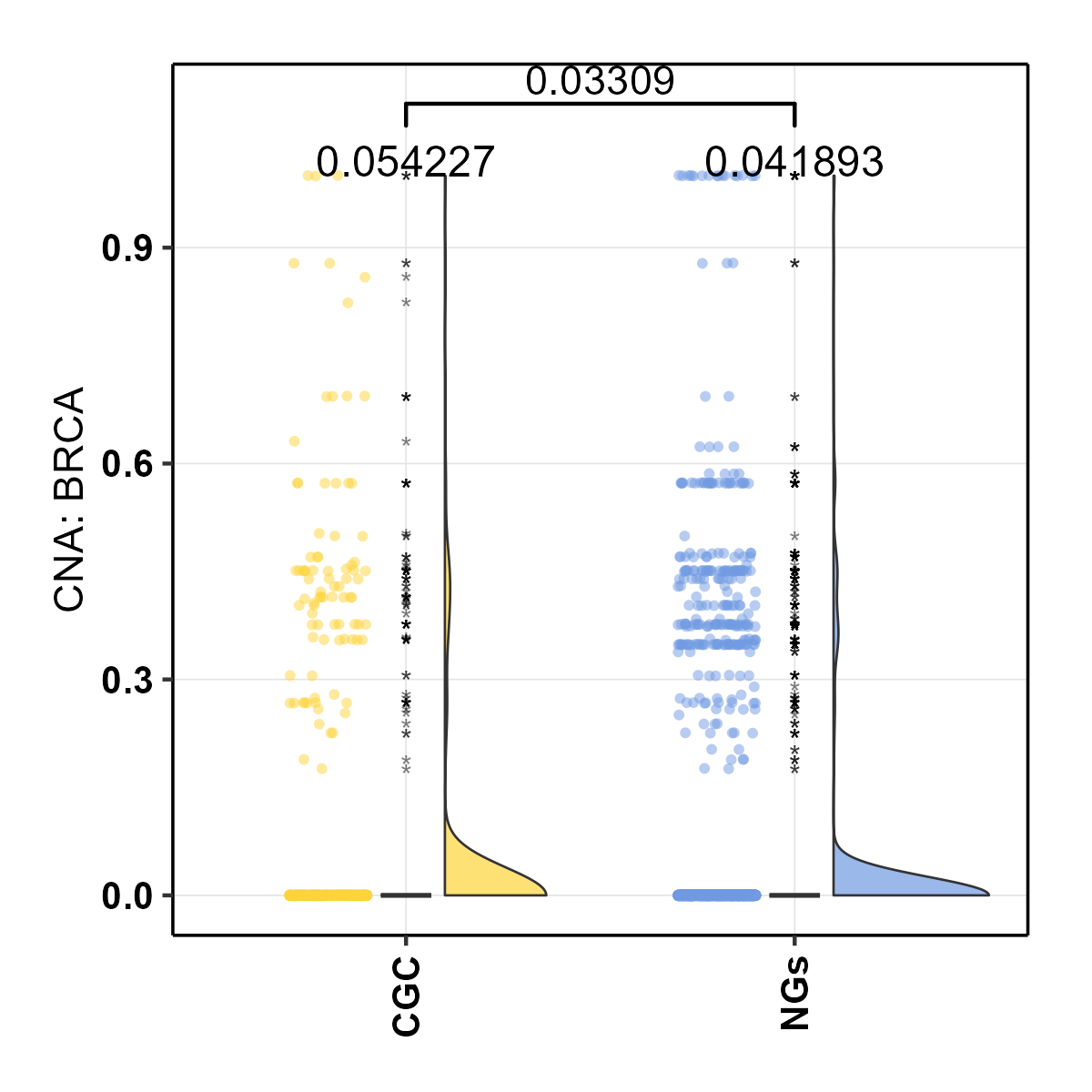

Supplement: Supplementary file 5 [file DataSheet2.ZIP › Supplementary file 5-2/PCNet/CNA_BRCA.png]

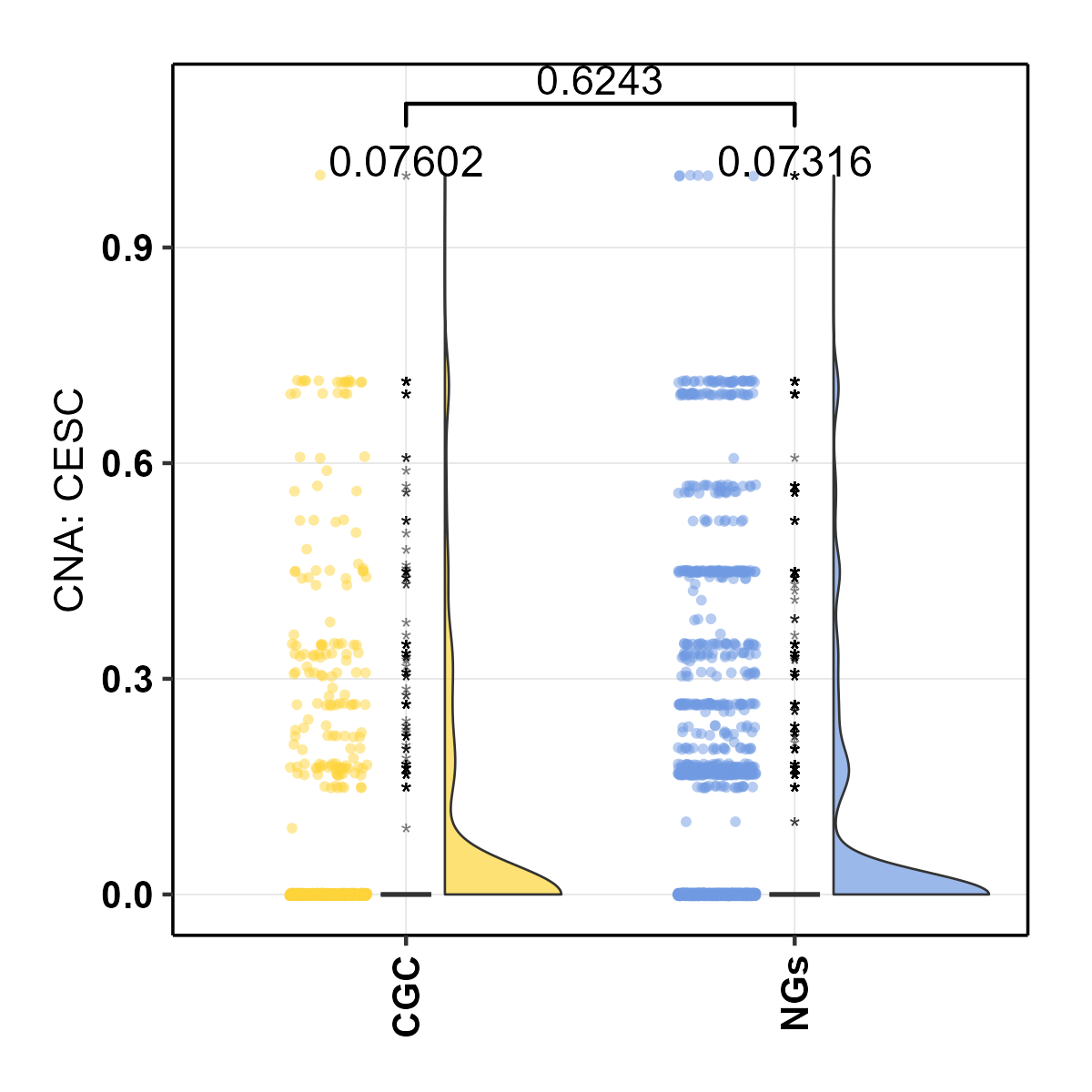

Supplement: Supplementary file 5 [file DataSheet2.ZIP › Supplementary file 5-2/PCNet/CNA_CESC.png]

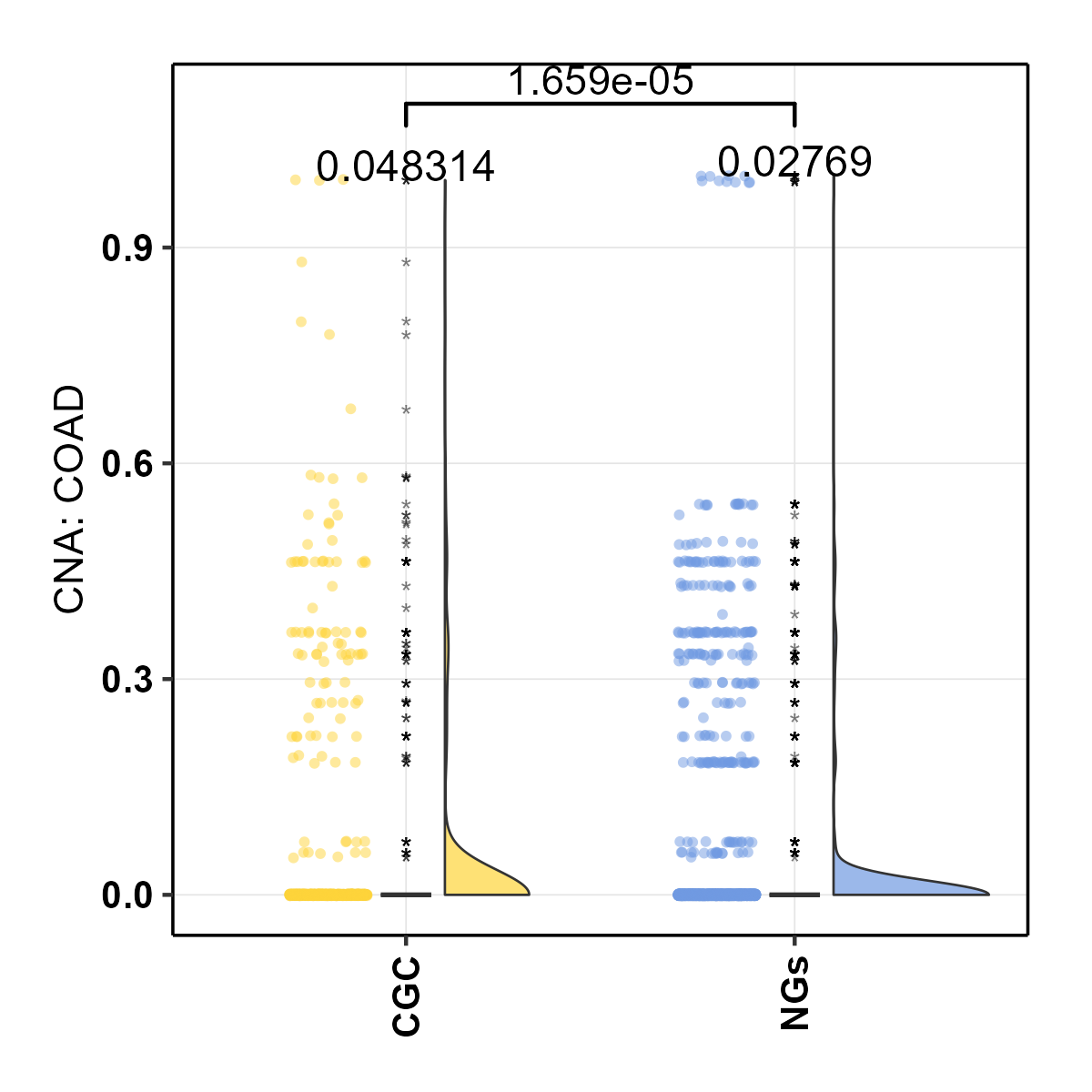

Supplement: Supplementary file 5 [file DataSheet2.ZIP › Supplementary file 5-2/PCNet/CNA_COAD.png]

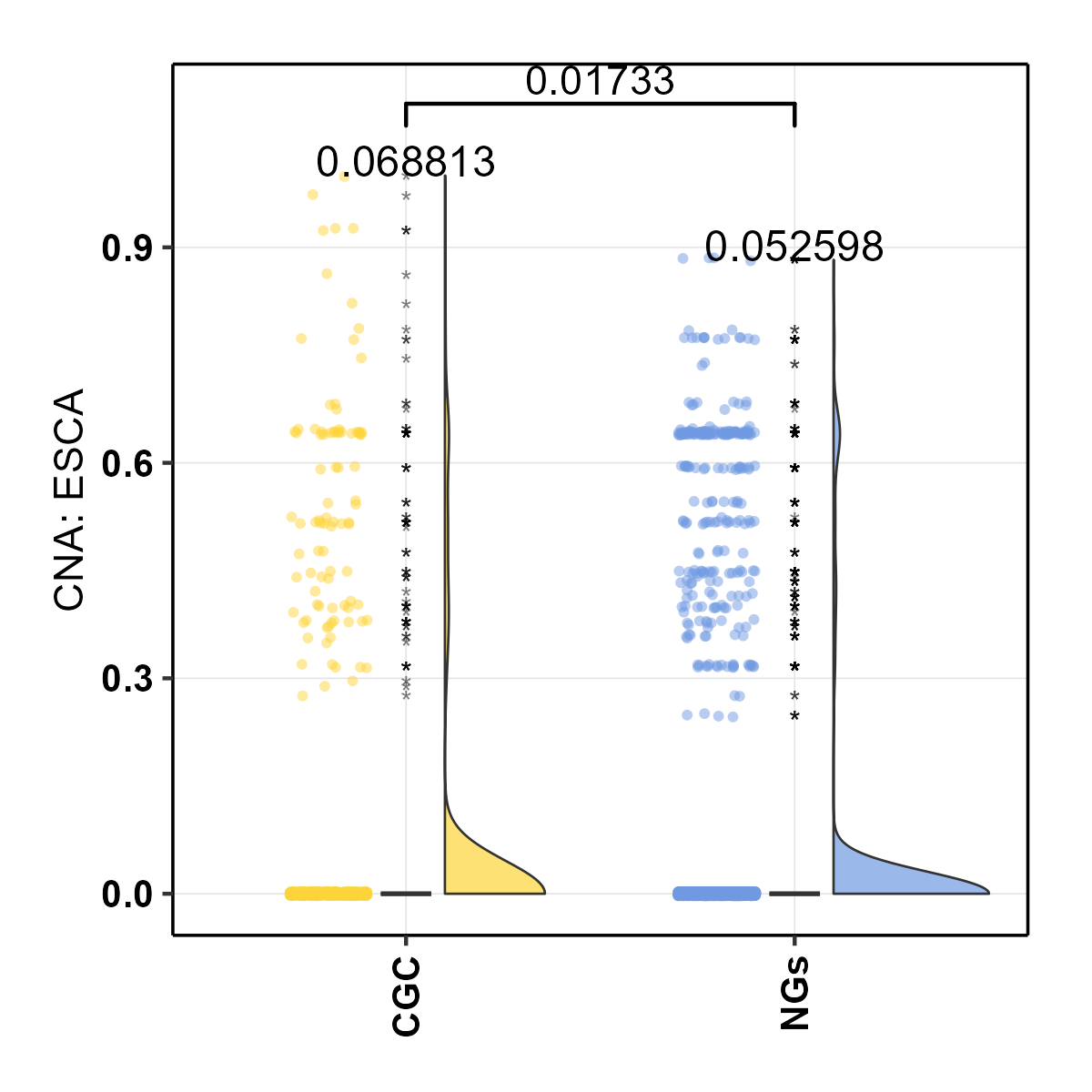

Supplement: Supplementary file 5 [file DataSheet2.ZIP › Supplementary file 5-2/PCNet/CNA_ESCA.png]

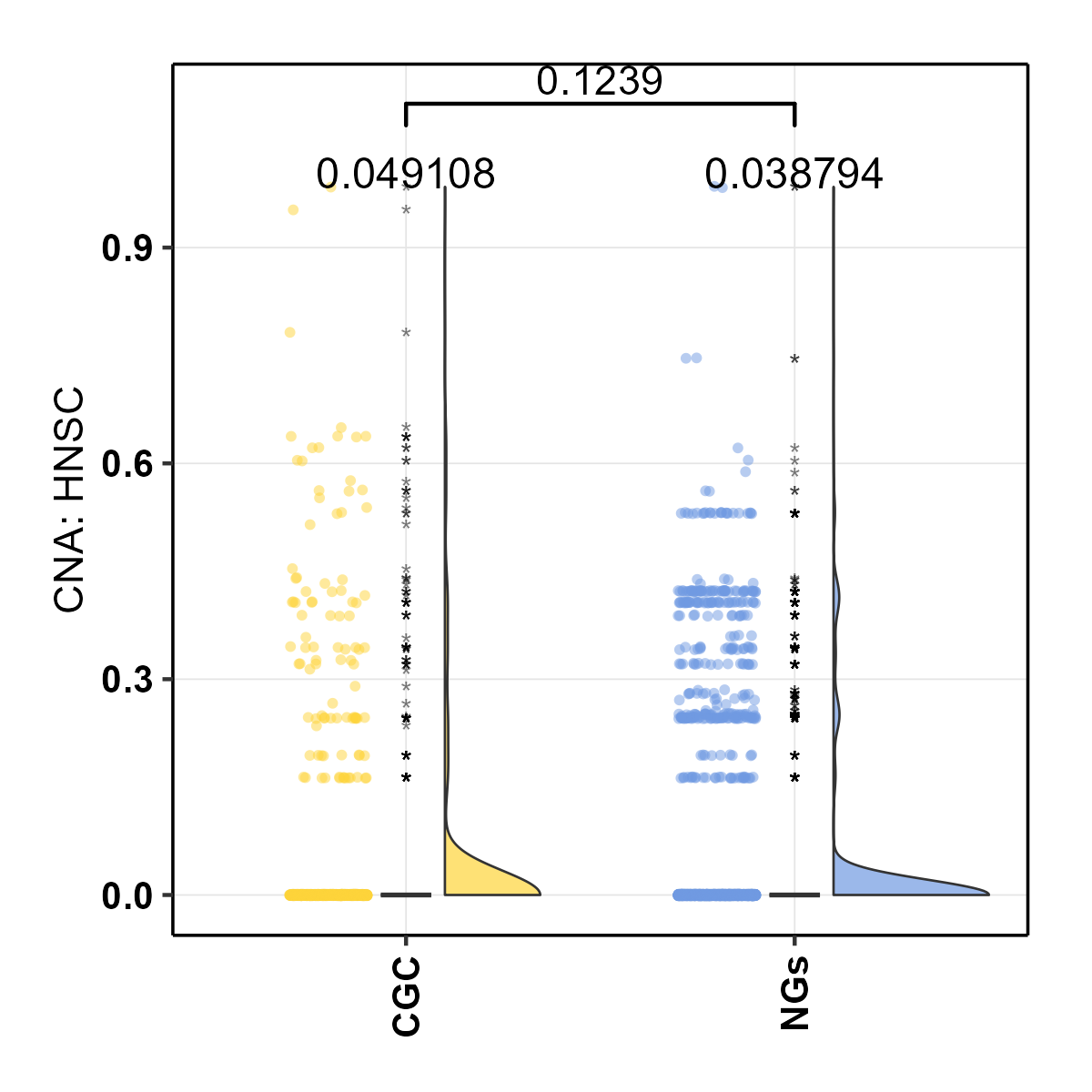

Supplement: Supplementary file 5 [file DataSheet2.ZIP › Supplementary file 5-2/PCNet/CNA_HNSC.png]

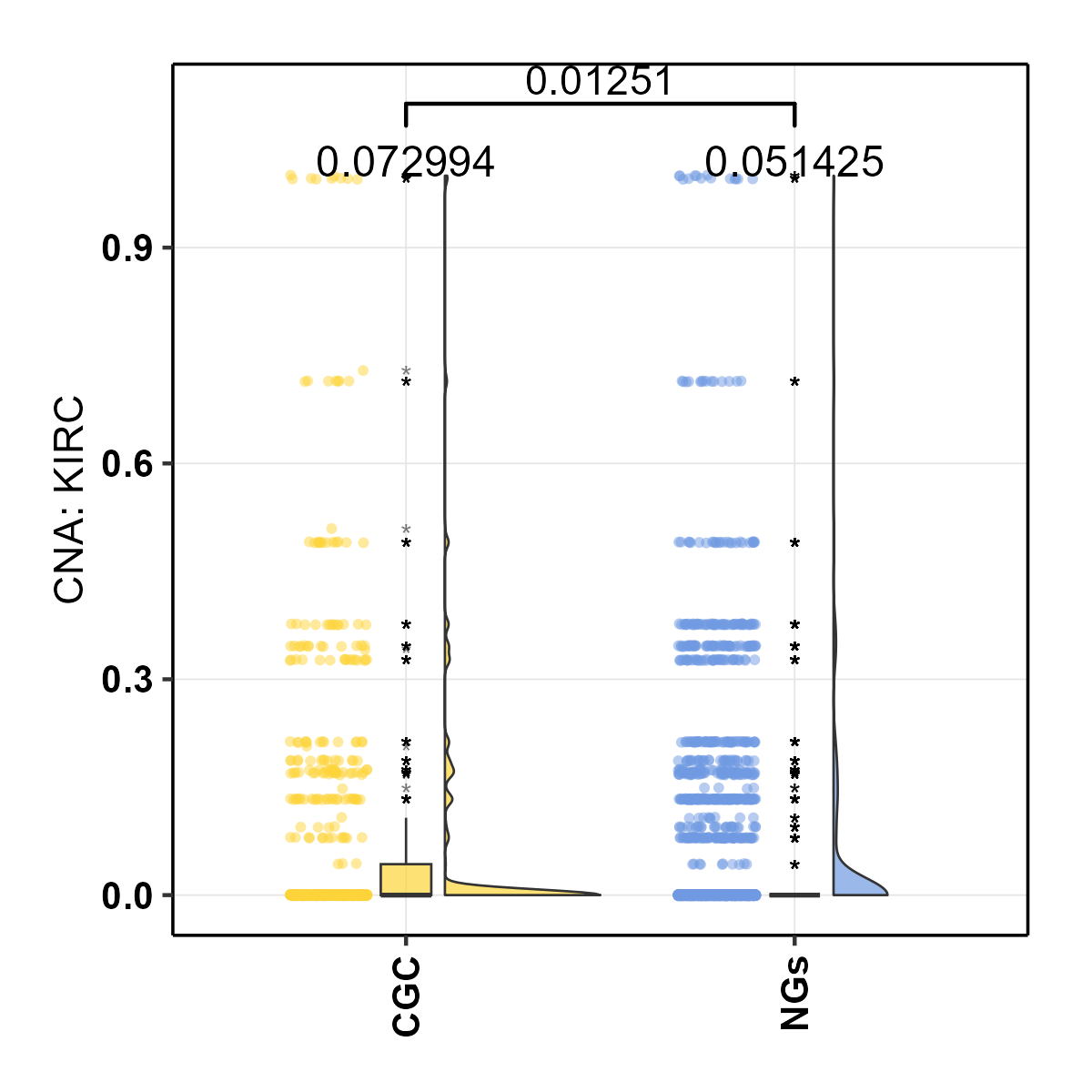

Supplement: Supplementary file 5 [file DataSheet2.ZIP › Supplementary file 5-2/PCNet/CNA_KIRC.png]

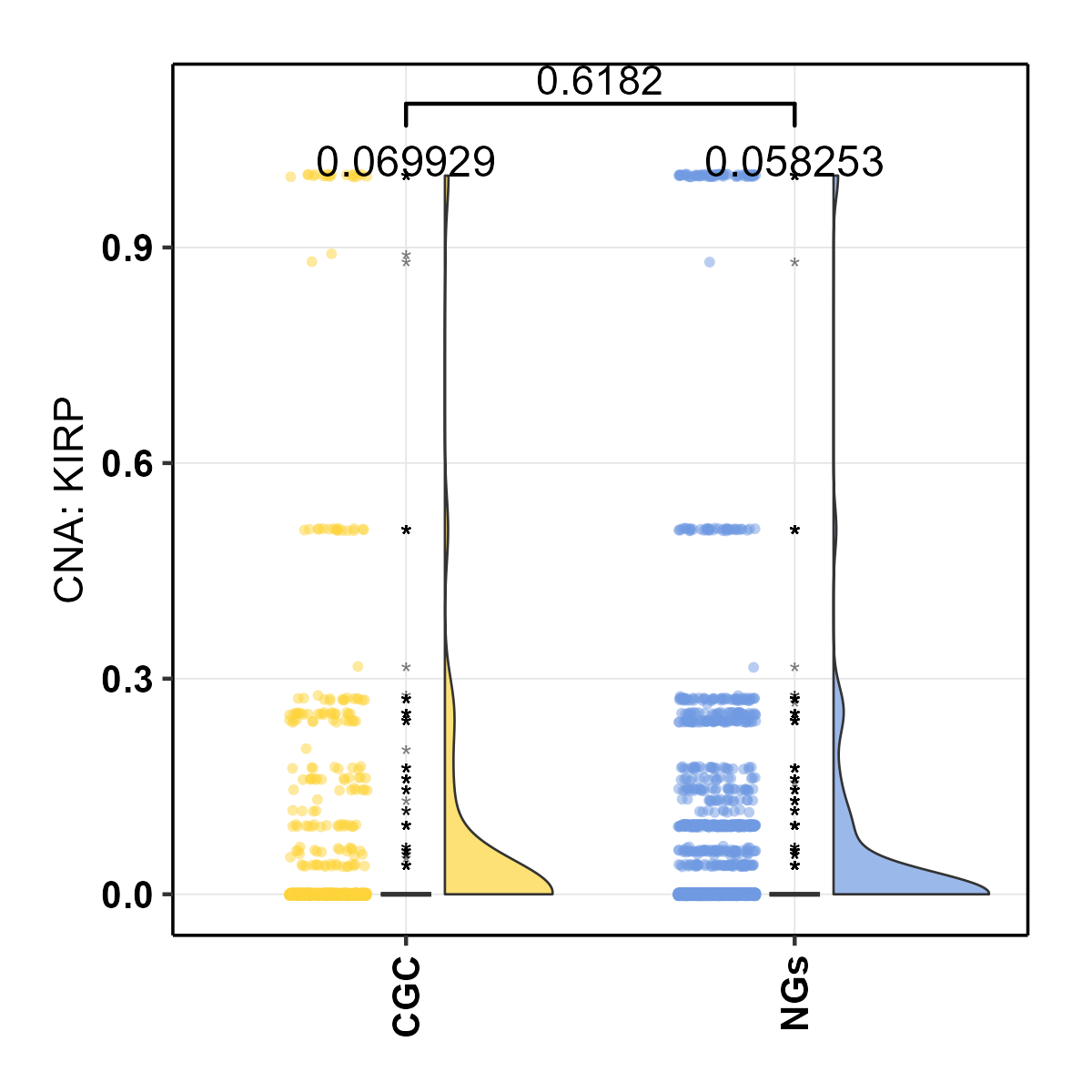

Supplement: Supplementary file 5 [file DataSheet2.ZIP › Supplementary file 5-2/PCNet/CNA_KIRP.png]
