# Supplementary figures and images for: MONet: cancer driver gene identification algorithm based on integrated analysis of multi-omics data and network models (part 1 of 4)
Source: Exp Biol Med (Maywood). 2025 Feb 4;250:10399. doi: 10.3389/ebm.2025.10399 (PMC11834253; doi:10.3389/ebm.2025.10399)

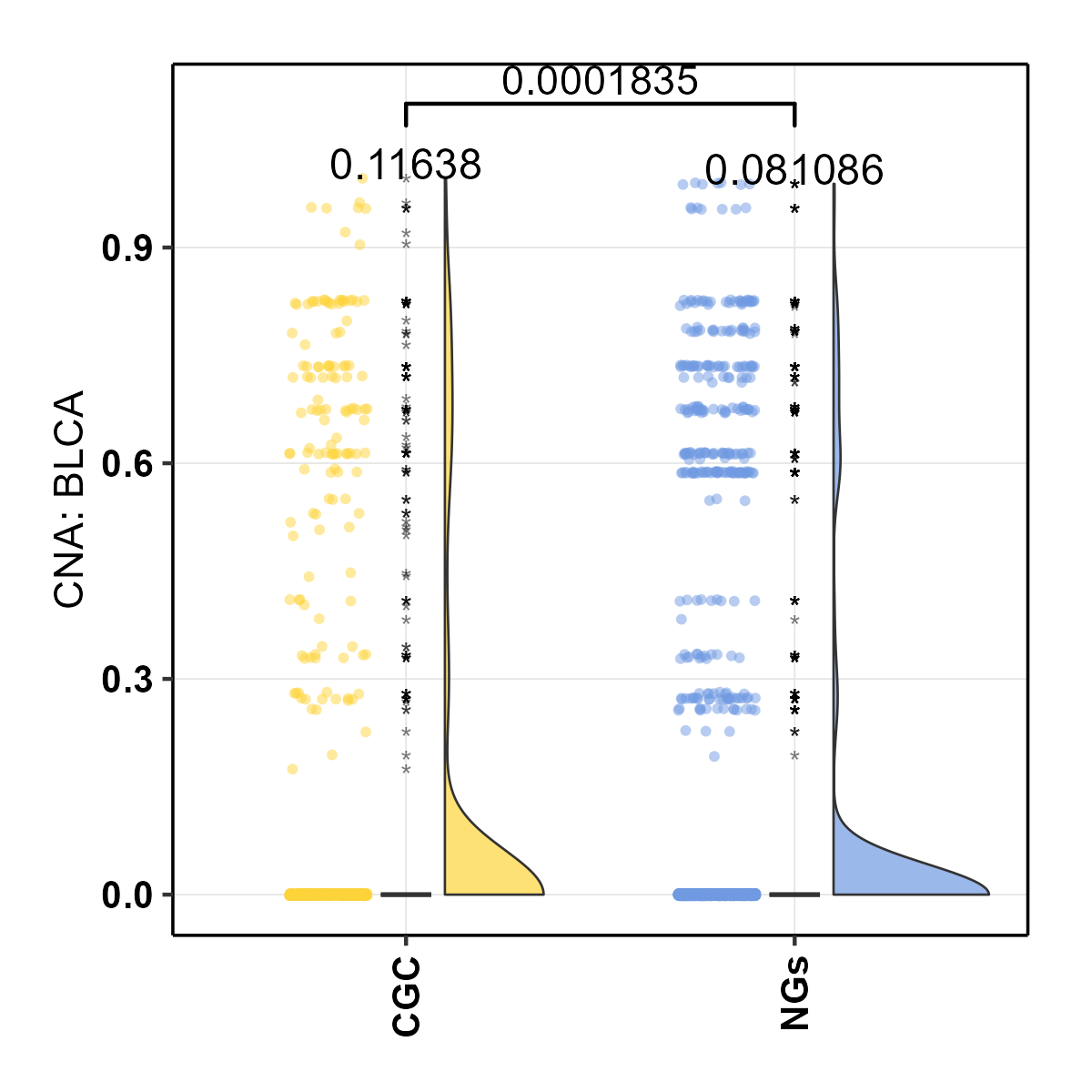

Supplement: Supplementary file 3 [file DataSheet1.ZIP › Supplementary file 5-1/IReflndex/CNA_BLCA.png]

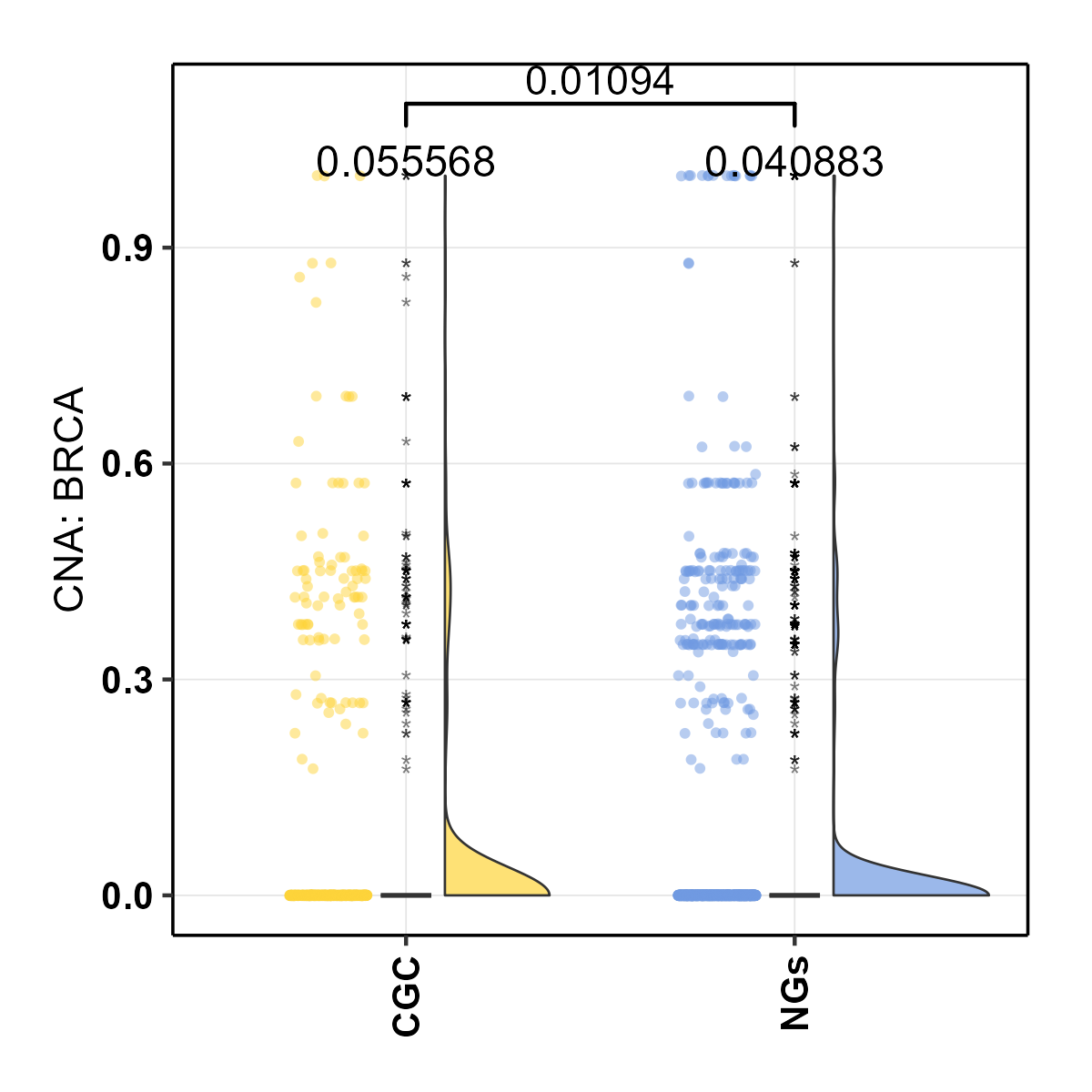

Supplement: Supplementary file 3 [file DataSheet1.ZIP › Supplementary file 5-1/IReflndex/CNA_BRCA.png]

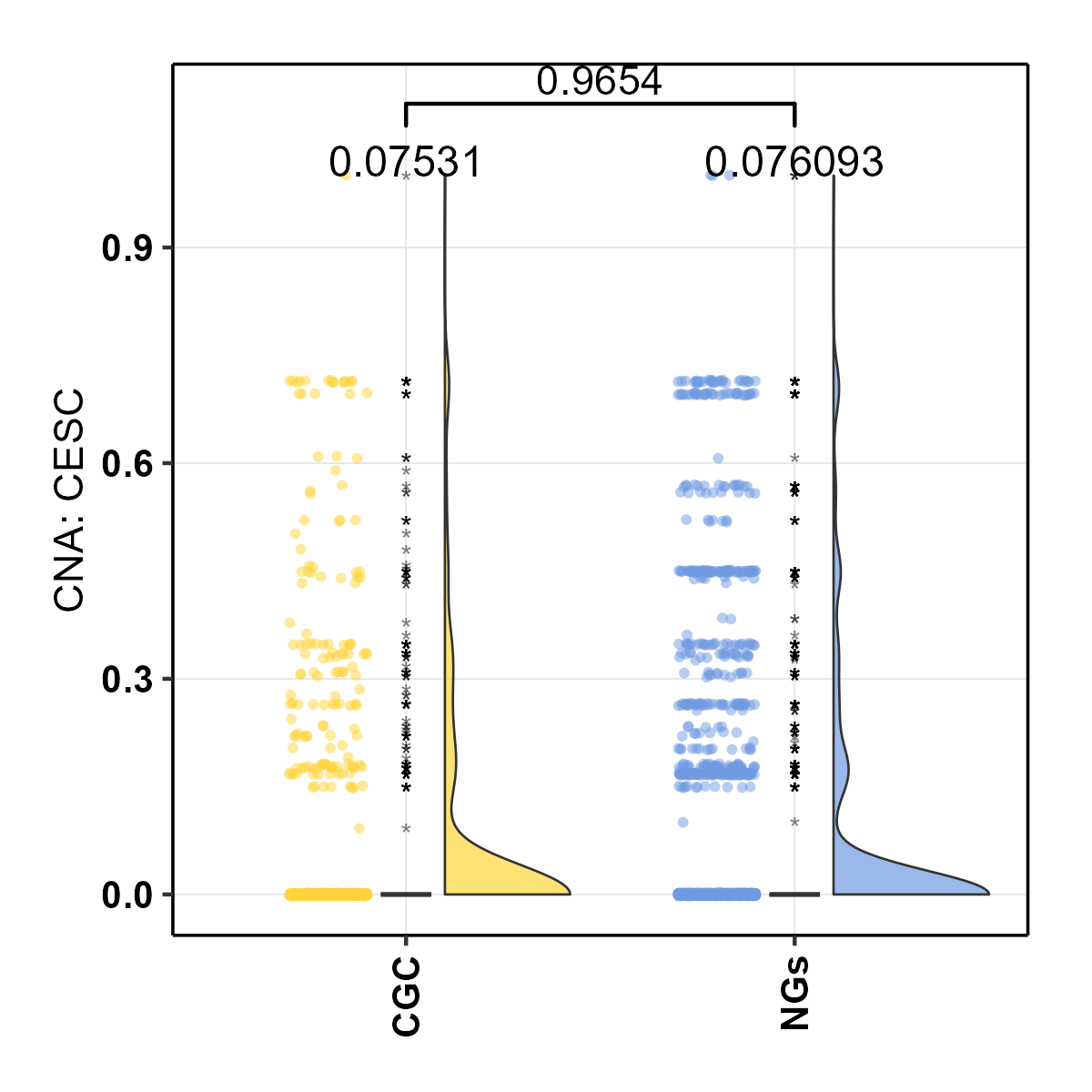

Supplement: Supplementary file 3 [file DataSheet1.ZIP › Supplementary file 5-1/IReflndex/CNA_CESC.png]

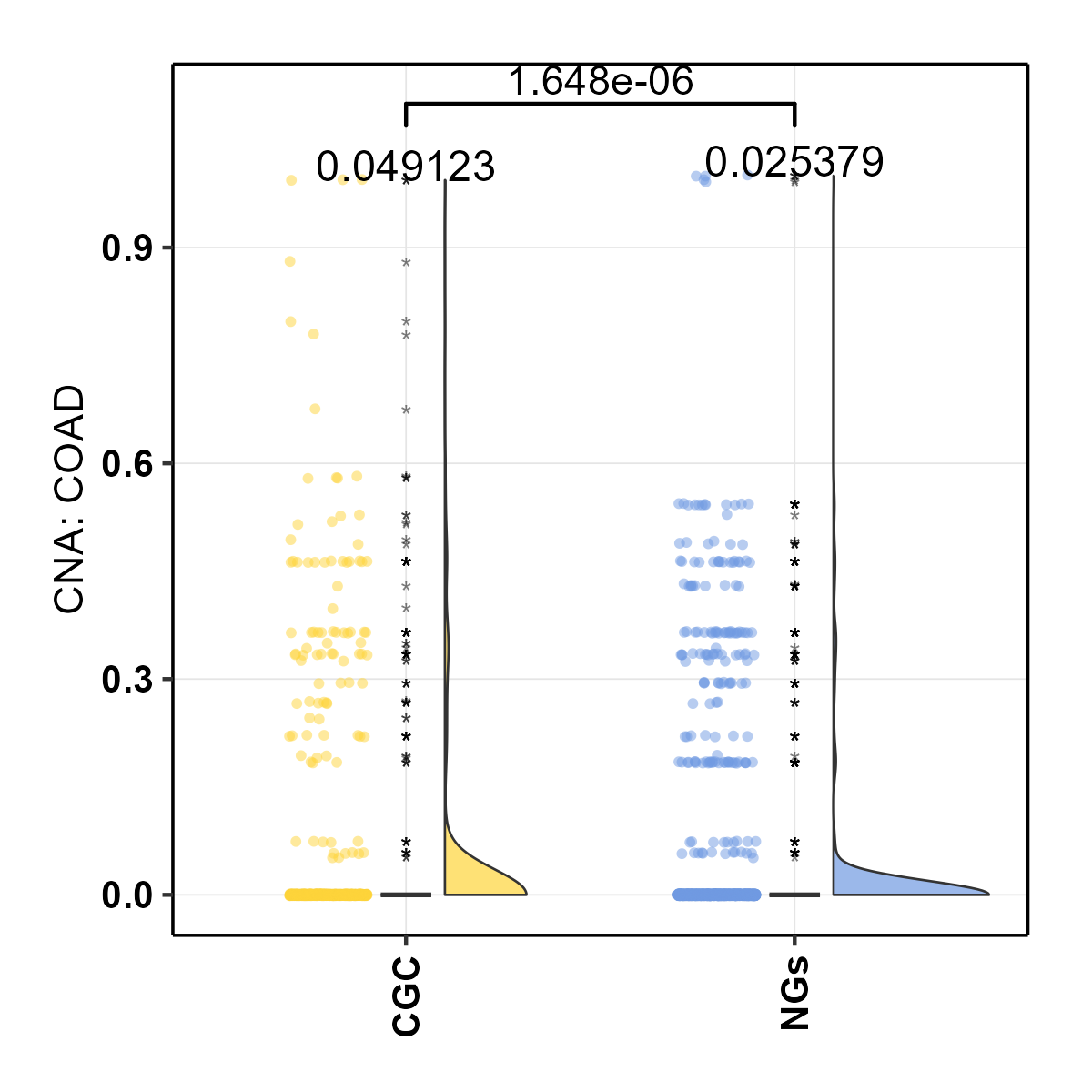

Supplement: Supplementary file 3 [file DataSheet1.ZIP › Supplementary file 5-1/IReflndex/CNA_COAD.png]

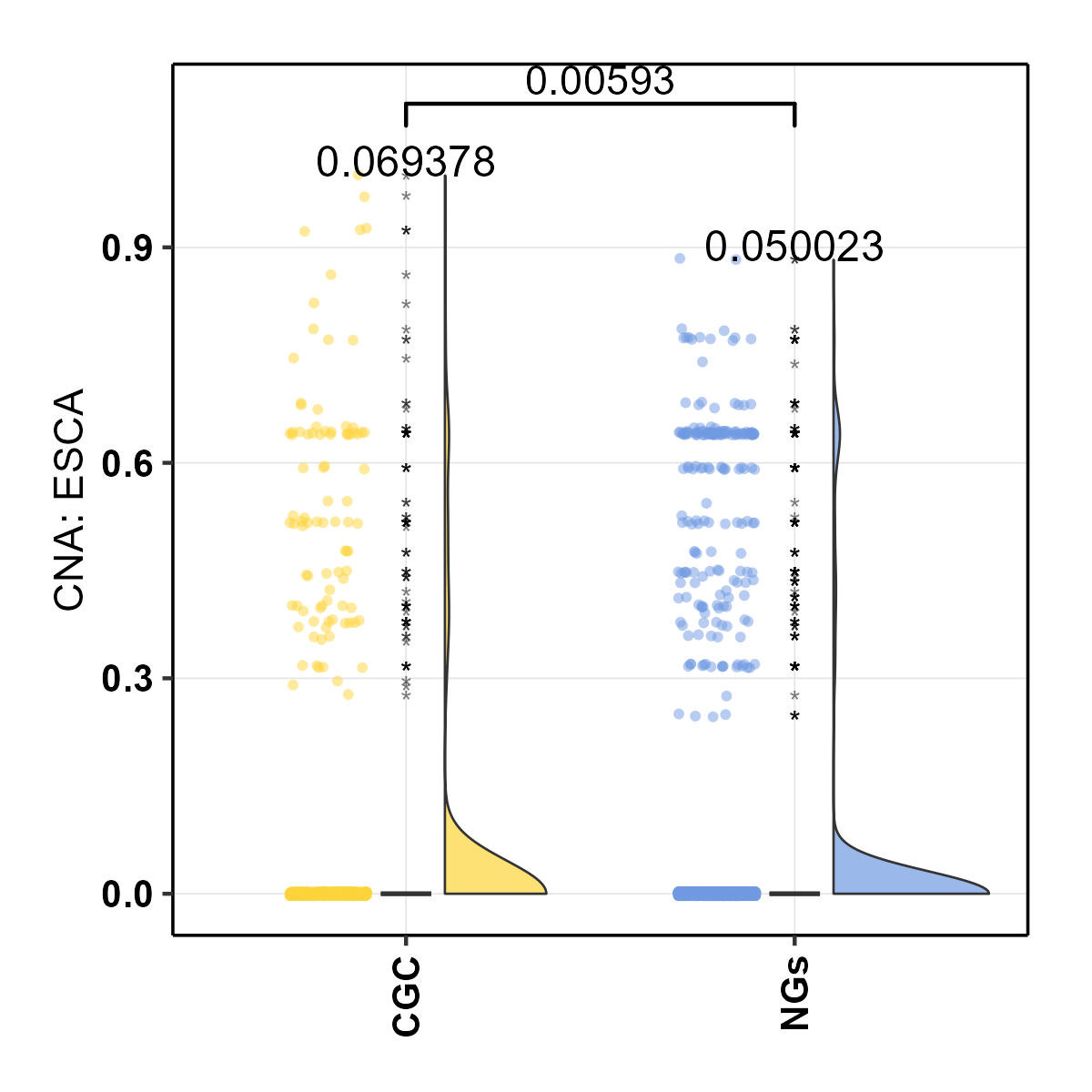

Supplement: Supplementary file 3 [file DataSheet1.ZIP › Supplementary file 5-1/IReflndex/CNA_ESCA.png]

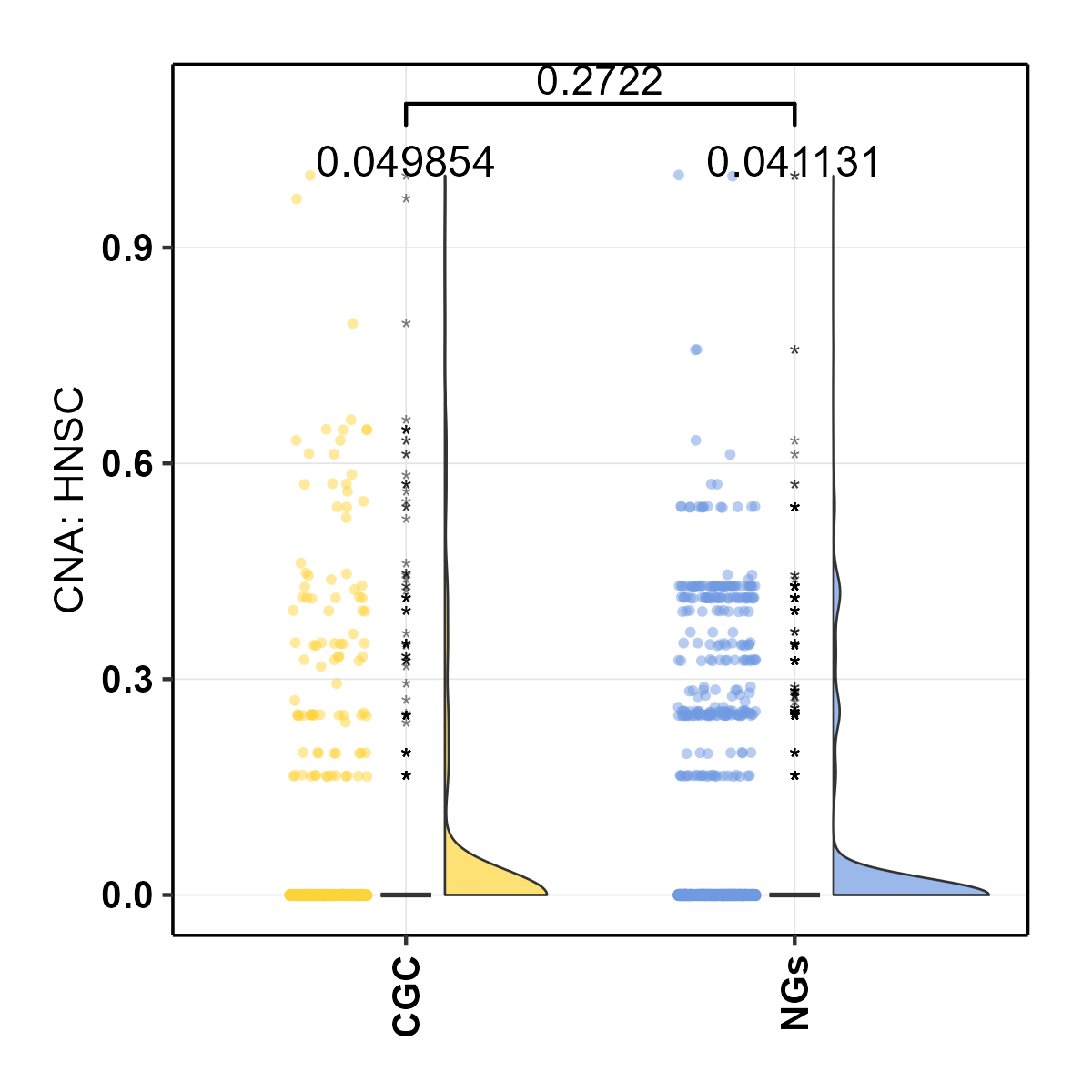

Supplement: Supplementary file 3 [file DataSheet1.ZIP › Supplementary file 5-1/IReflndex/CNA_HNSC.png]

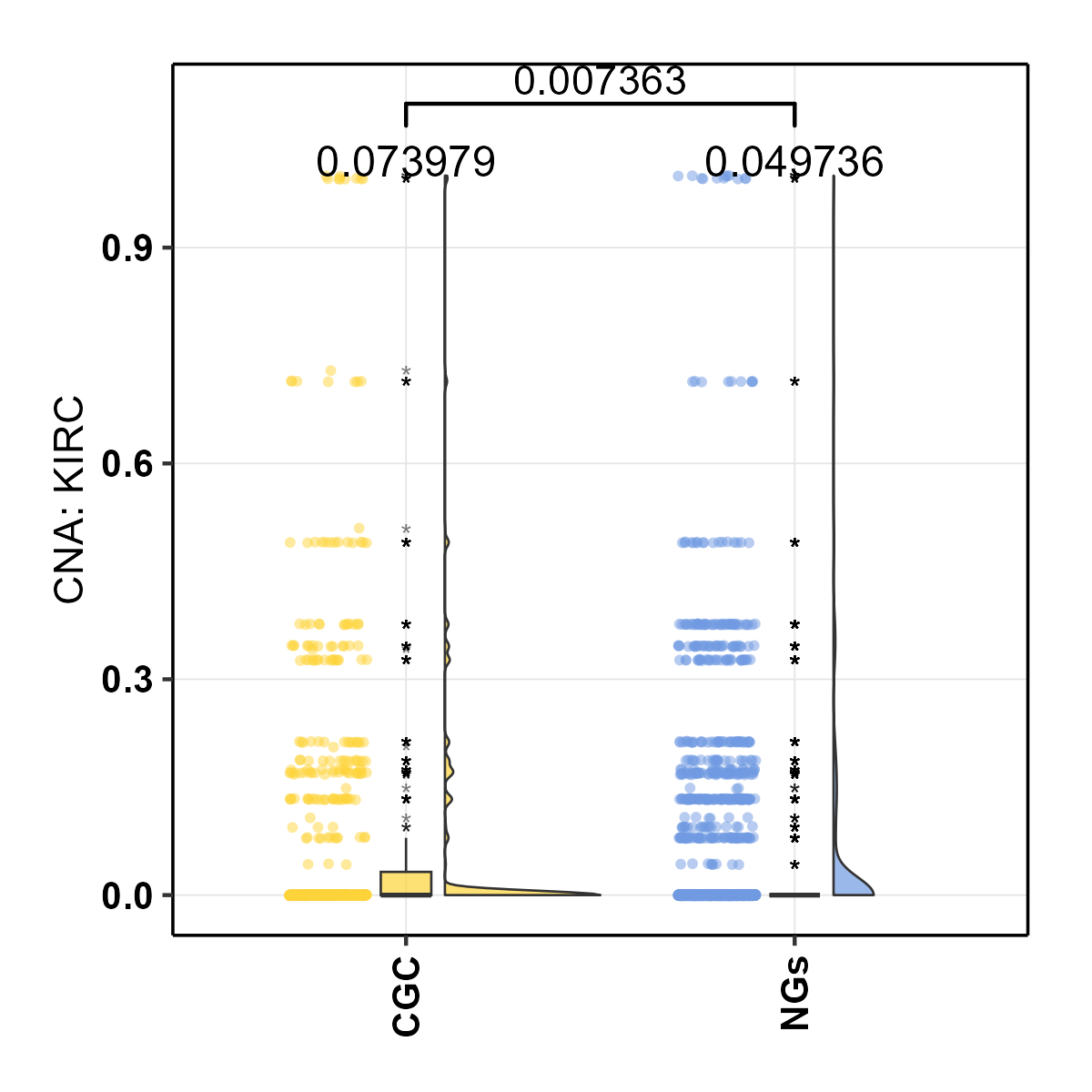

Supplement: Supplementary file 3 [file DataSheet1.ZIP › Supplementary file 5-1/IReflndex/CNA_KIRC.png]

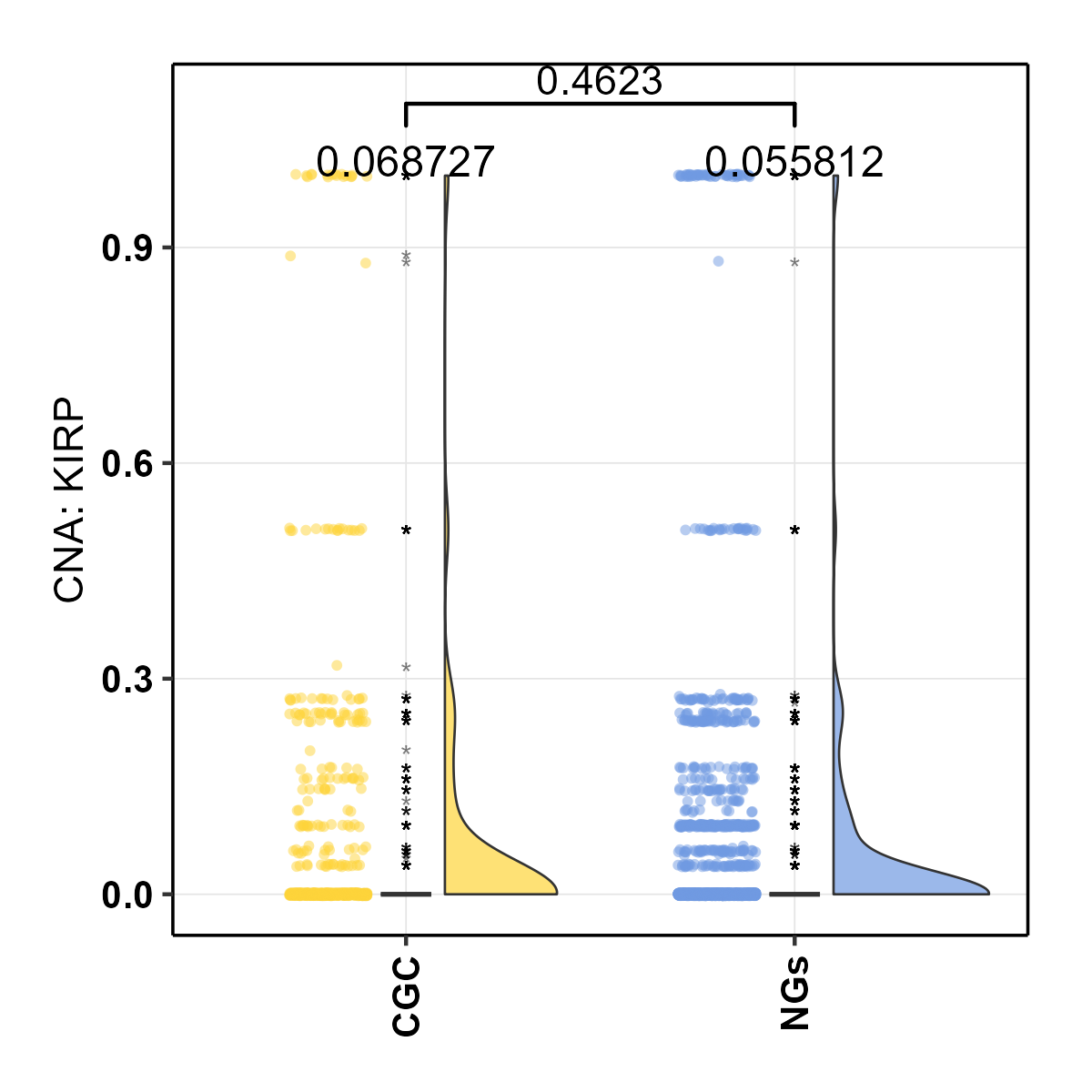

Supplement: Supplementary file 3 [file DataSheet1.ZIP › Supplementary file 5-1/IReflndex/CNA_KIRP.png]

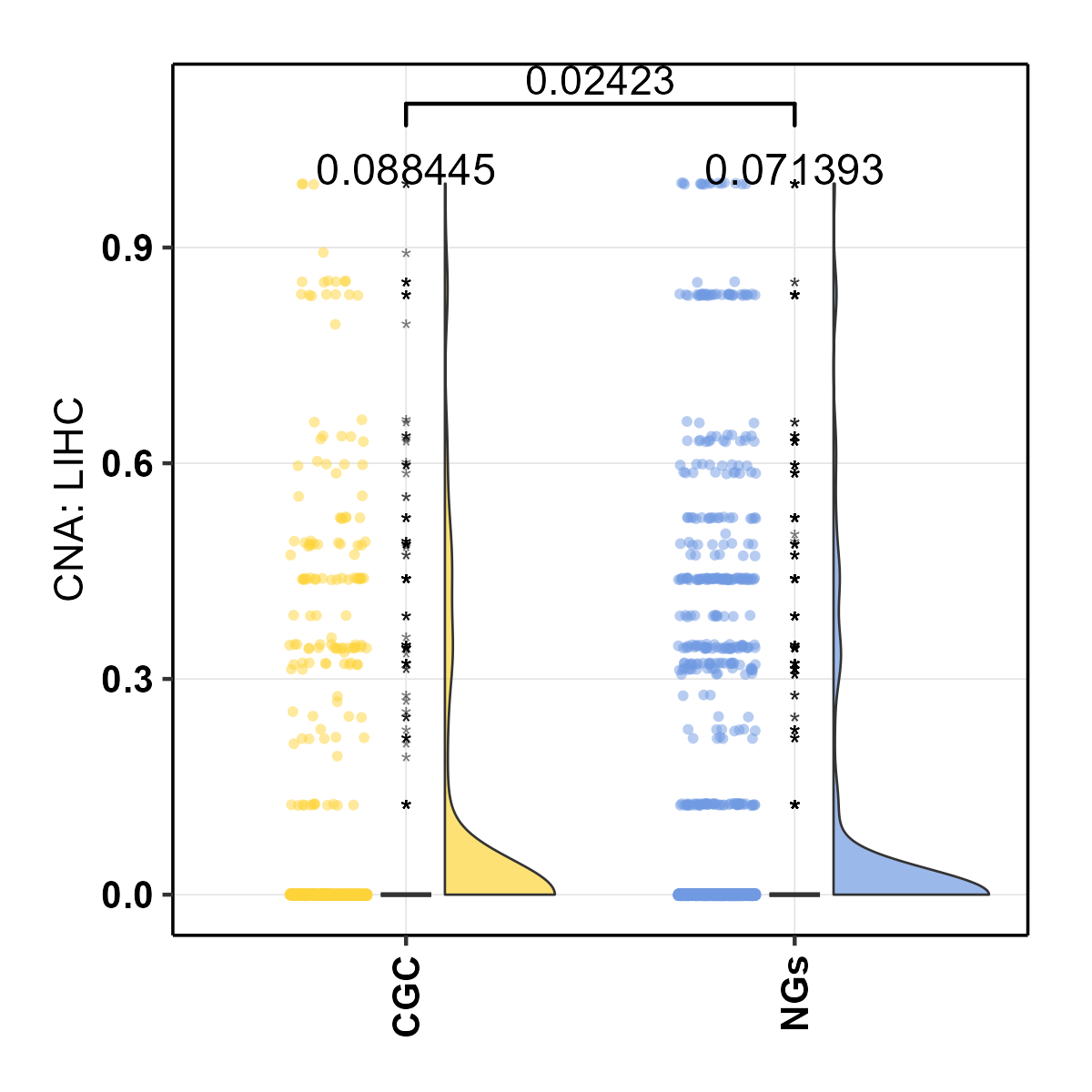

Supplement: Supplementary file 3 [file DataSheet1.ZIP › Supplementary file 5-1/IReflndex/CNA_LIHC.png]

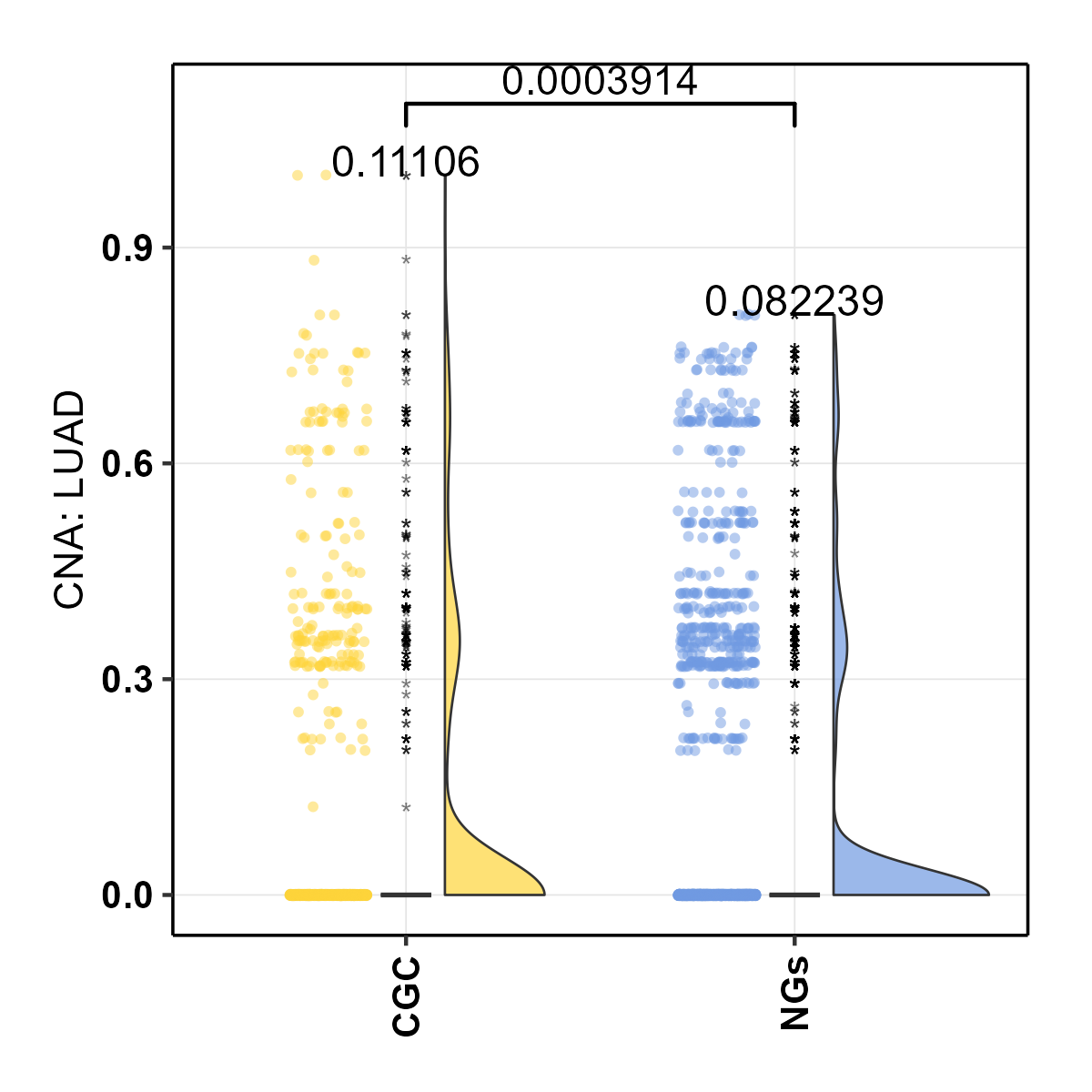

Supplement: Supplementary file 3 [file DataSheet1.ZIP › Supplementary file 5-1/IReflndex/CNA_LUAD.png]

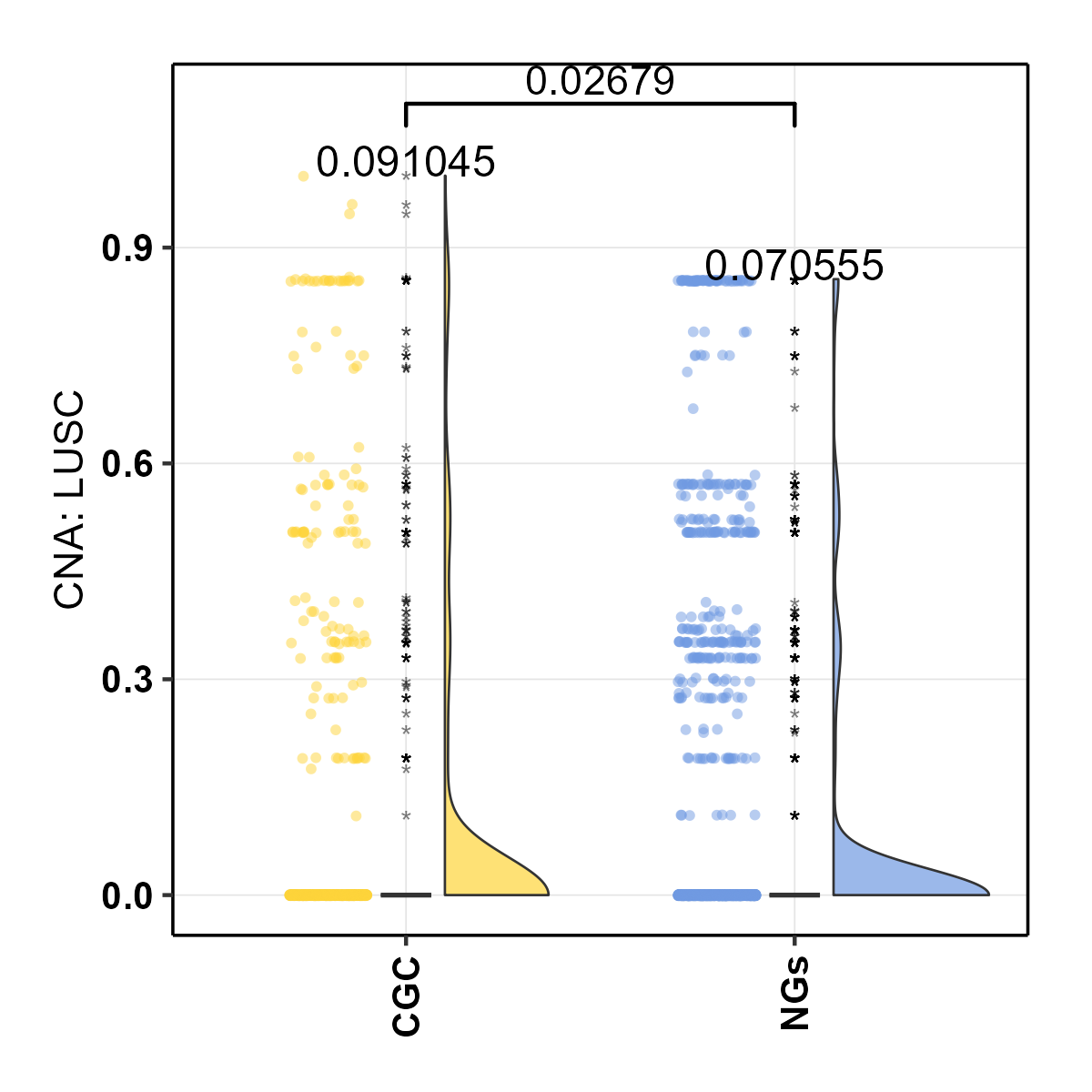

Supplement: Supplementary file 3 [file DataSheet1.ZIP › Supplementary file 5-1/IReflndex/CNA_LUSC.png]

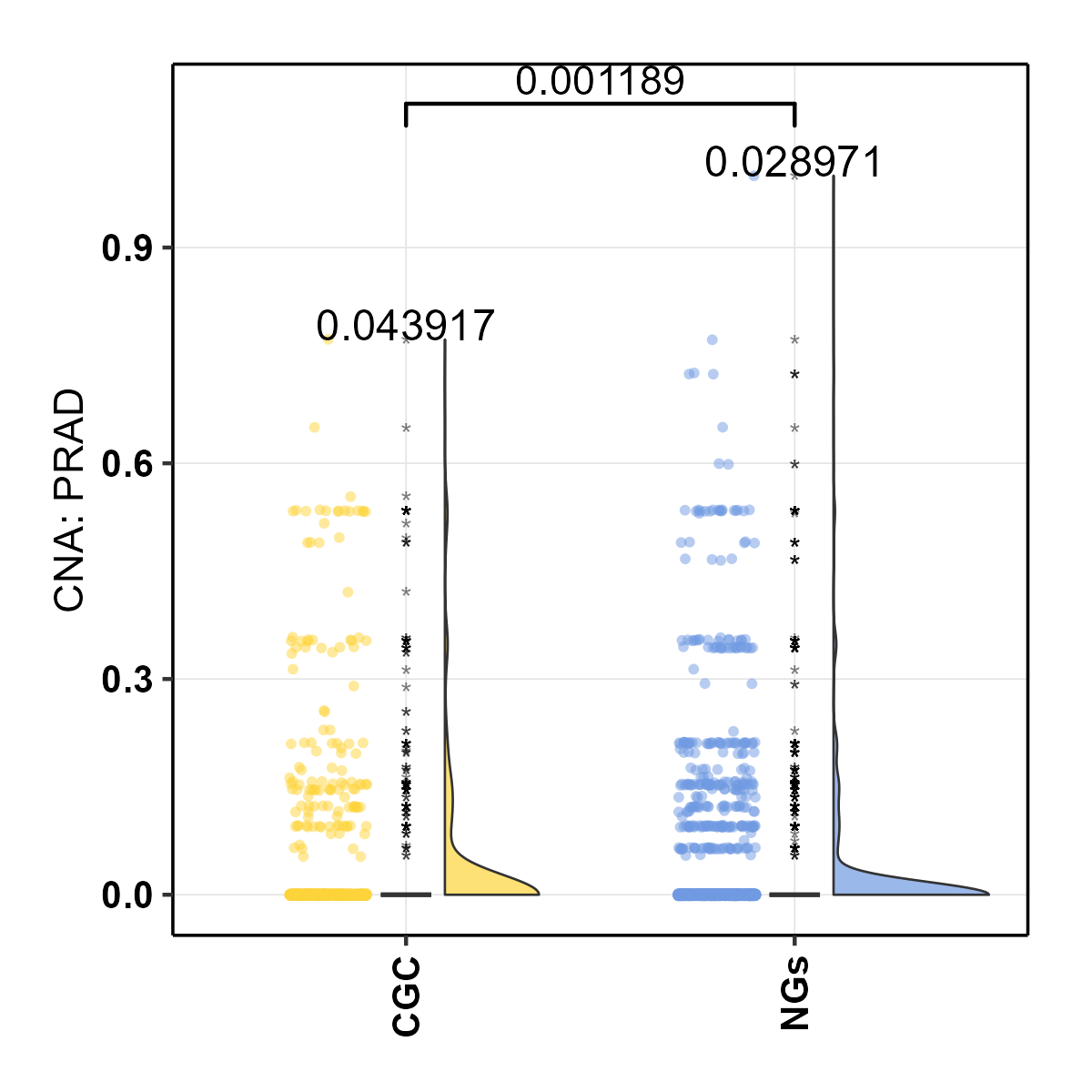

Supplement: Supplementary file 3 [file DataSheet1.ZIP › Supplementary file 5-1/IReflndex/CNA_PRAD.png]

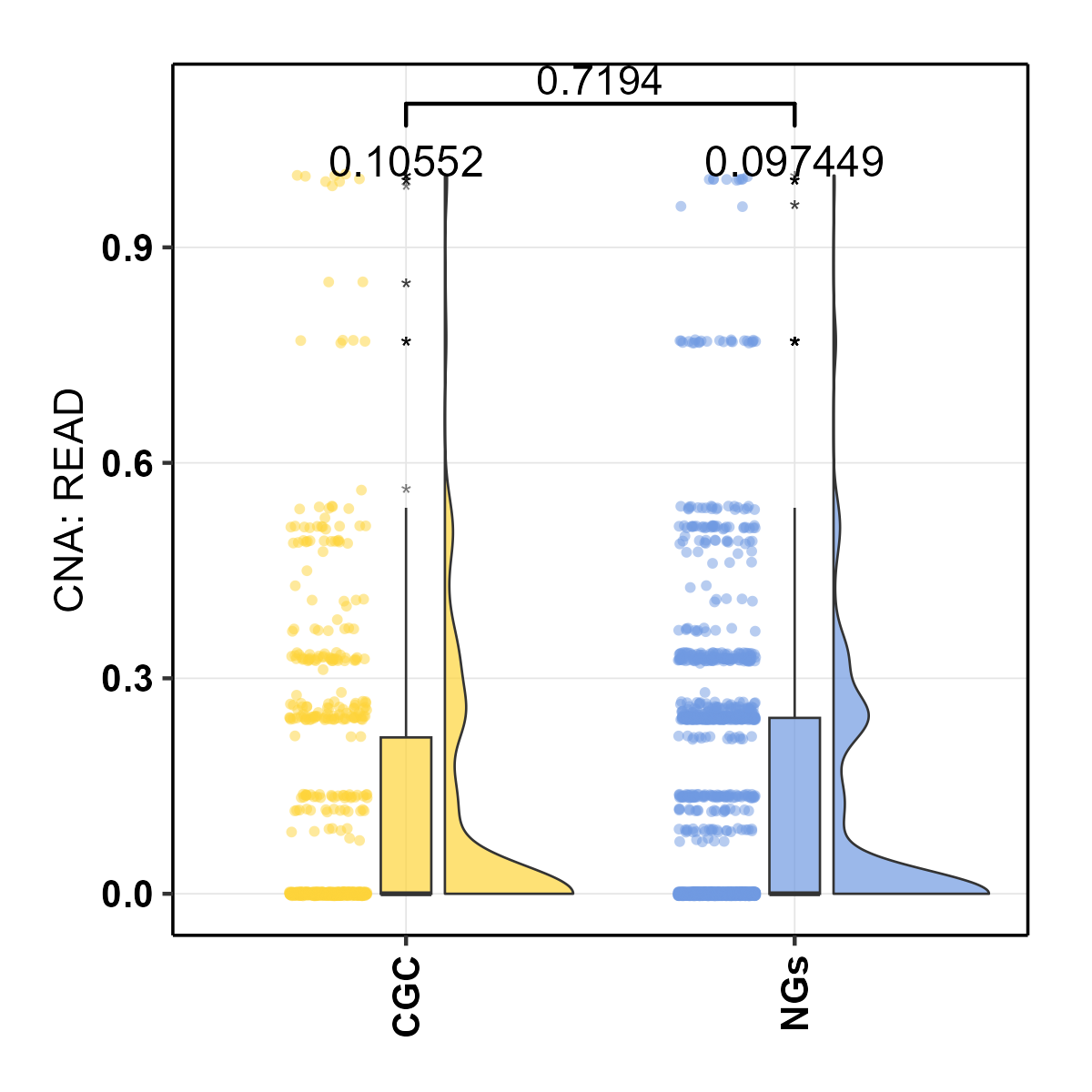

Supplement: Supplementary file 3 [file DataSheet1.ZIP › Supplementary file 5-1/IReflndex/CNA_READ.png]

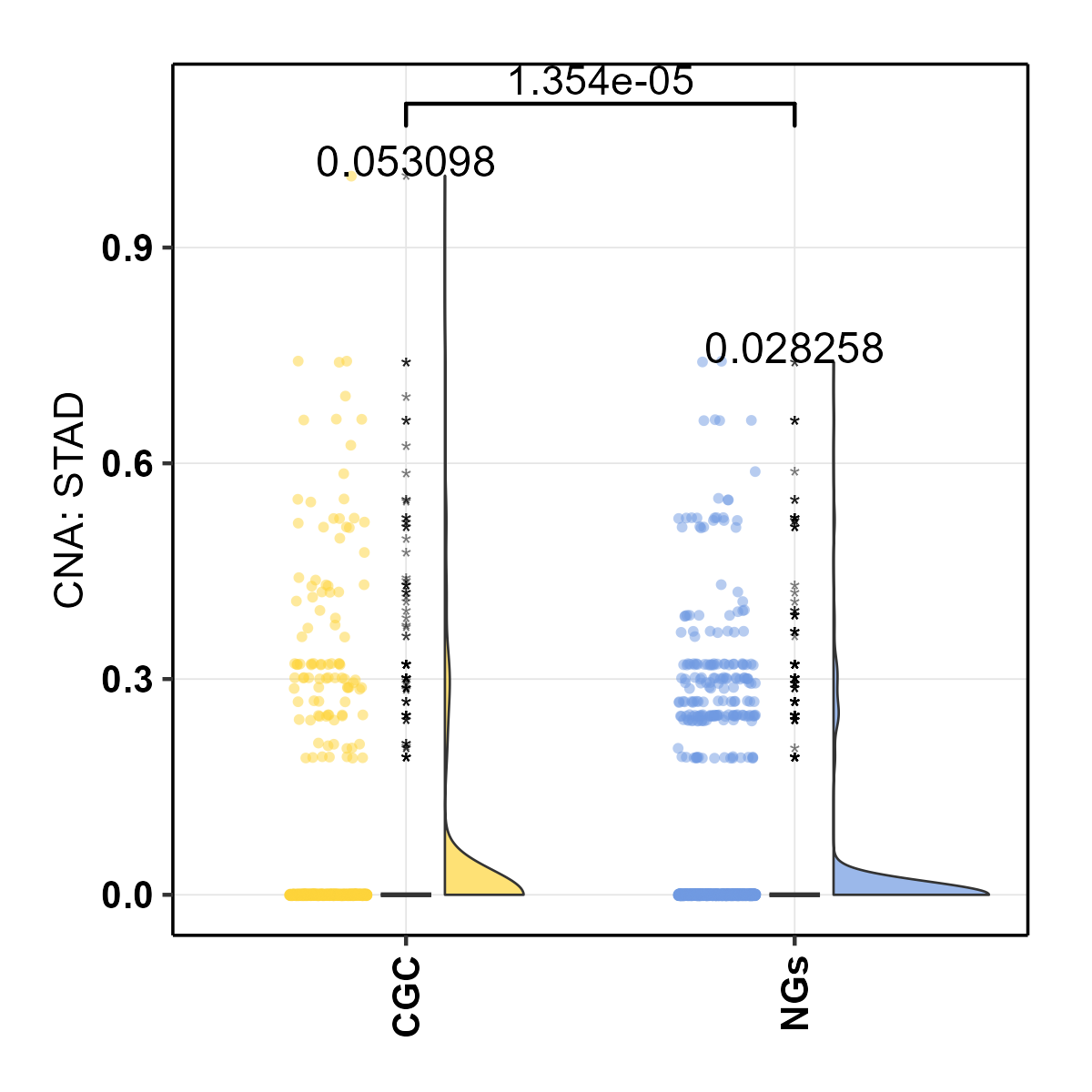

Supplement: Supplementary file 3 [file DataSheet1.ZIP › Supplementary file 5-1/IReflndex/CNA_STAD.png]

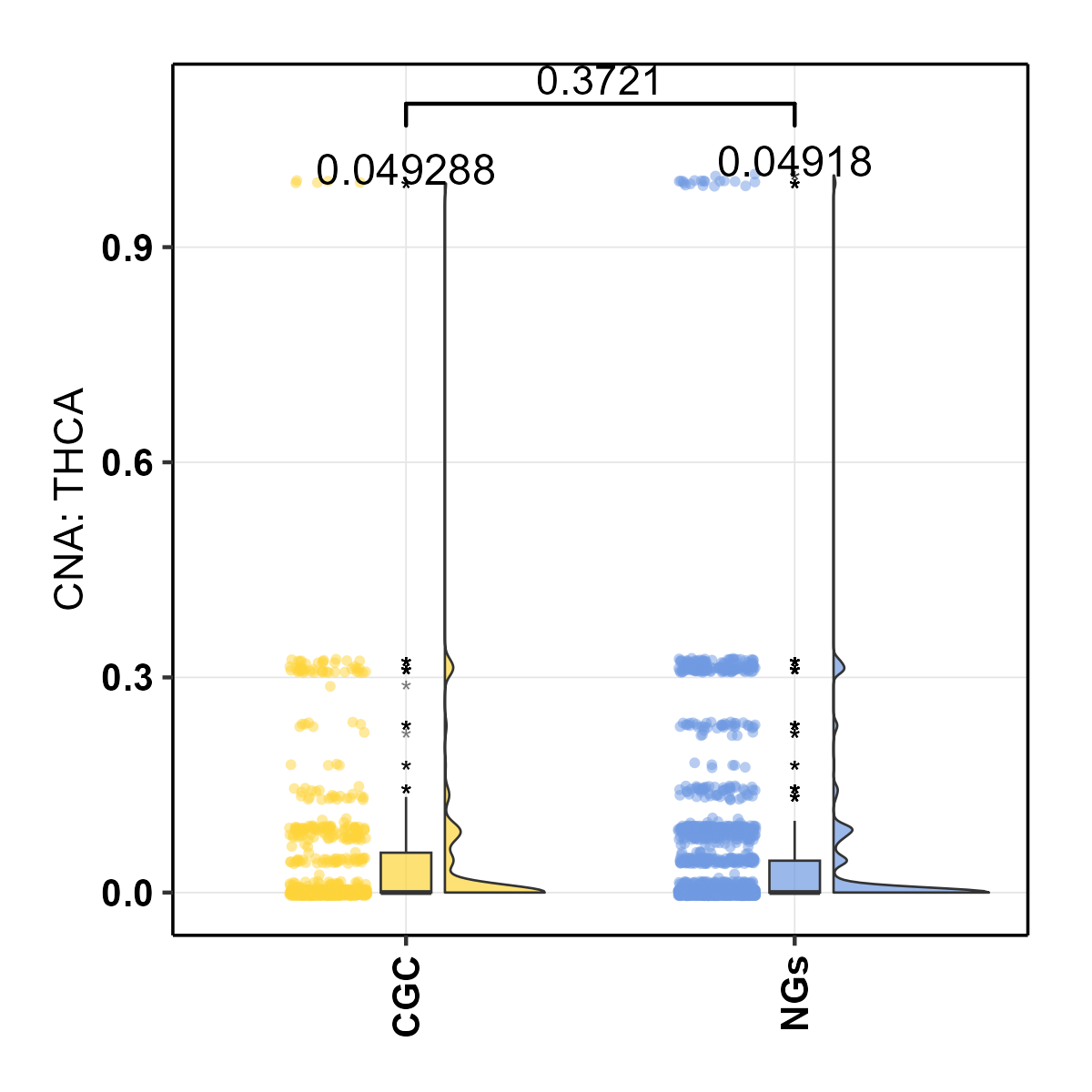

Supplement: Supplementary file 3 [file DataSheet1.ZIP › Supplementary file 5-1/IReflndex/CNA_THCA.png]

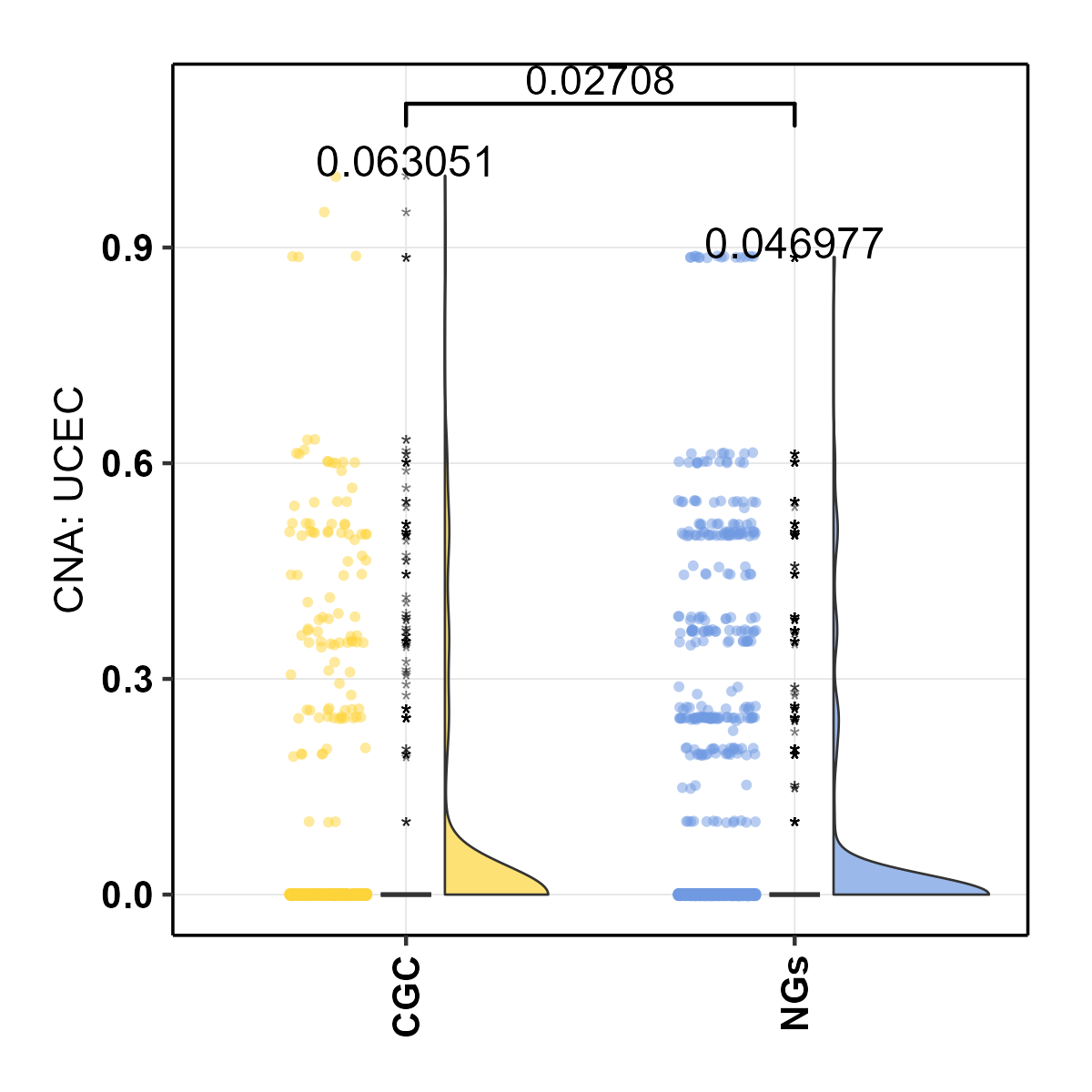

Supplement: Supplementary file 3 [file DataSheet1.ZIP › Supplementary file 5-1/IReflndex/CNA_UCEC.png]

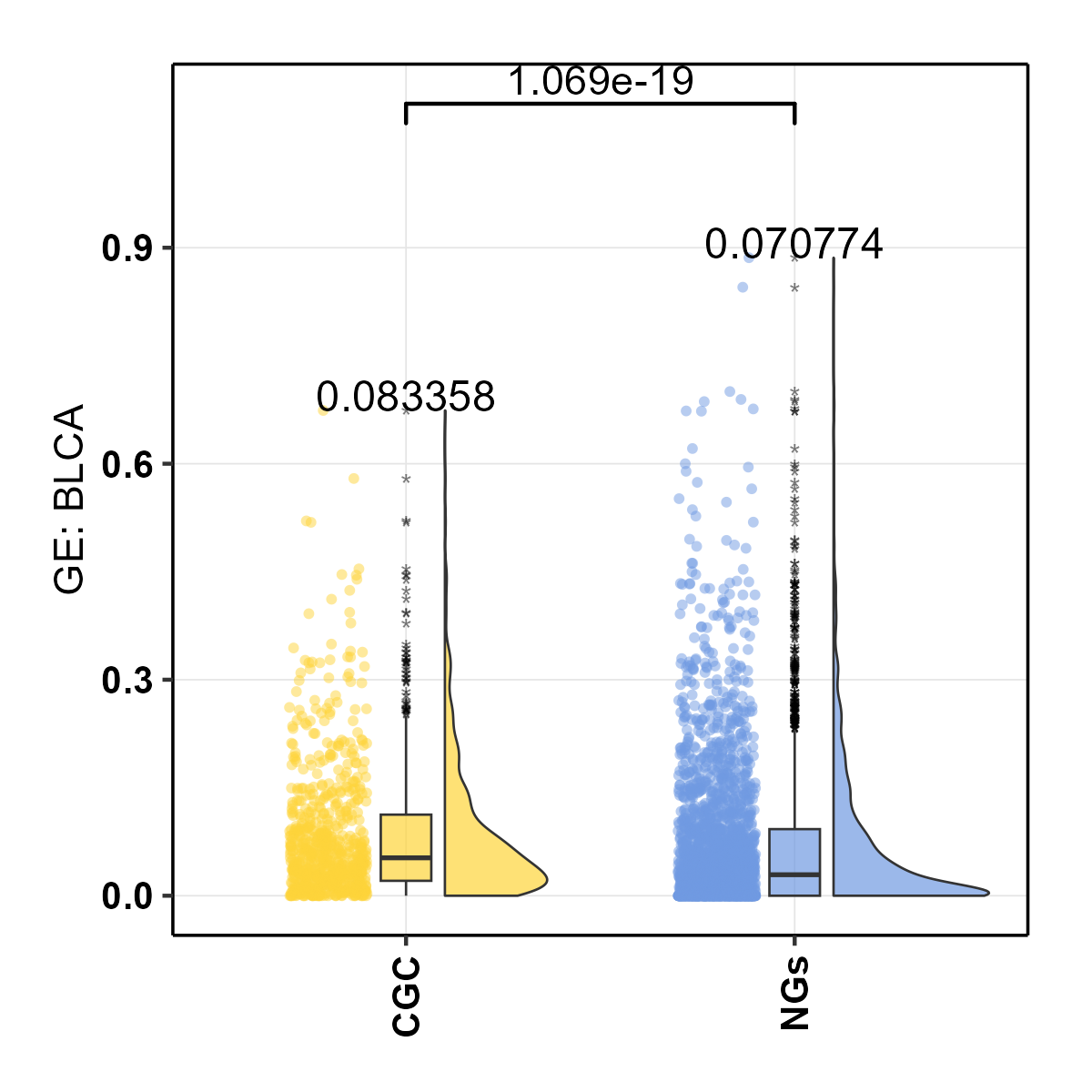

Supplement: Supplementary file 3 [file DataSheet1.ZIP › Supplementary file 5-1/IReflndex/GE_BLCA.png]

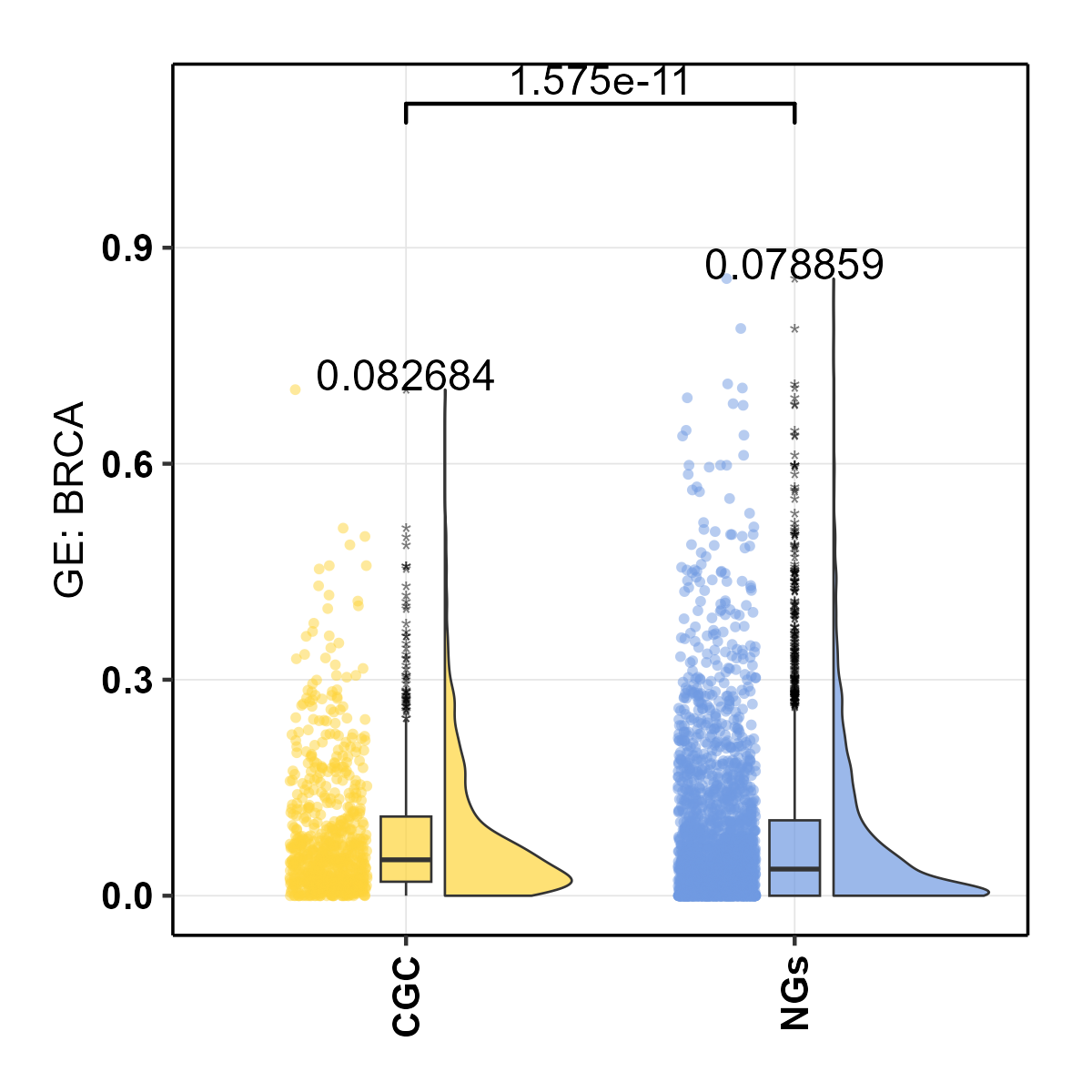

Supplement: Supplementary file 3 [file DataSheet1.ZIP › Supplementary file 5-1/IReflndex/GE_BRCA.png]

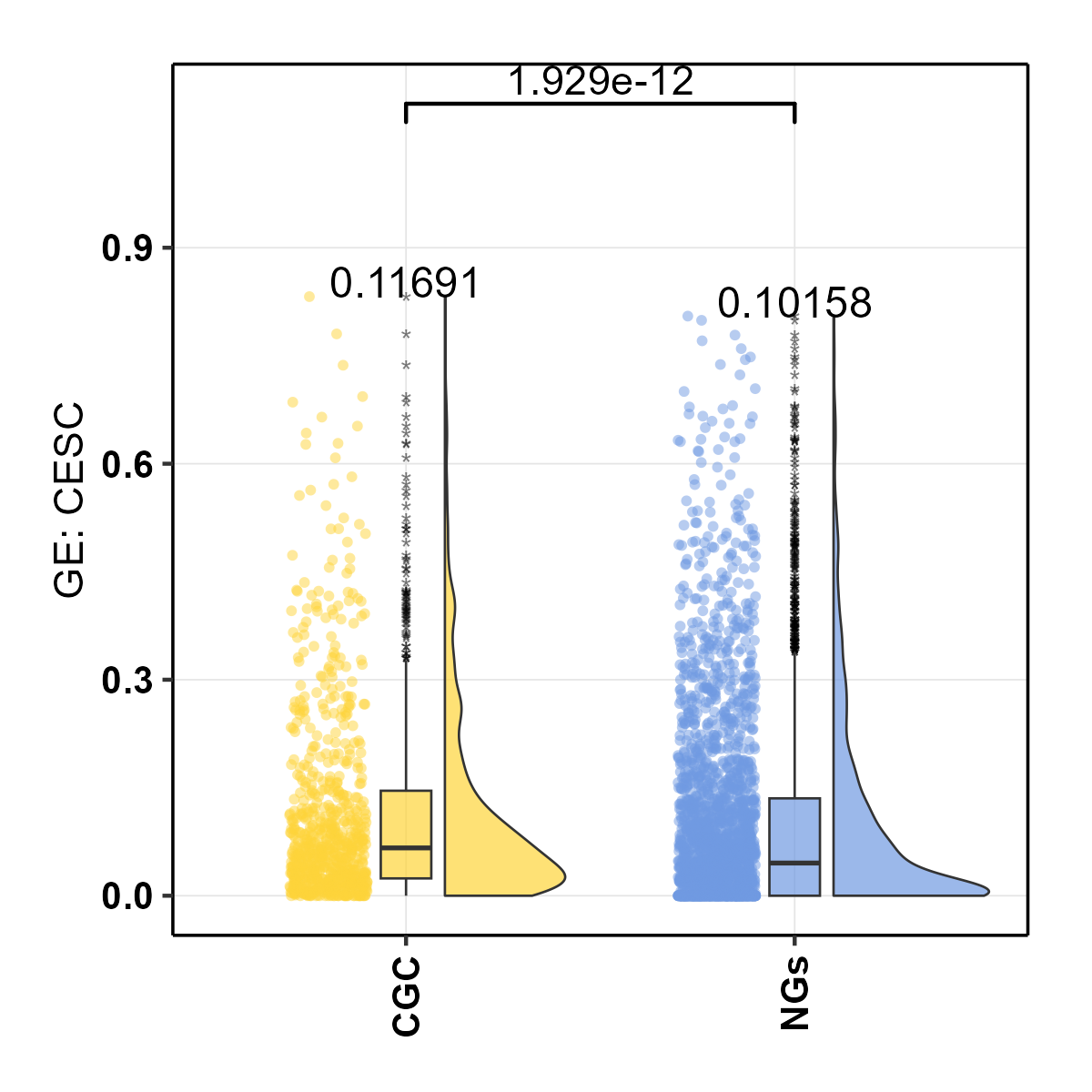

Supplement: Supplementary file 3 [file DataSheet1.ZIP › Supplementary file 5-1/IReflndex/GE_CESC.png]

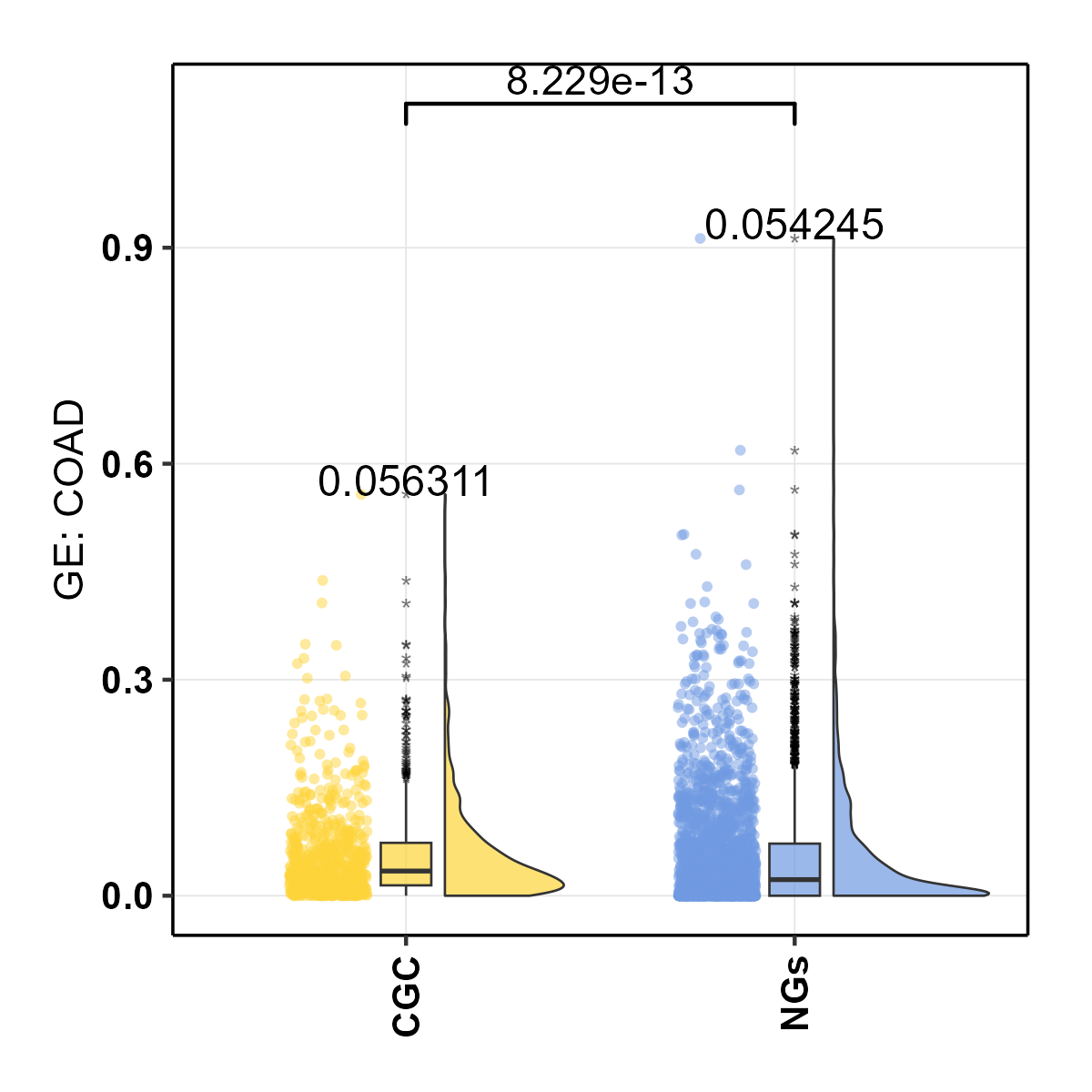

Supplement: Supplementary file 3 [file DataSheet1.ZIP › Supplementary file 5-1/IReflndex/GE_COAD.png]

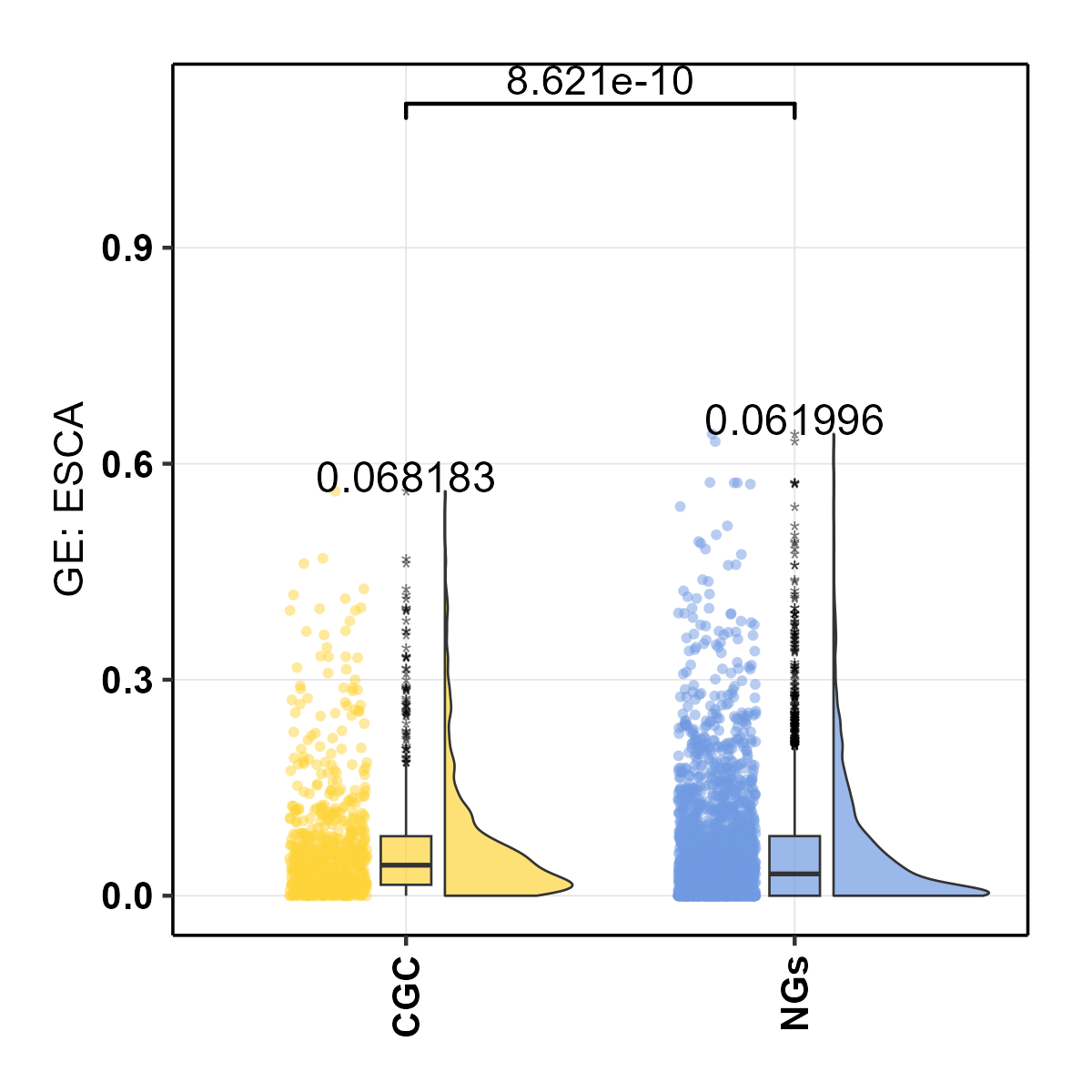

Supplement: Supplementary file 3 [file DataSheet1.ZIP › Supplementary file 5-1/IReflndex/GE_ESCA.png]

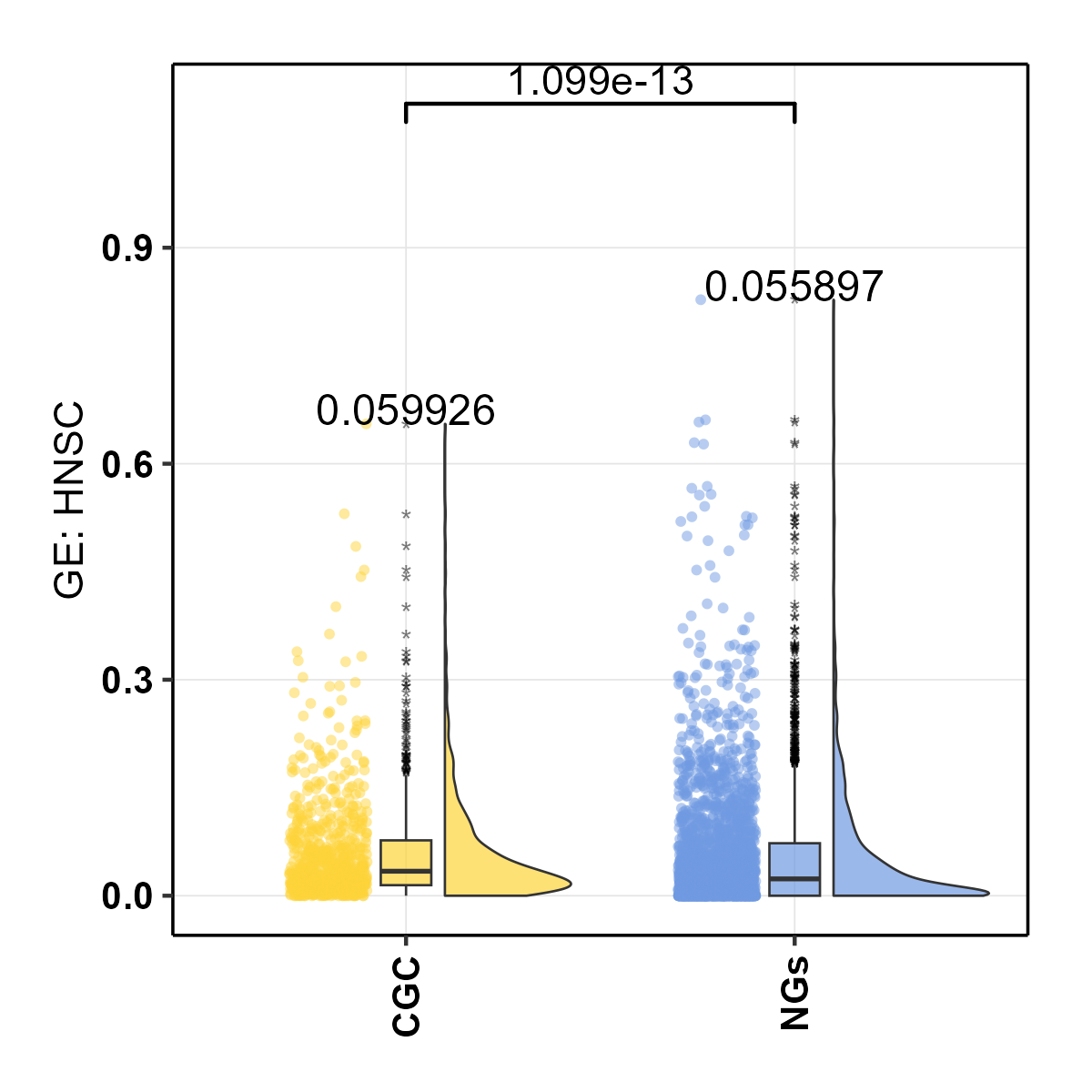

Supplement: Supplementary file 3 [file DataSheet1.ZIP › Supplementary file 5-1/IReflndex/GE_HNSC.png]

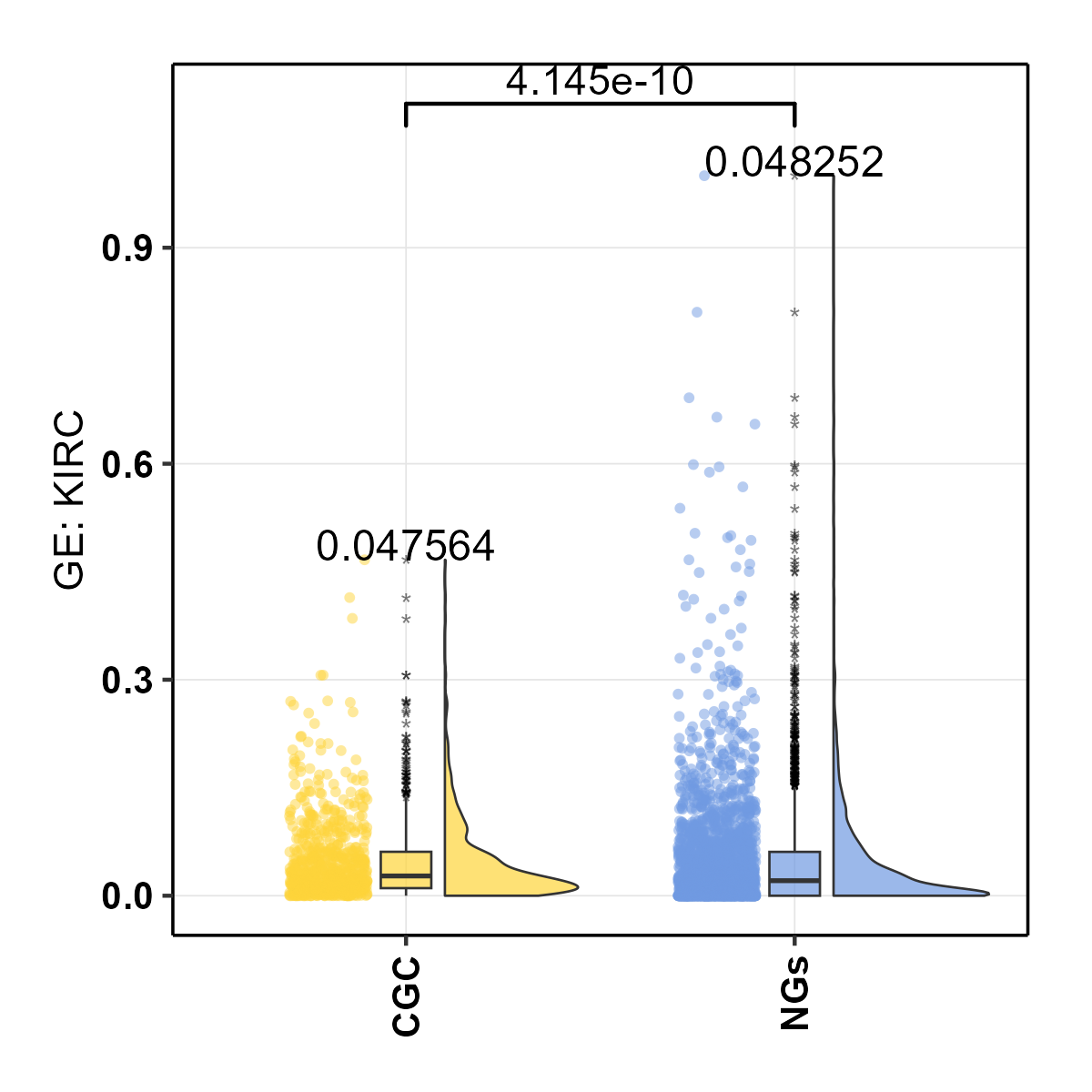

Supplement: Supplementary file 3 [file DataSheet1.ZIP › Supplementary file 5-1/IReflndex/GE_KIRC.png]

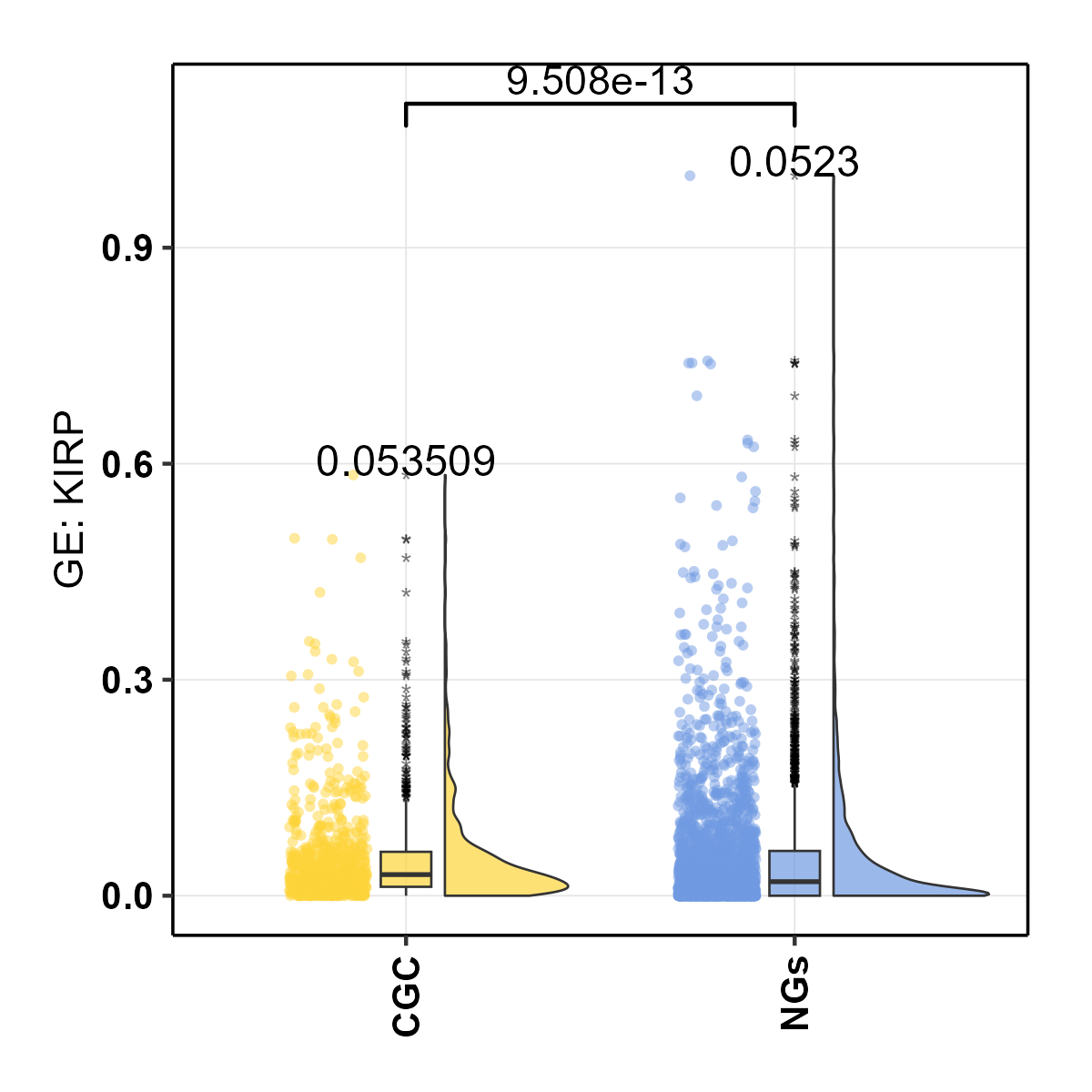

Supplement: Supplementary file 3 [file DataSheet1.ZIP › Supplementary file 5-1/IReflndex/GE_KIRP.png]

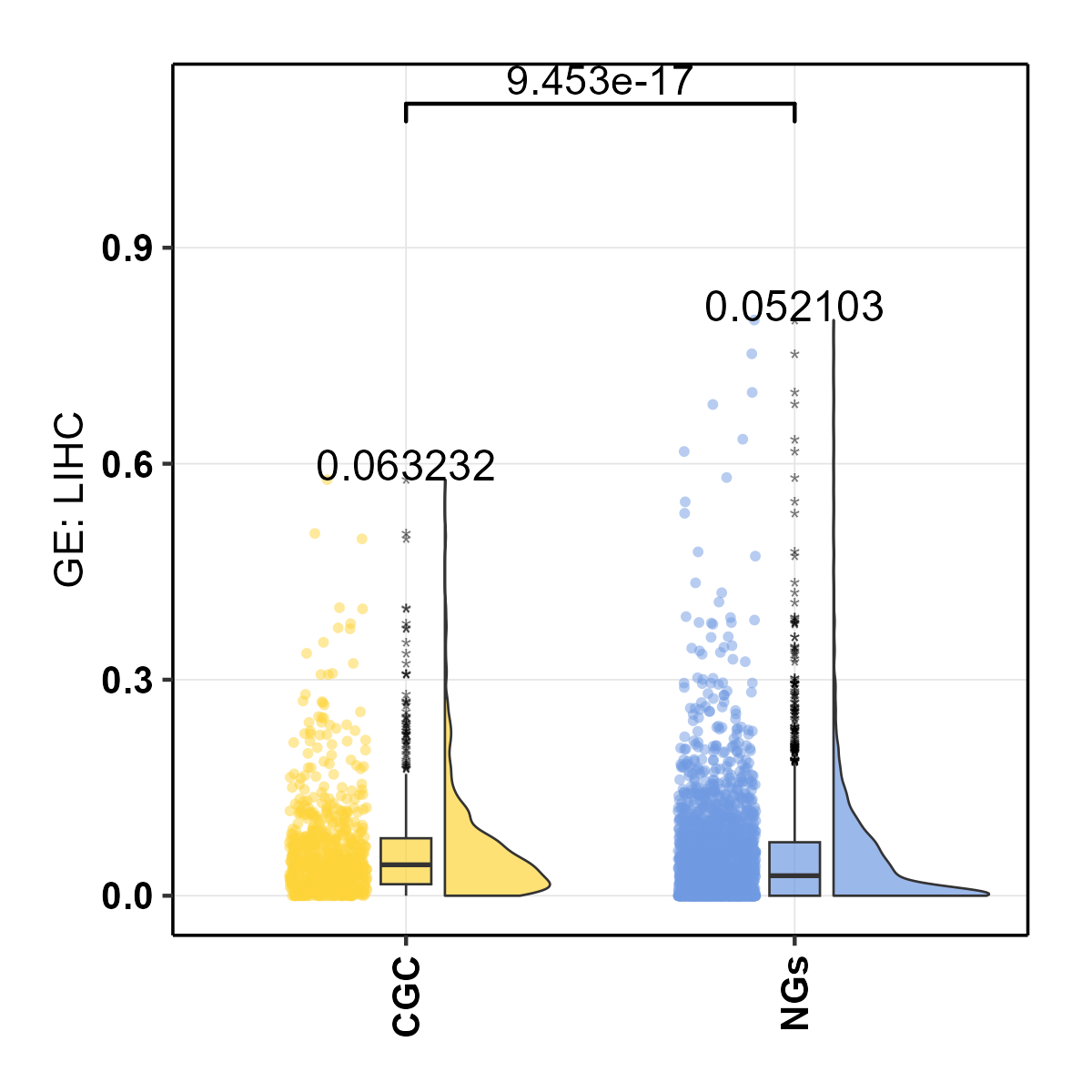

Supplement: Supplementary file 3 [file DataSheet1.ZIP › Supplementary file 5-1/IReflndex/GE_LIHC.png]

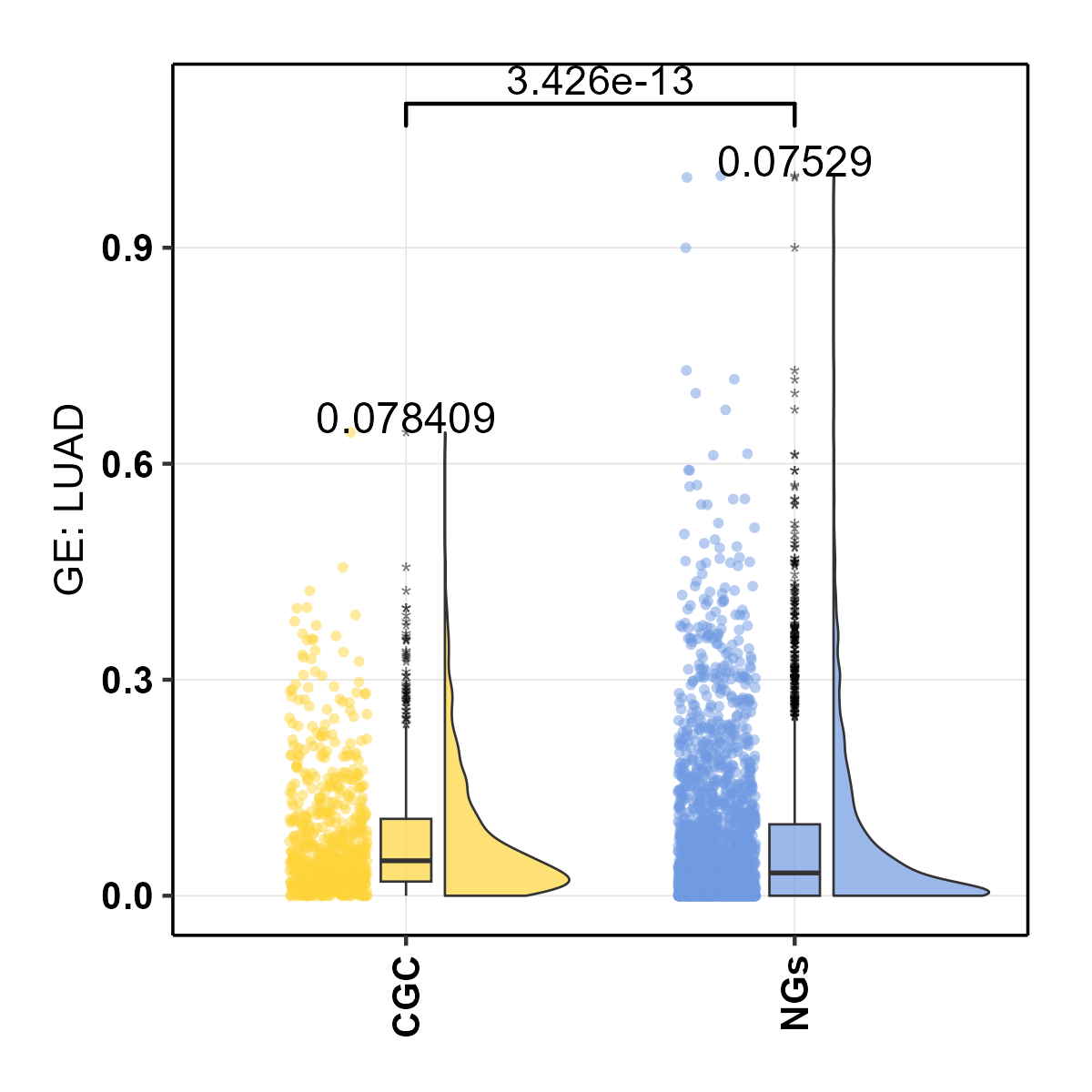

Supplement: Supplementary file 3 [file DataSheet1.ZIP › Supplementary file 5-1/IReflndex/GE_LUAD.png]

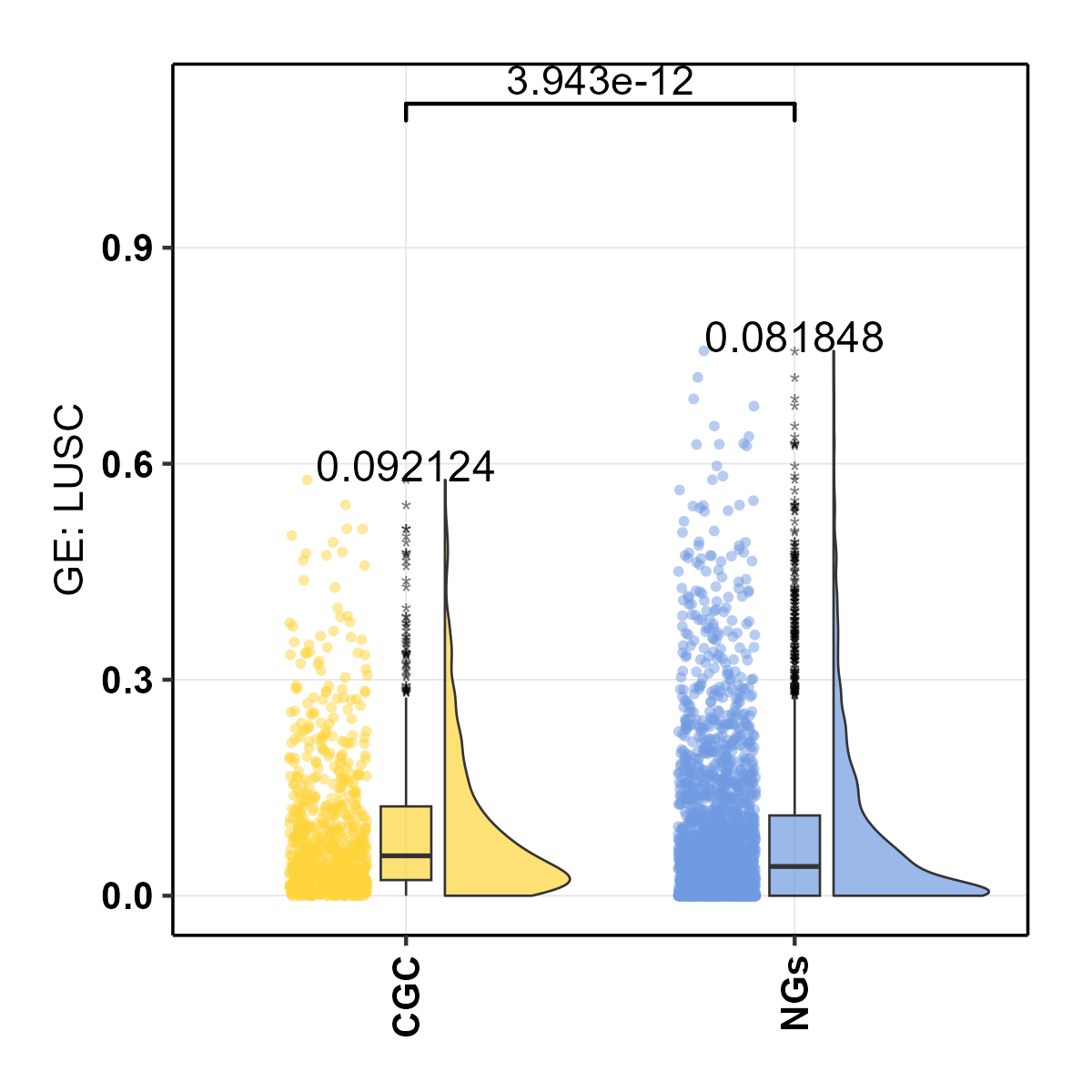

Supplement: Supplementary file 3 [file DataSheet1.ZIP › Supplementary file 5-1/IReflndex/GE_LUSC.png]

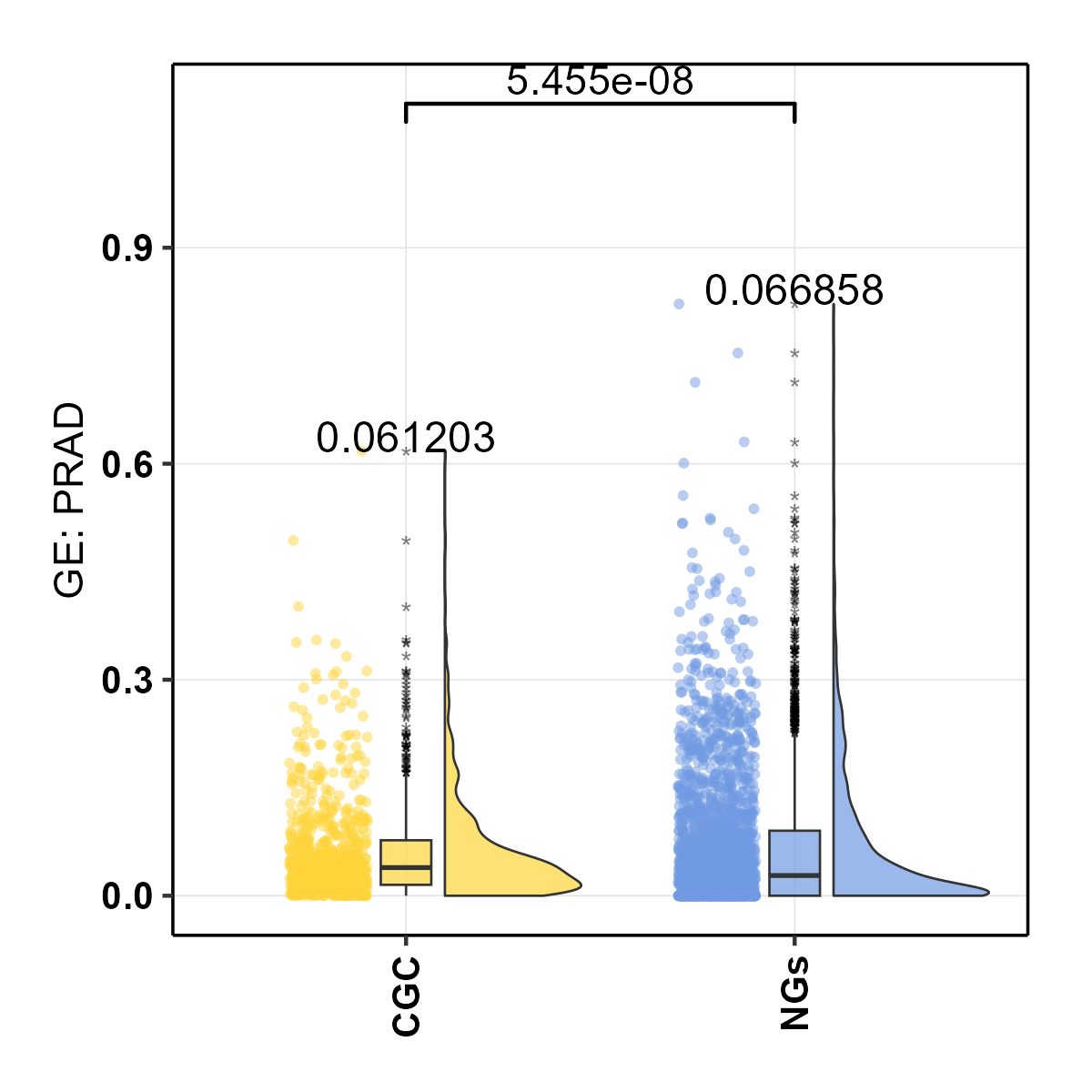

Supplement: Supplementary file 3 [file DataSheet1.ZIP › Supplementary file 5-1/IReflndex/GE_PRAD.png]

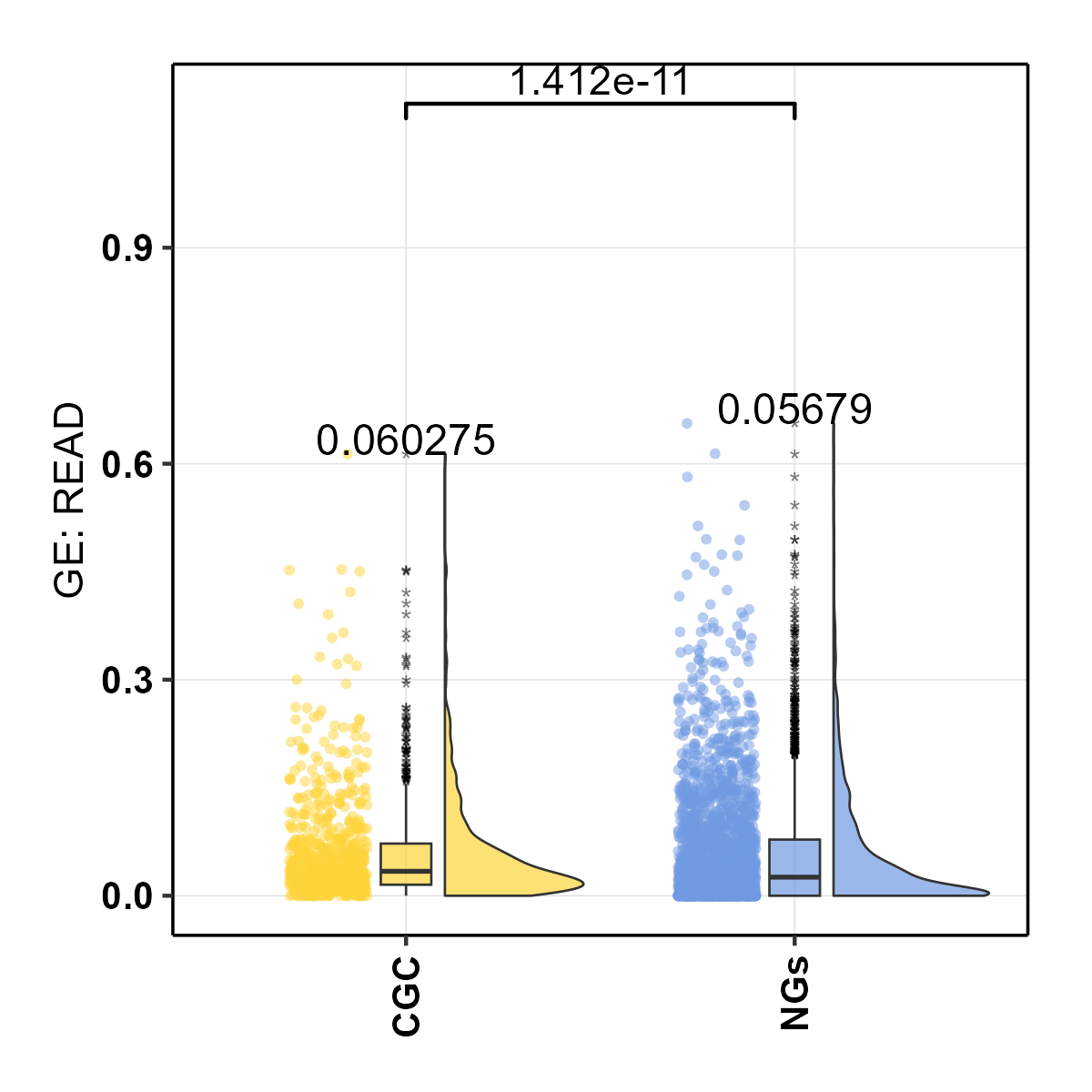

Supplement: Supplementary file 3 [file DataSheet1.ZIP › Supplementary file 5-1/IReflndex/GE_READ.png]

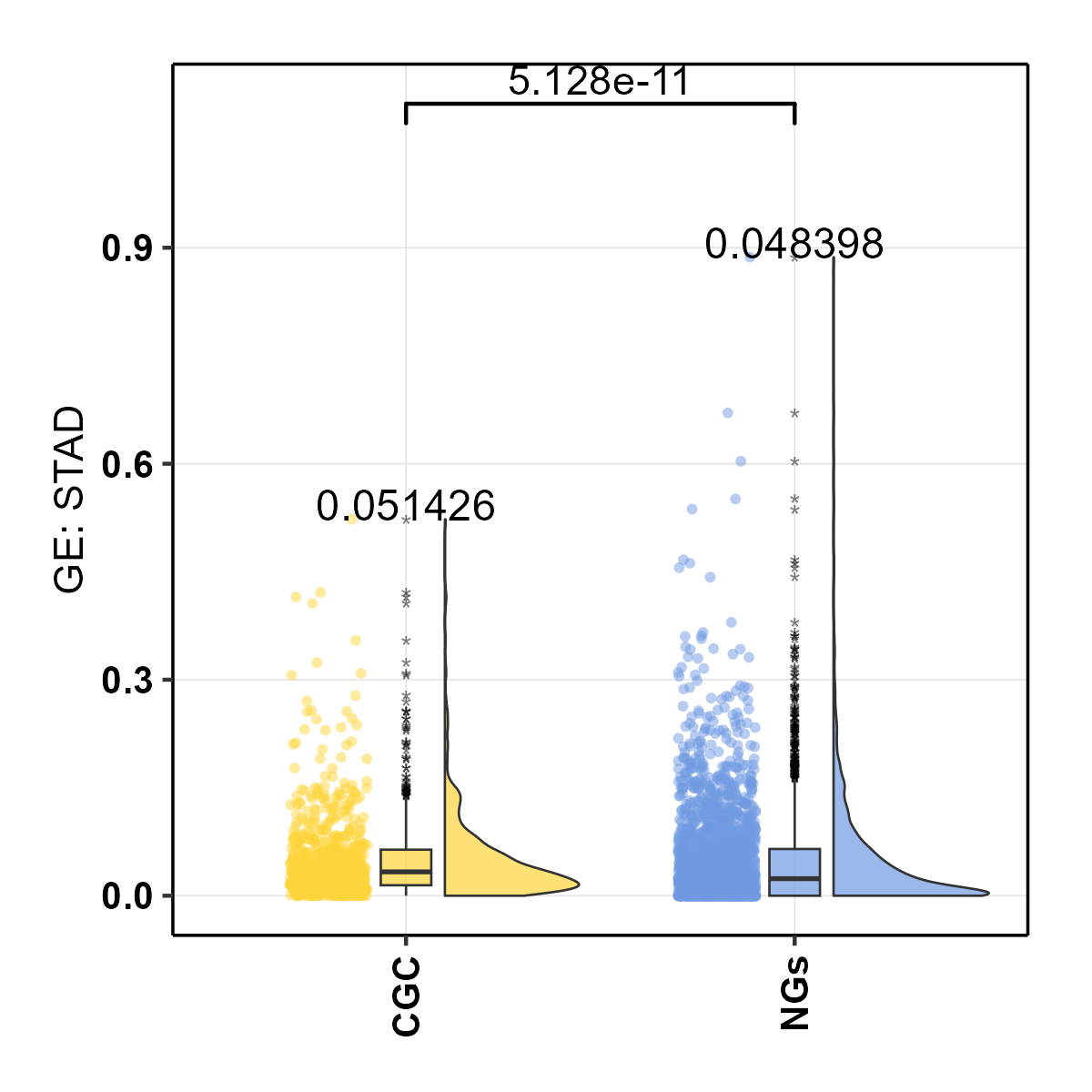

Supplement: Supplementary file 3 [file DataSheet1.ZIP › Supplementary file 5-1/IReflndex/GE_STAD.png]

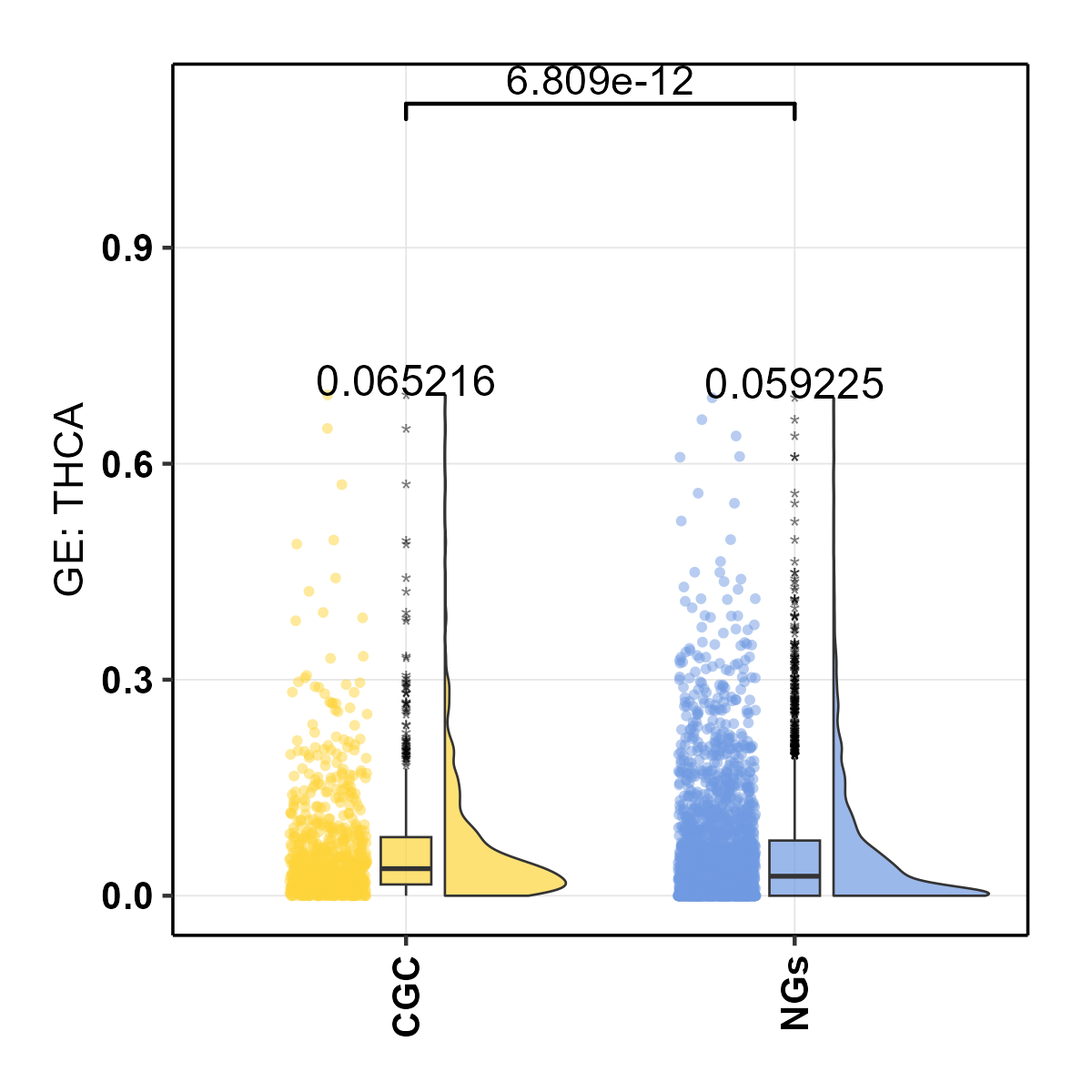

Supplement: Supplementary file 3 [file DataSheet1.ZIP › Supplementary file 5-1/IReflndex/GE_THCA.png]

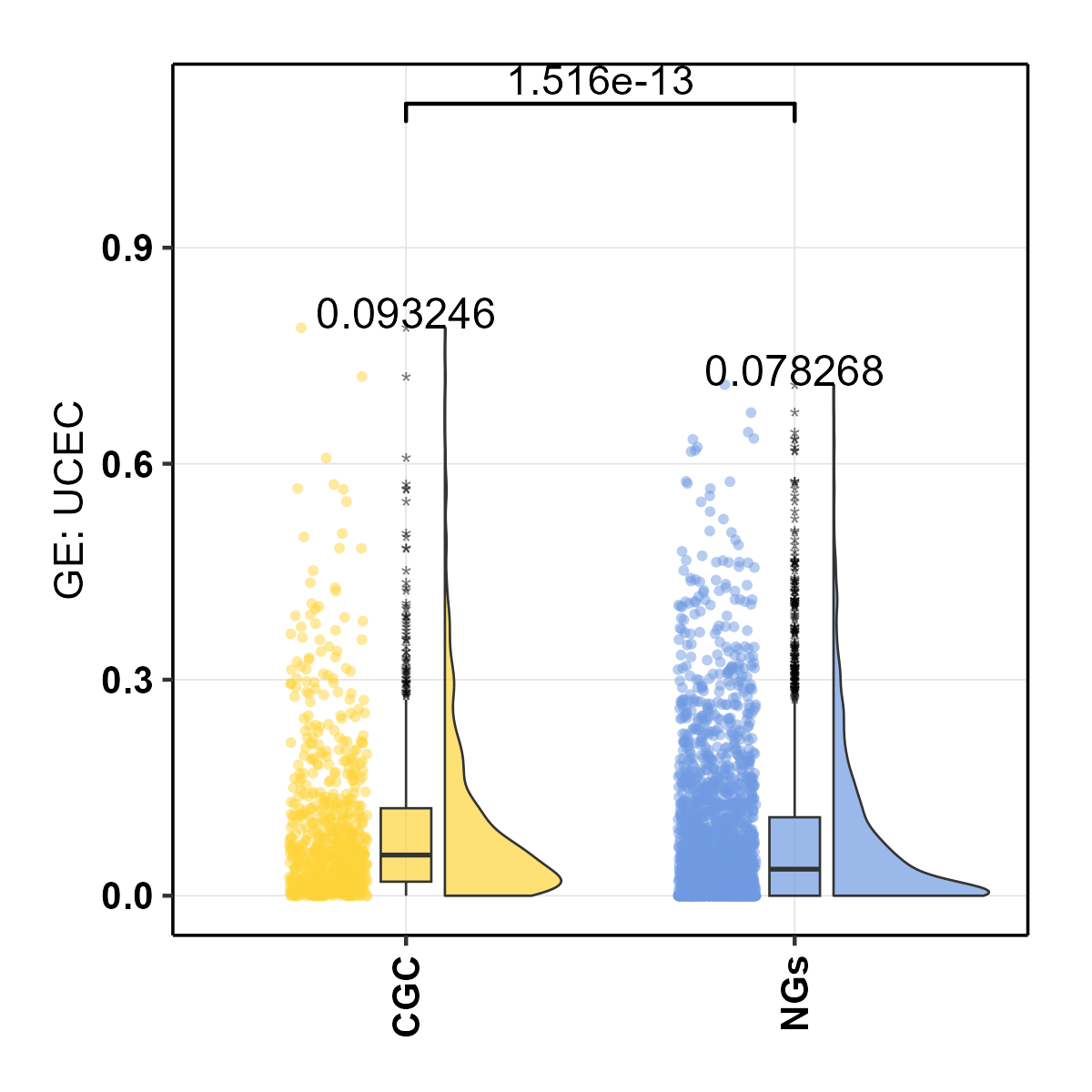

Supplement: Supplementary file 3 [file DataSheet1.ZIP › Supplementary file 5-1/IReflndex/GE_UCEC.png]

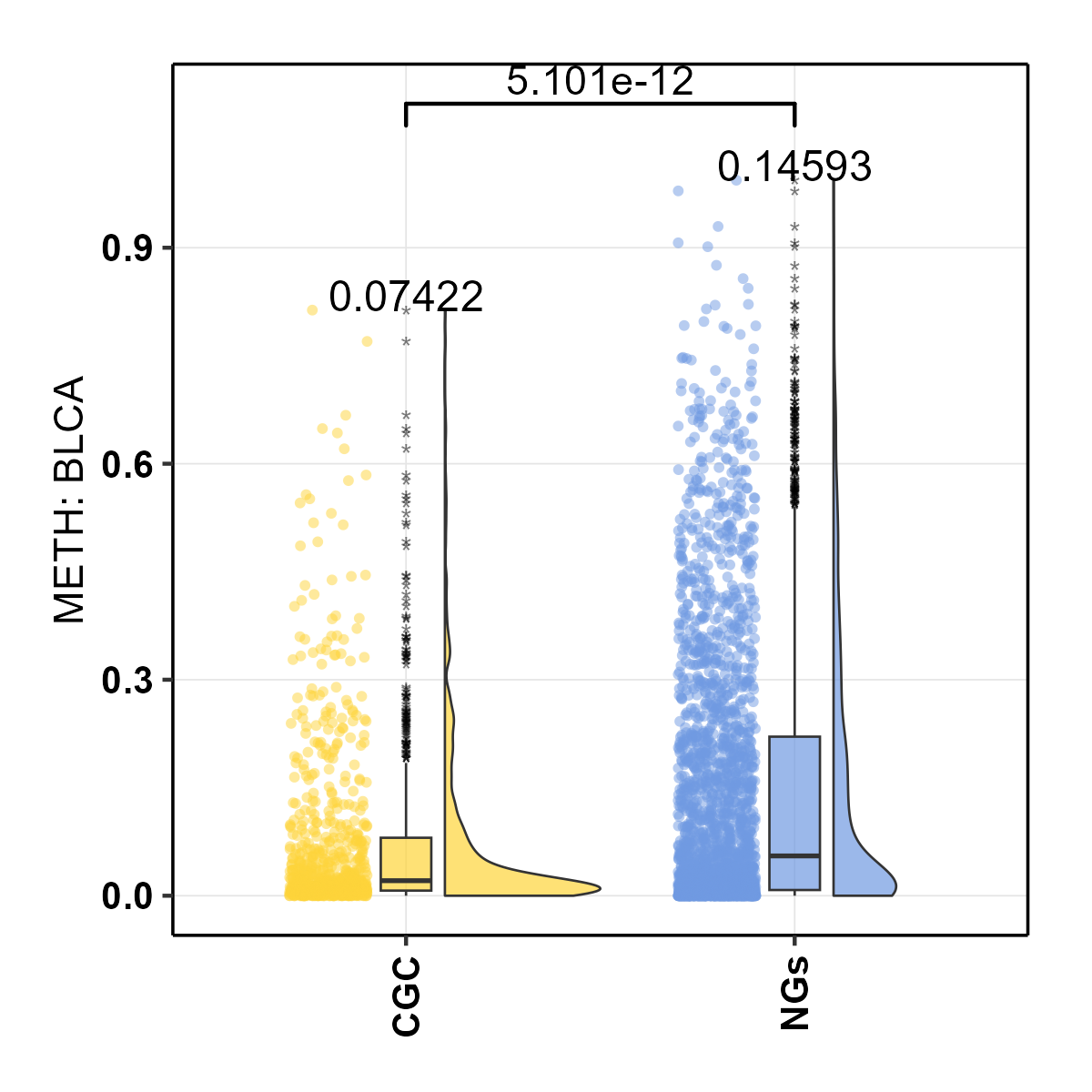

Supplement: Supplementary file 3 [file DataSheet1.ZIP › Supplementary file 5-1/IReflndex/METH_BLCA.png]

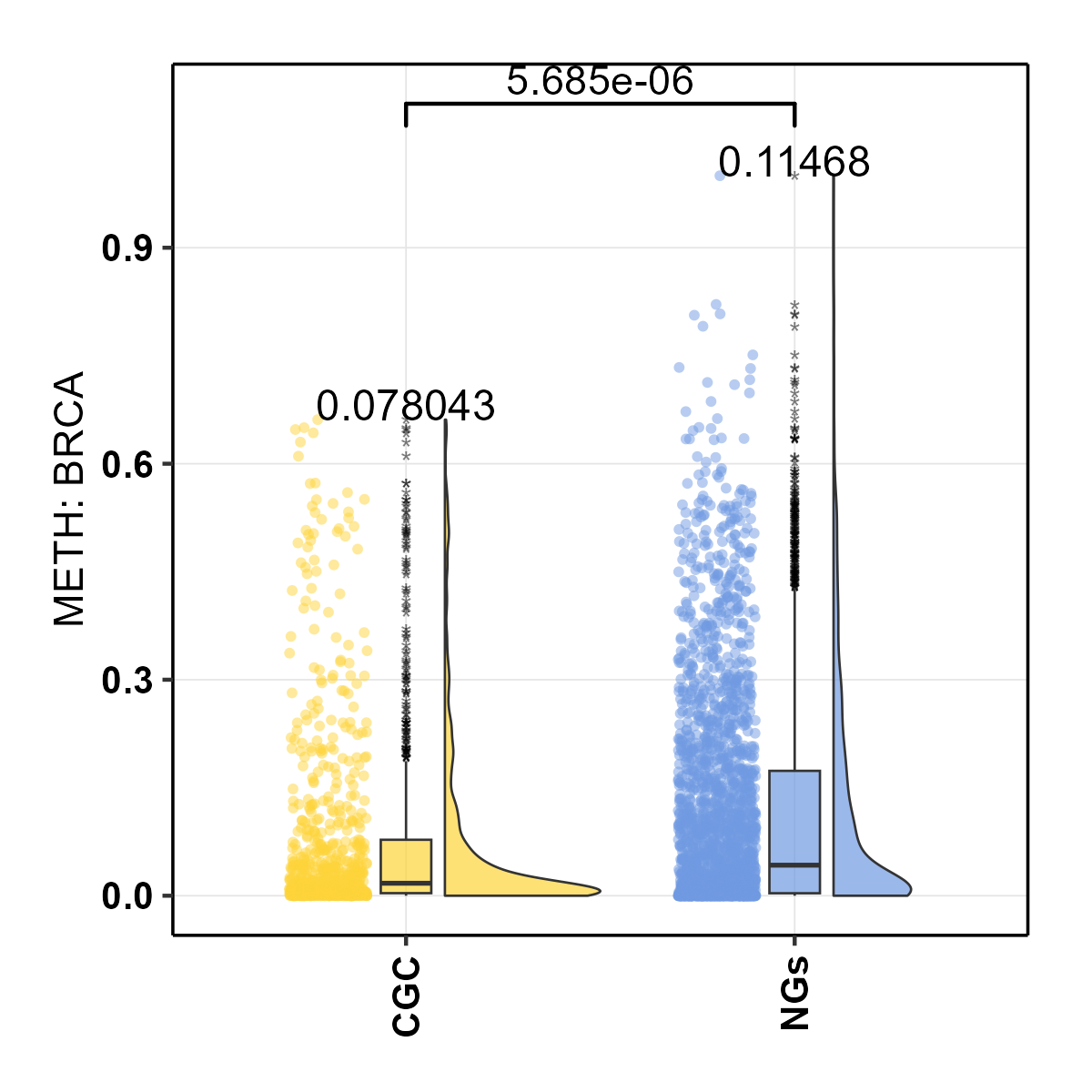

Supplement: Supplementary file 3 [file DataSheet1.ZIP › Supplementary file 5-1/IReflndex/METH_BRCA.png]

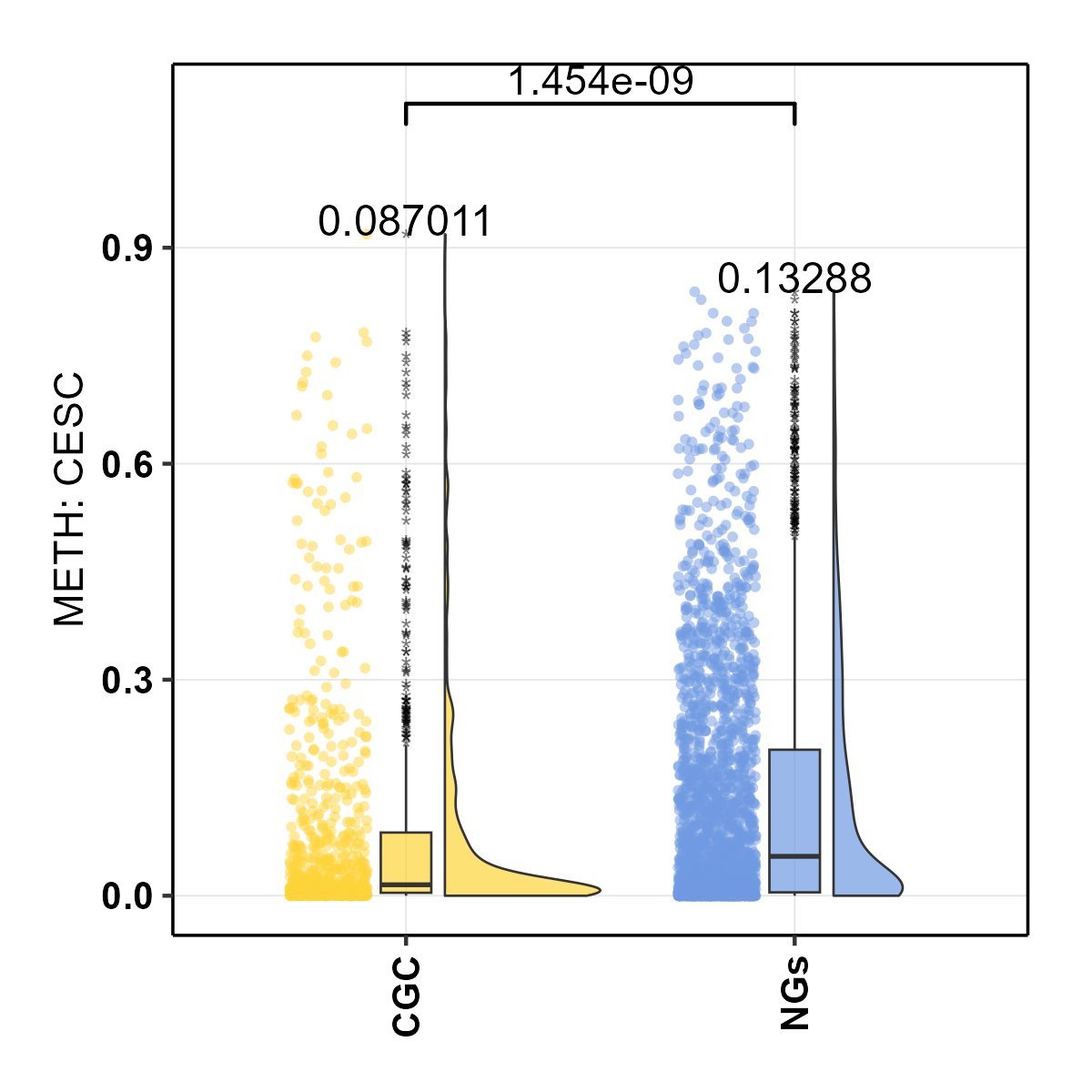

Supplement: Supplementary file 3 [file DataSheet1.ZIP › Supplementary file 5-1/IReflndex/METH_CESC.png]

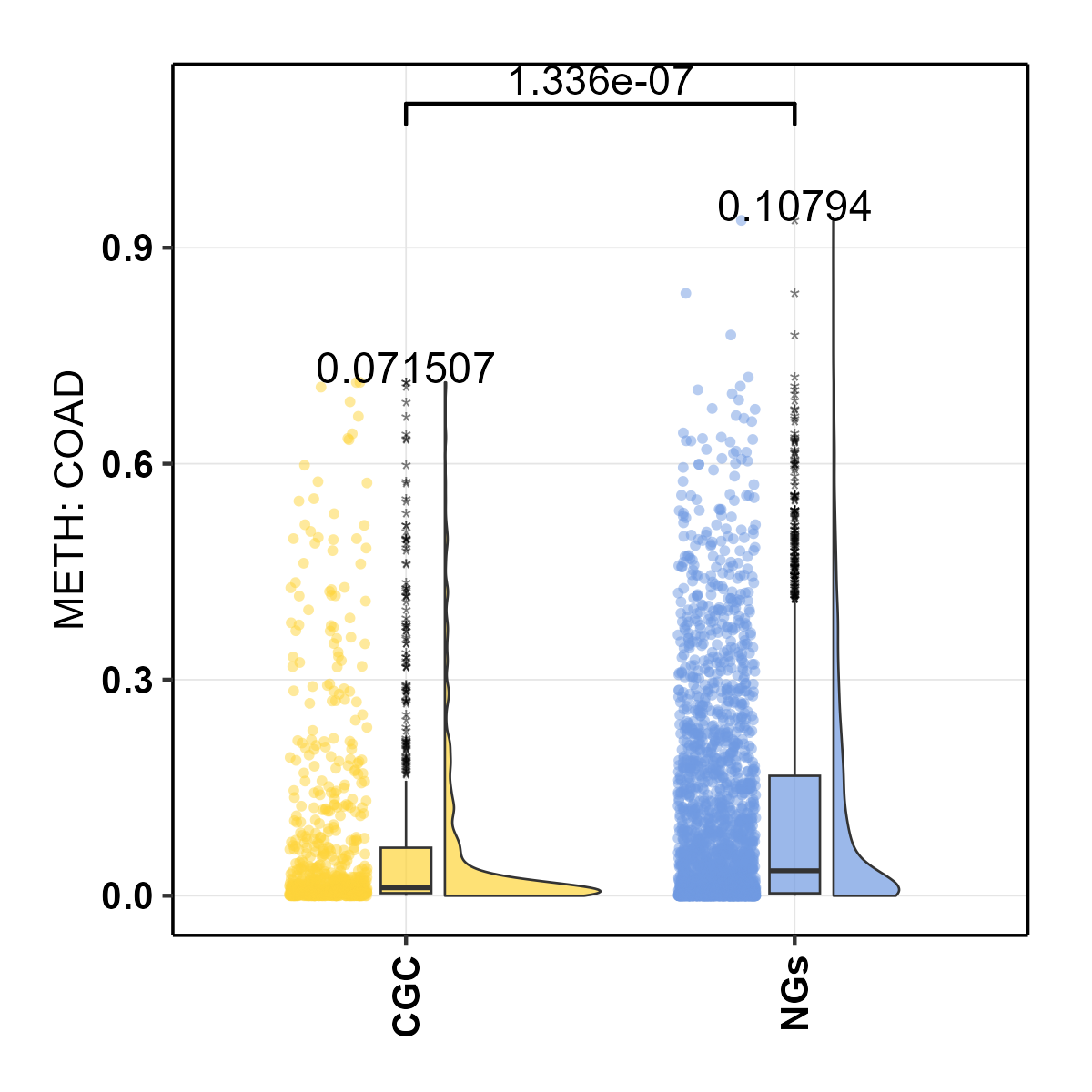

Supplement: Supplementary file 3 [file DataSheet1.ZIP › Supplementary file 5-1/IReflndex/METH_COAD.png]

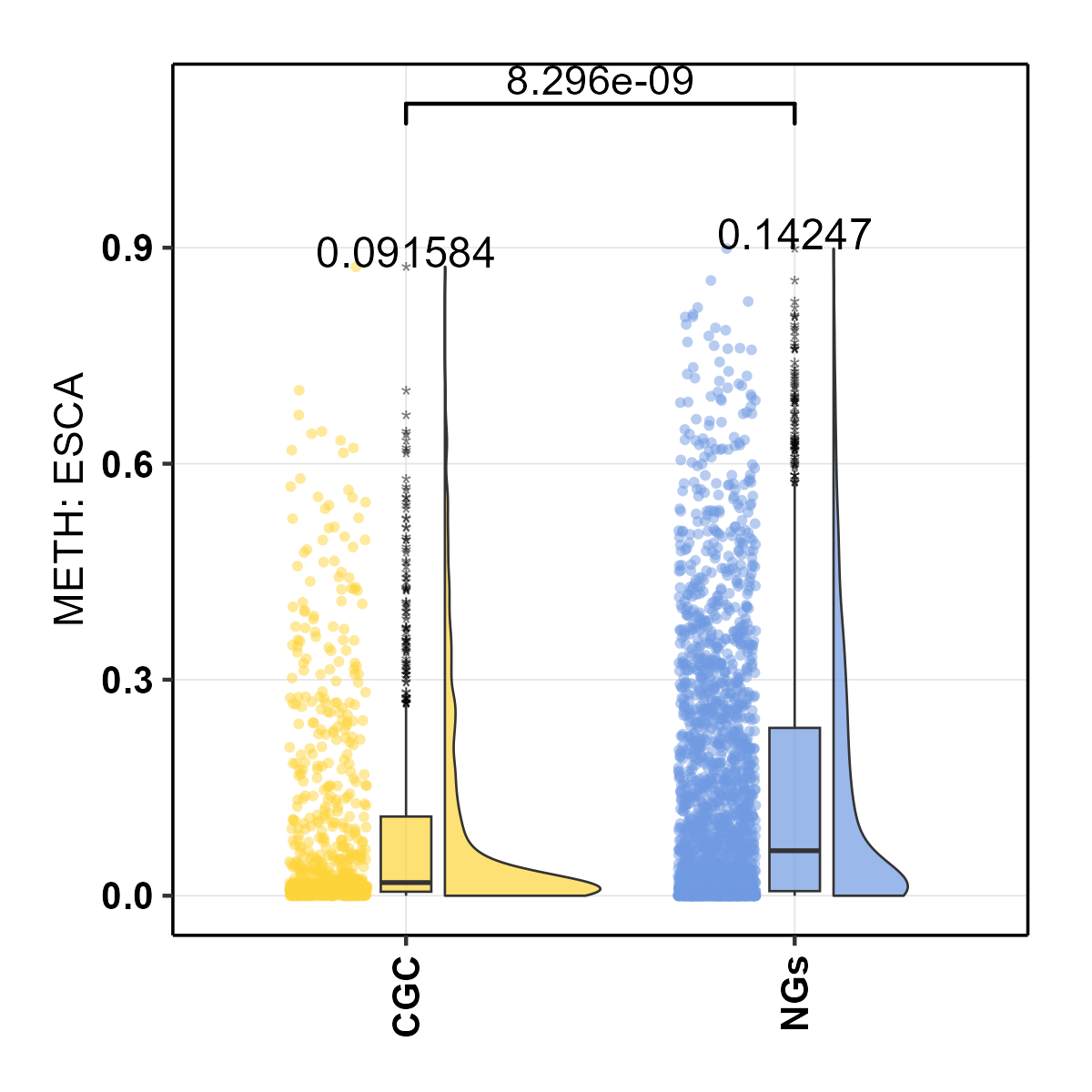

Supplement: Supplementary file 3 [file DataSheet1.ZIP › Supplementary file 5-1/IReflndex/METH_ESCA.png]

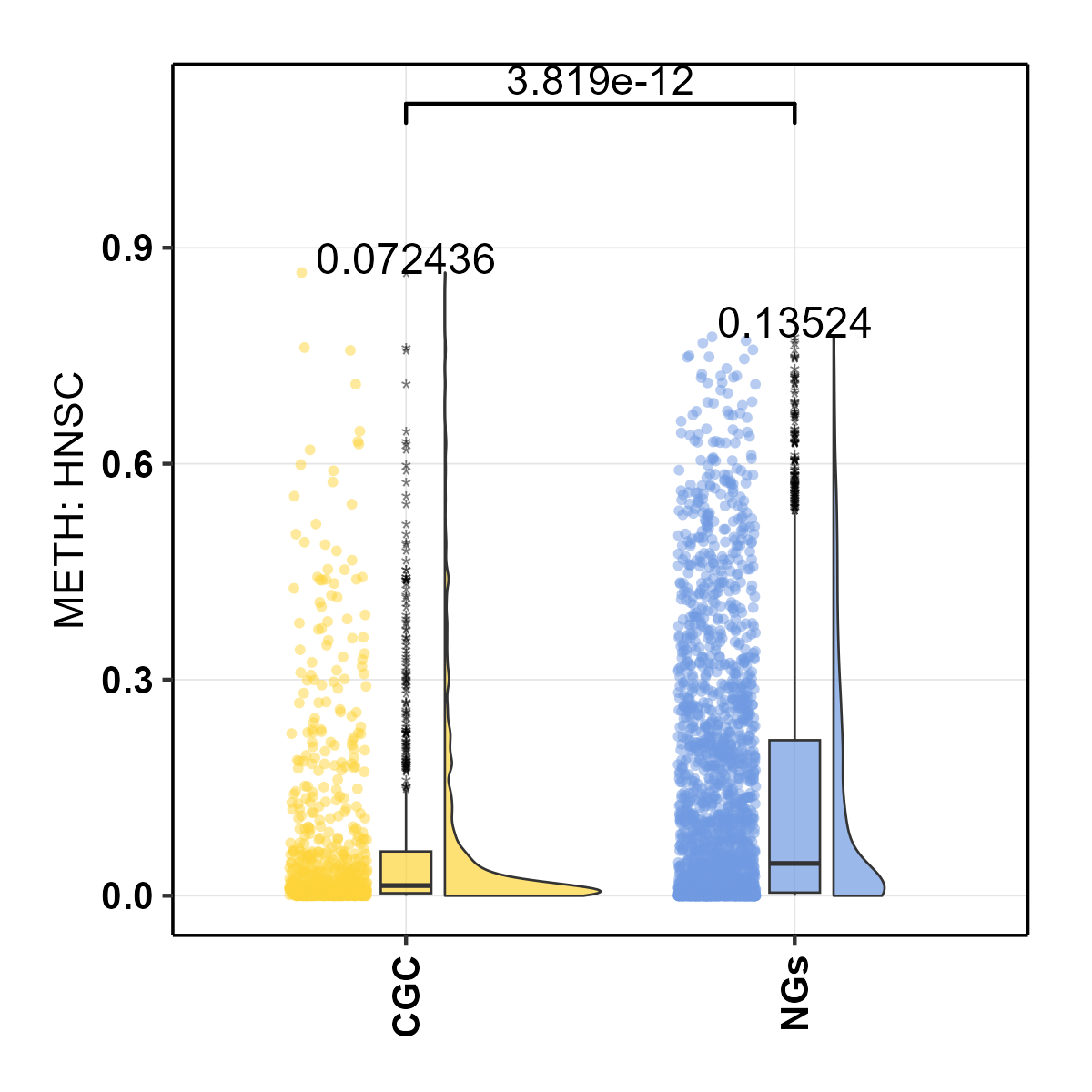

Supplement: Supplementary file 3 [file DataSheet1.ZIP › Supplementary file 5-1/IReflndex/METH_HNSC.png]

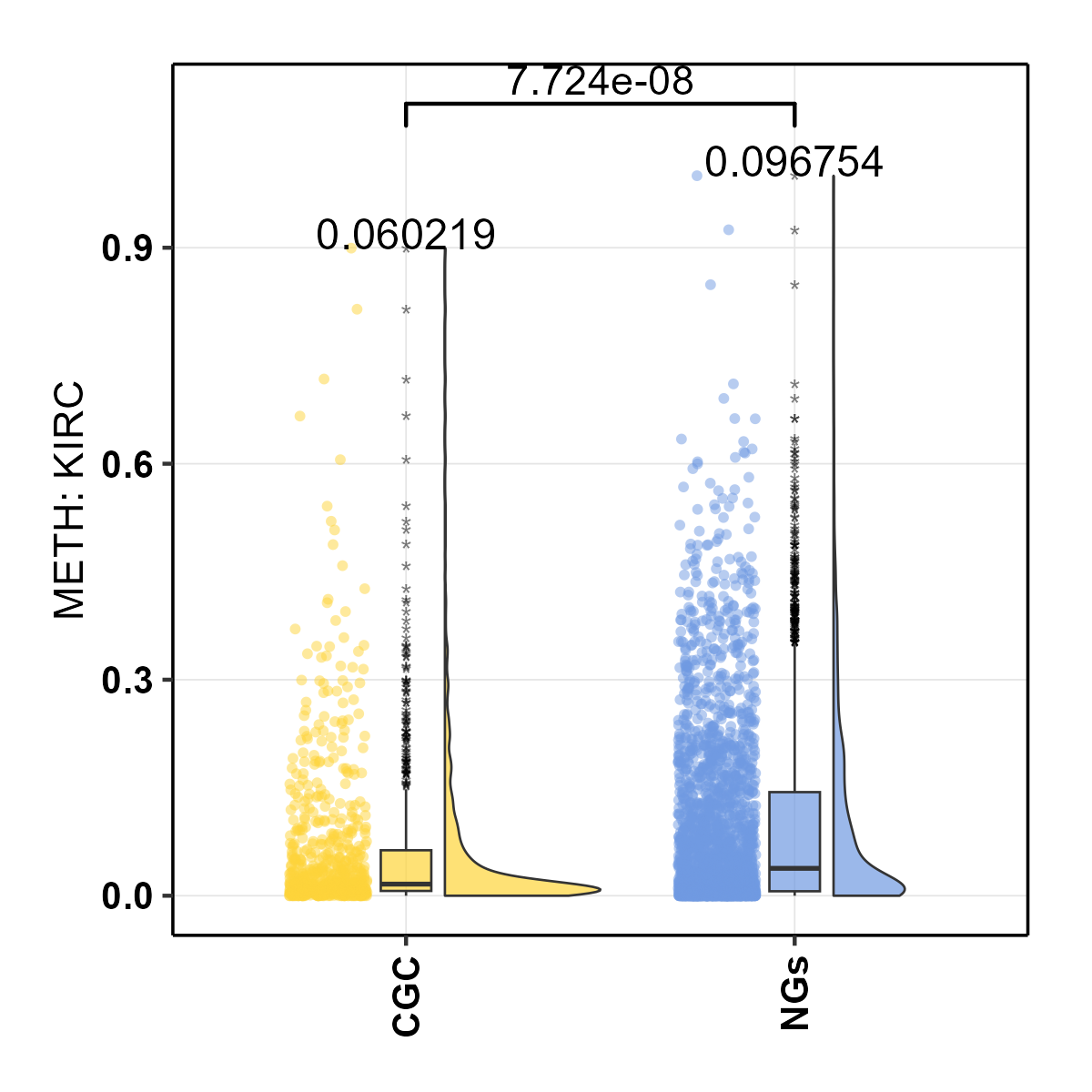

Supplement: Supplementary file 3 [file DataSheet1.ZIP › Supplementary file 5-1/IReflndex/METH_KIRC.png]

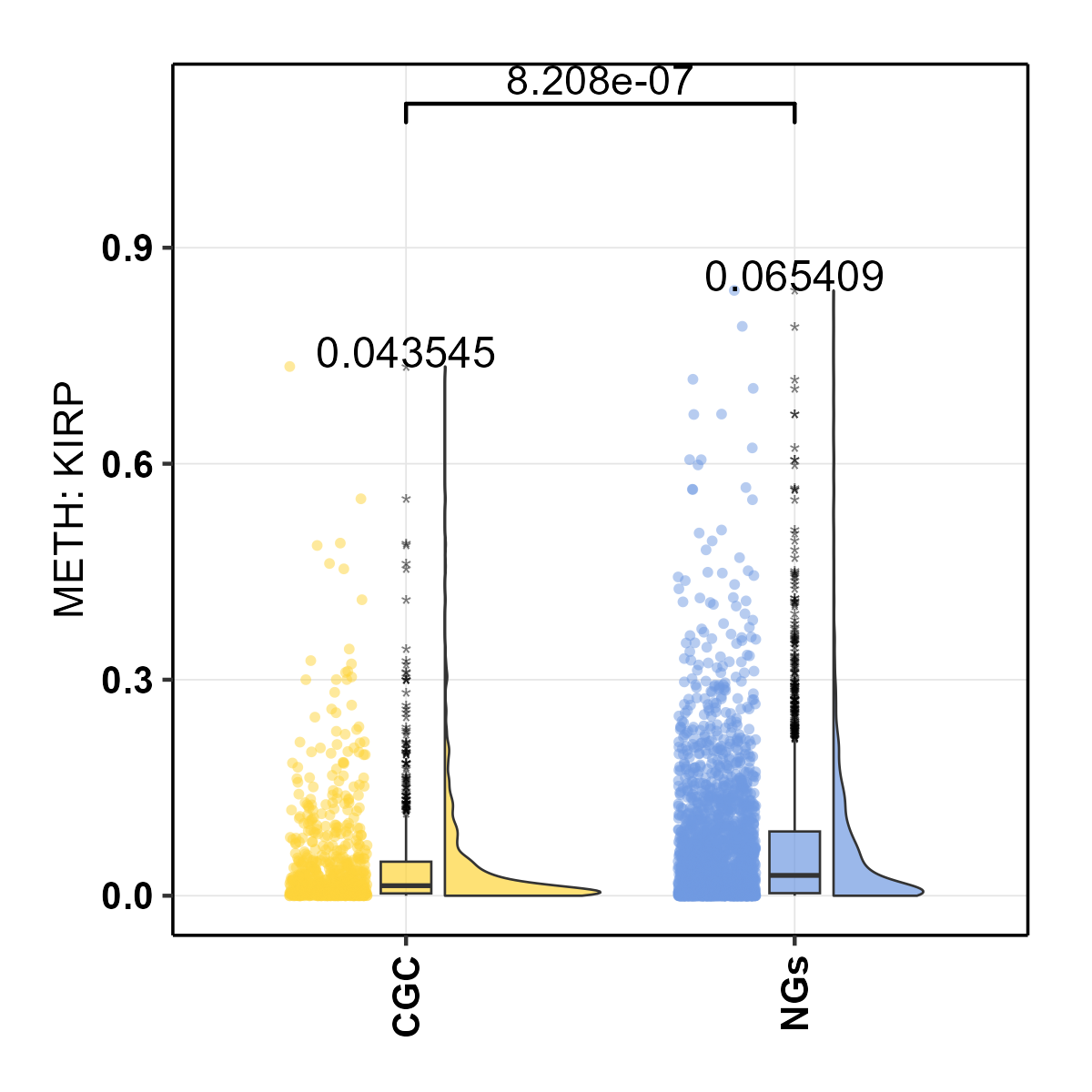

Supplement: Supplementary file 3 [file DataSheet1.ZIP › Supplementary file 5-1/IReflndex/METH_KIRP.png]

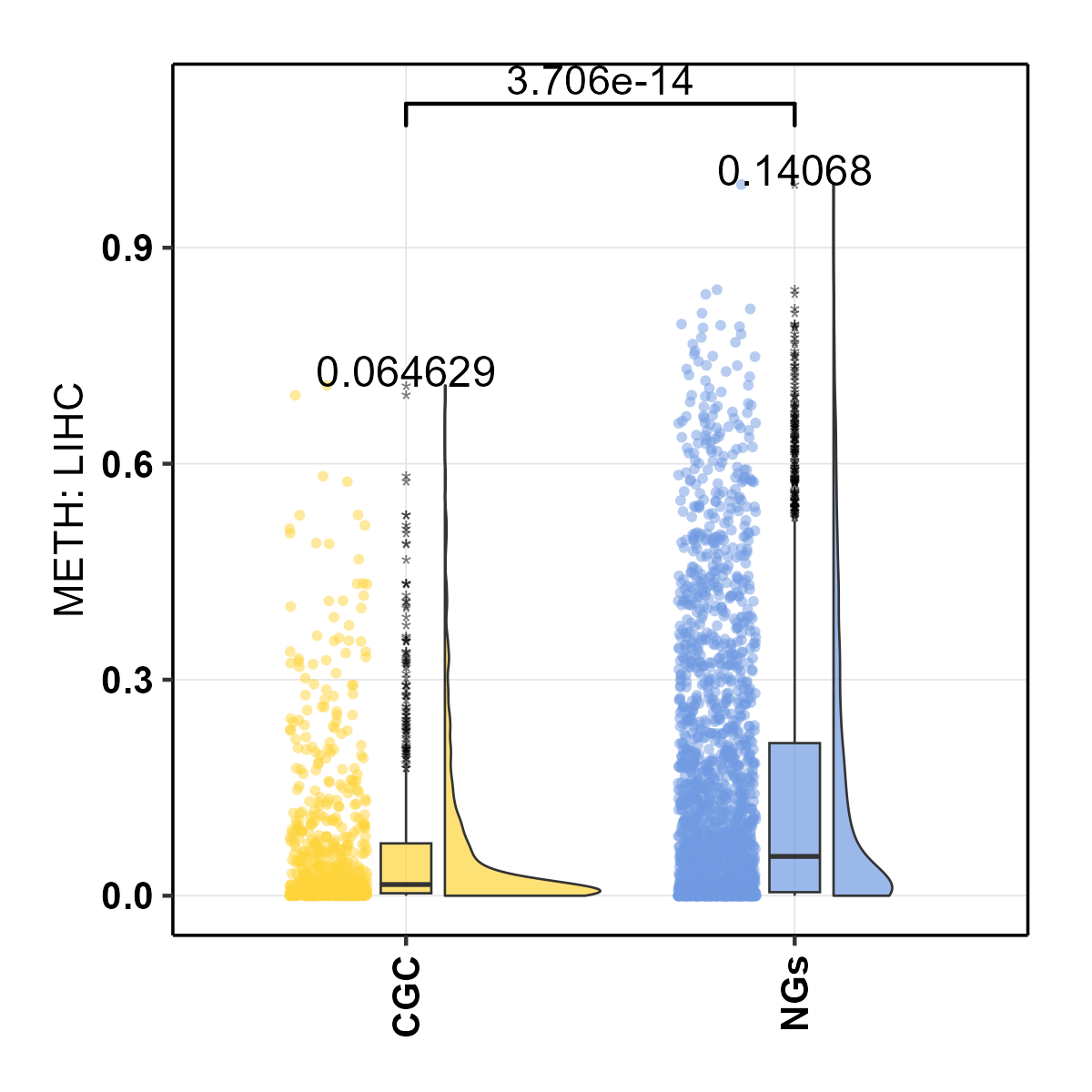

Supplement: Supplementary file 3 [file DataSheet1.ZIP › Supplementary file 5-1/IReflndex/METH_LIHC.png]

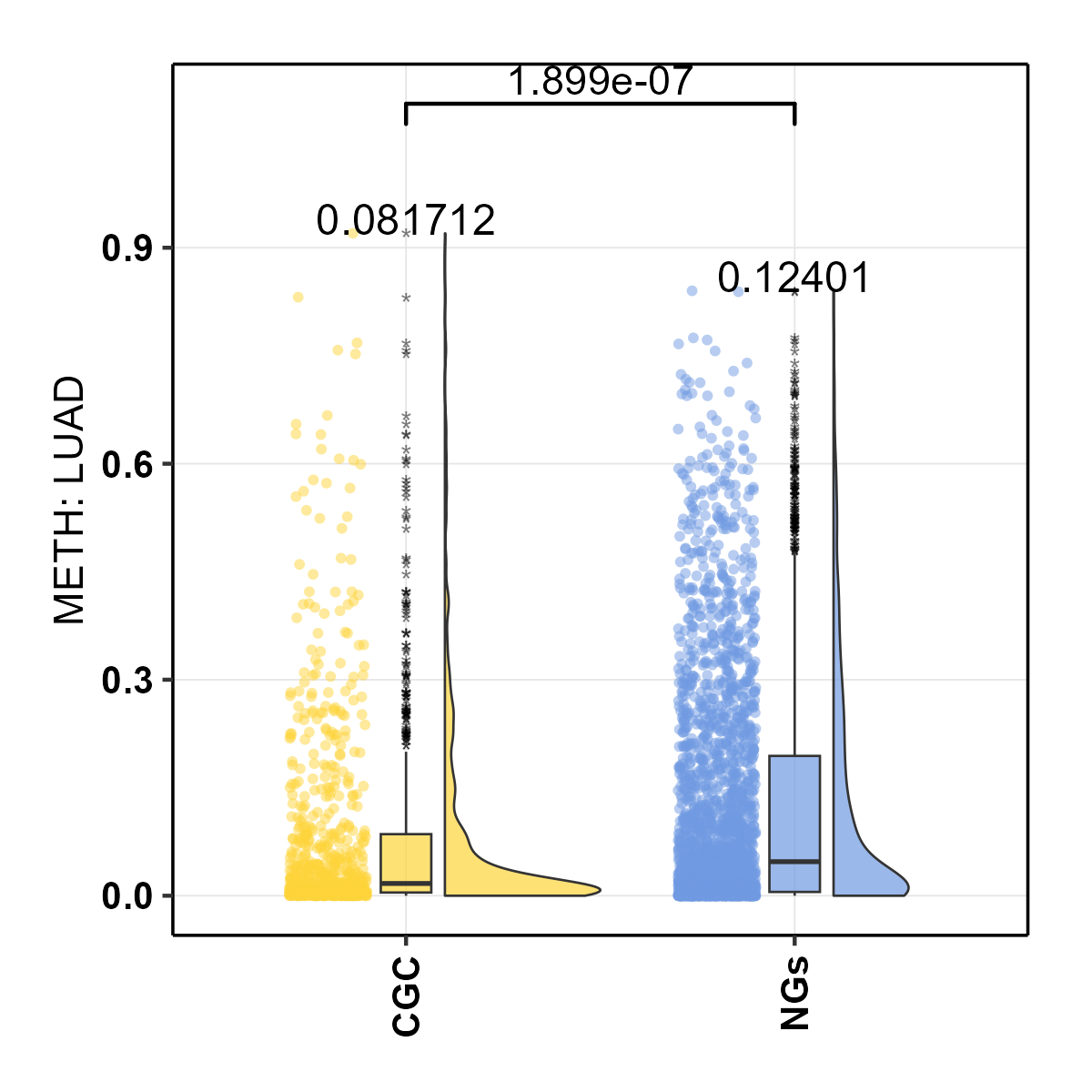

Supplement: Supplementary file 3 [file DataSheet1.ZIP › Supplementary file 5-1/IReflndex/METH_LUAD.png]

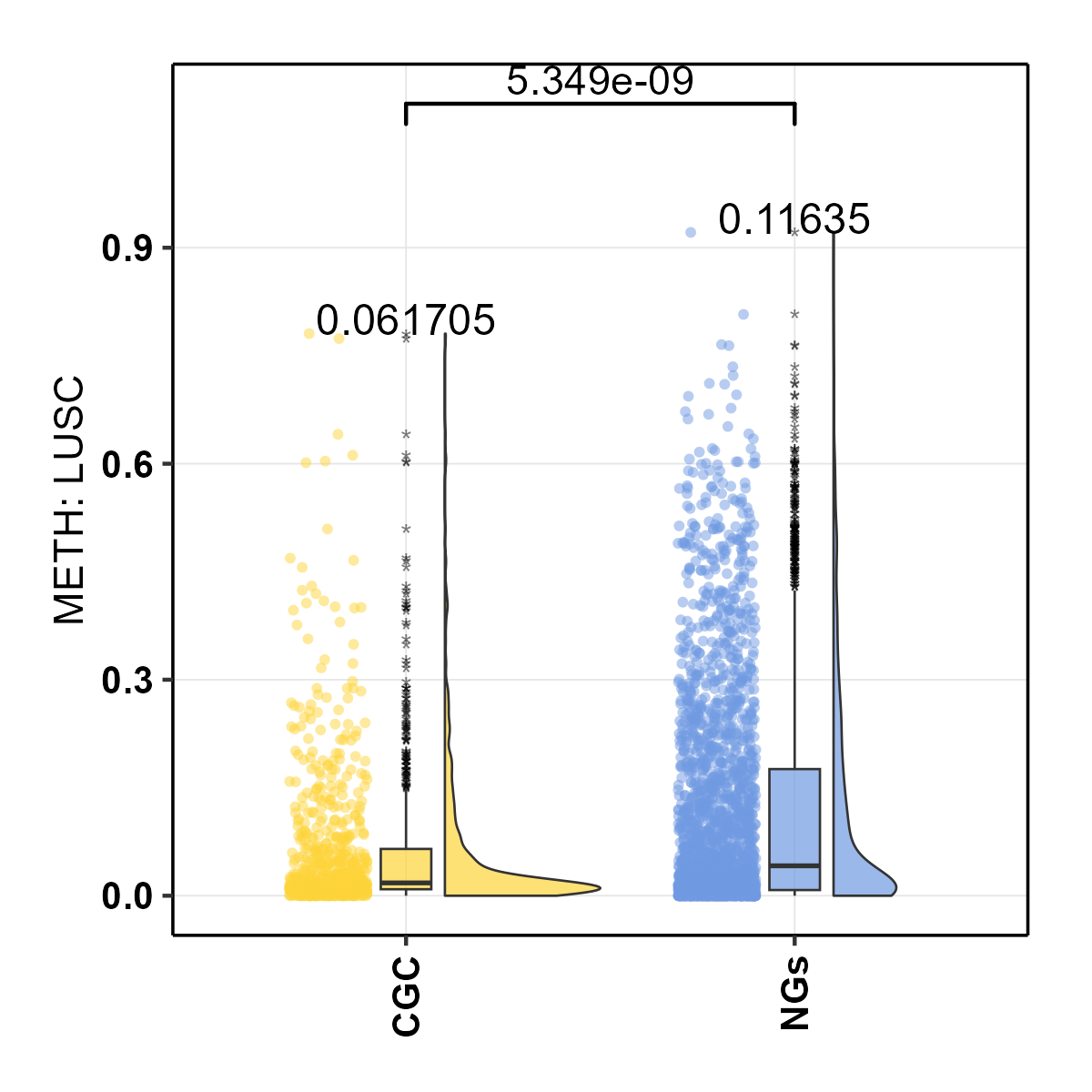

Supplement: Supplementary file 3 [file DataSheet1.ZIP › Supplementary file 5-1/IReflndex/METH_LUSC.png]

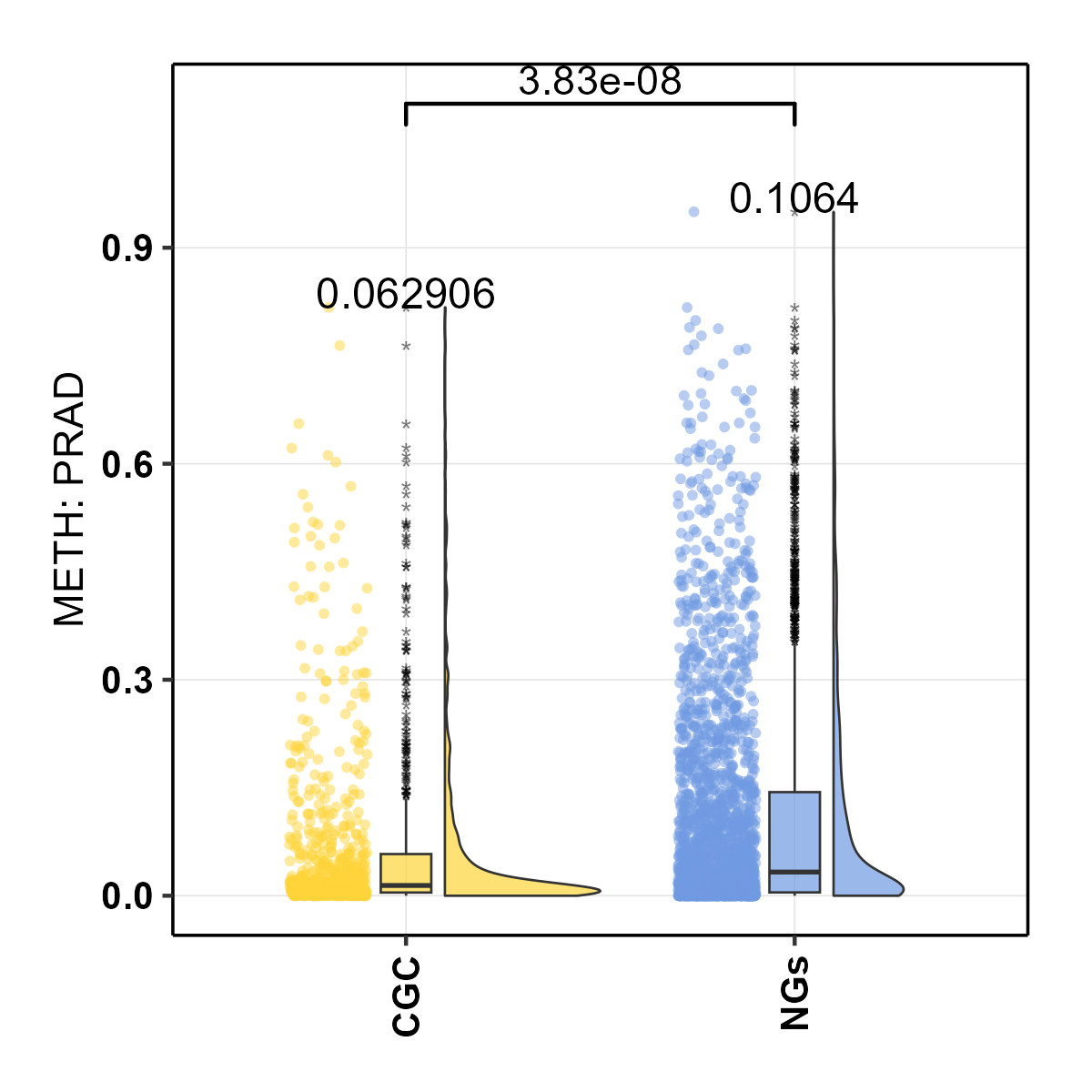

Supplement: Supplementary file 3 [file DataSheet1.ZIP › Supplementary file 5-1/IReflndex/METH_PRAD.png]

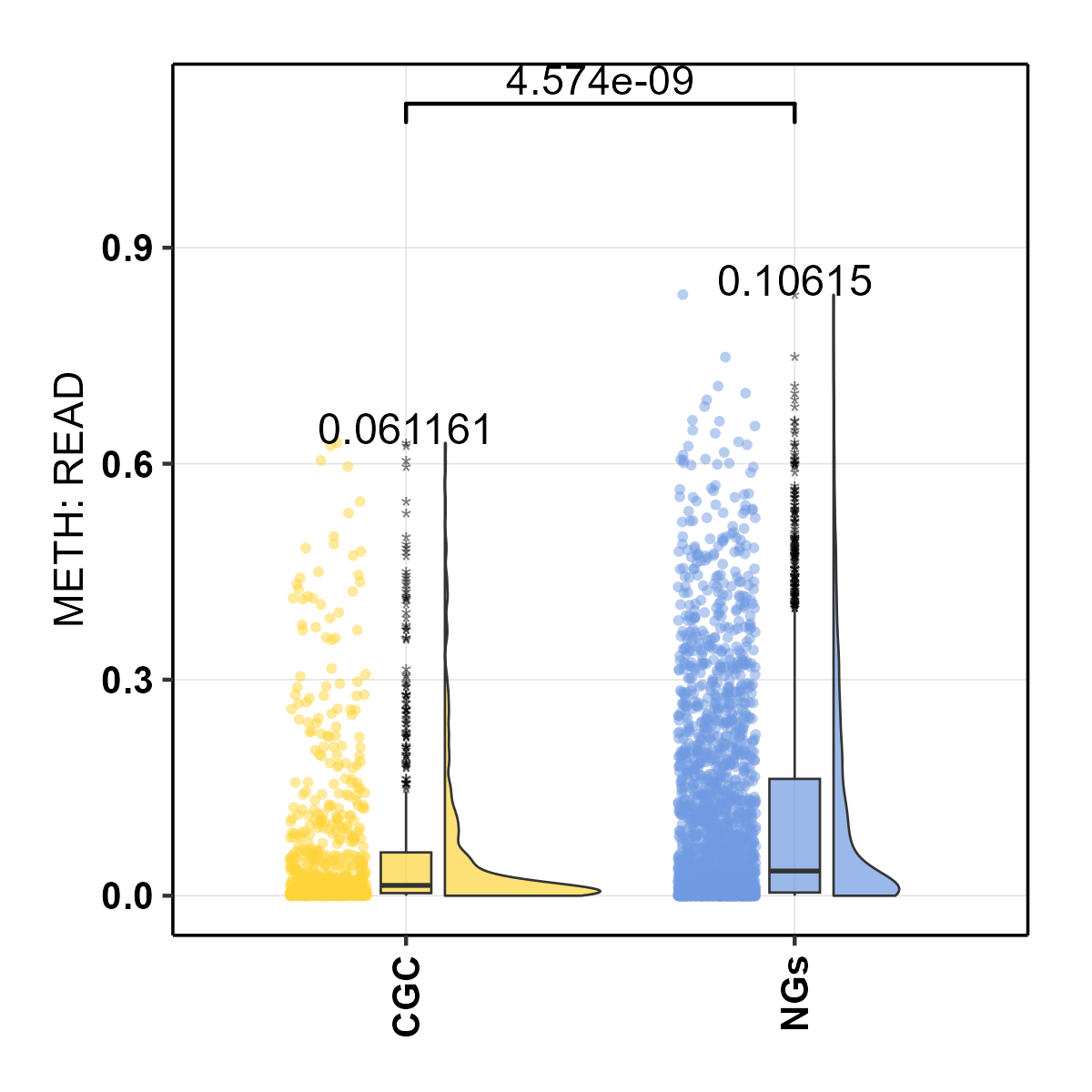

Supplement: Supplementary file 3 [file DataSheet1.ZIP › Supplementary file 5-1/IReflndex/METH_READ.png]

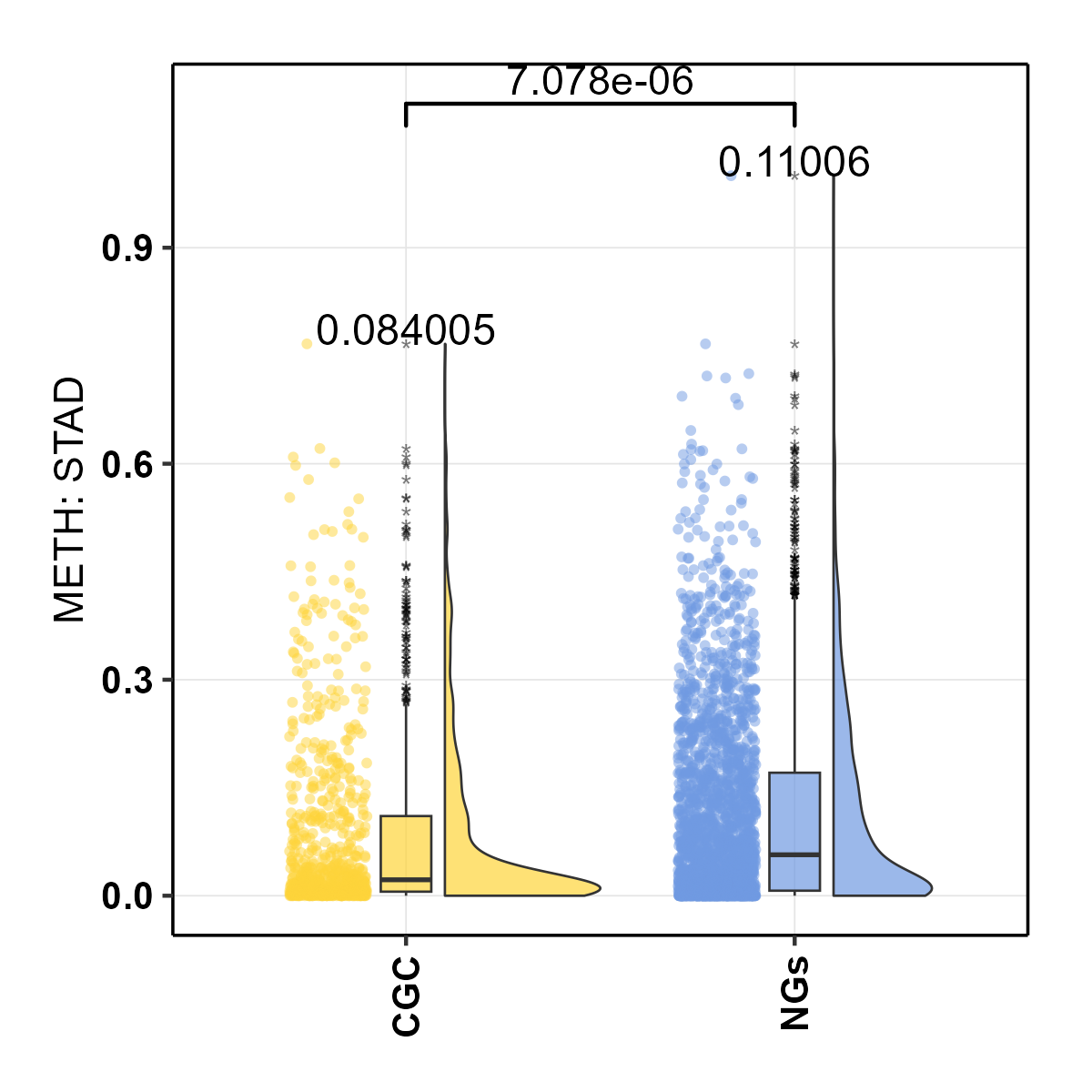

Supplement: Supplementary file 3 [file DataSheet1.ZIP › Supplementary file 5-1/IReflndex/METH_STAD.png]

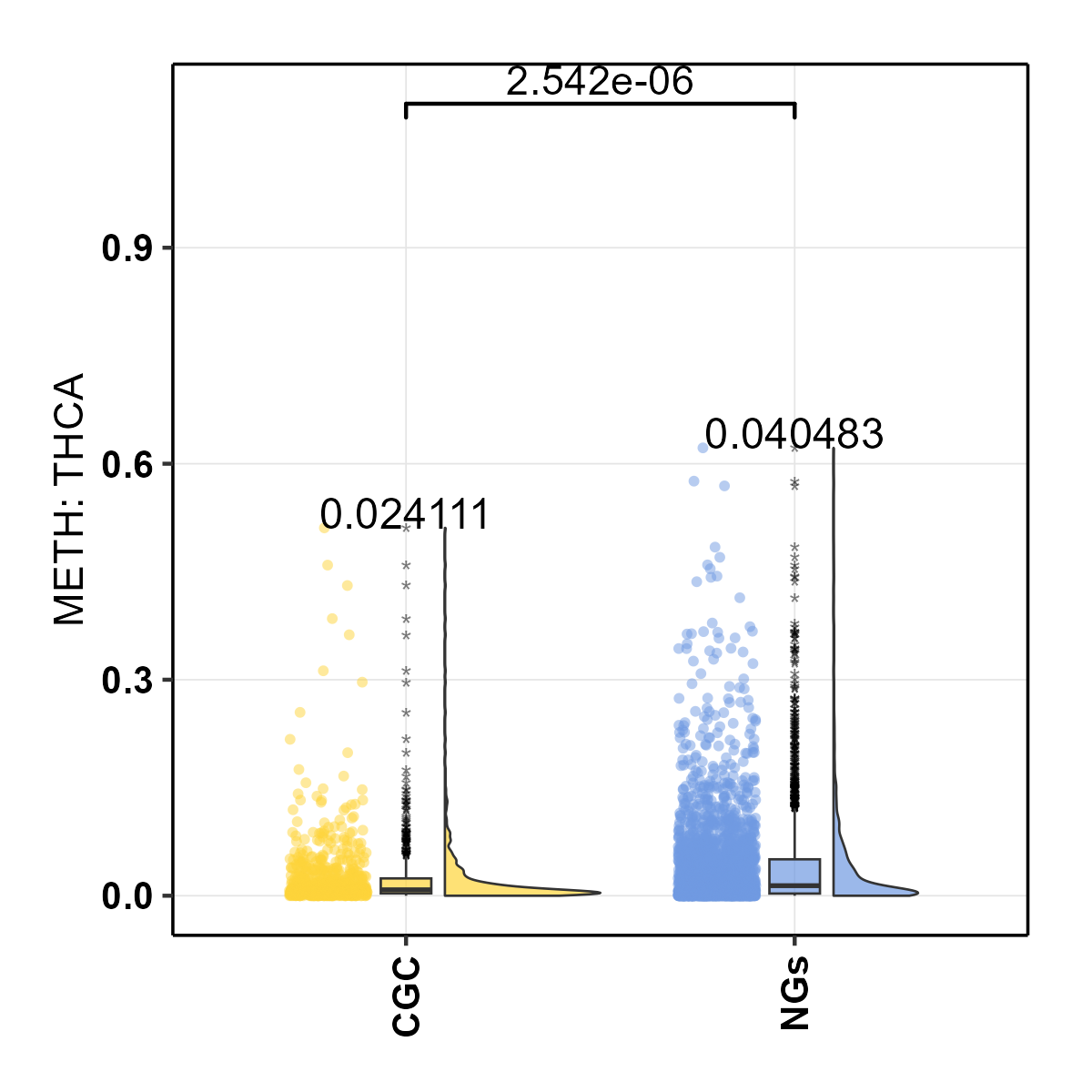

Supplement: Supplementary file 3 [file DataSheet1.ZIP › Supplementary file 5-1/IReflndex/METH_THCA.png]

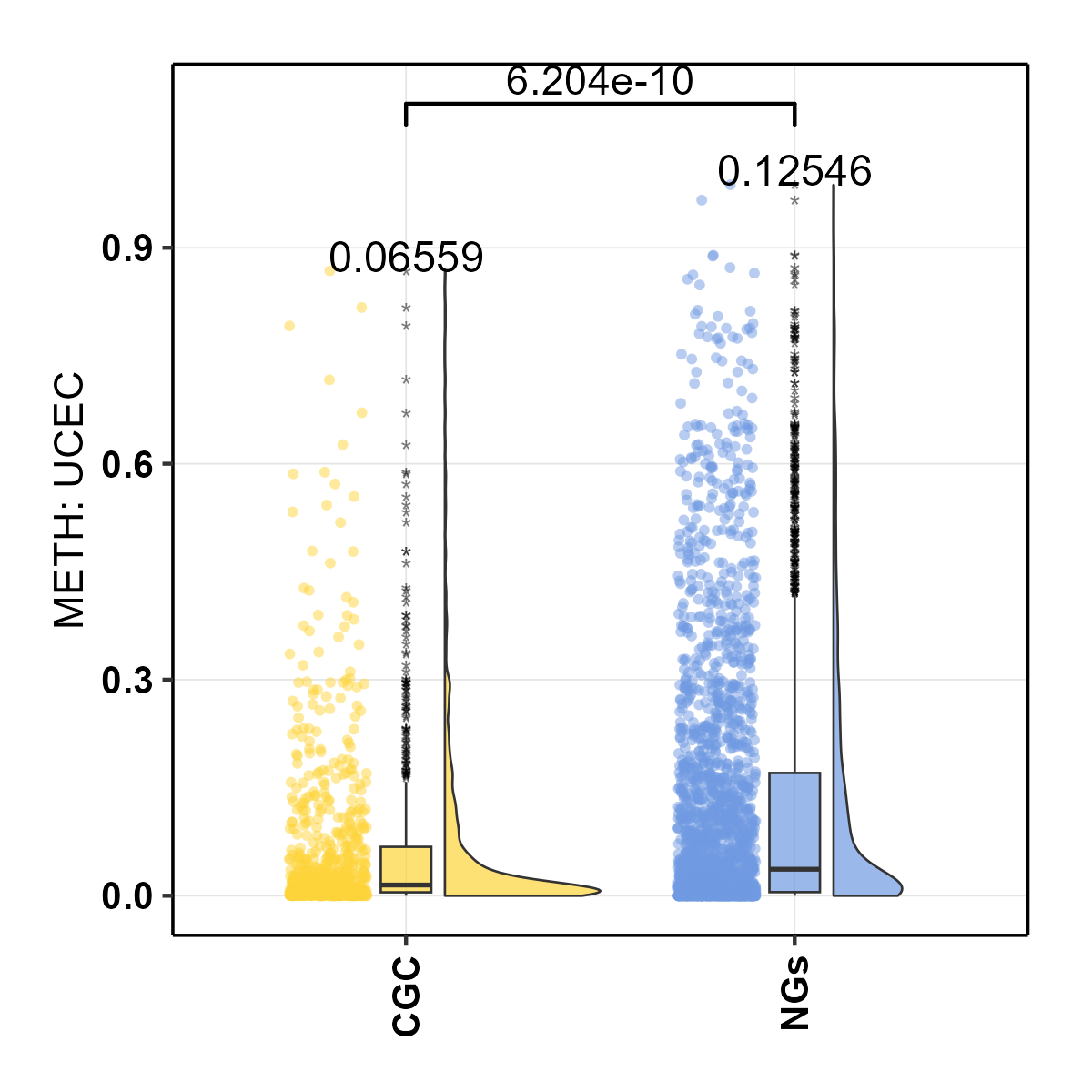

Supplement: Supplementary file 3 [file DataSheet1.ZIP › Supplementary file 5-1/IReflndex/METH_UCEC.png]

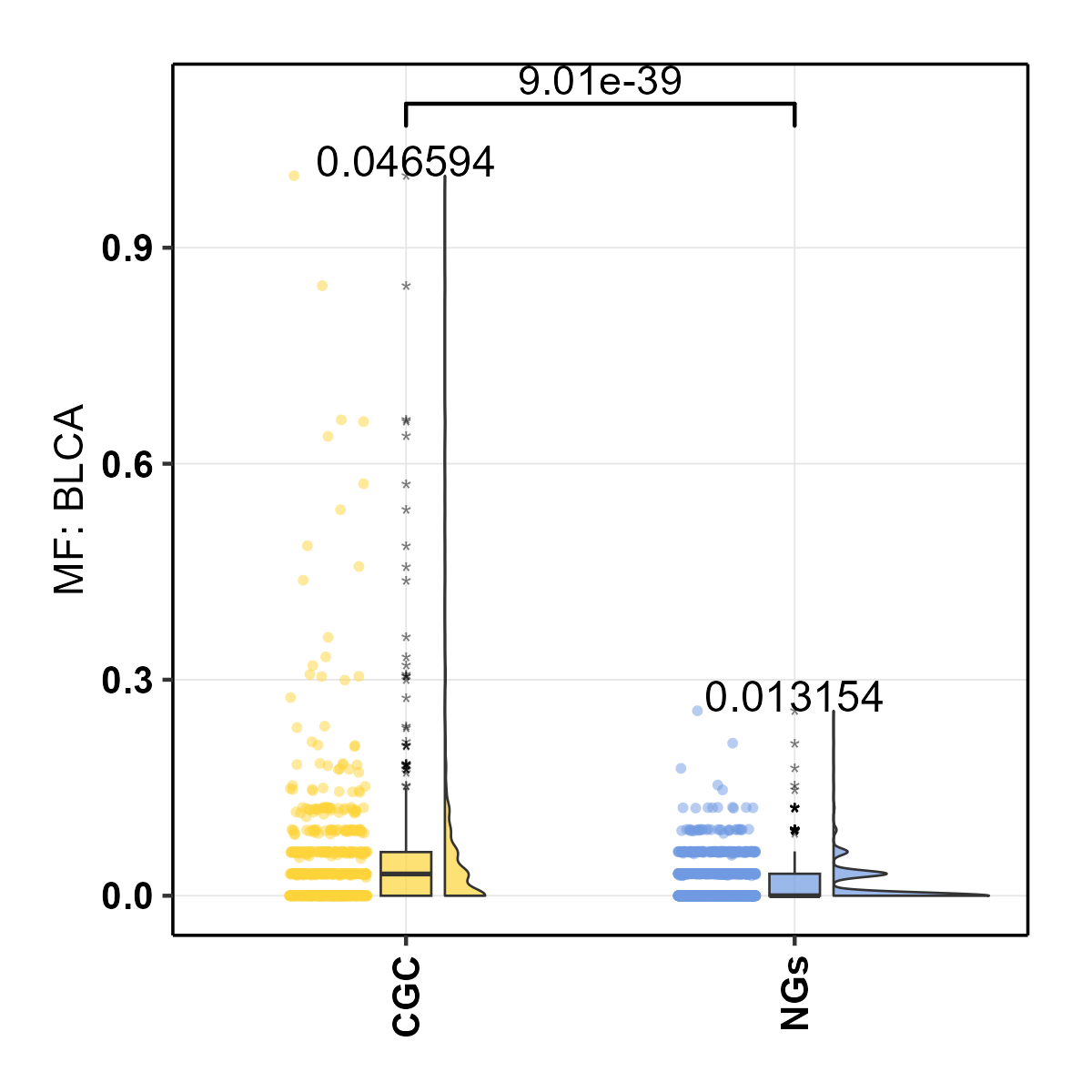

Supplement: Supplementary file 3 [file DataSheet1.ZIP › Supplementary file 5-1/IReflndex/MF_BLCA.png]

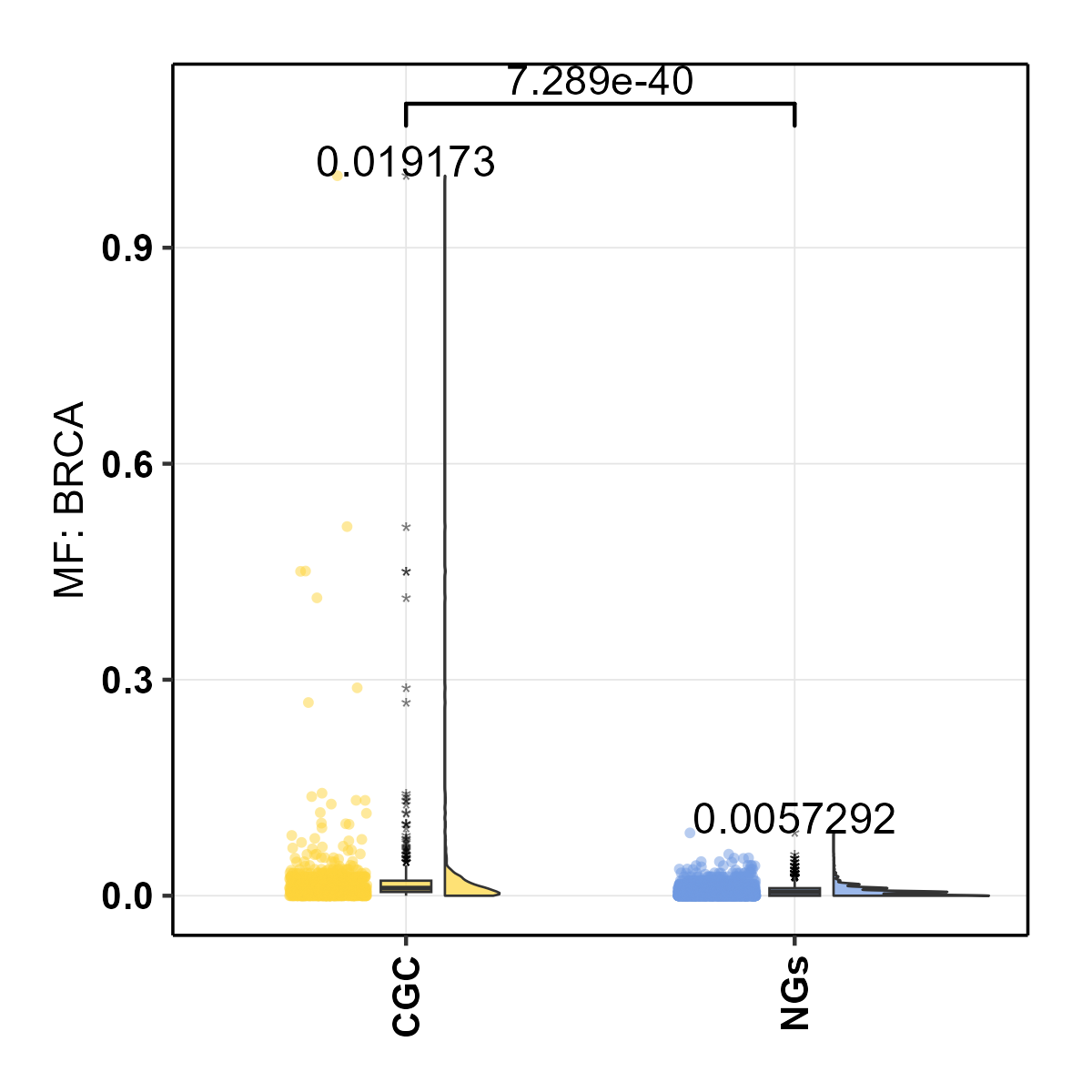

Supplement: Supplementary file 3 [file DataSheet1.ZIP › Supplementary file 5-1/IReflndex/MF_BRCA.png]

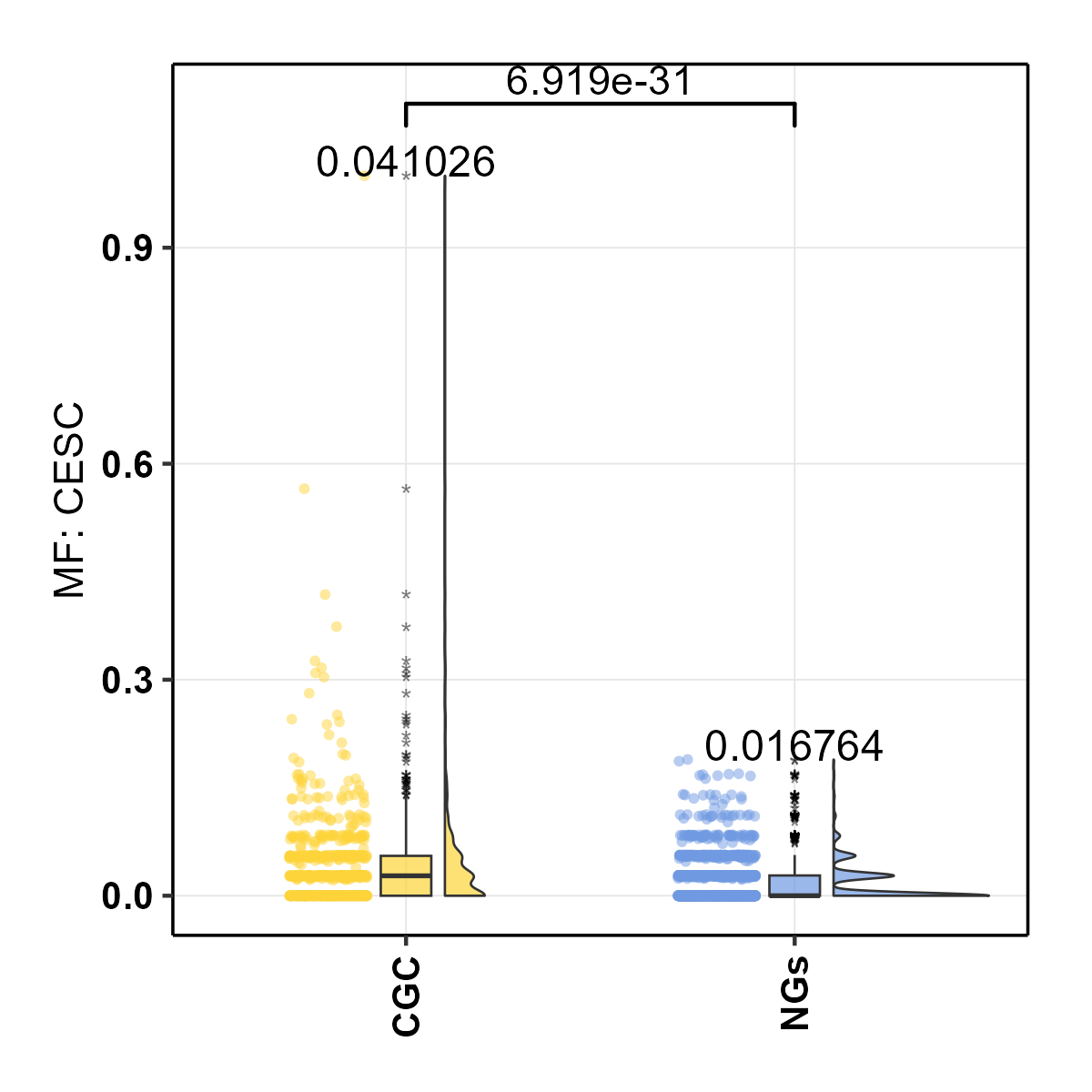

Supplement: Supplementary file 3 [file DataSheet1.ZIP › Supplementary file 5-1/IReflndex/MF_CESC.png]

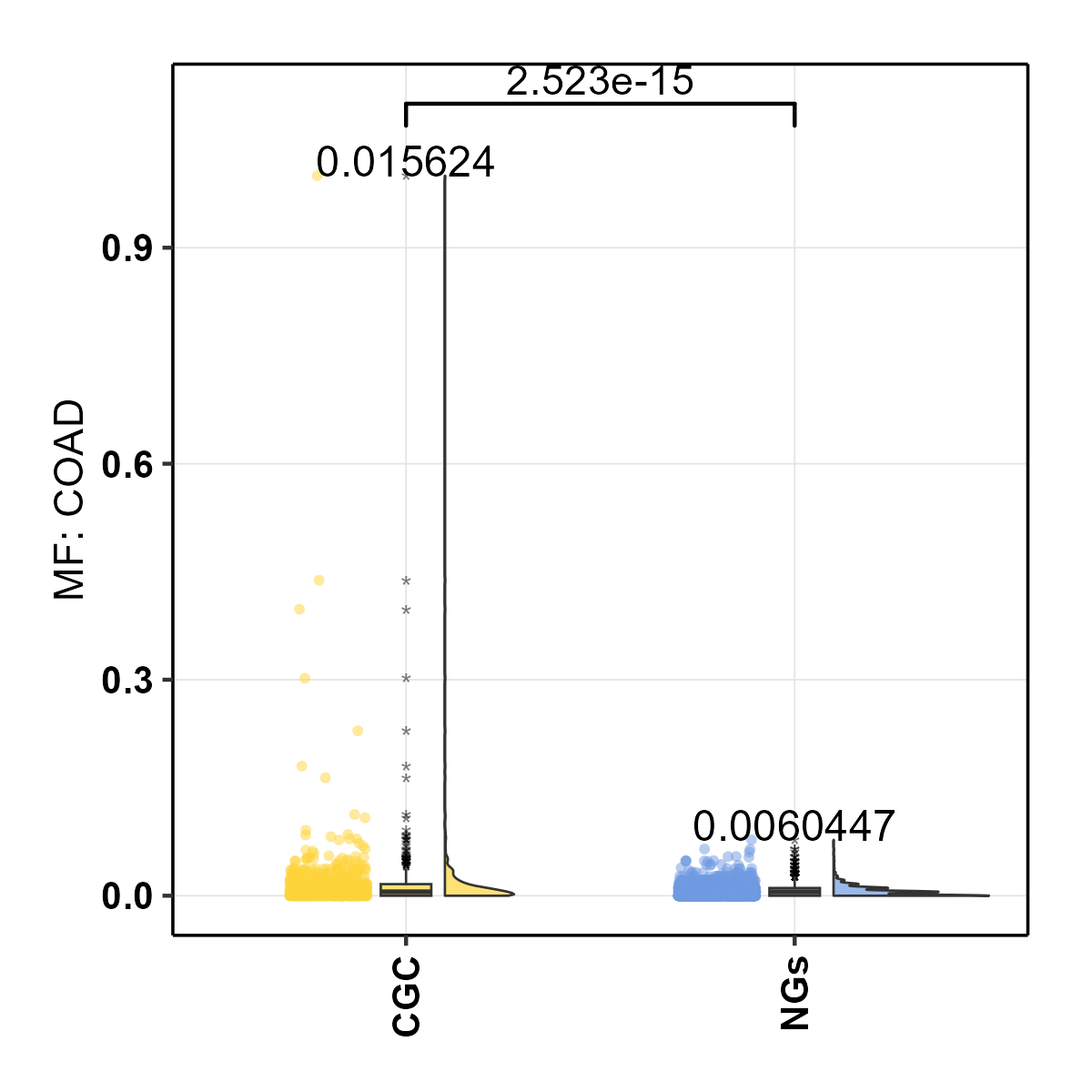

Supplement: Supplementary file 3 [file DataSheet1.ZIP › Supplementary file 5-1/IReflndex/MF_COAD.png]

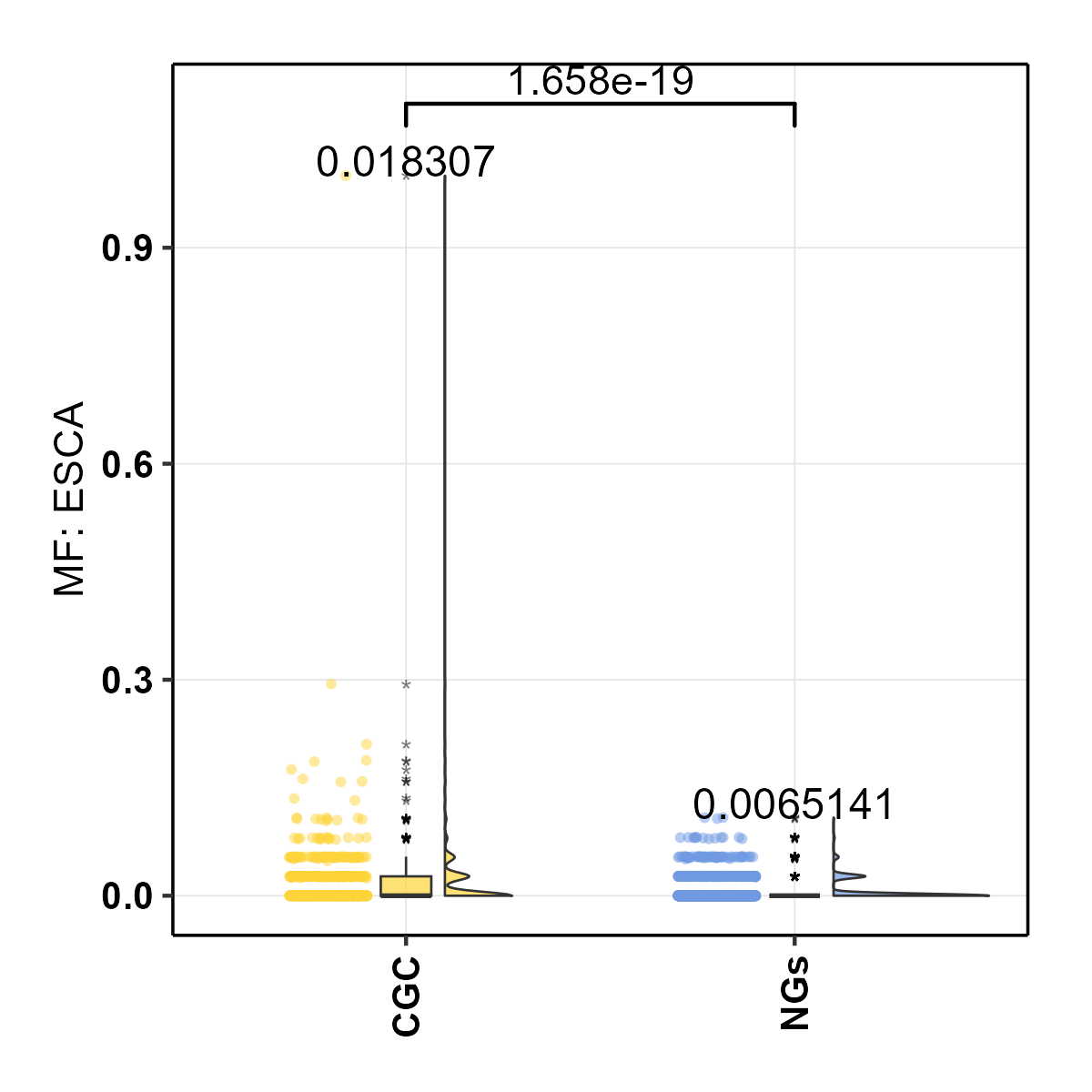

Supplement: Supplementary file 3 [file DataSheet1.ZIP › Supplementary file 5-1/IReflndex/MF_ESCA.png]

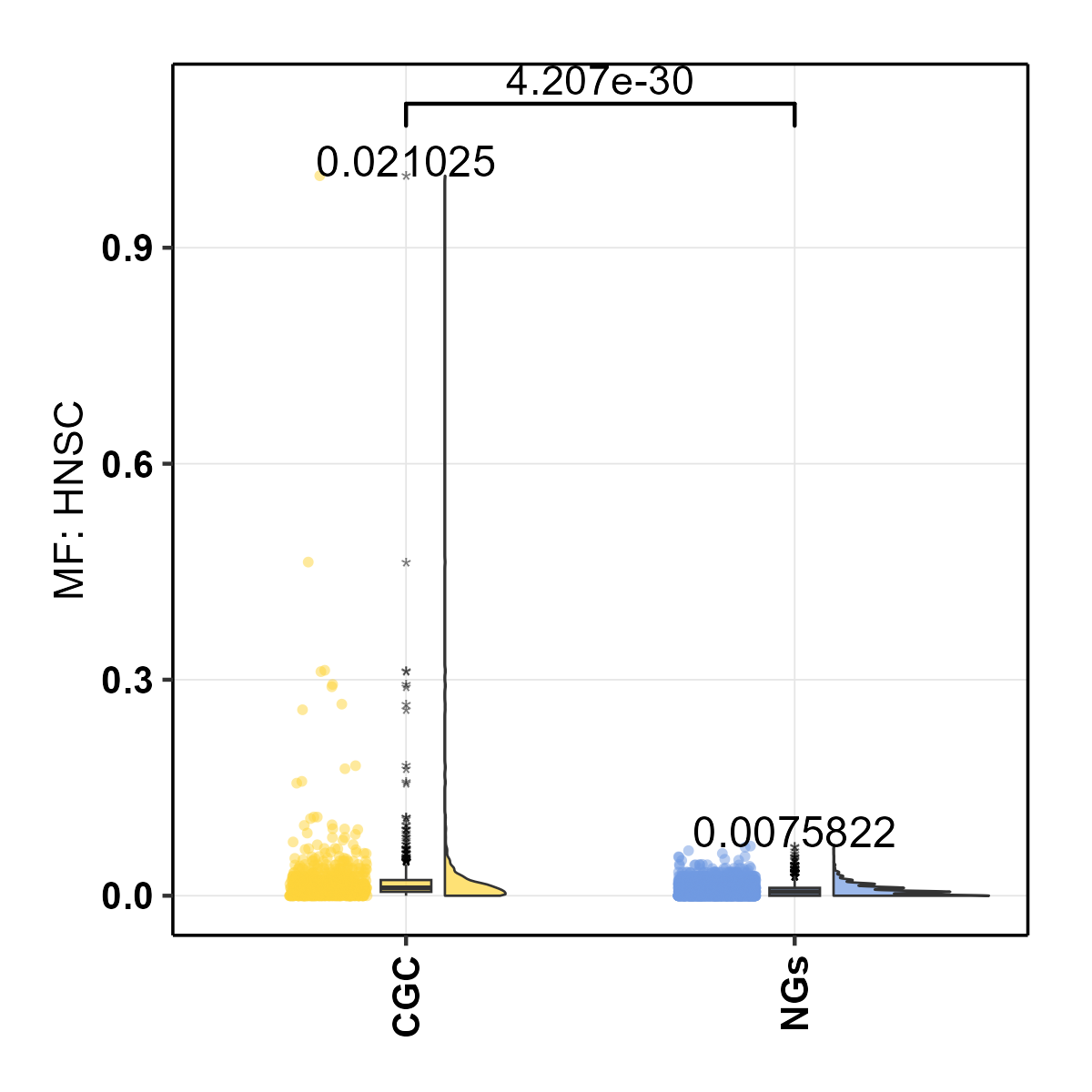

Supplement: Supplementary file 3 [file DataSheet1.ZIP › Supplementary file 5-1/IReflndex/MF_HNSC.png]

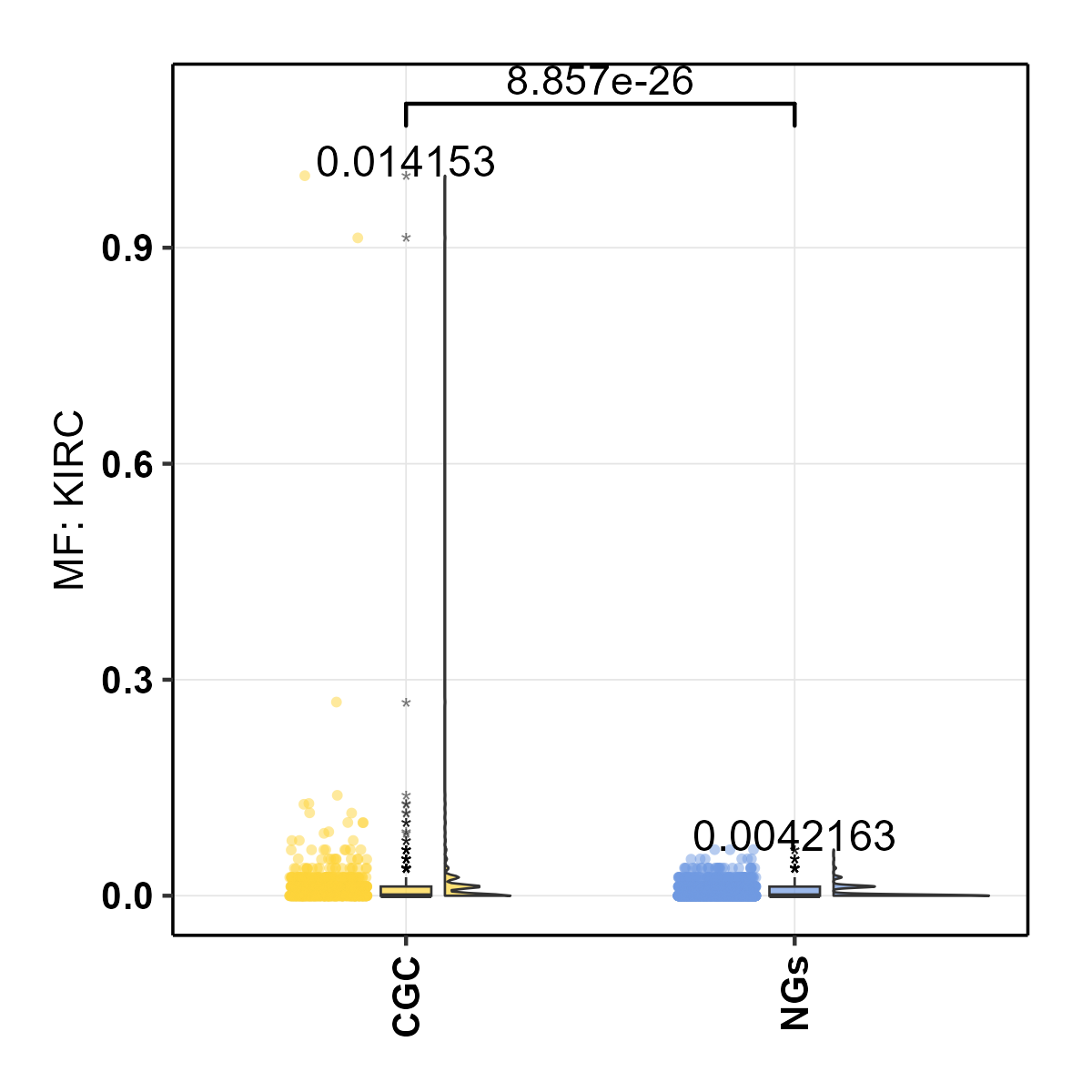

Supplement: Supplementary file 3 [file DataSheet1.ZIP › Supplementary file 5-1/IReflndex/MF_KIRC.png]

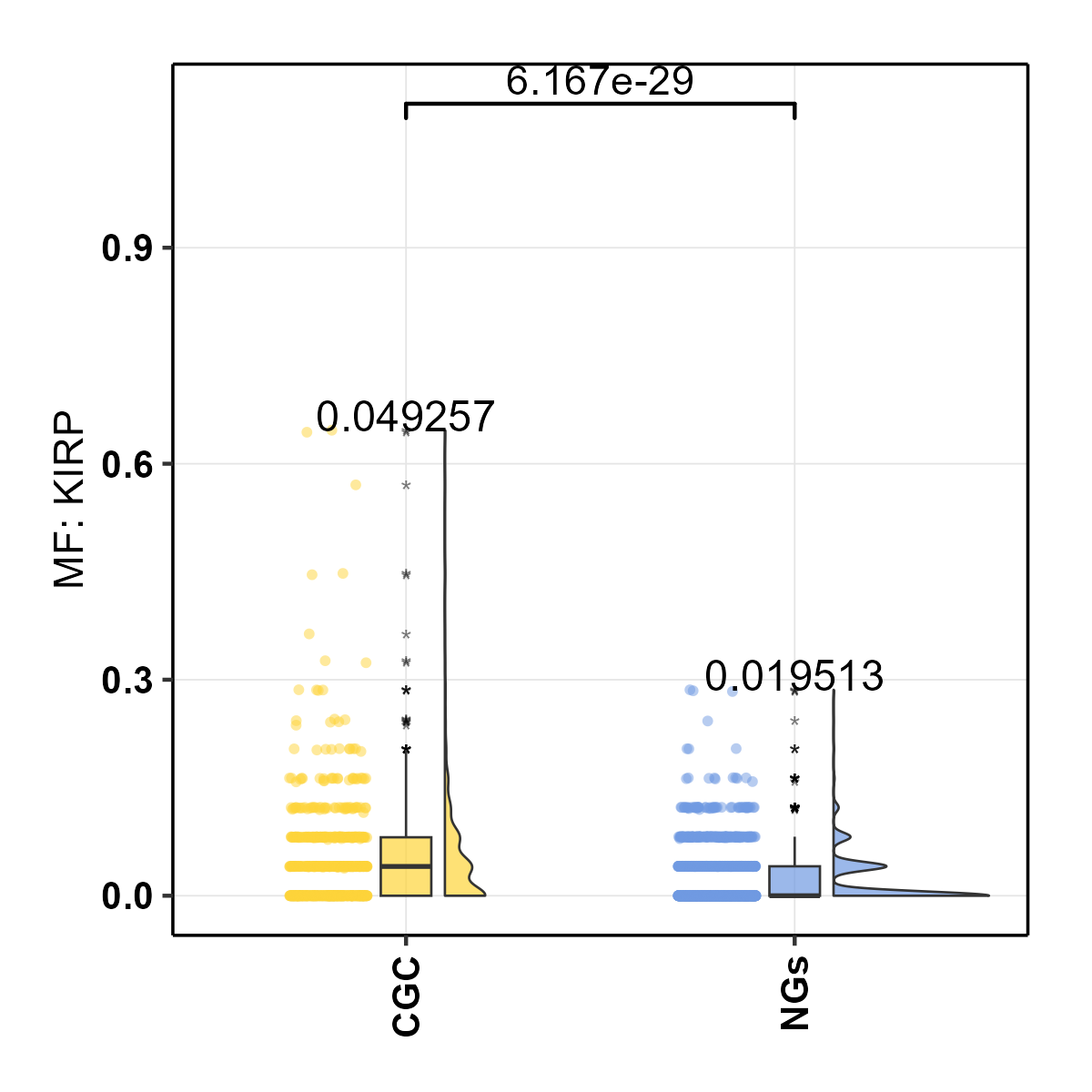

Supplement: Supplementary file 3 [file DataSheet1.ZIP › Supplementary file 5-1/IReflndex/MF_KIRP.png]

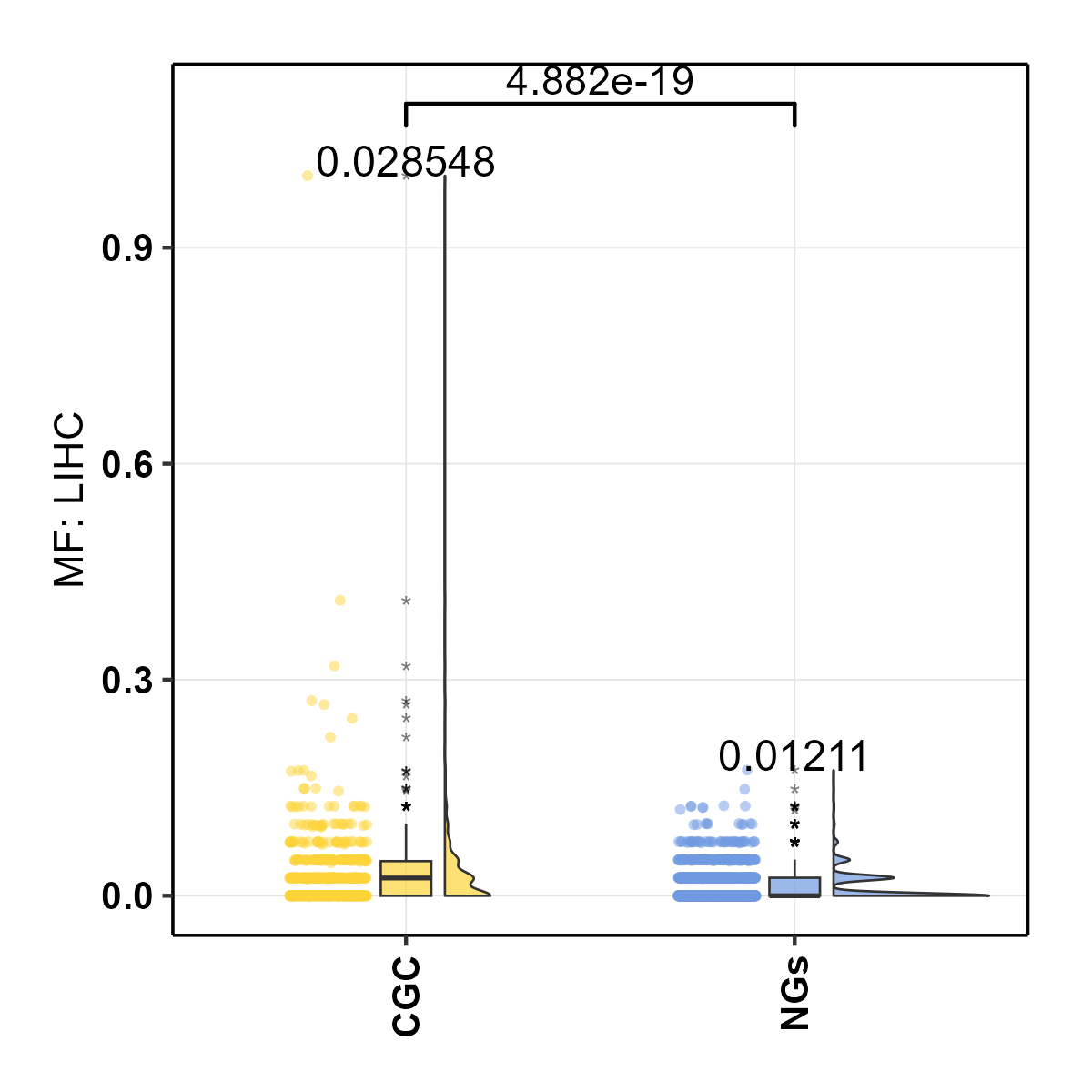

Supplement: Supplementary file 3 [file DataSheet1.ZIP › Supplementary file 5-1/IReflndex/MF_LIHC.png]

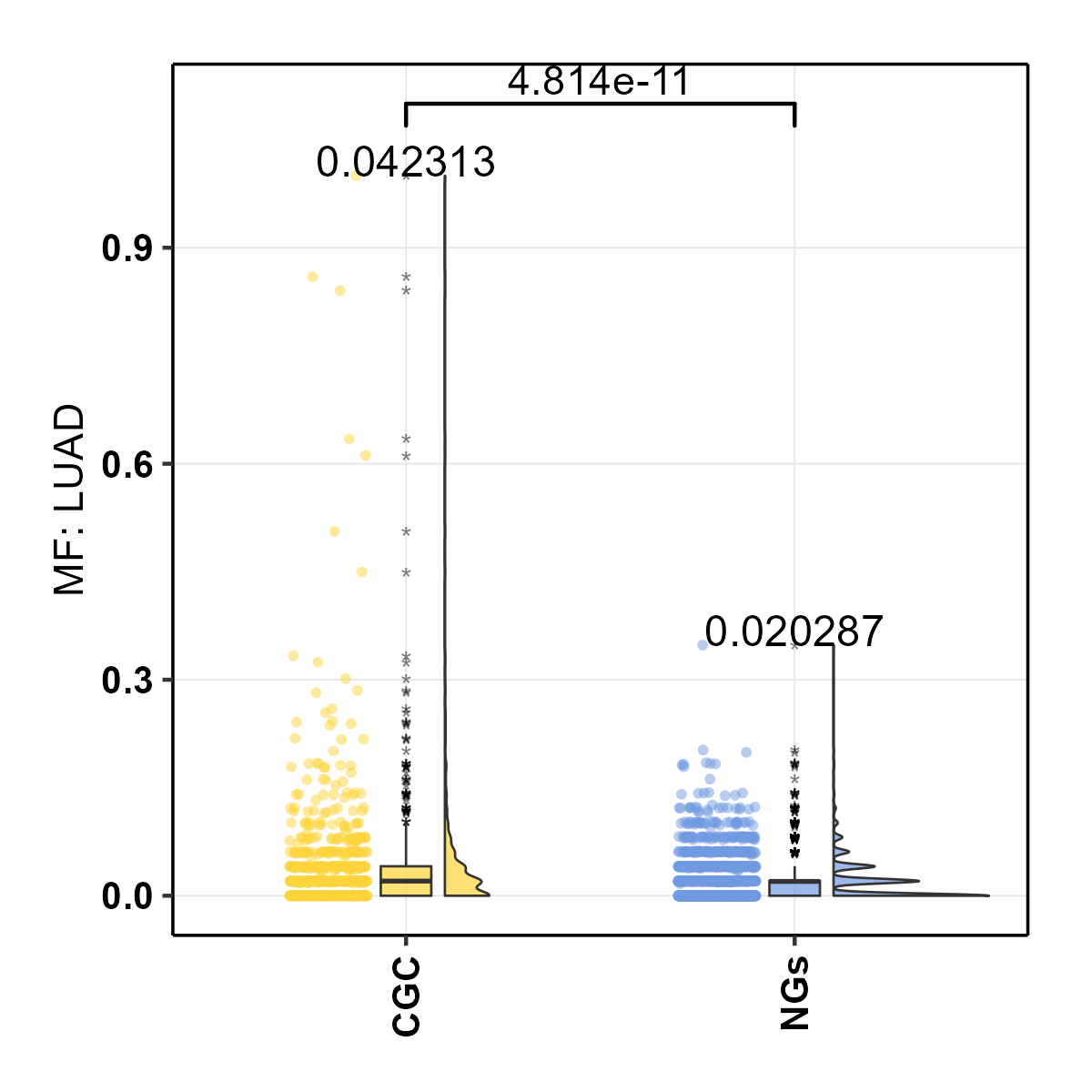

Supplement: Supplementary file 3 [file DataSheet1.ZIP › Supplementary file 5-1/IReflndex/MF_LUAD.png]

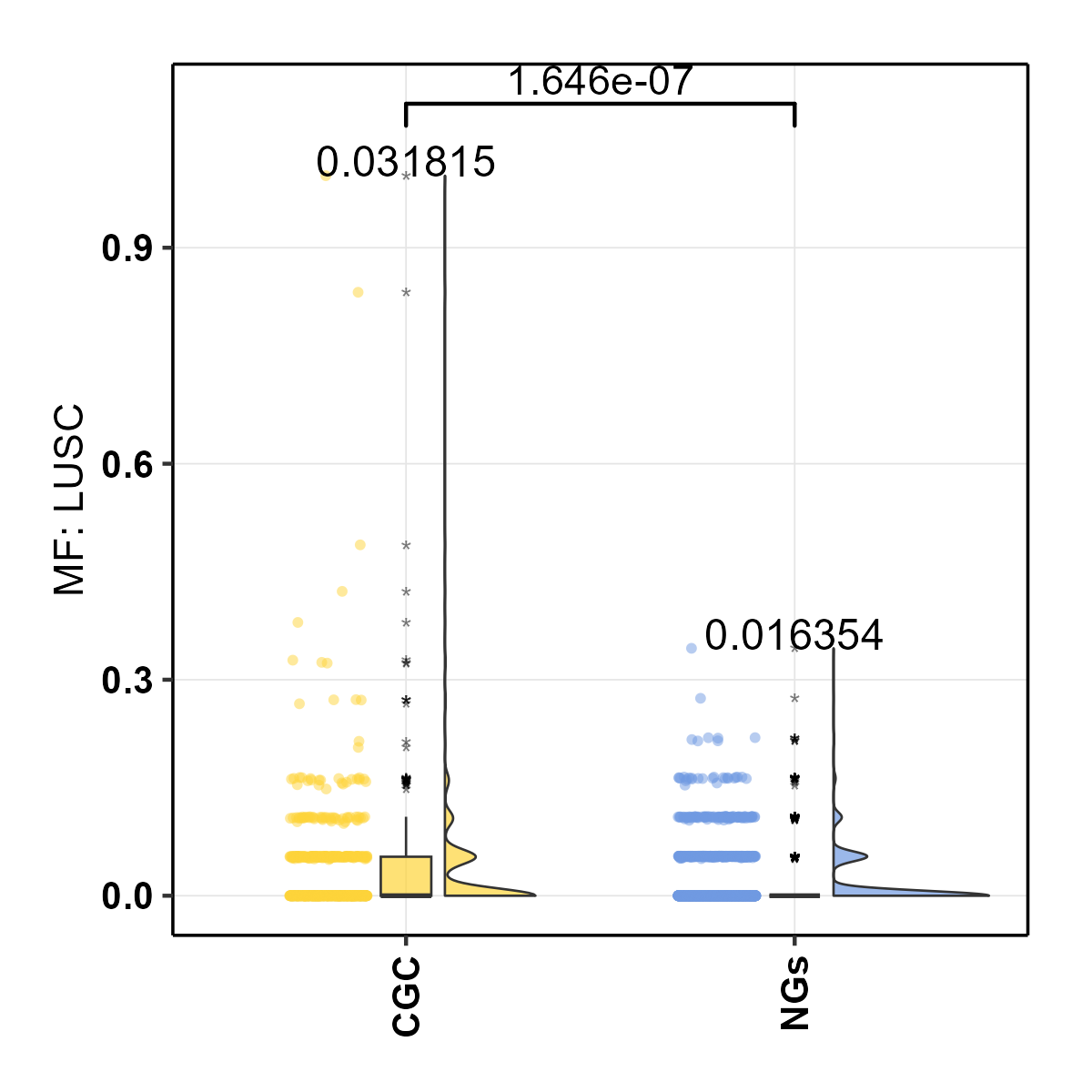

Supplement: Supplementary file 3 [file DataSheet1.ZIP › Supplementary file 5-1/IReflndex/MF_LUSC.png]

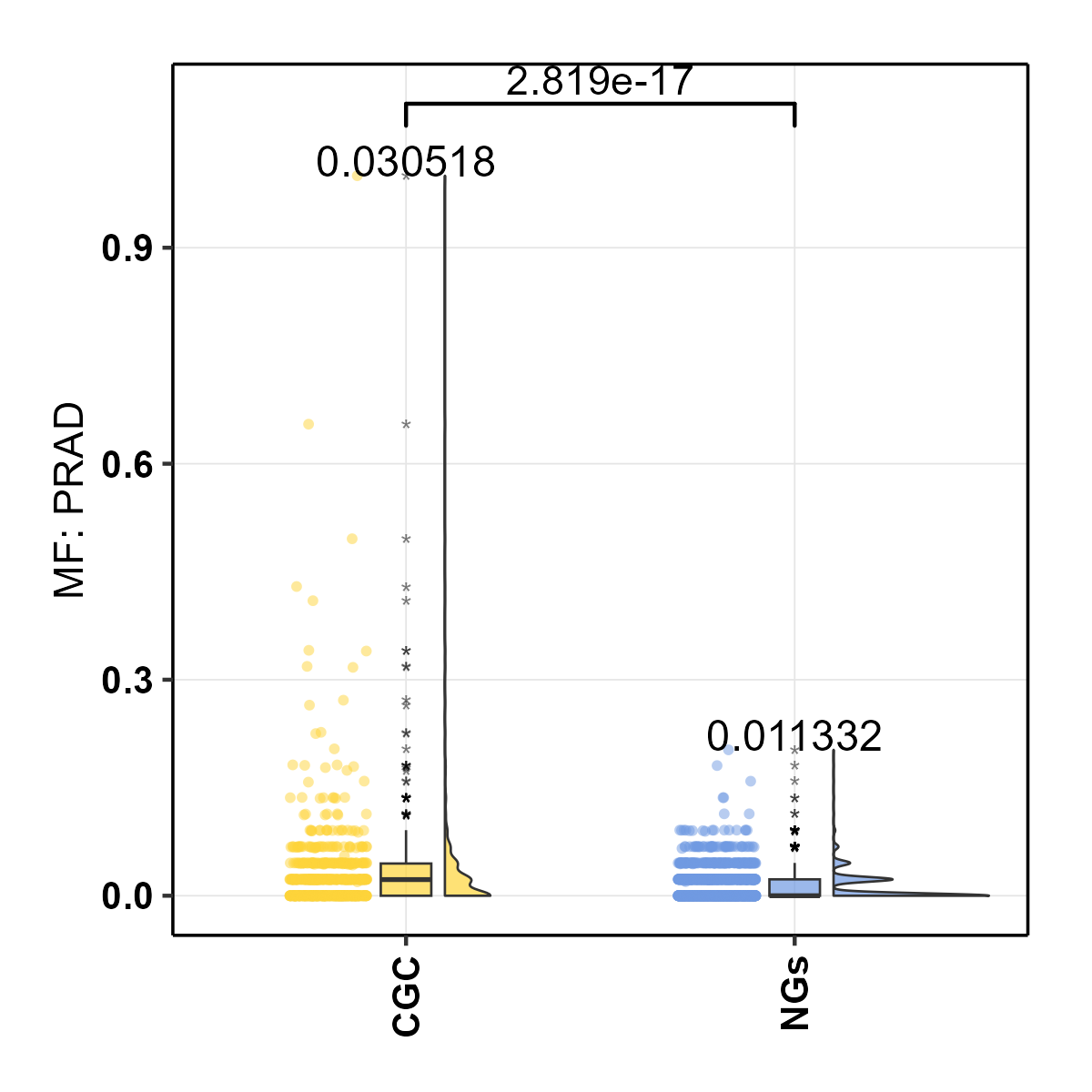

Supplement: Supplementary file 3 [file DataSheet1.ZIP › Supplementary file 5-1/IReflndex/MF_PRAD.png]

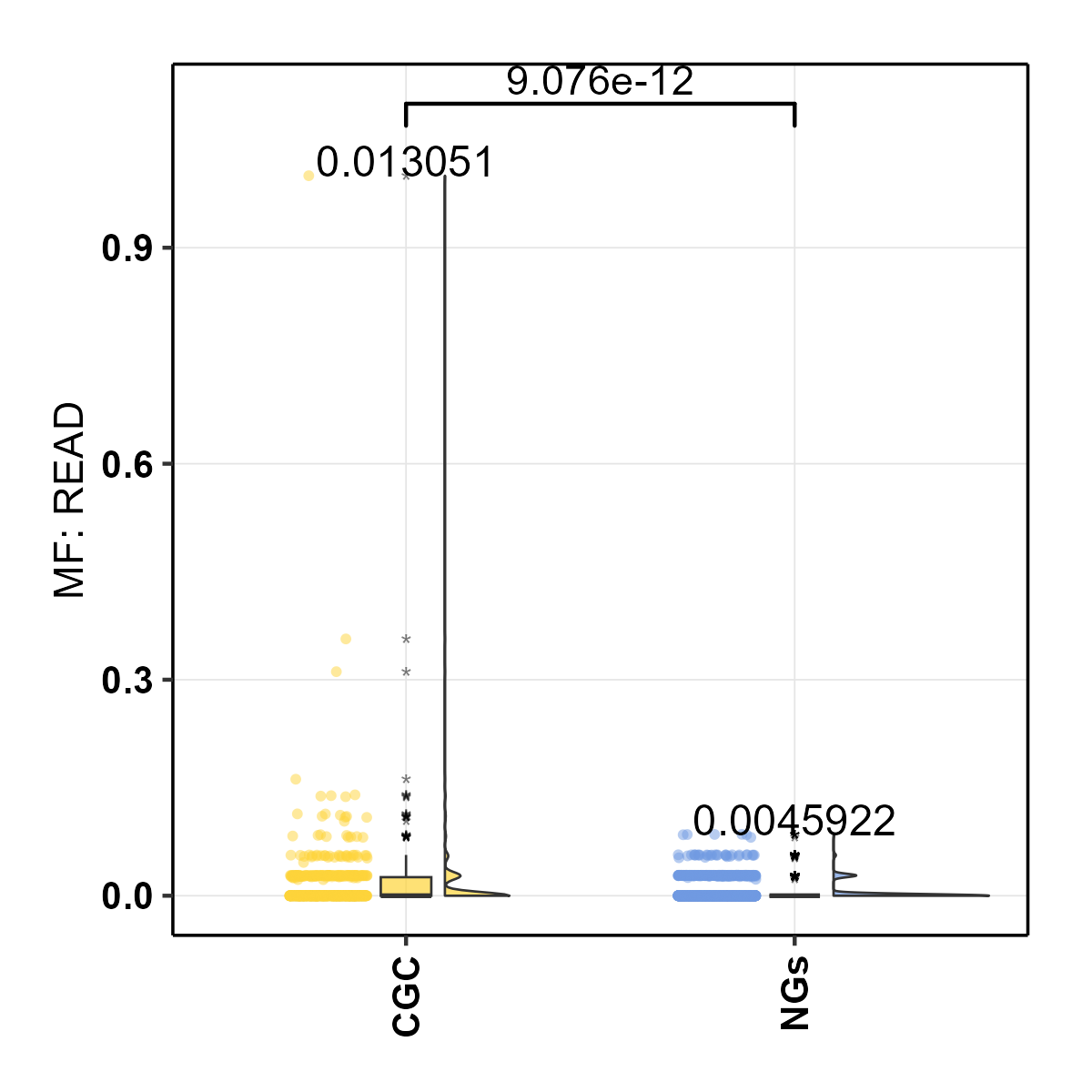

Supplement: Supplementary file 3 [file DataSheet1.ZIP › Supplementary file 5-1/IReflndex/MF_READ.png]

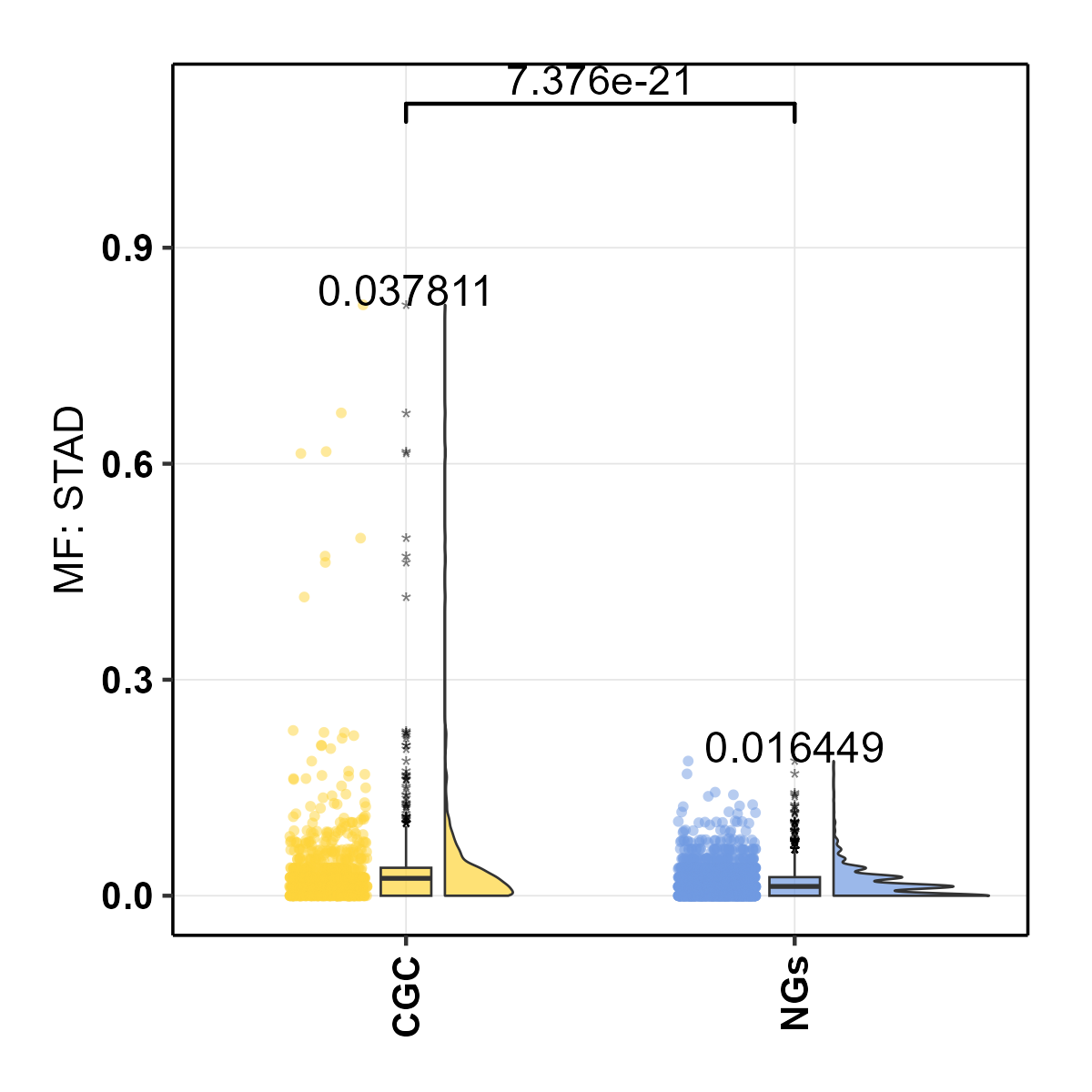

Supplement: Supplementary file 3 [file DataSheet1.ZIP › Supplementary file 5-1/IReflndex/MF_STAD.png]

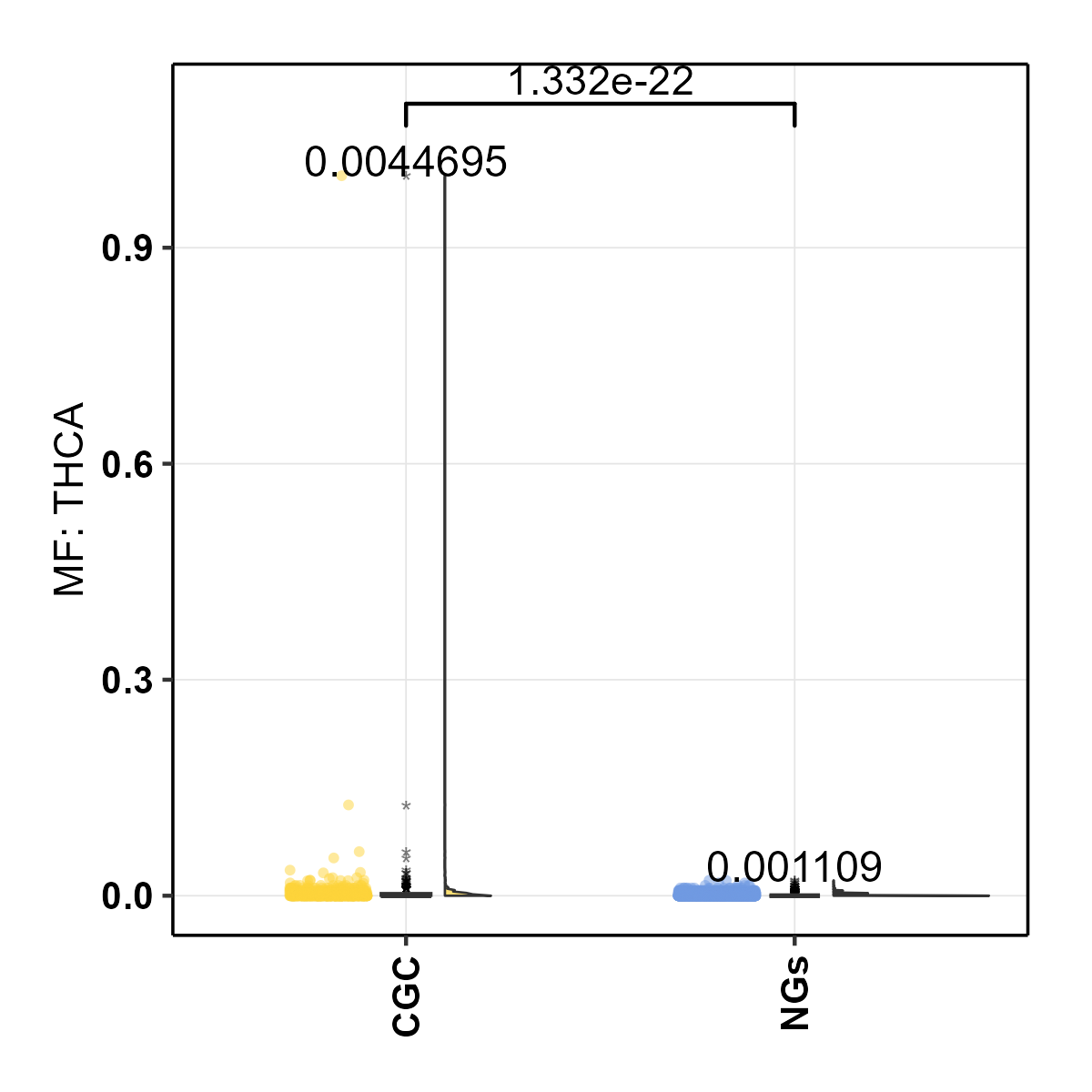

Supplement: Supplementary file 3 [file DataSheet1.ZIP › Supplementary file 5-1/IReflndex/MF_THCA.png]

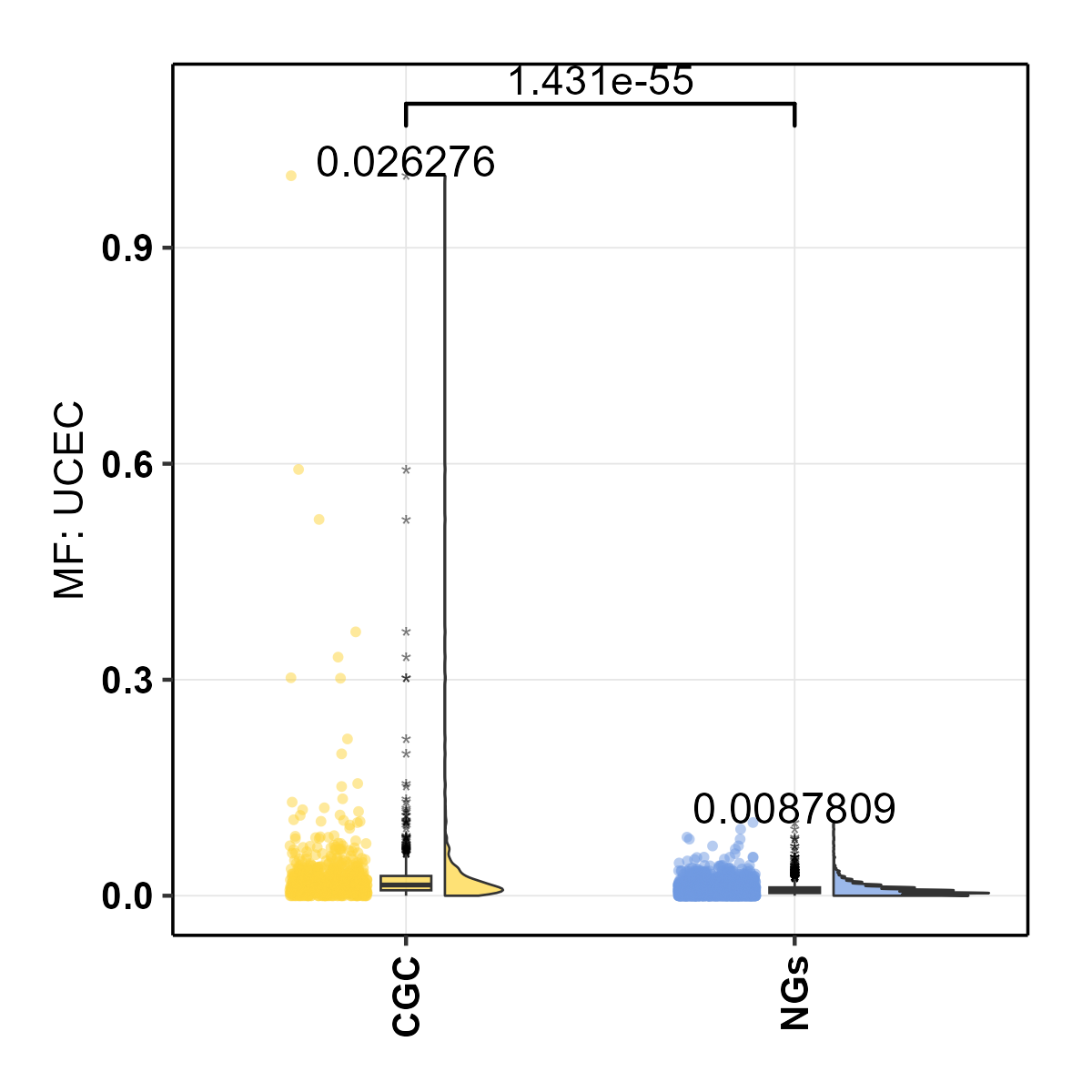

Supplement: Supplementary file 3 [file DataSheet1.ZIP › Supplementary file 5-1/IReflndex/MF_UCEC.png]

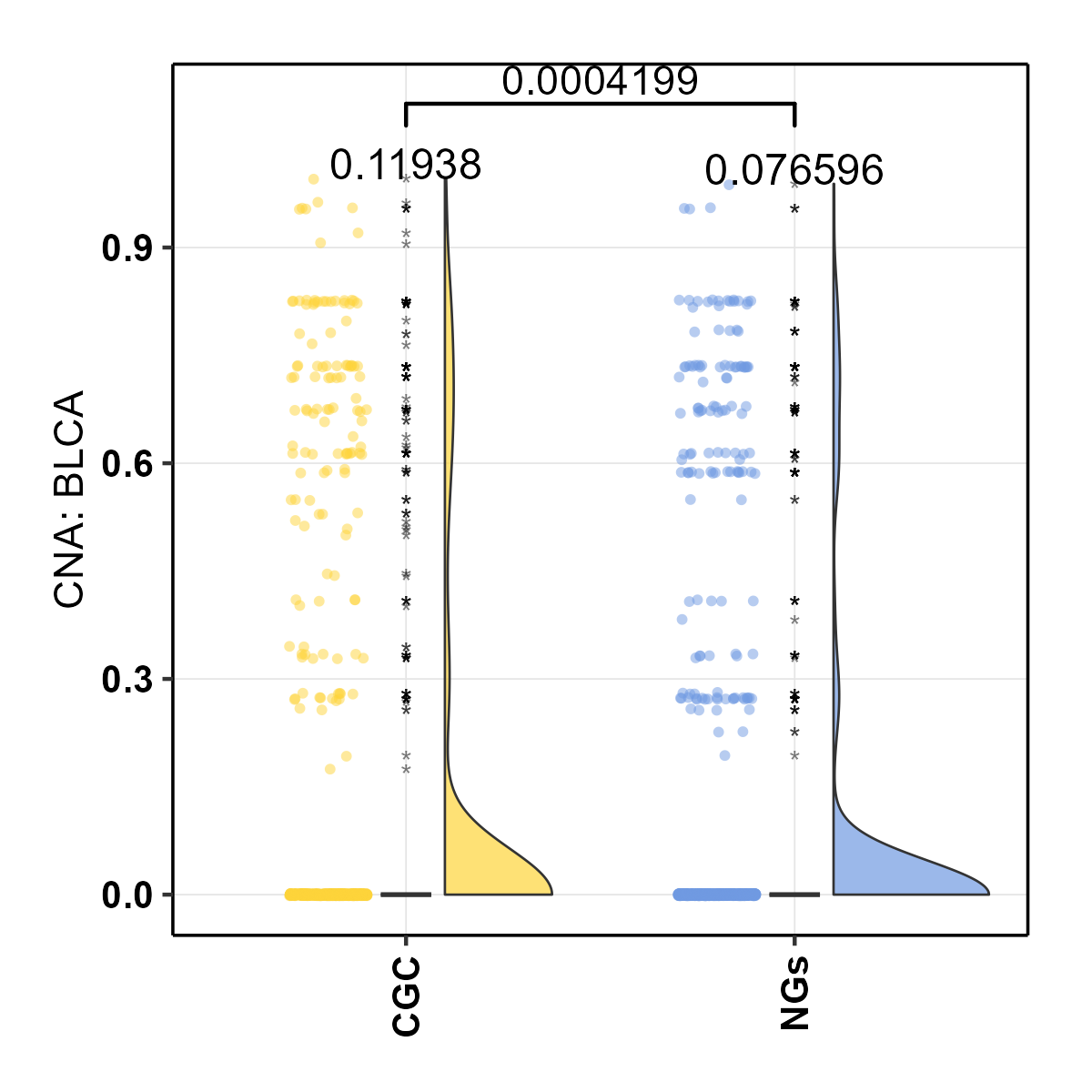

Supplement: Supplementary file 3 [file DataSheet1.ZIP › Supplementary file 5-1/IReflndex_2015/CNA_BLCA.png]

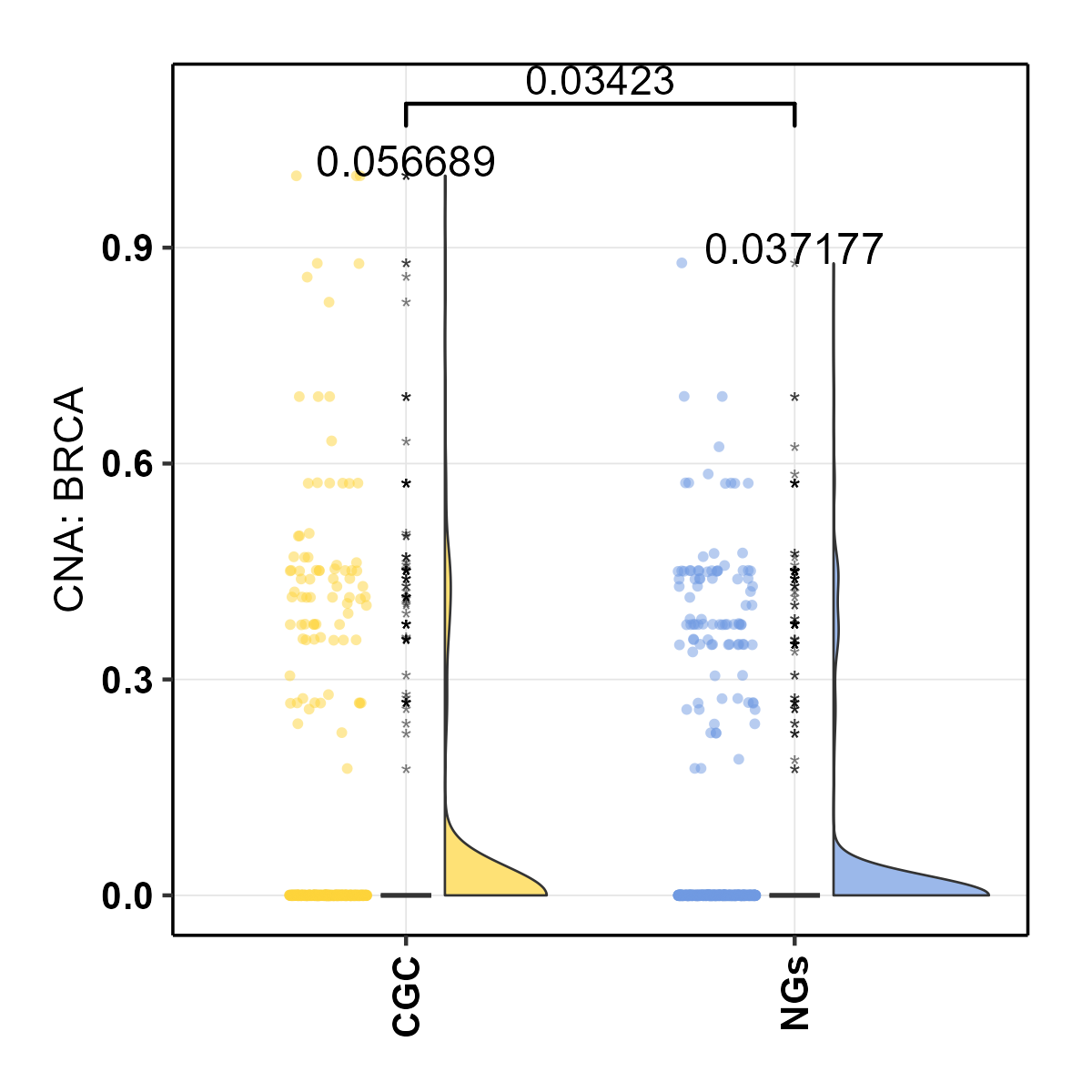

Supplement: Supplementary file 3 [file DataSheet1.ZIP › Supplementary file 5-1/IReflndex_2015/CNA_BRCA.png]

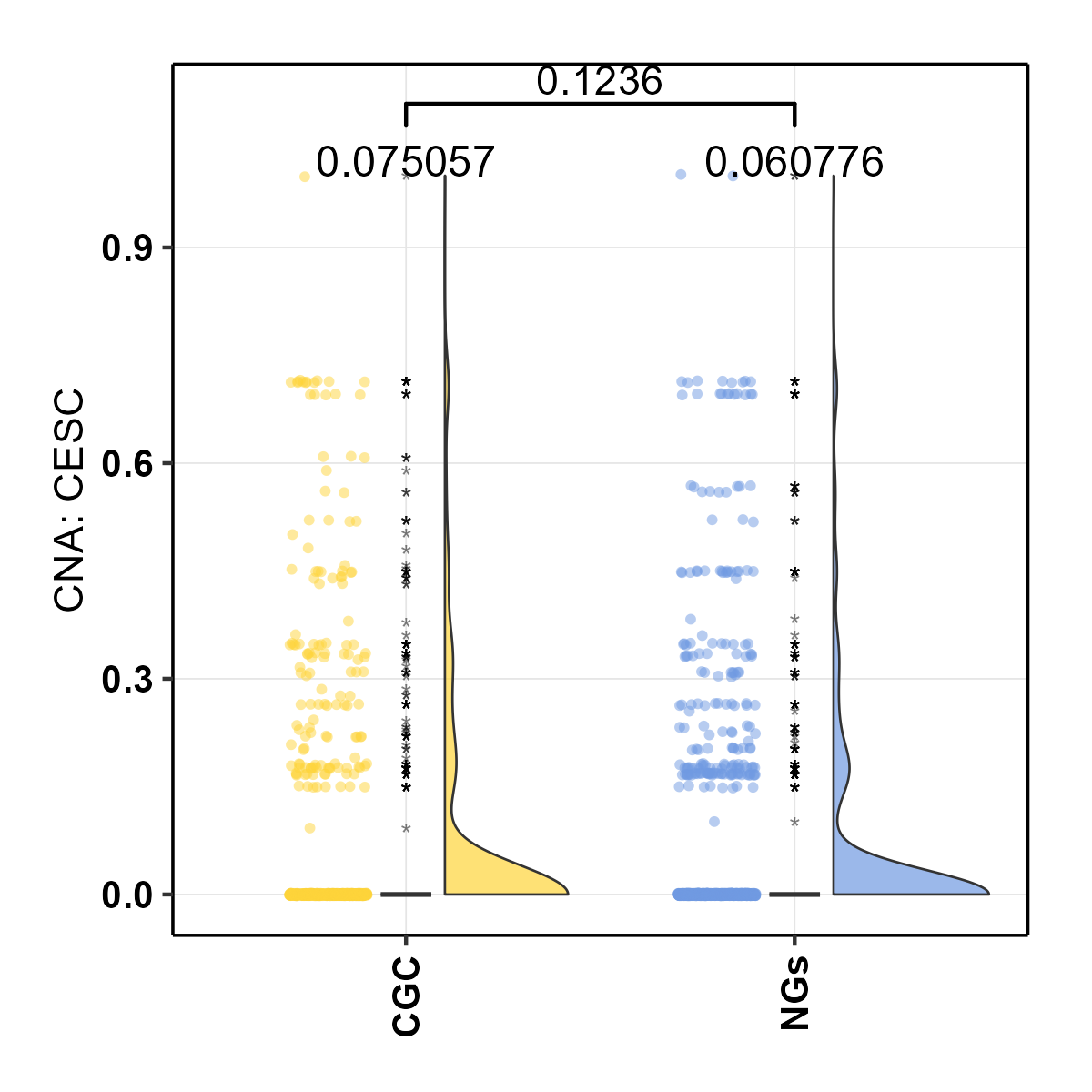

Supplement: Supplementary file 3 [file DataSheet1.ZIP › Supplementary file 5-1/IReflndex_2015/CNA_CESC.png]

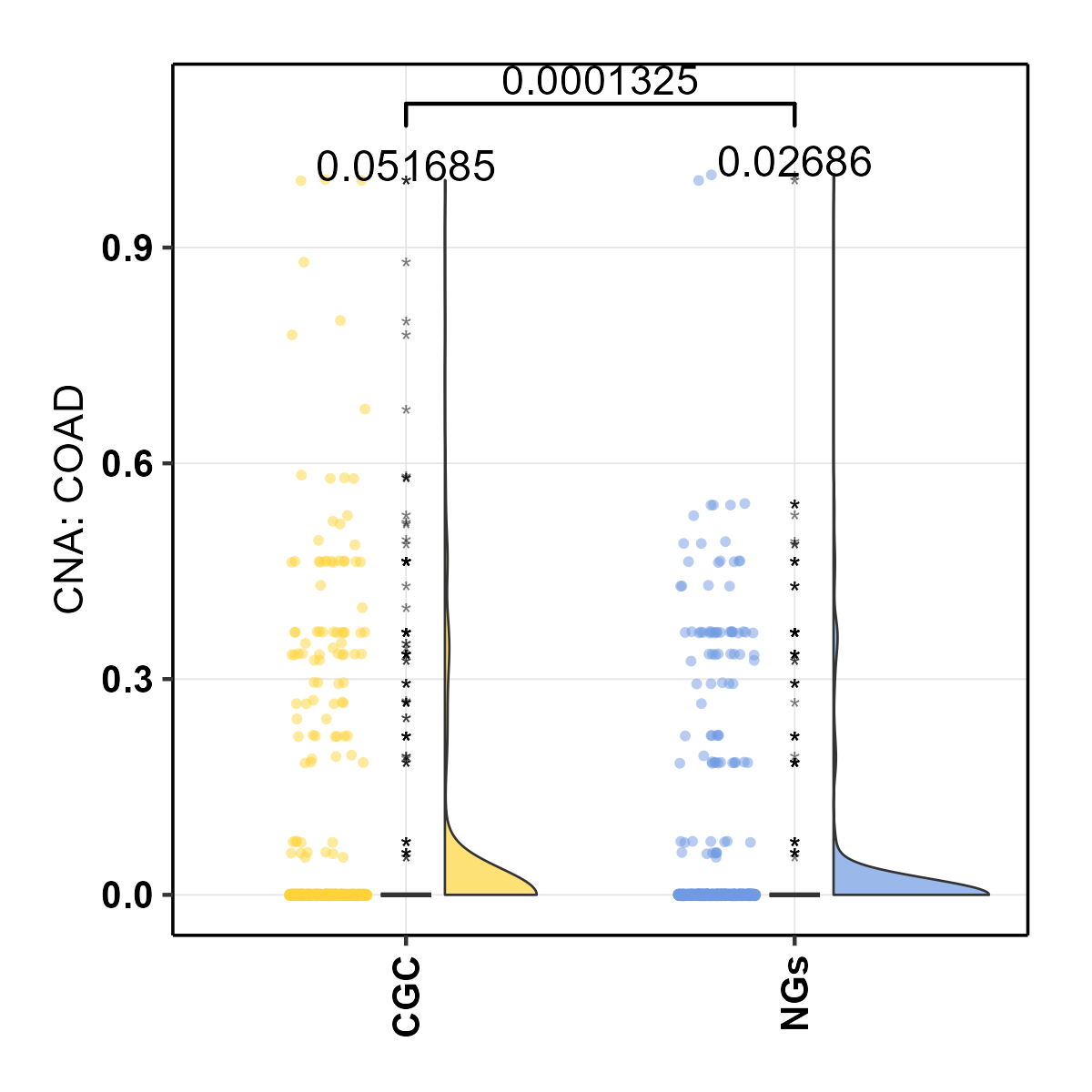

Supplement: Supplementary file 3 [file DataSheet1.ZIP › Supplementary file 5-1/IReflndex_2015/CNA_COAD.png]

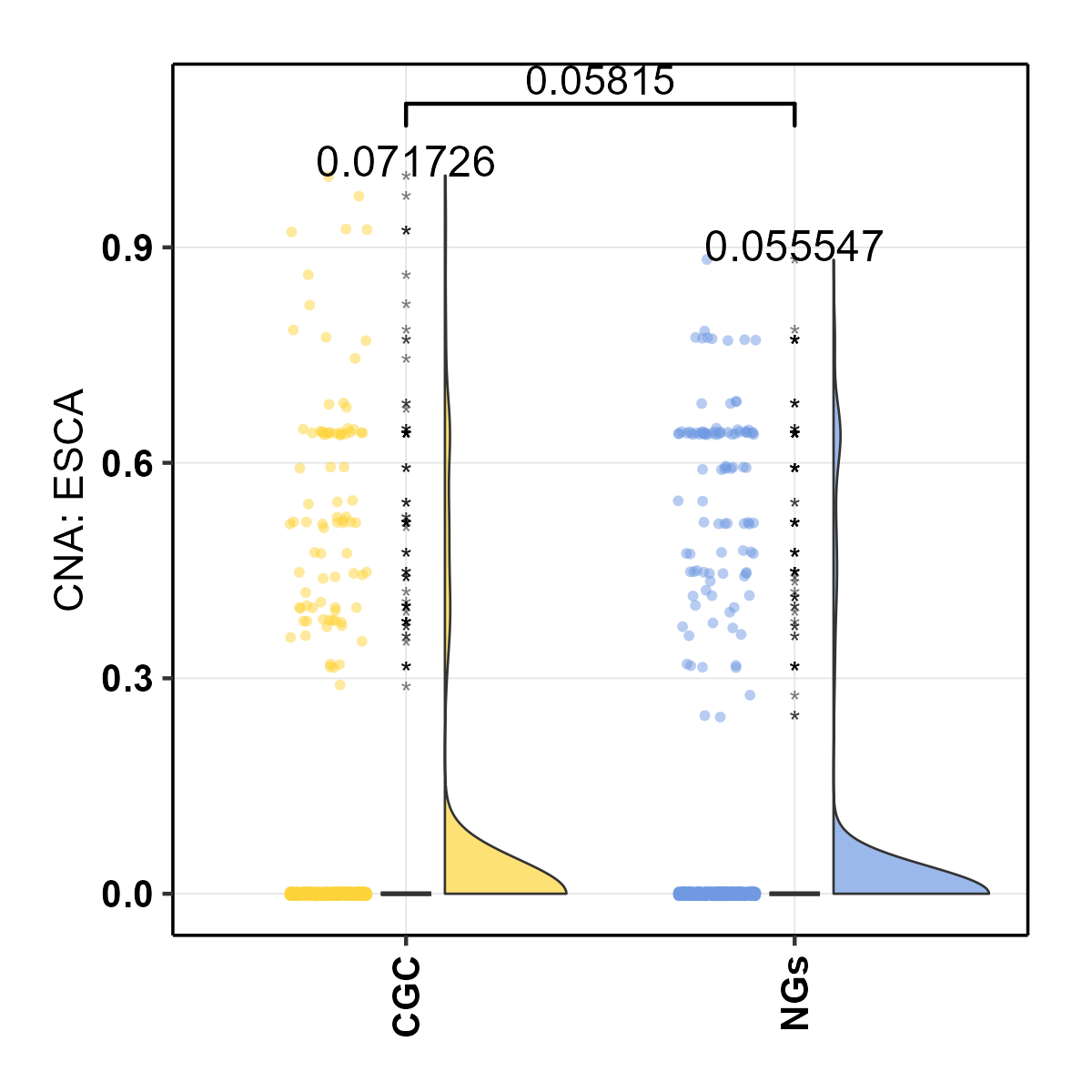

Supplement: Supplementary file 3 [file DataSheet1.ZIP › Supplementary file 5-1/IReflndex_2015/CNA_ESCA.png]

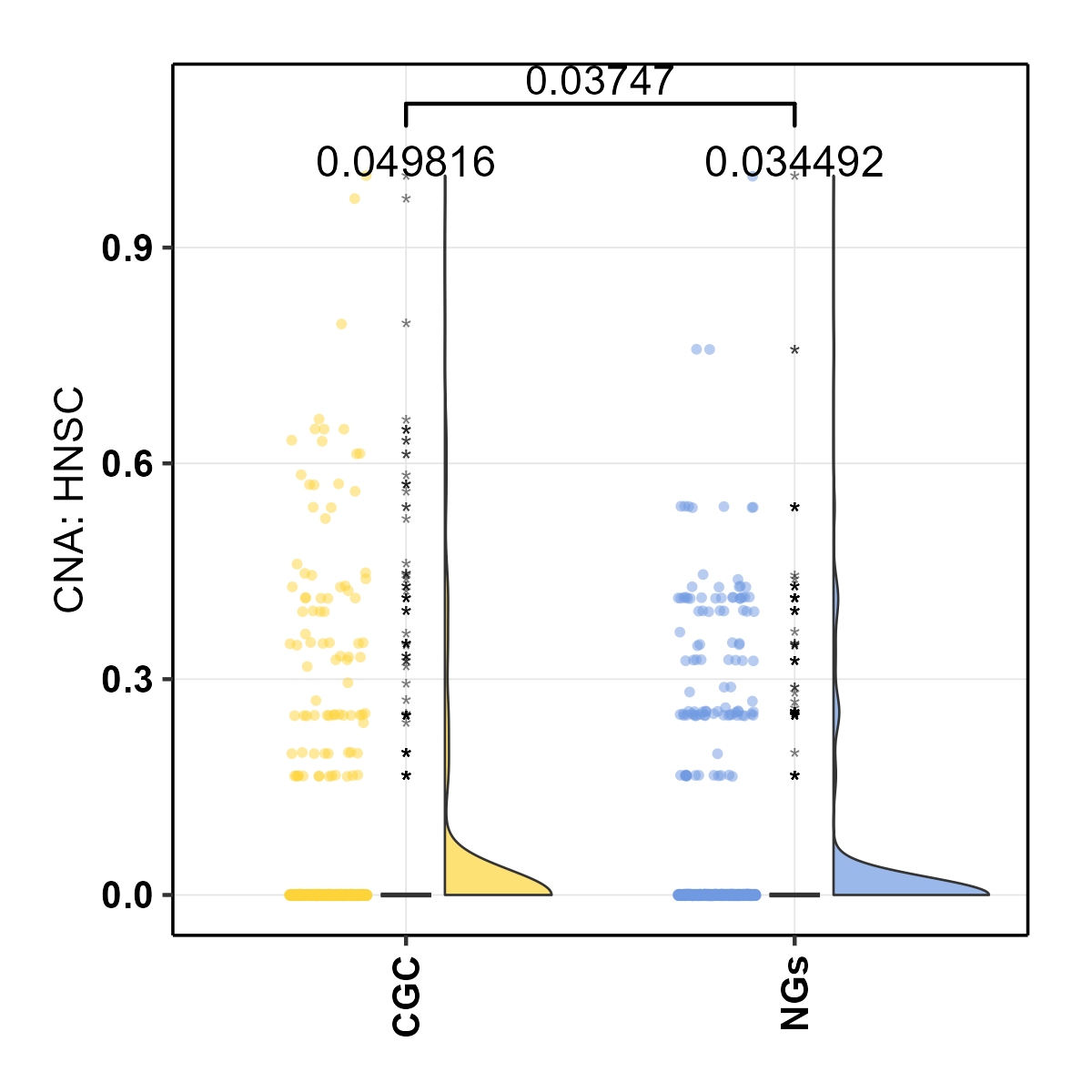

Supplement: Supplementary file 3 [file DataSheet1.ZIP › Supplementary file 5-1/IReflndex_2015/CNA_HNSC.png]

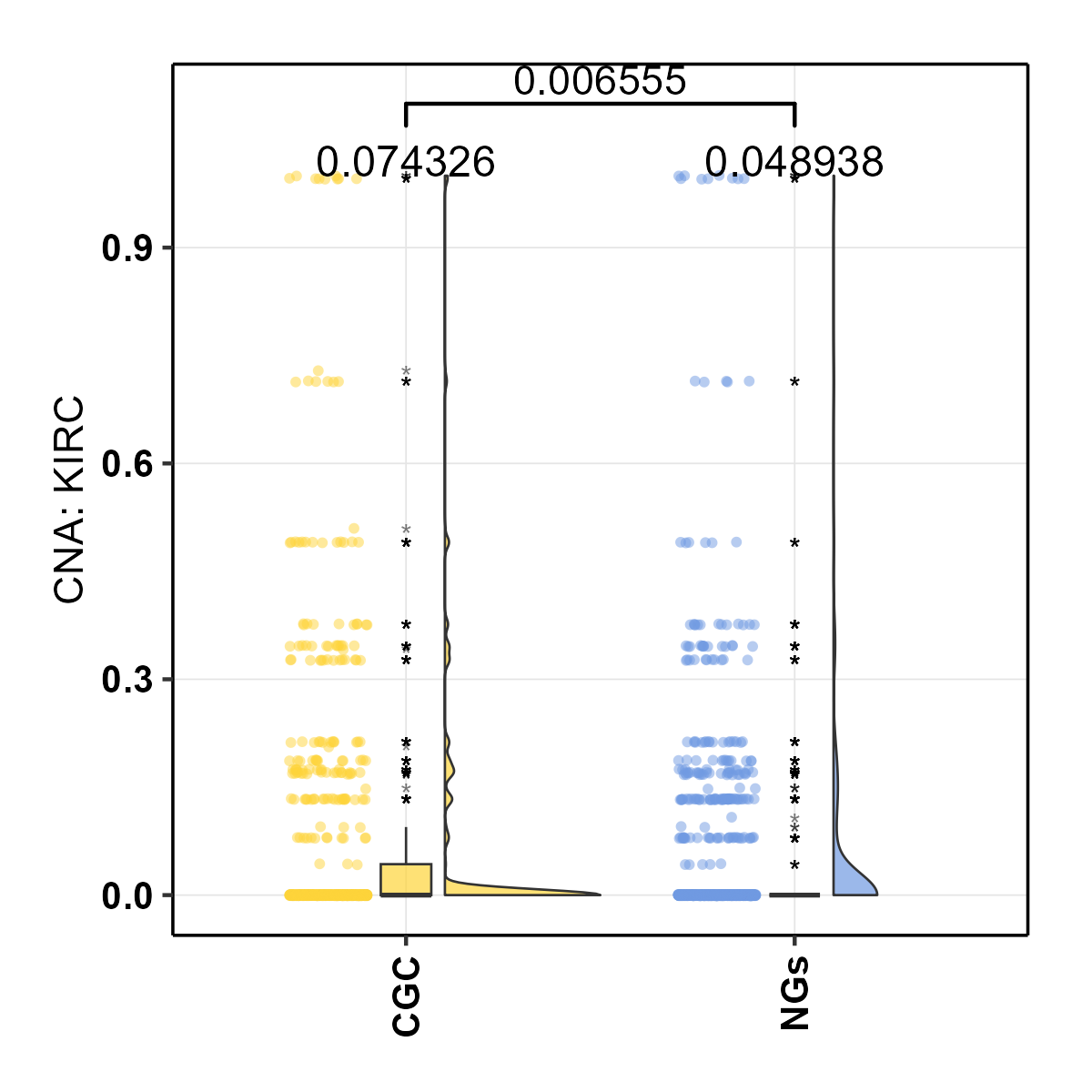

Supplement: Supplementary file 3 [file DataSheet1.ZIP › Supplementary file 5-1/IReflndex_2015/CNA_KIRC.png]

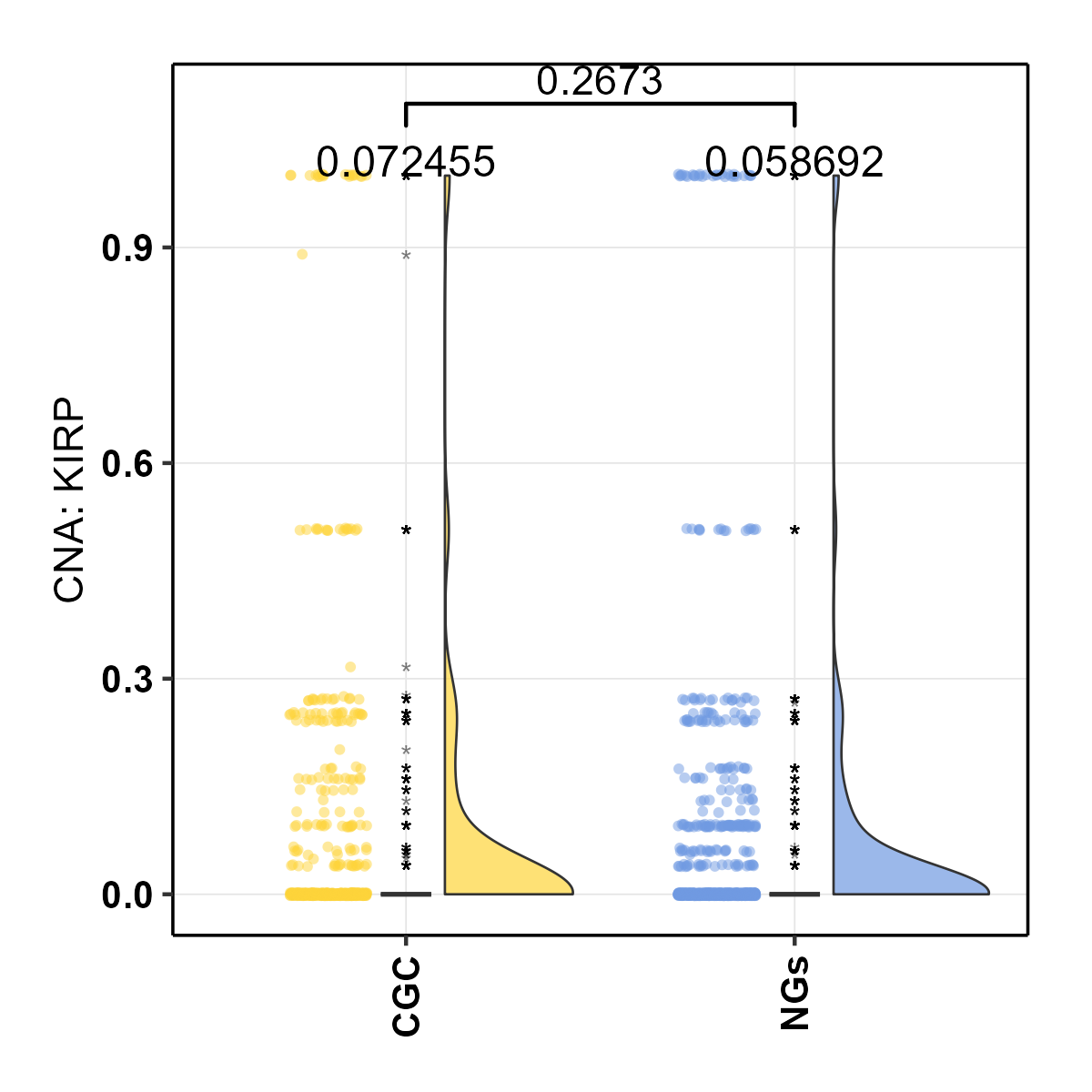

Supplement: Supplementary file 3 [file DataSheet1.ZIP › Supplementary file 5-1/IReflndex_2015/CNA_KIRP.png]

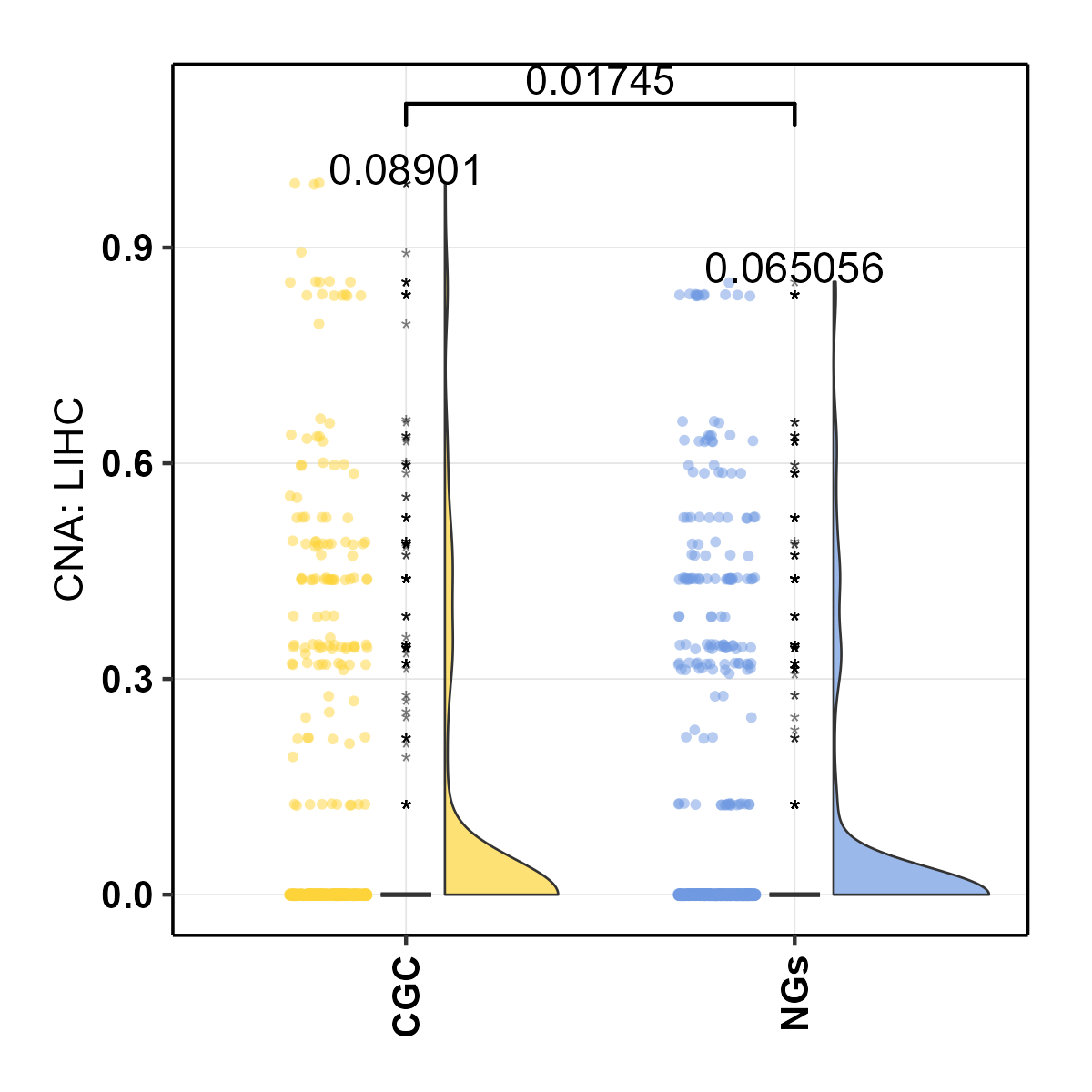

Supplement: Supplementary file 3 [file DataSheet1.ZIP › Supplementary file 5-1/IReflndex_2015/CNA_LIHC.png]

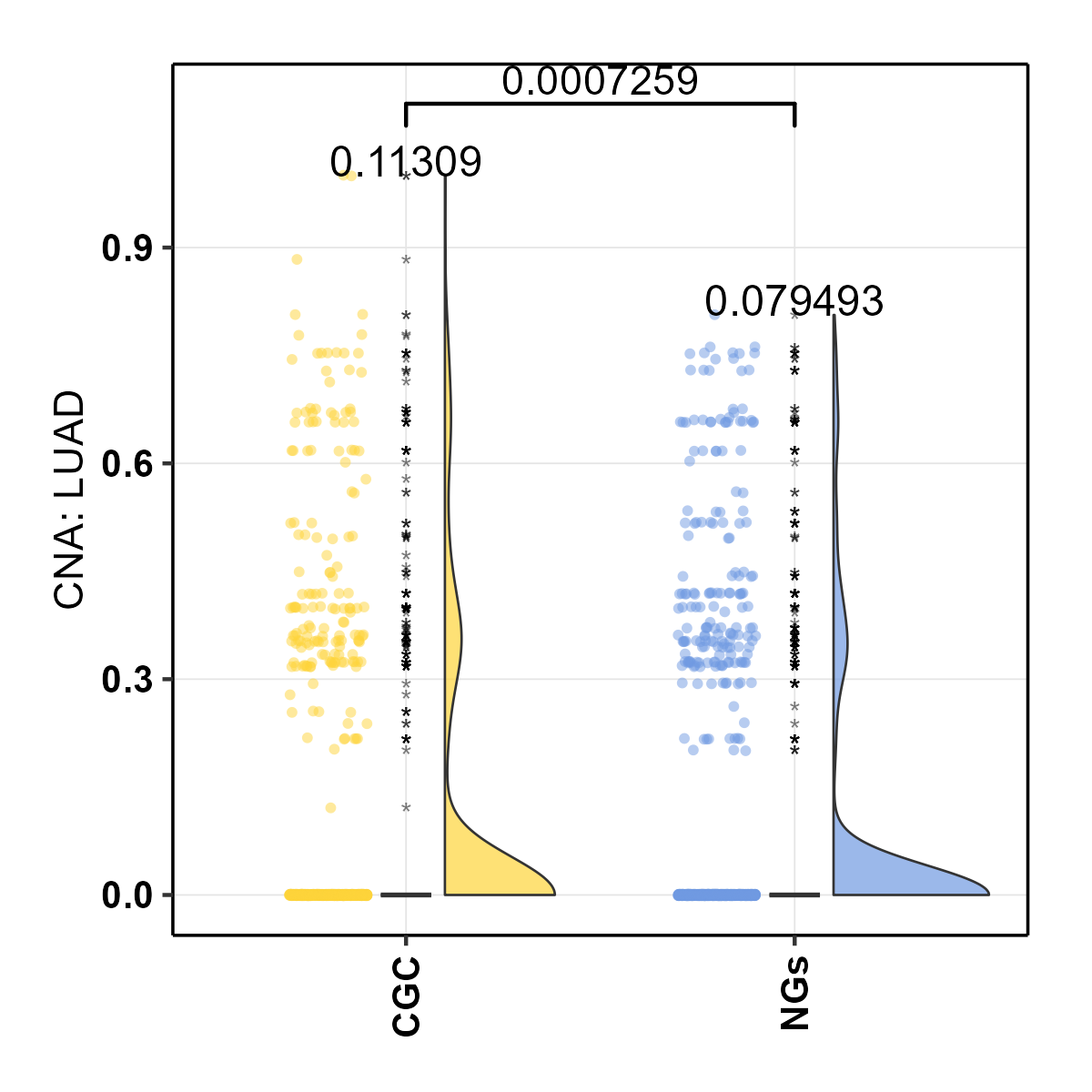

Supplement: Supplementary file 3 [file DataSheet1.ZIP › Supplementary file 5-1/IReflndex_2015/CNA_LUAD.png]

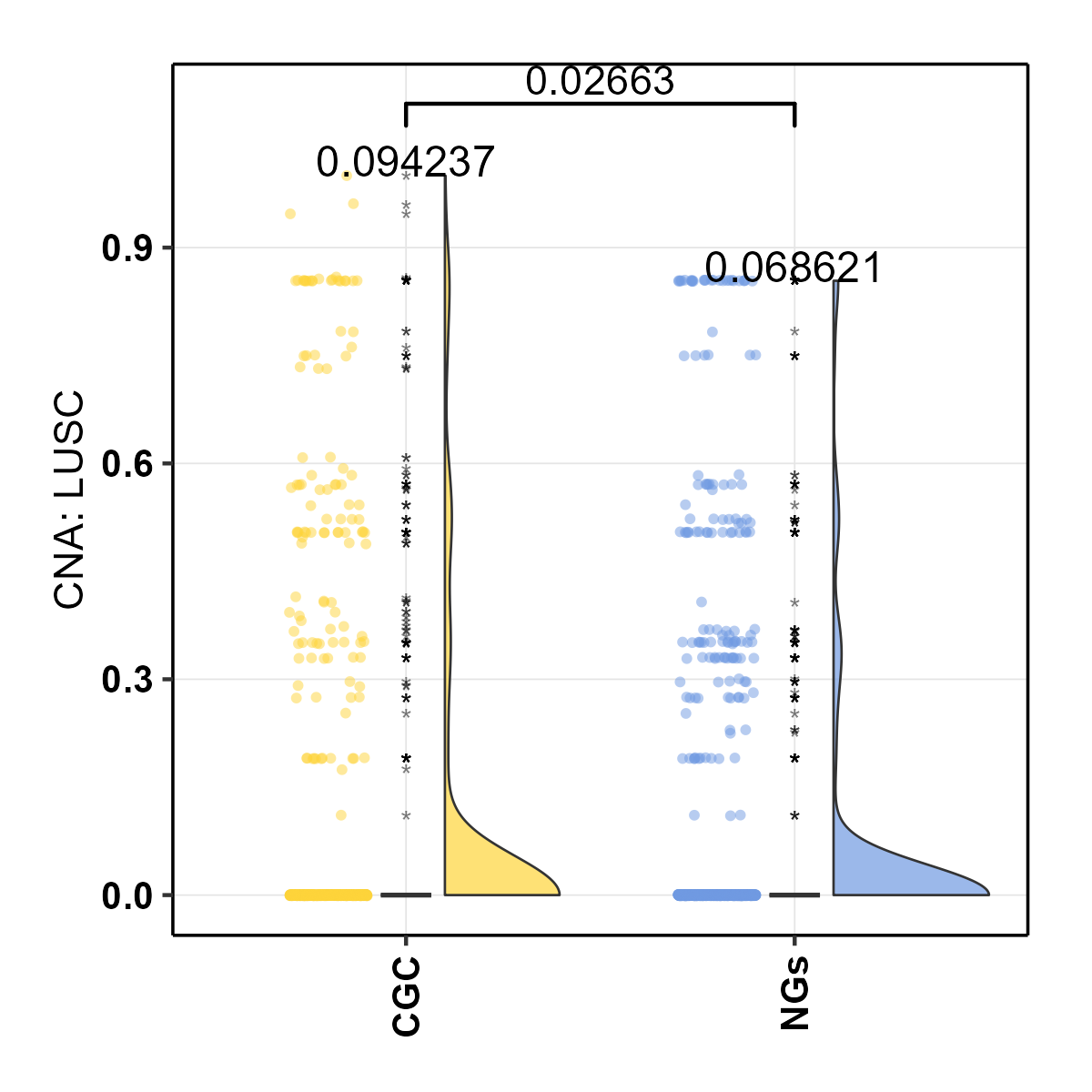

Supplement: Supplementary file 3 [file DataSheet1.ZIP › Supplementary file 5-1/IReflndex_2015/CNA_LUSC.png]

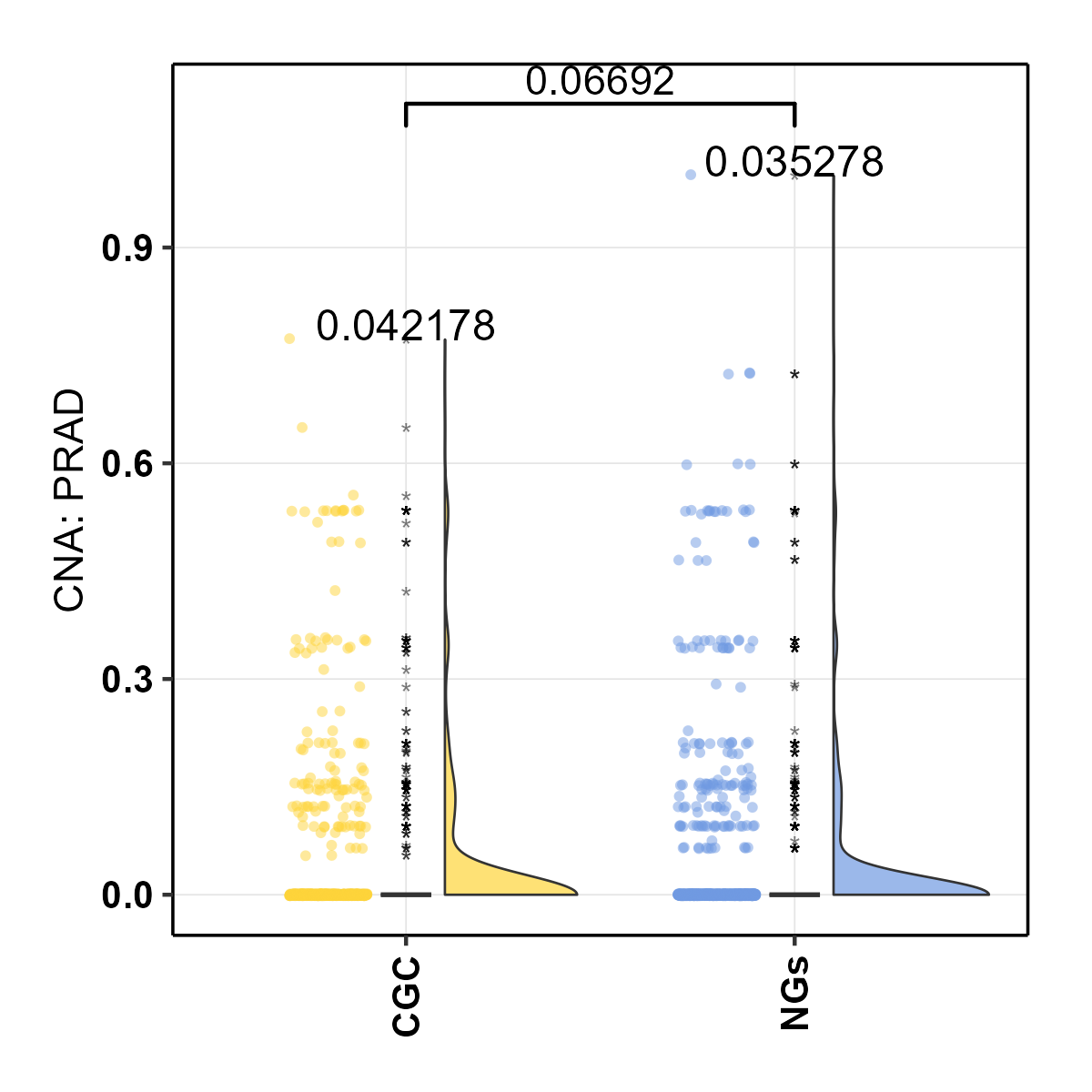

Supplement: Supplementary file 3 [file DataSheet1.ZIP › Supplementary file 5-1/IReflndex_2015/CNA_PRAD.png]

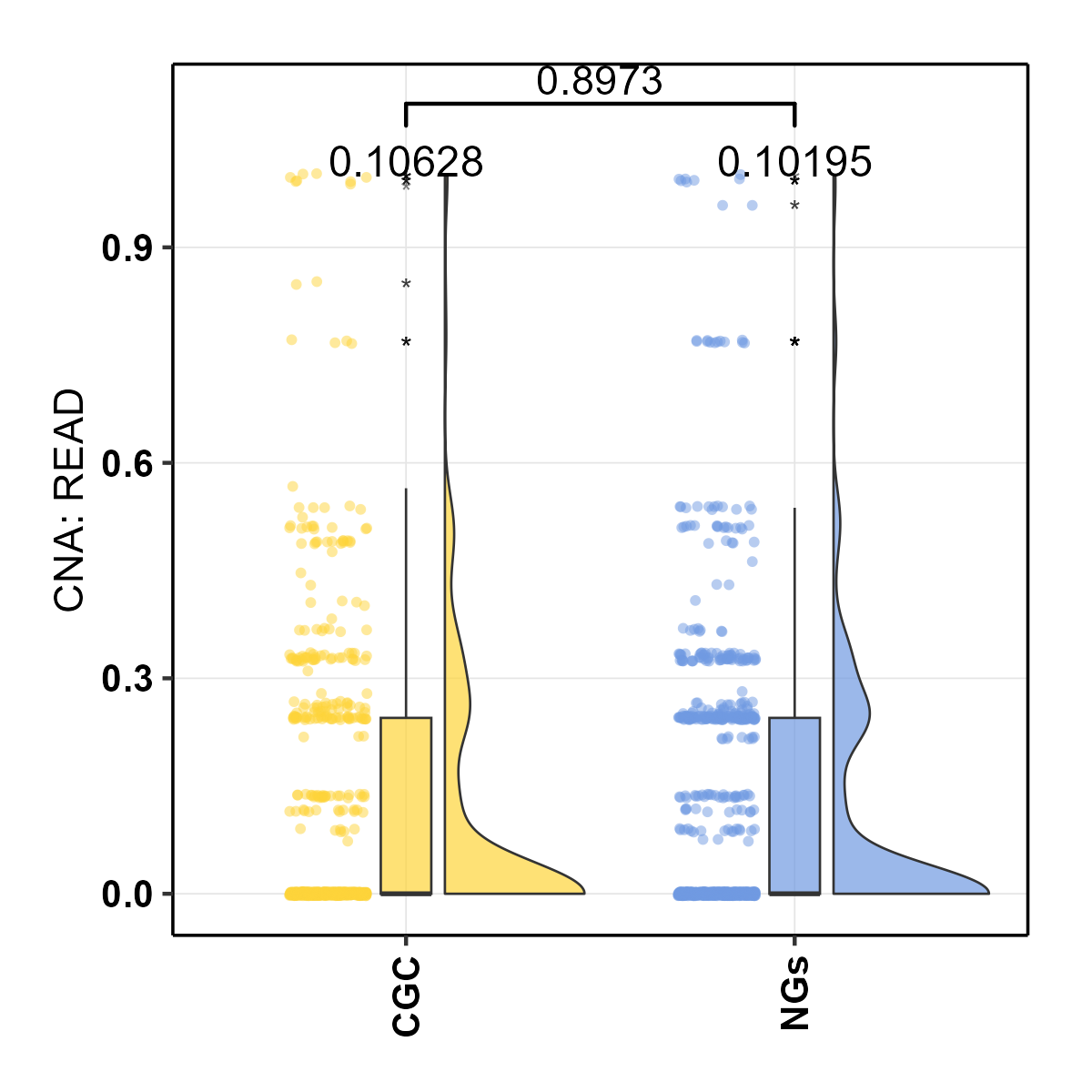

Supplement: Supplementary file 3 [file DataSheet1.ZIP › Supplementary file 5-1/IReflndex_2015/CNA_READ.png]

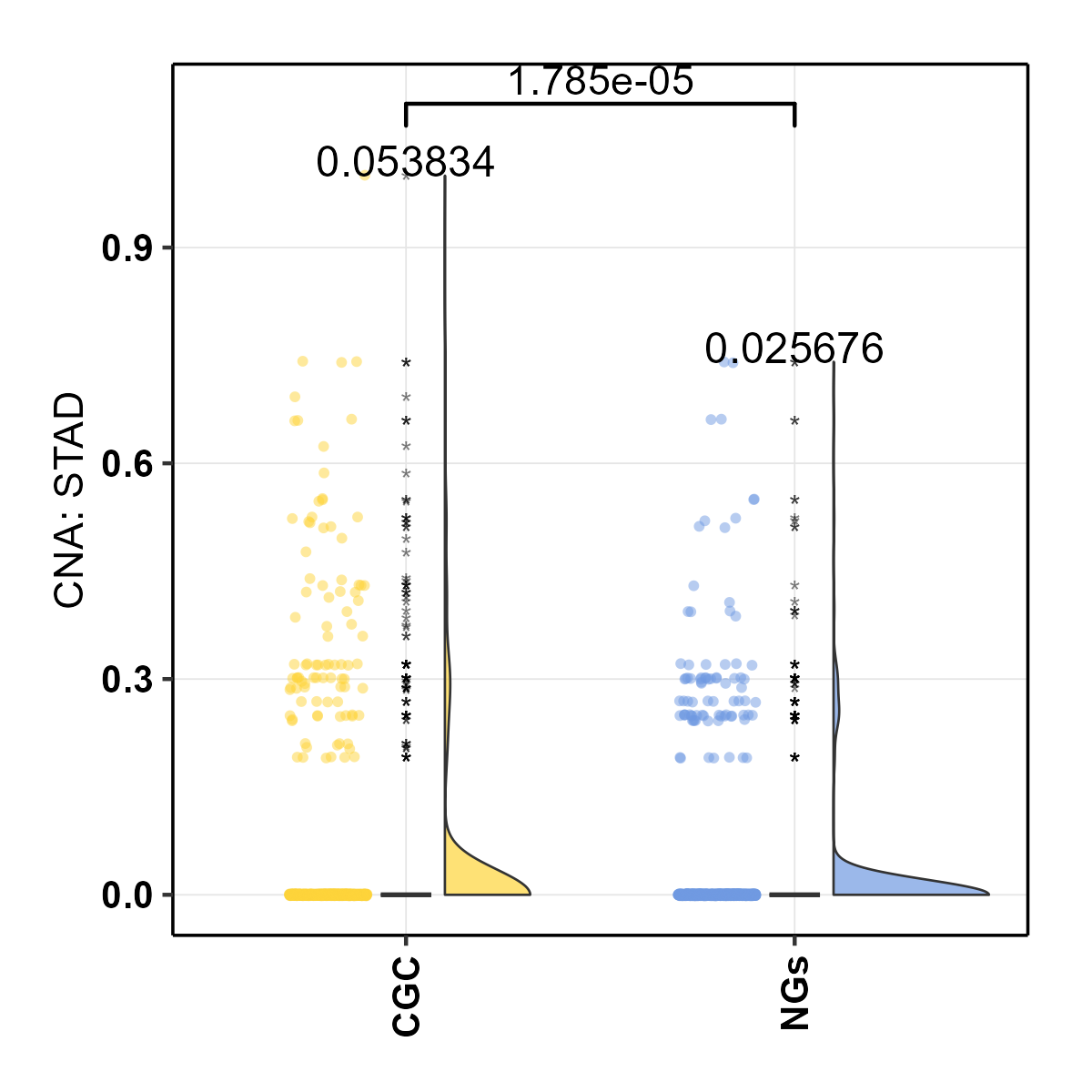

Supplement: Supplementary file 3 [file DataSheet1.ZIP › Supplementary file 5-1/IReflndex_2015/CNA_STAD.png]

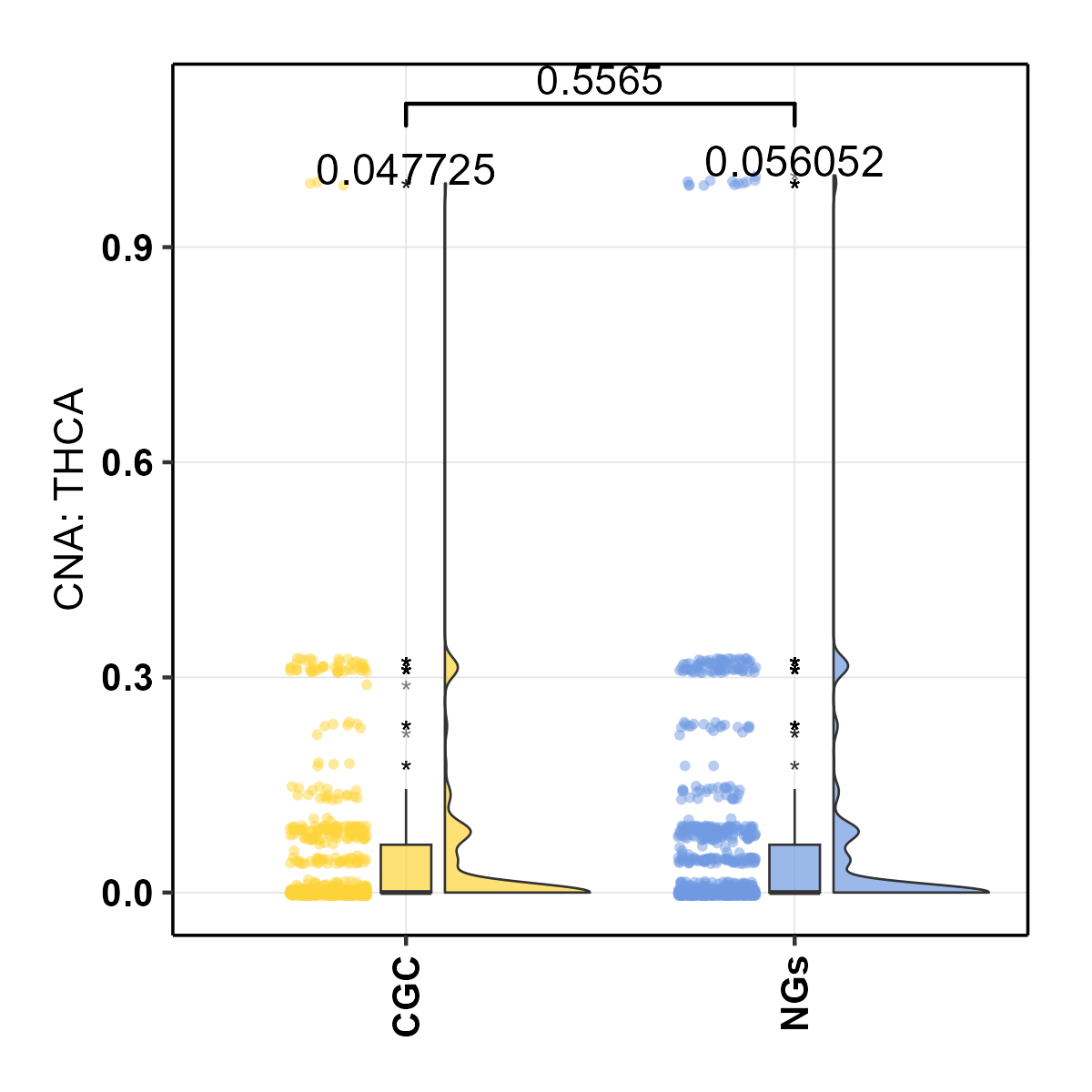

Supplement: Supplementary file 3 [file DataSheet1.ZIP › Supplementary file 5-1/IReflndex_2015/CNA_THCA.png]

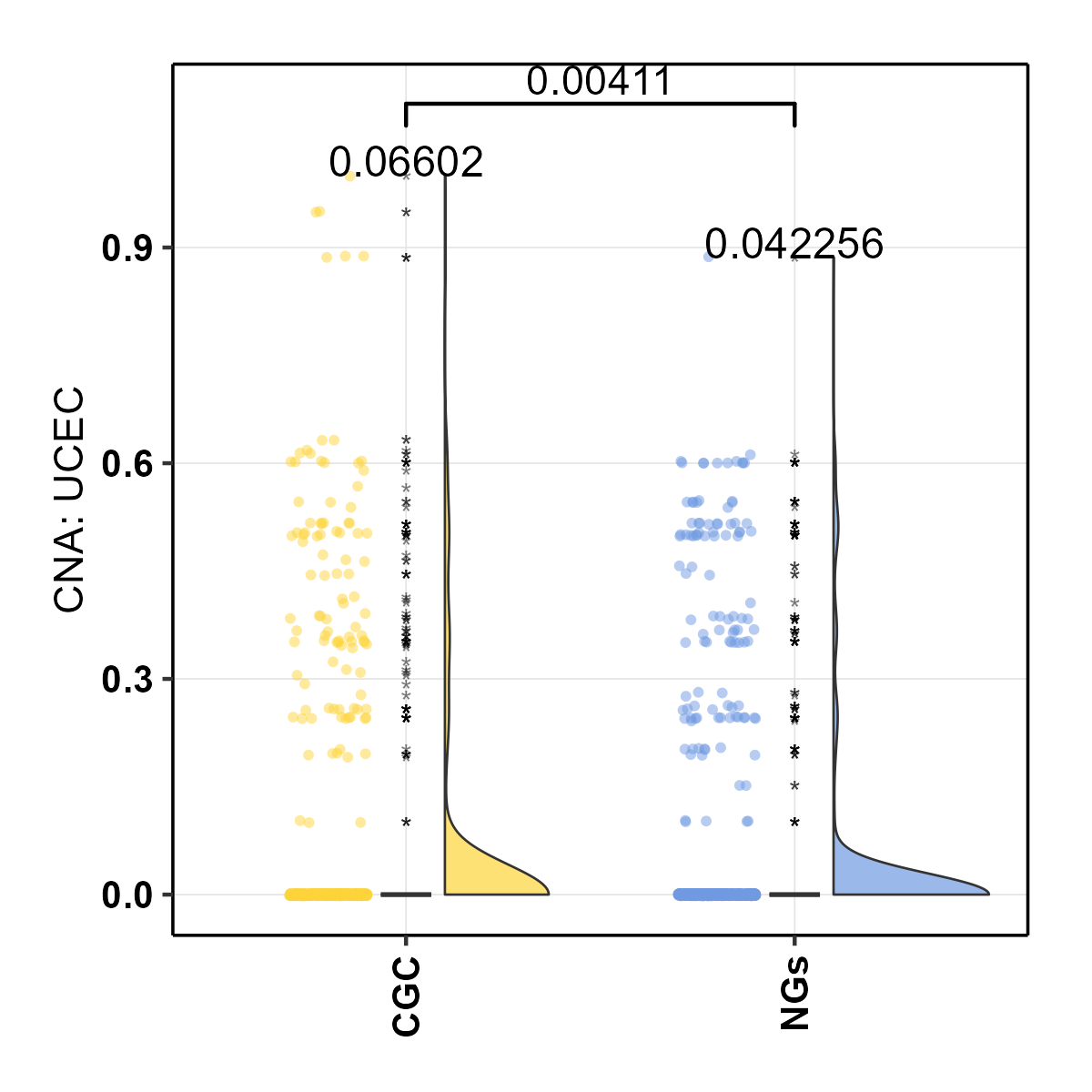

Supplement: Supplementary file 3 [file DataSheet1.ZIP › Supplementary file 5-1/IReflndex_2015/CNA_UCEC.png]

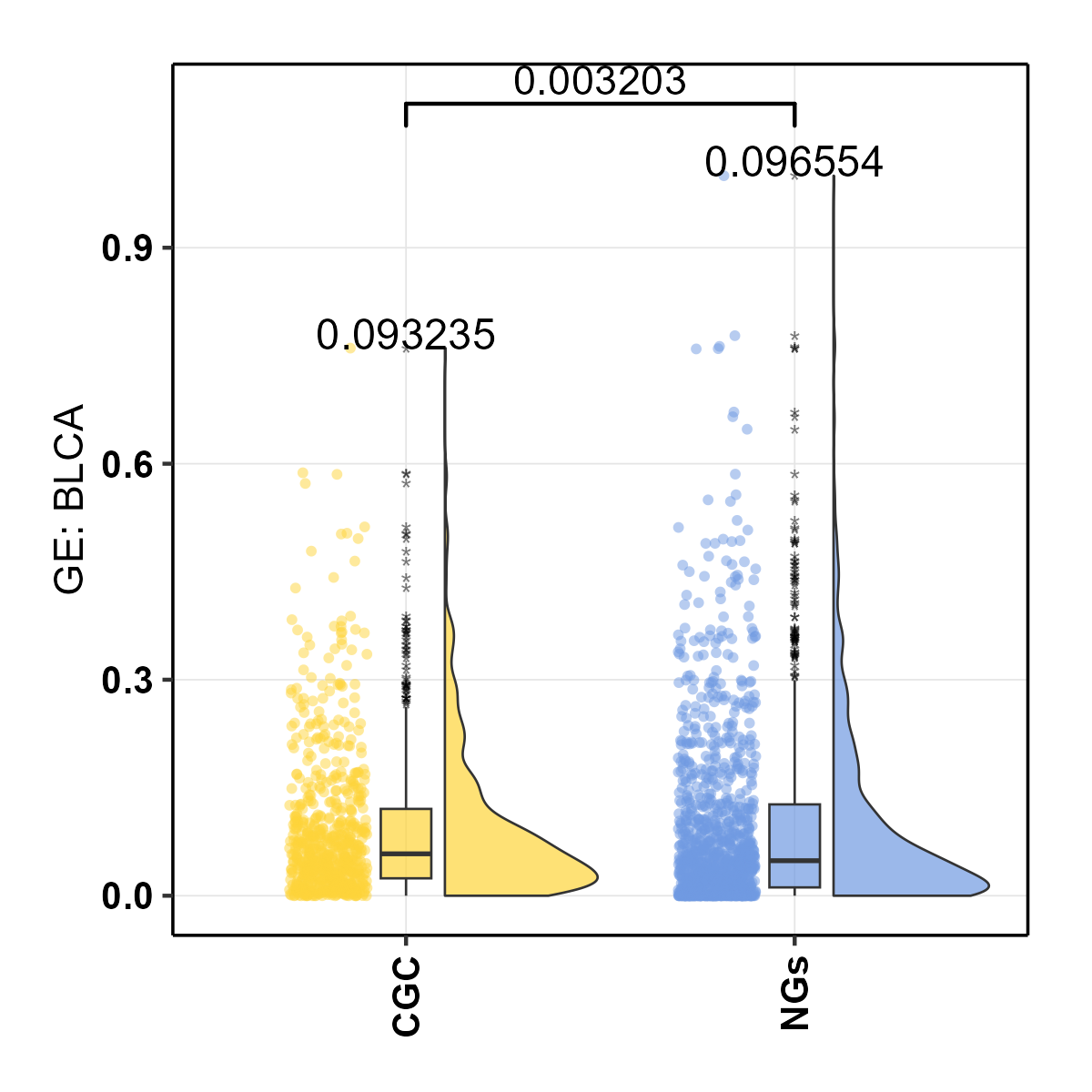

Supplement: Supplementary file 3 [file DataSheet1.ZIP › Supplementary file 5-1/IReflndex_2015/GE_BLCA.png]

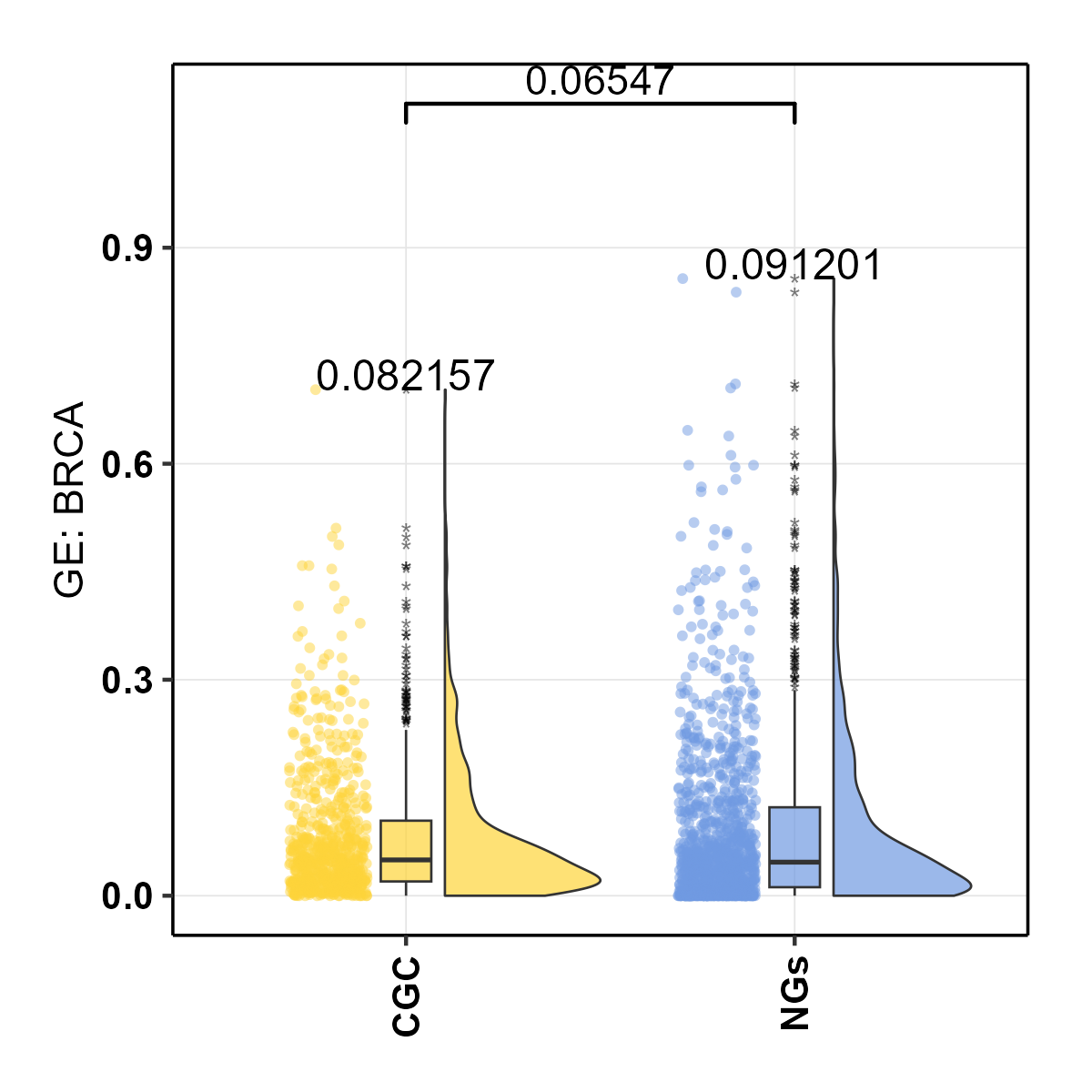

Supplement: Supplementary file 3 [file DataSheet1.ZIP › Supplementary file 5-1/IReflndex_2015/GE_BRCA.png]

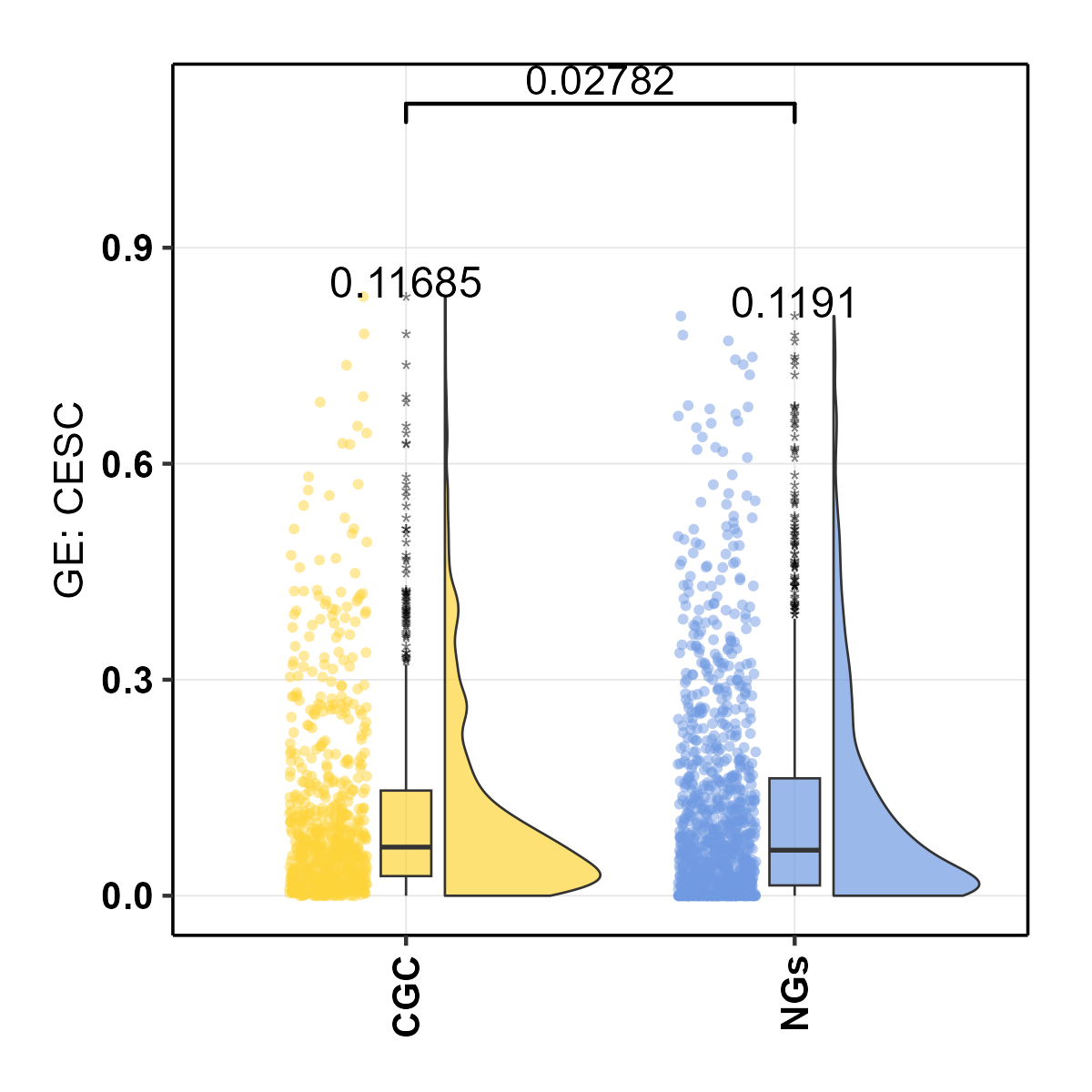

Supplement: Supplementary file 3 [file DataSheet1.ZIP › Supplementary file 5-1/IReflndex_2015/GE_CESC.png]

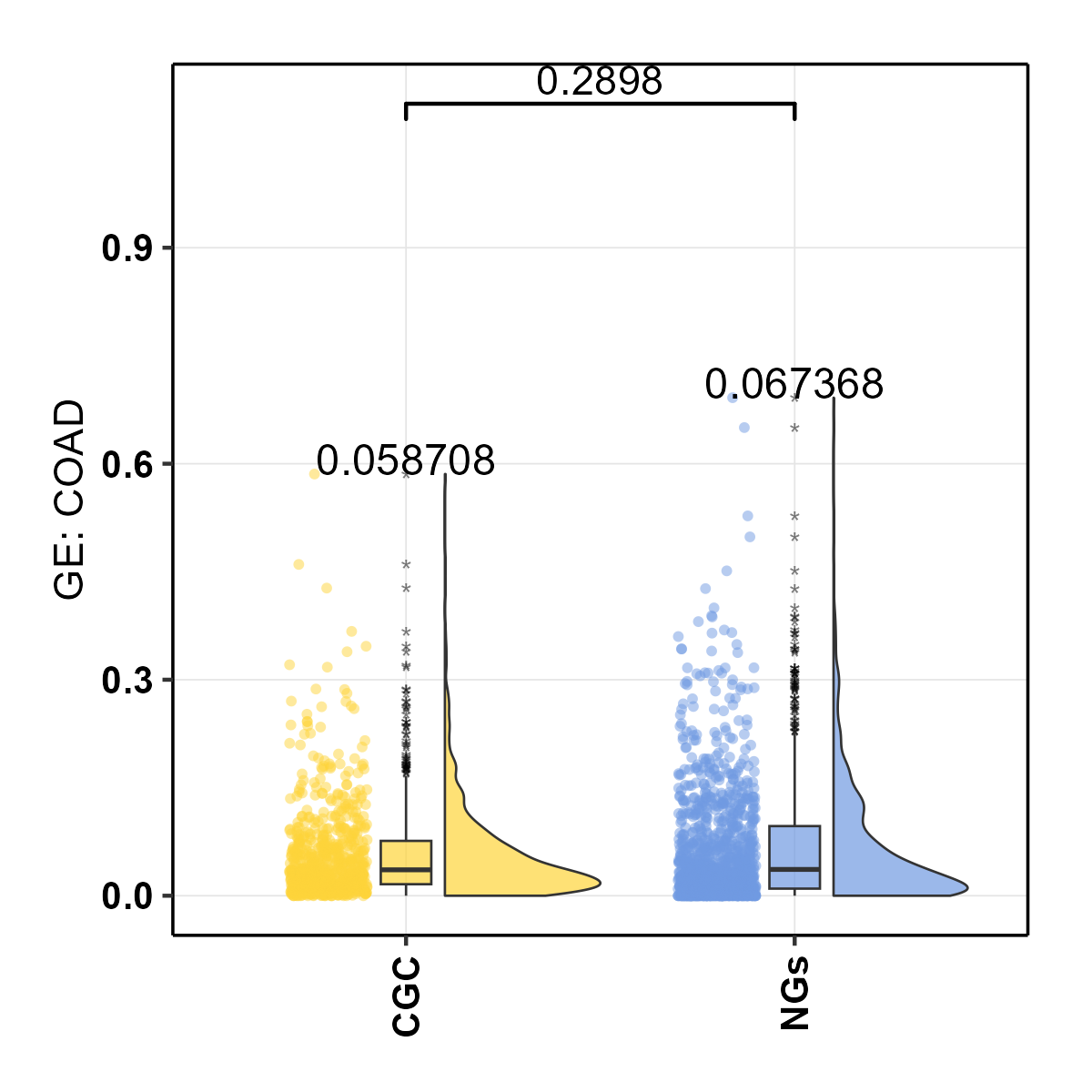

Supplement: Supplementary file 3 [file DataSheet1.ZIP › Supplementary file 5-1/IReflndex_2015/GE_COAD.png]

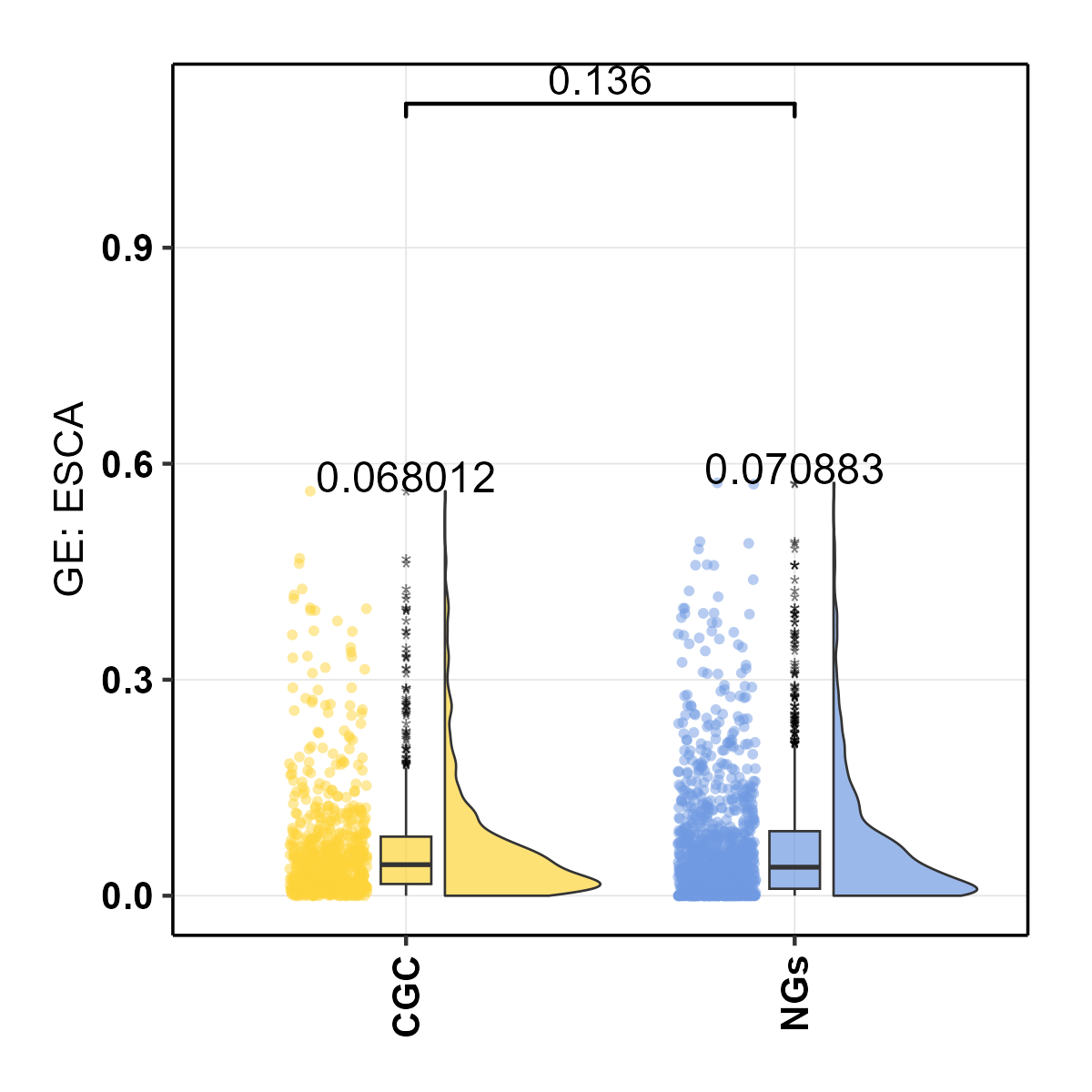

Supplement: Supplementary file 3 [file DataSheet1.ZIP › Supplementary file 5-1/IReflndex_2015/GE_ESCA.png]

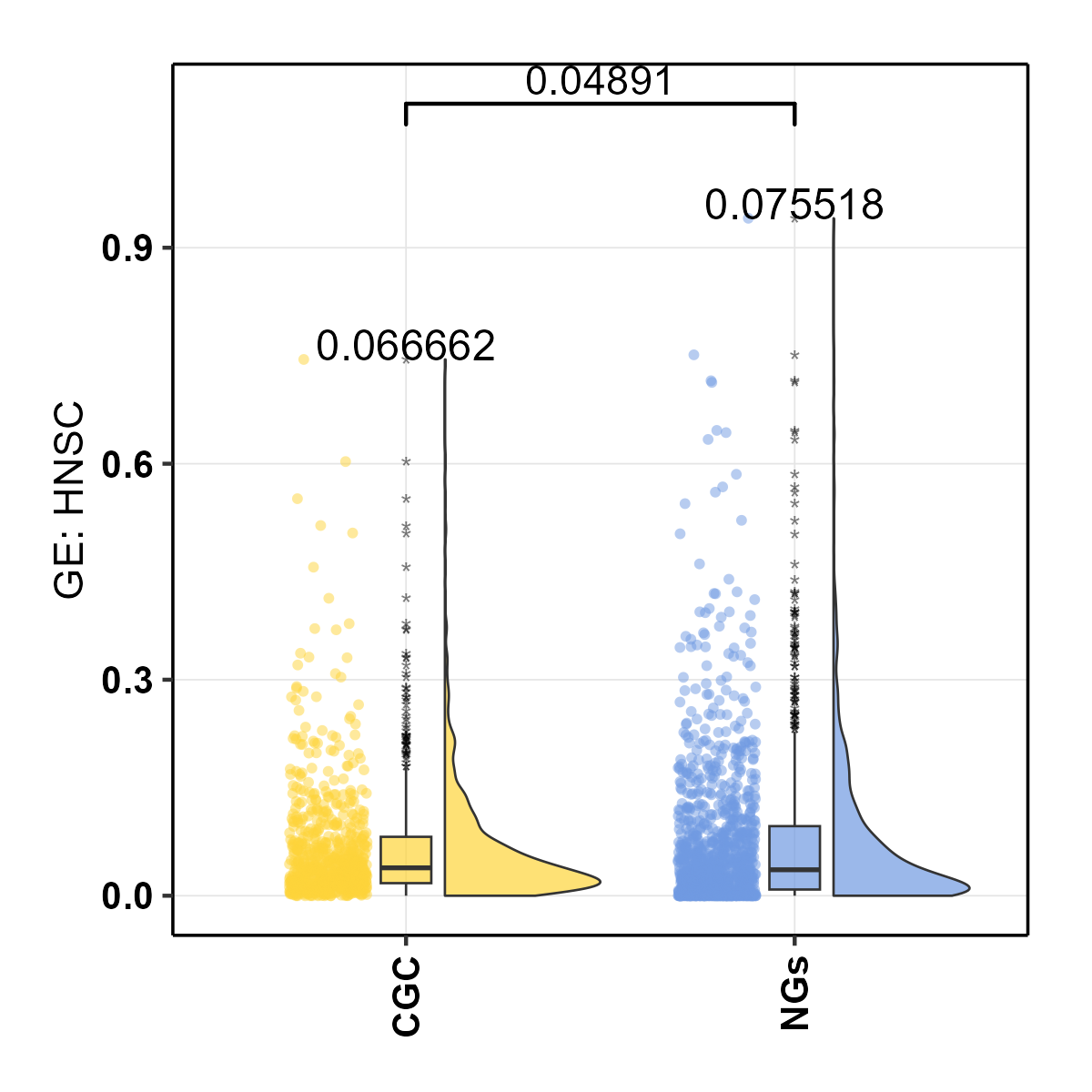

Supplement: Supplementary file 3 [file DataSheet1.ZIP › Supplementary file 5-1/IReflndex_2015/GE_HNSC.png]

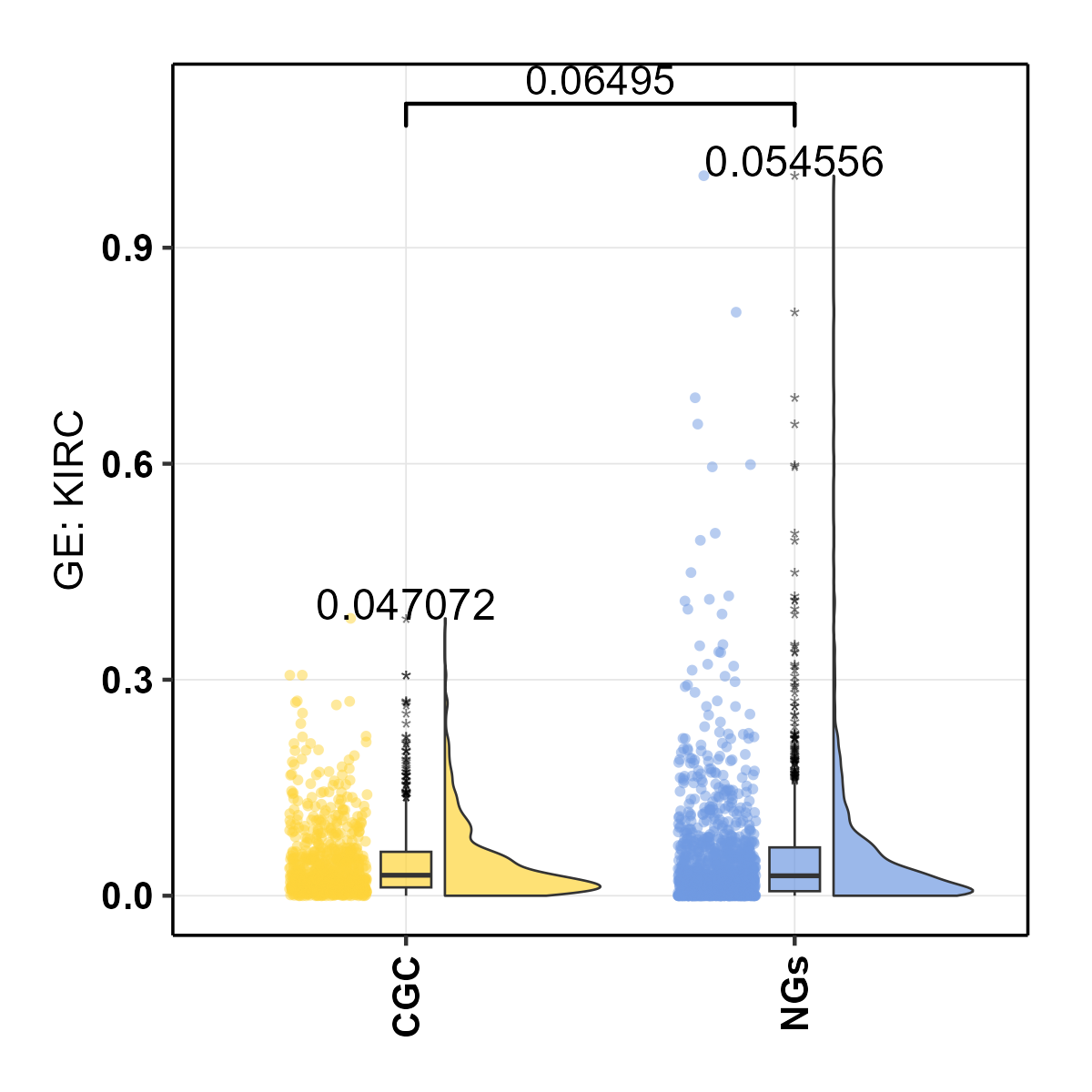

Supplement: Supplementary file 3 [file DataSheet1.ZIP › Supplementary file 5-1/IReflndex_2015/GE_KIRC.png]

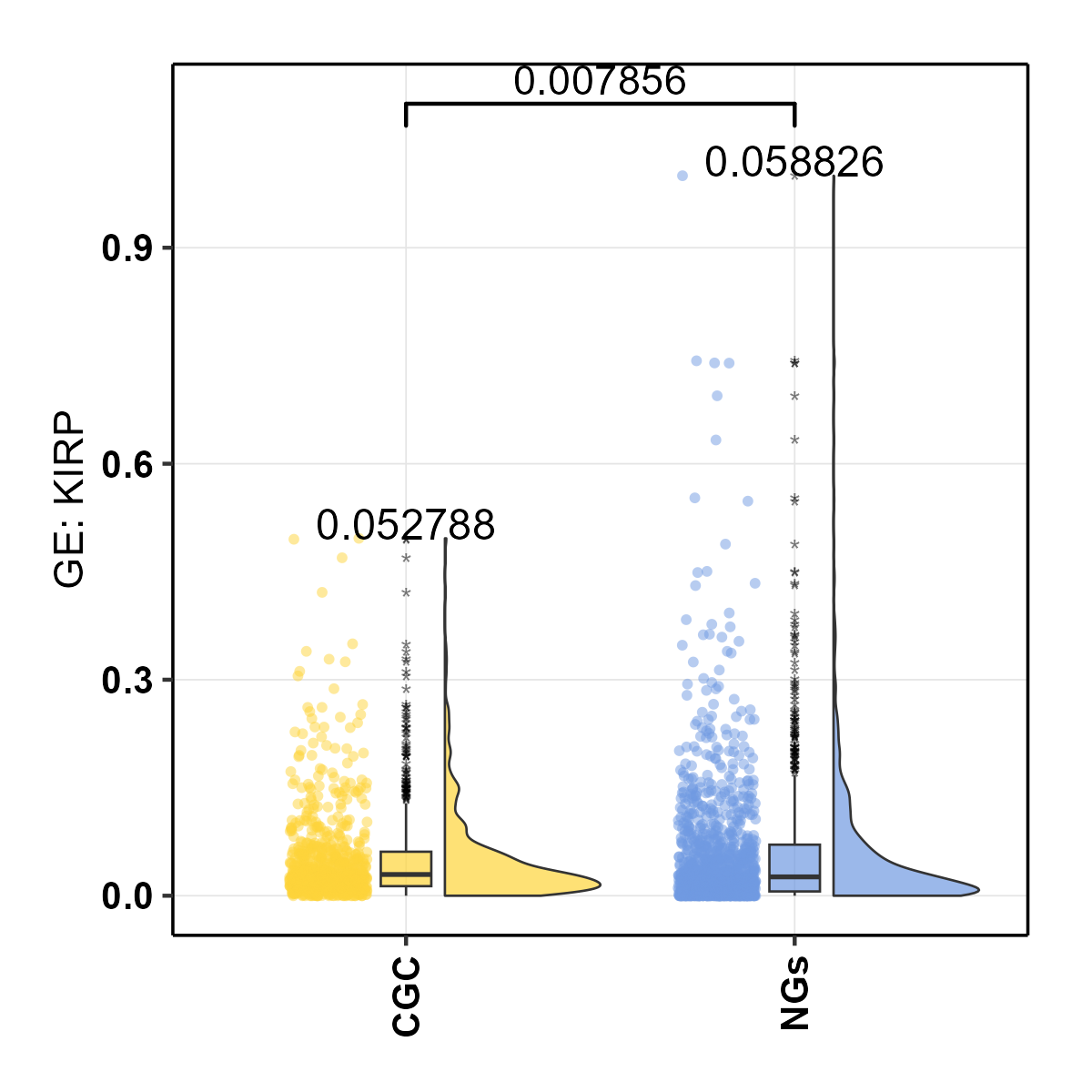

Supplement: Supplementary file 3 [file DataSheet1.ZIP › Supplementary file 5-1/IReflndex_2015/GE_KIRP.png]

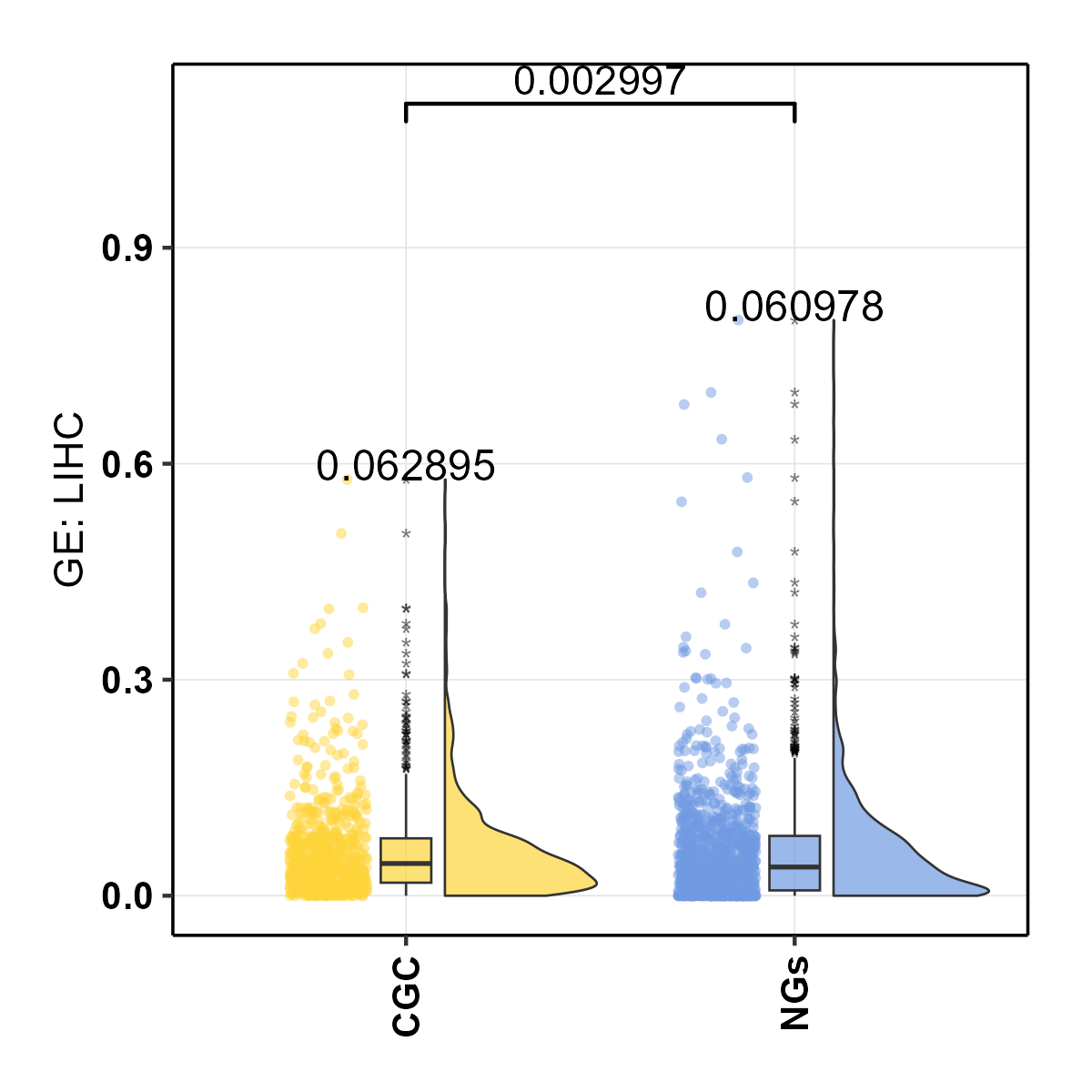

Supplement: Supplementary file 3 [file DataSheet1.ZIP › Supplementary file 5-1/IReflndex_2015/GE_LIHC.png]

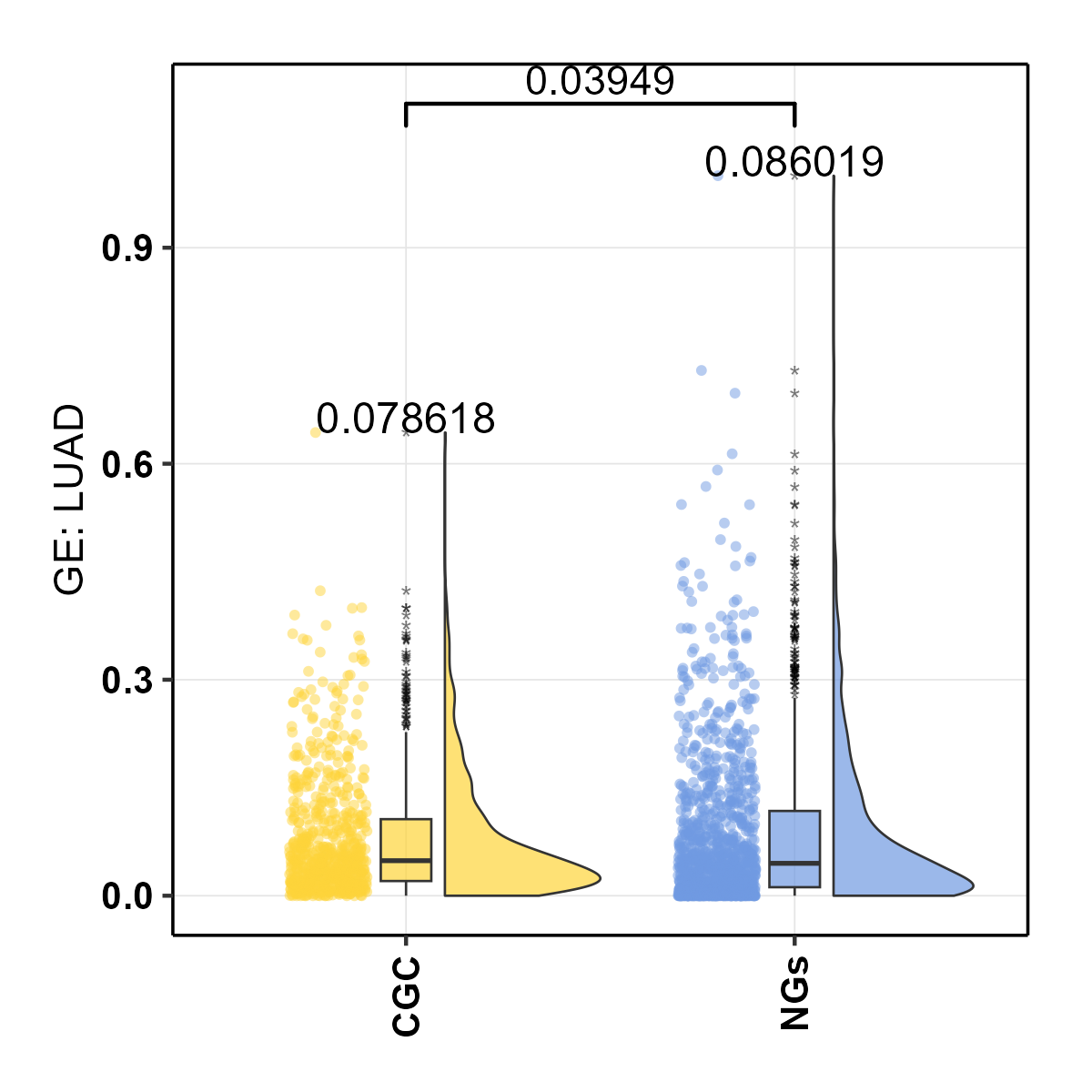

Supplement: Supplementary file 3 [file DataSheet1.ZIP › Supplementary file 5-1/IReflndex_2015/GE_LUAD.png]

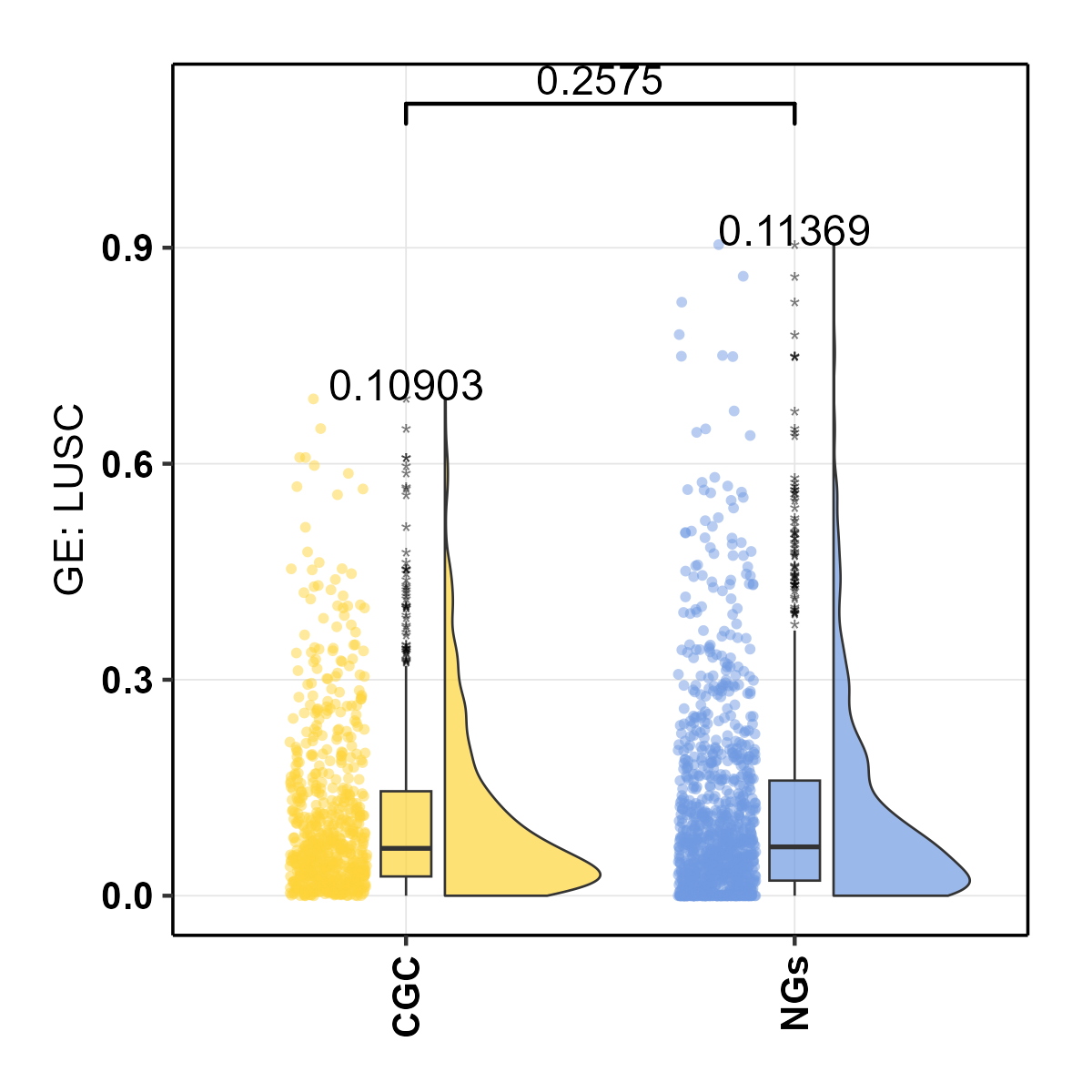

Supplement: Supplementary file 3 [file DataSheet1.ZIP › Supplementary file 5-1/IReflndex_2015/GE_LUSC.png]

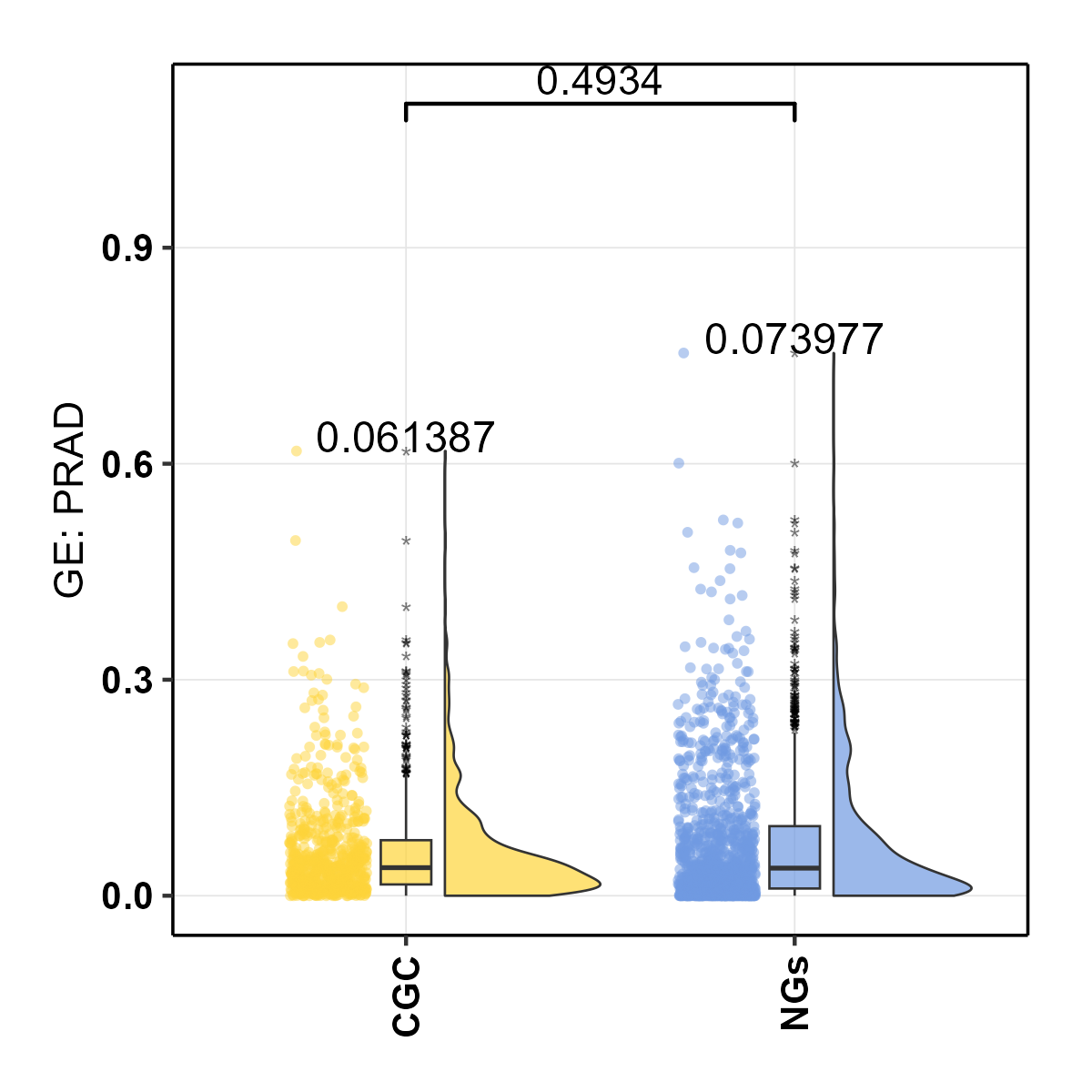

Supplement: Supplementary file 3 [file DataSheet1.ZIP › Supplementary file 5-1/IReflndex_2015/GE_PRAD.png]

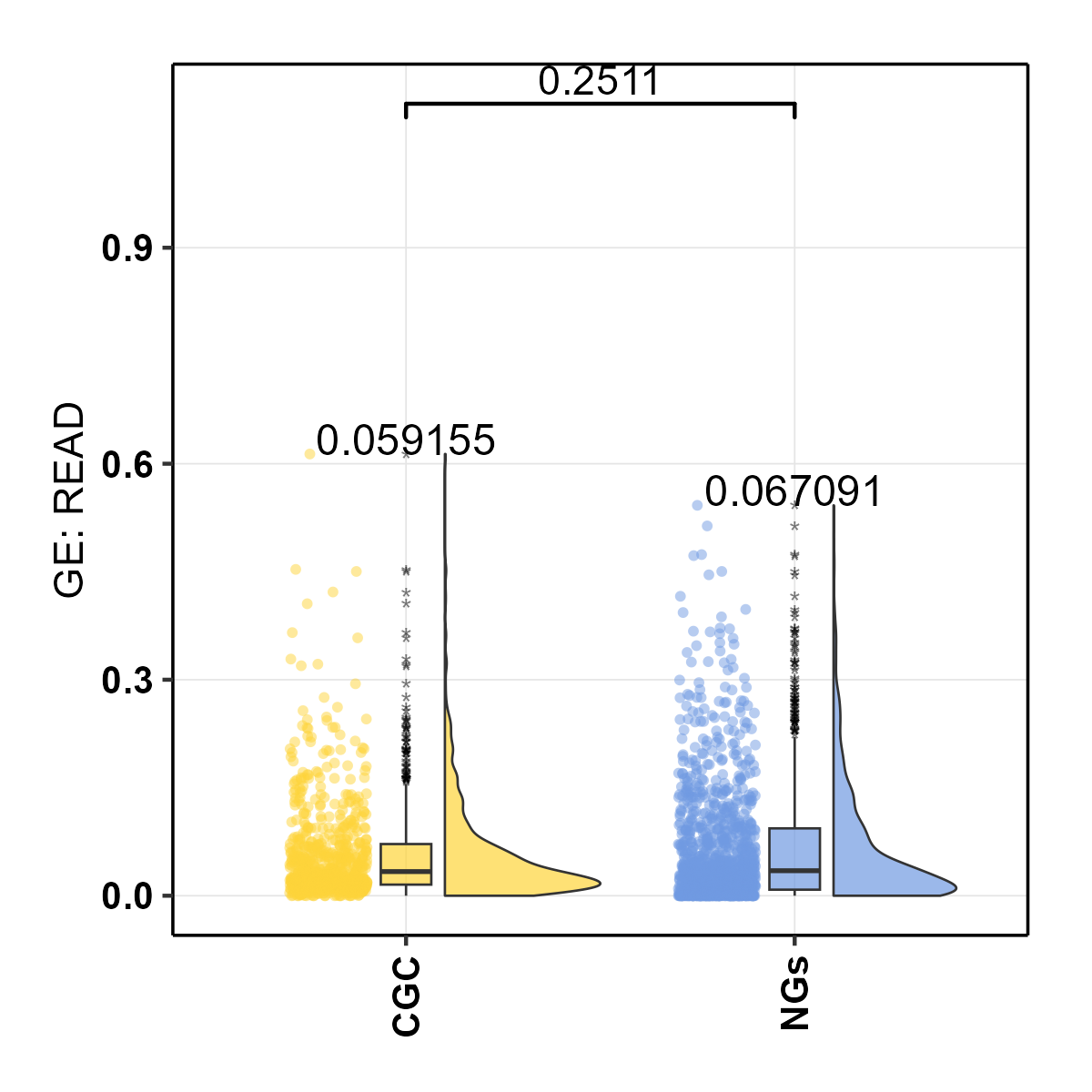

Supplement: Supplementary file 3 [file DataSheet1.ZIP › Supplementary file 5-1/IReflndex_2015/GE_READ.png]

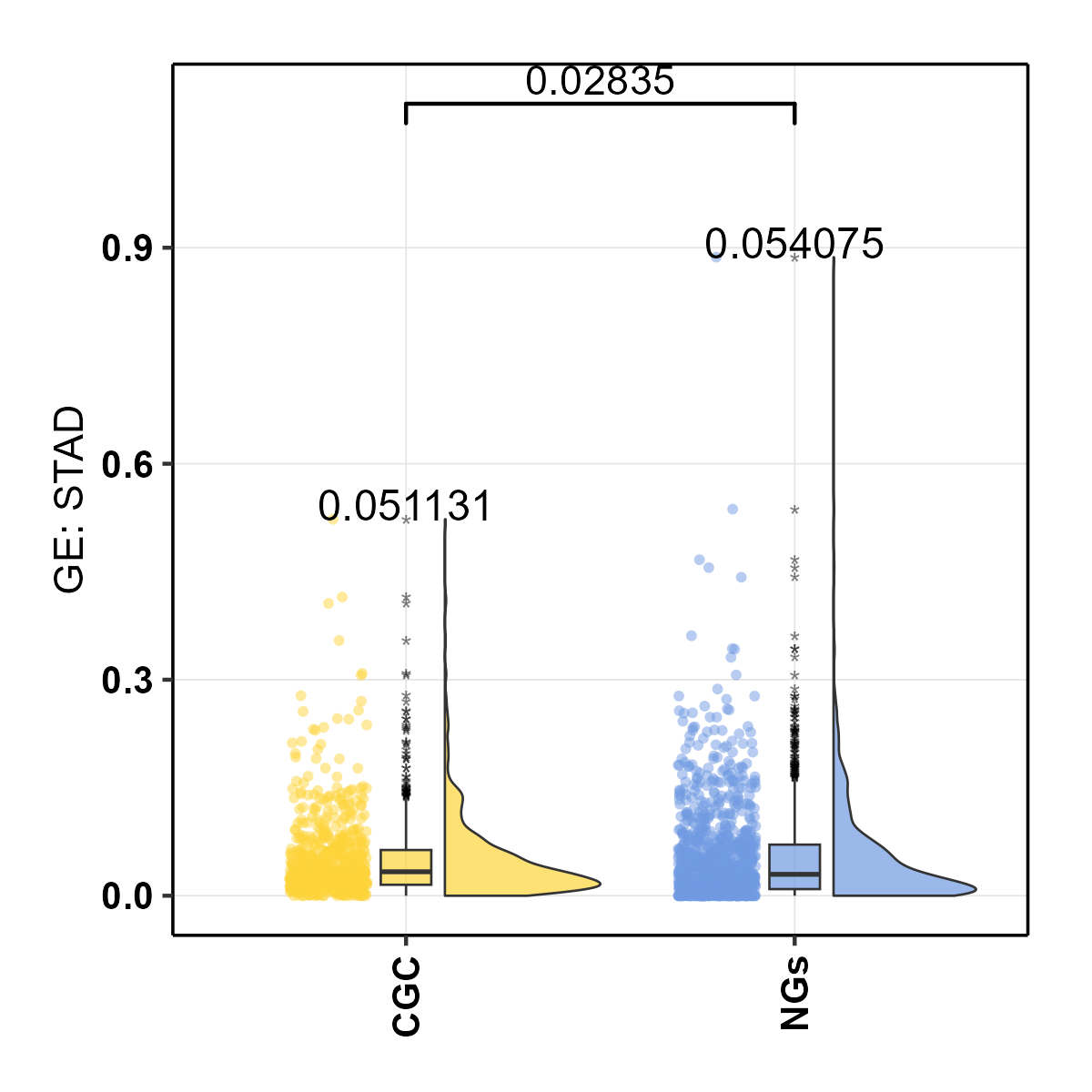

Supplement: Supplementary file 3 [file DataSheet1.ZIP › Supplementary file 5-1/IReflndex_2015/GE_STAD.png]

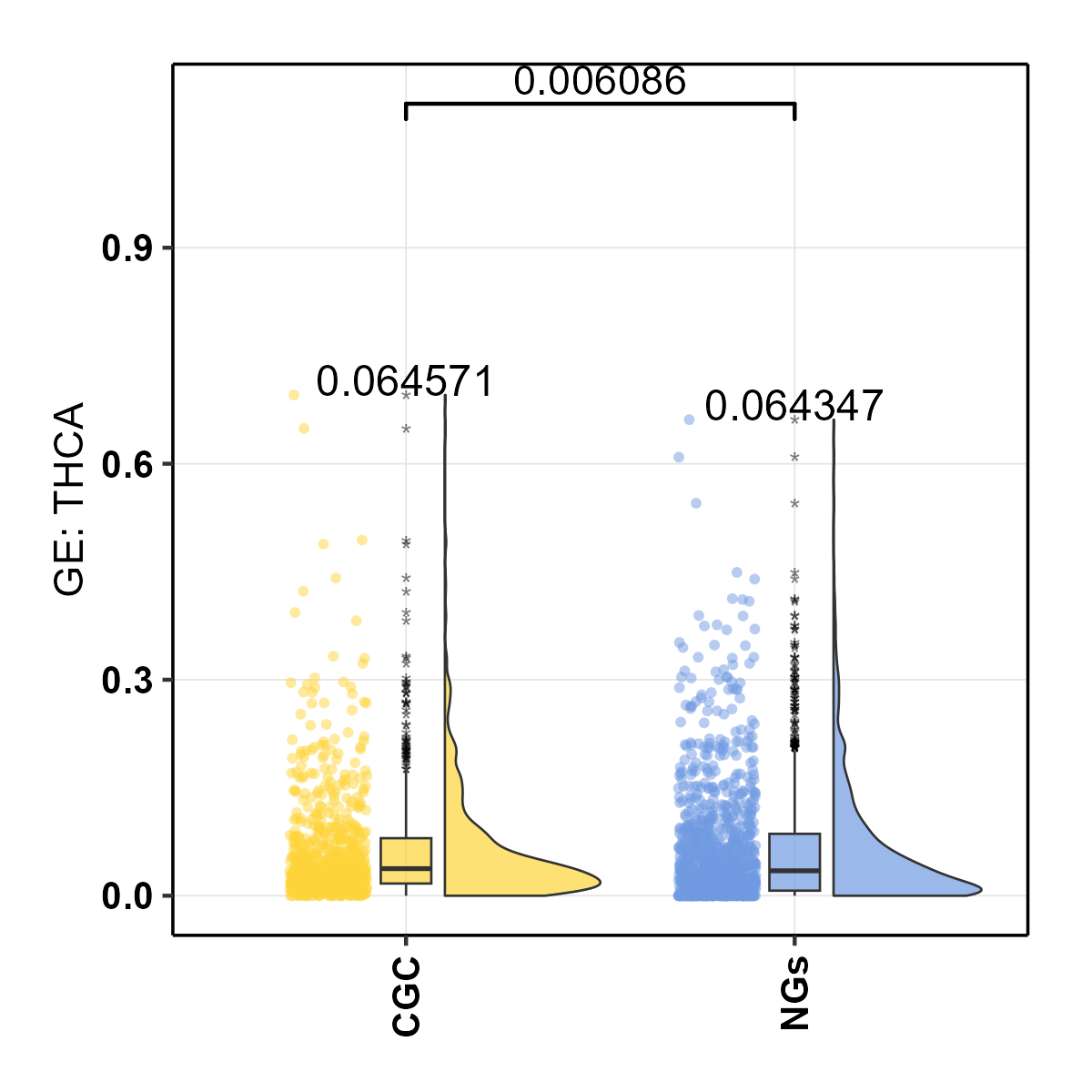

Supplement: Supplementary file 3 [file DataSheet1.ZIP › Supplementary file 5-1/IReflndex_2015/GE_THCA.png]

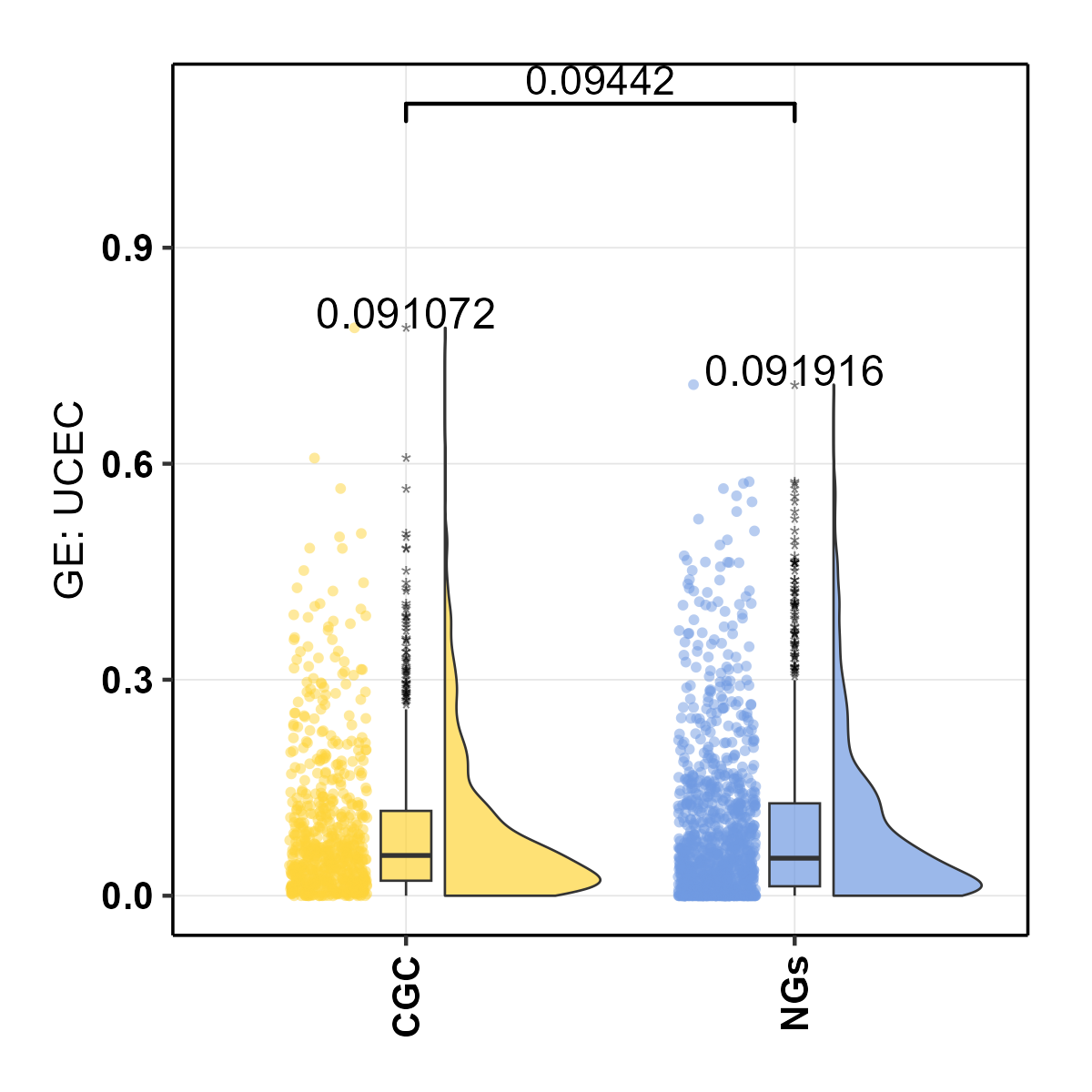

Supplement: Supplementary file 3 [file DataSheet1.ZIP › Supplementary file 5-1/IReflndex_2015/GE_UCEC.png]

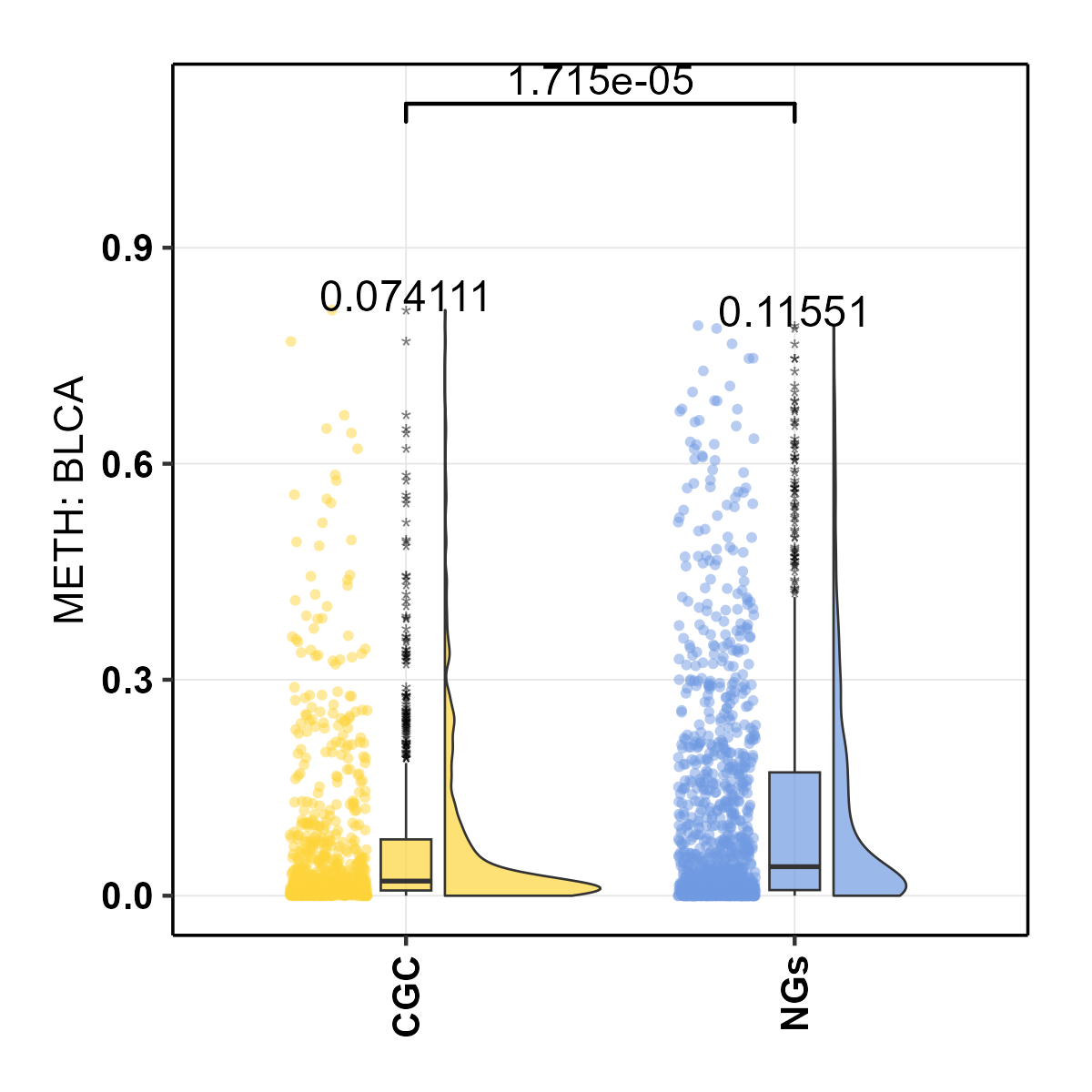

Supplement: Supplementary file 3 [file DataSheet1.ZIP › Supplementary file 5-1/IReflndex_2015/METH_BLCA.png]

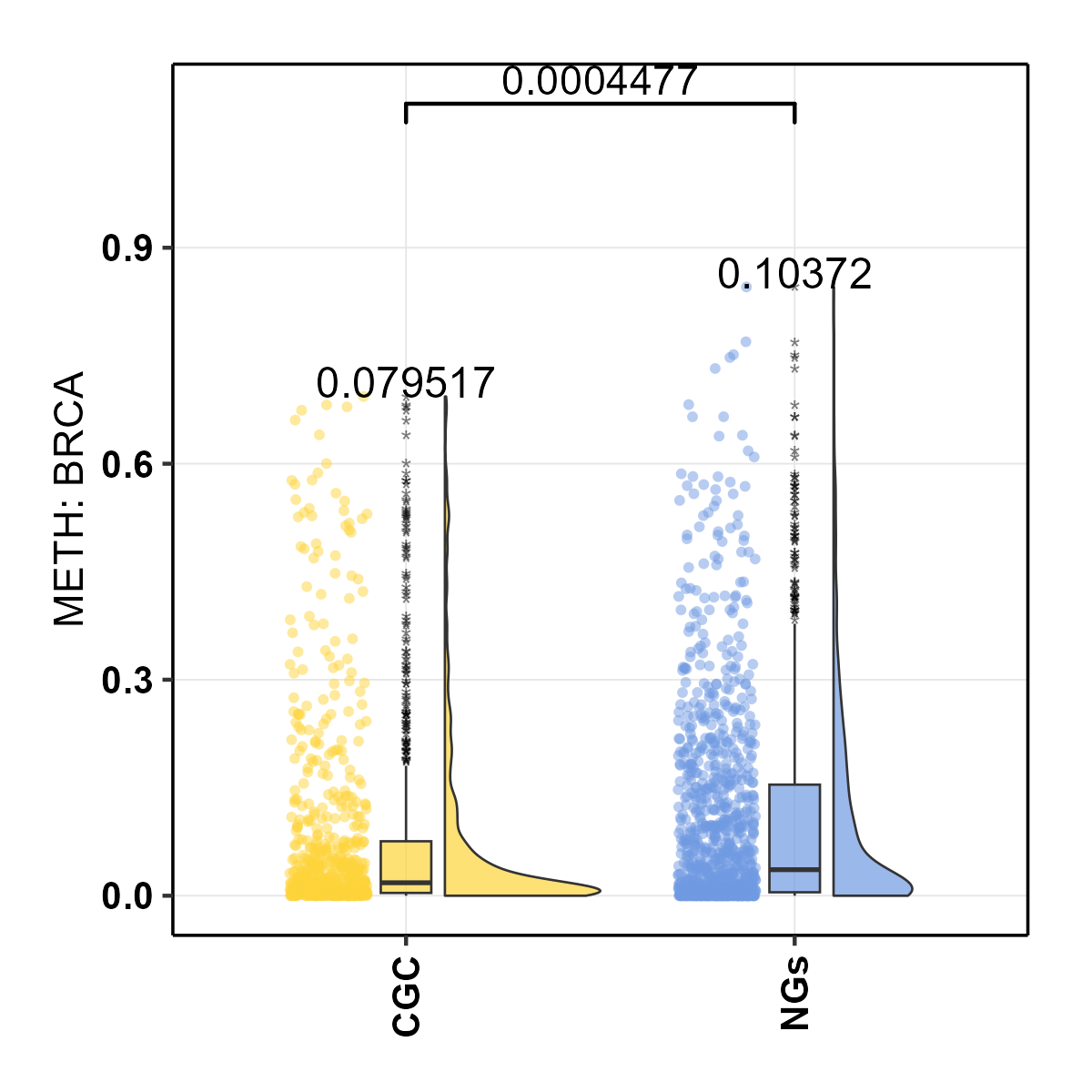

Supplement: Supplementary file 3 [file DataSheet1.ZIP › Supplementary file 5-1/IReflndex_2015/METH_BRCA.png]

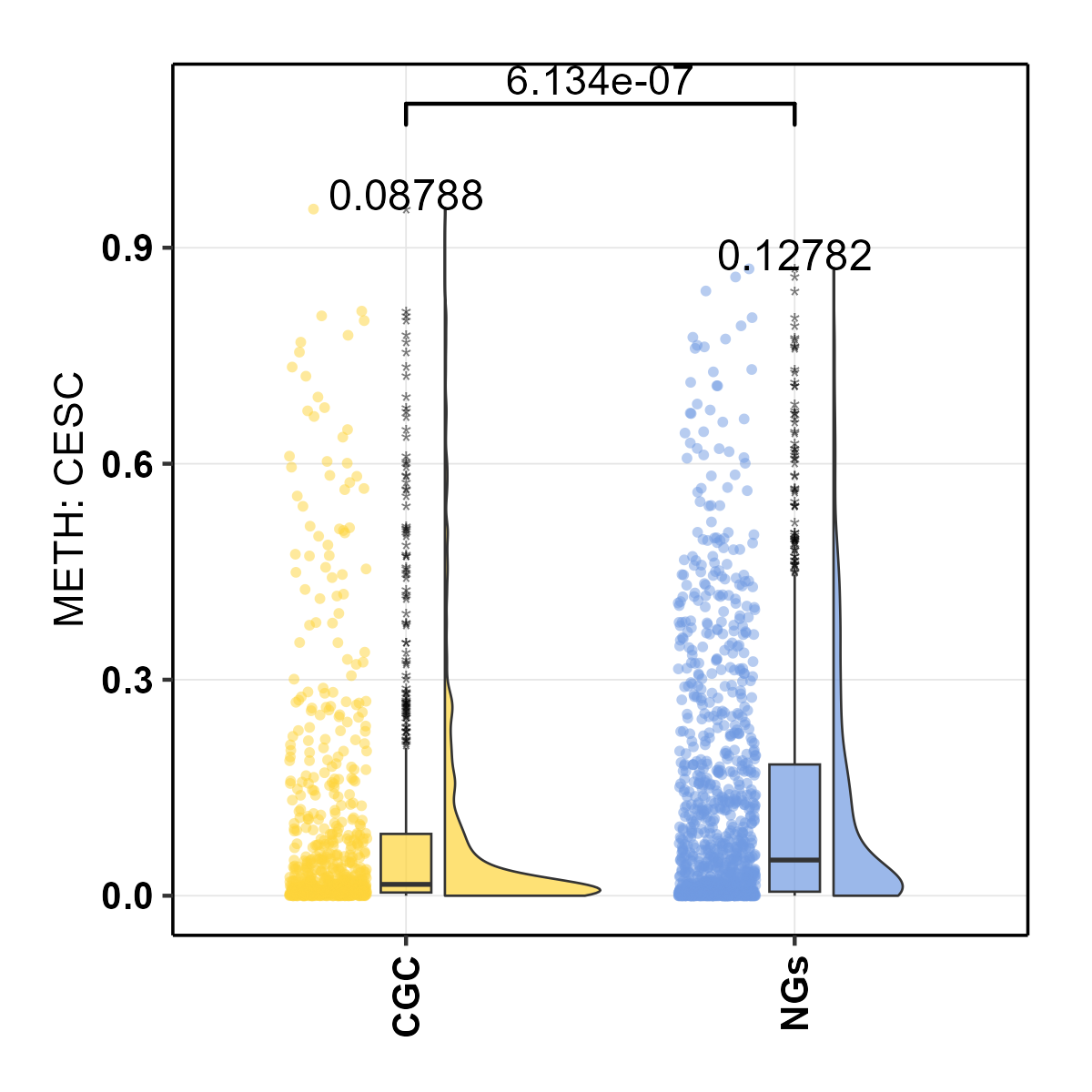

Supplement: Supplementary file 3 [file DataSheet1.ZIP › Supplementary file 5-1/IReflndex_2015/METH_CESC.png]

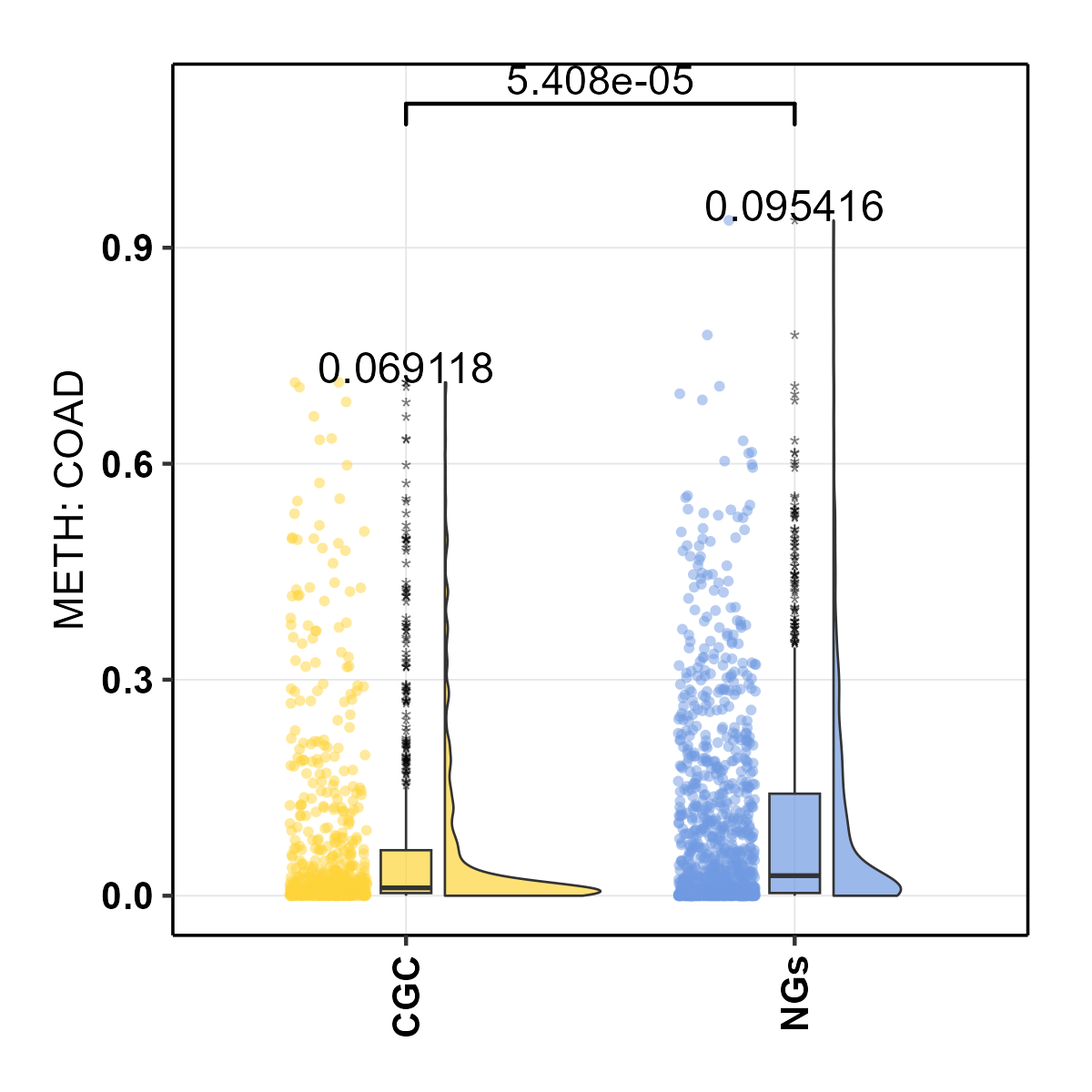

Supplement: Supplementary file 3 [file DataSheet1.ZIP › Supplementary file 5-1/IReflndex_2015/METH_COAD.png]
