# Supplementary figures and images for: MONet: cancer driver gene identification algorithm based on integrated analysis of multi-omics data and network models (part 4 of 4)
Source: Exp Biol Med (Maywood). 2025 Feb 4;250:10399. doi: 10.3389/ebm.2025.10399 (PMC11834253; doi:10.3389/ebm.2025.10399)

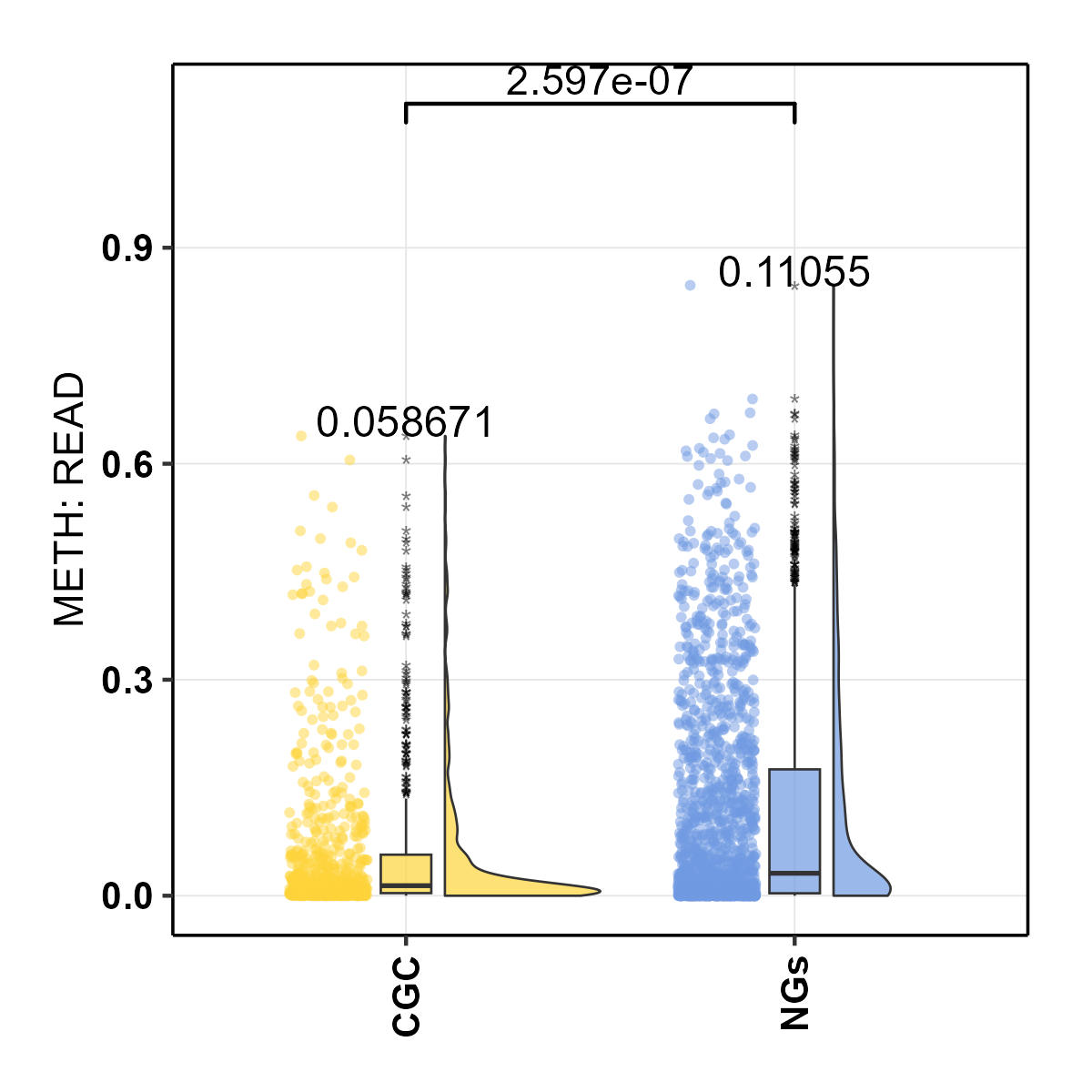

Supplement: Supplementary file 5 [file DataSheet2.ZIP › Supplementary file 5-2/STRINGdb/METH_READ.png]

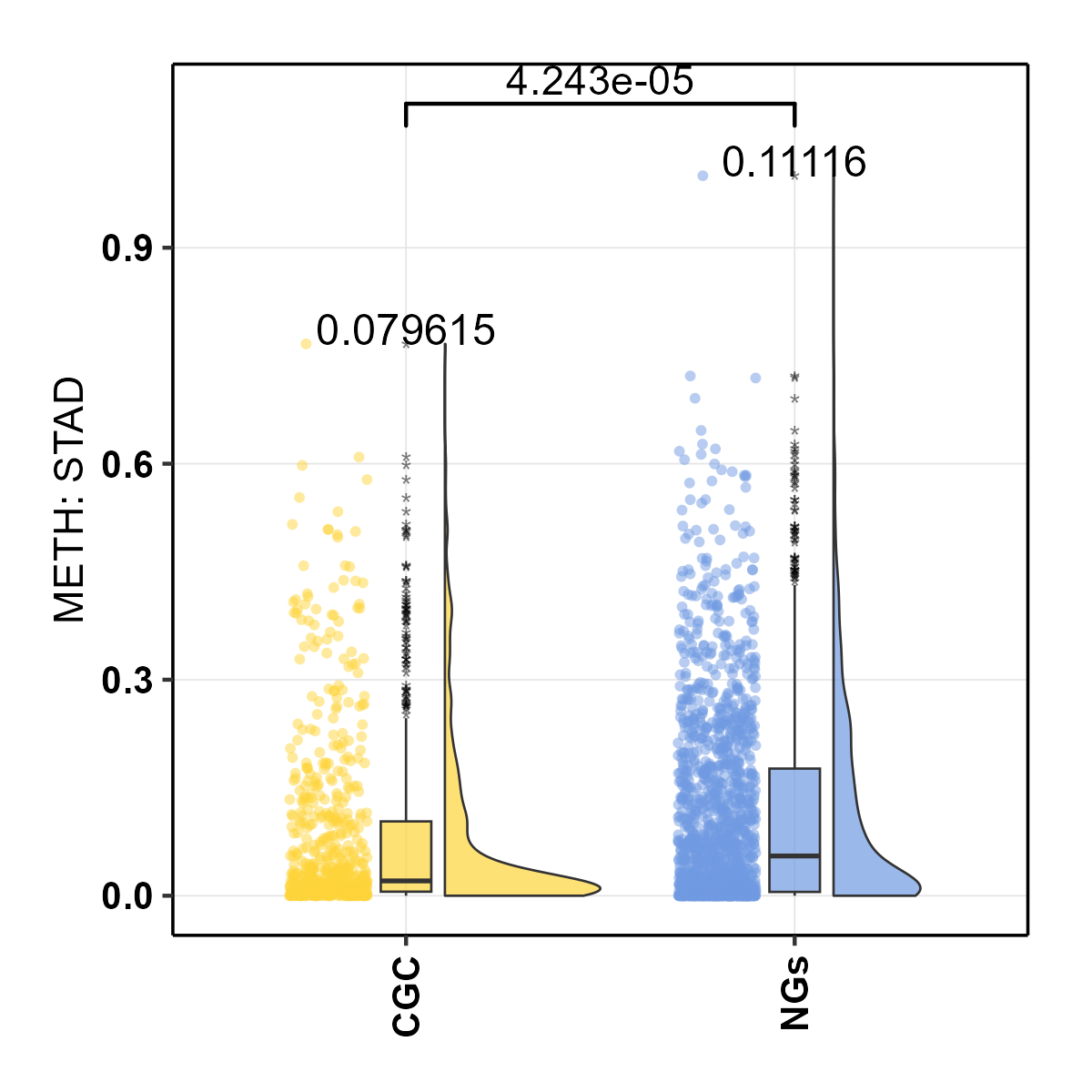

Supplement: Supplementary file 5 [file DataSheet2.ZIP › Supplementary file 5-2/STRINGdb/METH_STAD.png]

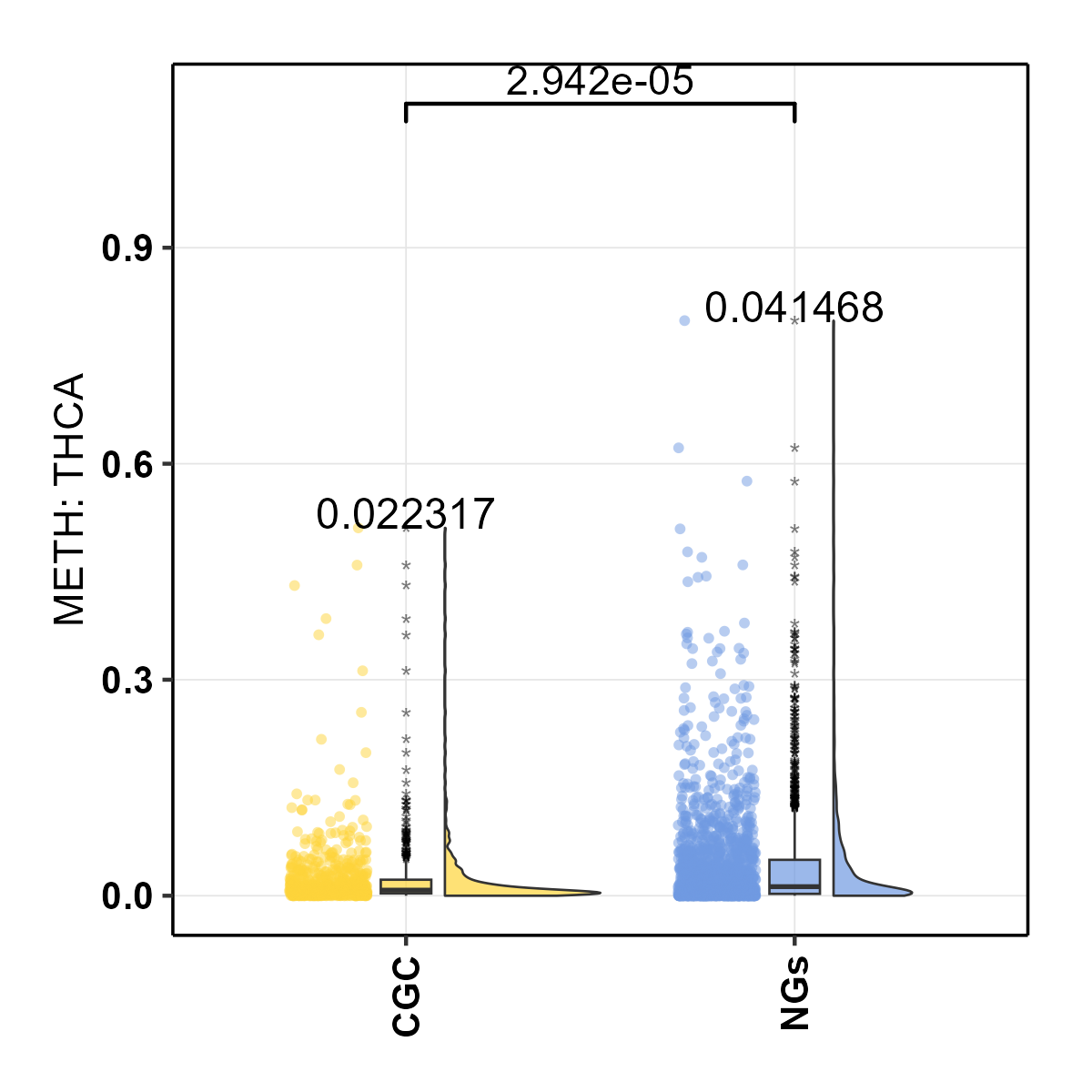

Supplement: Supplementary file 5 [file DataSheet2.ZIP › Supplementary file 5-2/STRINGdb/METH_THCA.png]

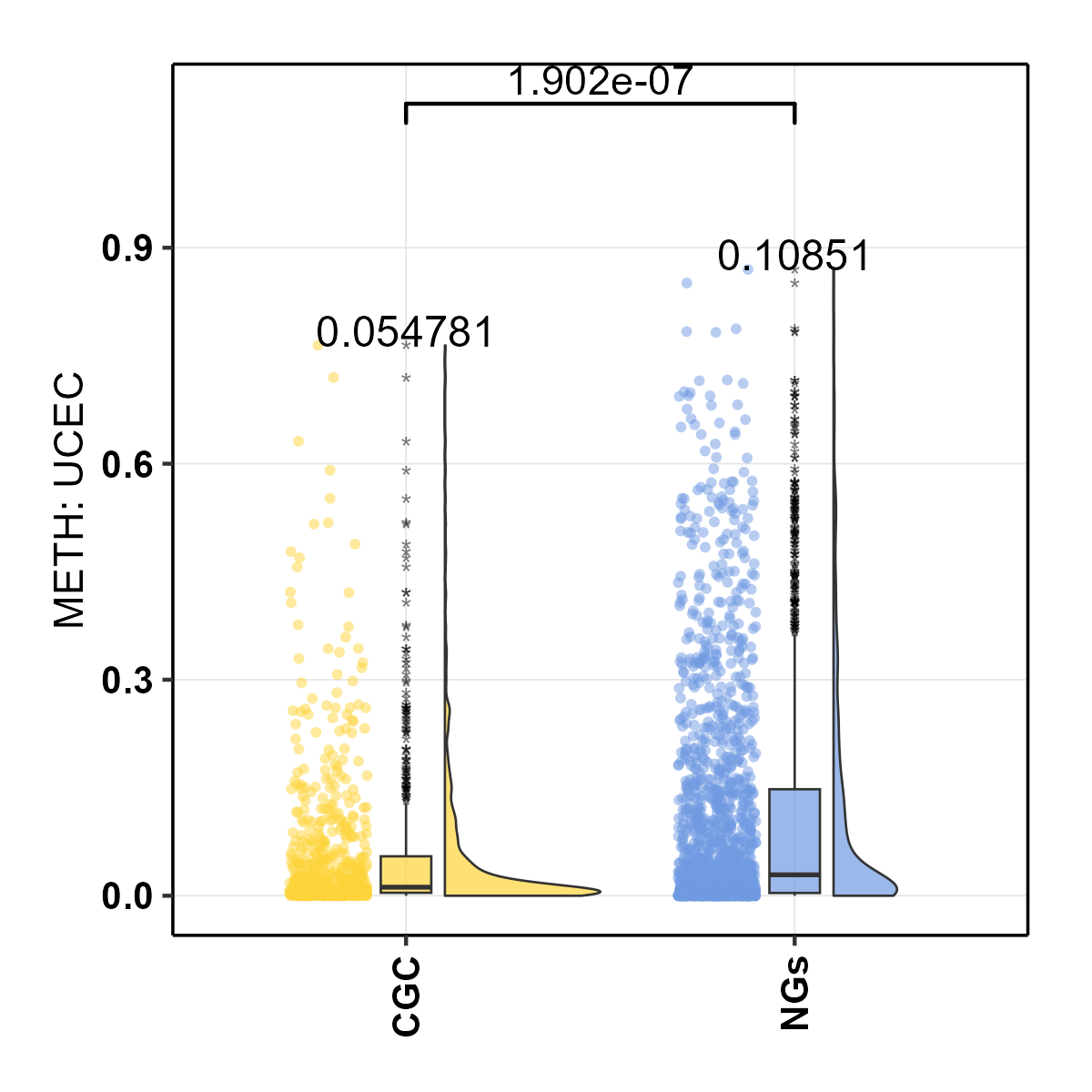

Supplement: Supplementary file 5 [file DataSheet2.ZIP › Supplementary file 5-2/STRINGdb/METH_UCEC.png]

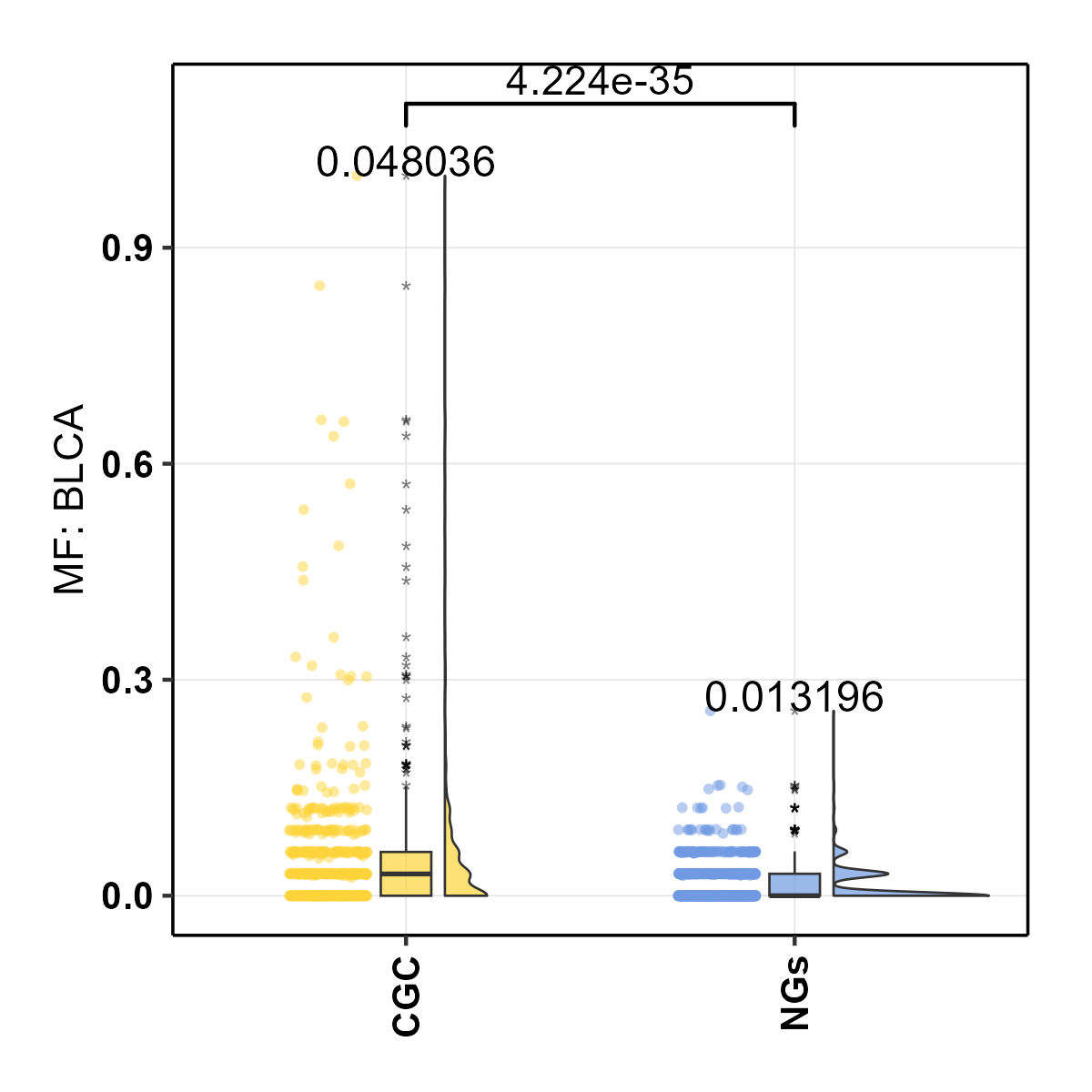

Supplement: Supplementary file 5 [file DataSheet2.ZIP › Supplementary file 5-2/STRINGdb/MF_BLCA.png]

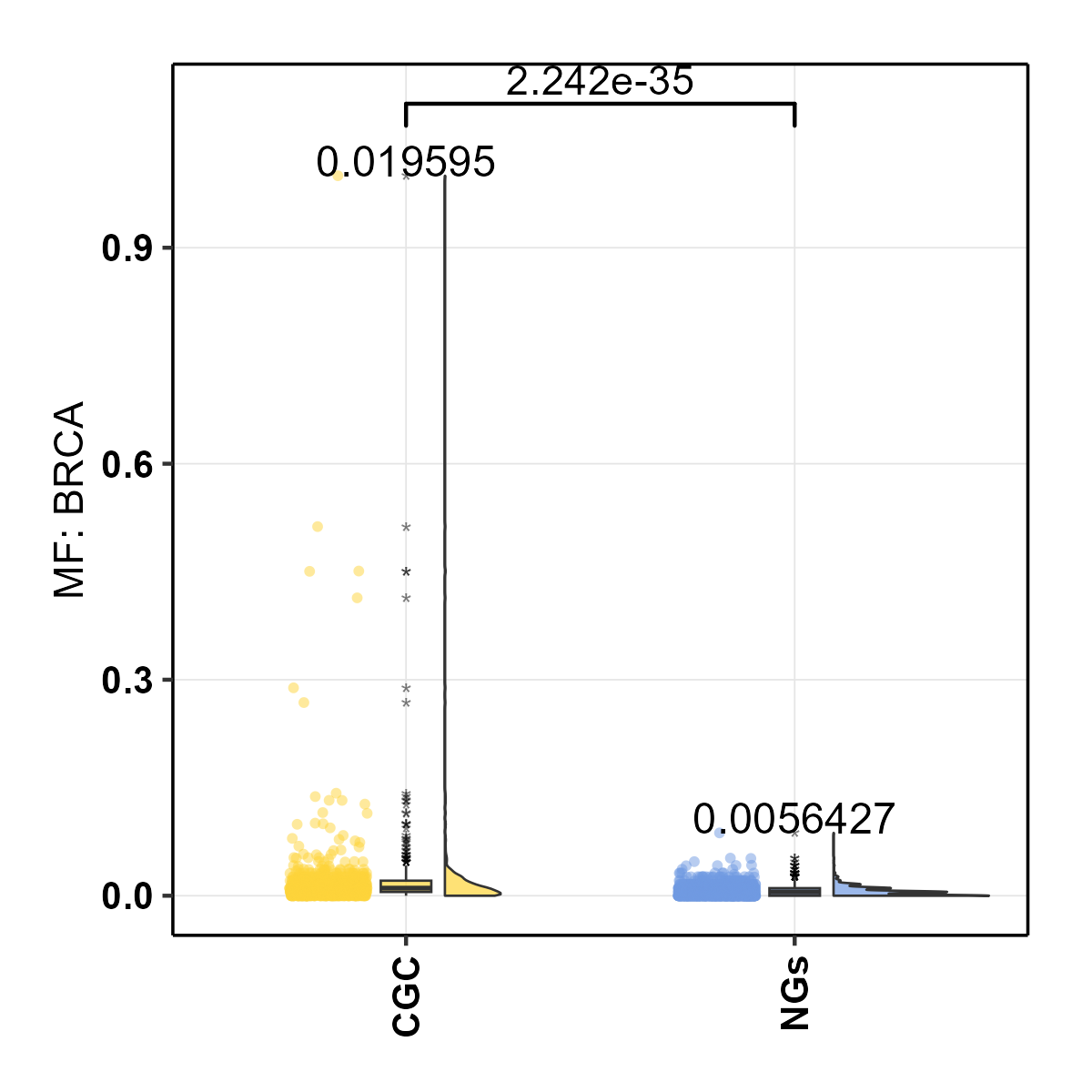

Supplement: Supplementary file 5 [file DataSheet2.ZIP › Supplementary file 5-2/STRINGdb/MF_BRCA.png]

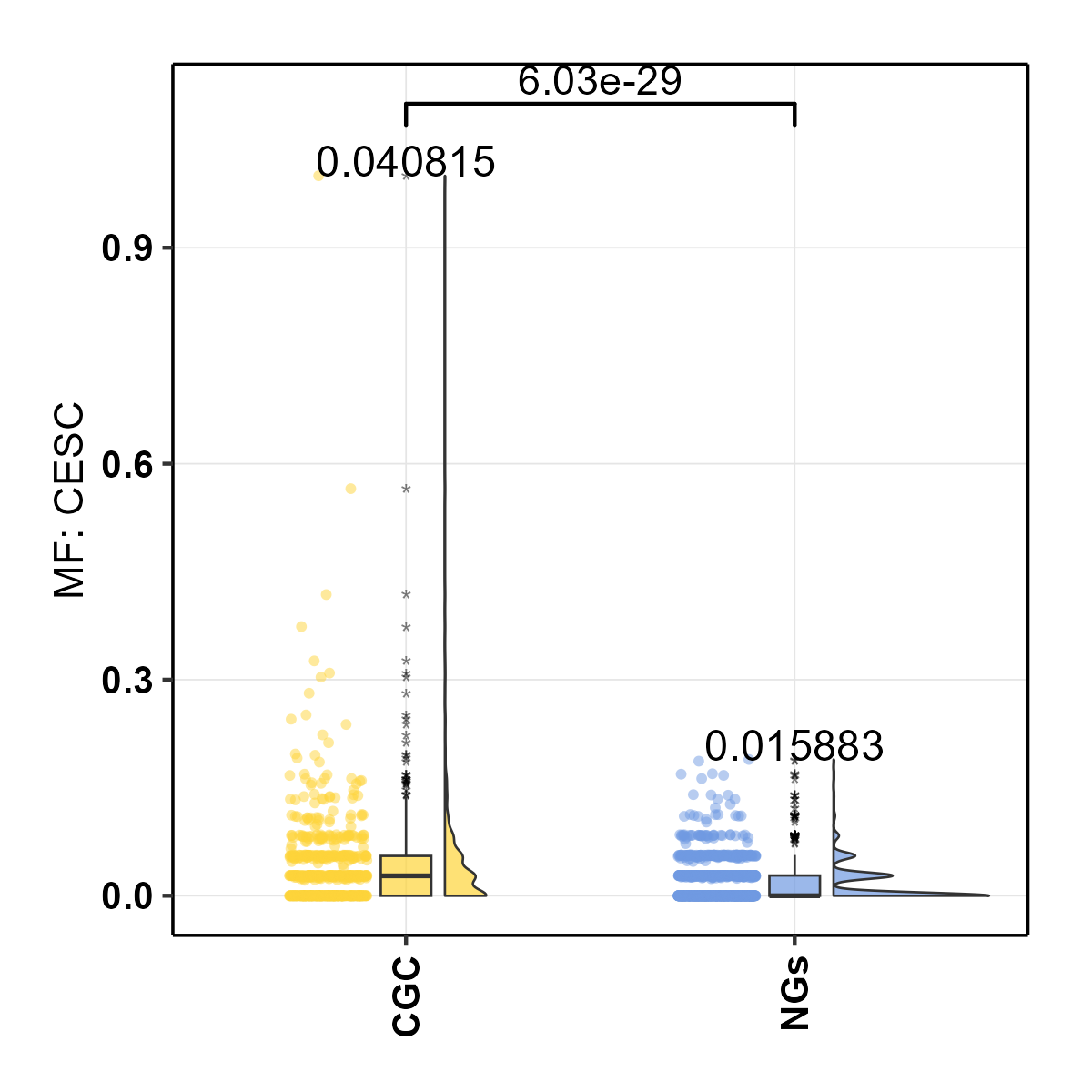

Supplement: Supplementary file 5 [file DataSheet2.ZIP › Supplementary file 5-2/STRINGdb/MF_CESC.png]

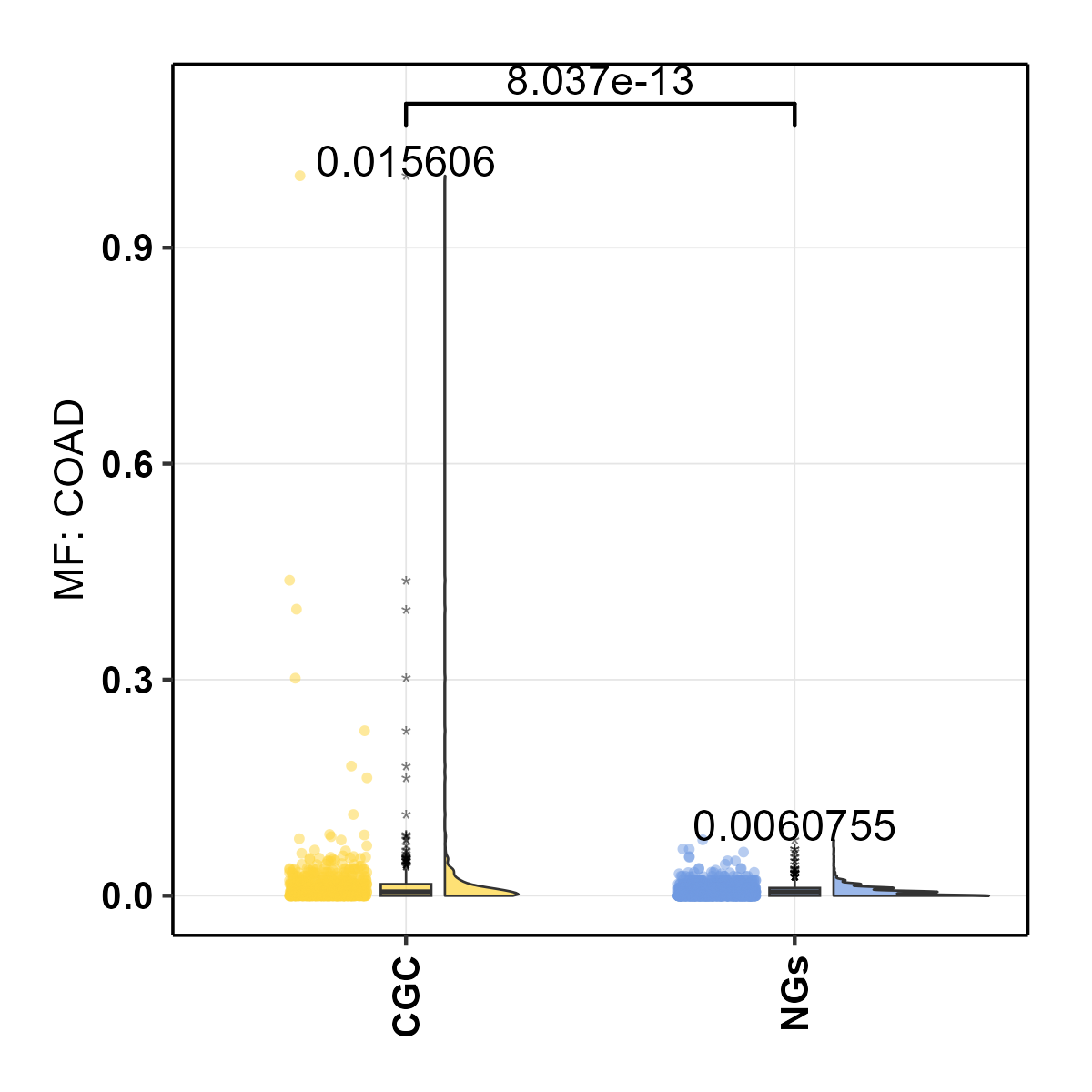

Supplement: Supplementary file 5 [file DataSheet2.ZIP › Supplementary file 5-2/STRINGdb/MF_COAD.png]

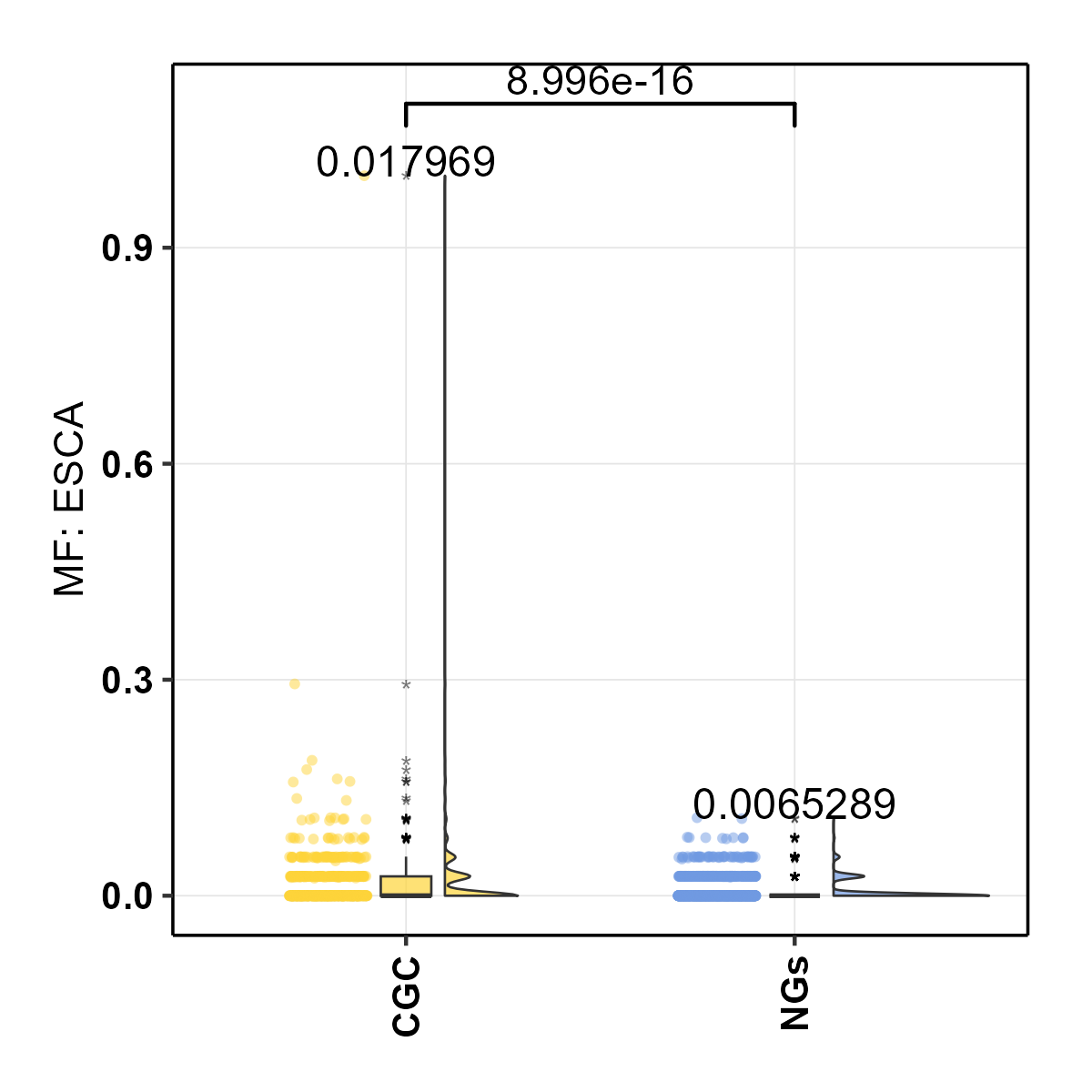

Supplement: Supplementary file 5 [file DataSheet2.ZIP › Supplementary file 5-2/STRINGdb/MF_ESCA.png]

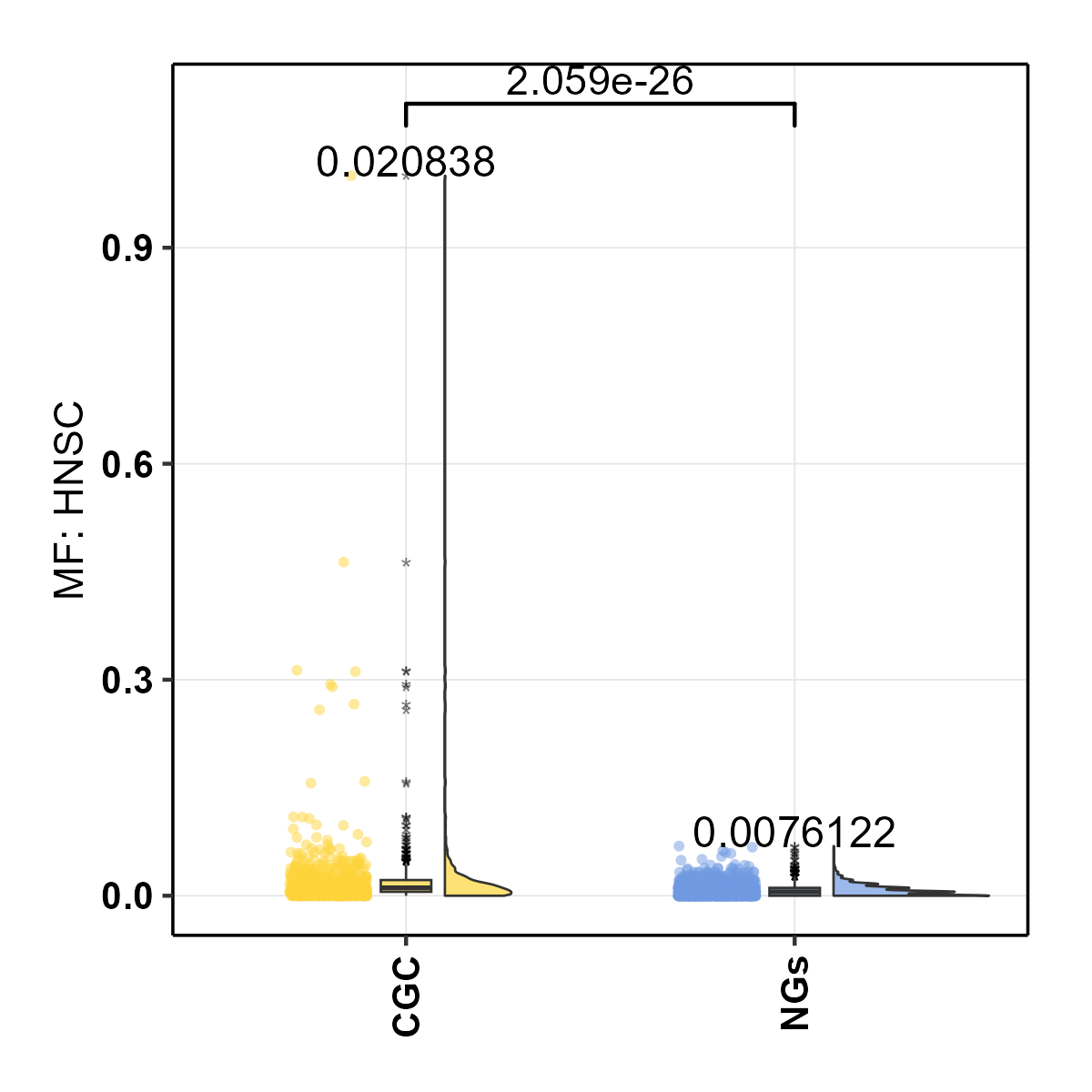

Supplement: Supplementary file 5 [file DataSheet2.ZIP › Supplementary file 5-2/STRINGdb/MF_HNSC.png]

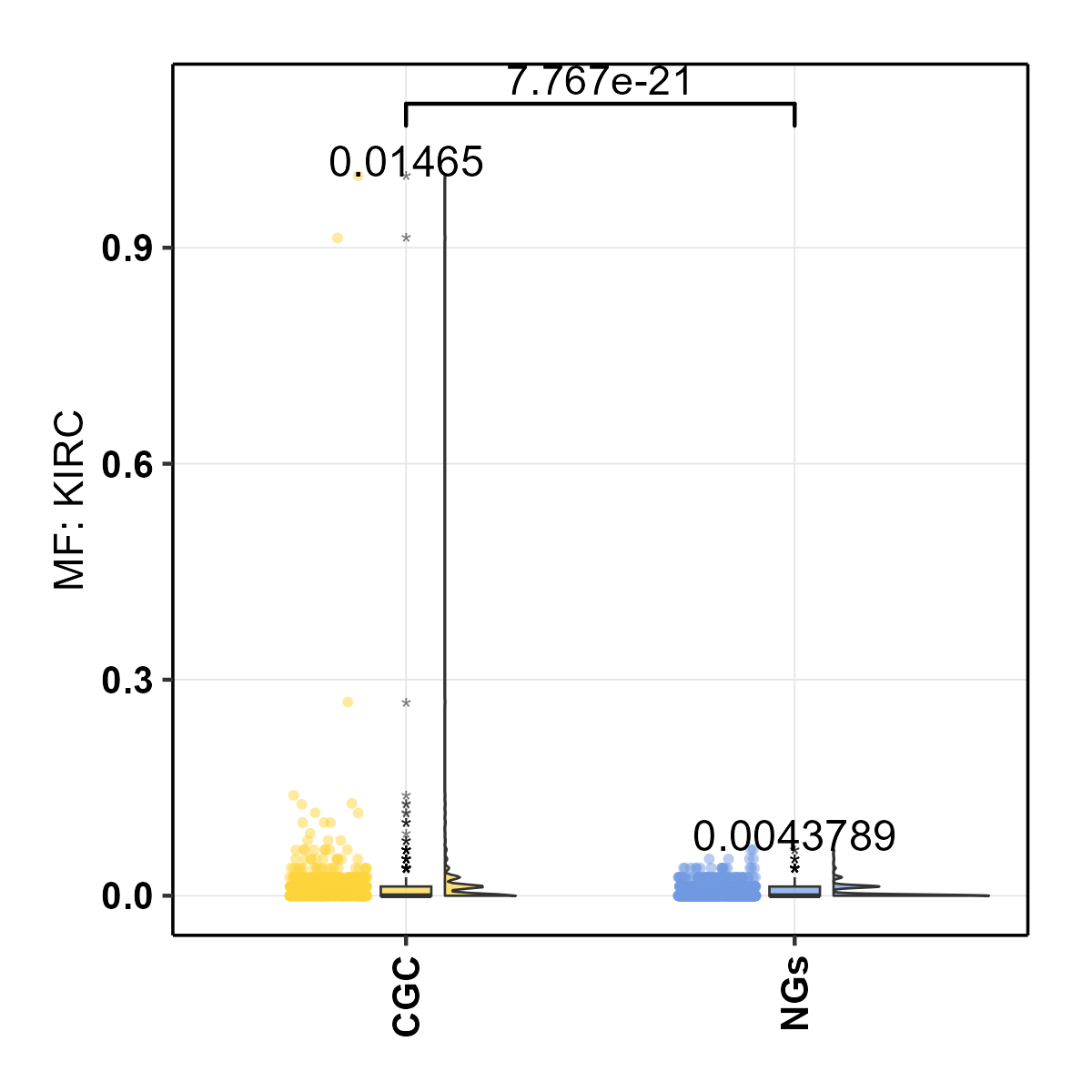

Supplement: Supplementary file 5 [file DataSheet2.ZIP › Supplementary file 5-2/STRINGdb/MF_KIRC.png]

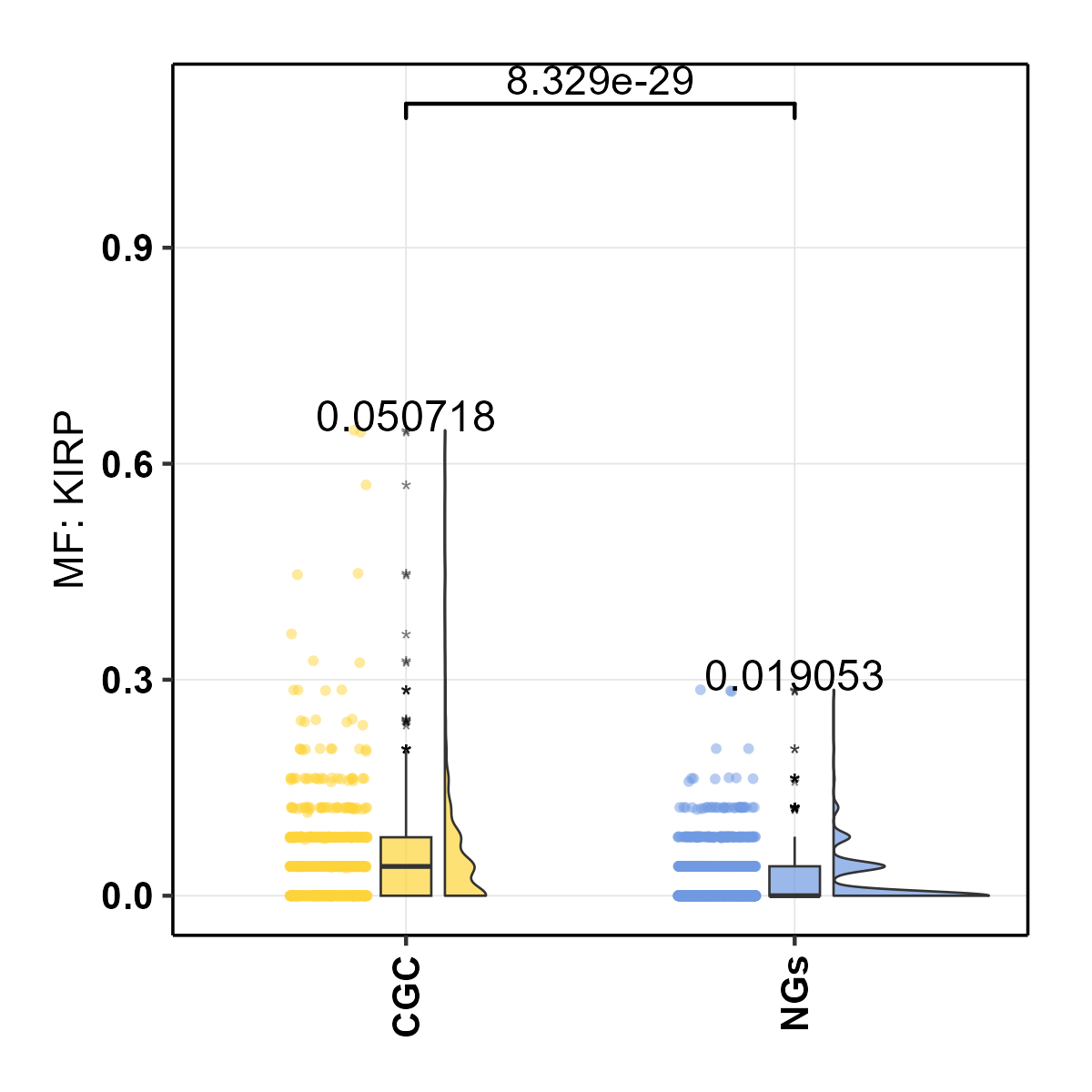

Supplement: Supplementary file 5 [file DataSheet2.ZIP › Supplementary file 5-2/STRINGdb/MF_KIRP.png]

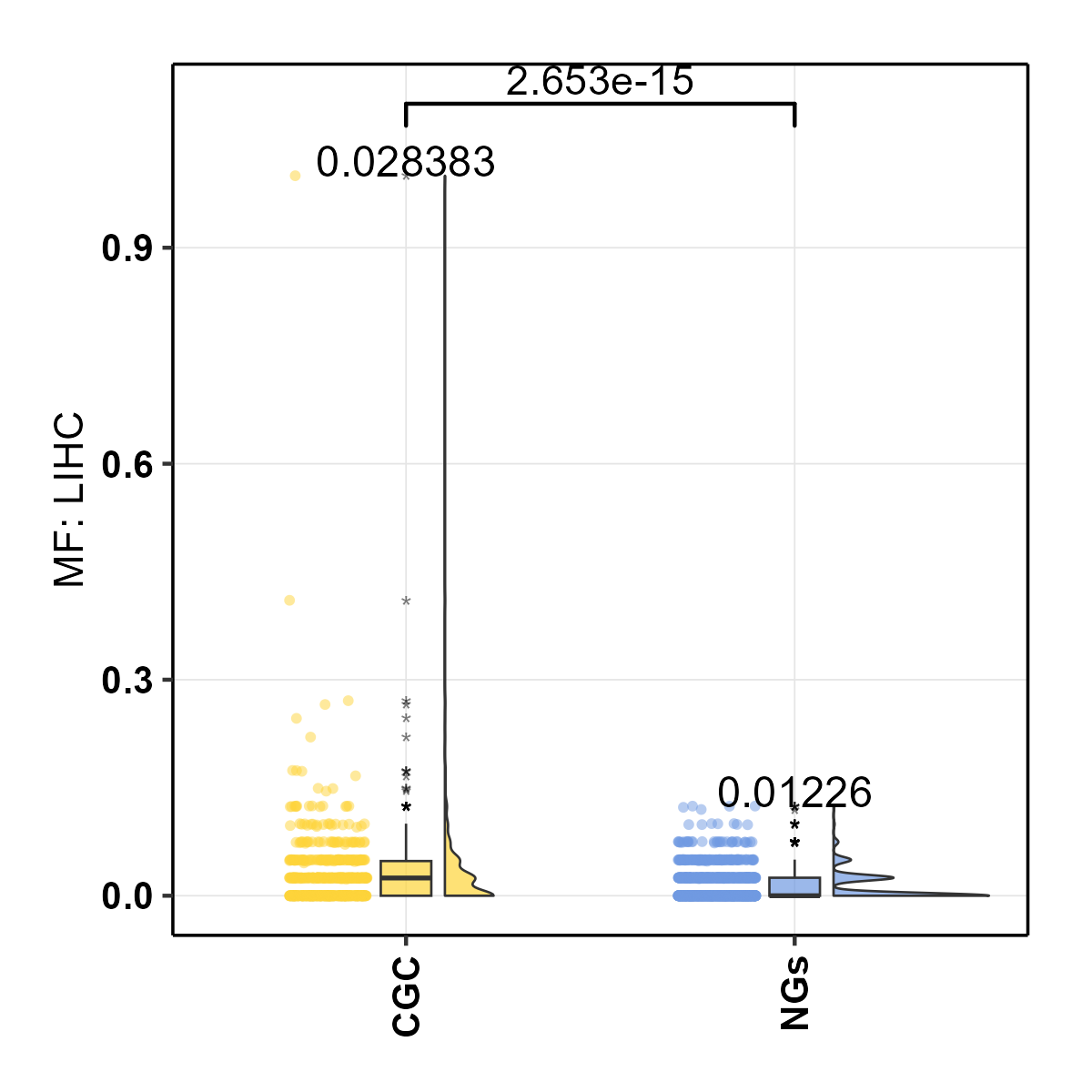

Supplement: Supplementary file 5 [file DataSheet2.ZIP › Supplementary file 5-2/STRINGdb/MF_LIHC.png]

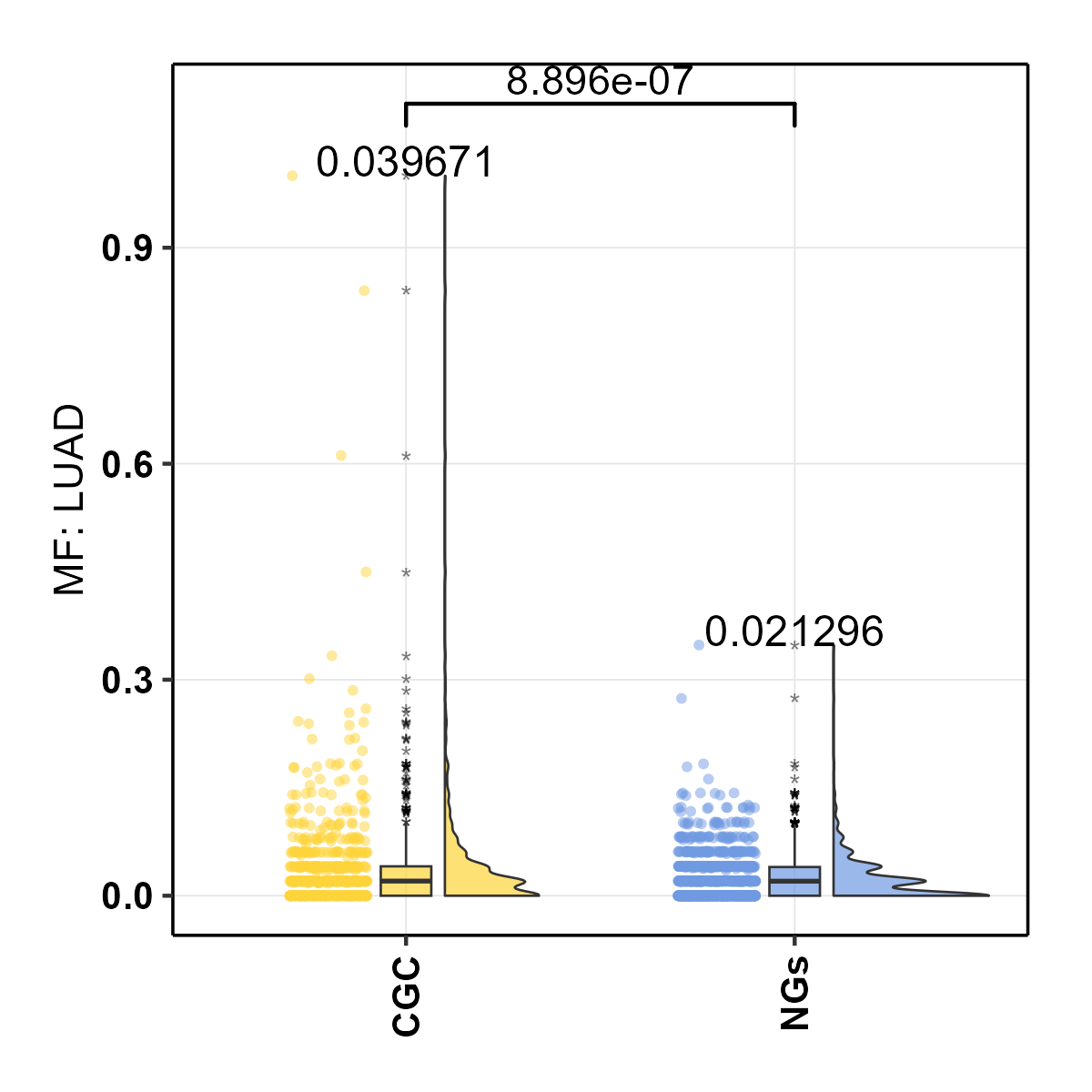

Supplement: Supplementary file 5 [file DataSheet2.ZIP › Supplementary file 5-2/STRINGdb/MF_LUAD.png]

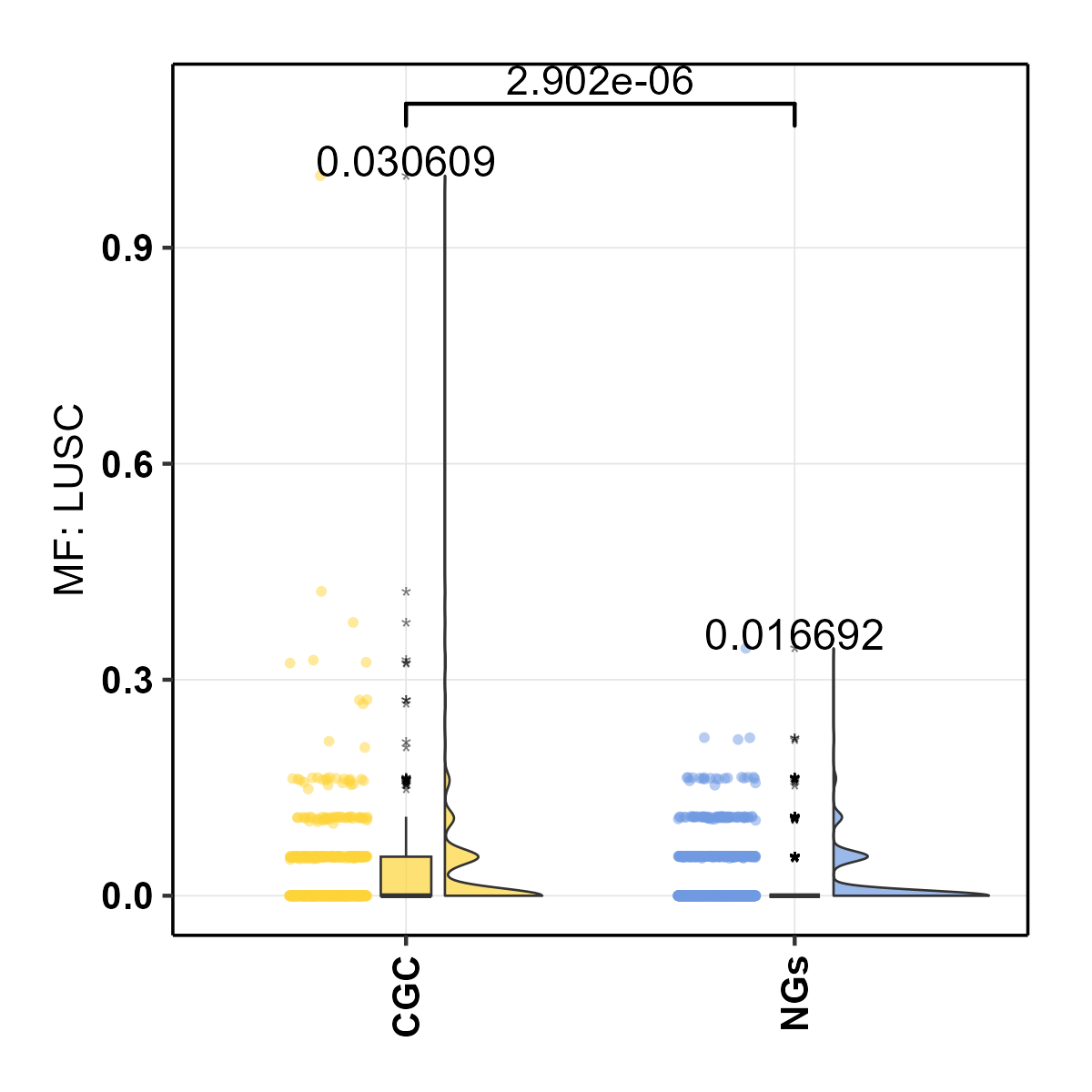

Supplement: Supplementary file 5 [file DataSheet2.ZIP › Supplementary file 5-2/STRINGdb/MF_LUSC.png]

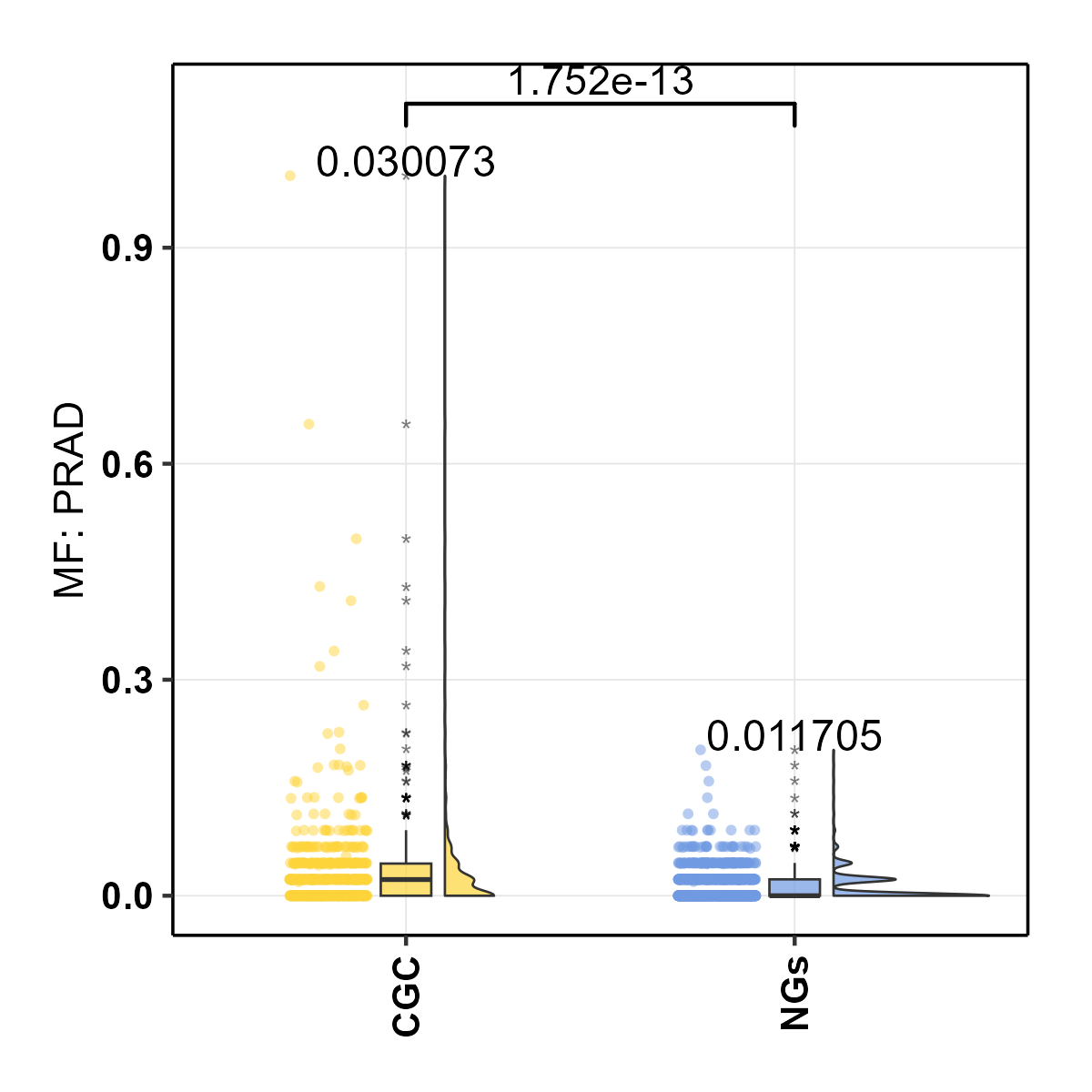

Supplement: Supplementary file 5 [file DataSheet2.ZIP › Supplementary file 5-2/STRINGdb/MF_PRAD.png]

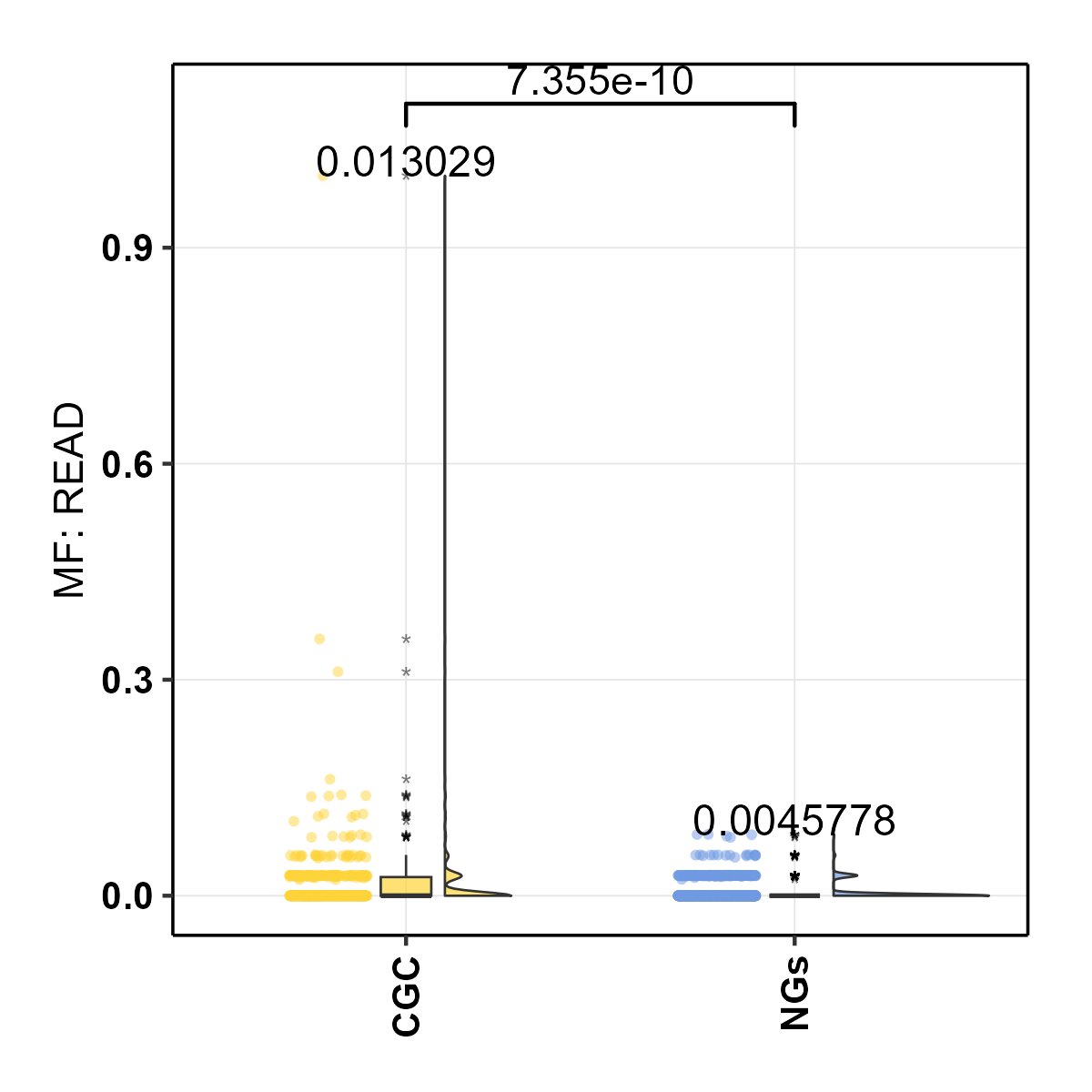

Supplement: Supplementary file 5 [file DataSheet2.ZIP › Supplementary file 5-2/STRINGdb/MF_READ.png]

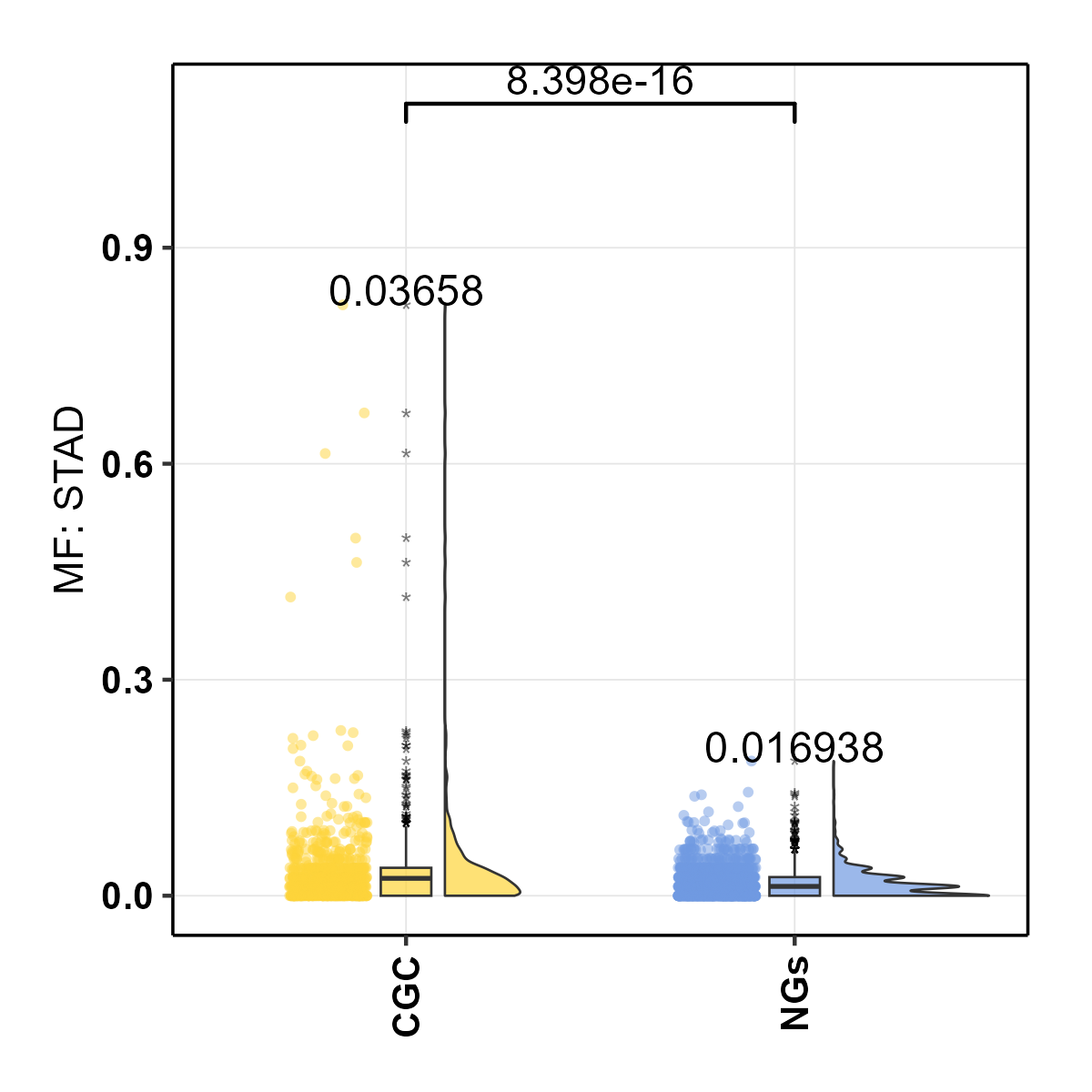

Supplement: Supplementary file 5 [file DataSheet2.ZIP › Supplementary file 5-2/STRINGdb/MF_STAD.png]

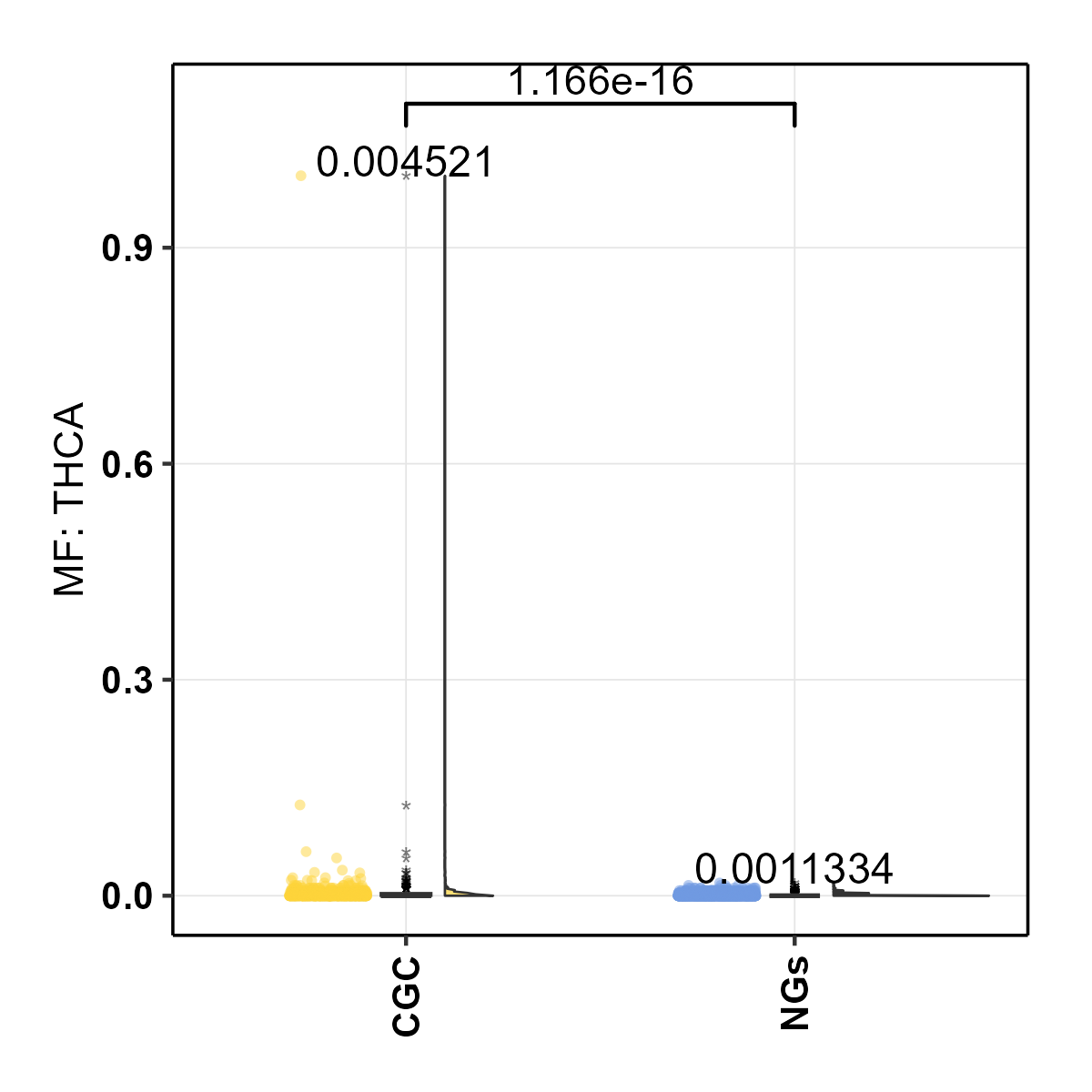

Supplement: Supplementary file 5 [file DataSheet2.ZIP › Supplementary file 5-2/STRINGdb/MF_THCA.png]

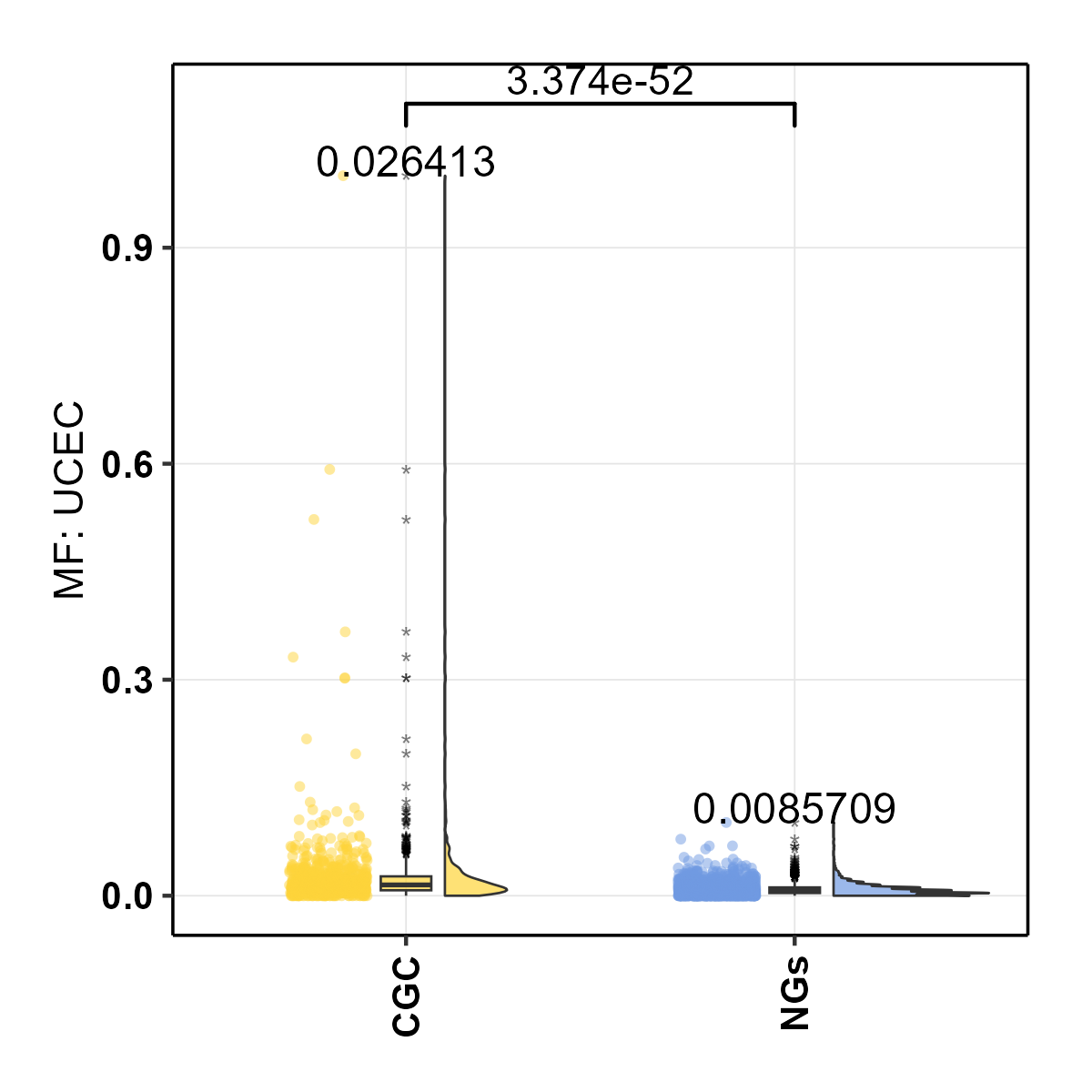

Supplement: Supplementary file 5 [file DataSheet2.ZIP › Supplementary file 5-2/STRINGdb/MF_UCEC.png]
