# Supplementary figures and images for: MONet: cancer driver gene identification algorithm based on integrated analysis of multi-omics data and network models (part 3 of 4)
Source: Exp Biol Med (Maywood). 2025 Feb 4;250:10399. doi: 10.3389/ebm.2025.10399 (PMC11834253; doi:10.3389/ebm.2025.10399)

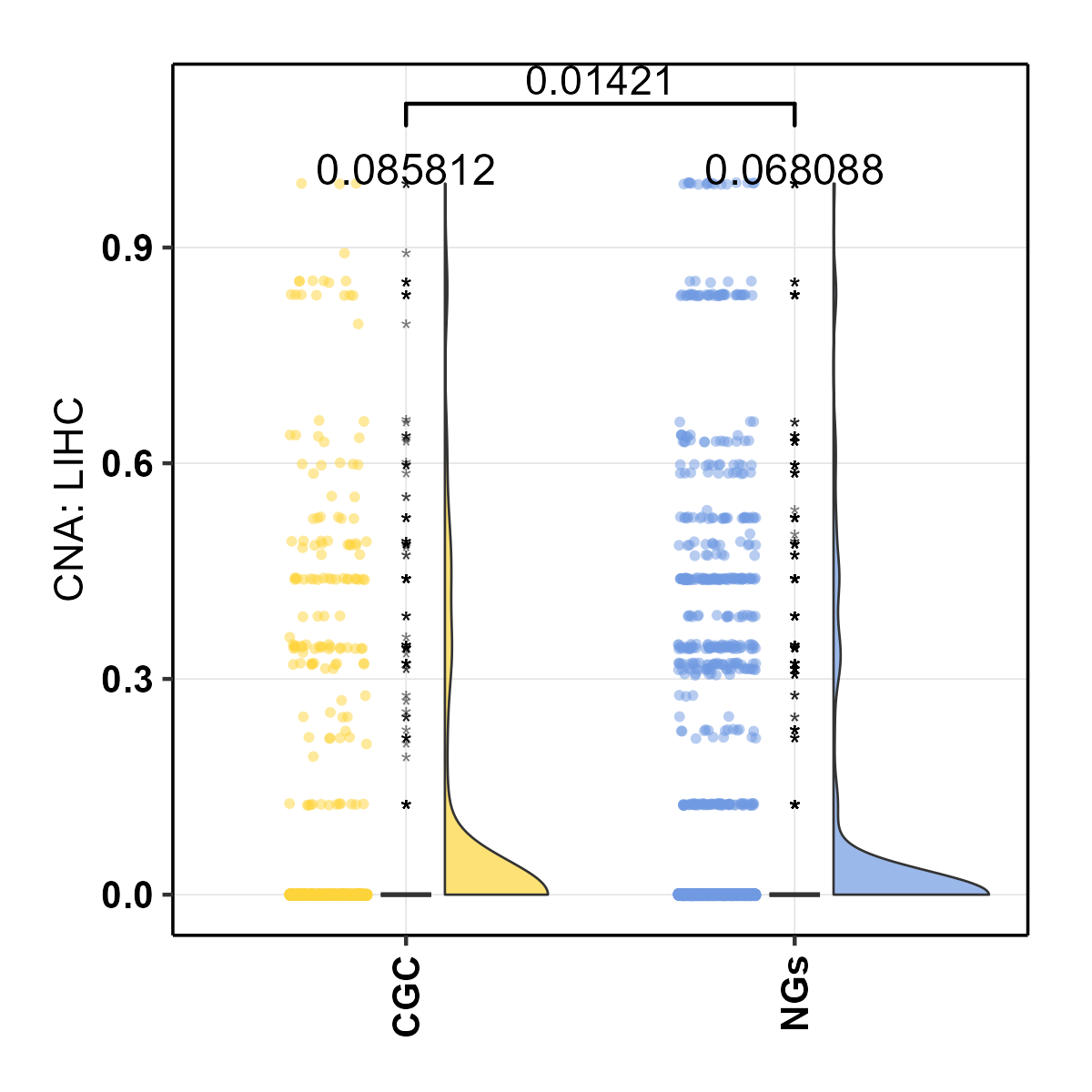

Supplement: Supplementary file 5 [file DataSheet2.ZIP › Supplementary file 5-2/PCNet/CNA_LIHC.png]

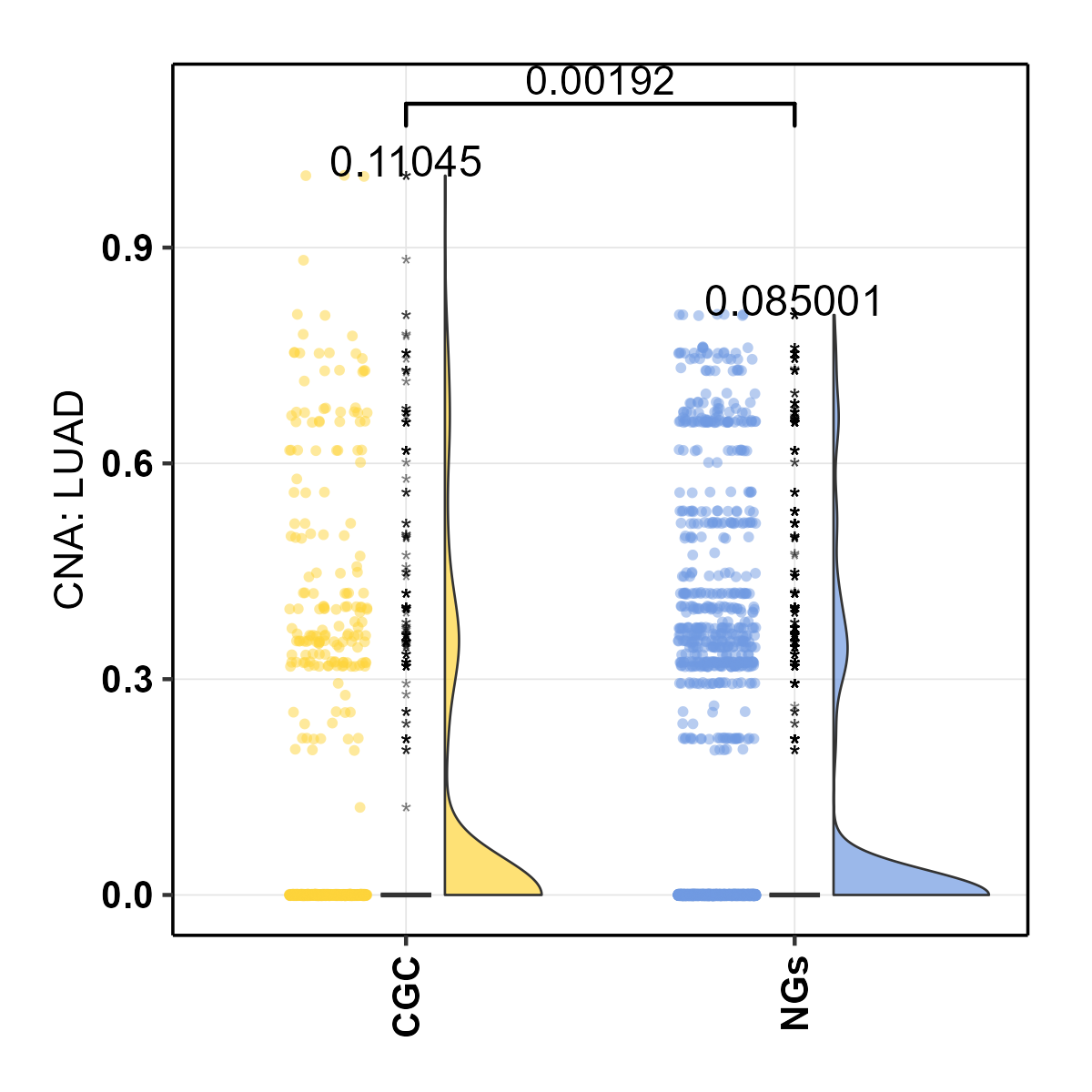

Supplement: Supplementary file 5 [file DataSheet2.ZIP › Supplementary file 5-2/PCNet/CNA_LUAD.png]

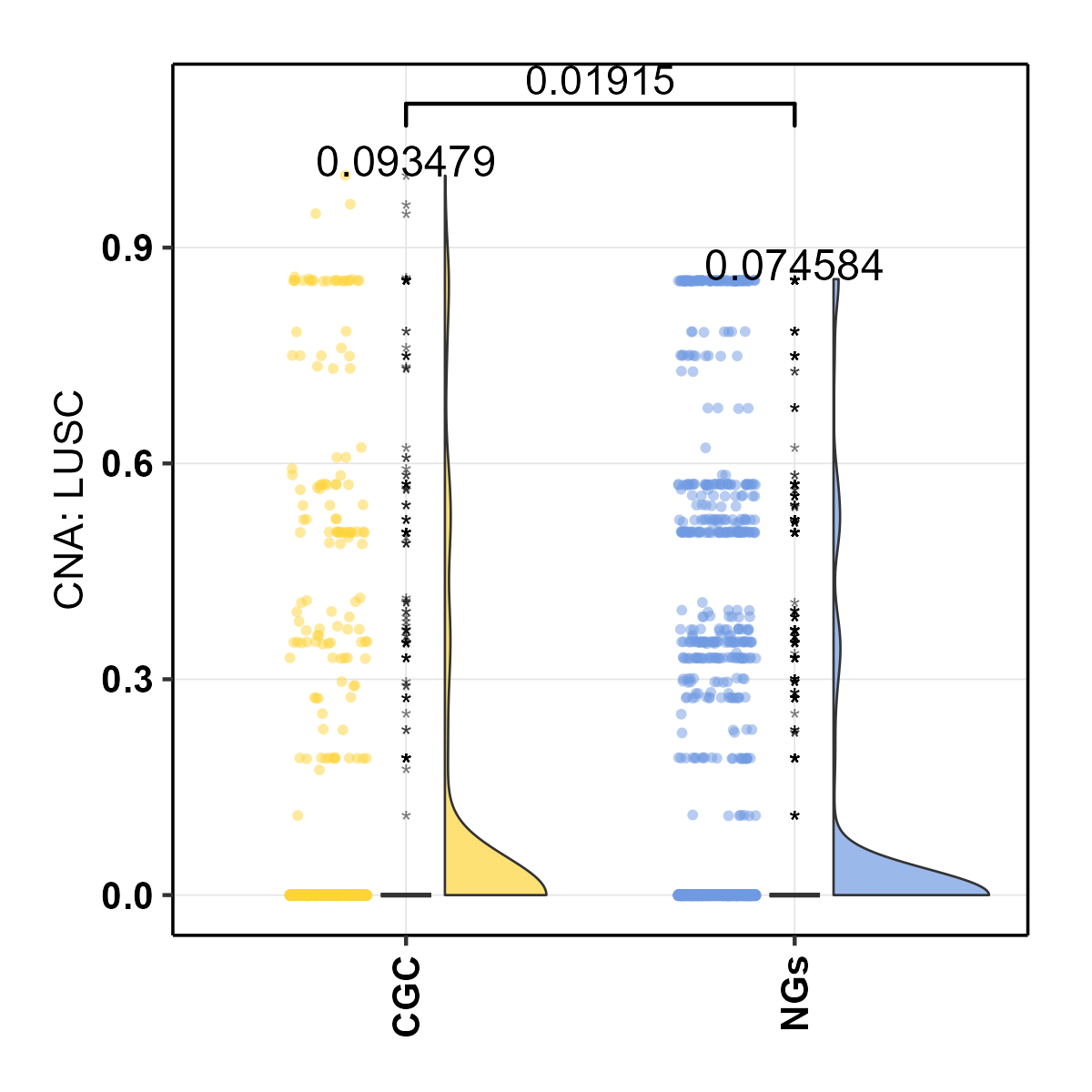

Supplement: Supplementary file 5 [file DataSheet2.ZIP › Supplementary file 5-2/PCNet/CNA_LUSC.png]

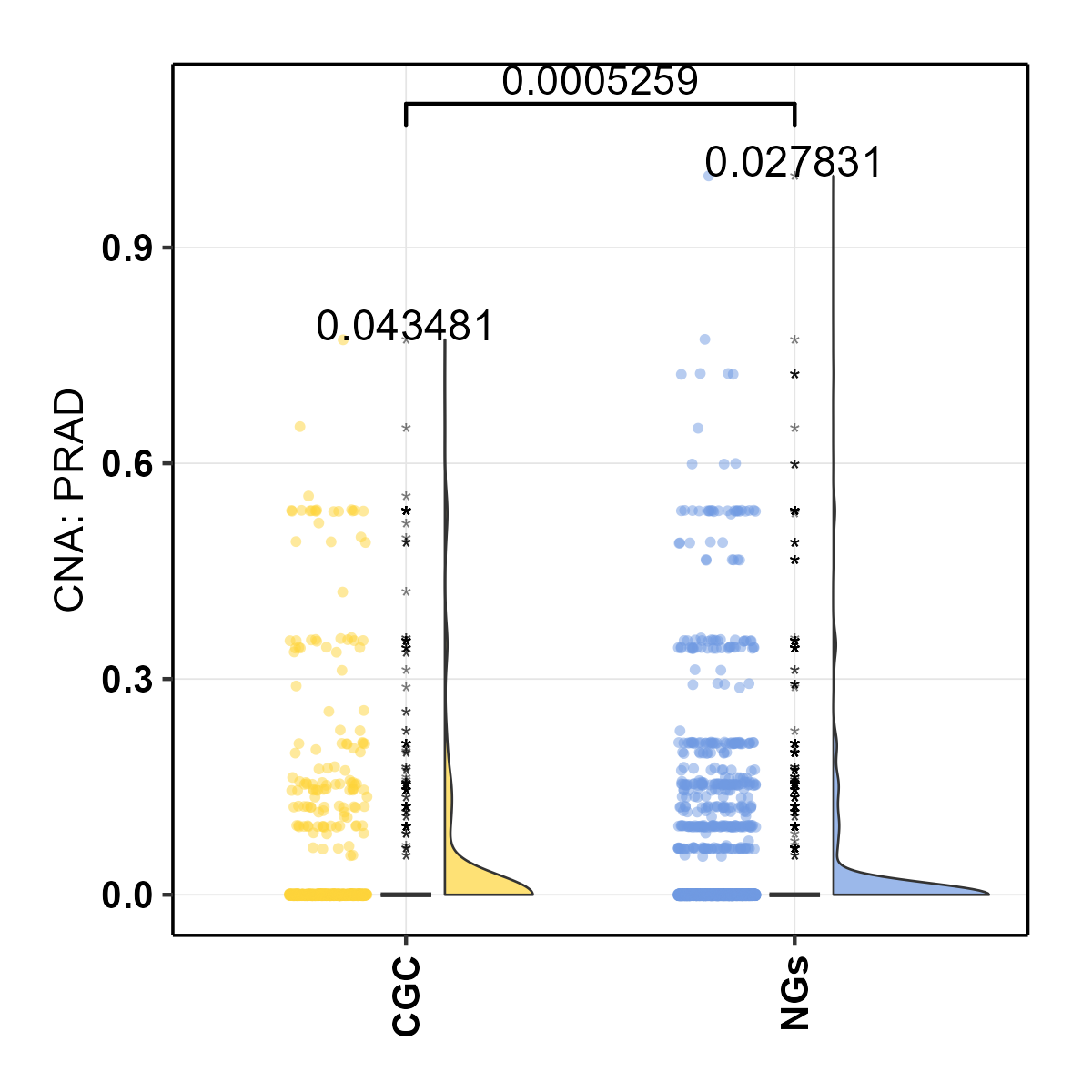

Supplement: Supplementary file 5 [file DataSheet2.ZIP › Supplementary file 5-2/PCNet/CNA_PRAD.png]

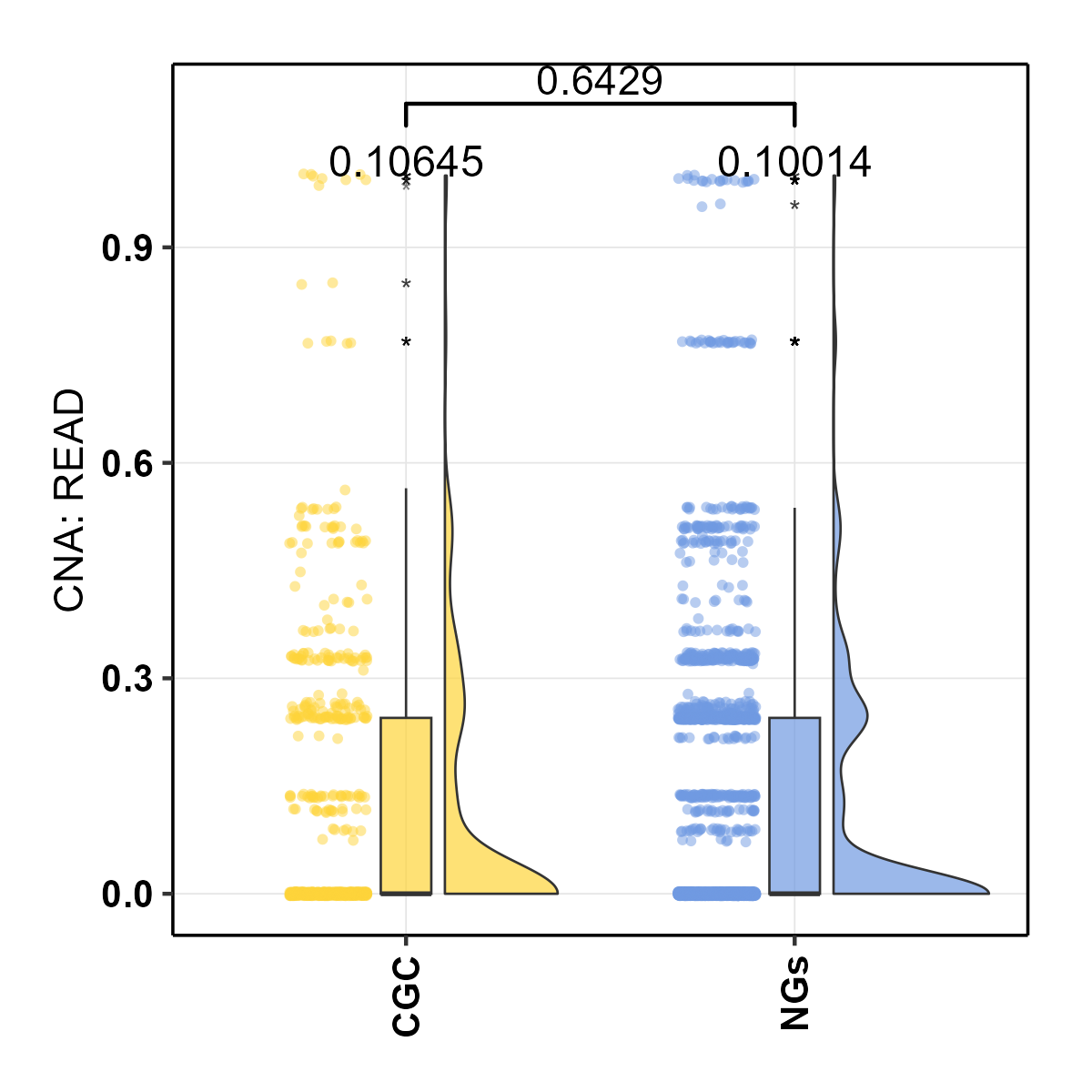

Supplement: Supplementary file 5 [file DataSheet2.ZIP › Supplementary file 5-2/PCNet/CNA_READ.png]

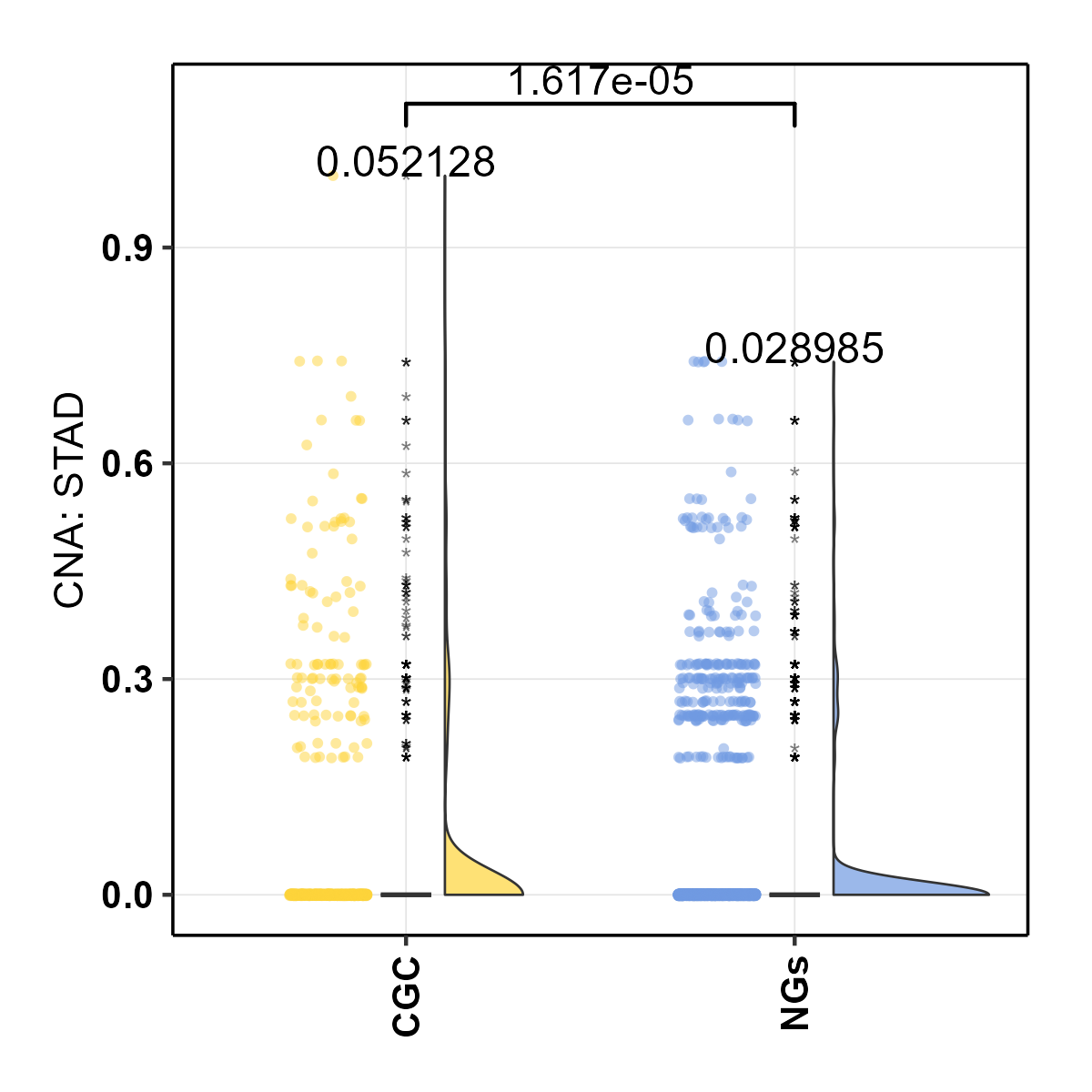

Supplement: Supplementary file 5 [file DataSheet2.ZIP › Supplementary file 5-2/PCNet/CNA_STAD.png]

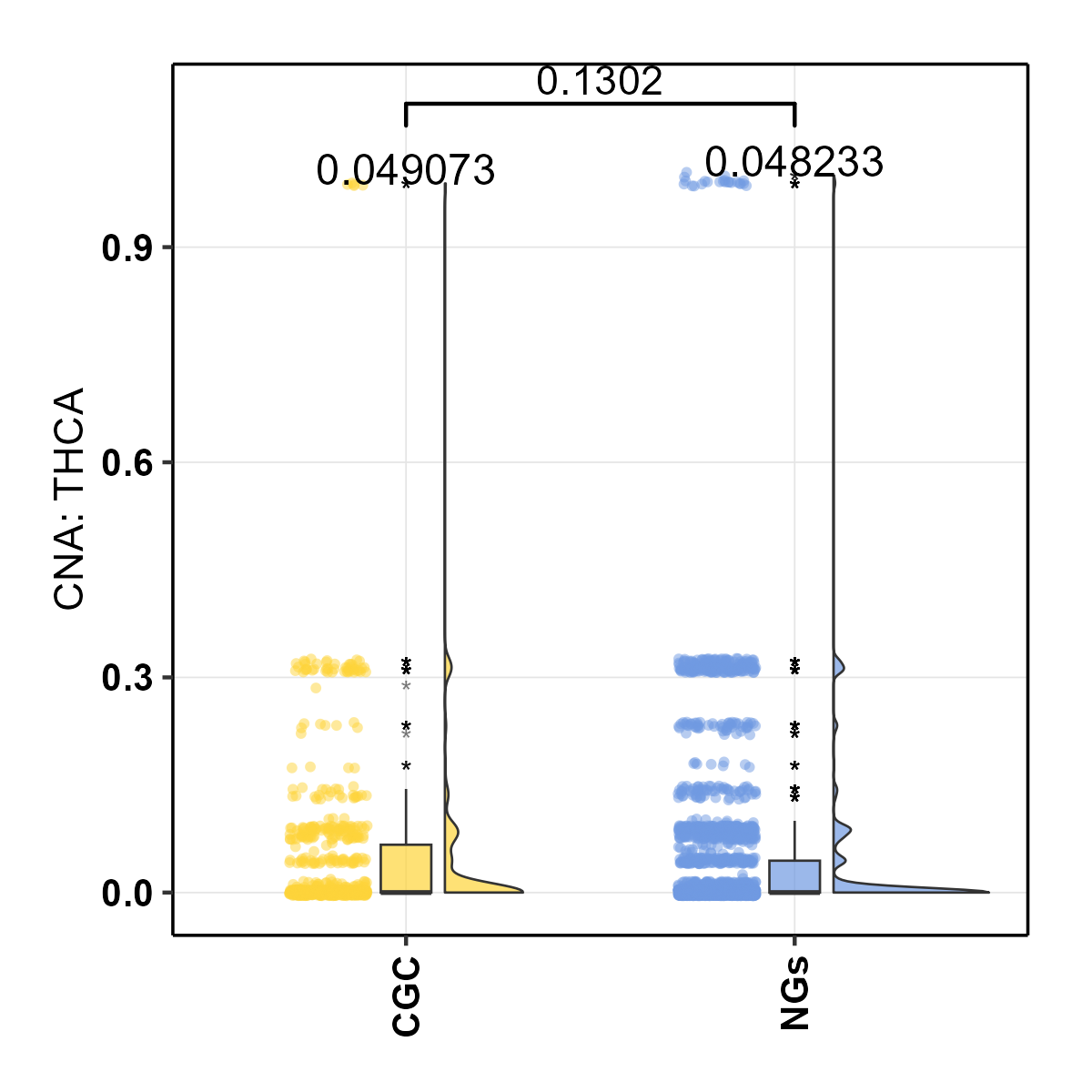

Supplement: Supplementary file 5 [file DataSheet2.ZIP › Supplementary file 5-2/PCNet/CNA_THCA.png]

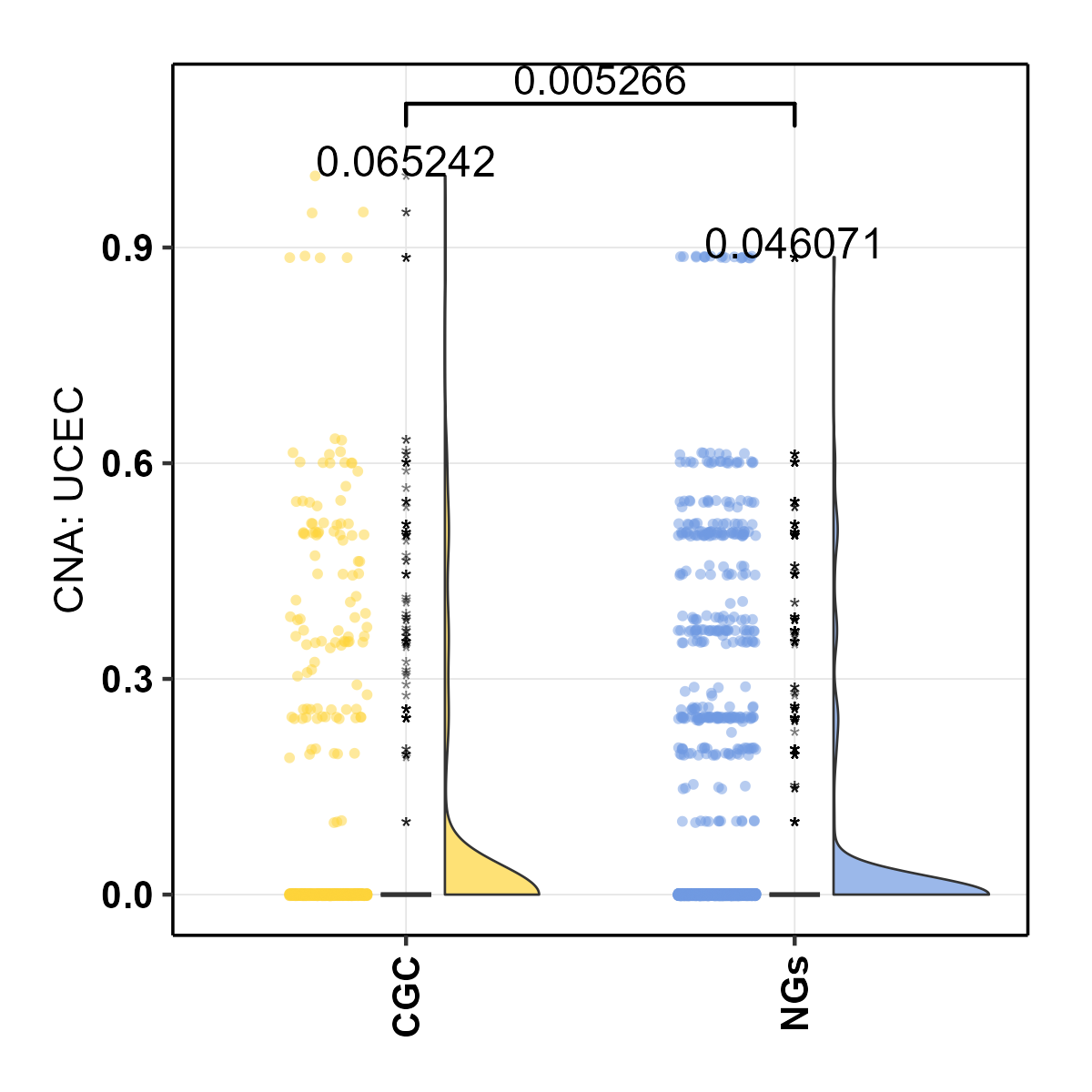

Supplement: Supplementary file 5 [file DataSheet2.ZIP › Supplementary file 5-2/PCNet/CNA_UCEC.png]

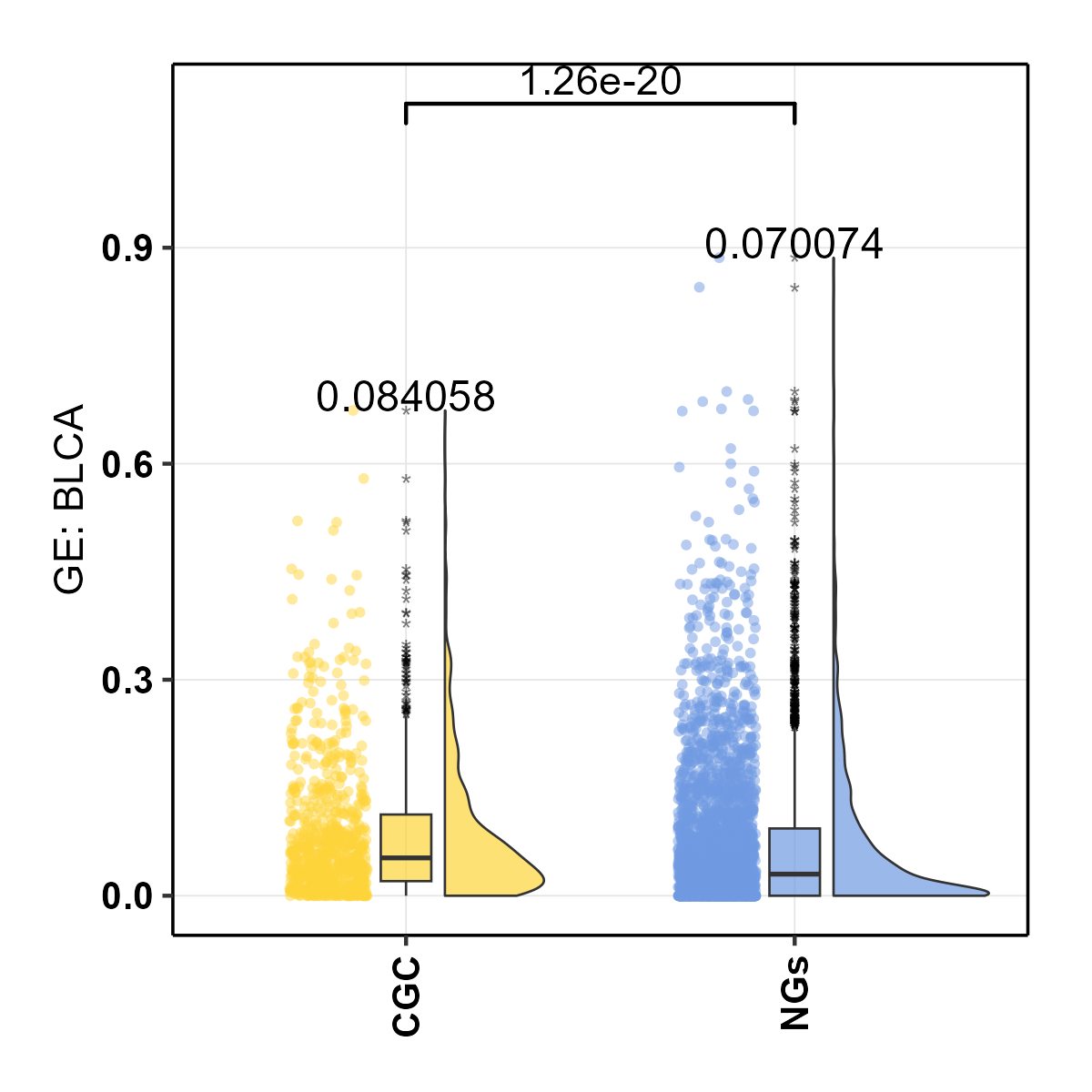

Supplement: Supplementary file 5 [file DataSheet2.ZIP › Supplementary file 5-2/PCNet/GE_BLCA.png]

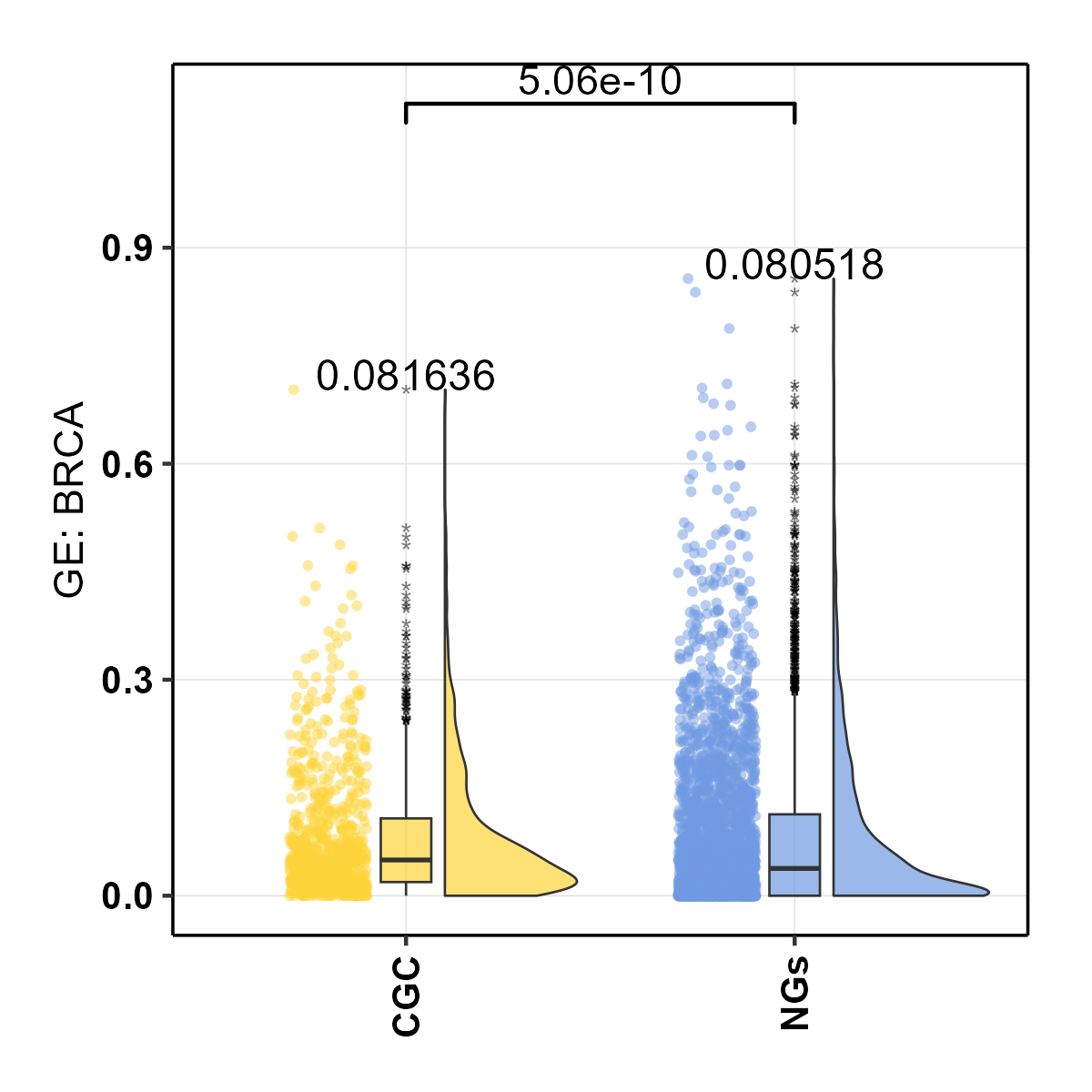

Supplement: Supplementary file 5 [file DataSheet2.ZIP › Supplementary file 5-2/PCNet/GE_BRCA.png]

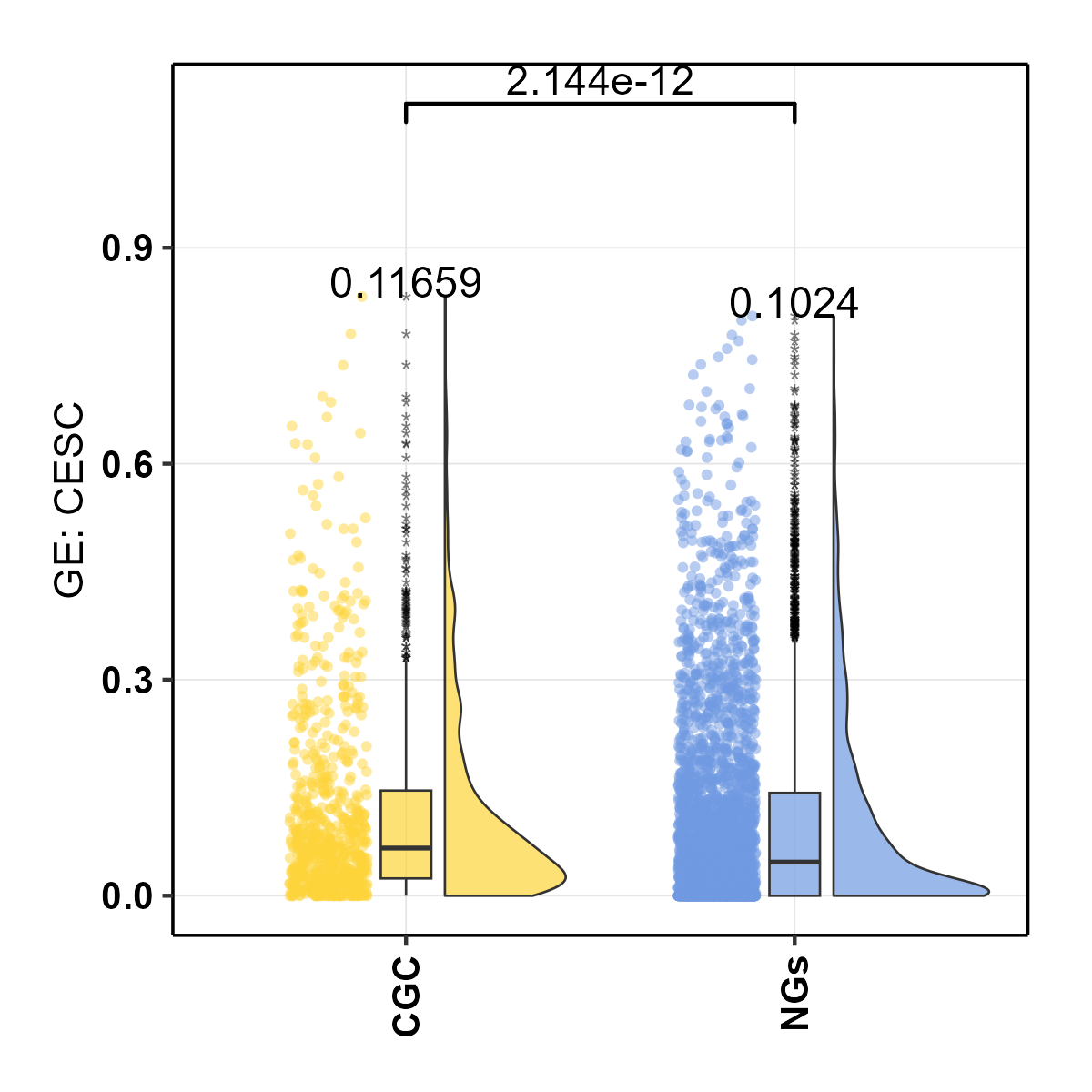

Supplement: Supplementary file 5 [file DataSheet2.ZIP › Supplementary file 5-2/PCNet/GE_CESC.png]

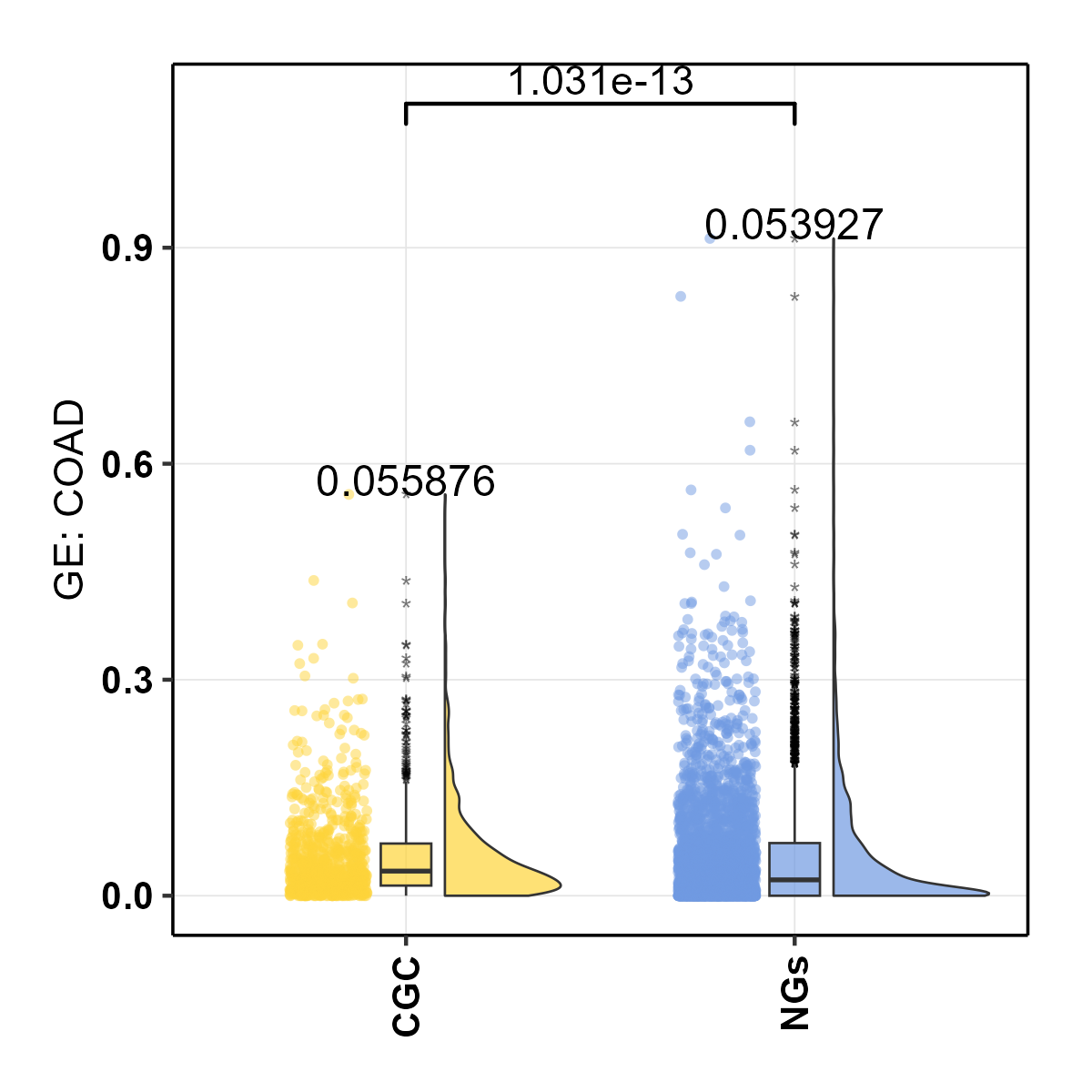

Supplement: Supplementary file 5 [file DataSheet2.ZIP › Supplementary file 5-2/PCNet/GE_COAD.png]

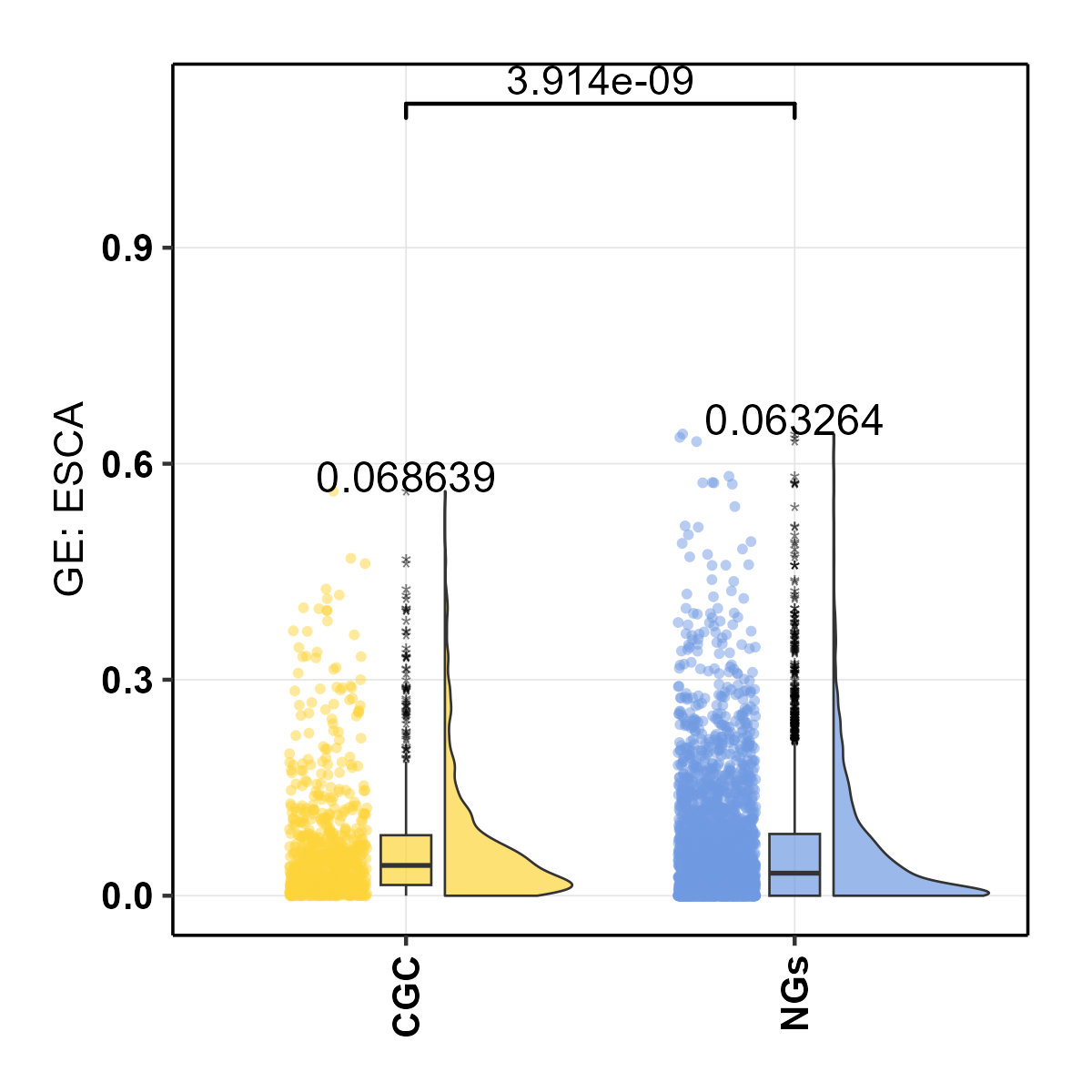

Supplement: Supplementary file 5 [file DataSheet2.ZIP › Supplementary file 5-2/PCNet/GE_ESCA.png]

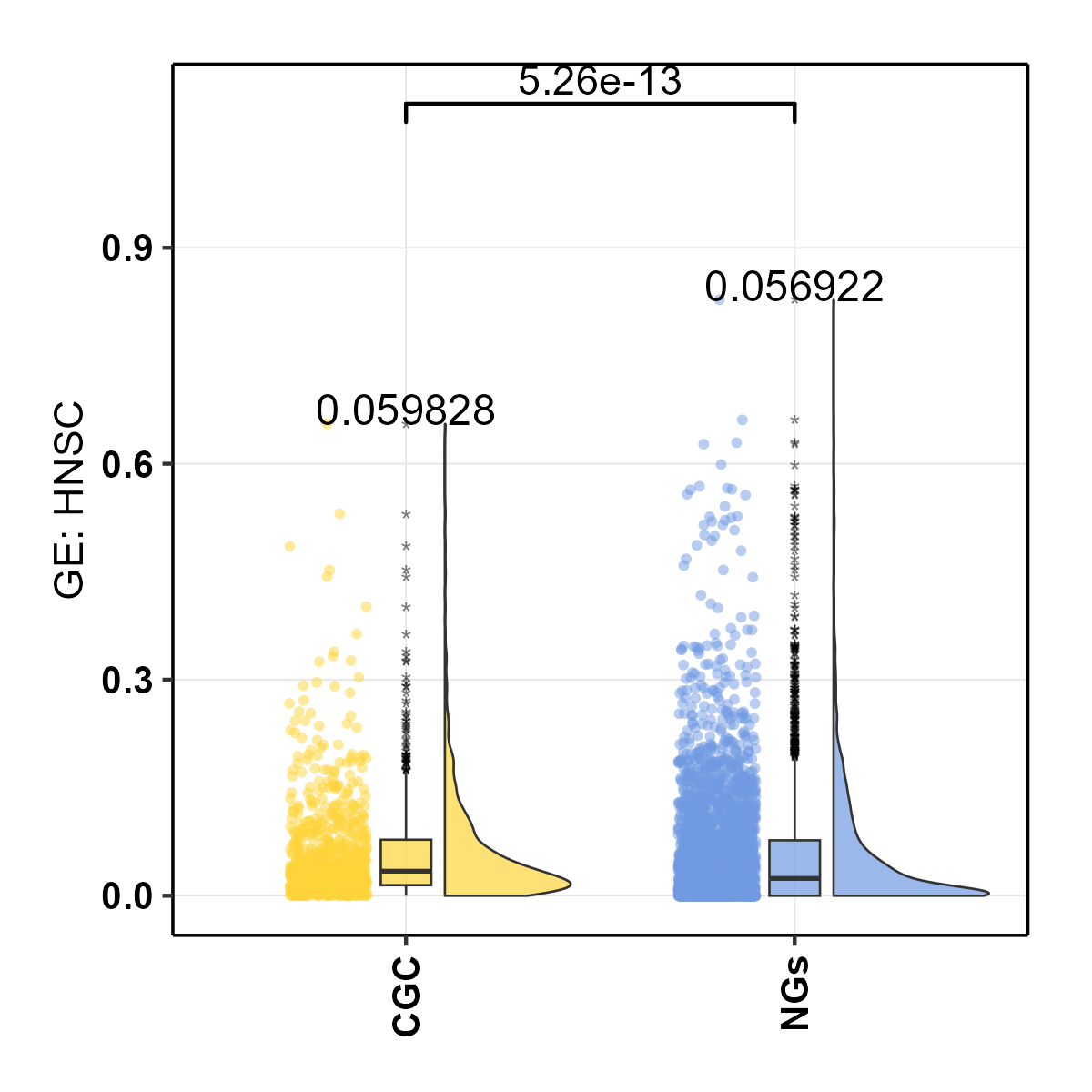

Supplement: Supplementary file 5 [file DataSheet2.ZIP › Supplementary file 5-2/PCNet/GE_HNSC.png]

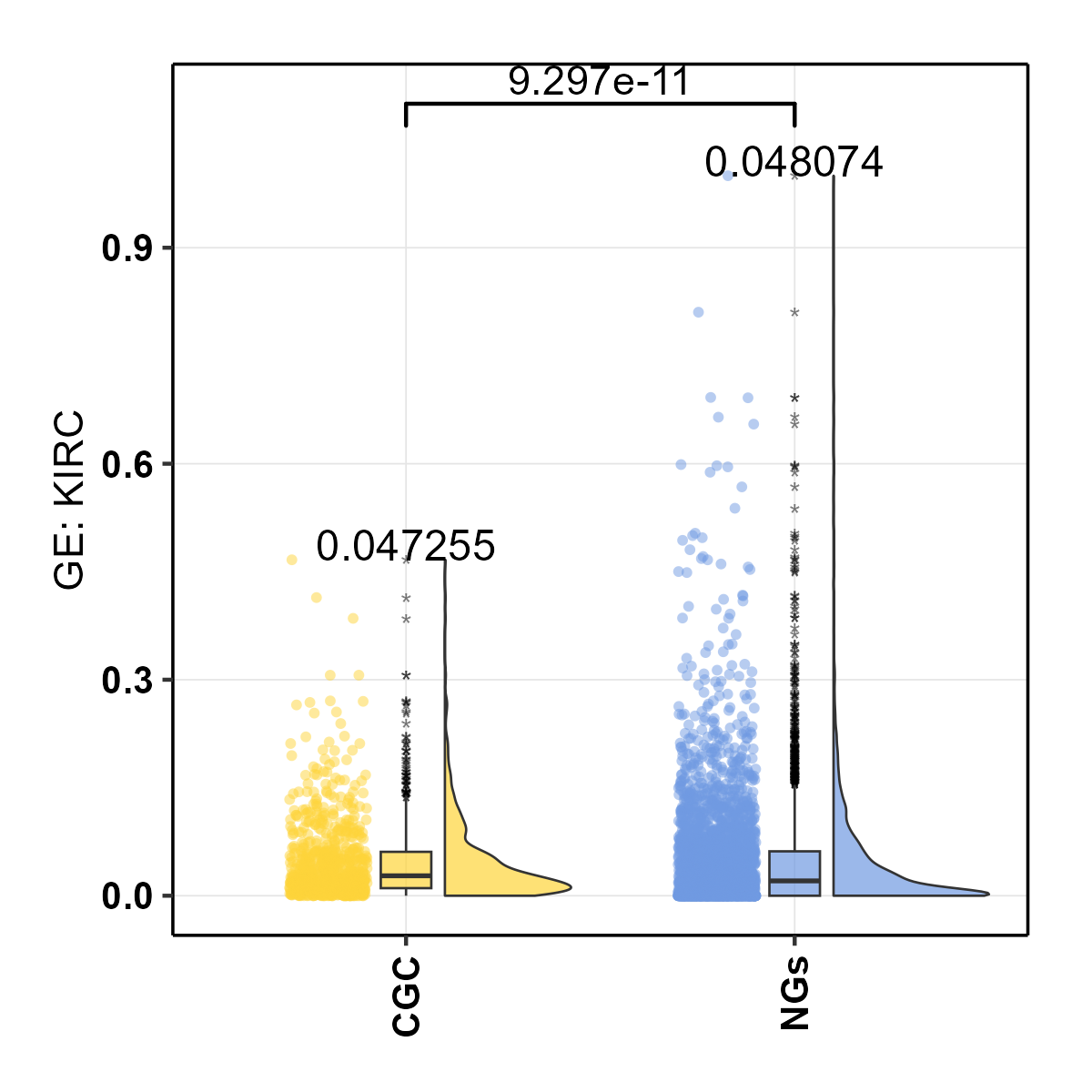

Supplement: Supplementary file 5 [file DataSheet2.ZIP › Supplementary file 5-2/PCNet/GE_KIRC.png]

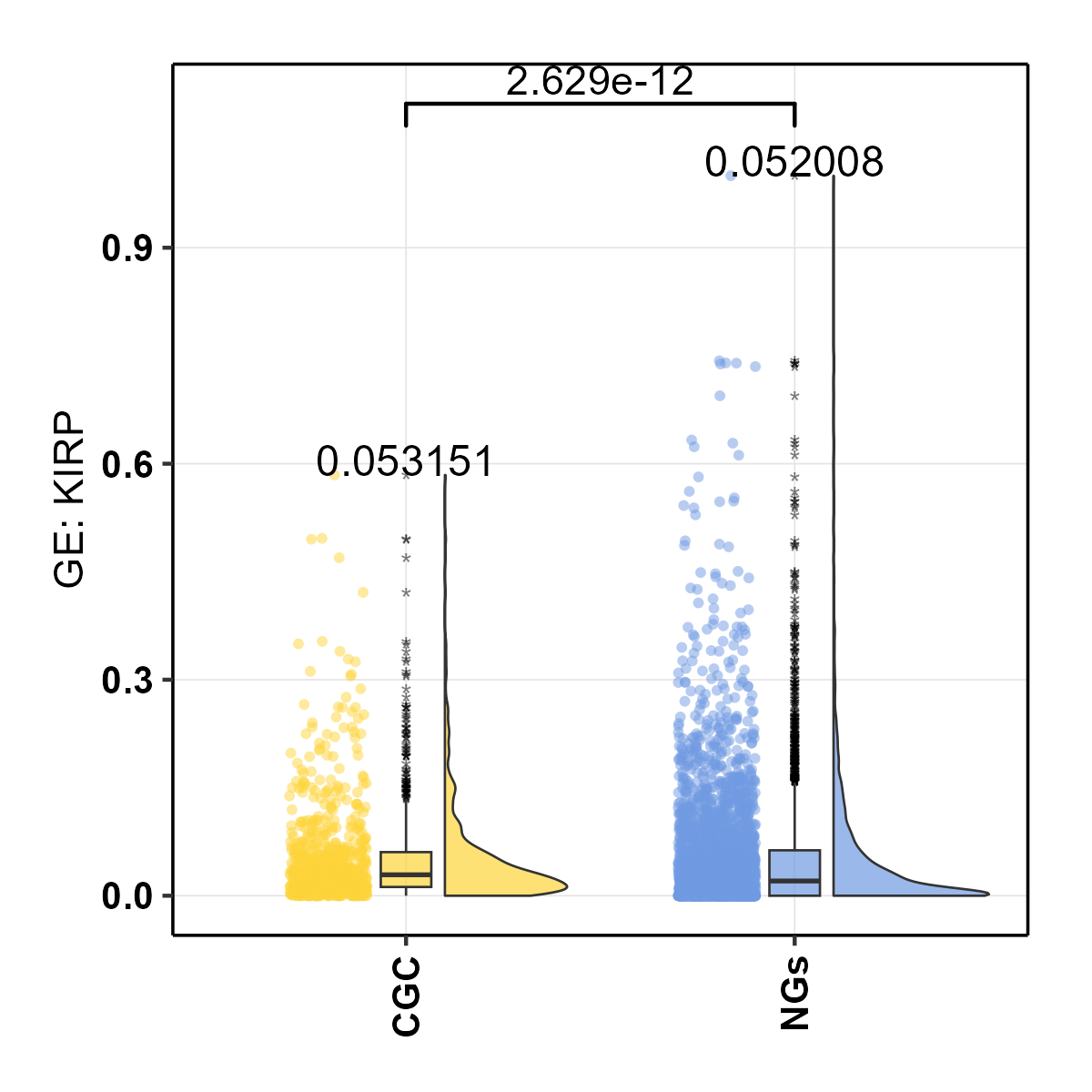

Supplement: Supplementary file 5 [file DataSheet2.ZIP › Supplementary file 5-2/PCNet/GE_KIRP.png]

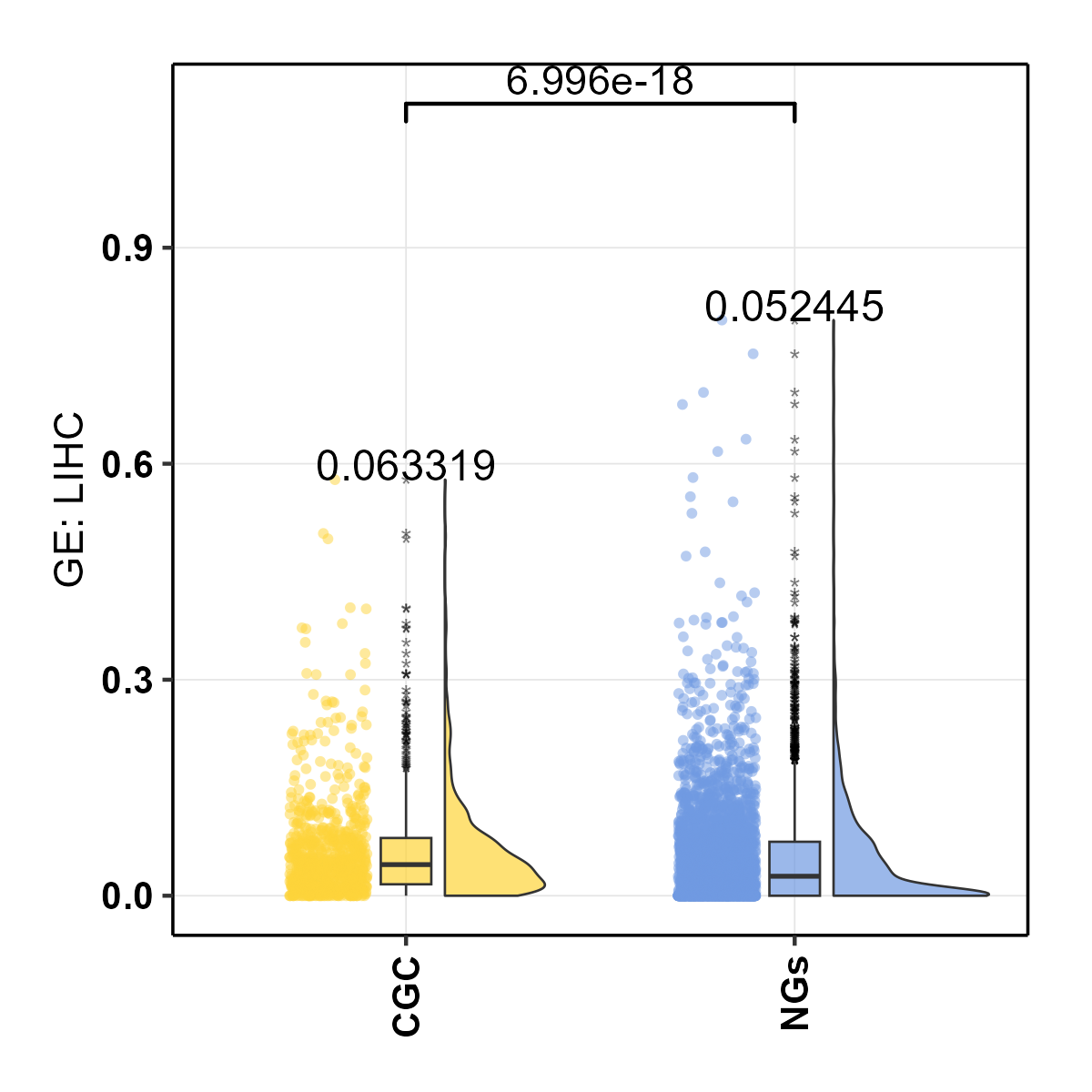

Supplement: Supplementary file 5 [file DataSheet2.ZIP › Supplementary file 5-2/PCNet/GE_LIHC.png]

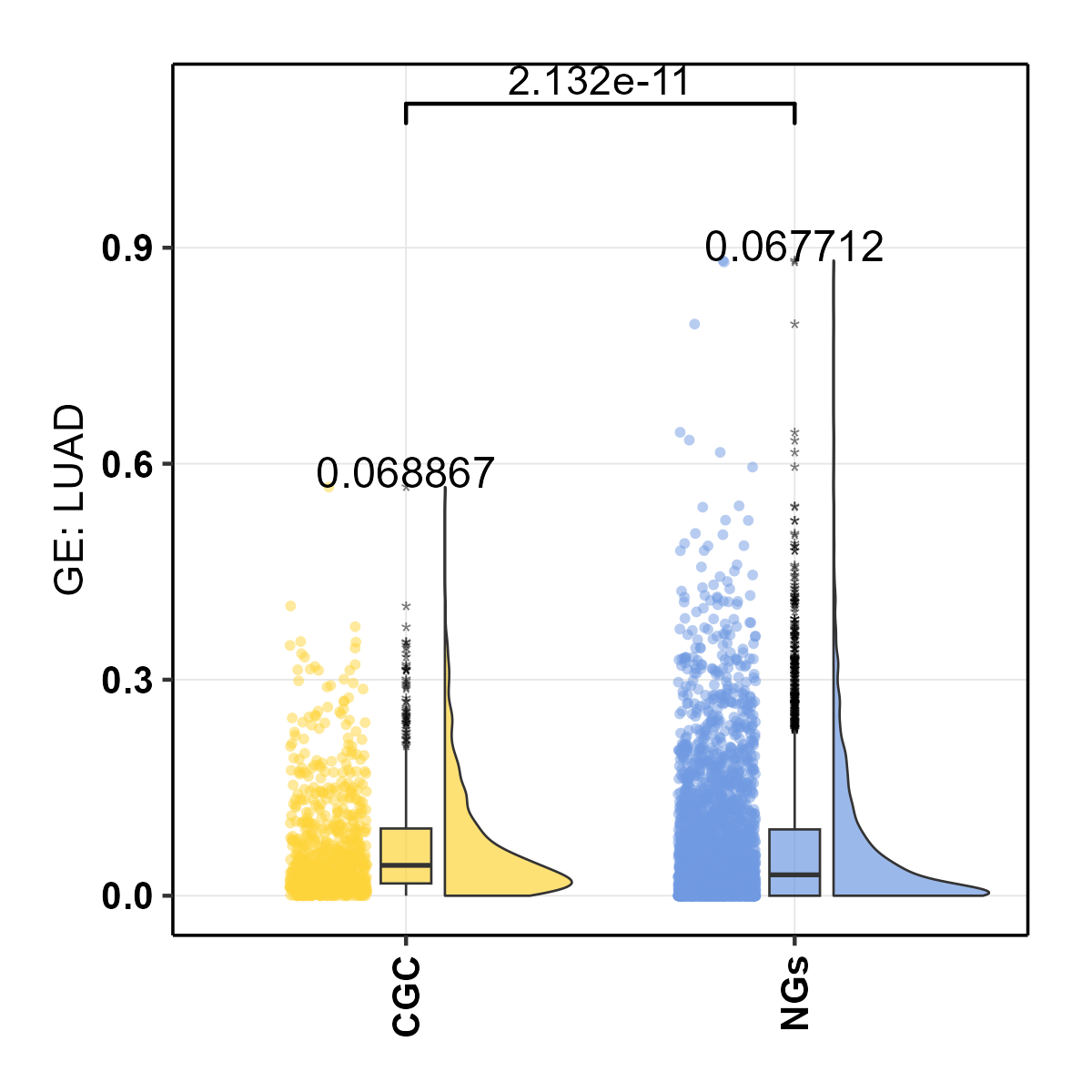

Supplement: Supplementary file 5 [file DataSheet2.ZIP › Supplementary file 5-2/PCNet/GE_LUAD.png]

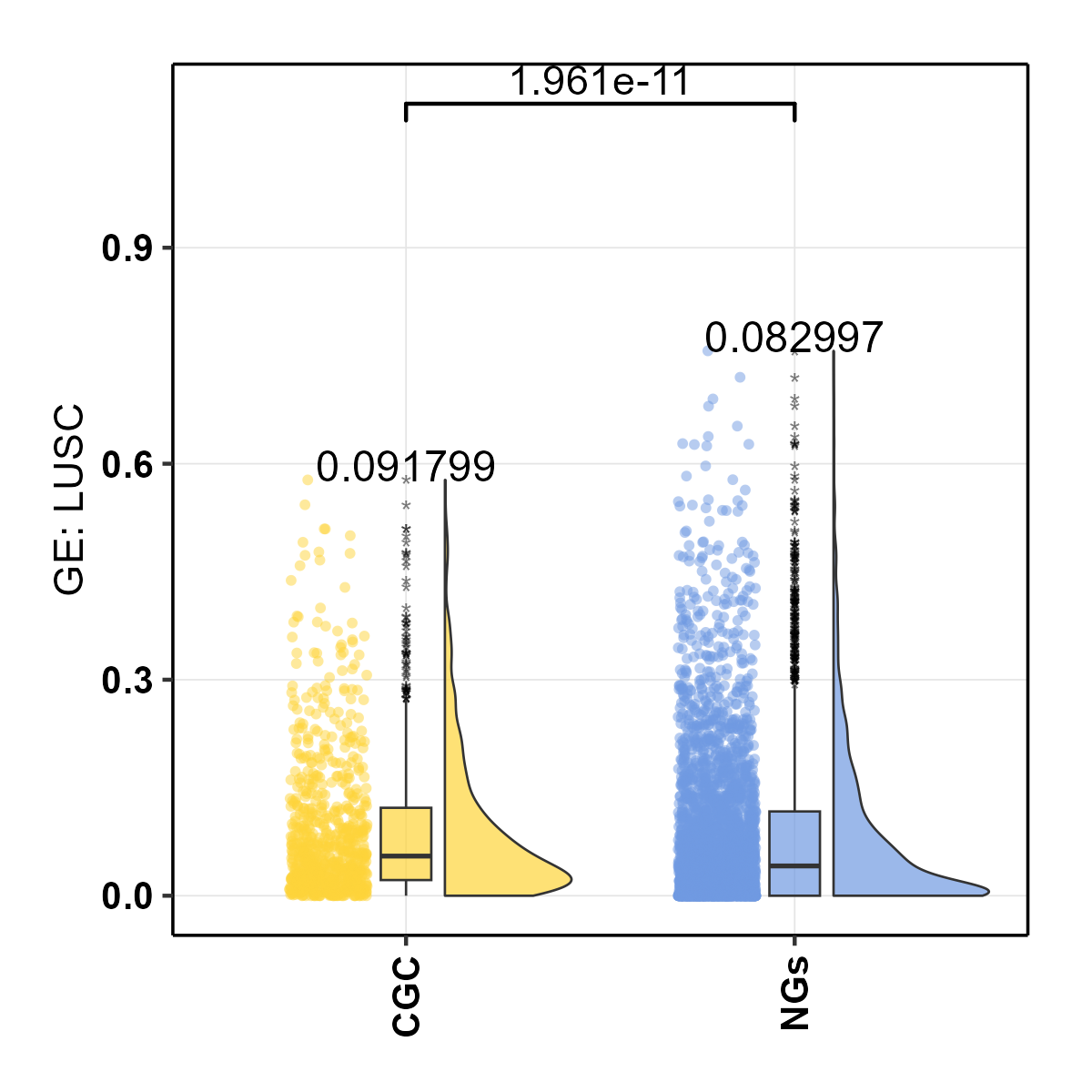

Supplement: Supplementary file 5 [file DataSheet2.ZIP › Supplementary file 5-2/PCNet/GE_LUSC.png]

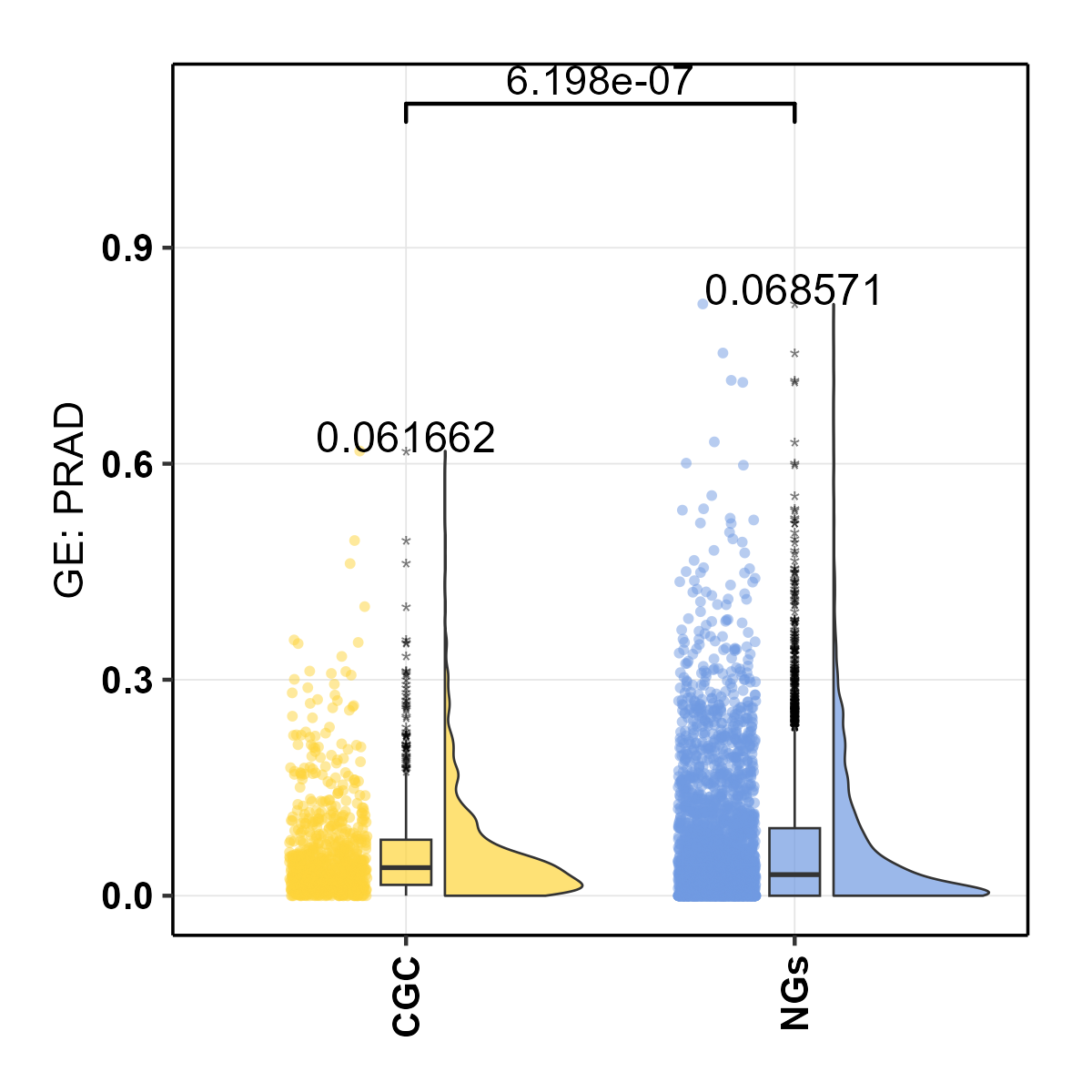

Supplement: Supplementary file 5 [file DataSheet2.ZIP › Supplementary file 5-2/PCNet/GE_PRAD.png]

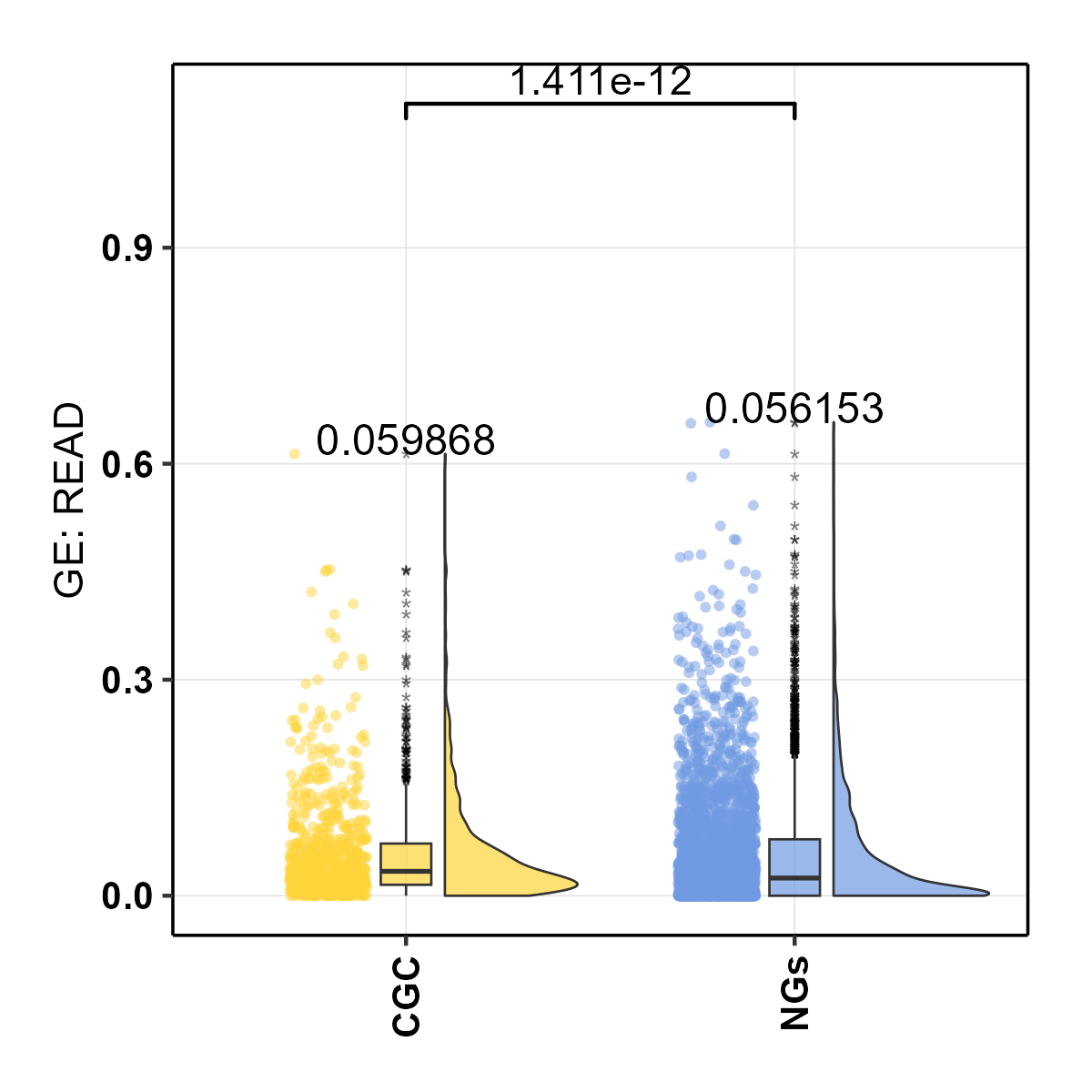

Supplement: Supplementary file 5 [file DataSheet2.ZIP › Supplementary file 5-2/PCNet/GE_READ.png]

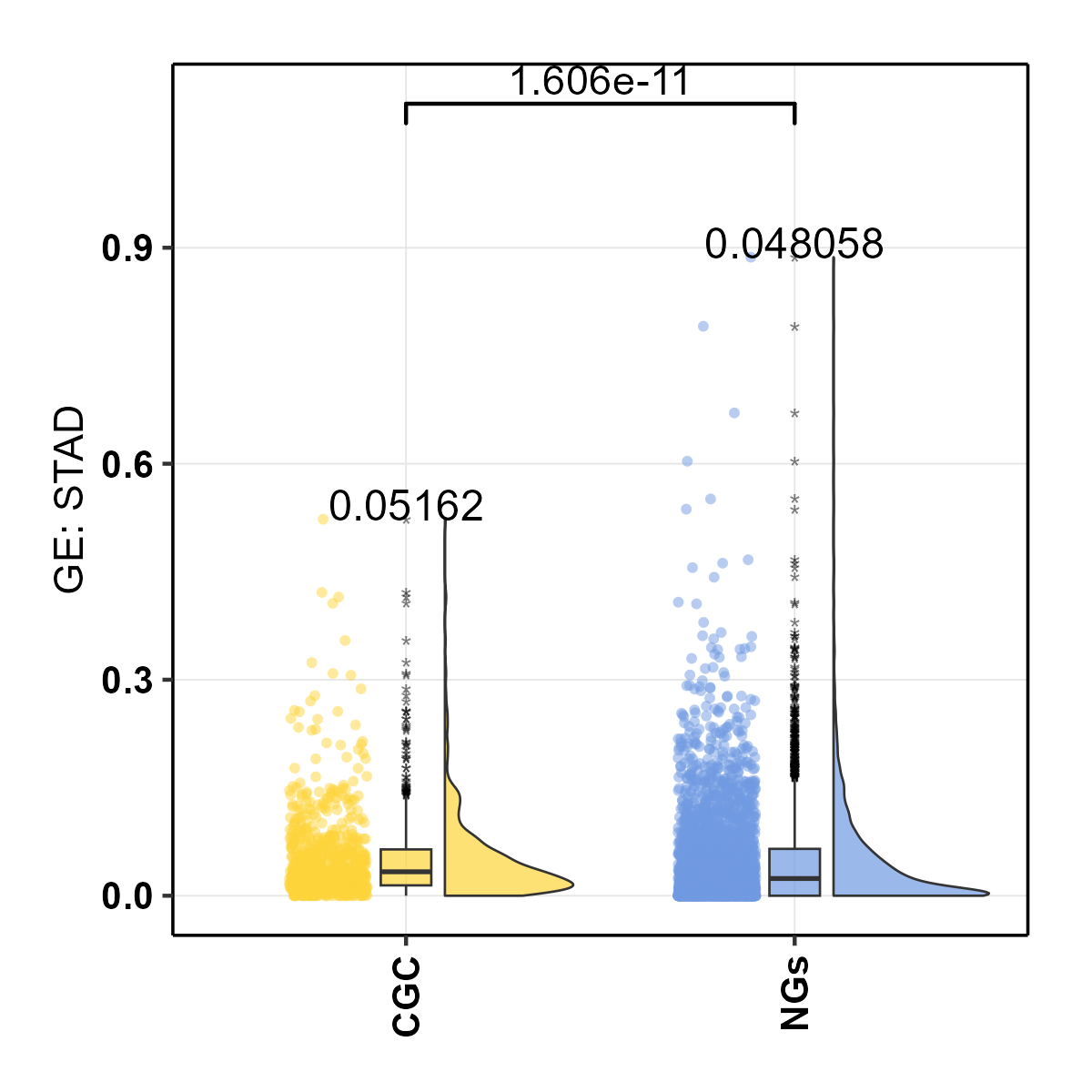

Supplement: Supplementary file 5 [file DataSheet2.ZIP › Supplementary file 5-2/PCNet/GE_STAD.png]

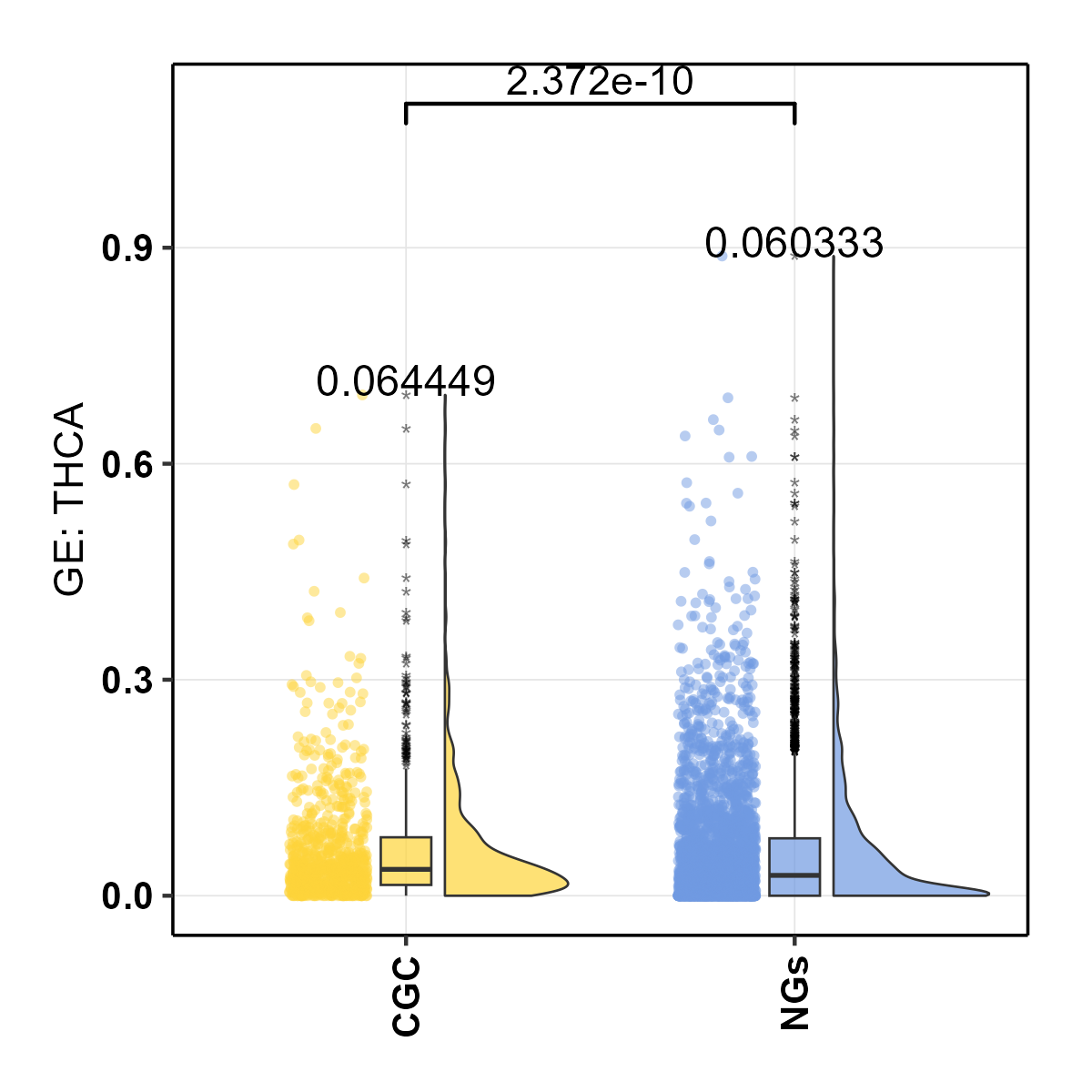

Supplement: Supplementary file 5 [file DataSheet2.ZIP › Supplementary file 5-2/PCNet/GE_THCA.png]

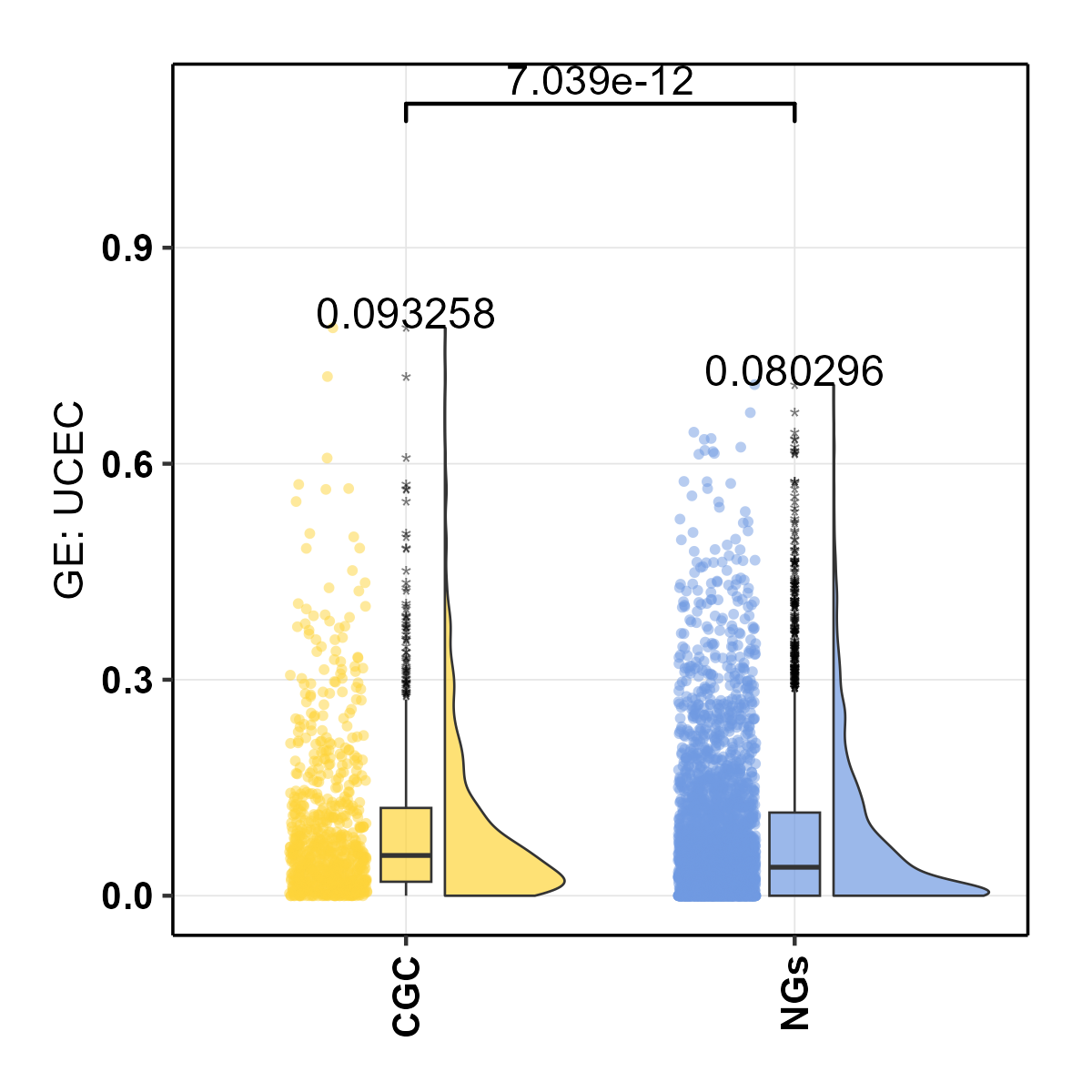

Supplement: Supplementary file 5 [file DataSheet2.ZIP › Supplementary file 5-2/PCNet/GE_UCEC.png]

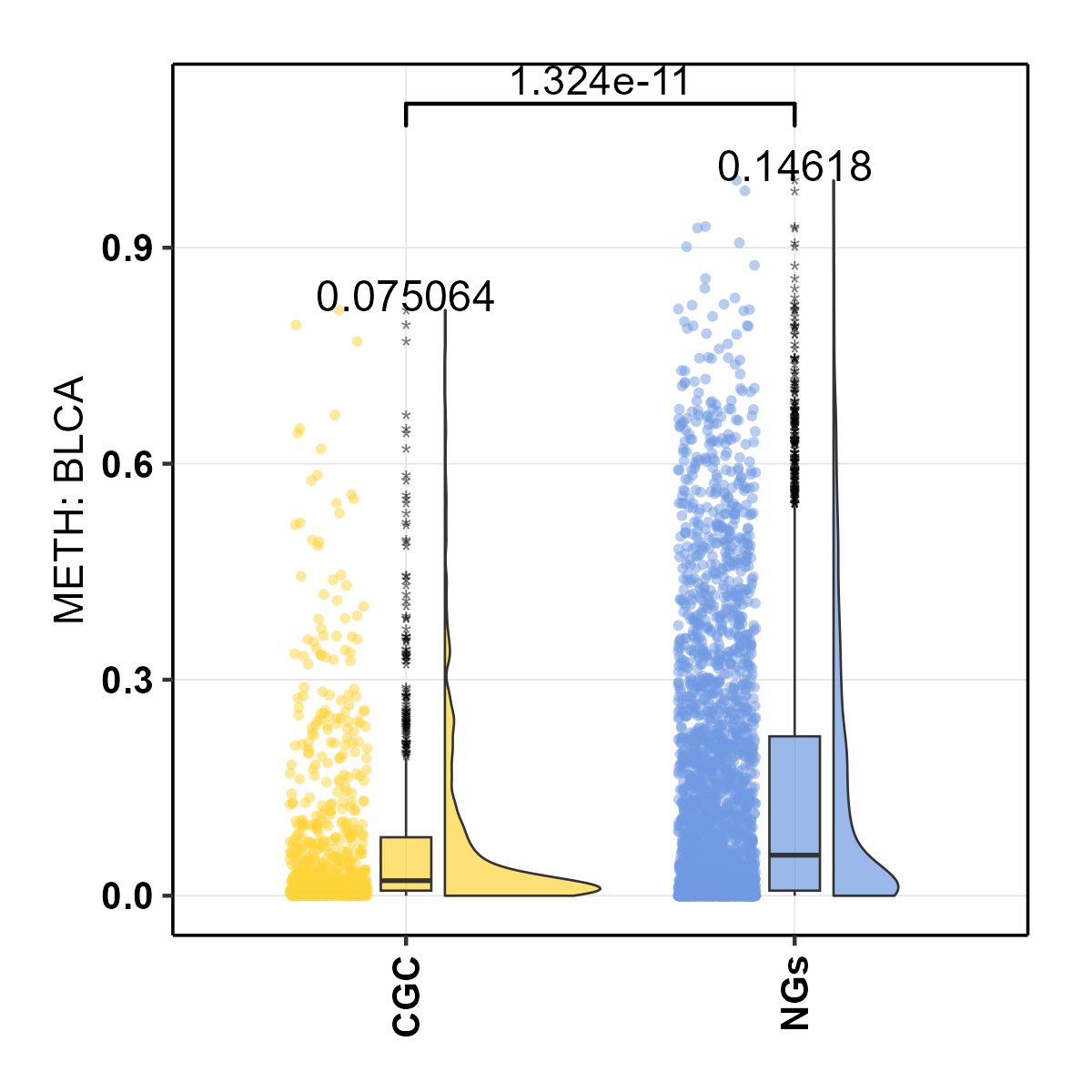

Supplement: Supplementary file 5 [file DataSheet2.ZIP › Supplementary file 5-2/PCNet/METH_BLCA.png]

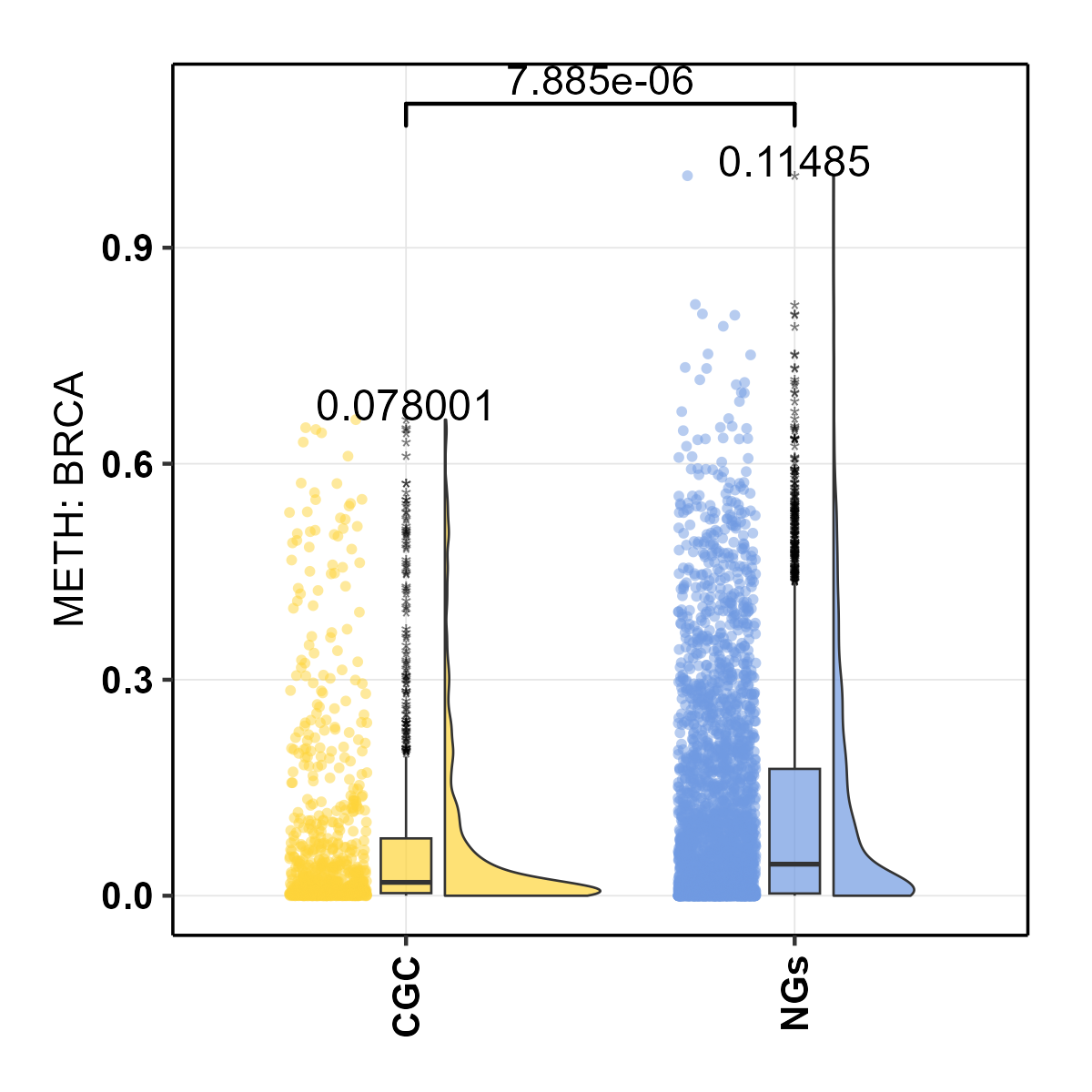

Supplement: Supplementary file 5 [file DataSheet2.ZIP › Supplementary file 5-2/PCNet/METH_BRCA.png]

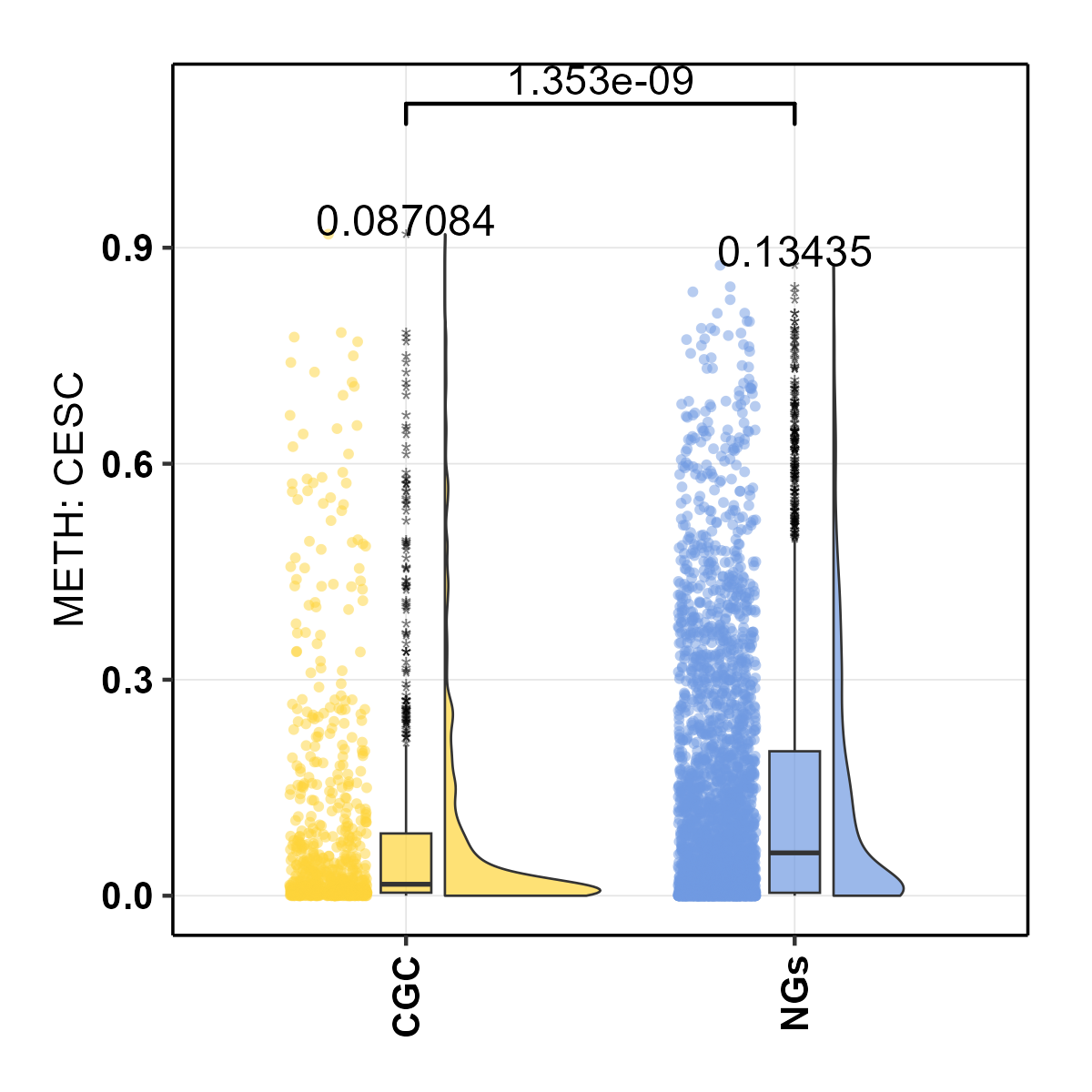

Supplement: Supplementary file 5 [file DataSheet2.ZIP › Supplementary file 5-2/PCNet/METH_CESC.png]

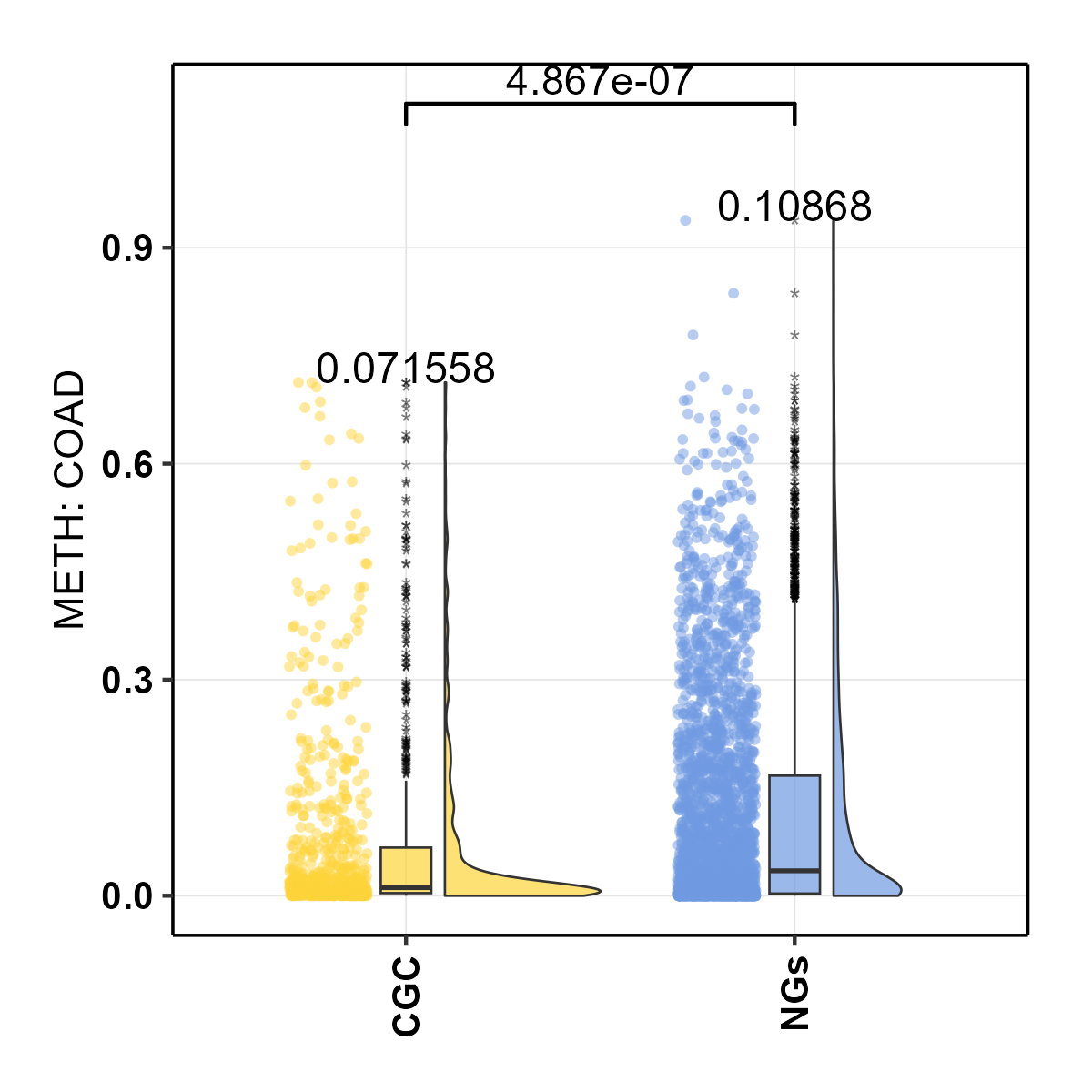

Supplement: Supplementary file 5 [file DataSheet2.ZIP › Supplementary file 5-2/PCNet/METH_COAD.png]

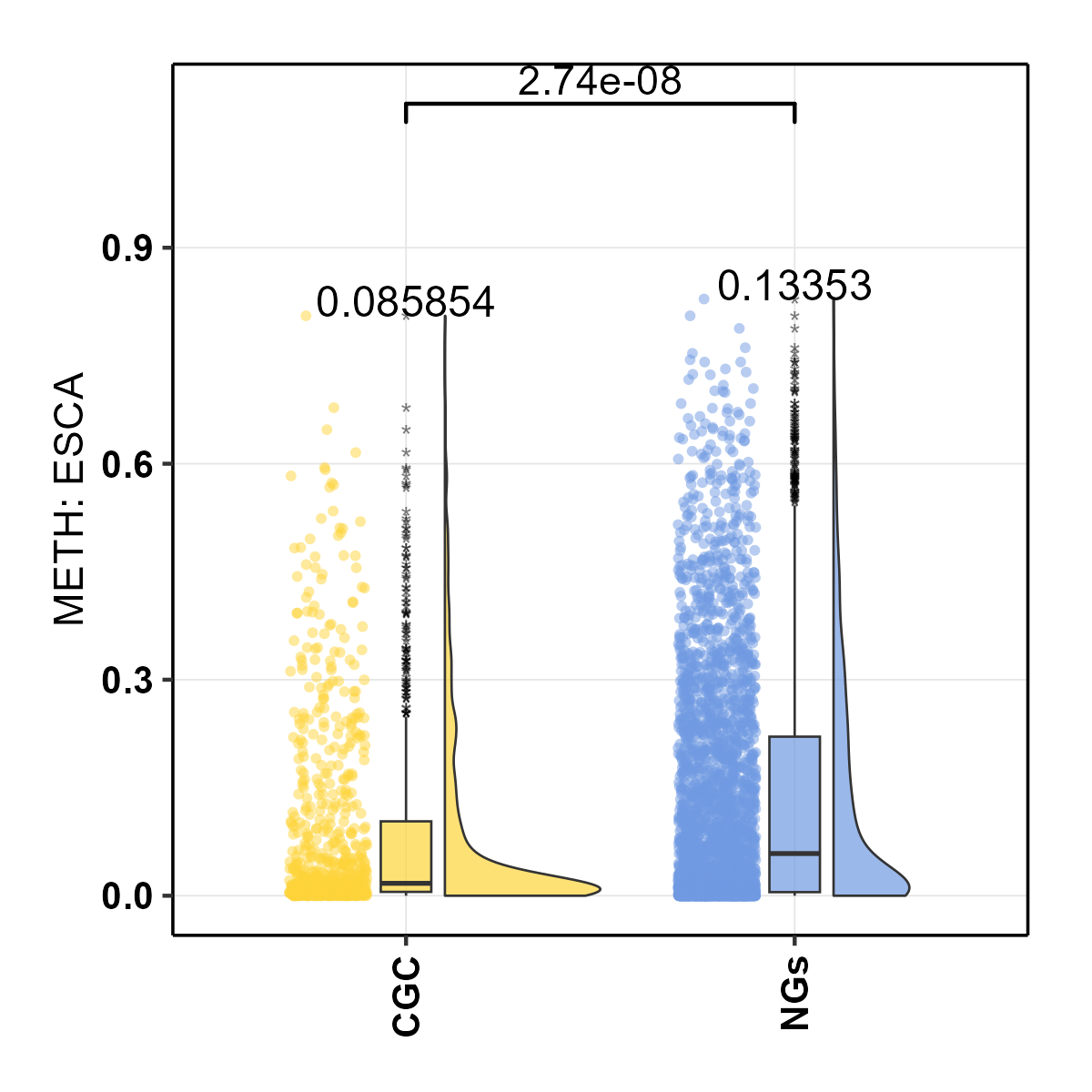

Supplement: Supplementary file 5 [file DataSheet2.ZIP › Supplementary file 5-2/PCNet/METH_ESCA.png]

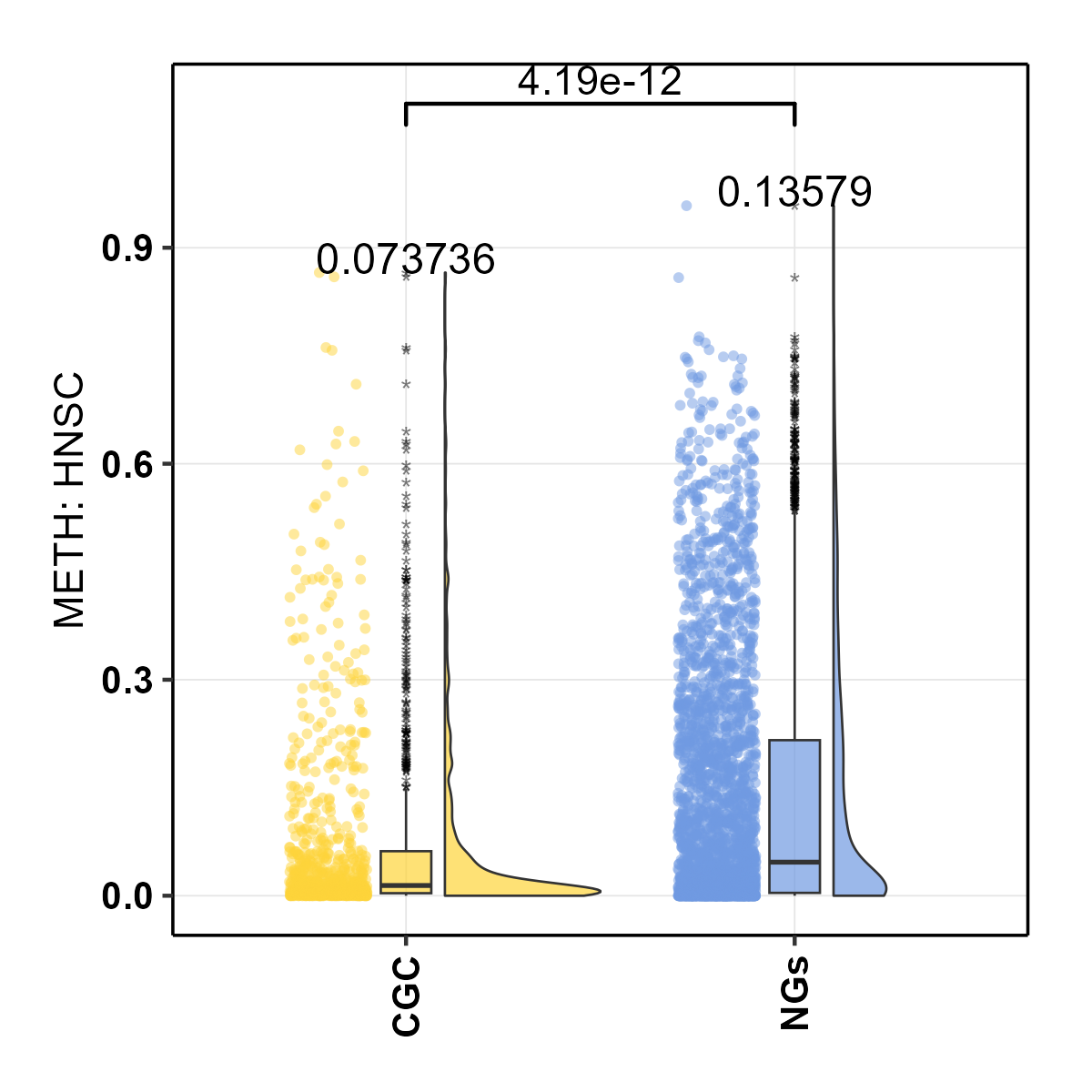

Supplement: Supplementary file 5 [file DataSheet2.ZIP › Supplementary file 5-2/PCNet/METH_HNSC.png]

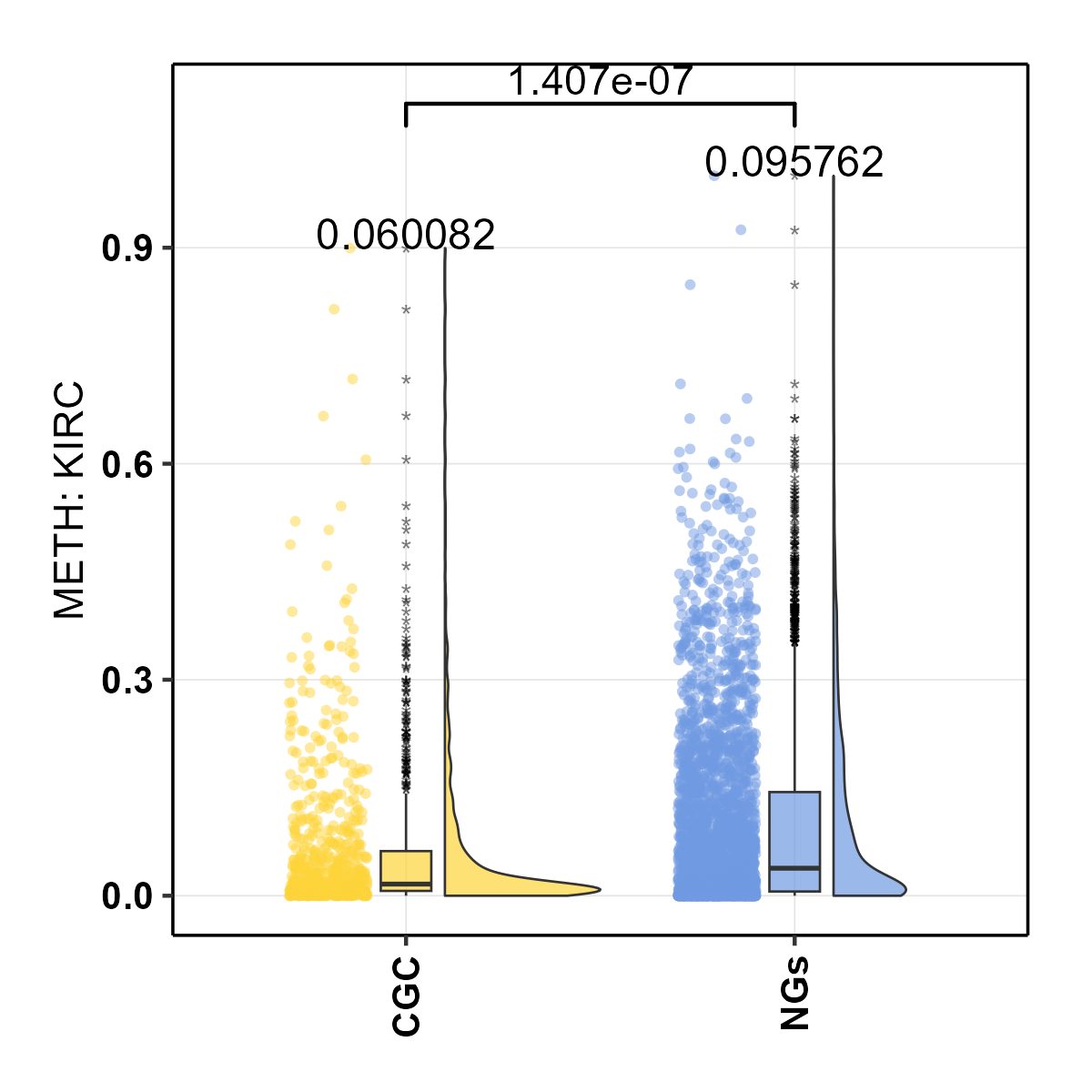

Supplement: Supplementary file 5 [file DataSheet2.ZIP › Supplementary file 5-2/PCNet/METH_KIRC.png]

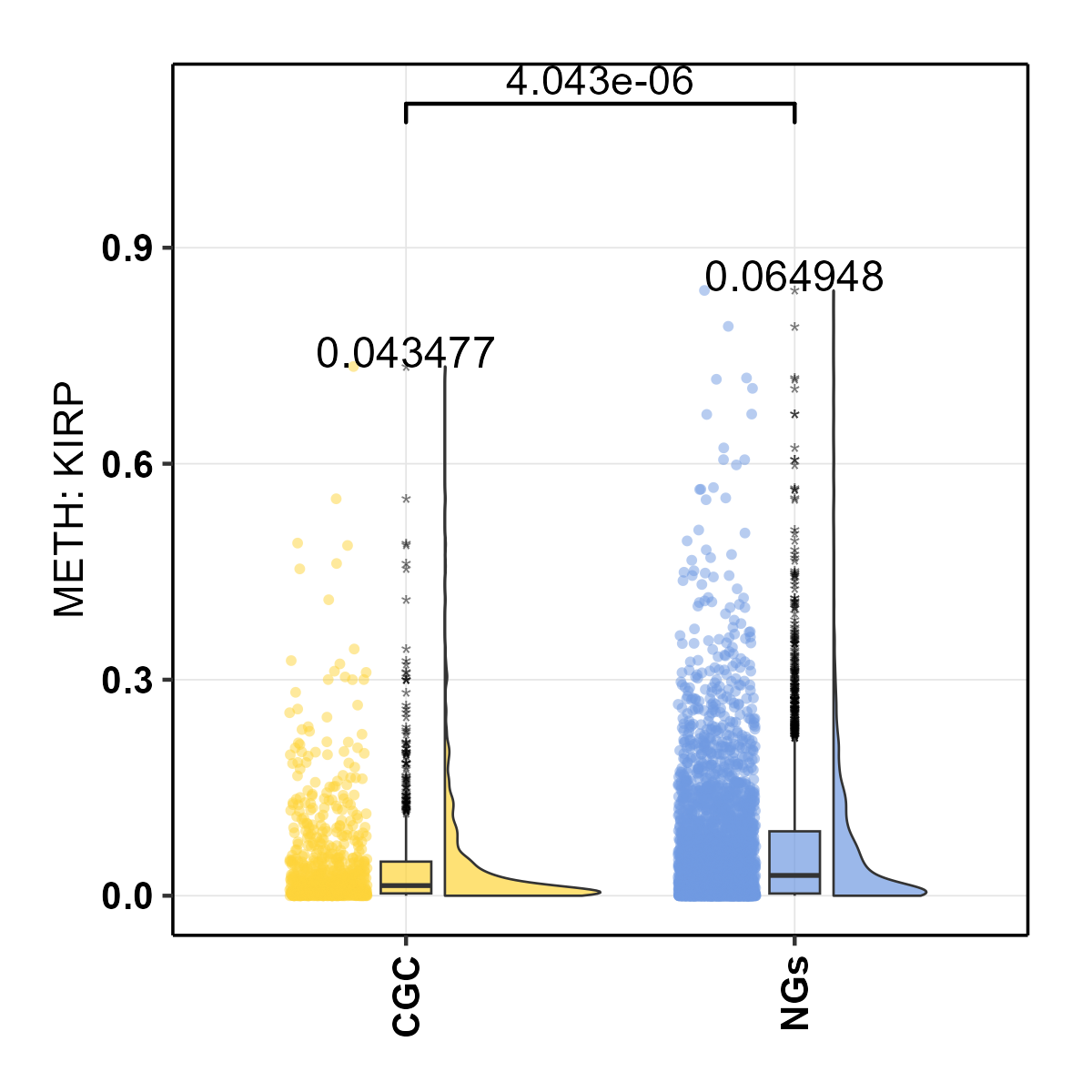

Supplement: Supplementary file 5 [file DataSheet2.ZIP › Supplementary file 5-2/PCNet/METH_KIRP.png]

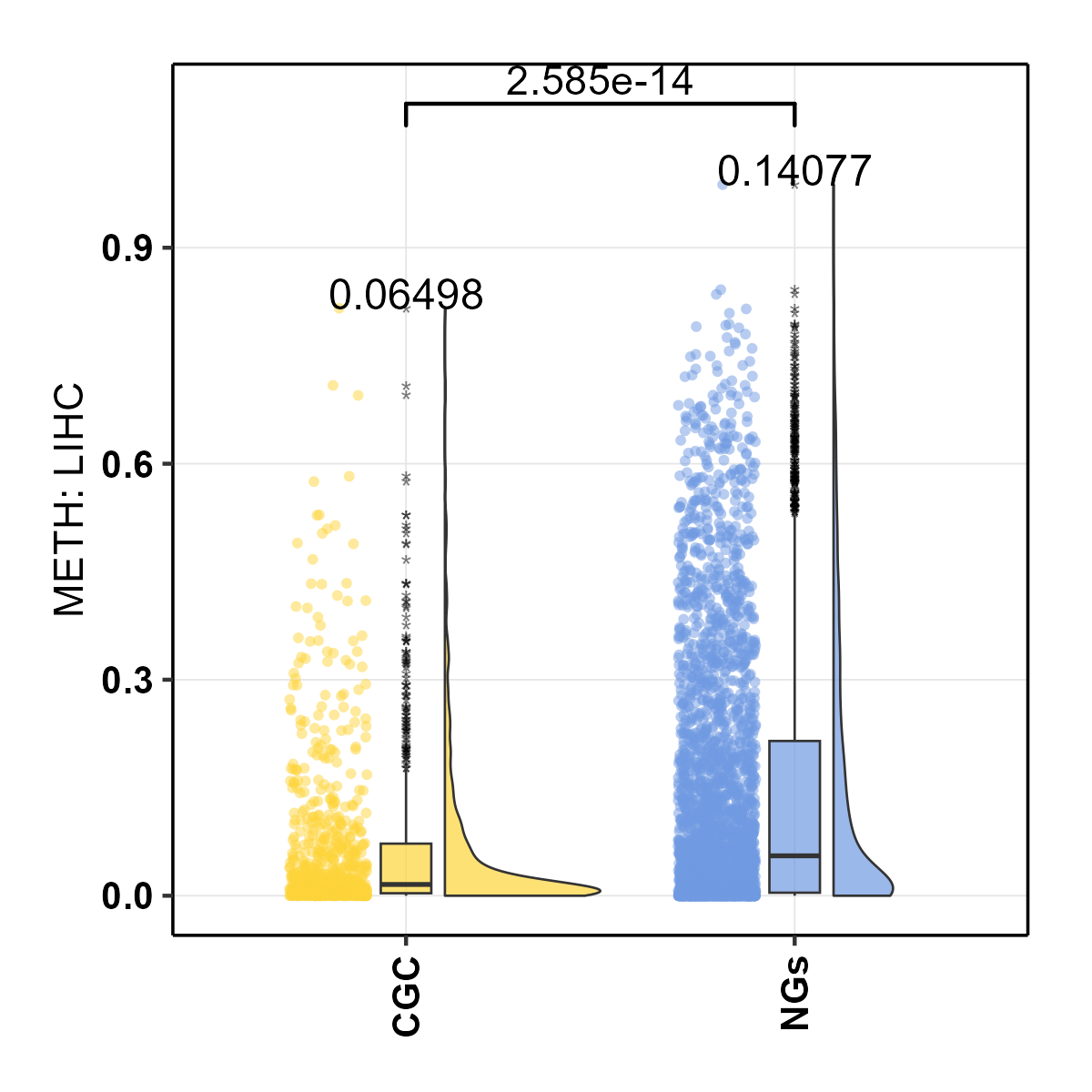

Supplement: Supplementary file 5 [file DataSheet2.ZIP › Supplementary file 5-2/PCNet/METH_LIHC.png]

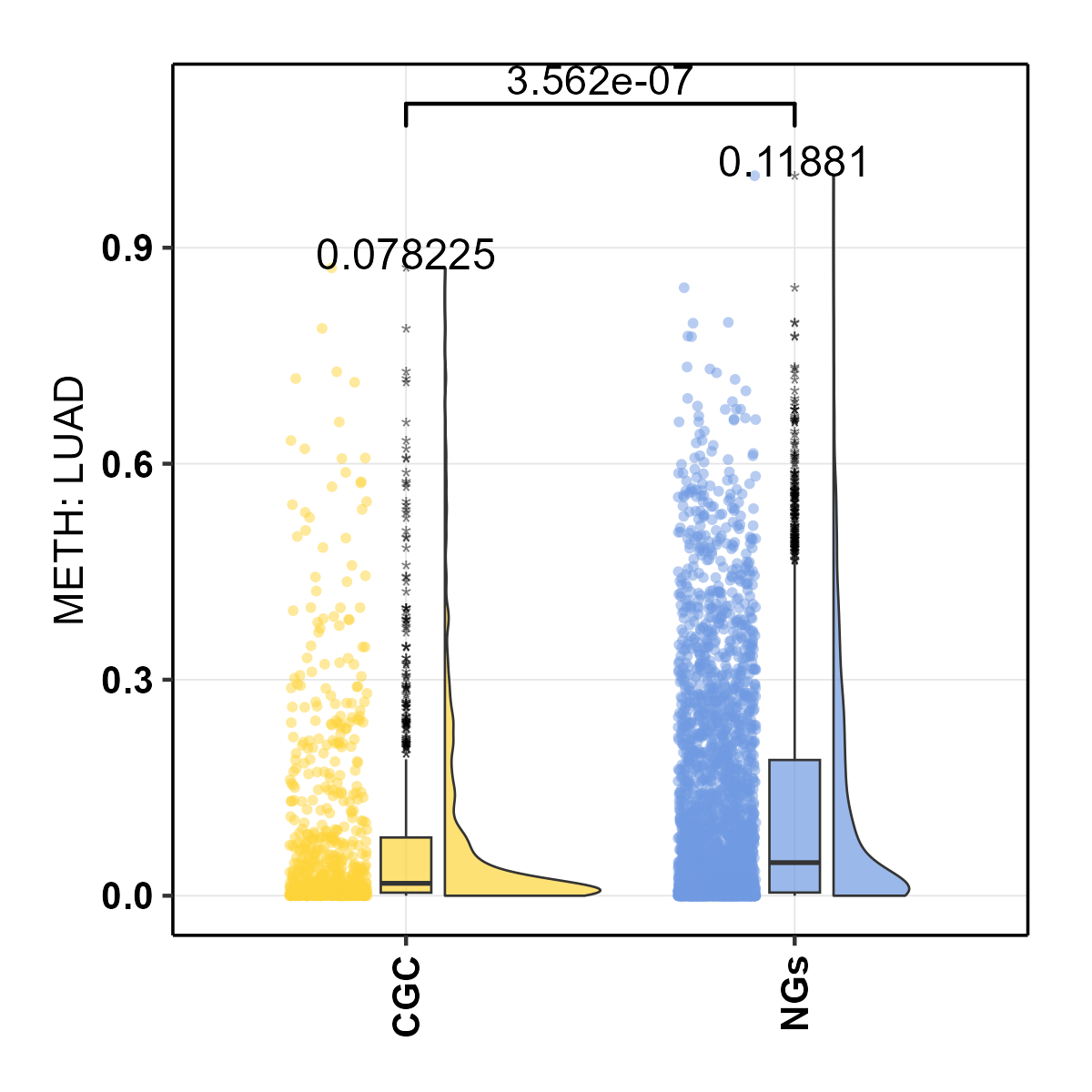

Supplement: Supplementary file 5 [file DataSheet2.ZIP › Supplementary file 5-2/PCNet/METH_LUAD.png]

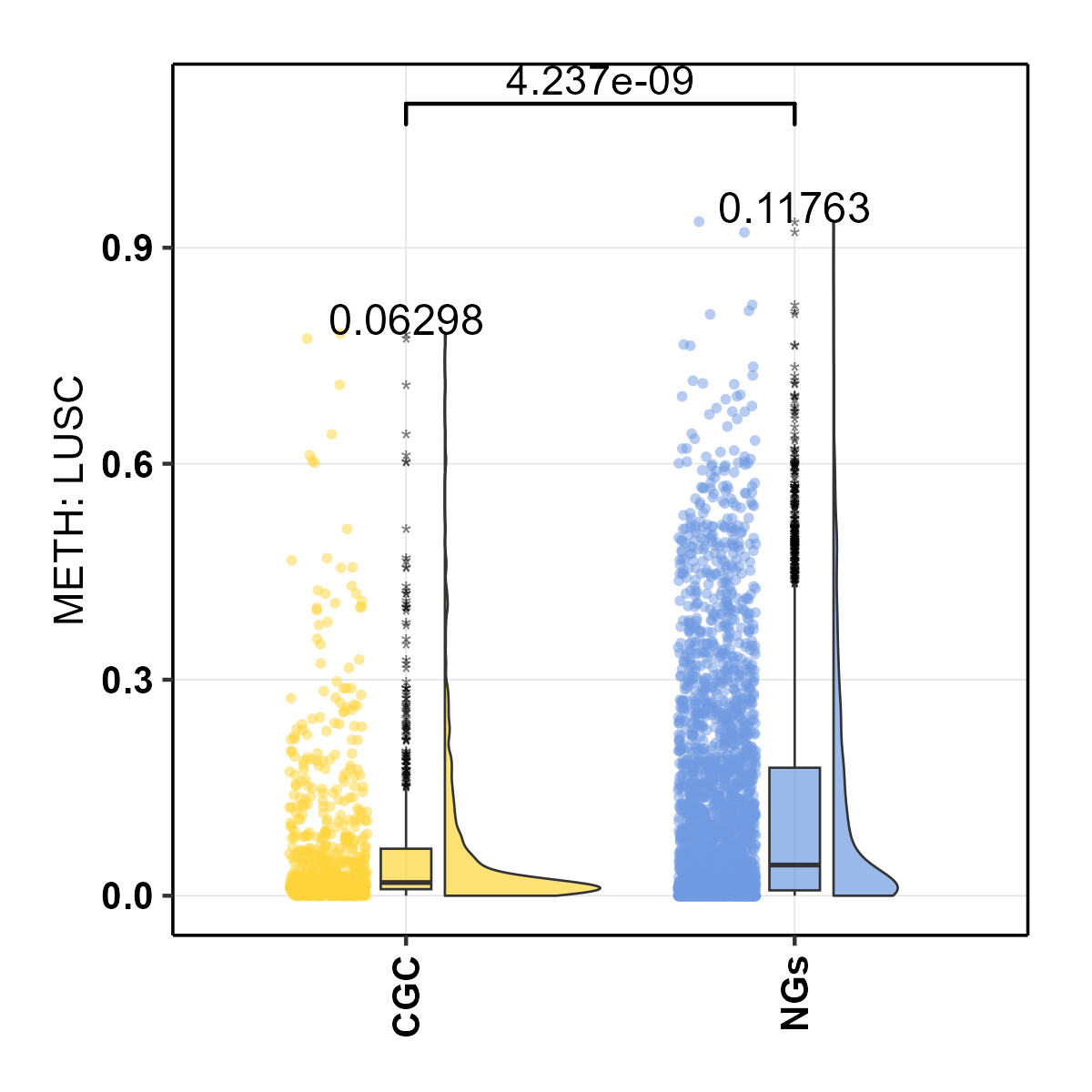

Supplement: Supplementary file 5 [file DataSheet2.ZIP › Supplementary file 5-2/PCNet/METH_LUSC.png]

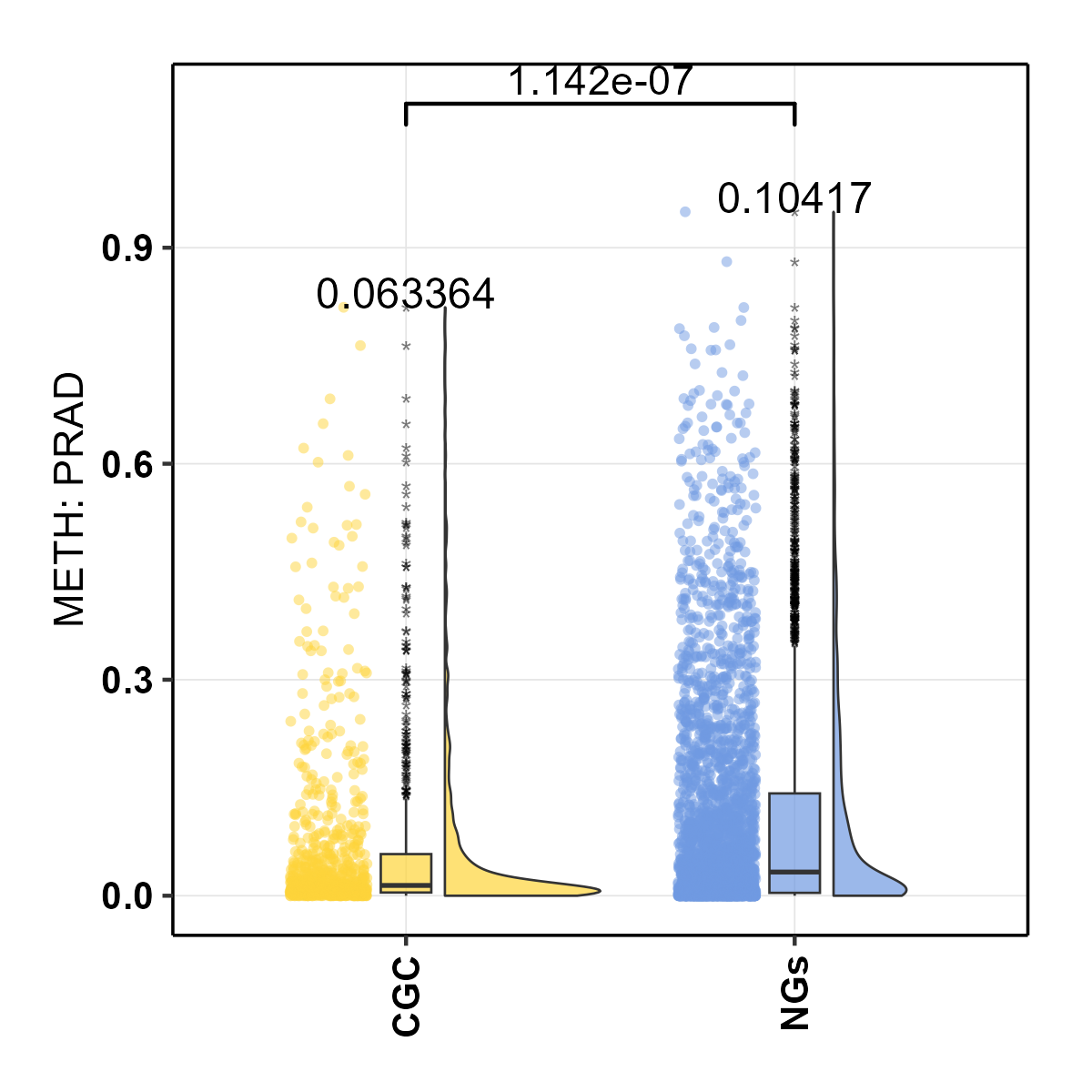

Supplement: Supplementary file 5 [file DataSheet2.ZIP › Supplementary file 5-2/PCNet/METH_PRAD.png]

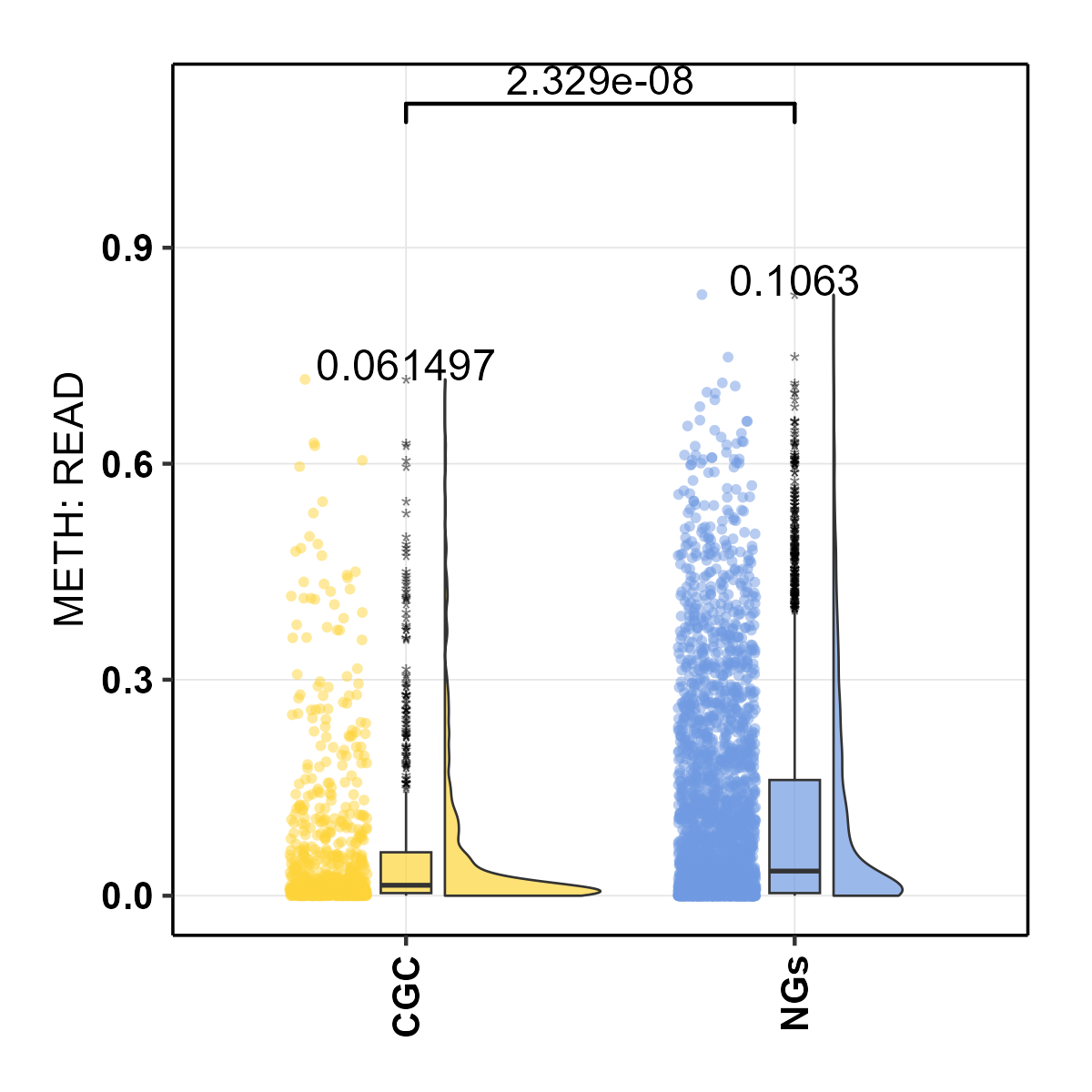

Supplement: Supplementary file 5 [file DataSheet2.ZIP › Supplementary file 5-2/PCNet/METH_READ.png]

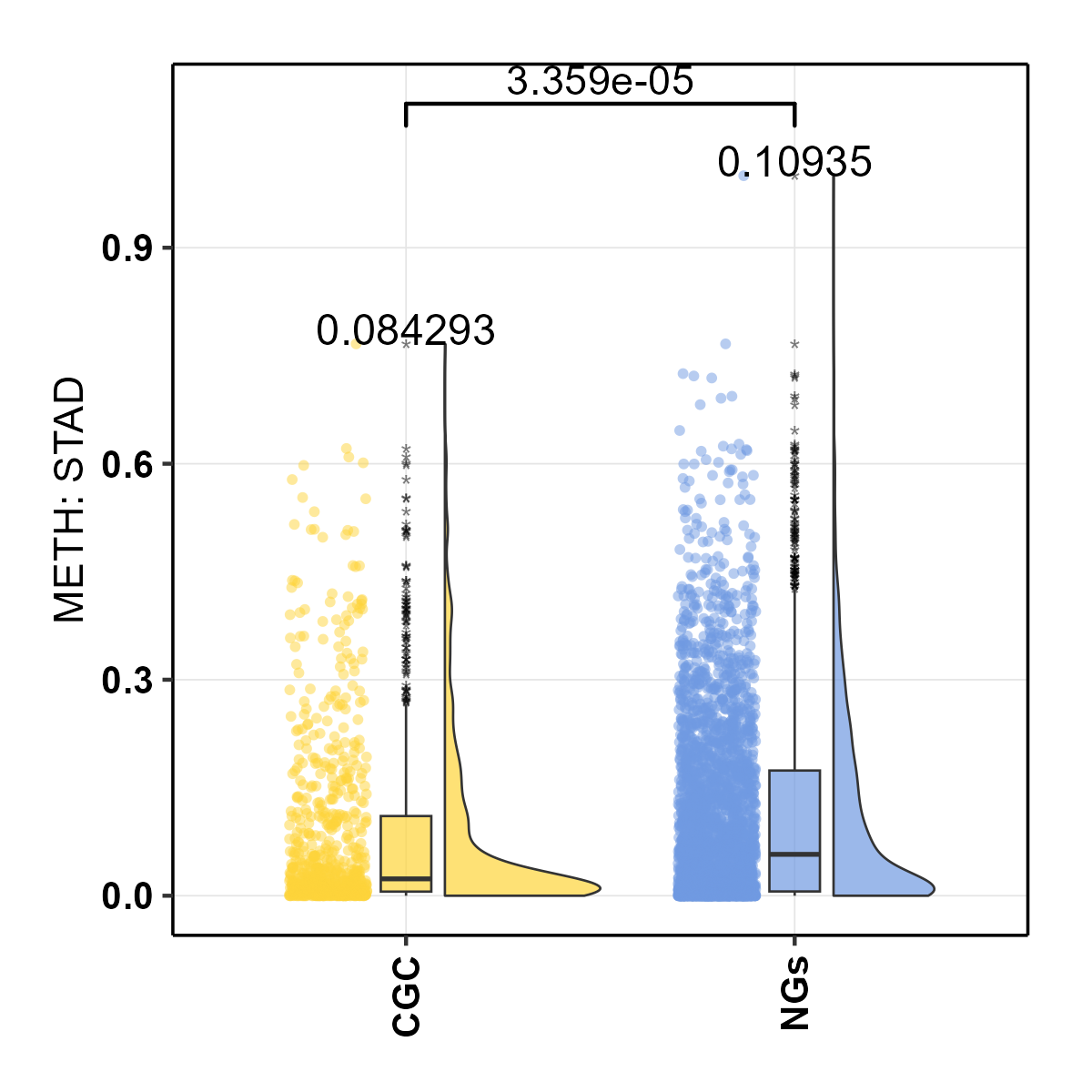

Supplement: Supplementary file 5 [file DataSheet2.ZIP › Supplementary file 5-2/PCNet/METH_STAD.png]

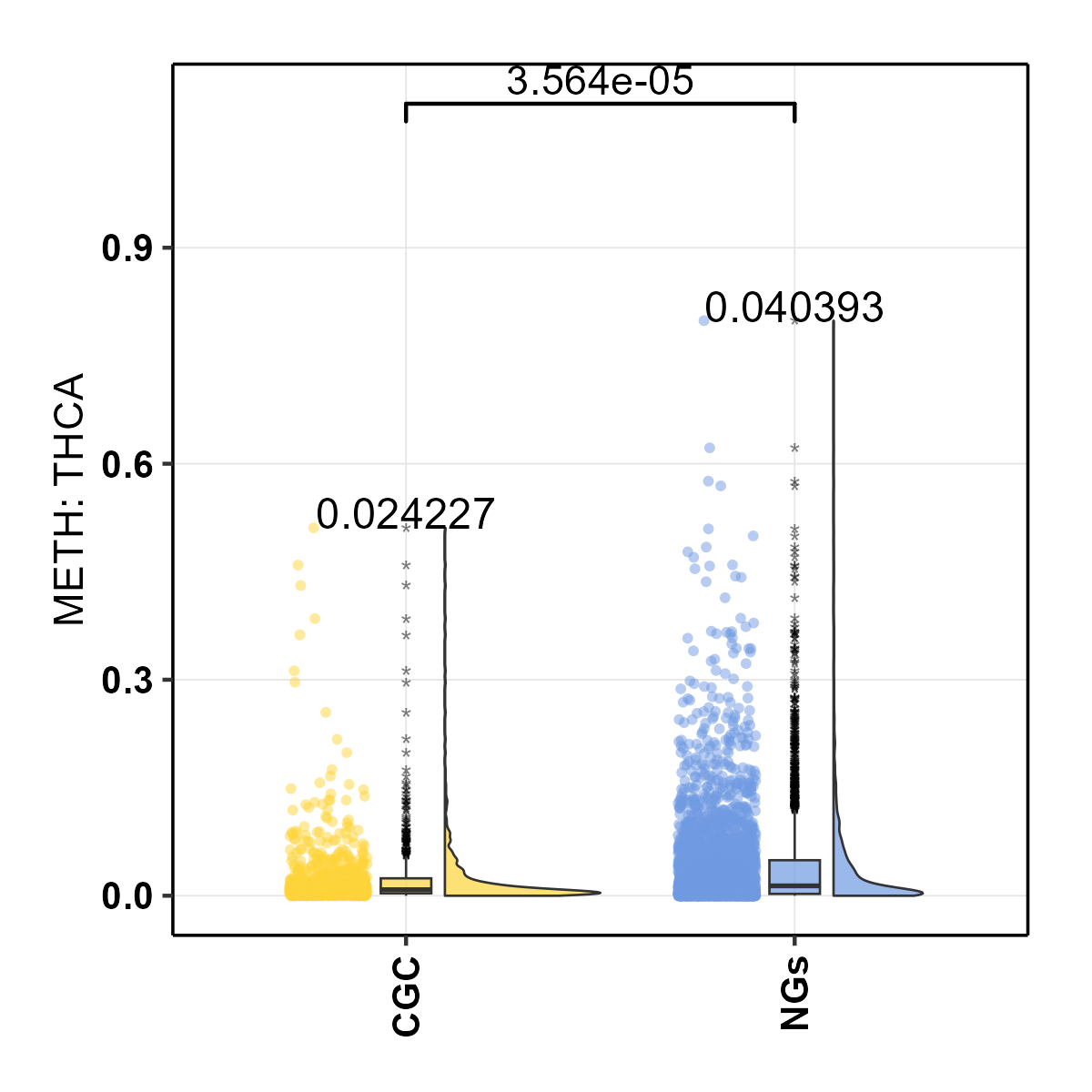

Supplement: Supplementary file 5 [file DataSheet2.ZIP › Supplementary file 5-2/PCNet/METH_THCA.png]

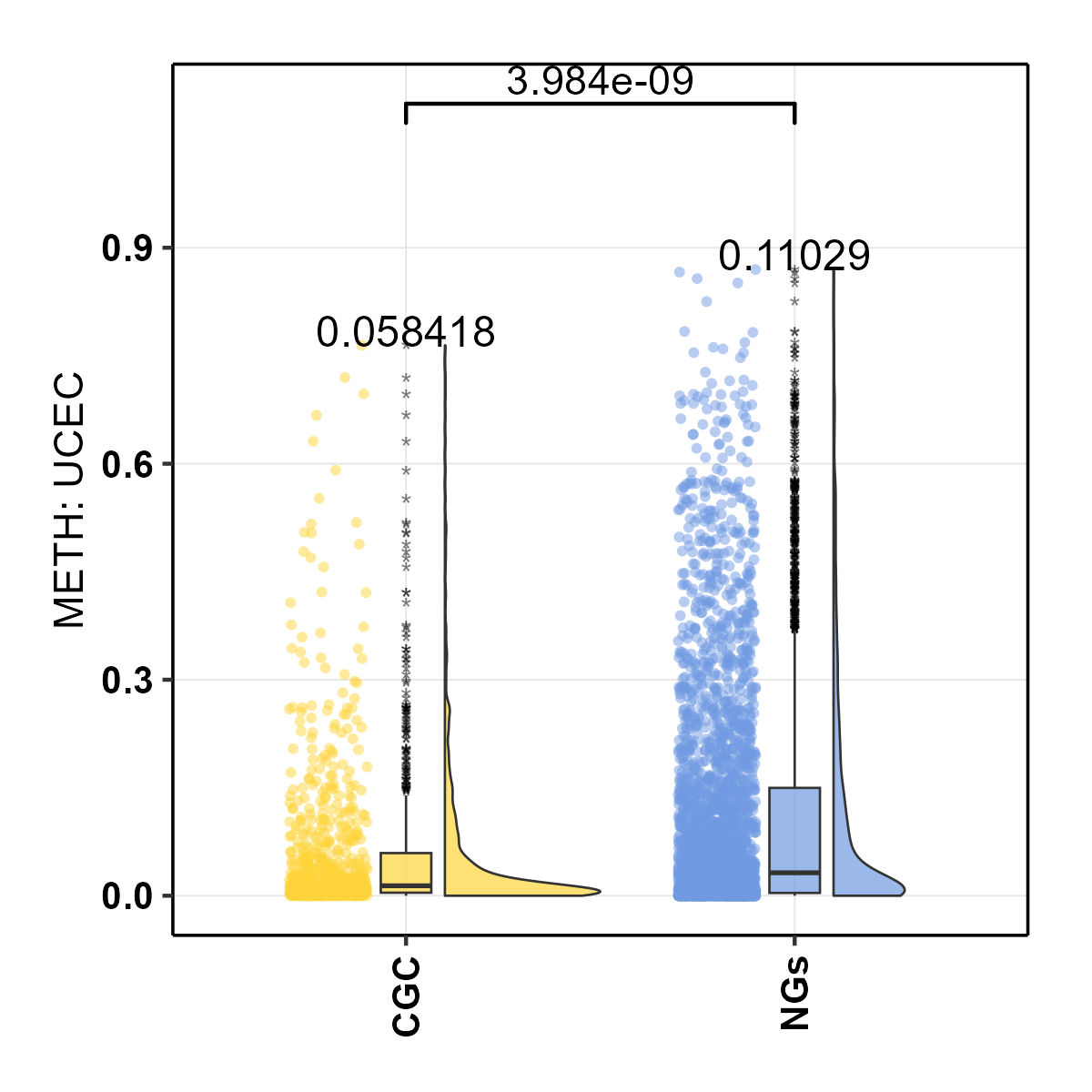

Supplement: Supplementary file 5 [file DataSheet2.ZIP › Supplementary file 5-2/PCNet/METH_UCEC.png]

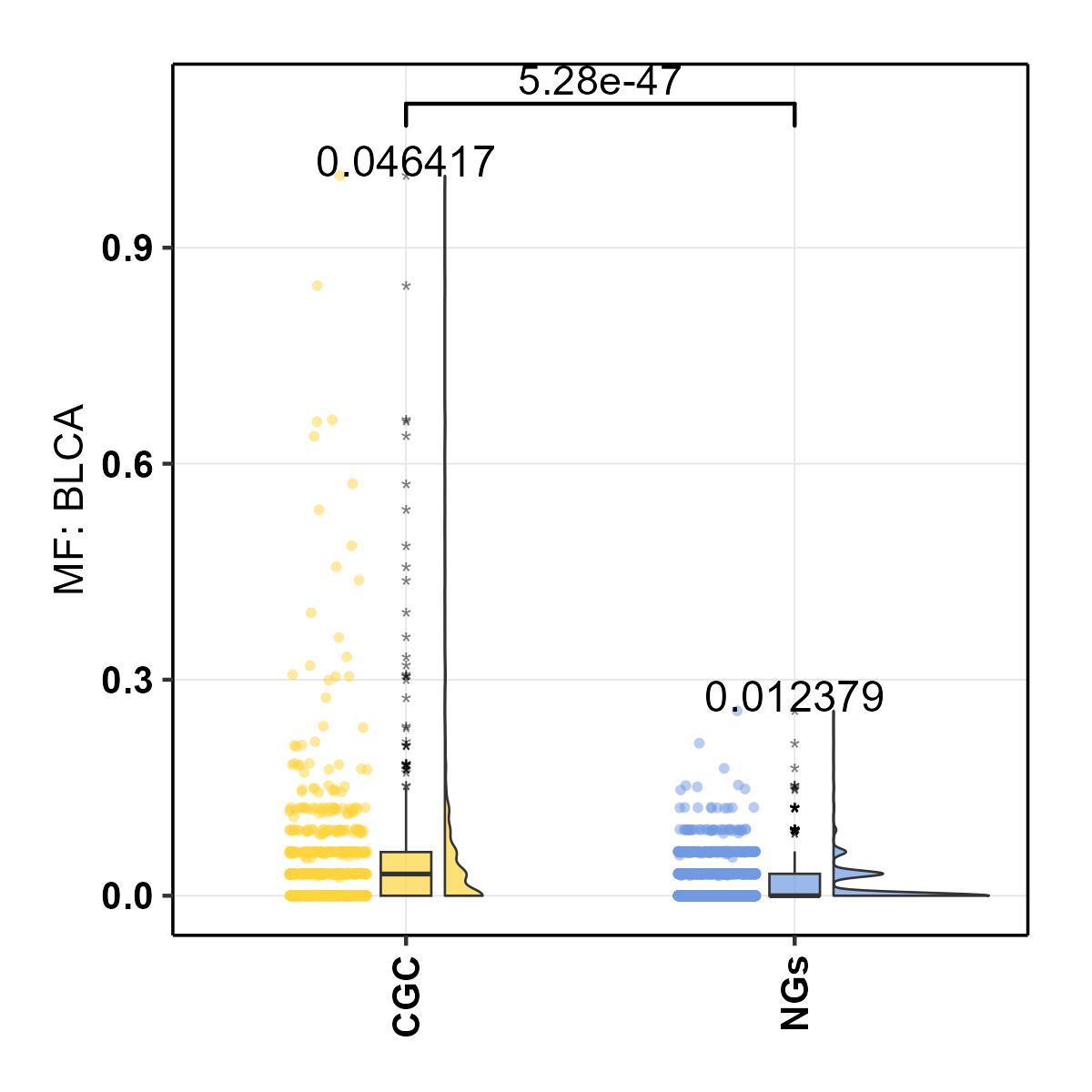

Supplement: Supplementary file 5 [file DataSheet2.ZIP › Supplementary file 5-2/PCNet/MF_BLCA.png]

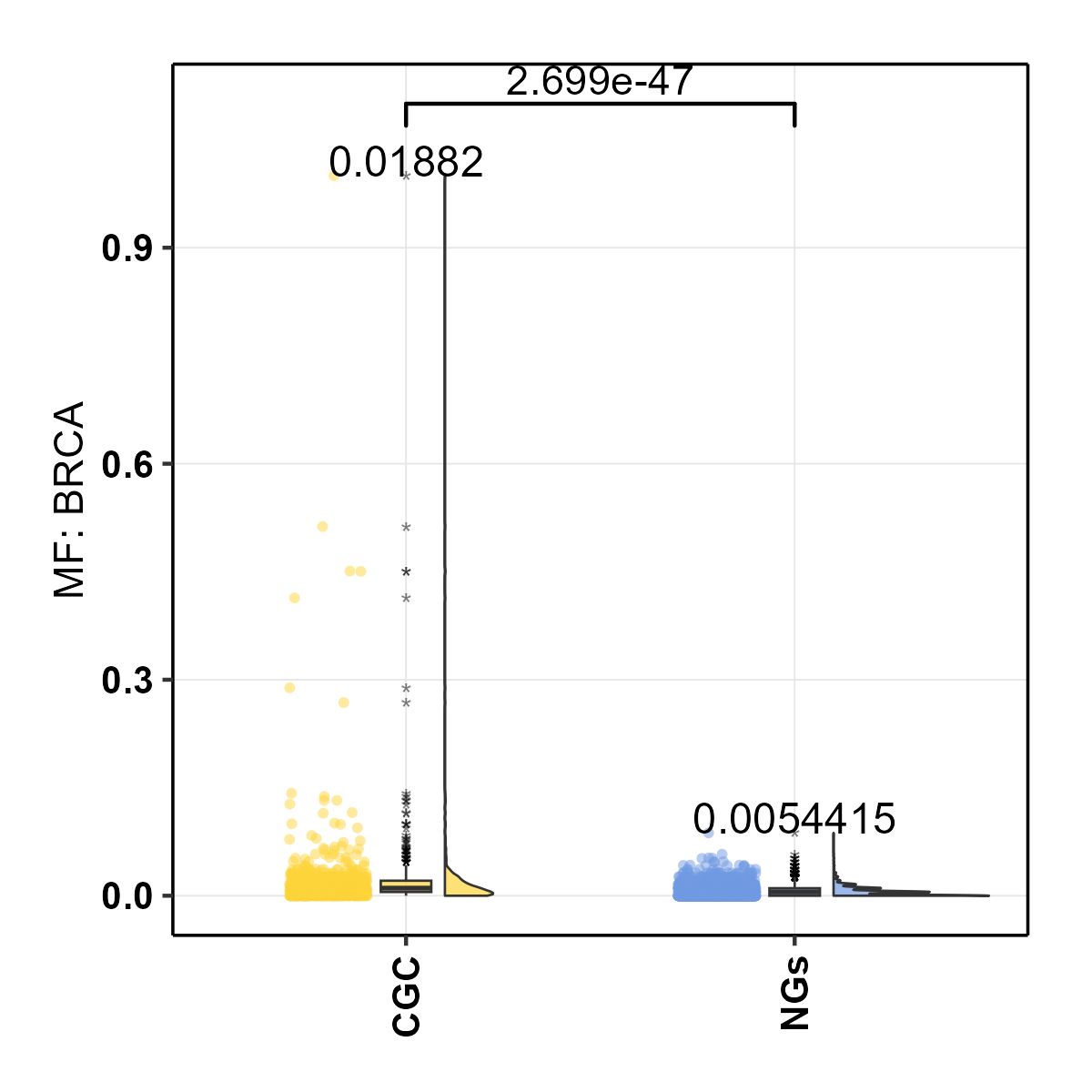

Supplement: Supplementary file 5 [file DataSheet2.ZIP › Supplementary file 5-2/PCNet/MF_BRCA.png]

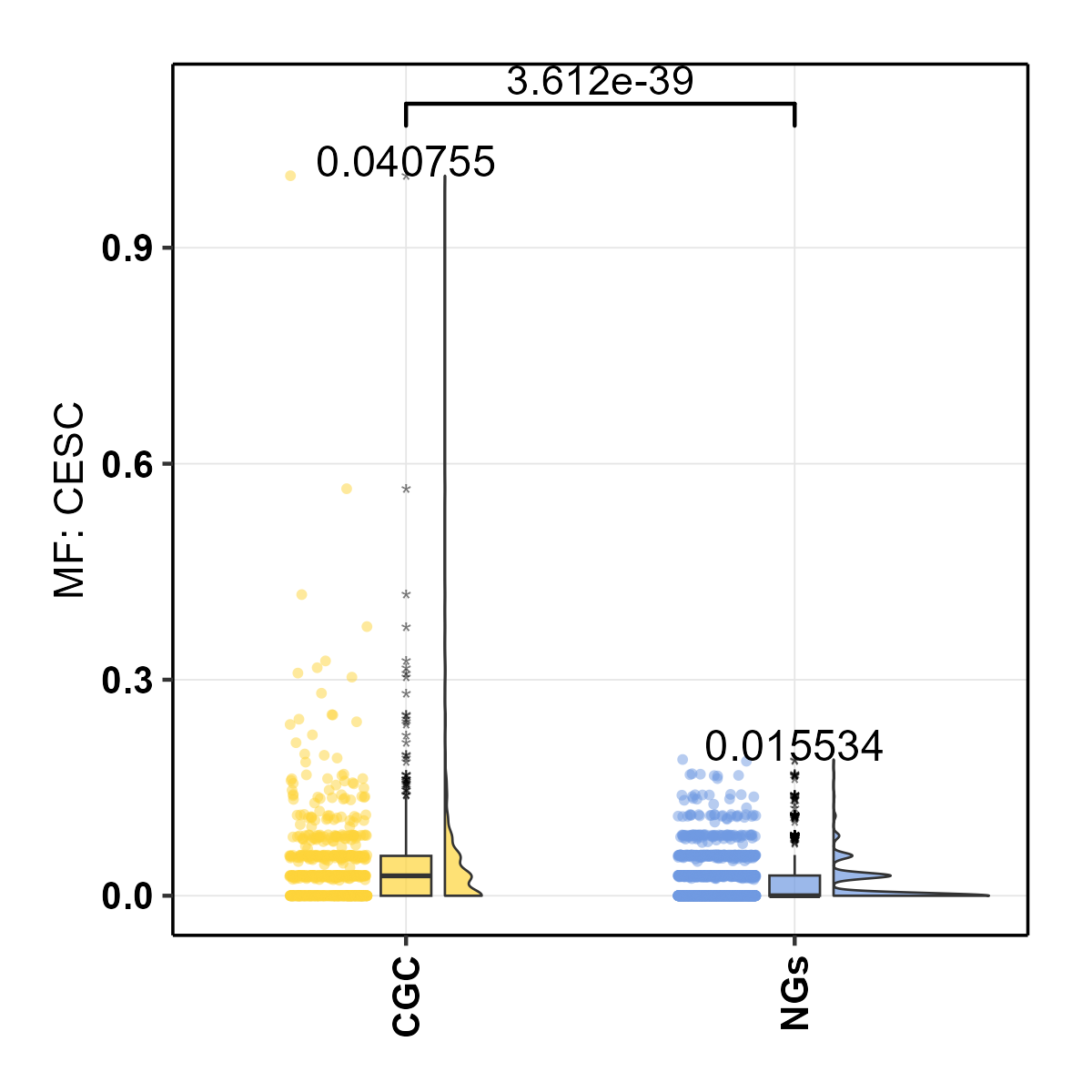

Supplement: Supplementary file 5 [file DataSheet2.ZIP › Supplementary file 5-2/PCNet/MF_CESC.png]

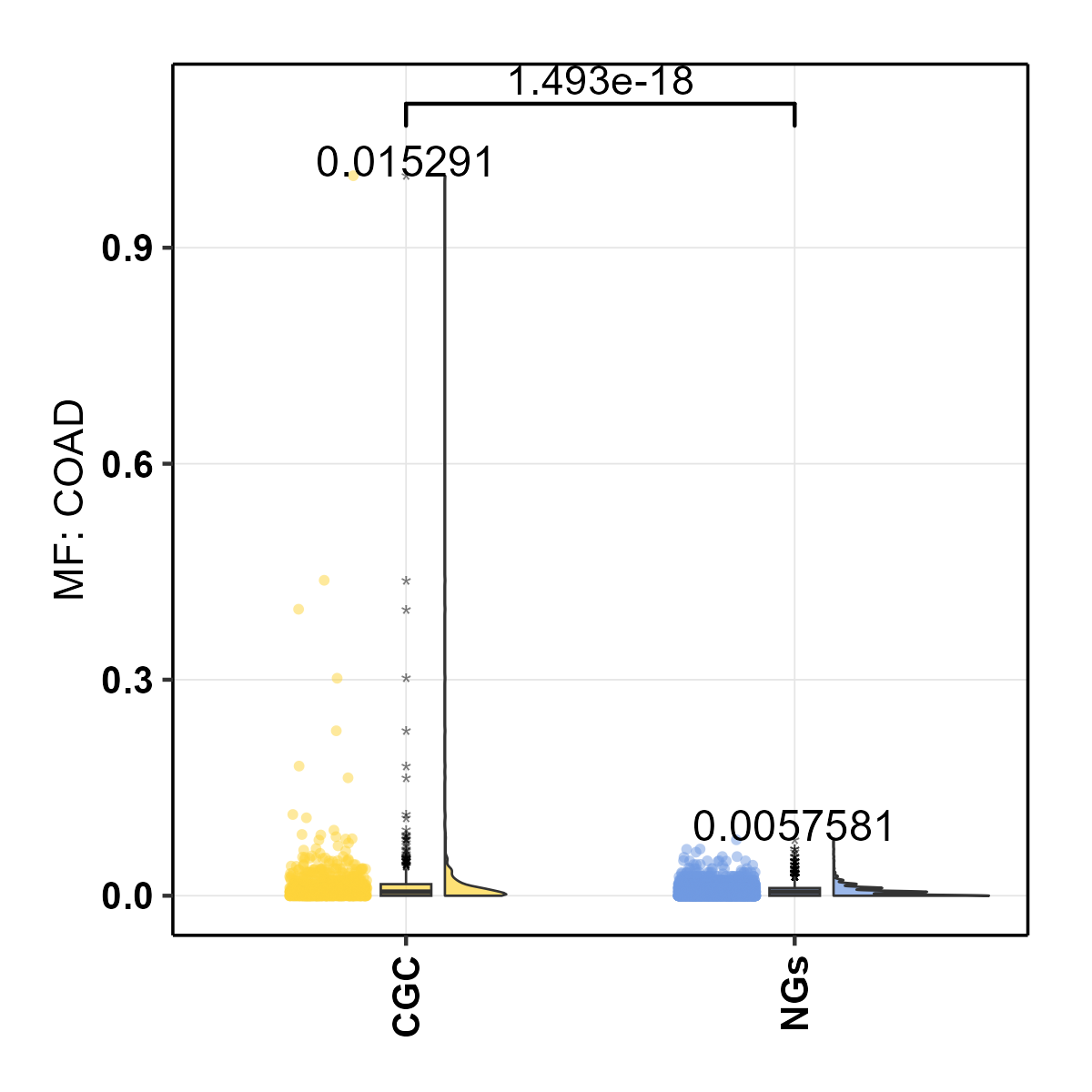

Supplement: Supplementary file 5 [file DataSheet2.ZIP › Supplementary file 5-2/PCNet/MF_COAD.png]

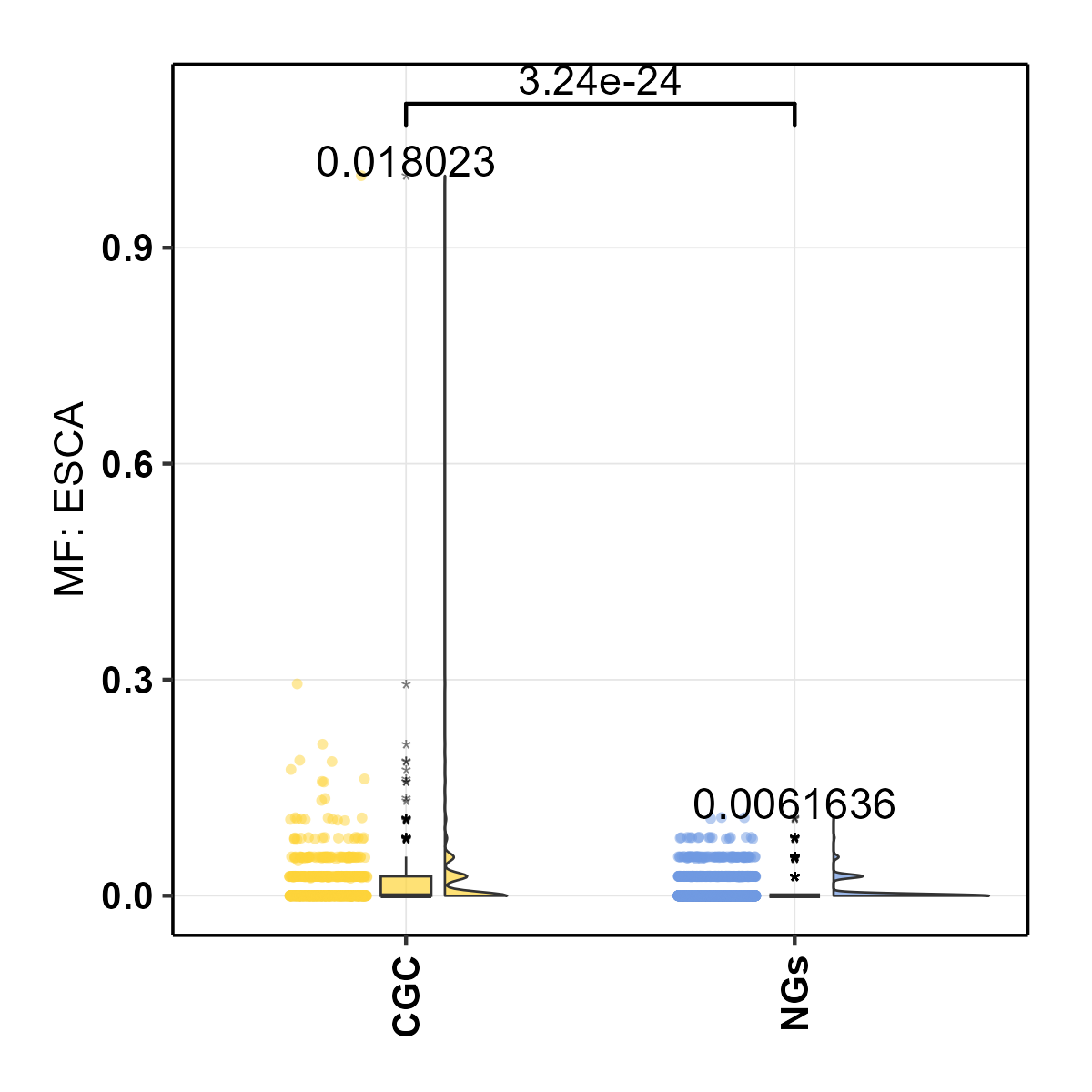

Supplement: Supplementary file 5 [file DataSheet2.ZIP › Supplementary file 5-2/PCNet/MF_ESCA.png]

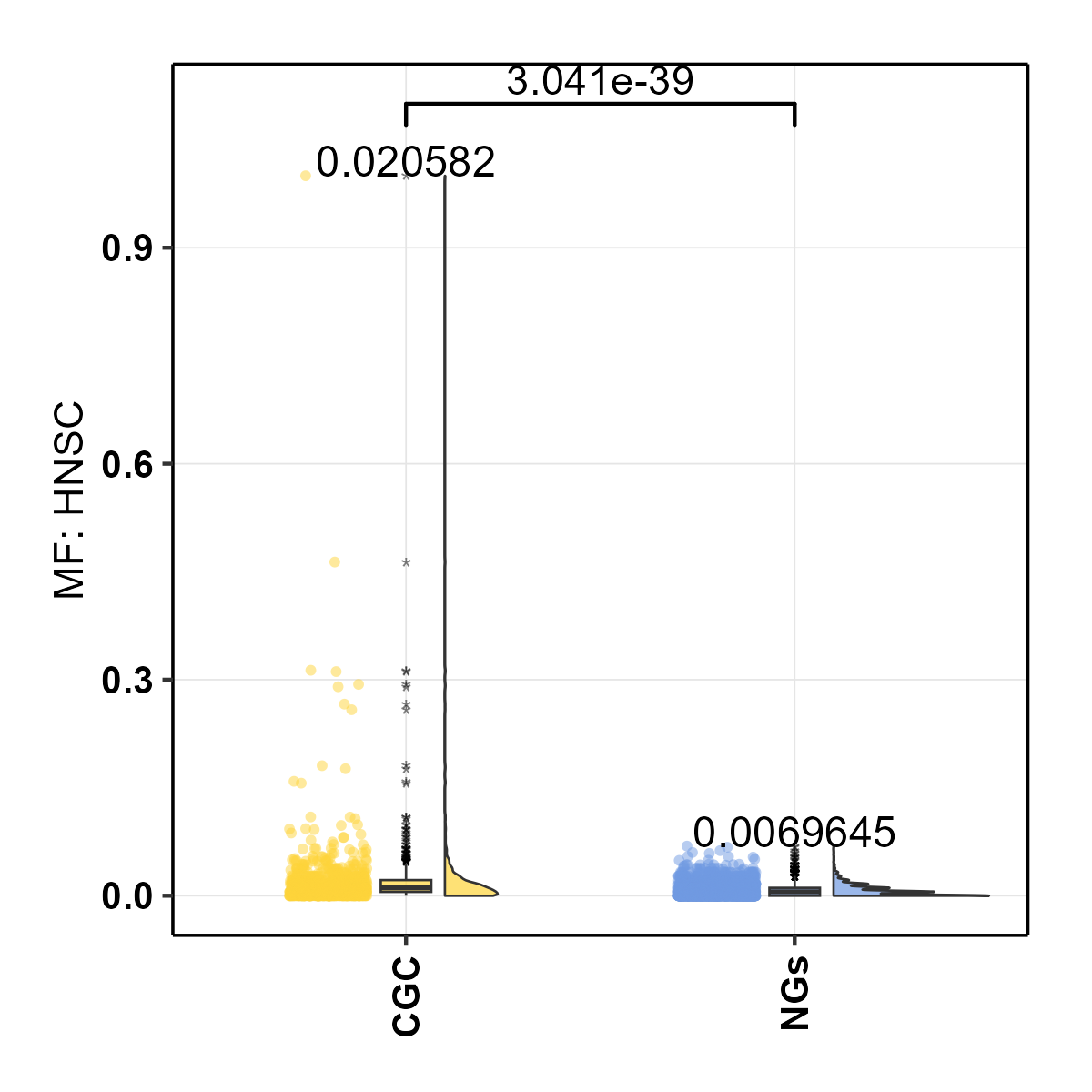

Supplement: Supplementary file 5 [file DataSheet2.ZIP › Supplementary file 5-2/PCNet/MF_HNSC.png]

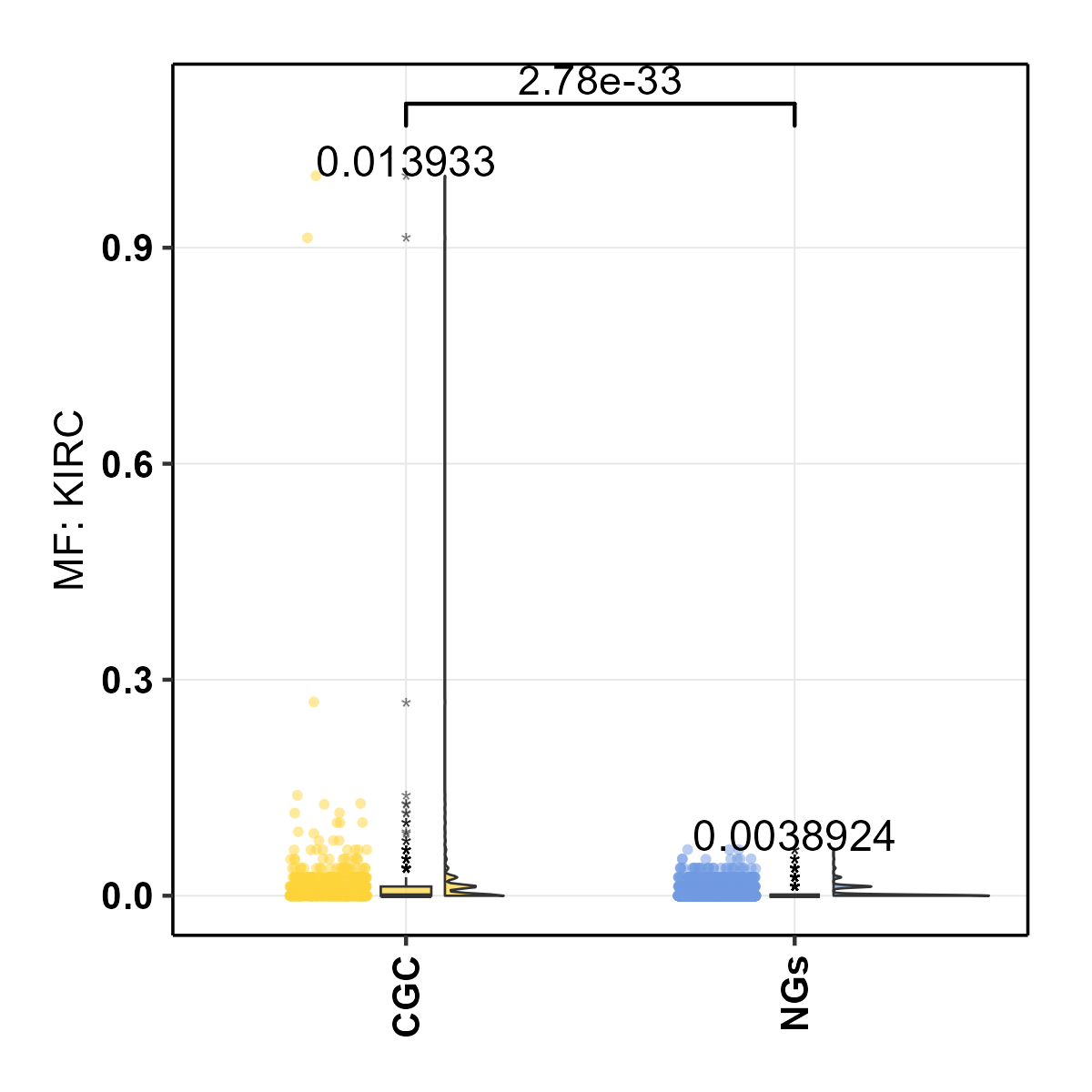

Supplement: Supplementary file 5 [file DataSheet2.ZIP › Supplementary file 5-2/PCNet/MF_KIRC.png]

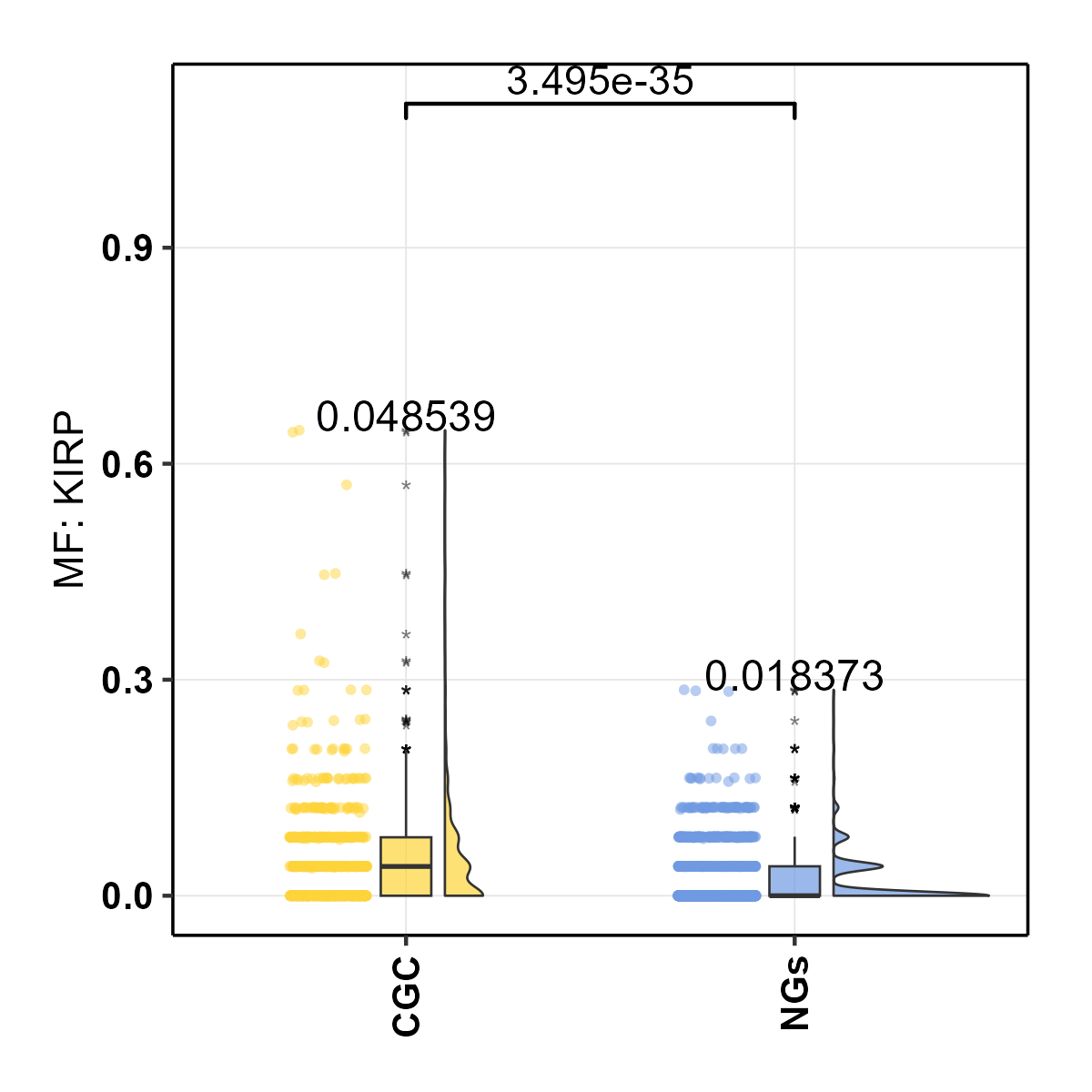

Supplement: Supplementary file 5 [file DataSheet2.ZIP › Supplementary file 5-2/PCNet/MF_KIRP.png]

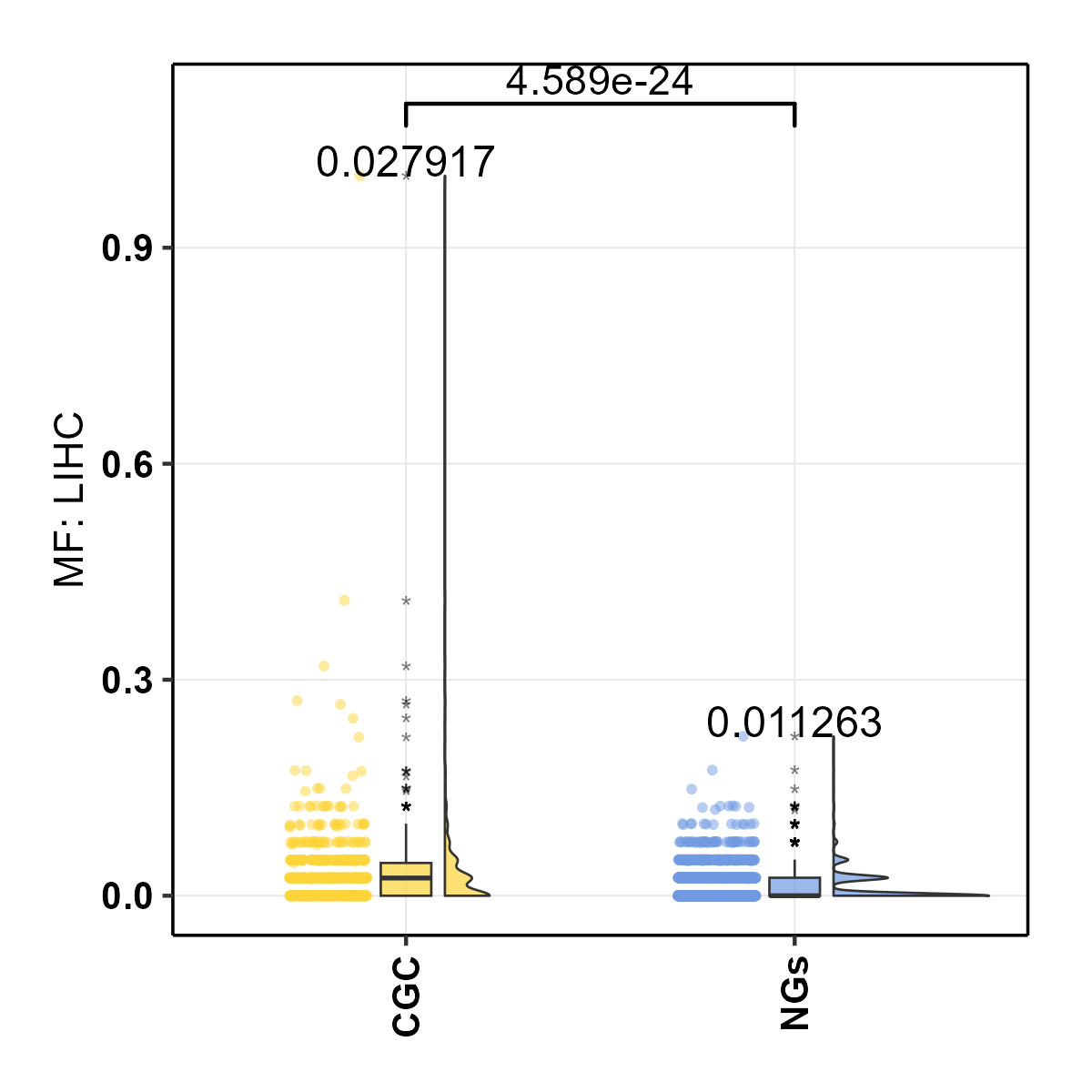

Supplement: Supplementary file 5 [file DataSheet2.ZIP › Supplementary file 5-2/PCNet/MF_LIHC.png]

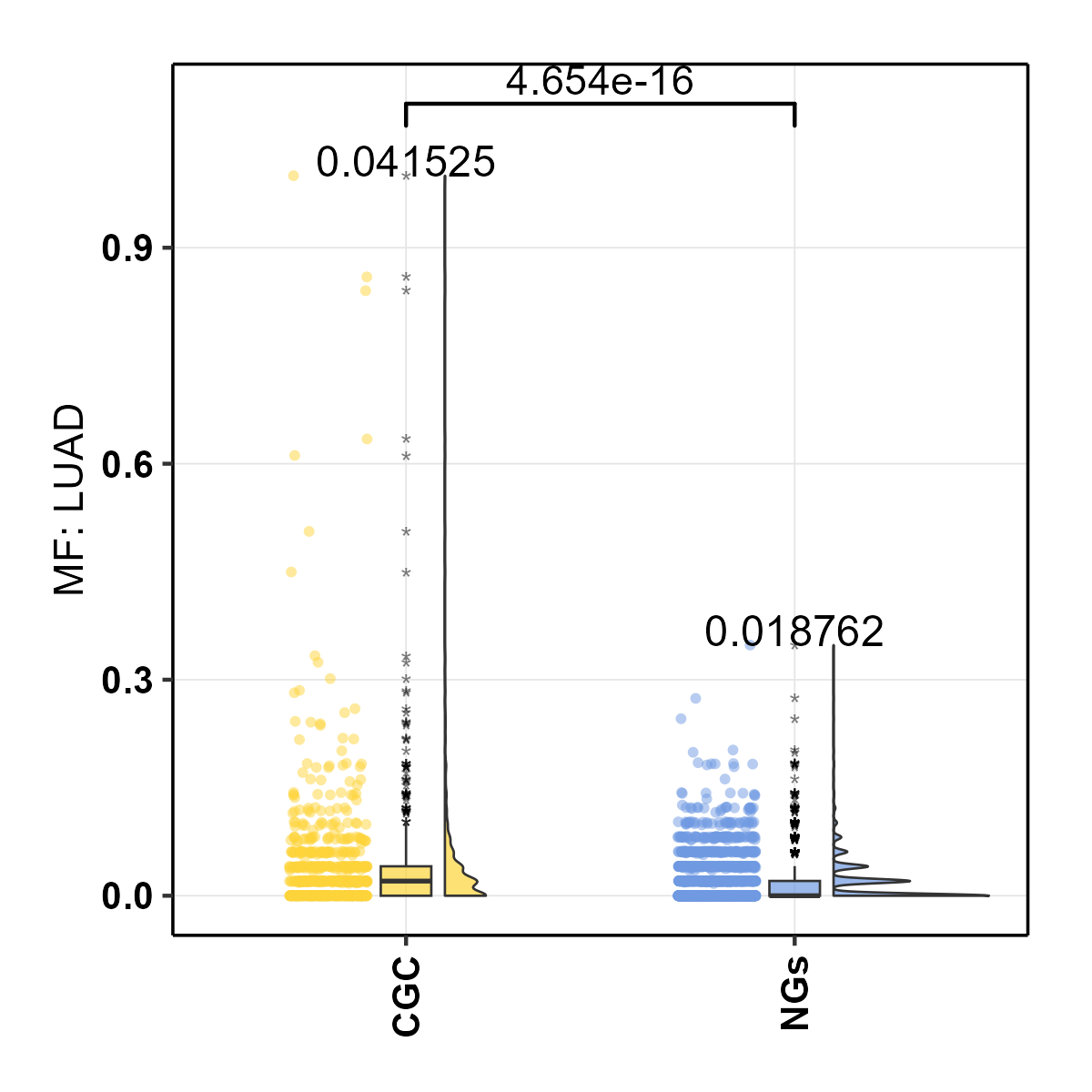

Supplement: Supplementary file 5 [file DataSheet2.ZIP › Supplementary file 5-2/PCNet/MF_LUAD.png]

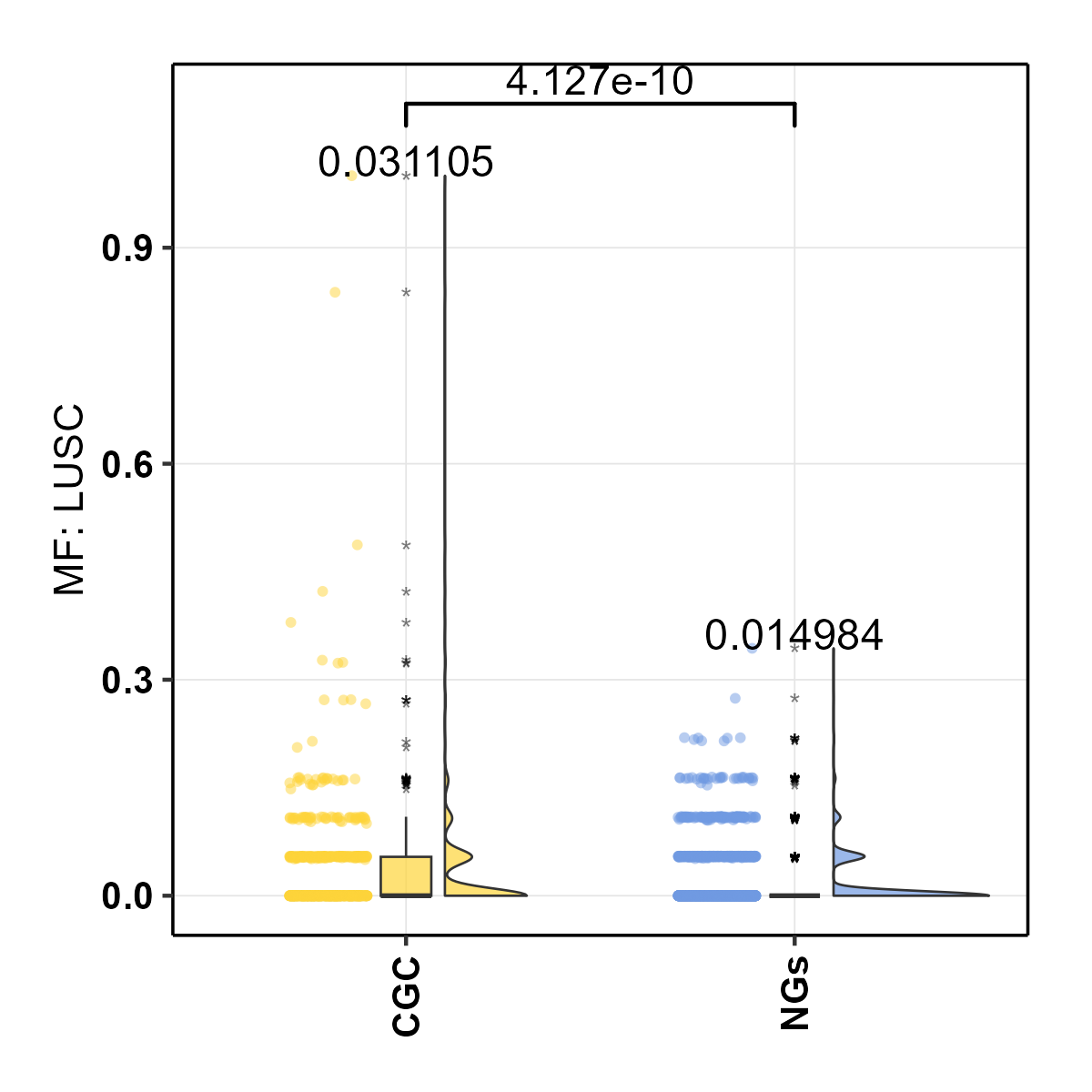

Supplement: Supplementary file 5 [file DataSheet2.ZIP › Supplementary file 5-2/PCNet/MF_LUSC.png]

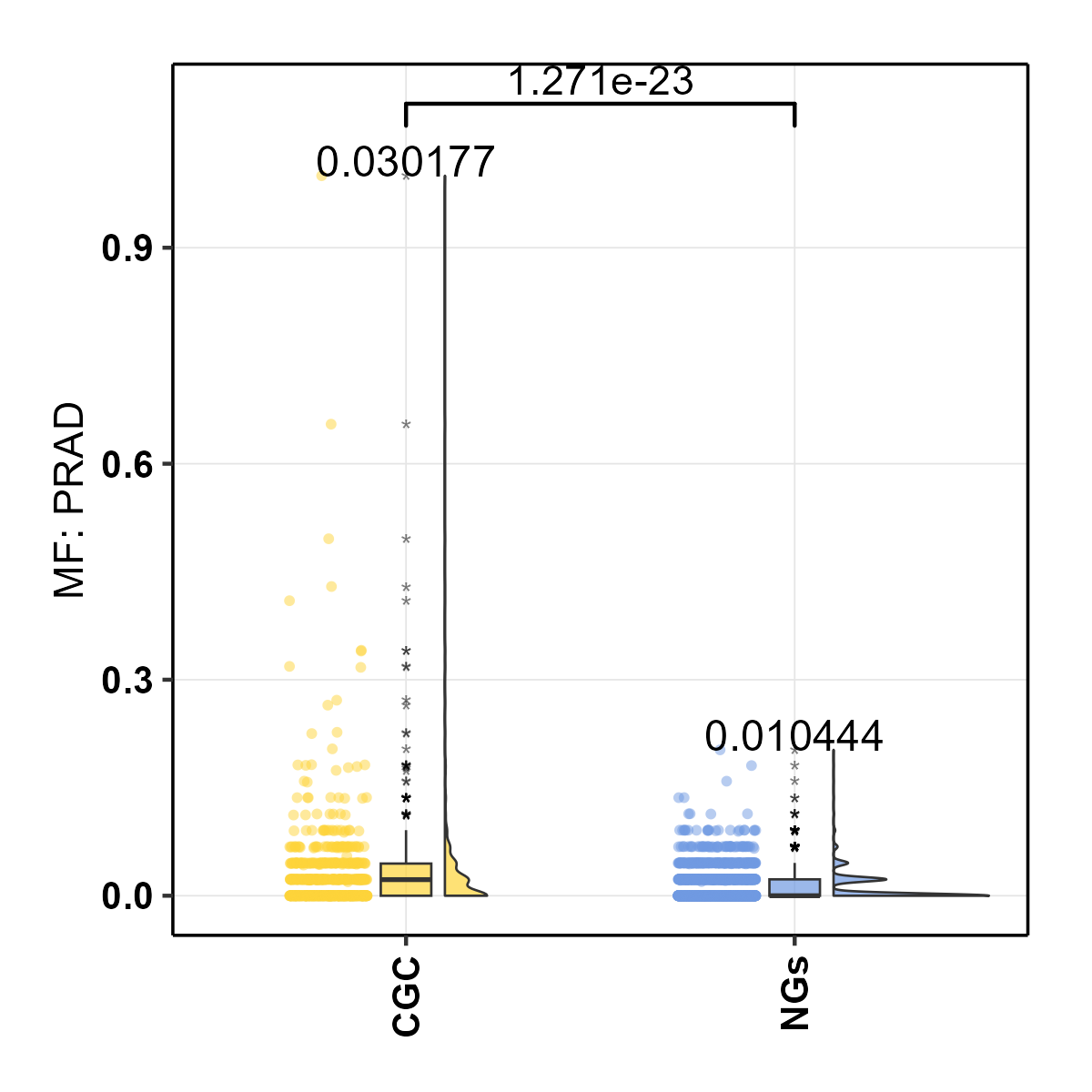

Supplement: Supplementary file 5 [file DataSheet2.ZIP › Supplementary file 5-2/PCNet/MF_PRAD.png]

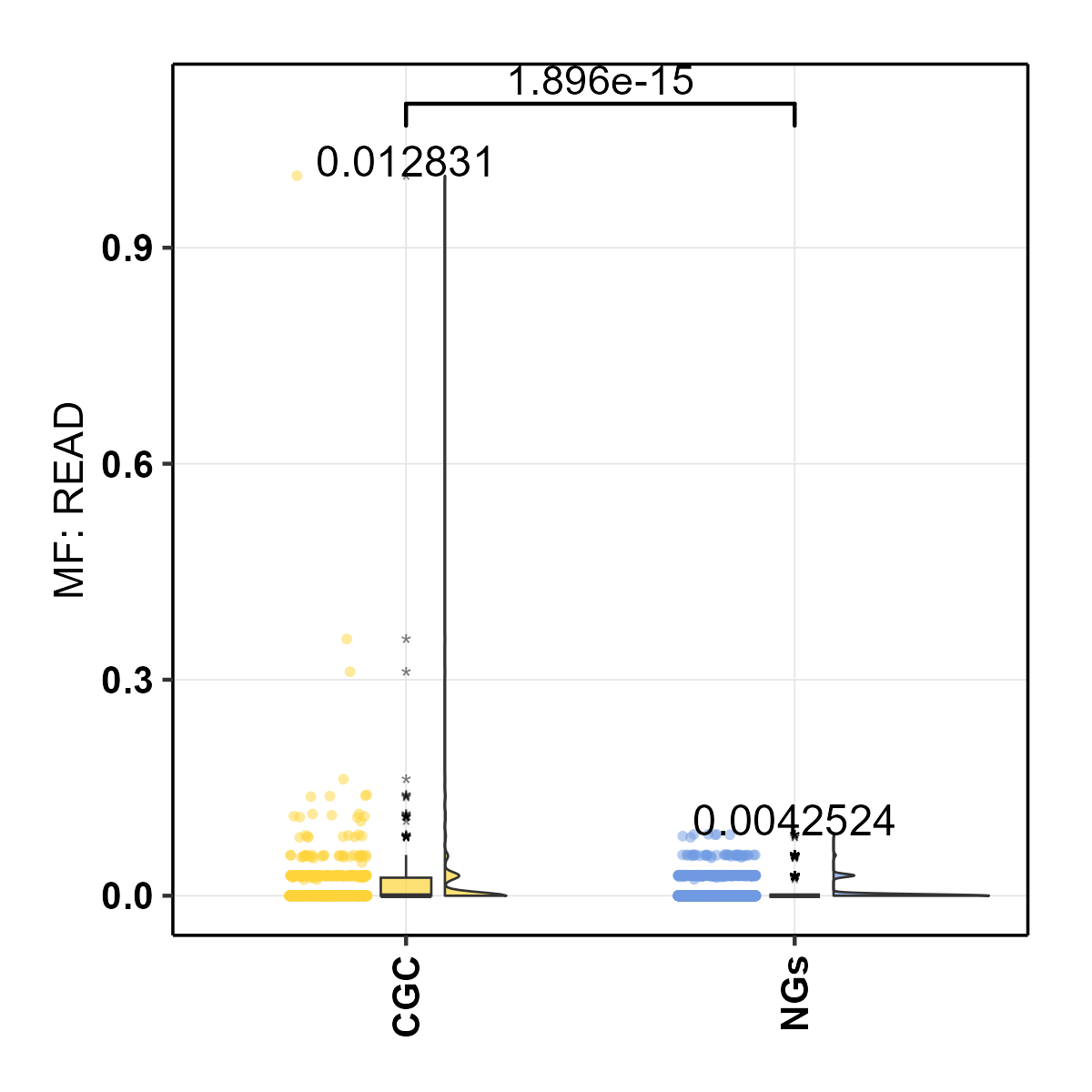

Supplement: Supplementary file 5 [file DataSheet2.ZIP › Supplementary file 5-2/PCNet/MF_READ.png]

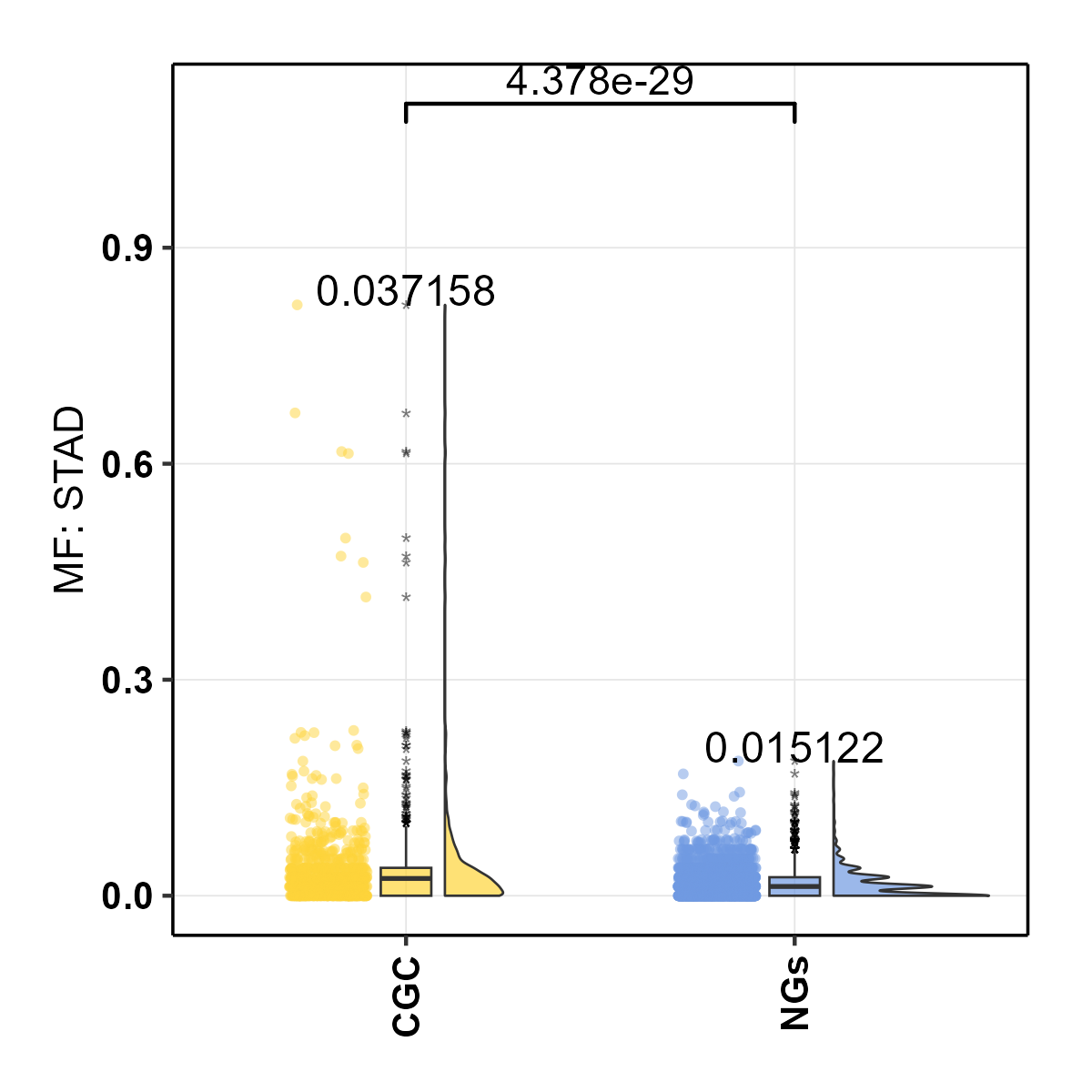

Supplement: Supplementary file 5 [file DataSheet2.ZIP › Supplementary file 5-2/PCNet/MF_STAD.png]

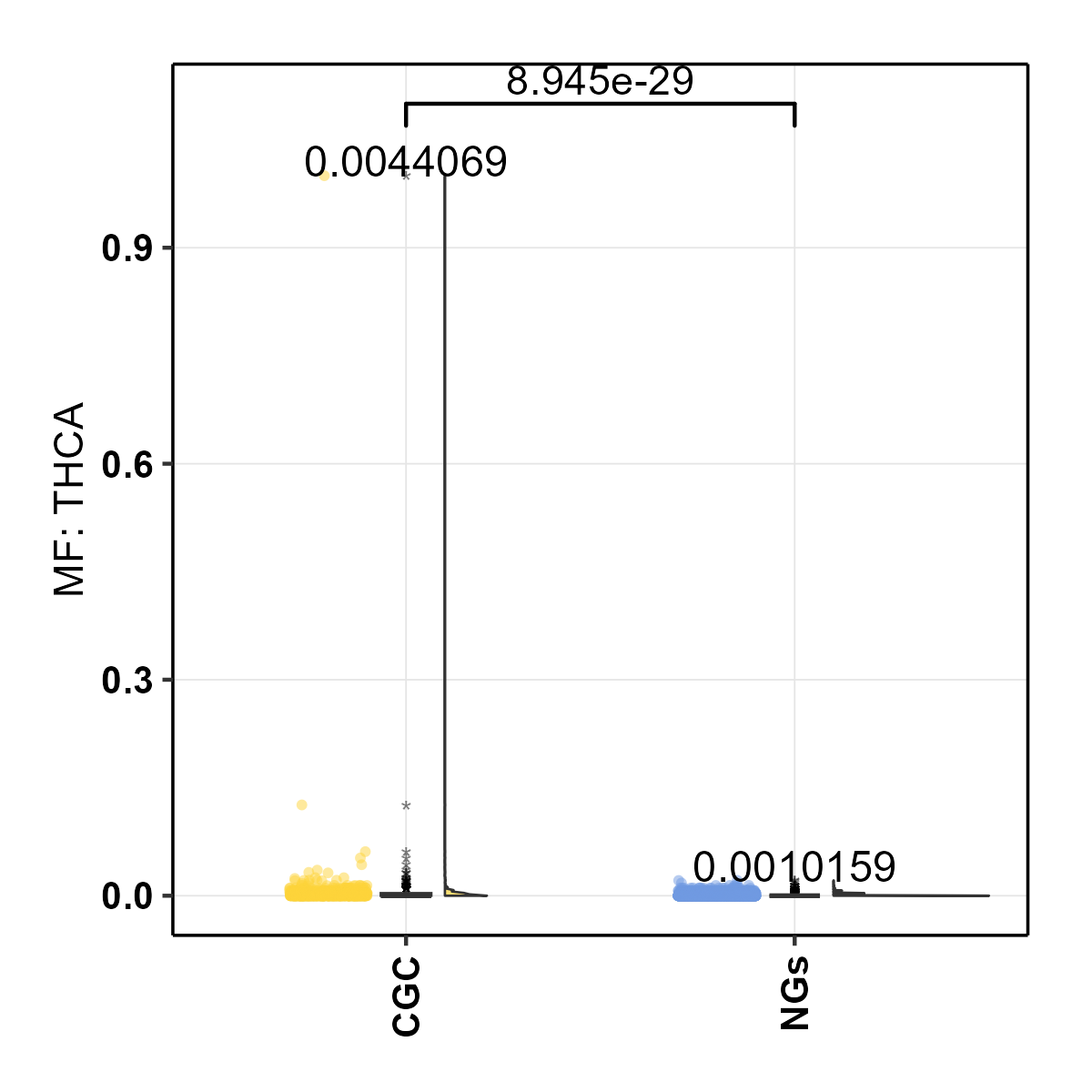

Supplement: Supplementary file 5 [file DataSheet2.ZIP › Supplementary file 5-2/PCNet/MF_THCA.png]

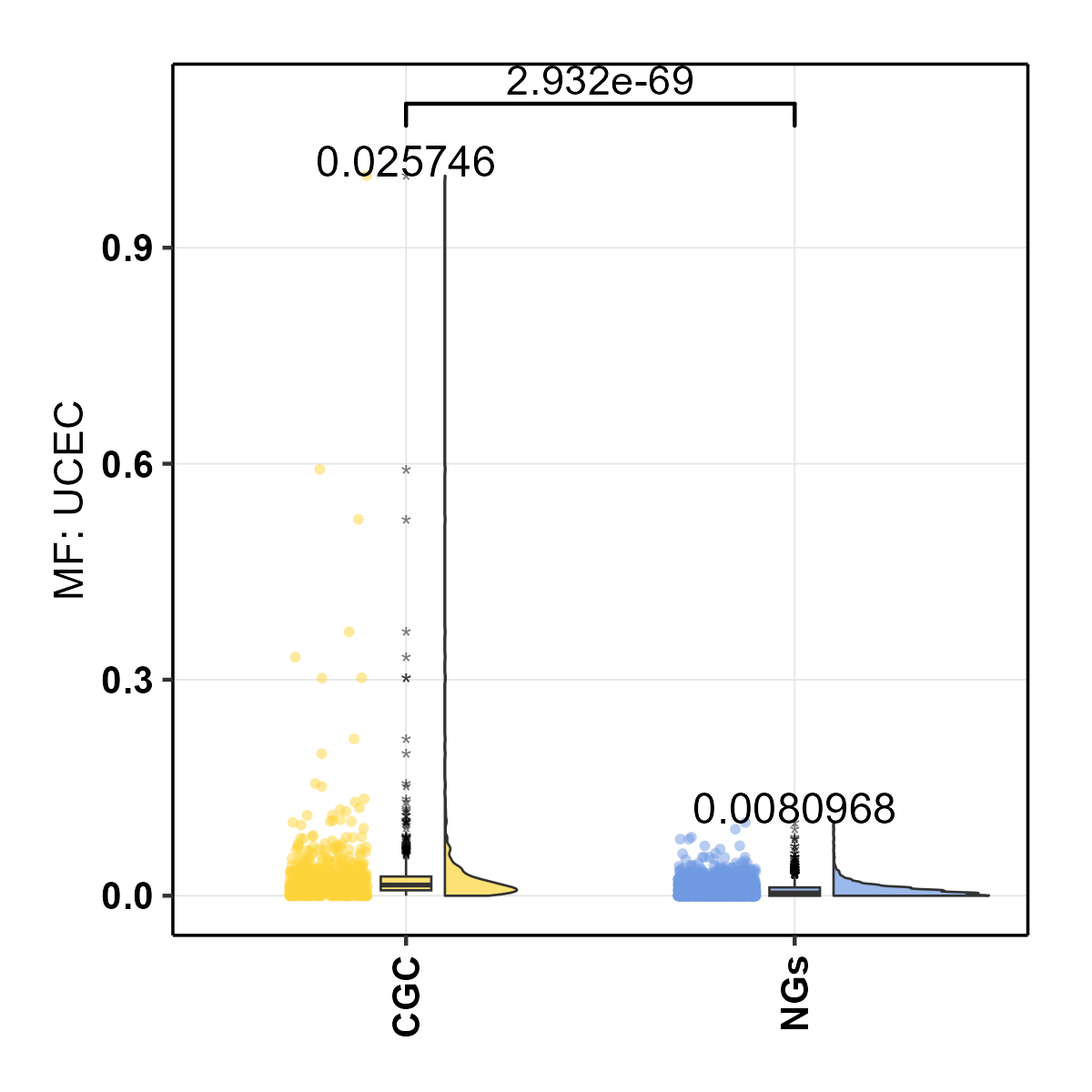

Supplement: Supplementary file 5 [file DataSheet2.ZIP › Supplementary file 5-2/PCNet/MF_UCEC.png]

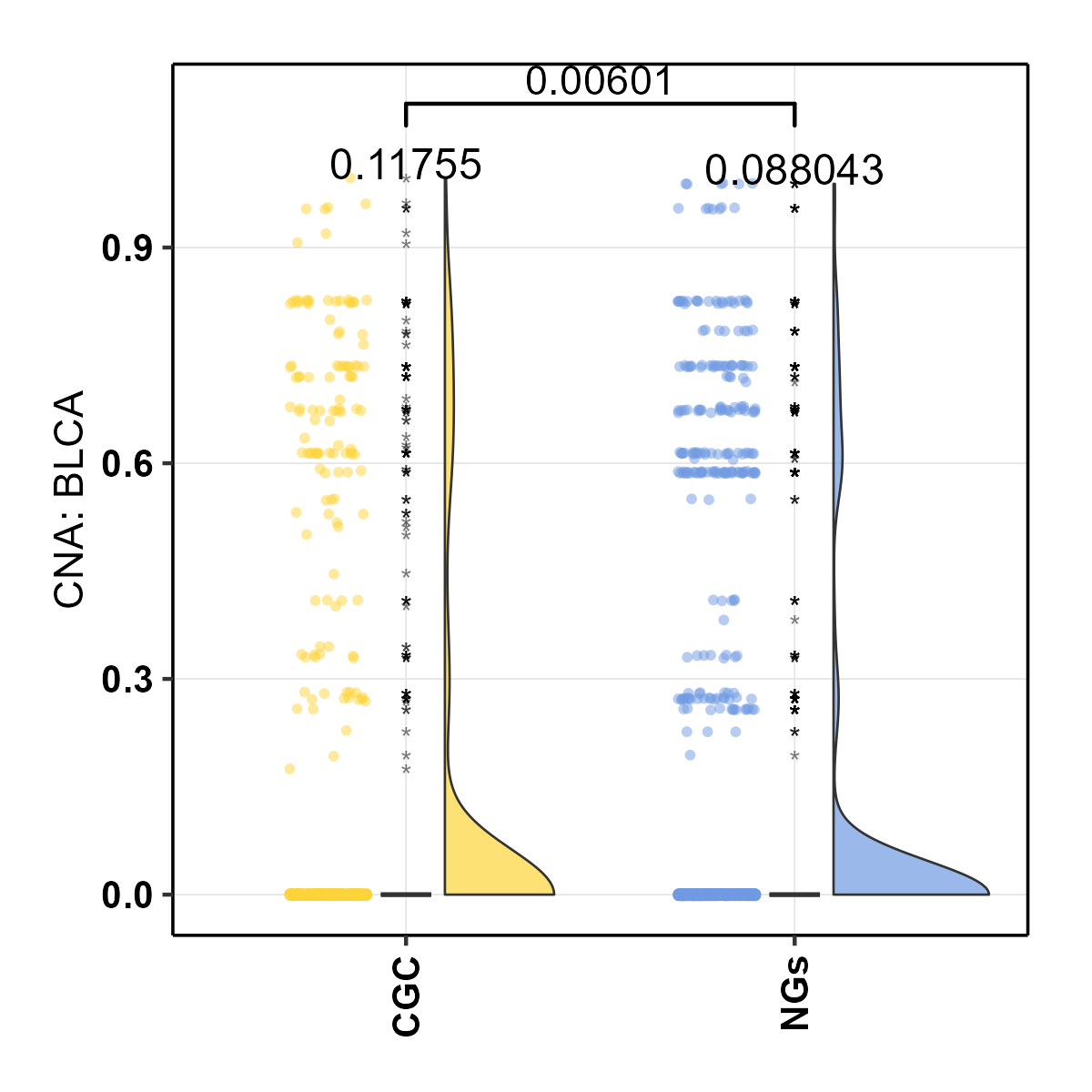

Supplement: Supplementary file 5 [file DataSheet2.ZIP › Supplementary file 5-2/STRINGdb/CNA_BLCA.png]

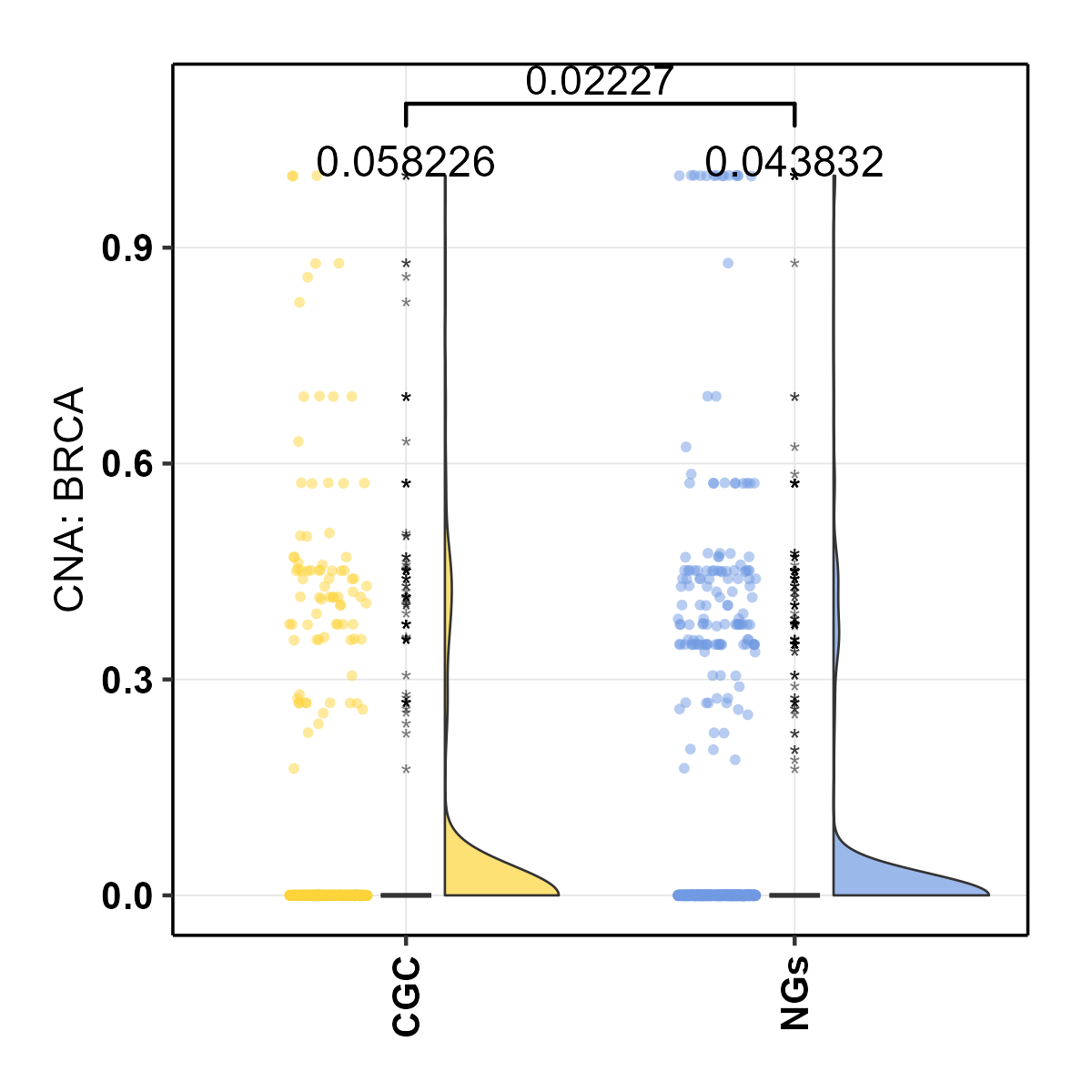

Supplement: Supplementary file 5 [file DataSheet2.ZIP › Supplementary file 5-2/STRINGdb/CNA_BRCA.png]

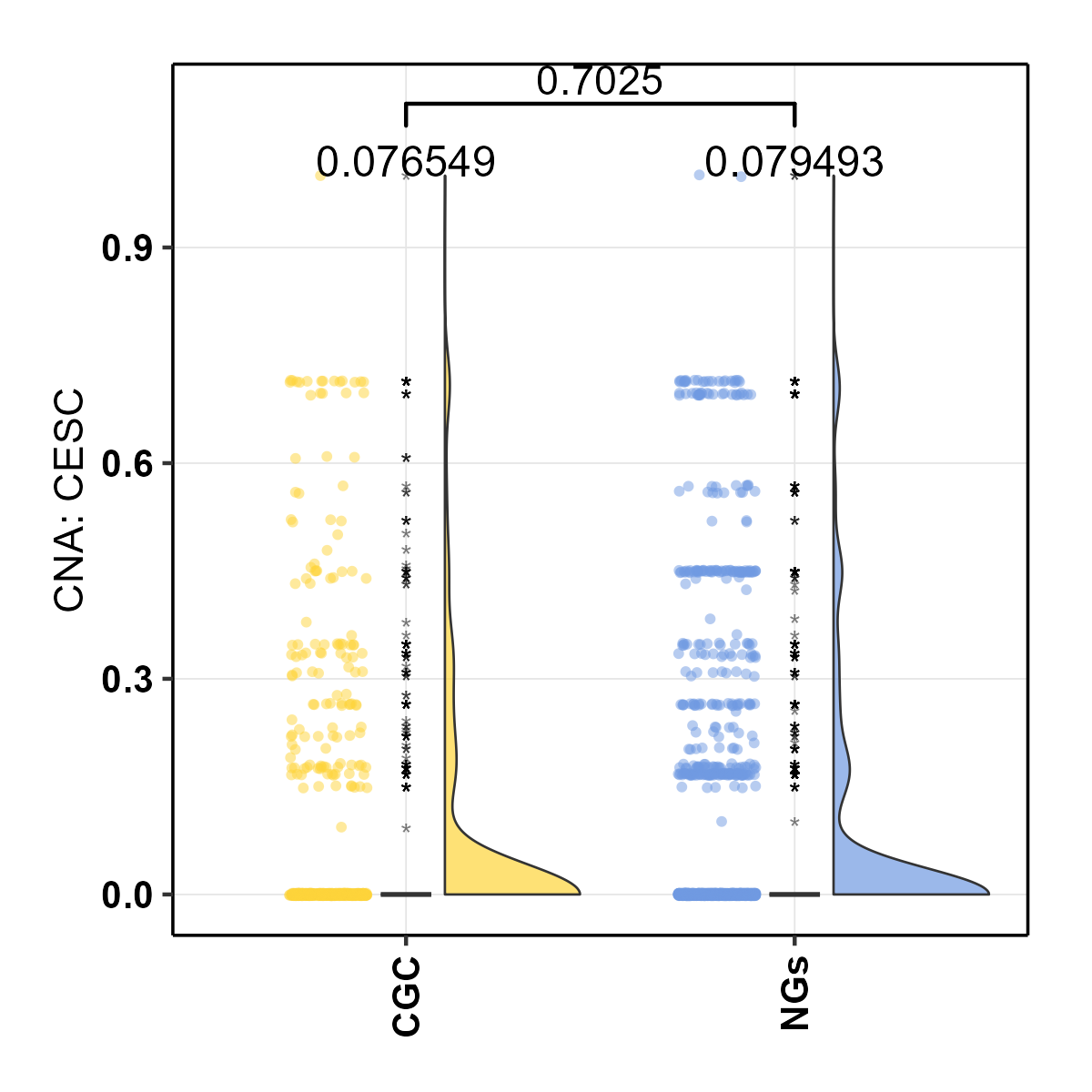

Supplement: Supplementary file 5 [file DataSheet2.ZIP › Supplementary file 5-2/STRINGdb/CNA_CESC.png]

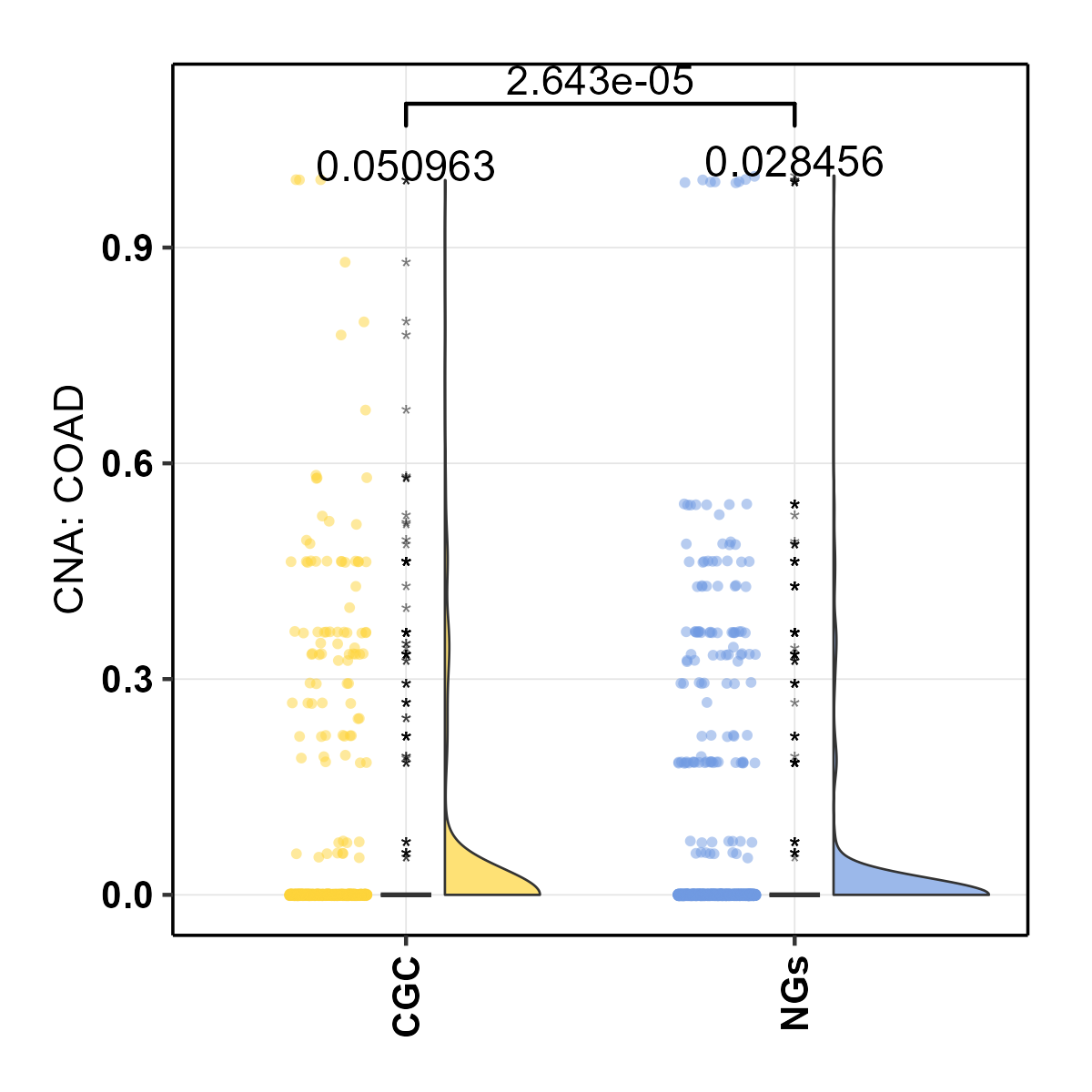

Supplement: Supplementary file 5 [file DataSheet2.ZIP › Supplementary file 5-2/STRINGdb/CNA_COAD.png]

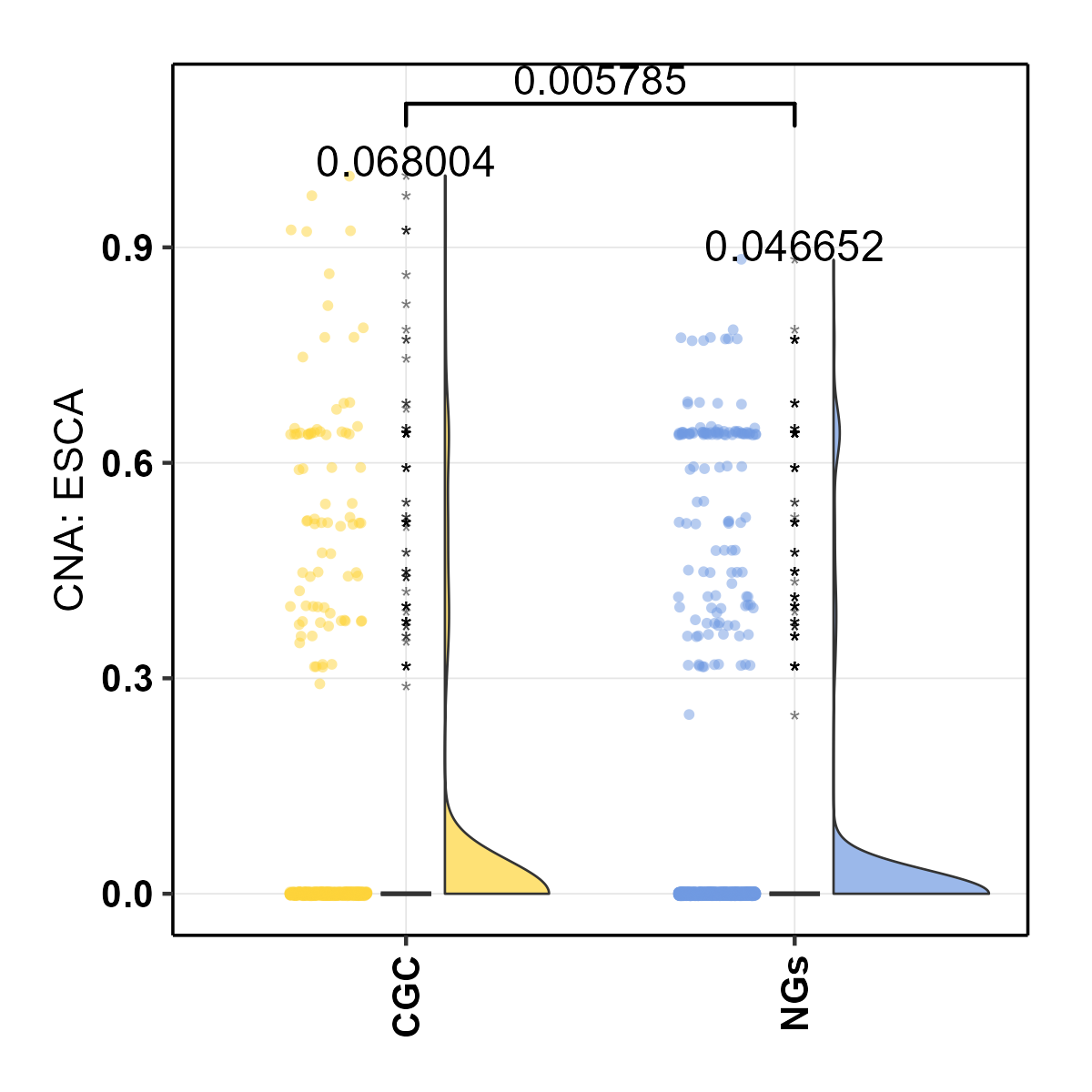

Supplement: Supplementary file 5 [file DataSheet2.ZIP › Supplementary file 5-2/STRINGdb/CNA_ESCA.png]

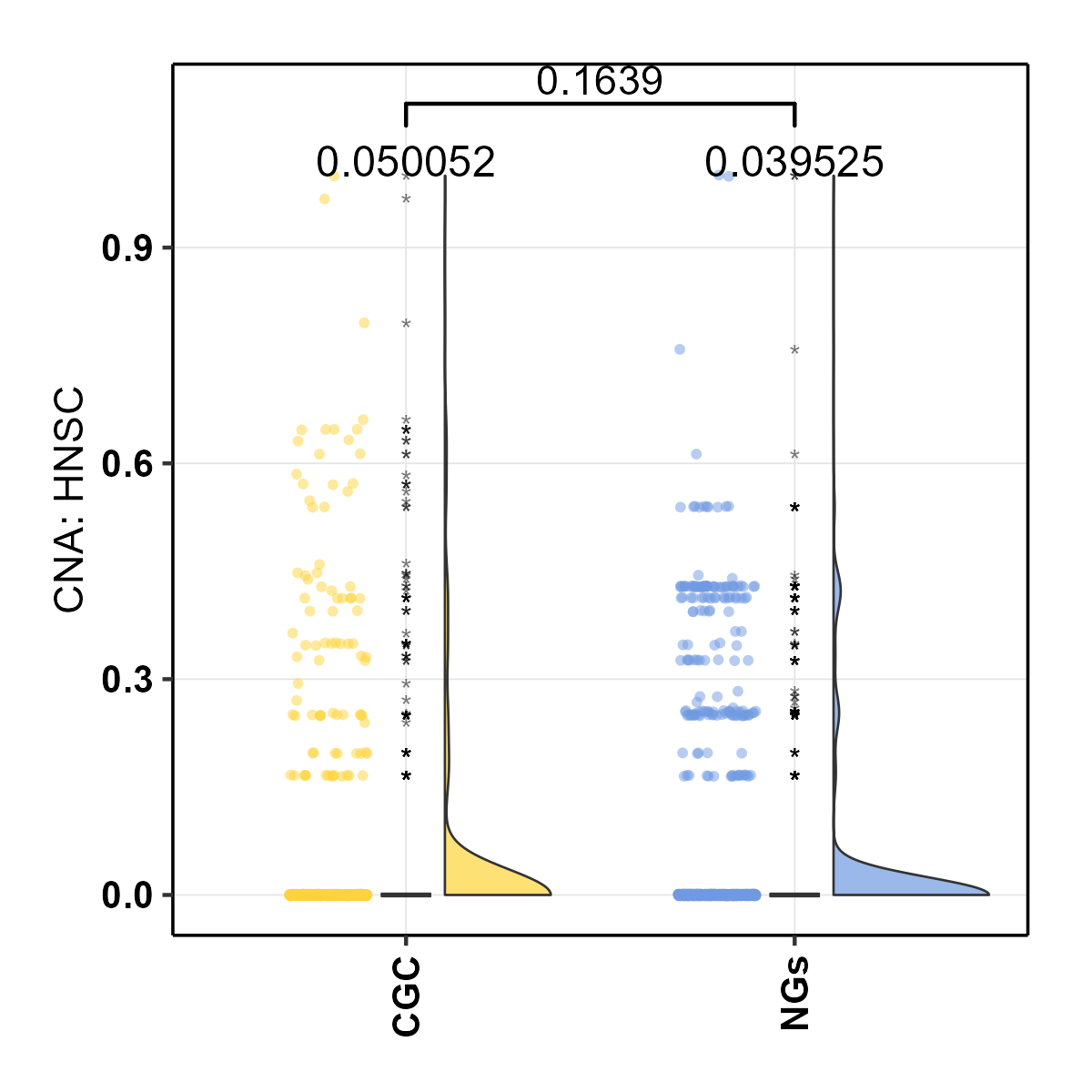

Supplement: Supplementary file 5 [file DataSheet2.ZIP › Supplementary file 5-2/STRINGdb/CNA_HNSC.png]

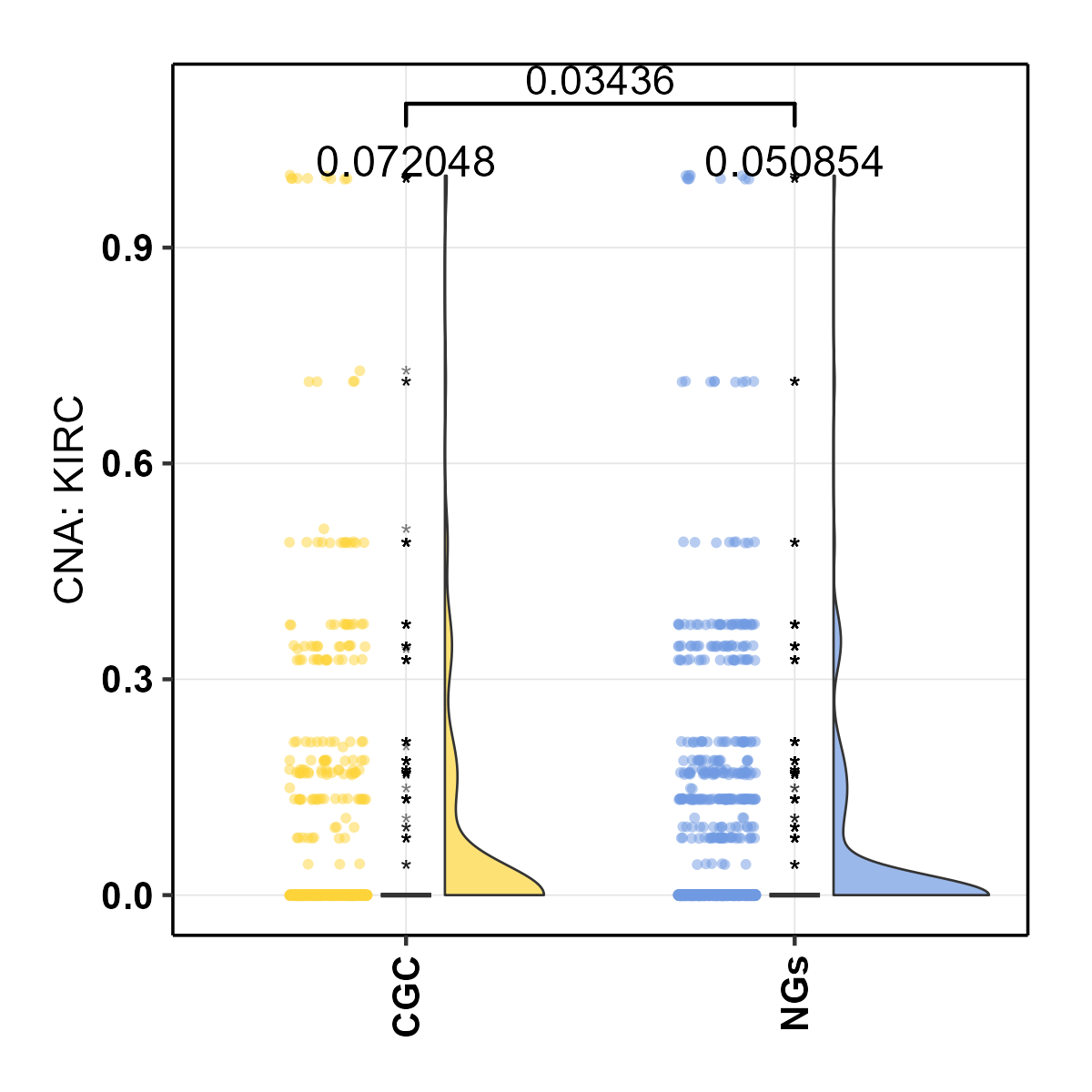

Supplement: Supplementary file 5 [file DataSheet2.ZIP › Supplementary file 5-2/STRINGdb/CNA_KIRC.png]

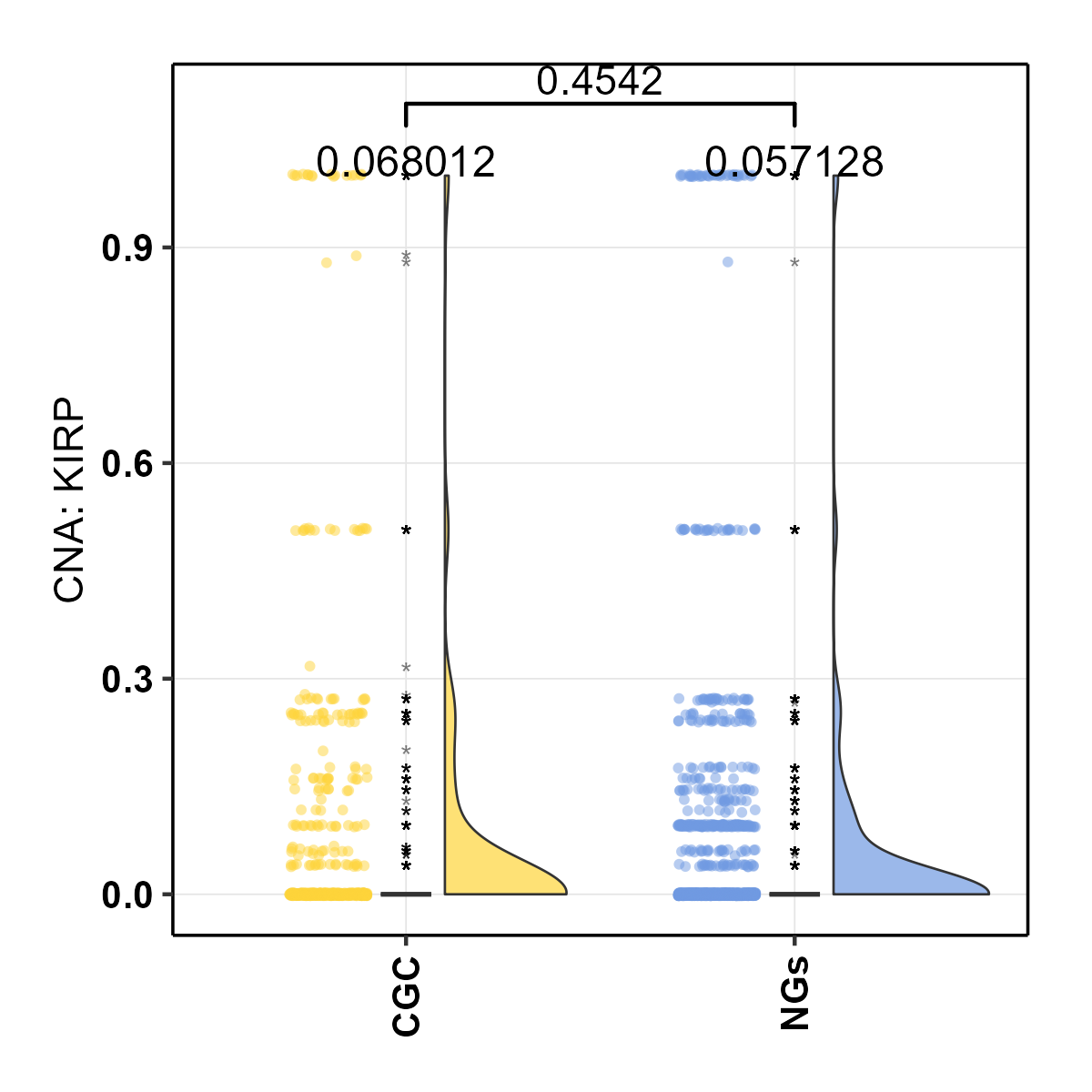

Supplement: Supplementary file 5 [file DataSheet2.ZIP › Supplementary file 5-2/STRINGdb/CNA_KIRP.png]

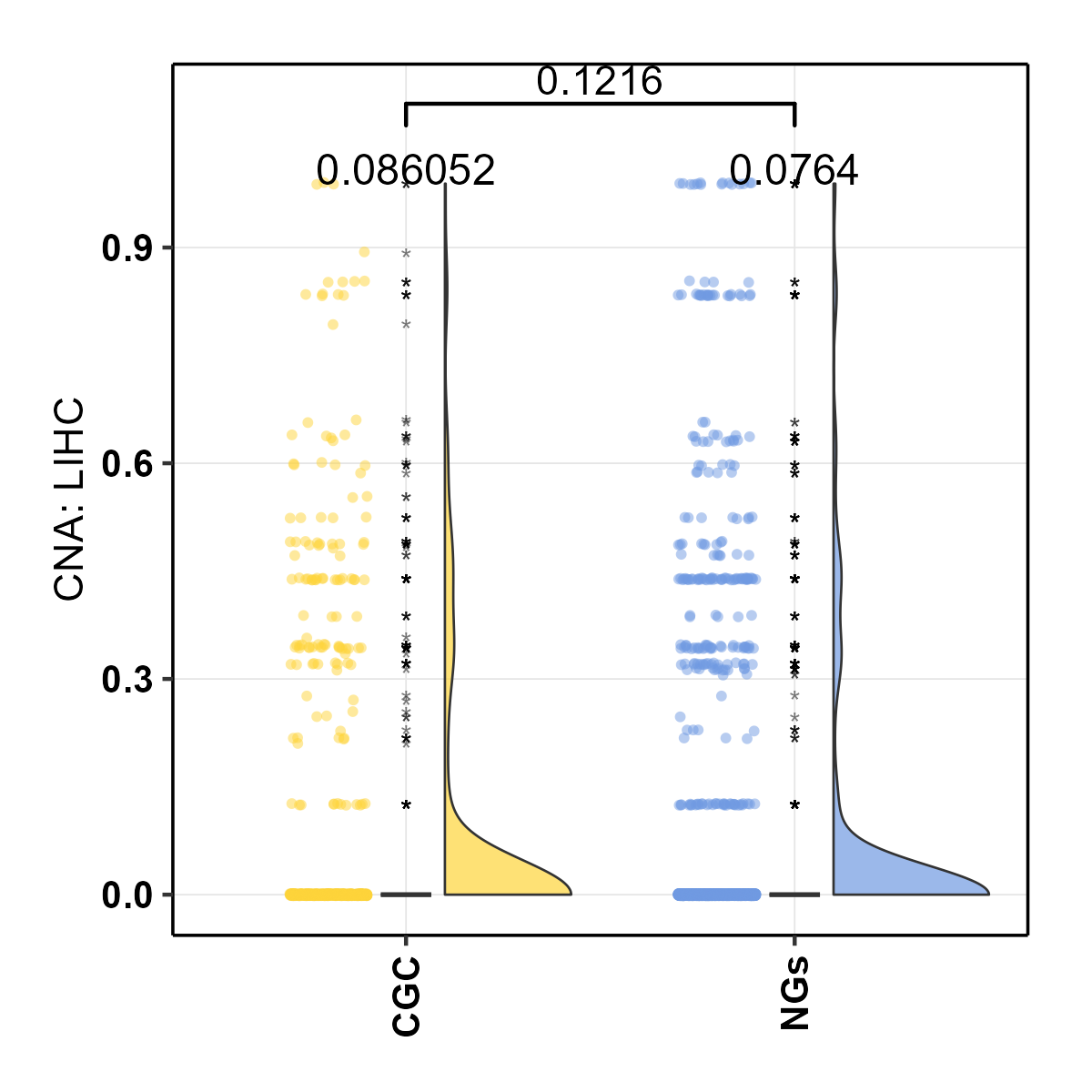

Supplement: Supplementary file 5 [file DataSheet2.ZIP › Supplementary file 5-2/STRINGdb/CNA_LIHC.png]

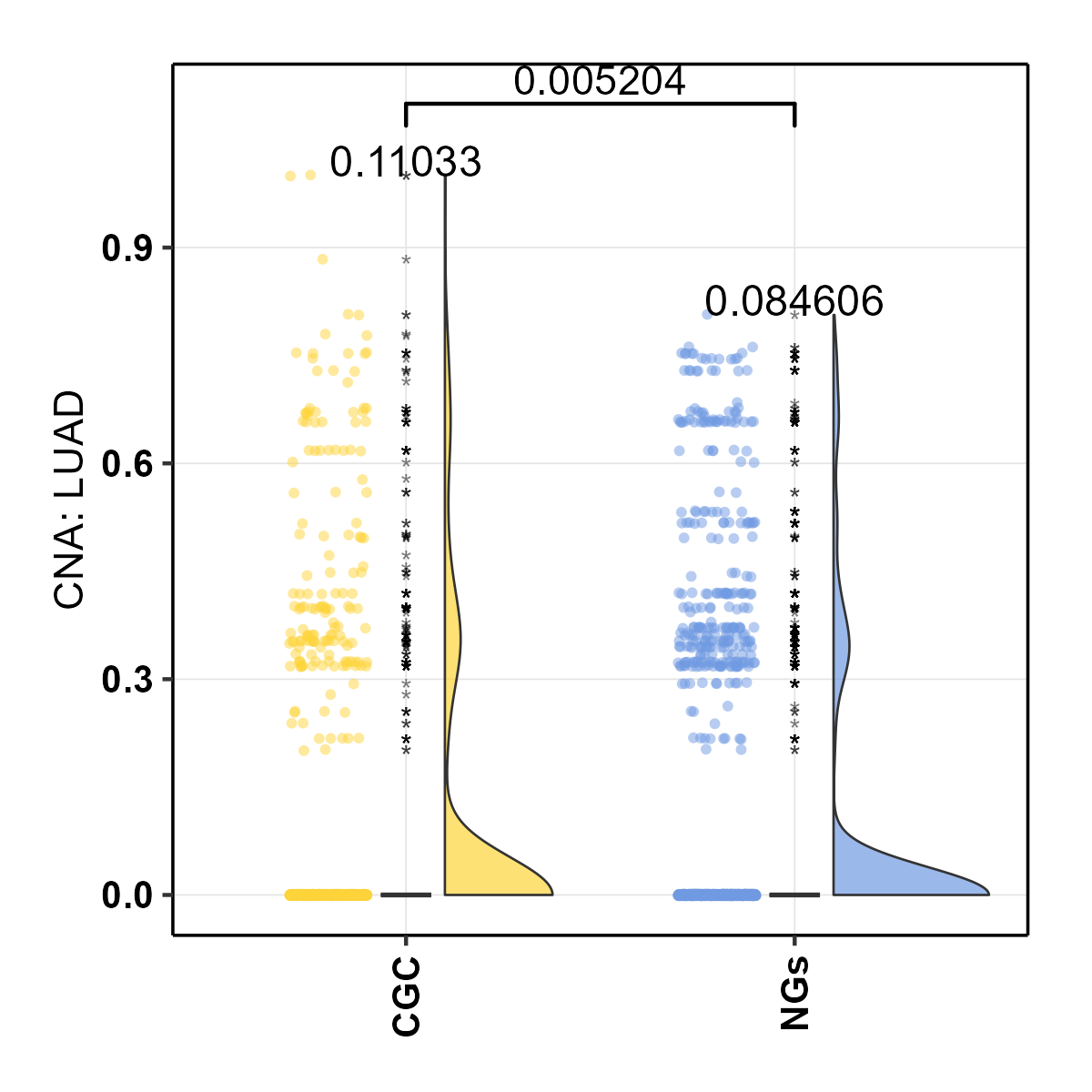

Supplement: Supplementary file 5 [file DataSheet2.ZIP › Supplementary file 5-2/STRINGdb/CNA_LUAD.png]

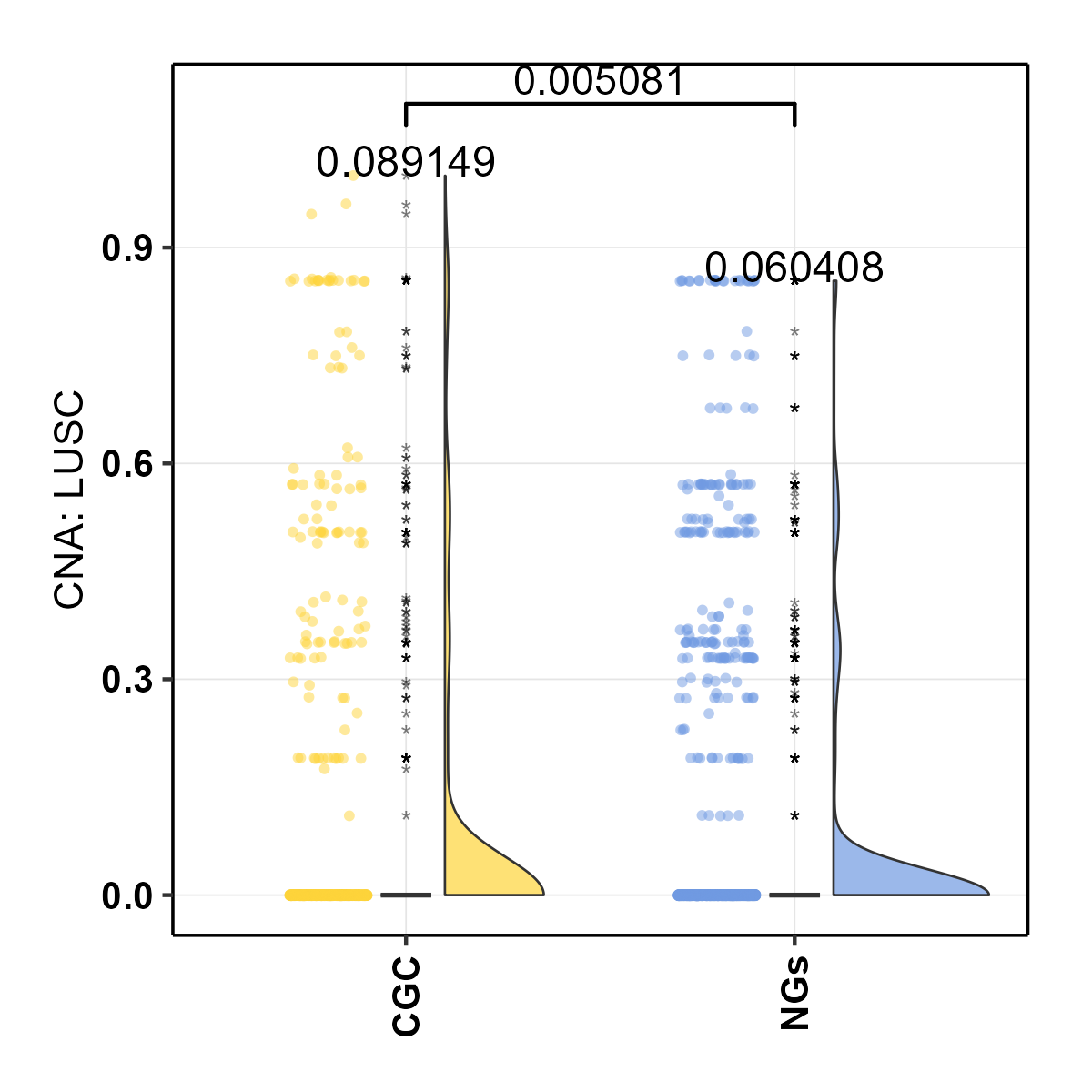

Supplement: Supplementary file 5 [file DataSheet2.ZIP › Supplementary file 5-2/STRINGdb/CNA_LUSC.png]

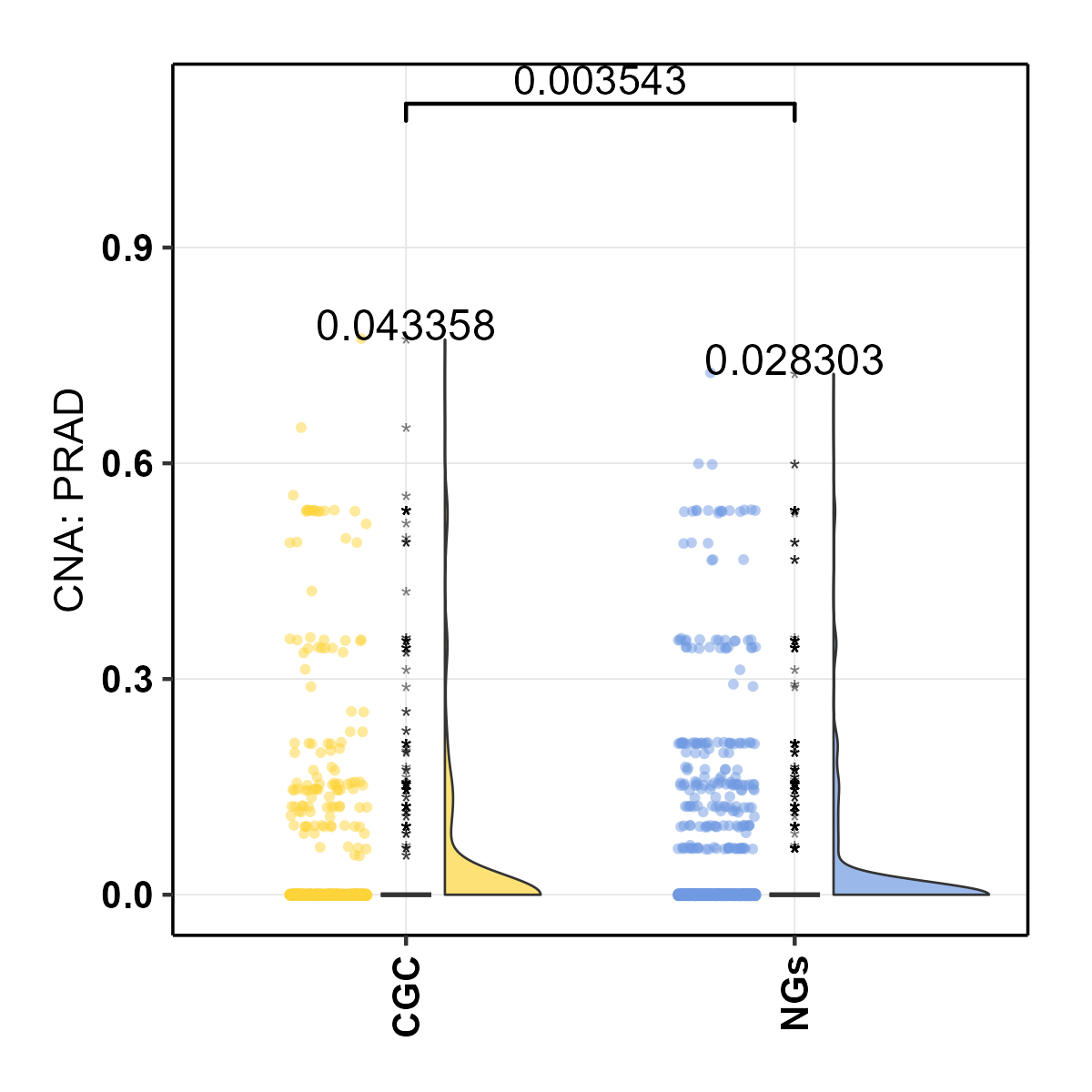

Supplement: Supplementary file 5 [file DataSheet2.ZIP › Supplementary file 5-2/STRINGdb/CNA_PRAD.png]

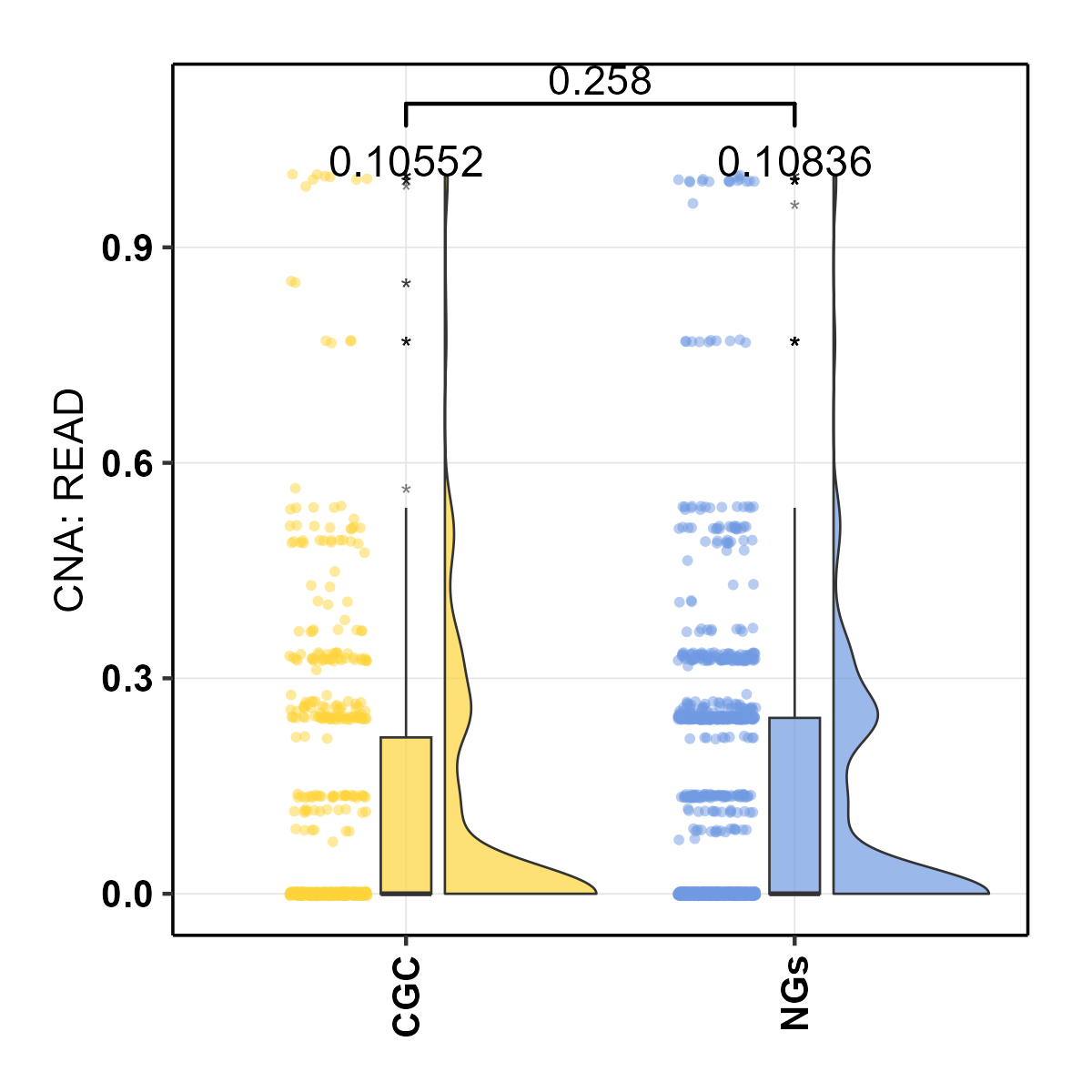

Supplement: Supplementary file 5 [file DataSheet2.ZIP › Supplementary file 5-2/STRINGdb/CNA_READ.png]

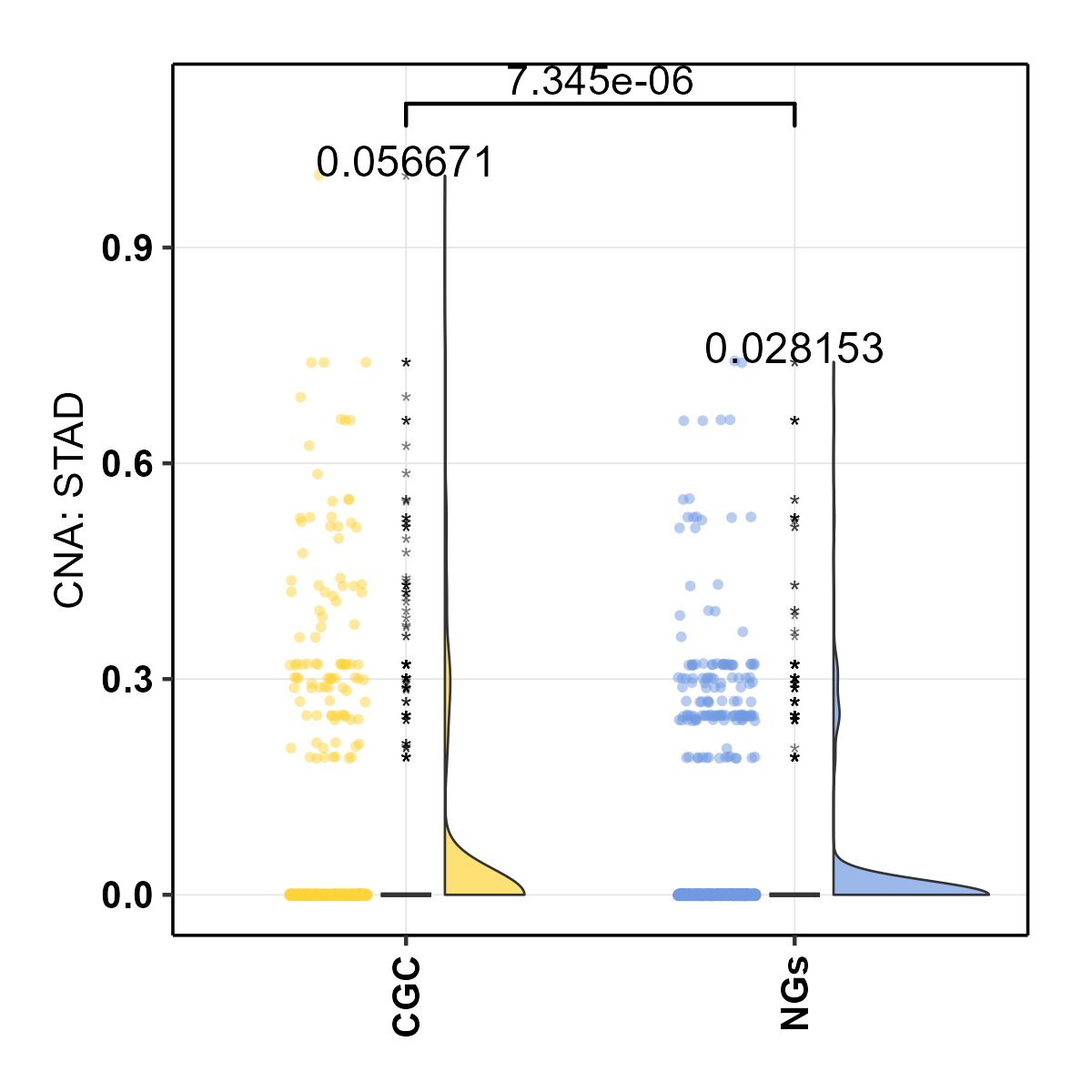

Supplement: Supplementary file 5 [file DataSheet2.ZIP › Supplementary file 5-2/STRINGdb/CNA_STAD.png]

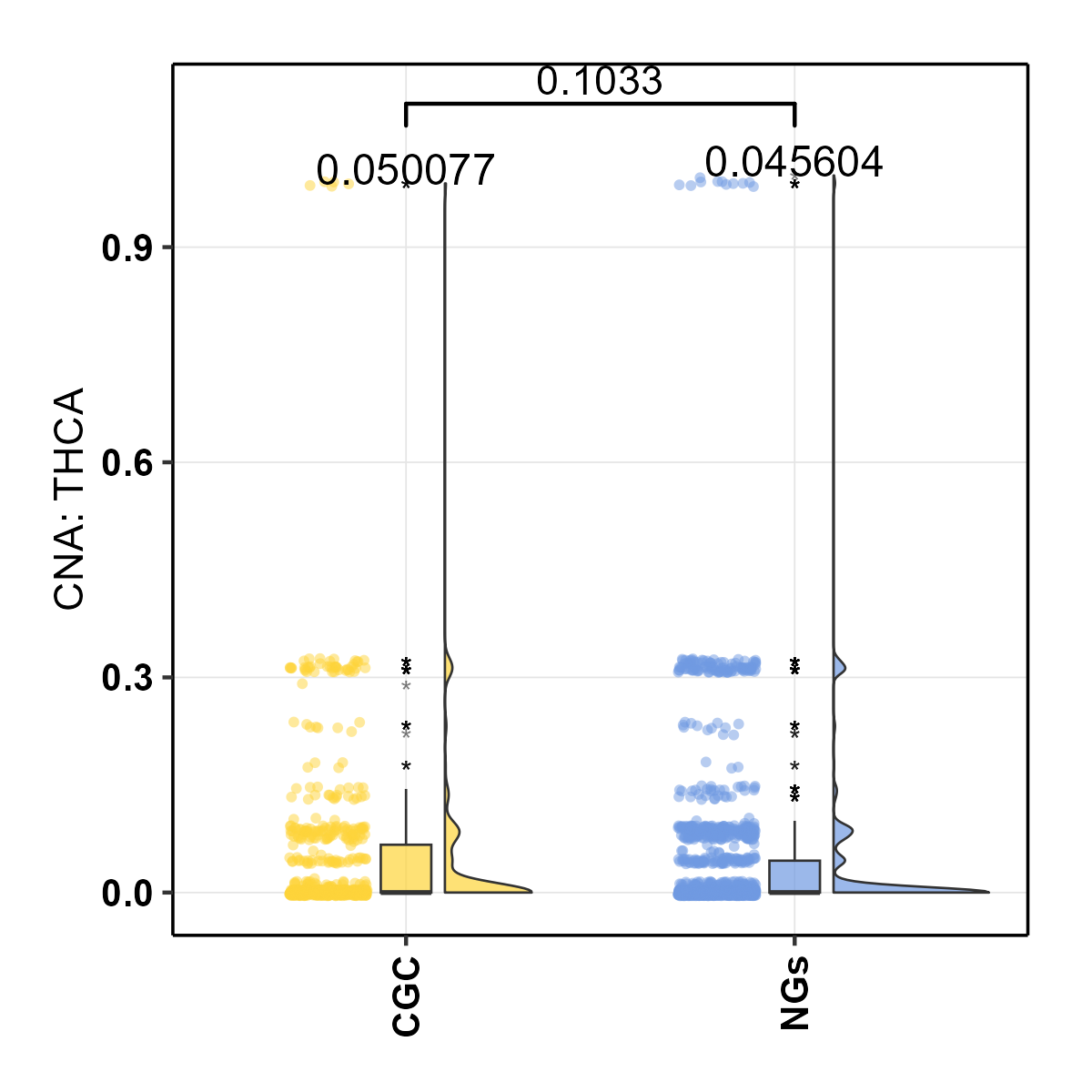

Supplement: Supplementary file 5 [file DataSheet2.ZIP › Supplementary file 5-2/STRINGdb/CNA_THCA.png]

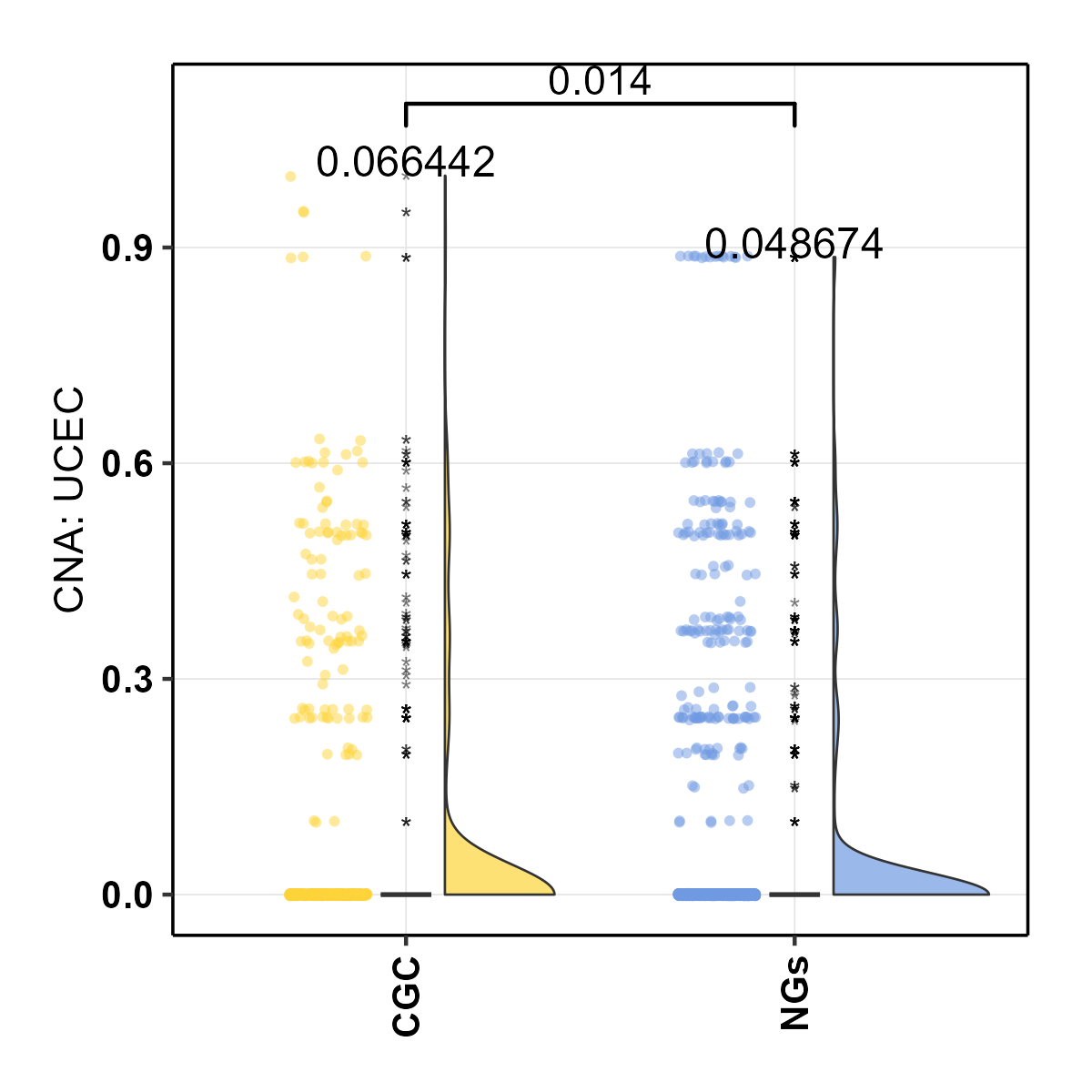

Supplement: Supplementary file 5 [file DataSheet2.ZIP › Supplementary file 5-2/STRINGdb/CNA_UCEC.png]

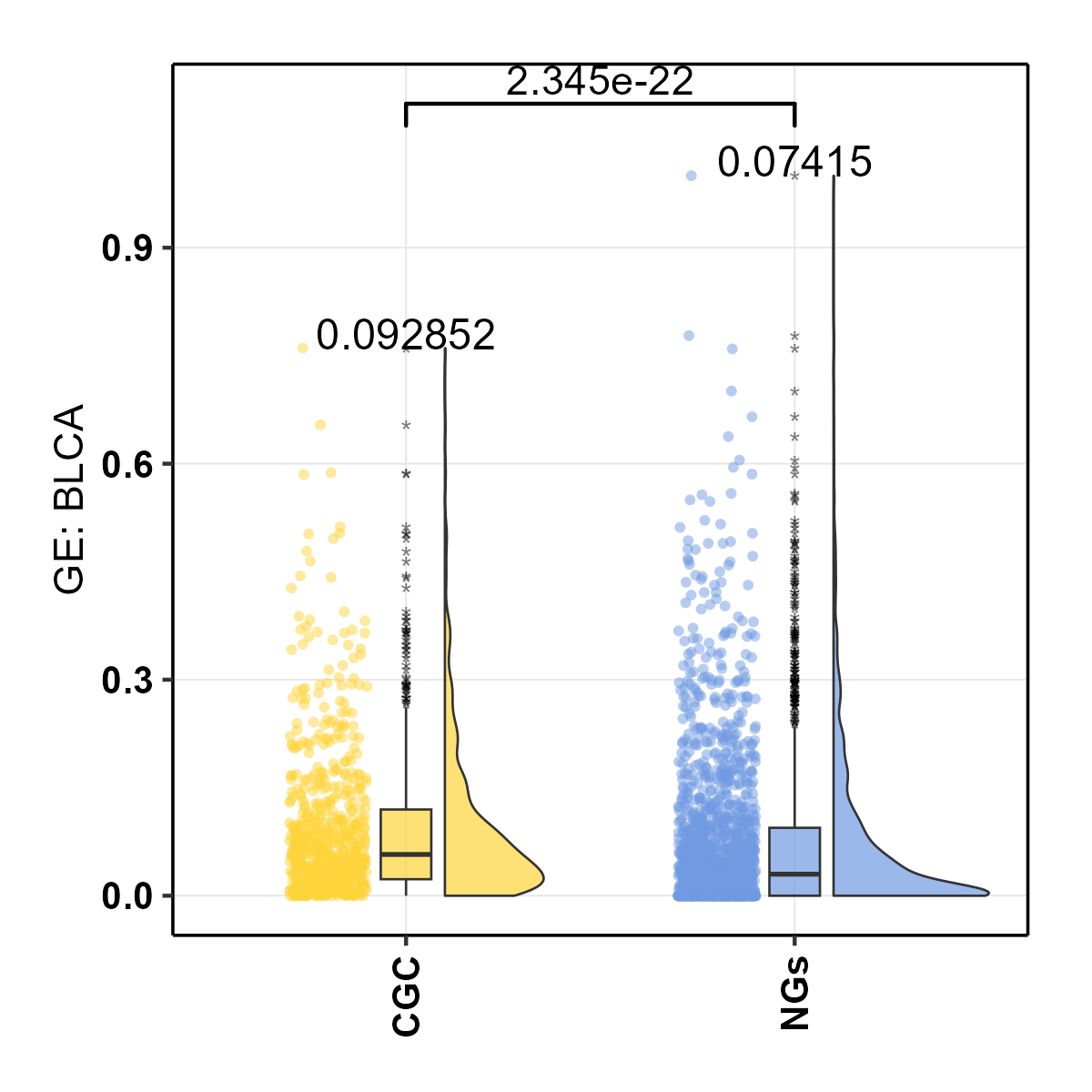

Supplement: Supplementary file 5 [file DataSheet2.ZIP › Supplementary file 5-2/STRINGdb/GE_BLCA.png]

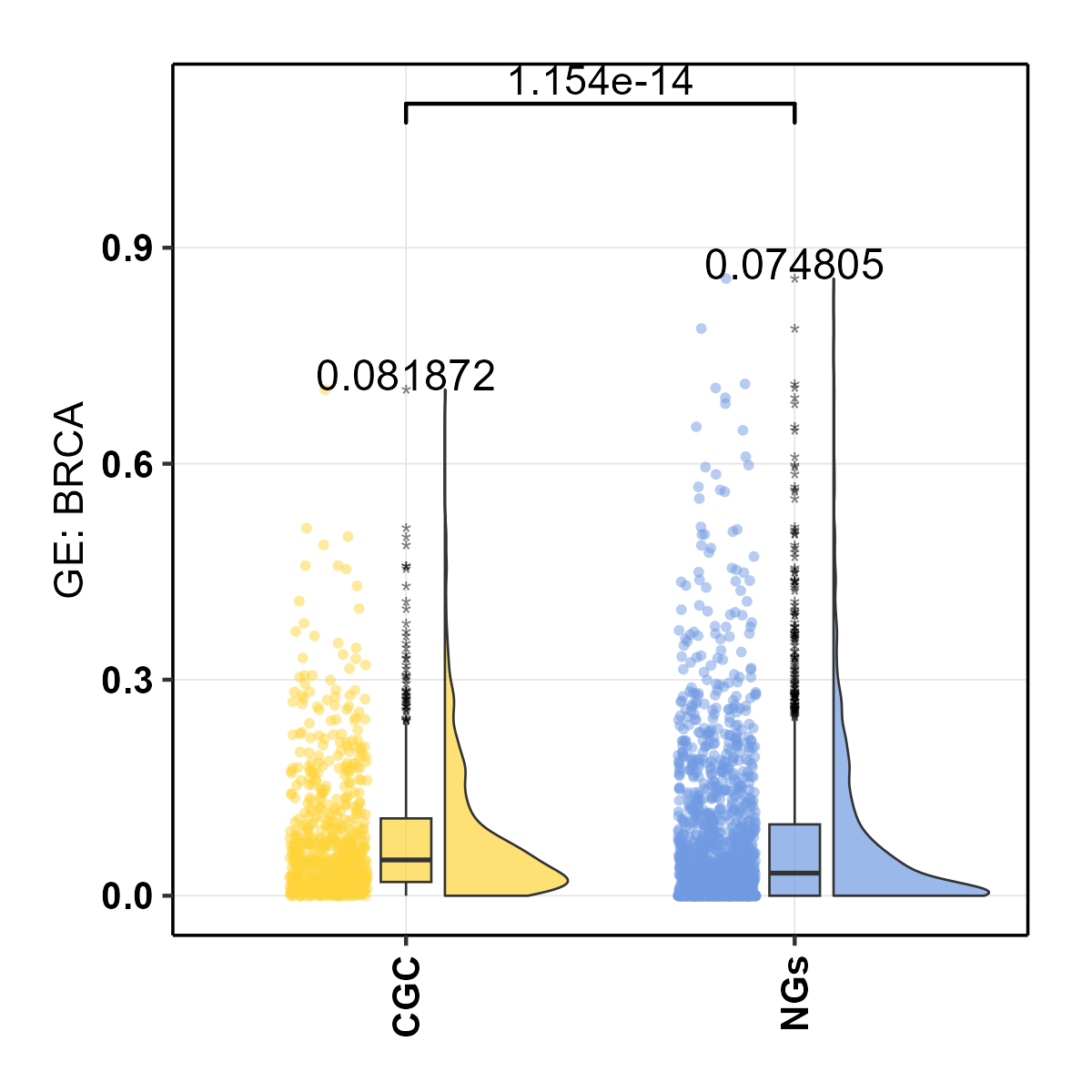

Supplement: Supplementary file 5 [file DataSheet2.ZIP › Supplementary file 5-2/STRINGdb/GE_BRCA.png]

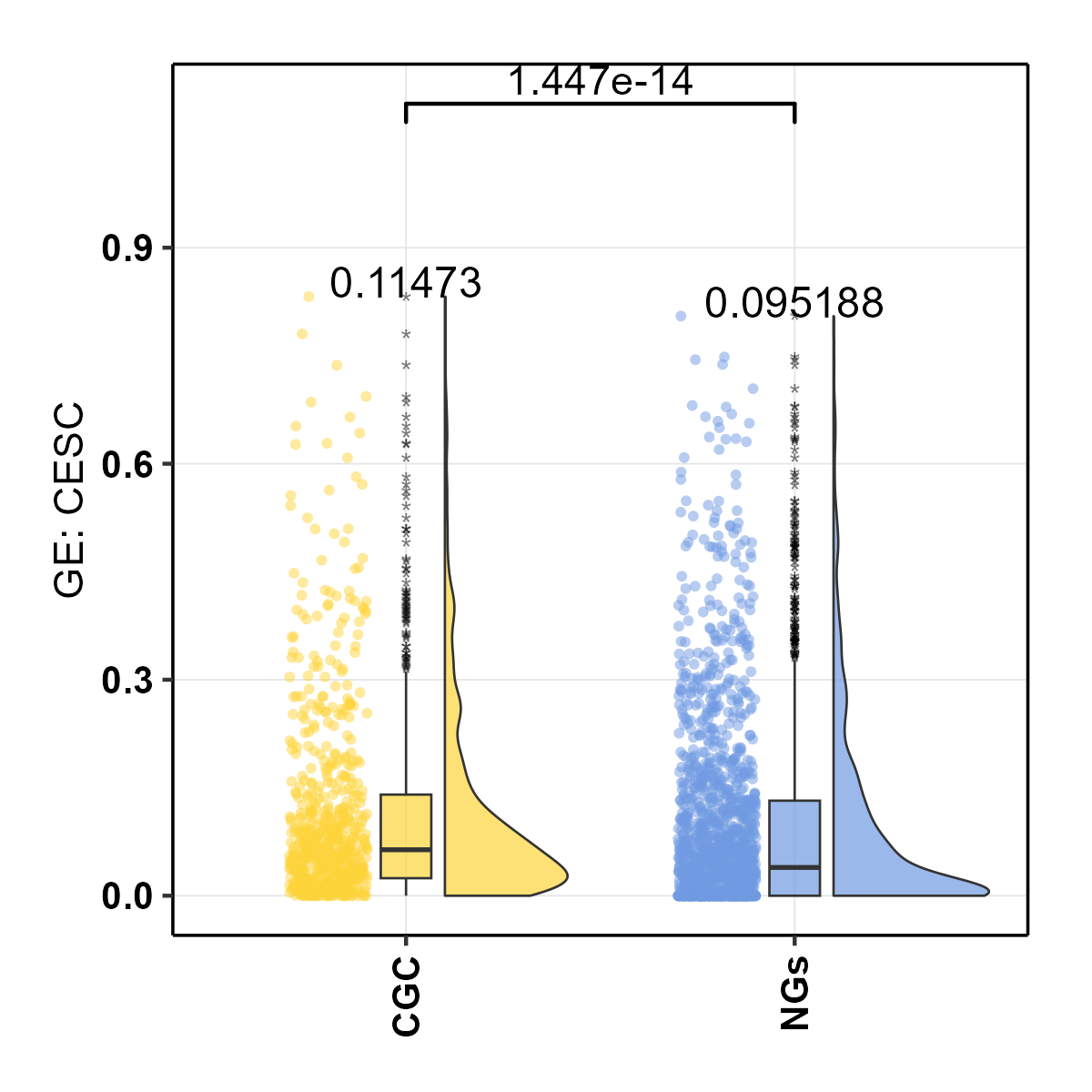

Supplement: Supplementary file 5 [file DataSheet2.ZIP › Supplementary file 5-2/STRINGdb/GE_CESC.png]

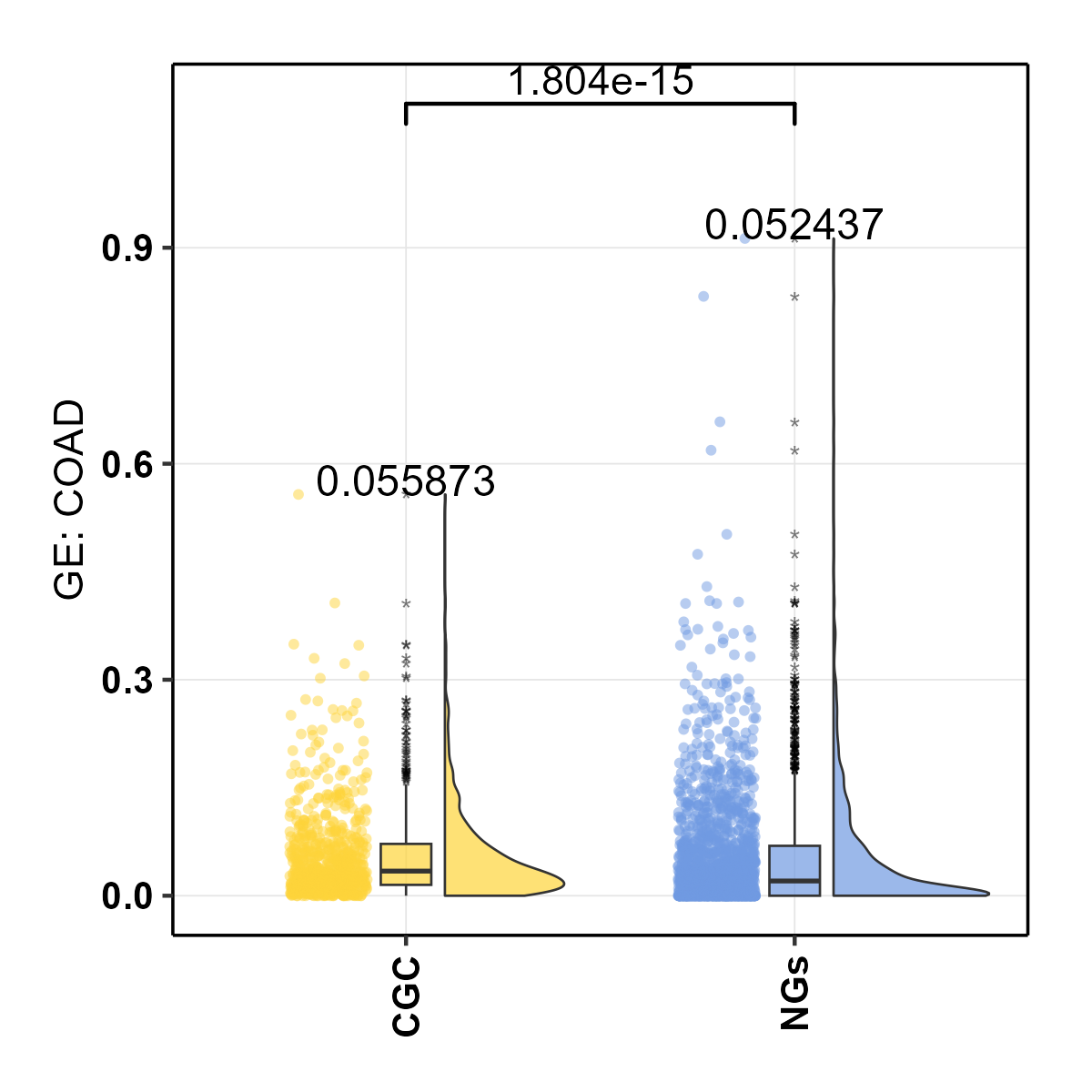

Supplement: Supplementary file 5 [file DataSheet2.ZIP › Supplementary file 5-2/STRINGdb/GE_COAD.png]

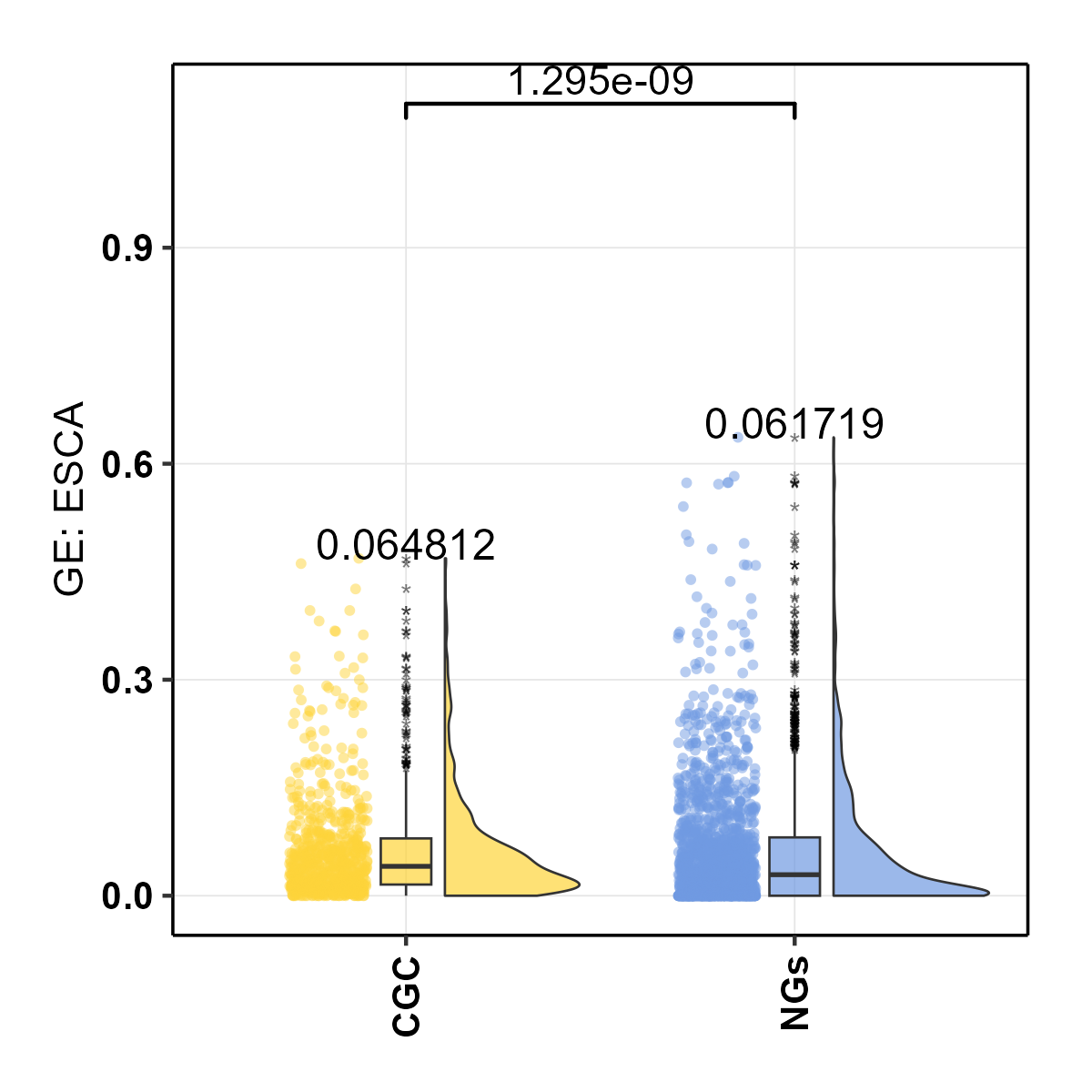

Supplement: Supplementary file 5 [file DataSheet2.ZIP › Supplementary file 5-2/STRINGdb/GE_ESCA.png]

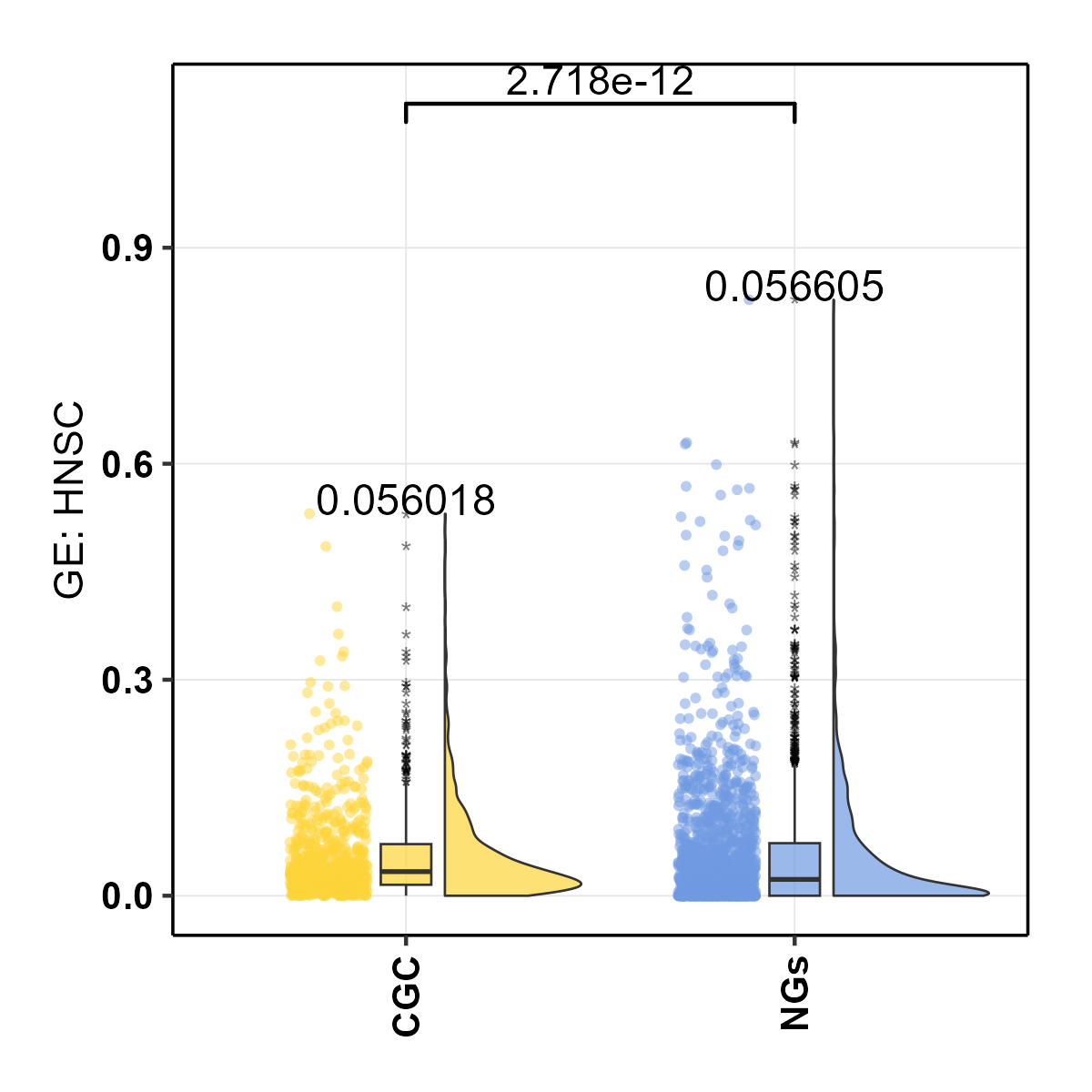

Supplement: Supplementary file 5 [file DataSheet2.ZIP › Supplementary file 5-2/STRINGdb/GE_HNSC.png]

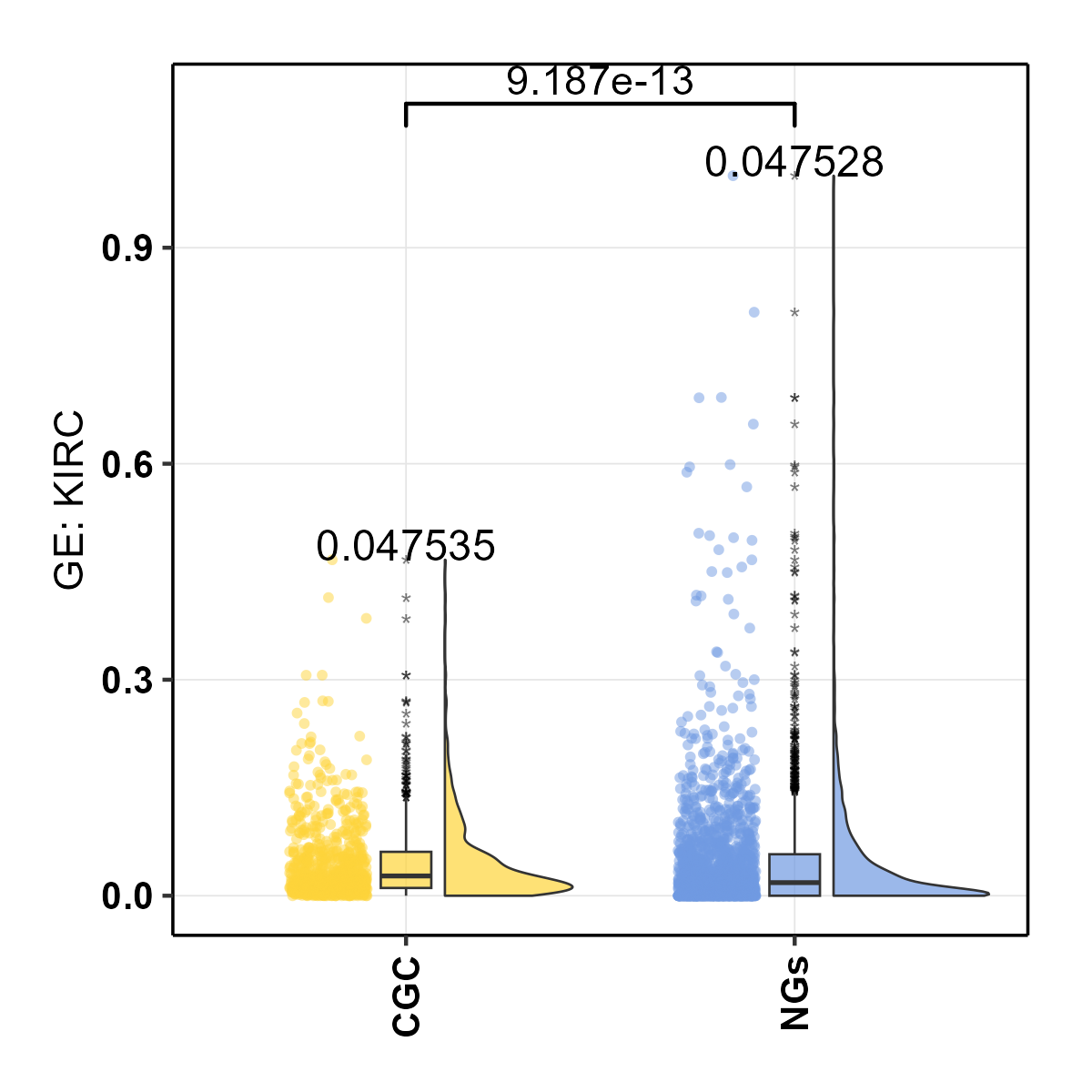

Supplement: Supplementary file 5 [file DataSheet2.ZIP › Supplementary file 5-2/STRINGdb/GE_KIRC.png]

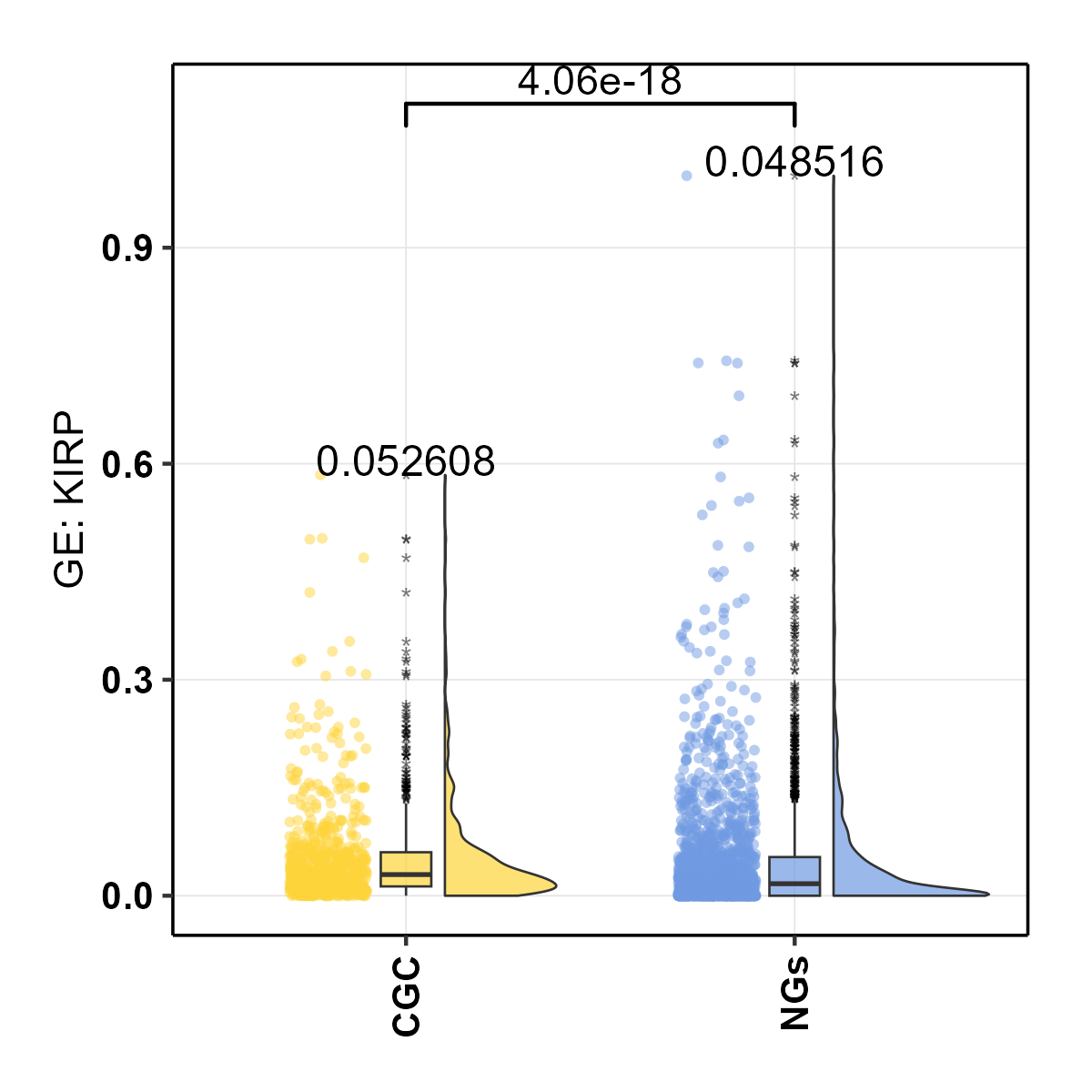

Supplement: Supplementary file 5 [file DataSheet2.ZIP › Supplementary file 5-2/STRINGdb/GE_KIRP.png]

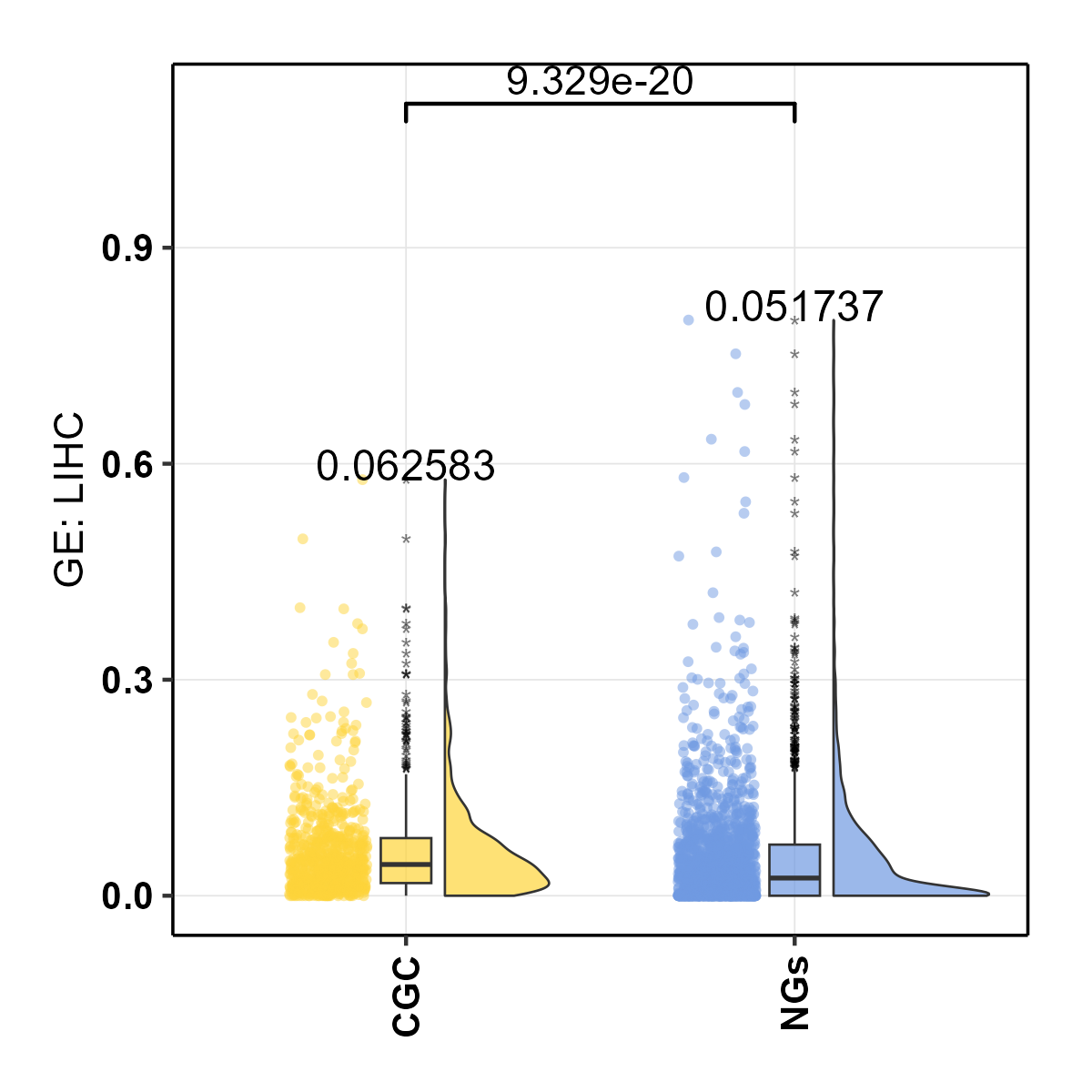

Supplement: Supplementary file 5 [file DataSheet2.ZIP › Supplementary file 5-2/STRINGdb/GE_LIHC.png]

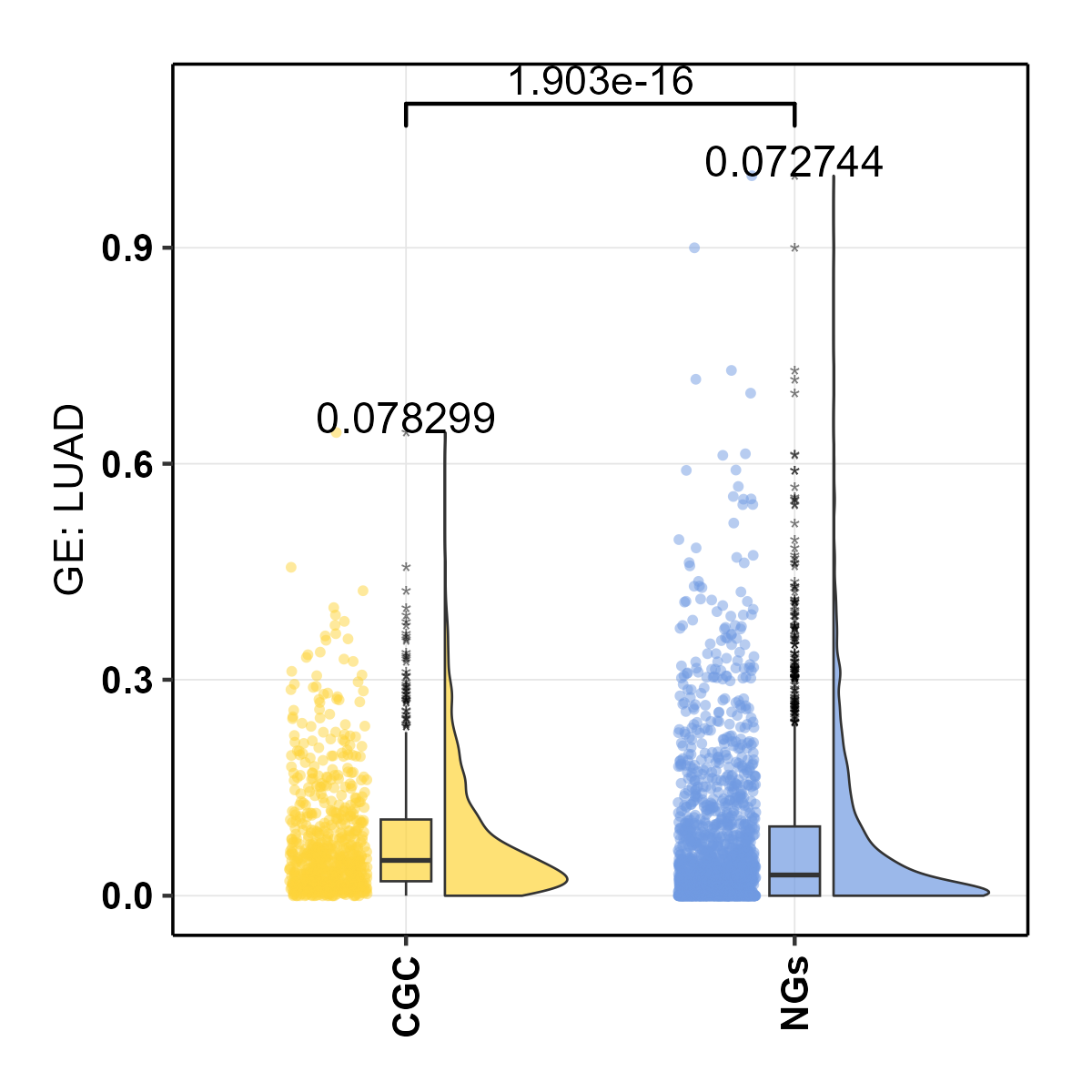

Supplement: Supplementary file 5 [file DataSheet2.ZIP › Supplementary file 5-2/STRINGdb/GE_LUAD.png]

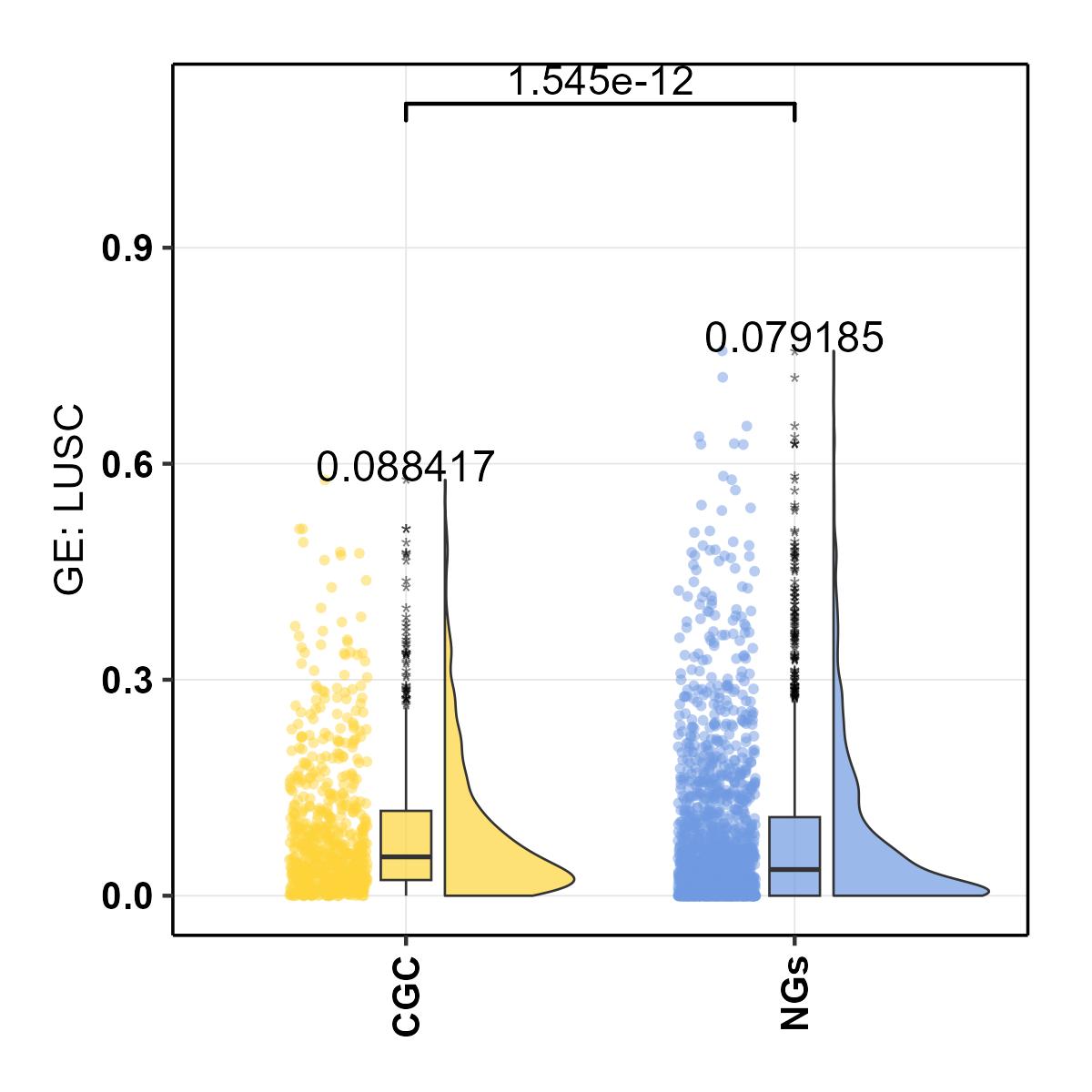

Supplement: Supplementary file 5 [file DataSheet2.ZIP › Supplementary file 5-2/STRINGdb/GE_LUSC.png]

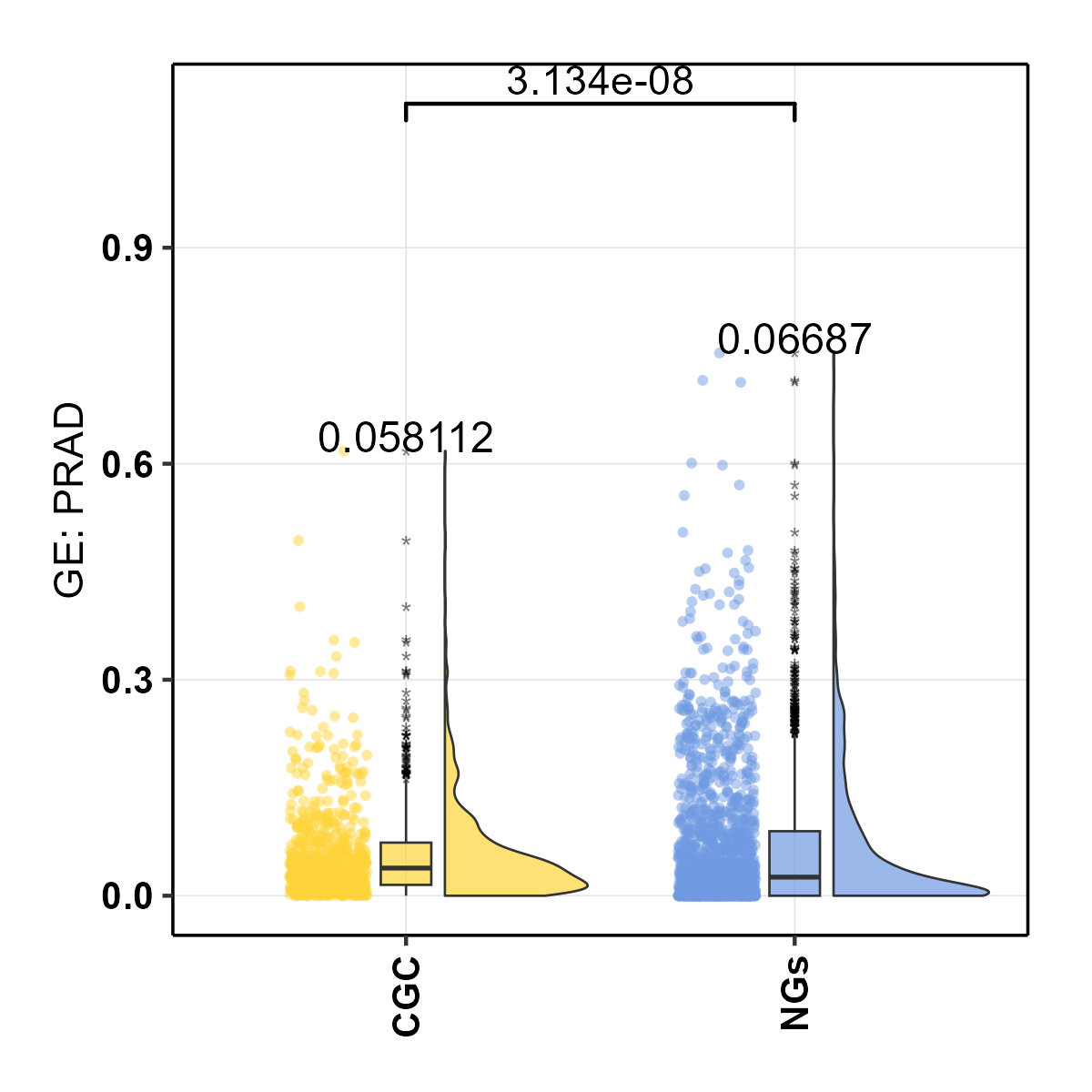

Supplement: Supplementary file 5 [file DataSheet2.ZIP › Supplementary file 5-2/STRINGdb/GE_PRAD.png]

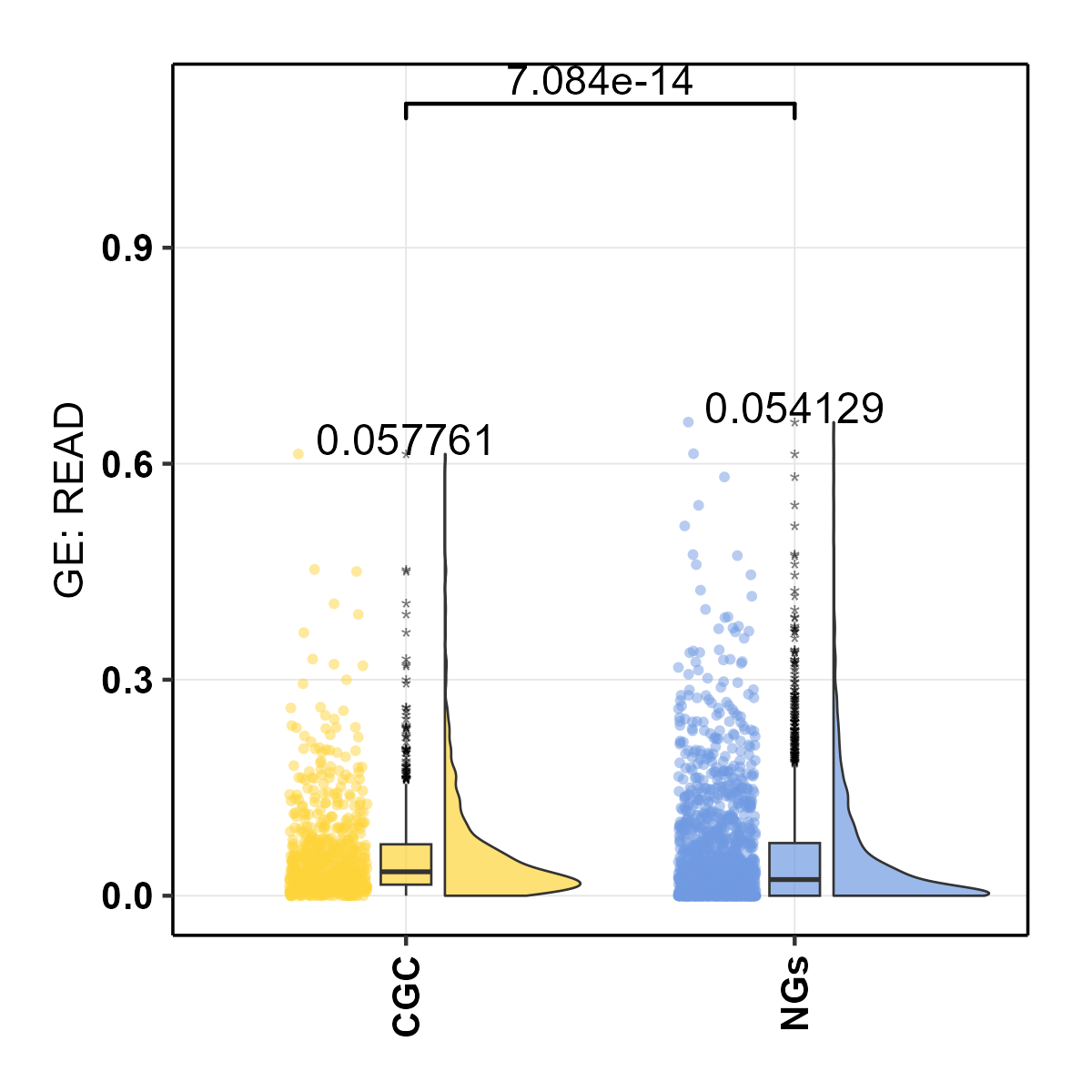

Supplement: Supplementary file 5 [file DataSheet2.ZIP › Supplementary file 5-2/STRINGdb/GE_READ.png]

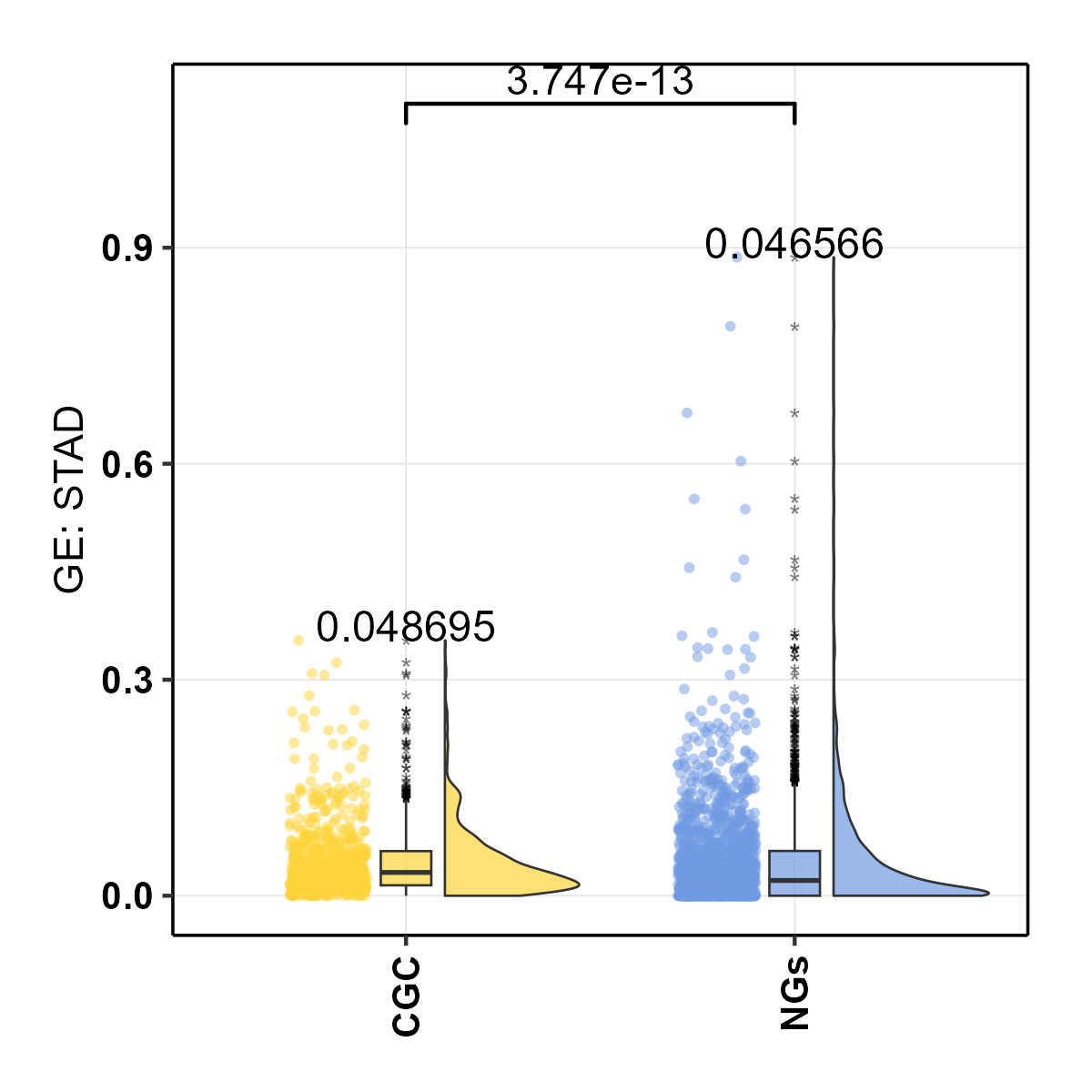

Supplement: Supplementary file 5 [file DataSheet2.ZIP › Supplementary file 5-2/STRINGdb/GE_STAD.png]

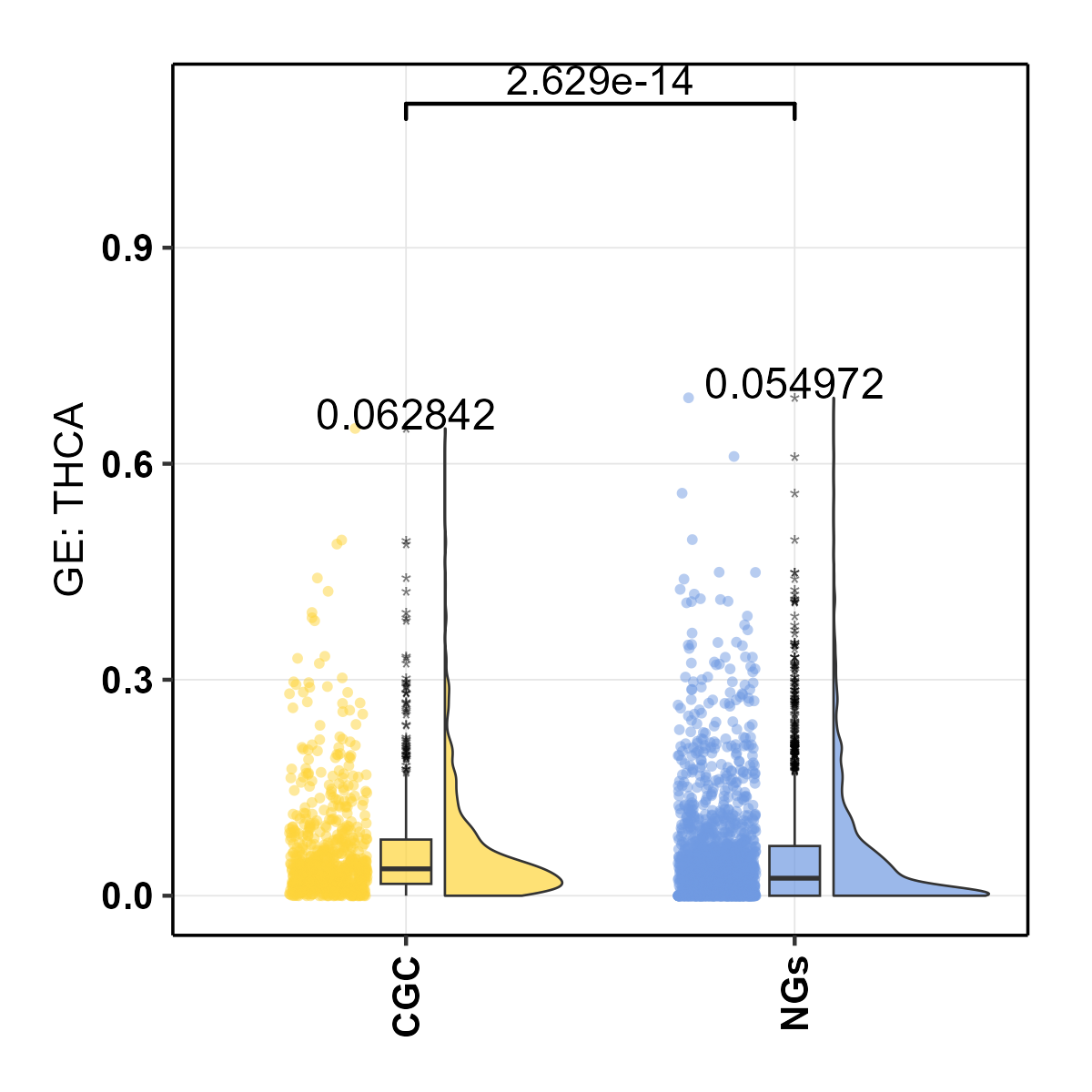

Supplement: Supplementary file 5 [file DataSheet2.ZIP › Supplementary file 5-2/STRINGdb/GE_THCA.png]

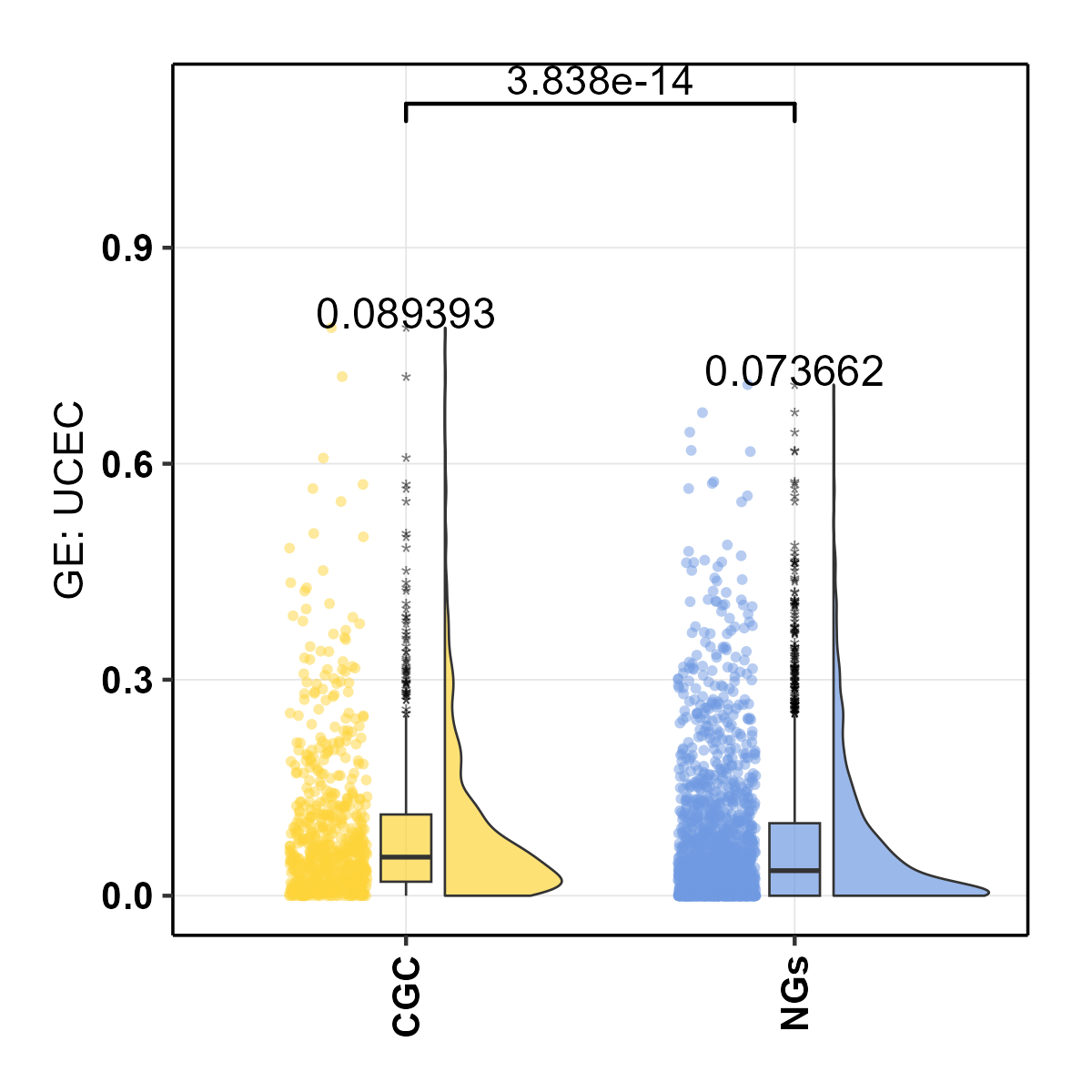

Supplement: Supplementary file 5 [file DataSheet2.ZIP › Supplementary file 5-2/STRINGdb/GE_UCEC.png]

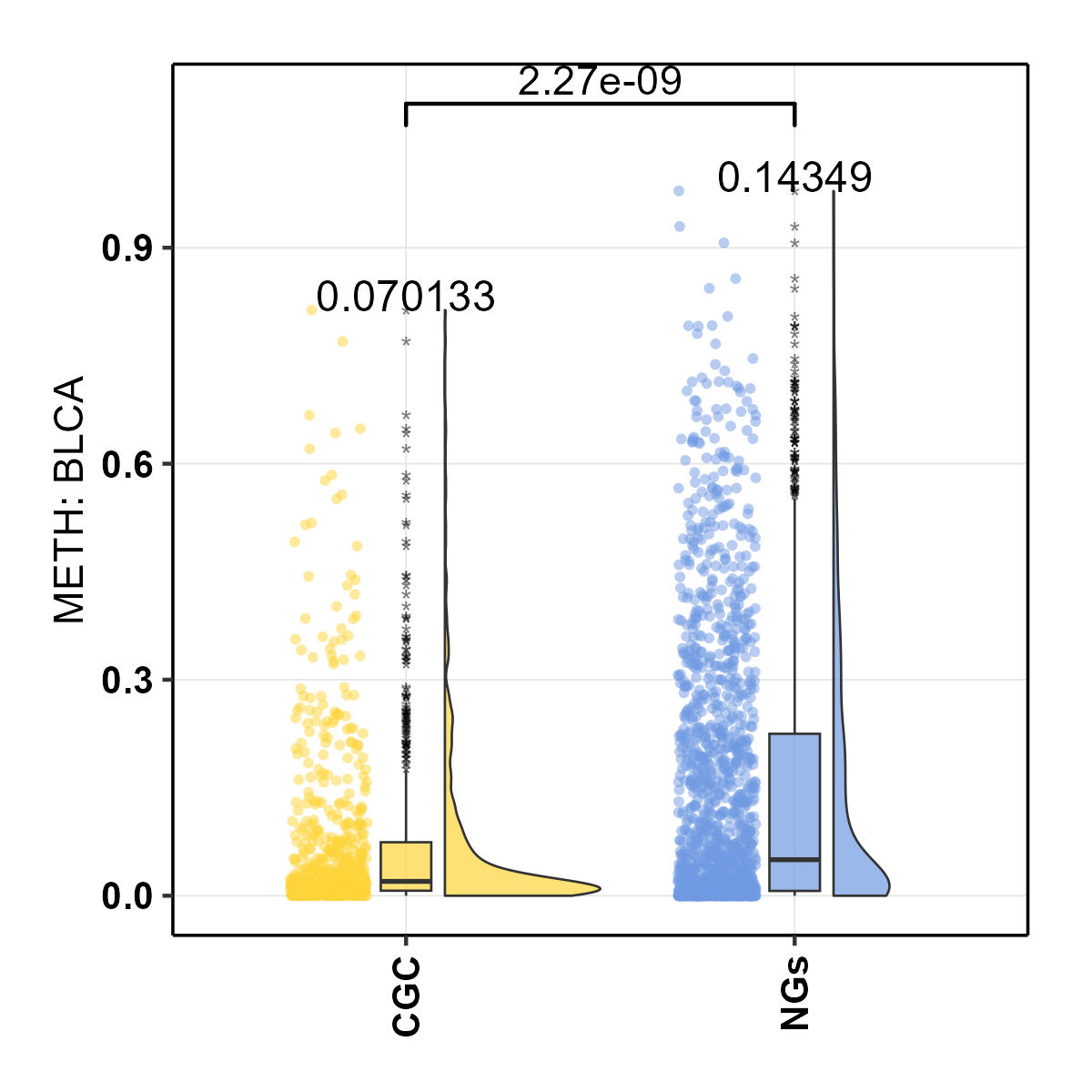

Supplement: Supplementary file 5 [file DataSheet2.ZIP › Supplementary file 5-2/STRINGdb/METH_BLCA.png]

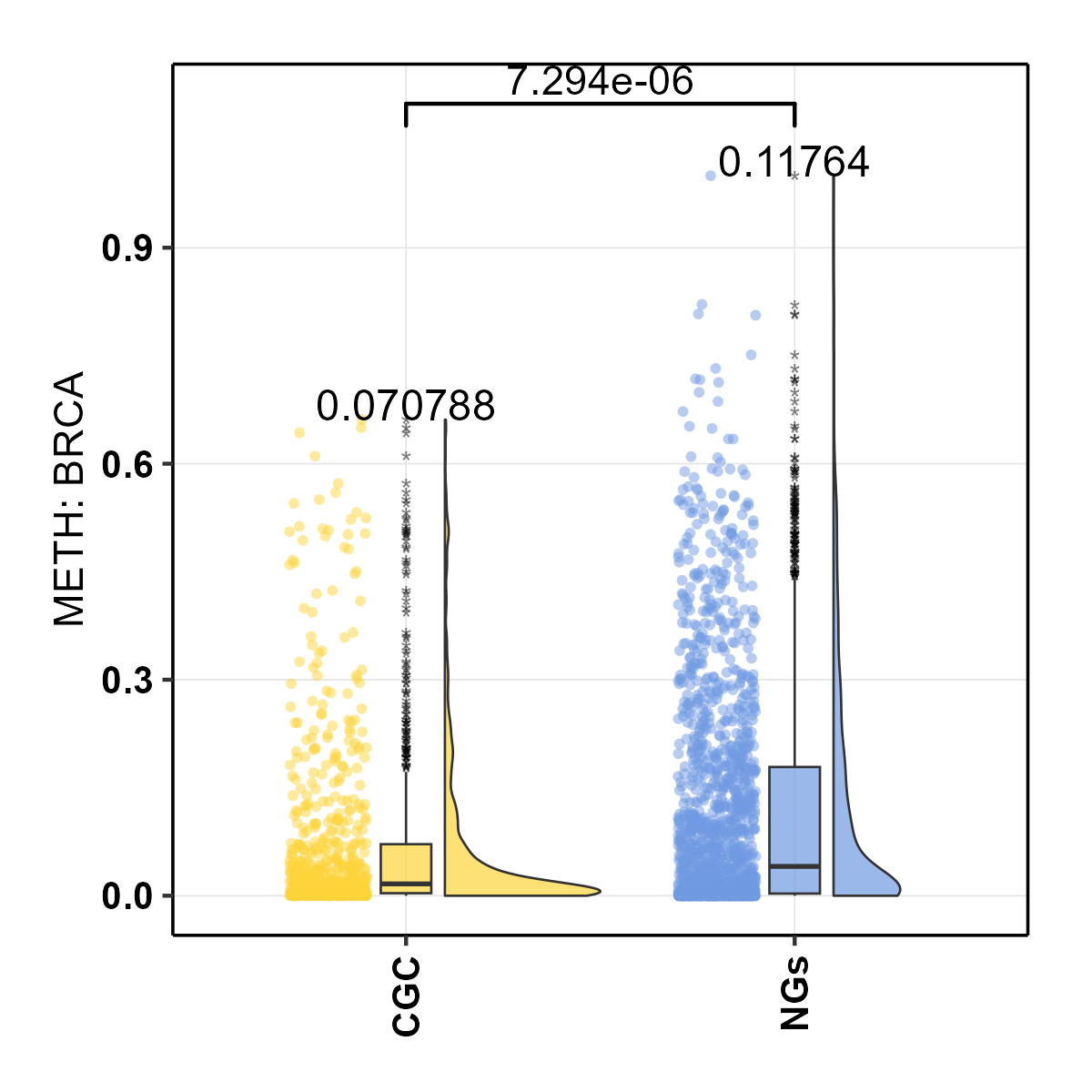

Supplement: Supplementary file 5 [file DataSheet2.ZIP › Supplementary file 5-2/STRINGdb/METH_BRCA.png]

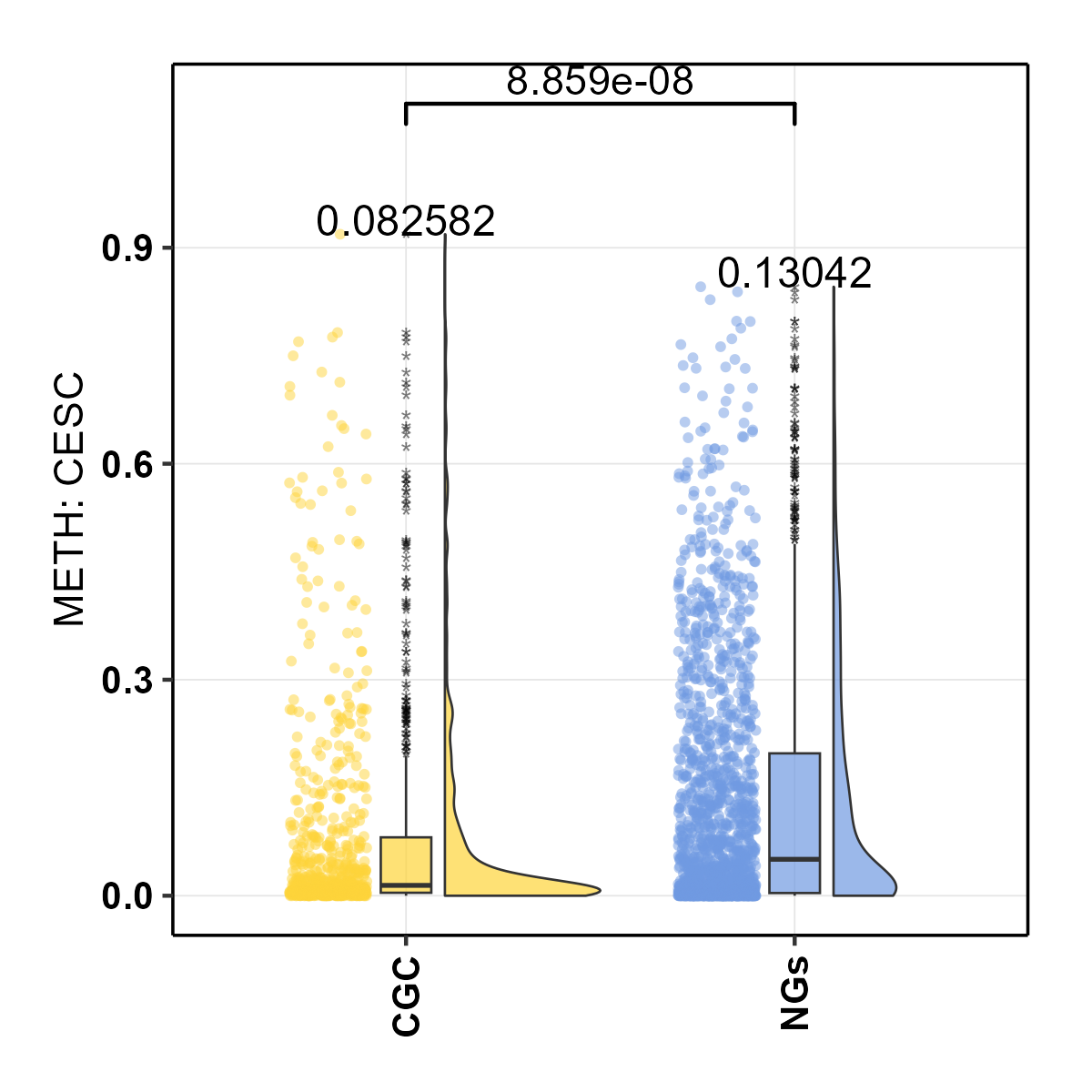

Supplement: Supplementary file 5 [file DataSheet2.ZIP › Supplementary file 5-2/STRINGdb/METH_CESC.png]

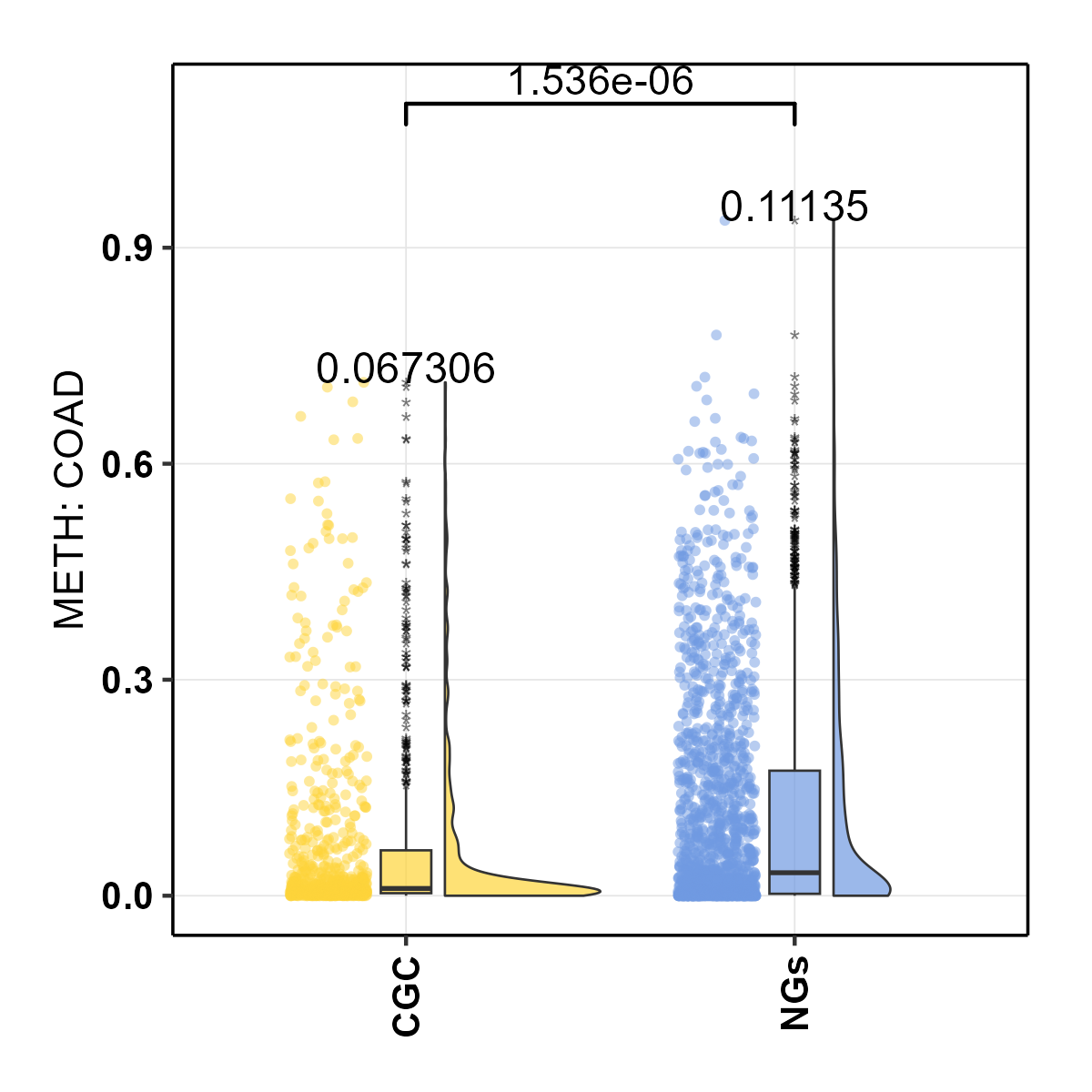

Supplement: Supplementary file 5 [file DataSheet2.ZIP › Supplementary file 5-2/STRINGdb/METH_COAD.png]

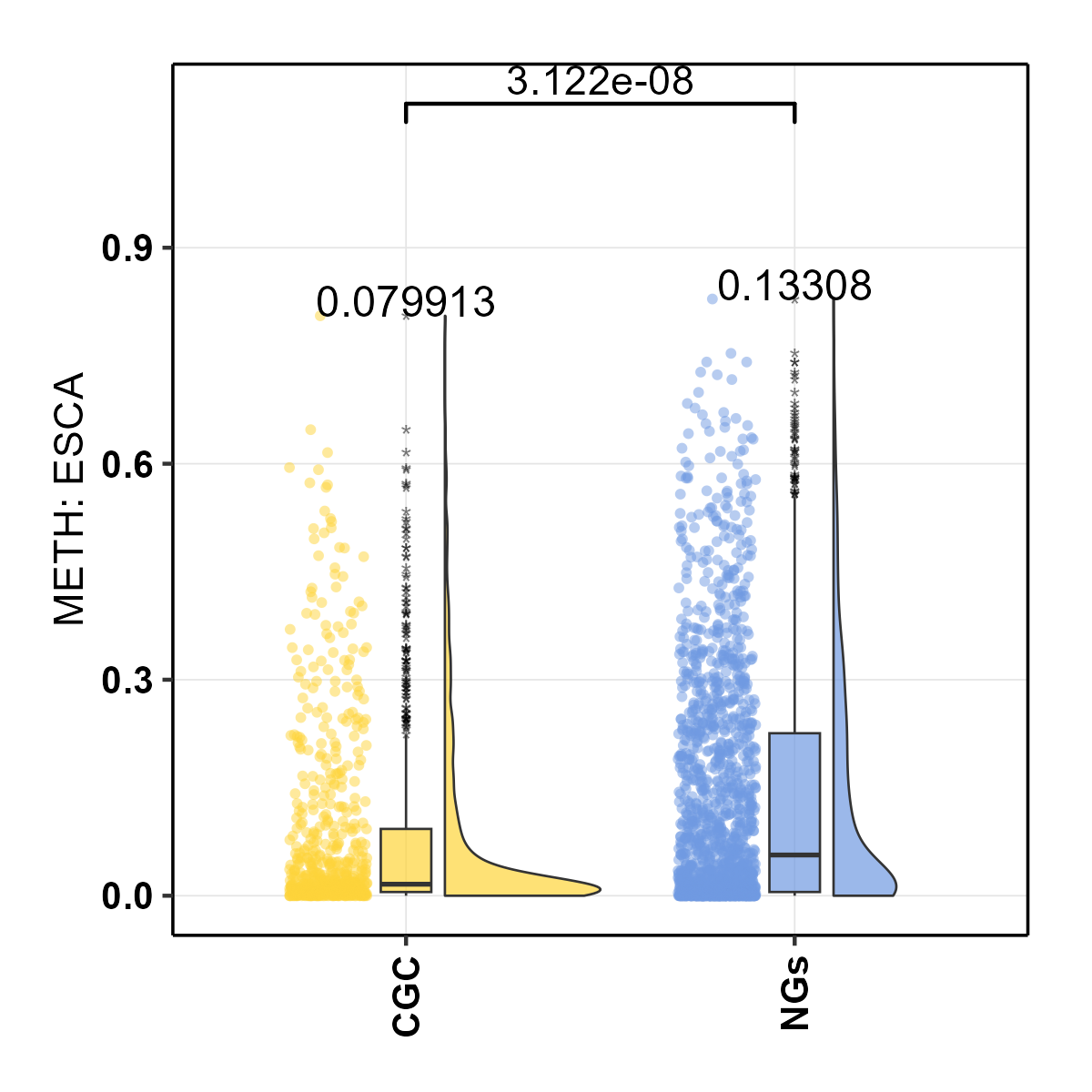

Supplement: Supplementary file 5 [file DataSheet2.ZIP › Supplementary file 5-2/STRINGdb/METH_ESCA.png]

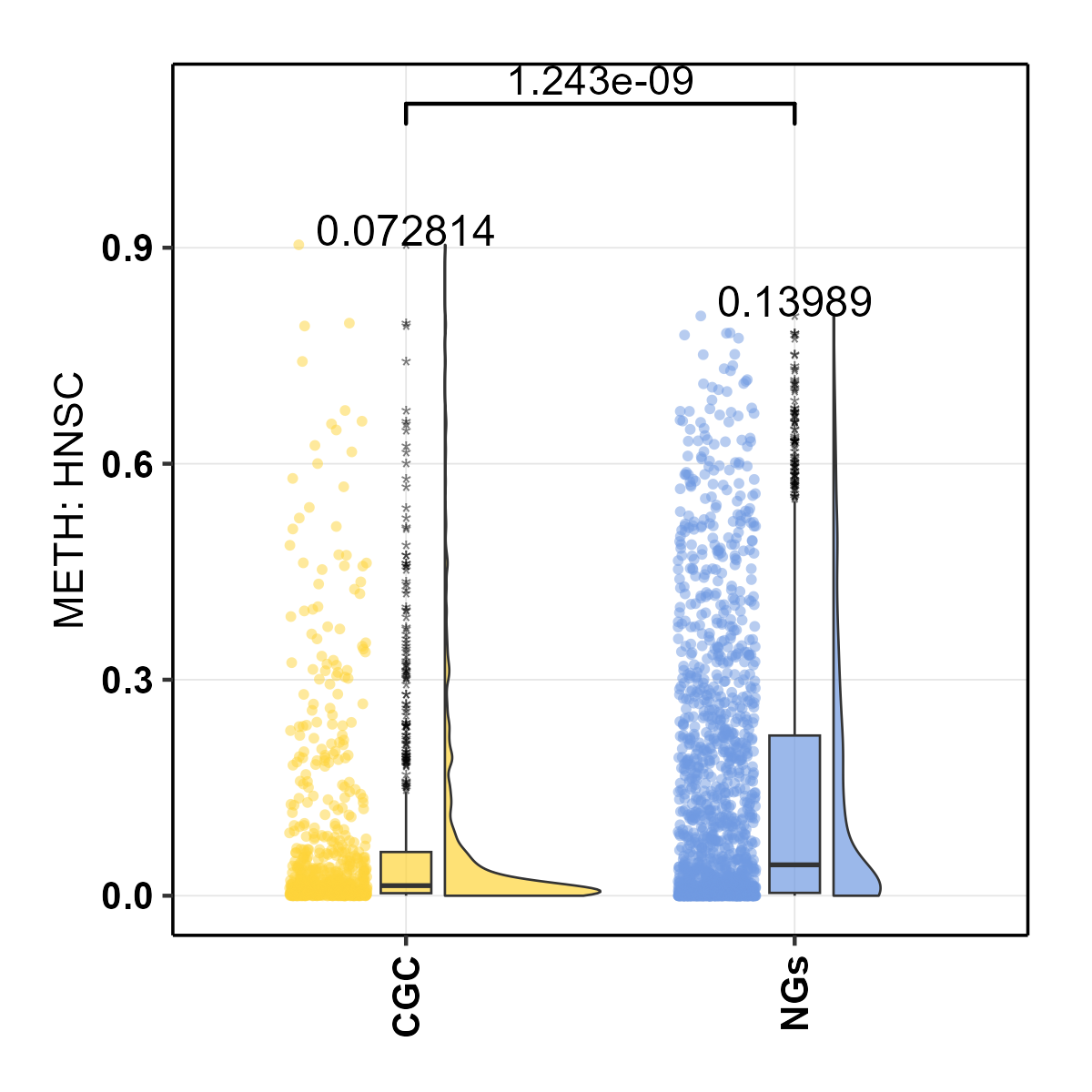

Supplement: Supplementary file 5 [file DataSheet2.ZIP › Supplementary file 5-2/STRINGdb/METH_HNSC.png]

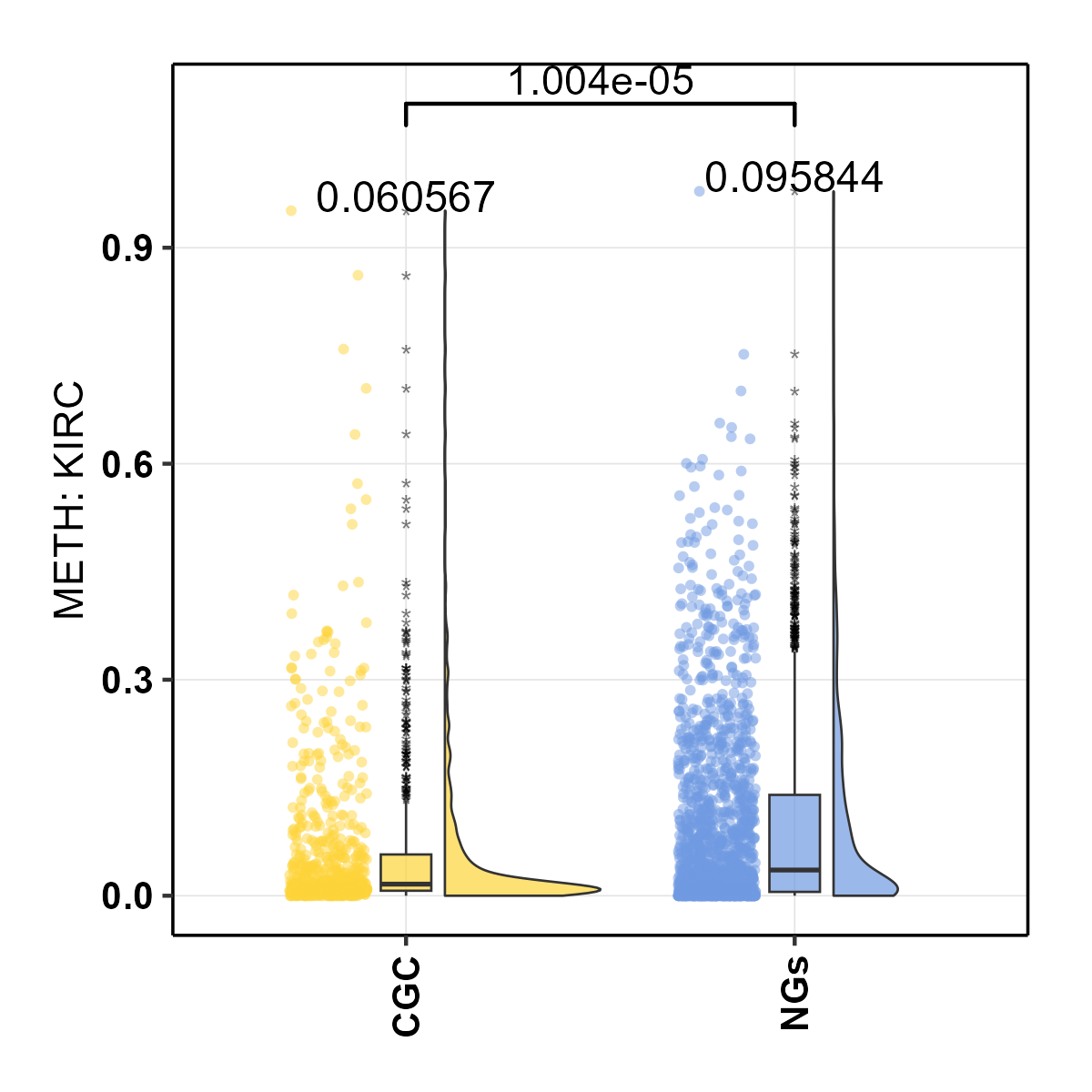

Supplement: Supplementary file 5 [file DataSheet2.ZIP › Supplementary file 5-2/STRINGdb/METH_KIRC.png]

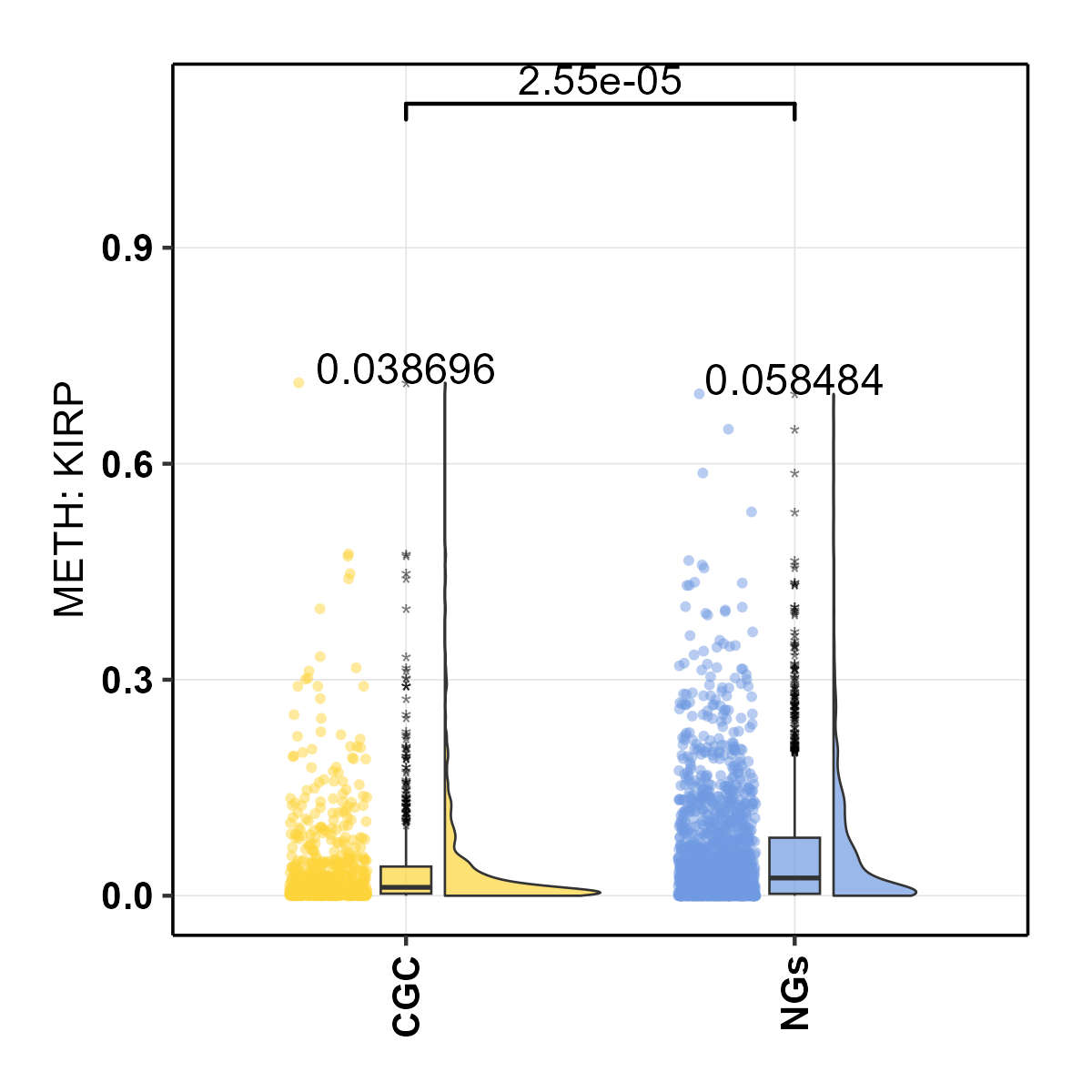

Supplement: Supplementary file 5 [file DataSheet2.ZIP › Supplementary file 5-2/STRINGdb/METH_KIRP.png]

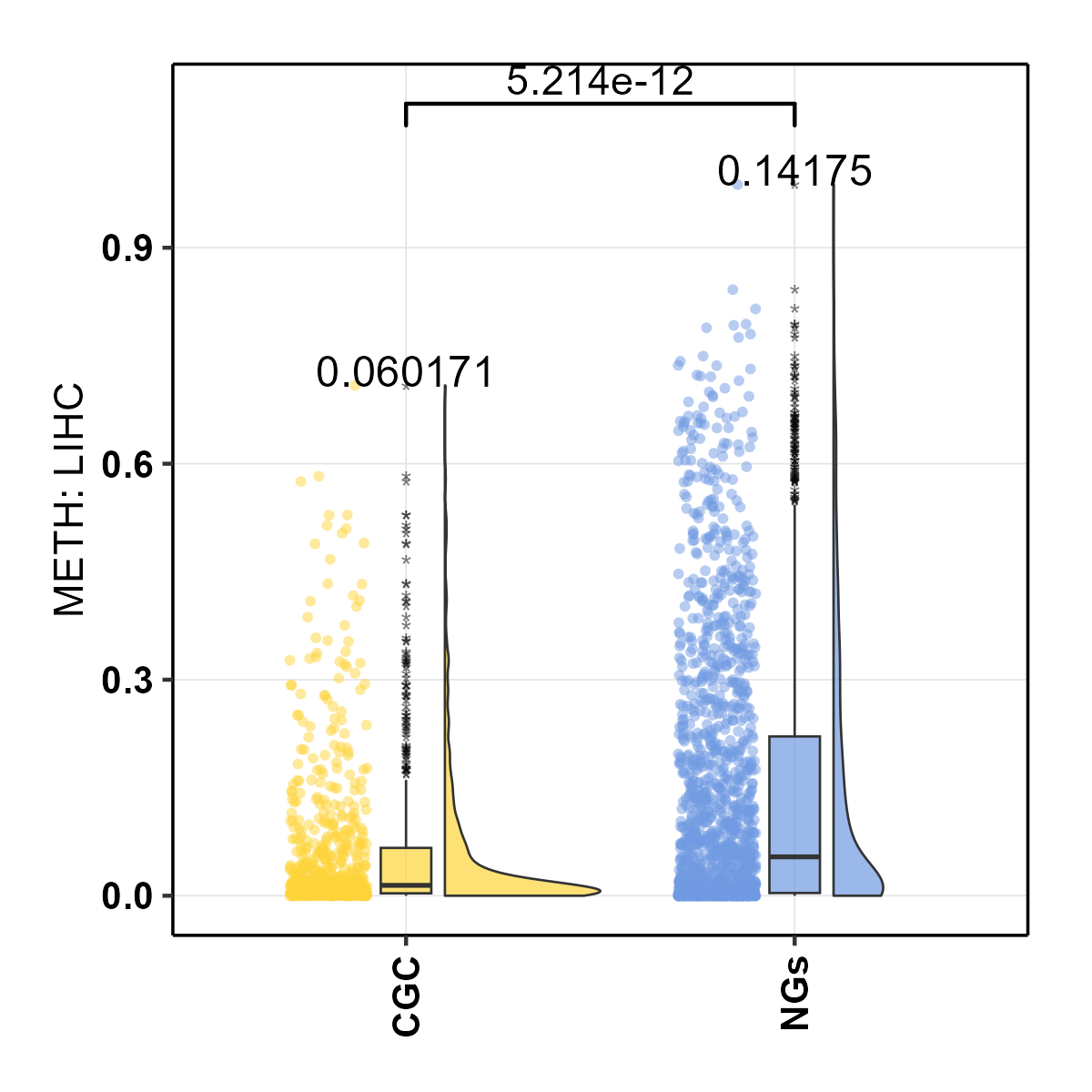

Supplement: Supplementary file 5 [file DataSheet2.ZIP › Supplementary file 5-2/STRINGdb/METH_LIHC.png]

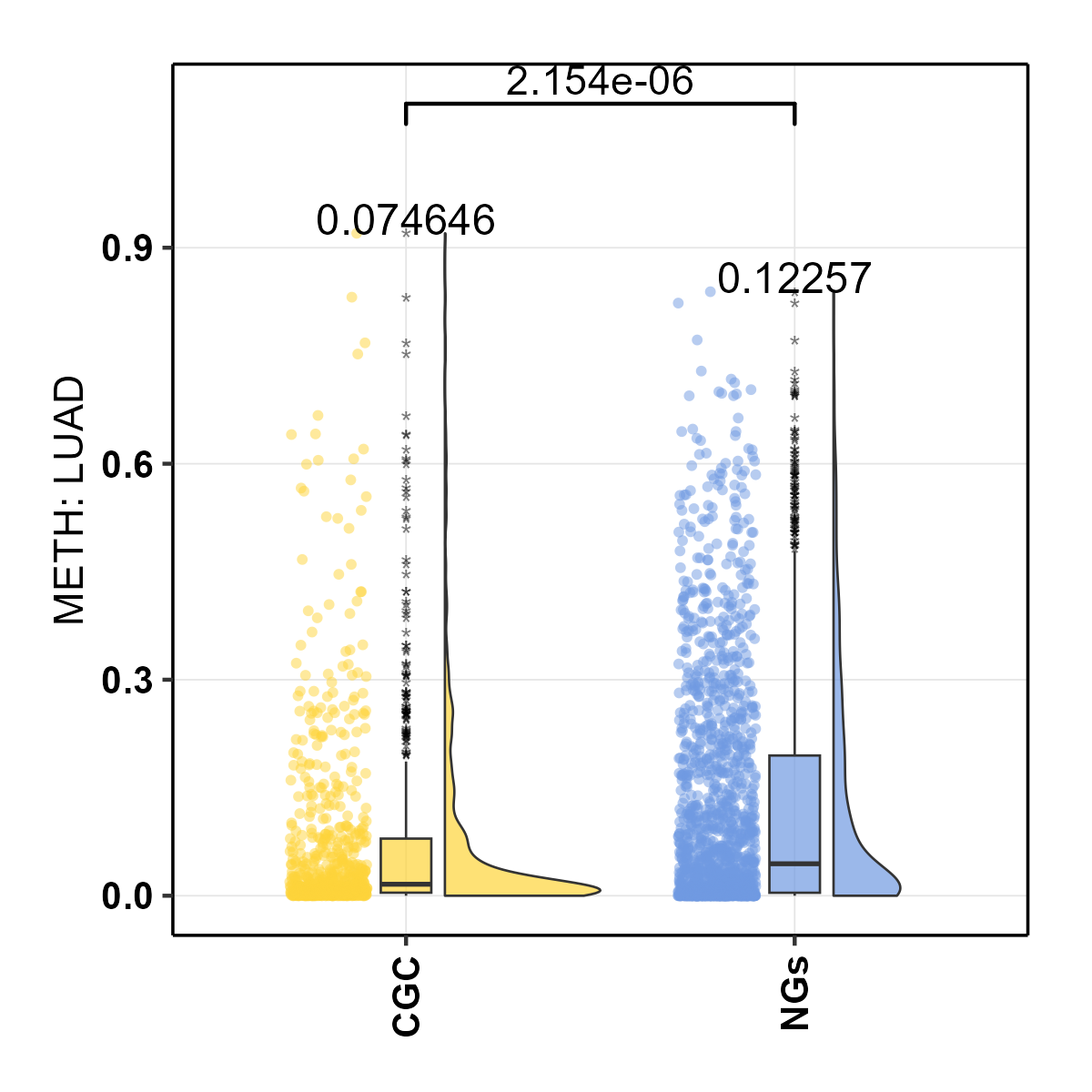

Supplement: Supplementary file 5 [file DataSheet2.ZIP › Supplementary file 5-2/STRINGdb/METH_LUAD.png]

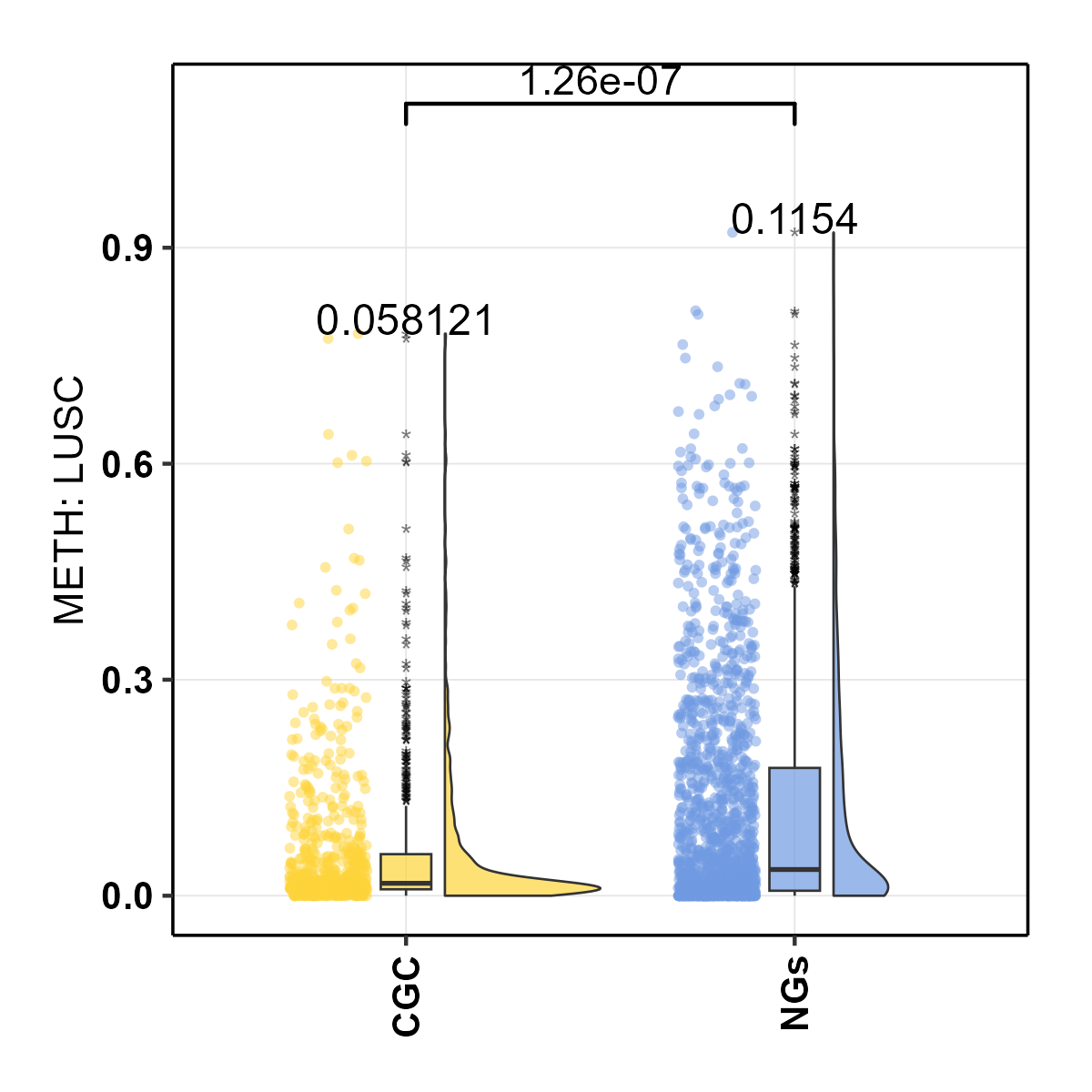

Supplement: Supplementary file 5 [file DataSheet2.ZIP › Supplementary file 5-2/STRINGdb/METH_LUSC.png]

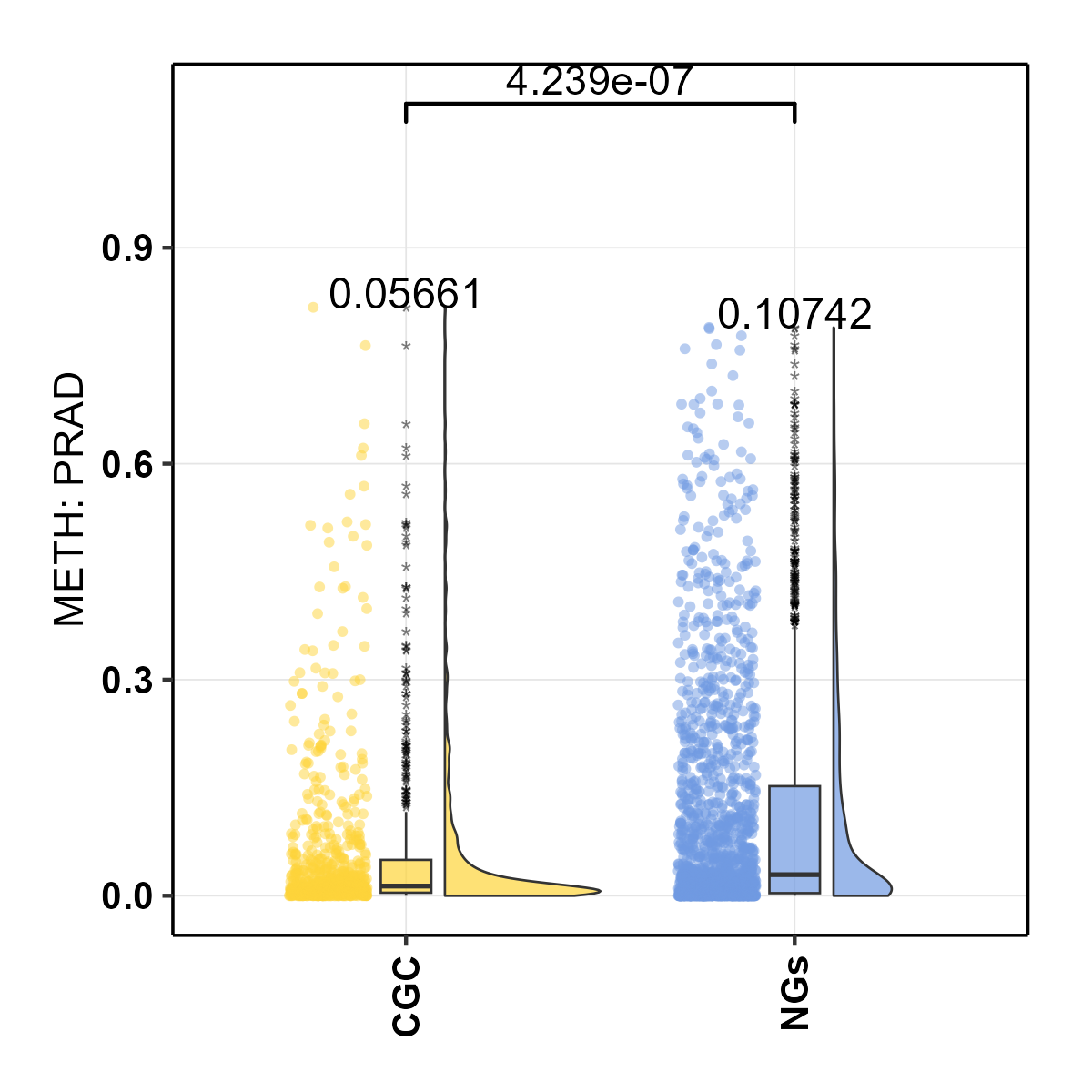

Supplement: Supplementary file 5 [file DataSheet2.ZIP › Supplementary file 5-2/STRINGdb/METH_PRAD.png]
